# Influence of Selective Deoxyfluorination on the Molecular Structure of Type-2 *N*-Acetyllactosamine

**DOI:** 10.1021/acs.joc.4c00879

**Published:** 2024-08-23

**Authors:** Martin Kurfiřt, Lucie Červenková Št’astná, Martin Dračínský, Radek Pohl, Ivana Císařová, Jan Sýkora, Martin Balouch, Michal Baka, Vojtěch Hamala, F. Javier Cañada, Ana Ardá, Jesús Jiménez-Barbero, Jindřich Karban

**Affiliations:** † Institute of Chemical Process Fundamentals, Czech Academy of Sciences, Rozvojová 1/135, CZ-165 00 Praha 6, Czech Republic; ‡ Institute of Organic Chemistry and Biochemistry, Czech Academy of Sciences, Flemingovo náměstí 542/2, CZ-160 00 Praha 6, Czech Republic; § Department of Inorganic Chemistry, Faculty of Science, Charles University in Prague, Hlavova 8, CZ-128 43 Praha 2, Czech Republic; ∥ Institute of Entomology, Biology Centre of the Czech Academy of Sciences, Branišovská 31, 370 05 České Budějovice, Czech Republic; ^⊥^Department of Organic Chemistry, ^#^Department of Analytical Chemistry, ^∇^Department of Chemical Engineering, and ^•^Department of Food Analysis and Nutrition, University of Chemistry and Technology, Prague, Technická 5, 166 28 Prague 6, Czech Republic; ° Centro de Investigaciones Biológicas Margarita Salas, Ramiro de Maeztu 9, 28040 Madrid, Spain; ¶ CICbioGUNE, Basque Research & Technology Alliance (BRTA), Bizkaia Technology Park, Building 800, 48162 Derio Bizkaia, Spain; △ Ikerbasque, Basque Foundation for Science, Plaza Euskadi 2, 48013 Bilbao Bizkaia, Spain; ▲ Department of Organic and Inorganic Chemistry, Faculty of Science and Technology, University of the Basque Country, EHU-UPV, 48940 Leioa, Spain; □ CIBER de Enfermedades Respiratorias (CIBERES), Avda Monforte de Lemos 3-5, 28029 Madrid, Spain

## Abstract

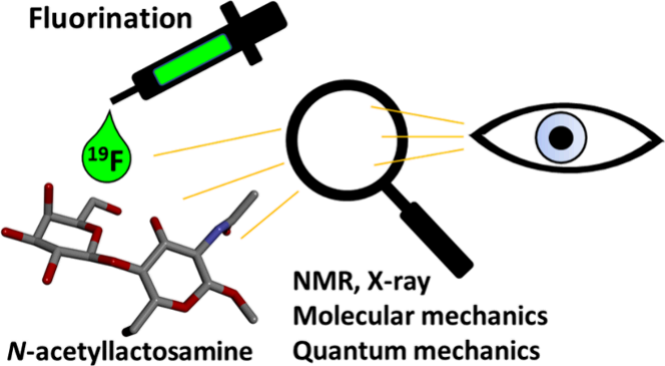

*N*-Acetyllactosamine is a common saccharide motif found in various biologically active glycans. This motif usually works as a backbone for additional modifications and thus significantly influences glycan conformational behavior and biological activity. In this work, we have investigated the type-2 *N*-acetyllactosamine scaffold using the complete series of its monodeoxyfluorinated analogs. These glycomimetics have been studied by molecular mechanics, quantum mechanics, X-ray crystallography, and various NMR techniques, which have provided a comprehensive and complete insight into the role of individual hydroxyl groups in the conformational behavior and lipophilicity of *N*-acetyllactosamine.

## Introduction

Oligosaccharides and polysaccharides, generally referred to as glycans^1^ in glycobiology, form a dense three-dimensional layer at the cell surface—glycocalyx—and they also occur abundantly in the extracellular matrix. Thanks to their enormous structural complexity and variability, glycans encode information that is read and translated by lectins and other glycan-binding proteins.^2,3^ To understand the molecular basis of protein−glycan recognition and exploit this understanding in the design of lectin-targeting therapeutics, glycochemists routinely employ synthetically modified glycans or glycomimetics.^4−6^ Conformational preferences of both natural and glycomimetic lectin ligands have a great impact on the binding energetics of glycan−protein interactions, because the nature of the glycosidic linkage provides most glycans with a significant degree of flexibility, resulting in highly dynamic conformational equilibria in solution, whereas only a limited subset of conformers is found in glycan−protein complexes.^7,8^ Indeed, the improvement of the conformational preorganization of a ligand to increase its inhibitory potency is an important factor in the design of glycomimetics.^9^ It is much more difficult to study the conformational behavior of glycans than of other biomolecules. X-ray crystallography is of limited informative value because the high flexibility of glycans complicates the formation of crystals suitable for a single-crystal X-ray diffraction analysis.^10^ NMR spectroscopy is very useful, but its routine application is hampered by severe degeneration of chemical shifts in glycans, which in turn requires sophisticated NMR techniques, the use of isotopically labeled glycans or an insertion of a paramagnetic tag.^11,12^

Over the last decades, the incorporation of fluorine has consistently belonged to the most common strategies in the development of drugs, agrochemicals and other bioactive compounds for research or industry. The replacement of an OH group with fluorine (deoxyfluorination) has served many purposes in the design of glycomimetics.^13^ A deoxyfluorinated carbohydrate shows a negligible steric deviation from the parent compound because the fluorine atom and the hydroxyl group have a comparable size and electronegativity, and the C−F and C−OH bonds are similar in terms of their length and polarity.^13^ However, deoxyfluorination removes the hydrogen-bond-donating capacity of the deoxyfluorinated position, reduces its hydrogen-bond-accepting ability, influences the electron density of adjacent atoms and reduces the polar surface area. All of these effects can, in principle, influence the conformational behavior of a deoxyfluorinated glycan. In general, deoxyfluorination has a minimal impact on the ring conformation of hexopyranoses or their simple glycosides as they retain their usual ^4^*C*_1_ conformation.^14^ The exocyclic fluoromethyl group tends to adopt the *gg* conformation in gluco-configured fluoroanalogs,^15,16^ while the *gt* conformer has been proposed as the most populated for galacto-configured molecules.^16−19^ In contrast to deoxyfluorinated monosaccharides, the conformational behavior of deoxyfluorinated oligosaccharides has been rarely investigated, which in part results from challenges associated with the synthesis of a set of deoxyfluorinated glycans.

**Figure 1:**
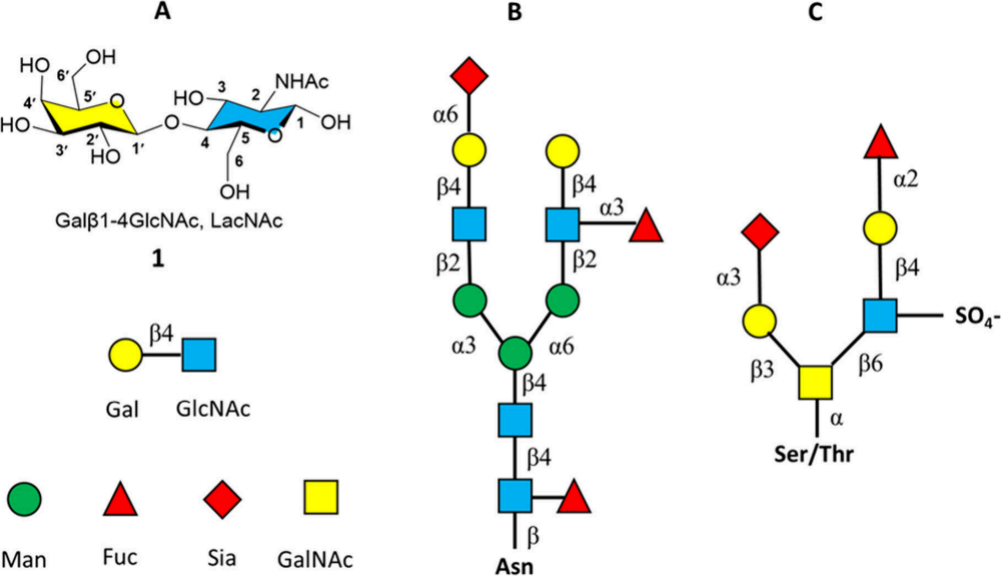
A) The structure of *N*-acetyllactosamine **1** with atom numbering and its representation according to the symbol nomenclature for glycans (SNFG).^28^ B) An example of a complex *N*-glycan on mature glycoproteins containing two LacNAc units.^20^ C) An extended core 2 *O*-GalNAc glycan from human respiratory mucins containing one LacNAc unit.^21^

LacNAc **1**, also termed as type-2 *N*-acetyllactosamine (Galβ1−4GlcNAc, Figure 1A), is a biologically highly relevant disaccharide and a ubiquitous building block of various mammalian N- and O-linked glycans.^20,21^ In N-glycans, the LacNAc motif usually elongates the branching mannoses of the pentasaccharide N-glycan core (Figure 1B), whereas in O-glycans, it is frequently attached to GalNAc or Gal, forming a part of the extended O-glycan cores (Figure 1C). In both of these glycan types, the LacNAc motif can be repeated, forming an oligo- or poly-*N*-acetyllactosamine chain, which functions as a backbone for additional glycan modifications. Therefore, the type-2 LacNAc is a part and precursor of several glycan-terminating structures, including Lewis blood-group determinants Lewis X, sialyl-Lewis X, and Lewis Y, and type-2 human blood groups H, A and B,^22^ which are involved in processes such as pathogen adhesion,^23^ the spread of metastases, or fertilization.^24^ The LacNAc motif itself acts as a binding ligand for various carbohydrate-binding proteins such as the plant lectins isolated from *Solanum tuberosum* and *Ricinus communis,*(25) or, importantly, for galectins,^26^ which participate in tumor-associated or immune-system-related processes in humans. Moreover, LacNAc and its related structures can also be found in human milk oligosaccharides (HMOs), where they show protective and prebiotic effects.^27^

**Figure 2:**
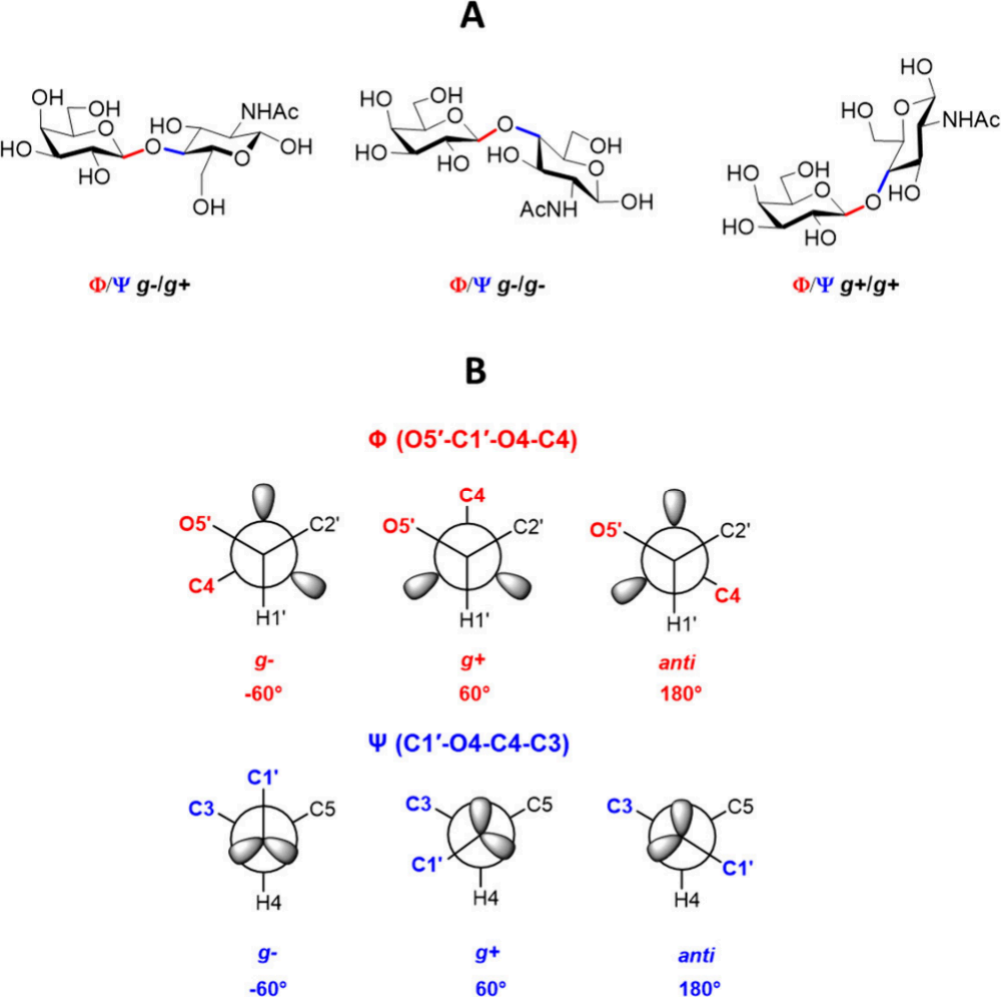
A) The typical glycoside linkage conformational minima of the LacNAc system Φ/Ψ *g−*/*g+*, *g−*/*g−* and *g+*/*g+*. B) The definition of the torsions Φ and Ψ, based on heavy atoms, and the corresponding terminology of the ideal staggered Φ and Ψ rotamers.

Because of the biological relevancy of LacNAc, detailed knowledge of its structure is of high importance. Diverse studies have addressed the conformational behavior of LacNAc **1**(29,30) and related β-1,4-linked disaccharides such as lactose (Galβ1−4Glc),^17,31−33^ cellobiose (Glcβ1−4Glc)^34−36^ and chitobiose (GlcNAcβ1−4GlcNAc).^37−40^ These studies are in general agreement that β-1,4-linked disaccharides predominantly adopt the Φ/Ψ *g−*/*g+* conformation of the glycosidic linkage accompanied by minor *g−*/*g−* and *g+*/*g+* conformers (Figure 2).^41^ These conformational minima are mostly governed by stereoelectronic interactions^42^ and hydrogen bonds.^43,44^

**Figure 3:**
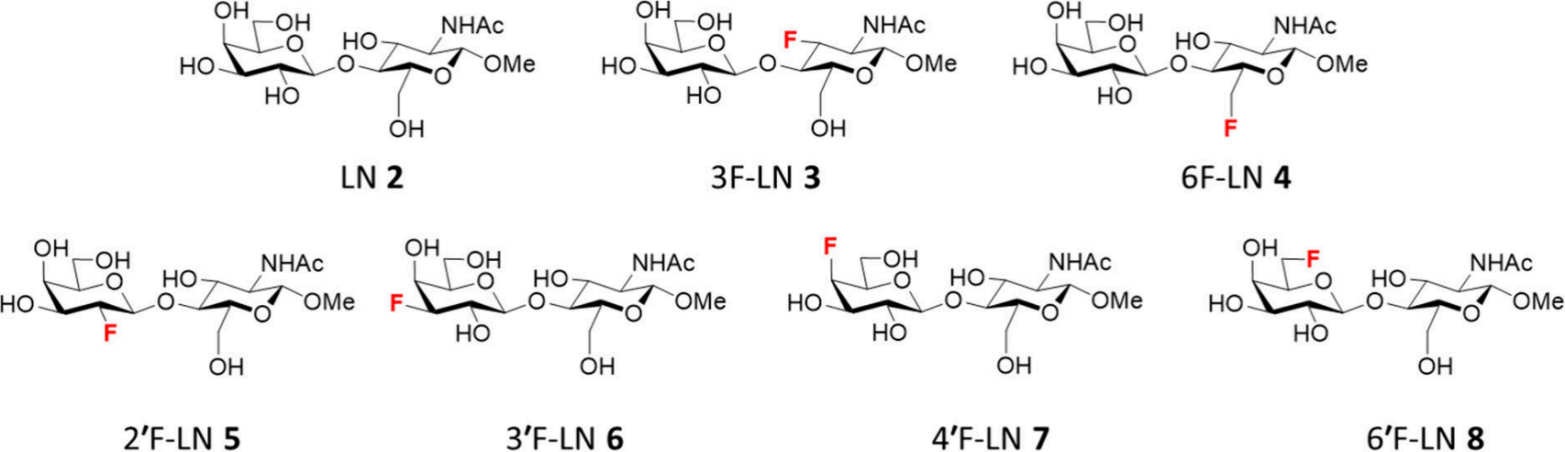
Structures of the LacNAc analogs (LNs) **2**−**8** investigated in this study.

In this work, we aspire to increase the knowledge of the behavior of LacNAc-derived glycomimetics by studying the influence of monodeoxyfluorination on its conformational states and lipophilicity. To fulfill such aspiration, we have employed a complete series of the systematically monodeoxyfluorinated LacNAcβ1-OMe (LN, **2**) analogs **3**−**8** (Figure 3), which have recently been prepared in our laboratory.^45^ The methyl β-glycosides (LNs) have been selected for their synthetic accessibility and the presence of β-glycosidic linkage in the repeating poly-*N*-acetyllactosamine chains of natural glycans. The LNs **2**−**8** have been studied by a combination of computational and NMR approaches utilizing ^13^C chemical shifts, *J*-coupling constants, NOE/ROE correlations and ^1^H NMR temperature dependences of exchangeable protons. The series has also been studied by single-crystal X-ray diffraction analysis, as we succeeded in obtaining suitable crystals for all deoxyfluorinated LacNAc analogs. The combination of these techniques made it possible to decipher the influence of the systematic monodeoxyfluorination on the conformational behavior of the glycosidic linkage, exocyclic groups at C6/6′ and the acetamido group at C2. The stablest conformers of the compounds **3**−**8** were subsequently utilized for the calculation of their 1-octanol/water partition coefficients (log *P*) using the COSMO-RS methodology,^46,47^ which showed excellent agreement with experimental data.

## Results and Discussion

### Molecular Mechanics

**Figure 4:**
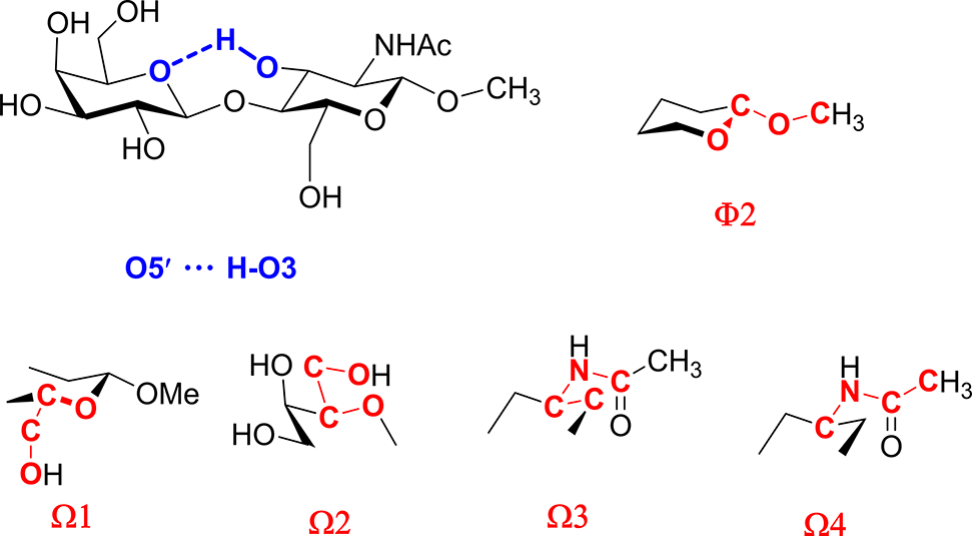
Spatial structure of the LN **2**, showing the inter-residue O5′···H−O3 hydrogen bond and the torsions Φ2 and Ω1−Ω4.

Initially, the conformational behavior of the LNs **2**−**8** has been investigated by systematic relaxed potential energy scans of two glycosidic-bond dihedral angles Φ (O5′−C1′−O4−C4) and Ψ (C1′−O4−C4−C3) using molecular mechanics (Figures S1−S7).^17,31,48^ The LNs **2** and **4**−**8** showed highly similar Φ/Ψ adiabatic population diagrams with a high occupancy of the Φ/Ψ *g−*/*g+* region, covering 2.05−2.70% of Φ/Ψ conformational space at the 90% probability level (Table 1). In contrast, 3F-LN **3** covers approximately 4.4% of conformational space and exhibits a high population of conformers outside the defined regions (Table 1), predicting its higher conformational flexibility around the glycosidic linkage. Such different computational results probably reflect the disruption of the inter-residue O5′···H−O3 hydrogen bond (Figure 4), commonly found in β-1,4-linked disaccharides.^29,38,43,44,49^

### X-ray Crystallography

We have succeeded in obtaining crystals of the disaccharides **3**−**8** suitable for X-ray diffraction analysis (Figures S8−S13). As expected, the LNs **3**−**8** crystallized in the Φ/Ψ *g−*/*g+* conformation in accordance with the global minimum found in molecular mechanics, corresponding to the crystal structure of the parental nonfluorinated LacNAc **1**.^29^ To gain a clear insight into the effects of individual fluorine incorporations, we compared the lengths of inter-residue O5′···H−O3 hydrogen bonds and the dihedral angles Φ, Ψ, Φ2, Ω1, Ω2, Ω3 and Ω4 (Figures 2, 4 and Table 2). The dihedral angle Φ2 [O5−C1−O−CH_3_] describes the conformation of the OMe glycosidic linkage, the torsions Ω1 [O5−C5−C6−O6] and Ω2 [O5′−C5′−C6′−O6′] describe the orientation of exocyclic hydroxyls, and the remaining torsions Ω3 [C1−C2−N−C] and Ω4 [C2−N−C−C] define the conformation of the acetamido group.

**Figure 5:**
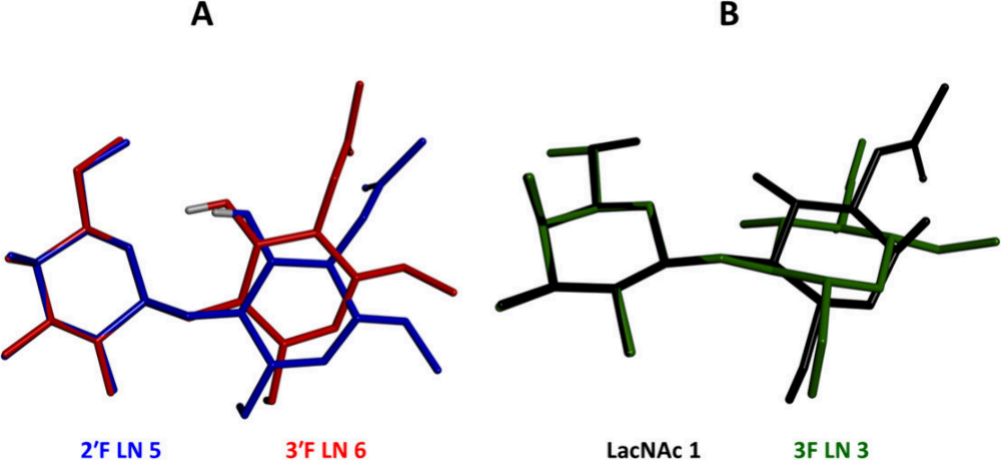
Overlay of the crystal structures A) 2′F-LN **5** and 3′F-LN **6**; B) α-d-LacNAc **1** and 3F-LN **3**.

Interestingly, the compounds **4**−**7** showed analogous trends in the length of the O5′···H−O3 hydrogen bond and the glycoside torsions Φ, Φ2 and, with minor exceptions, also Ψ (Table 2). Compared to α-d-LacNAc^29^ (O5′···O3 = 2.79 Å), 2′F-LN **5** and 6F-LN **4** showed longer O5′···O3 distances, namely 3.04 and 3.02 Å, respectively. This trend seems to indicate that the deoxyfluorination of the positions 2′ and 6 somehow weakens the inter-residue hydrogen bond. It is tempting to speculate that the longer O5′···O3 distances are related to an increase in flexibility around the glycosidic linkage. In contrast, the fluorination of the 3′-position (**6**) and 6′-position (**8**) has significantly decreased the O5′··· O3 distance to 2.68 and 2.71 Å, respectively. Again, it is tempting to speculate that this shortening of the O5′···O3 distance indicates some rigidification of the glycosidic linkage. 4′F-LN (**7**) has shown approximately the same O5′··· O3 distance as **1** (2.82 Å), which implies that 4′-fluorination has not influenced the glycoside conformational behavior. The significant variations between the three-dimensional structures of the limiting compounds 2′F-LN **5** and 3′F-LN **6** are illustrated by their overlay, as depicted in Figure 5A.

**Figure 6:**
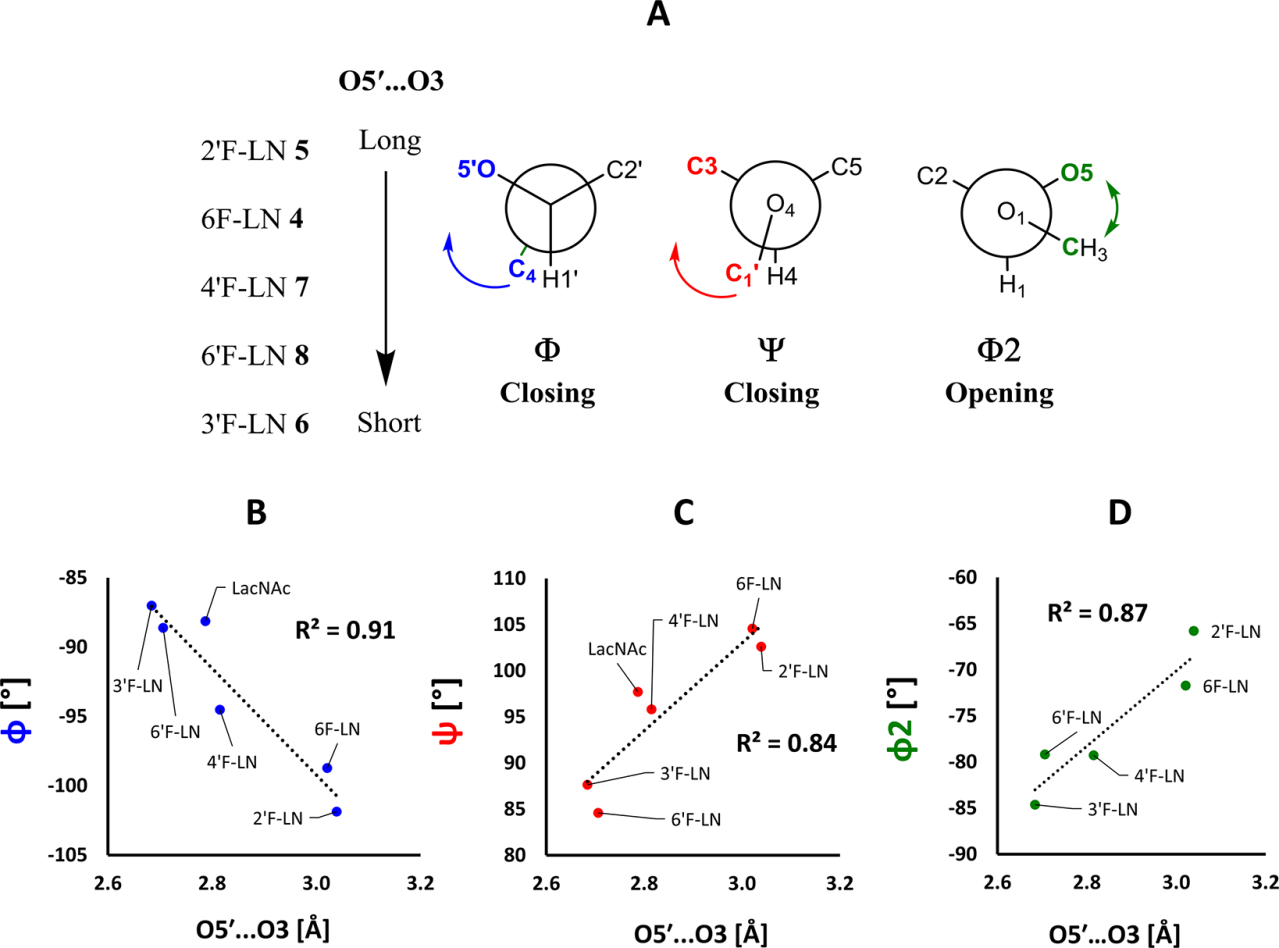
A) A summary of the changes of the torsions Φ, Ψ and Φ2 in the series **4**−**8**, with the decreasing O5′···O3 distance. B)−D) Correlations between Φ, Ψ and Φ2 torsions and the O5′···O3 distance.

In the series **5** → **4** → **7** → **8** → **6**, the absolute values of Φ torsions gradually decreased, which can be illustrated as a dihedral closing (Figure 6A). This closing significantly correlated with the decreasing O5′···O3 distance (R^2^ = 0.91, Figure 6B), indicating the interdependence of these two molecular descriptors. Similarly, Ψ torsion exhibited a gradual decrease with the decreasing O5′···O3 distance (Figure 6A), also with a high coefficient of determination (R^2^ = 0.84, Figure 6C). Surprisingly, the absolute value of Φ2 torsion also showed a gradual increase in the series **5** → **4** → **7** → **8** → **6**, represented by its opening (Figure 6A), strongly correlating with the O5′···O3 distance (R^2^ = 0.87, Figure 6D). Therefore, we may hypothesize that the conformation around the glycosidic linkage in β-1,4-linked oligosaccharides is somehow influenced by the modulation of the O5′···H−O3 hydrogen bond strength of a neighboring disaccharide unit. In 3F-LN **3**, the value of Ψ was the lowest in the series **3**−**8**. This exceptional behavior of 3F-LN **3** in comparison with the remaining compounds illustrates that the precise geometry within the Φ/Ψ *g−*/*g+* conformational region of LacNAc is somehow modulated by the disruption of the O5′···H−O3 hydrogen bond. The effect of 3-fluorination can be illustrated by the overlay of the crystal structures of 3F-LN **3** and LacNAc **1** (Figure 5B), which shows a change of the relative position of the *gluco*- and *galacto*-configured rings upon fluorination.

**Figure 7:**
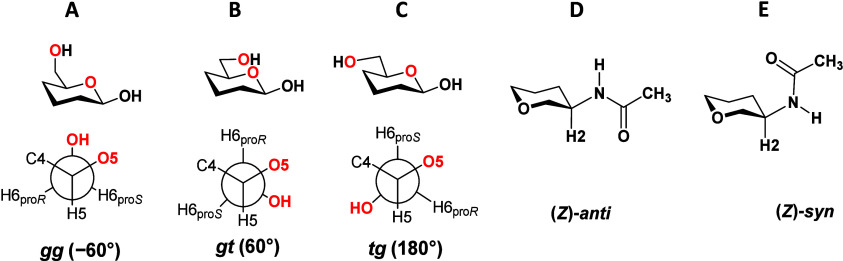
A)−C) Three possible staggered conformers, *gg*, *gt* and *tg,* of the exocyclic hydroxymethyl group in hexopyranoses according to the IUPAC rules.^51^ D)−E) The definition of two possible conformations, (*Z*)-*anti* and (*Z*)-*syn*, on the acetamido group.

The torsion Ω1, describing the conformation of the exocyclic 6-hydroxymethyl/fluoromethyl groups in the GlcNAc rings, was independent of the deoxyfluorination pattern, with values being close to −60° (Table 2 and Figure 7A, *gg* conformation). Similarly, the torsion Ω2, describing the conformation of the exocyclic 6′-hydroxymethyl/fluoromethyl groups in the Gal moieties, showed similar values, close to 60° (Table 2 and Figure 7B, *gt* conformation) in compounds **4**−**8**. Intriguingly, 3F-LN **3** had Ω2 = 173°, which is dramatically different from those of the remaining disaccharides and close to the staggered *tg* orientation (Table 2, Figures 5B and 7C). Although the crystallization of **3** as the 6′-*tg* conformer could be accidental because both *gt* and *tg* rotamers are present in Gal moieties in solution, the increased preference for the 6′-*tg* conformation was independently determined by NMR in DMSO-*d*_6_ solution, discussed in the next section. The values of the remaining torsions, Ω3 and Ω4, were consistent with the (*Z*)-*anti* conformation of the acetamido group (Figure 7D), which is regarded as stabler than the second possible (*Z*)-*syn* conformation (Figure 7E).^50^

### NMR Analysis

To understand the behavior of the LNs **2**−**8** in solution, we performed a quantum-mechanics-assisted NMR analysis. The analysis was based on the evaluation of chemical shifts, indirect *J*-coupling constants, qualitative NOEs (ROEs), and the temperature dependence of the chemical shifts of exchangeable protons.^52^ This investigation was carried out in DMSO-*d*_6_ solution as it enables facile observation of exchangeable protons.

### Chemical Shifts of Skeletal Protons and Carbons

Deoxyfluorination shifted the geminal protons by approximately 1 ppm downfield and the directly attached ^13^C carbons by more than 20 ppm downfield in all cases (Table S2, green cells). Similarly, vicinal protons separated from the fluorine atom by three single bonds were shifted downfield by 0.18−0.41 ppm. (Table S2A, blue cells). Conversely, ^13^C carbons separated from the fluorine by two or three bonds were generally shifted upfield by 0.3−5.9 ppm, although there were a few exceptions of a minor downfield shift by 0.1−0.7 ppm (Table S2B, blue cells). These observations are also in agreement with the data reported for other fluorinated sugars.^53,54^

**Figure 8:**
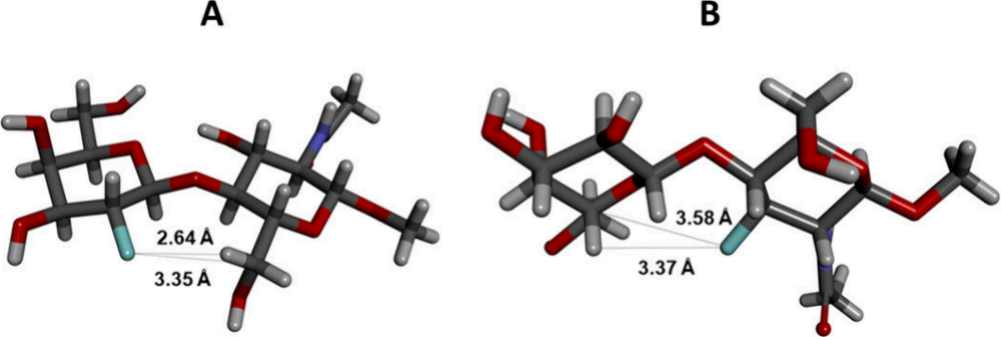
A) The crystal structure of 2′F-LN **5** with a depiction of the F···H6_pro*R*_/C6 distances. B) The crystal structure of 3F-LN **3** with a depiction of the F···H5′/C6′ distances.

Interestingly, both 2′F-LN **5** and 3F-LN **3** showed selective ^1^H and ^13^C long-range fluorine-induced perturbation of chemical shifts (Δδ Table S2, yellow cells). In **5**, the protons H6_pro*R*_ and H6_pro*S*_, separated from 2′-fluorine by seven bonds, were shifted upfield by 0.07 and 0.09 ppm, respectively. No other proton in **5**, separated from fluorine by six (H6′_pro*R*_, H6′_pro*S*_, H3, H5) or seven bonds (H2), showed Δδ of such magnitude. Additionally, the carbon C6 exhibited an upfield shift by 0.7 ppm, the highest value among the carbons separated from 2′-fluorine by five or six bonds. These long-range Δδs indicate that the fluorine at the 2′-position influences the carbon and protons at the 6-position via a through-space effect. This conclusion is supported by the relatively short distance between the fluorine atom and H6_pro*R*_ (2.64 Å) or C6 (3.35 Å) in the X-ray structure of 2′F-LN **5** (Figure 8A). Furthermore, we have detected a through-space 2′-fluorine−carbon C6 coupling in the disaccharide 2′F-LN **5**. This coupling has been described in detail elsewhere.^55^ Similarly, 3F-LN **3** has shown a significant long-range Δδs of H5′ and C5′, also associated with a short distance between the 3-fluorine nucleus and H5′ (3.37 Å) or C5′ (3.58 Å) in the crystal structure of 3F-LN **3** (Figure 8B). Therefore, these observed long-range Δδs in **5** and **3** are in accordance with the preference for the Φ/Ψ *g−*/*g+* conformation in DMSO-*d*_6_ solution.

### ^13^C NMR Chemical Shift-Based Conformational Analysis

**Figure 9:**
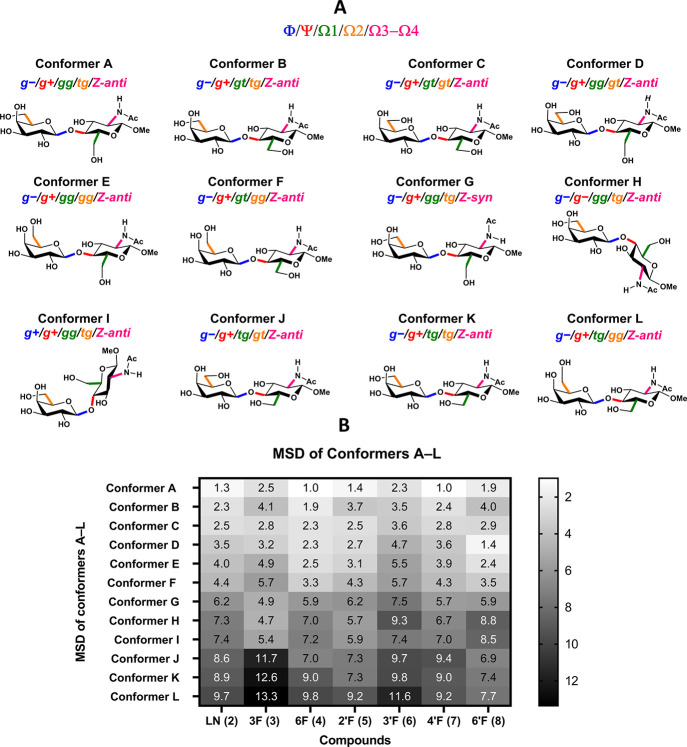
A) The structures of the conformers **A**−**L**, represented by the LN **2**. B) The mean-square deviations (MSDs) between calculated and experimentally obtained ^13^C NMR shifts for the conformers **A**−**L** of **2**−**8**. The conformers H and I of 6′F-LN **8** represent the only exception from the series, as their 6′-hydroxymethyl conformation was set to *gt*. *t.*

To support the interpretation of the NMR data, the geometries of the major conformational minima of the LNs **2**−**8** were optimized by means of density functional theory (DFT) to generate the conformers **A**−**L** (Figure 9A). These geometries had been selected to probe the conformations around the glycosidic linkages, the exocyclic hydroxyls, and the acetamido moieties systematically. The comparison of the zero-point-corrected free energies (Figures S14 and S15) had been identified as inappropriate for conformational analysis because of the tendency of DFT methods to overestimate the strength of hydrogen bonds.^56,57^ Therefore, the conformational analysis was based on the correlation between the DFT-calculated and the experimentally obtained NMR parameters (Tables S14−S20). Initially, we focused on ^13^C chemical shifts given their sensitivity to the conformational changes of the glycosidic linkage and the exocyclic CH_2_OH/F groups, and their relative insensitivity to subtle alterations in the orientation of exchangeable protons.^58−60^ The deviations between a set of experimental and calculated ^13^C chemical shifts were expressed as a mean-square deviation (MSD, Figure 9B).

Generally speaking, the calculated ^13^C NMR chemical shifts of the conformer Φ/Ψ *g−*/*g+* (**A**) showed significantly better agreement (lower MSDs) with the experimentally obtained ^13^C NMR shifts than those predicted for the conformers Φ/Ψ *g−*/*g−* (**H**) and Φ/Ψ *g+*/*g+* (**I**). This trend in MSDs confirms the preference for the Φ/Ψ *g−*/*g+* glycoside conformation in all compounds in accordance with X-ray analysis and the reported data.^17^ Moreover, as expected, the predicted ^13^C NMR shifts of the 6-*tg* conformers **J**, **K** and **L** exhibited significantly worse agreement (higher MSDs) with the experimental ^13^C NMR shifts than those of the 6-*gg* conformers **A**, **D** and **E**, and the 6-*gt* conformers **B**, **C** and **F**. This observation further confirms the general preference for the 6-*gg* and 6-*gt* orientations over the 6-*tg* orientation of the GlcNAc exocyclic CH_2_OH group.^17,18^ Similarly, the 6′-*gg* conformers (**E** and **F**) exhibited higher MSDs than the 6′-*tg* (**A** and **B**) and 6′-*gt* (**C** and **D**) rotamers, also in accordance with the expectations for Gal moieties.^17,18^ The acetamido group showed a preference for the (*Z*)-*anti* conformation (**A**) over the (*Z*)-*syn* conformation (**G**) in accordance with the X-ray analysis and reported data for acetamide sugar moieties.^50^

Apart from these general trends, the methodology enabled us to identify subtle differences in the conformational behavior of the LNs **2**−**8**, caused by selective deoxyfluorination. The fluorinated disaccharides 6F-LN **4**, 2′F-LN **5** and 4′F-LN **7** provided comparable MSDs in all the conformers **A**−**L** as LN **2**, indicating a minimal impact of the fluorination of these positions on the conformational behavior. In comparison with the LN **2**, 3F-LN **3** showed higher MSD values of the conformers Φ/Ψ *g−*/*g+* (**A**−**F)**, and lower MSD values of the conformers Φ/Ψ *g−*/*g−* (**H**) and Φ/Ψ *g+*/*g+* (**I**), which is consistent with the 3F-LN **3** being more conformationally flexible than the LN **2**, probably because of the disruption of the O5′···H−O3 hydrogen bond. The 3′F-LN **6** exhibited higher MSDs of the Φ/Ψ *g−*/*g+* conformers **A**−**D** than the LN **2**, also implying the presence of additional conformational dynamics in solution. For the 6′F-LN **8**, the best fit between the experimental and calculated shifts was deduced for the conformer **D**, with the 6′-*gt* conformation of the galactopyranoside exocyclic fluoromethyl group. This fact is in agreement with previous observations,^18^ which reported that the fluorine atom increases the gauche effect, thus stabilizing the *gt* conformer over the *tg* conformer in the *galacto*-configuration (Figure 7 and 9).

In summary, the correlation between the DFT-calculated and the experimentally determined ^13^C chemical shifts has provided a detailed insight into the conformational behavior of the LNs **2**−**8** in solution concerning the conformation of the glycosidic linkage, exocyclic hydroxyls, and acetamido group. Fittingly, the results obtained are also in agreement with the corresponding crystal structures.

### *J*-Coupling-Based Conformational Analysis

To complement the ^13^C-based methodology discussed above, the conformations of the exocyclic hydroxymethyl/fluoromethyl groups were investigated using vicinal *J*-couplings between the diastereotopic protons at the 6- and 6′-positions and the corresponding vicinal protons at the 5- and 5′-positions.^18^ The conformation of these groups can be defined as a fast dynamic equilibrium between three staggered conformers denoted as *gg*, *gt* and *tg* (Figure 7). Each of these conformers is characterized by the specific values of the limiting ^3^*J*_H5-H6pro*R*_ and ^3^*J*_H5-H6pro*S*_ coupling constants, which makes it possible to calculate the *gg*/*gt*/*tg* ratio from the corresponding experimental ^3^*J* coupling constants (Table 3). We have calculated the limiting couplings of *gg*/*gt*/*tg* conformers by DFT (Table S21) and used them for the estimation of the populations of individual conformations (*f*_*gg*_, *f*_*gt*_, *f*_*tg*_ values). The assignment of pro*R* and pro*S* protons is described in the Supporting Information

For the GlcNAc units, the LNs **2** and **4**−**8** exhibited the couplings ^3^*J*_H5-H6pro*R*_ (4.6−5.1 Hz) and ^3^*J*_H5-H6pro*S*_ (1.8−2.4 Hz), corresponding to the strong preference of 6-*gg* (63−66%) over 6-*gt* (30−35%) conformers, and a negligible population of the 6-*tg* conformer (≤5%). The 3F-LN **3** showed decreased ^3^*J*_H5-H6pro*R*_ coupling (4.0 Hz), consistent with a slightly enhanced population of the 6-*gg* conformer (71%). The negligible population of the *tg* conformer in the GlcNAc unit is explained by an unfavorable steric 1,3-interaction (syn-pentane-type) between the O6H and O4H hydroxyl groups and the absence of a stabilizing stereoelectronic gauche effect.^18^

For the Gal units, the compounds **4**−**7** exhibited comparable values of the diagnostic couplings ^3^*J*_H5′-H6′pro*R*_ (6.7−7.3 Hz) and ^3^*J*_H5′-H6′pro*S*_ (5.2−5.9 Hz), corresponding to the *gg*/*gt*/*tg* conformer ratio of ca. 15/55/30 in accordance with the previously published conformational analysis of lactose analogs.^17^ The 6′F-LN **8** showed the largest difference between the coupling constants ^3^*J*_H5′-H6′pro*R*_ (7.9 Hz) and ^3^*J*_H5′-H6′pro*S*_ (3.2 Hz), corresponding to the *gg*/*gt*/*tg* ratio of 17/76/7. The strong preference for the 6′-*gt* is a result of the enhancement of the gauche effect via fluorine electronegativity, which is in agreement with the ^13^C-based conformational analysis described above and reported data.^18^ Interestingly, the 3F-LN **3** was the only analog showing a larger ^3^*J*_H5′-H6′proS_ coupling constant (7.1 Hz) than ^3^*J*_H5′-H6′pro*R*_ (5.9 Hz), corresponding to the highest population of the 6′-*tg* conformer (50%). Such an observation is in accordance with the above-discussed X-ray analysis because 3F-LN **3** crystallized in the 6′-*tg* conformation (Figure 5B). Furthermore, the fact that the conformation around C6′H_2_OH is affected by the fluorination of the 3-position indicates an interaction between the hydroxyls at the 3- and 6′-positions, consistent with the temperature-dependent ^1^H NMR analysis discussed below.

The conformational behavior of an acetamido group in carbohydrates is usually described as a dynamic equilibrium between (*Z*)-*anti* and (*Z*)-*syn* rotamers (Figure 7), where (*Z*)-*anti* is considered the stabler geometry.^50^ However, a qualitative analysis of the *J*_H2-NH_ couplings could indicate the prevalence of the (*Z*)-*syn* conformation in solution, because the experimental *J*_H2-NH_ coupling values were 8.5−9.1 Hz (Table 3), closer to the *J*_H2-NH_ ≈ 8.3 Hz values estimated by DFT calculations for the (*Z*)-*syn* conformer, and relatively far from the limiting coupling value *J*_H2-NH_ ≈ 11.6 Hz, computed for the (*Z*)-*anti* geometry (Table S22). To explain such a discrepancy, the conformational behavior of the acetamido group was investigated by DFT in the presence of an explicit DMSO molecule (Figure S18). Interestingly, the explicit solvation significantly influenced the acetamide geometry and the calculated *J*_H2-NH_ coupling. Therefore, the implicit solvation model is not an appropriate approximation of solvation effects concerning acetamido protons, which are involved in hydrogen bonding with the solvent molecules.^61^ In fact, the analysis of NOE contacts between the NH amide proton and H1, H2 and H3 ring protons (see below) was crucial for the proper definition of the preferential conformation of the acetamide.

### Analysis of ROESY NMR Spectra

**Figure 10:**
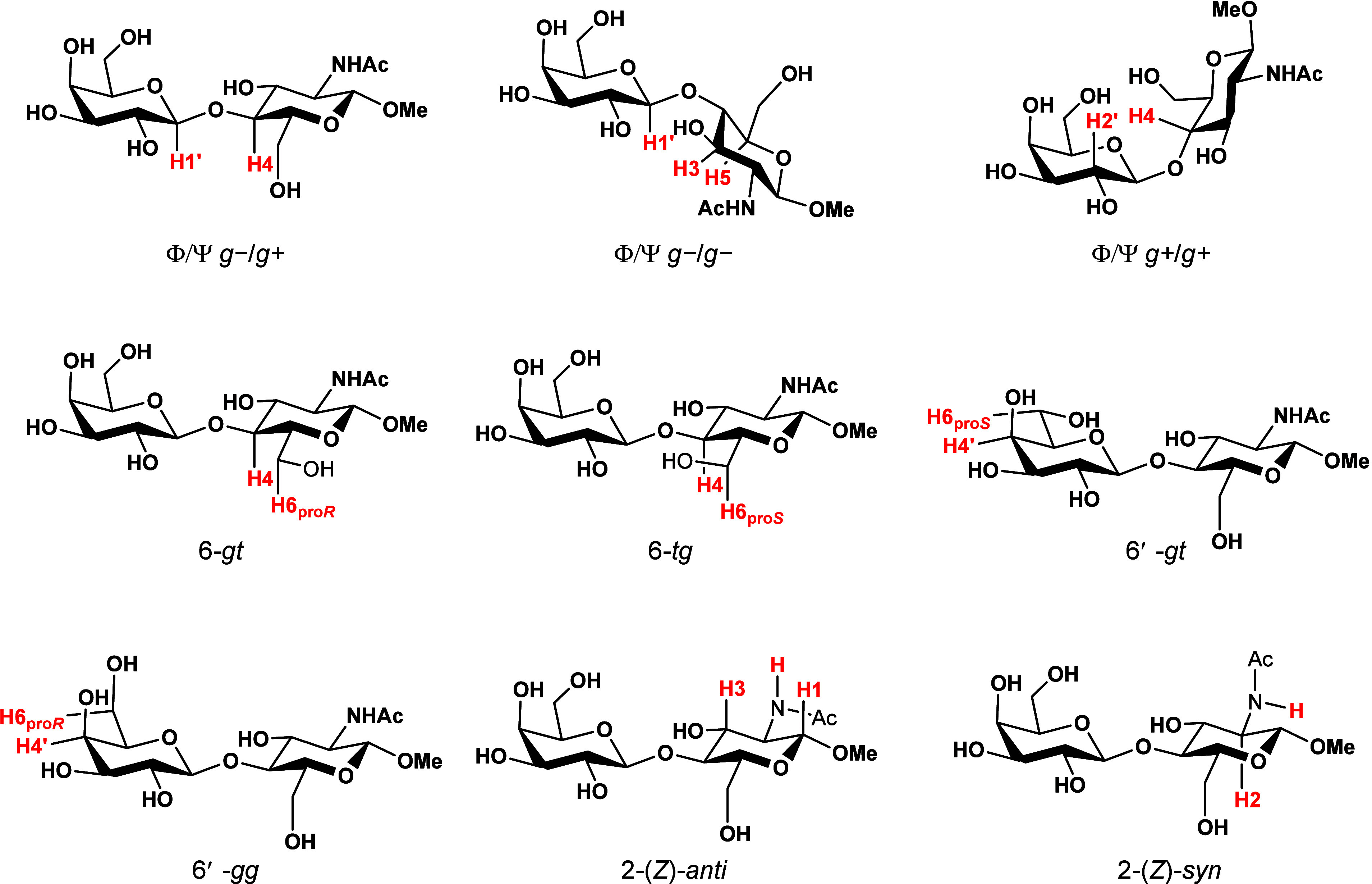
Possible conformations around the glycosidic linkage, the exocyclic hydroxymethyl groups and the acetamido group, together with the corresponding expected exclusive NOE/ROE spatial contacts for every geometry.

Complementarily to the ^13^C NMR-based and *J*-coupling-based results, the compounds **2**−**8** were studied using ^1^H-^1^H ROESY NMR to estimate the spatial proximity of protons, which can be related to the presence of specific conformations (Figure 10).

The LNs **3**−**8** exhibited strong H1′/H4 contacts, confirming the expected dominance of the Φ/Ψ *g−*/*g+* conformation (Table 4, Figure 10). The H1′-H3/H5 contacts were significantly weaker, yet detectable, indicating the presence of a minor population of the Φ/Ψ *g−*/*g−* conformation in all fluoro-analogs. The presence of the H2′-H4 contact, characteristic of the Φ/Ψ *g+*/*g+* conformation, could only be assessed for the 2′F-LN **5** and 3′F-LN **6** because of the signal overlap in the other compounds. Nevertheless, none of those compounds showed a clear H2′-H4 NOE correlation, indicating that the population of the Φ/Ψ *g+*/*g+* conformer is marginal, if present at all.

The LNs **4**−**8** showed a stronger H4/H6_pro*R*_ NOE than H4/H6_pro*S*_, indicating a preference for the 6-*gt* conformer over the 6-*tg* in the GlcNAc unit, in agreement with the DFT analysis of both ^13^C chemical shifts and *J*-couplings described above. In LN **2** and 3F-LN **3**, these contacts could not be analyzed separately because of the overlap of the protons H6_pro*R*_ and H6_pro*S*_. A similar issue with the overlapping protons H6′_pro*R*_ and H6′_pro*S*_ was found in the compounds **2** and **4**−**6**. In 3F-LN **3**, 4′F-LN **7** and 6′F-LN **8**, however the introduction of the fluorine atom caused the separation of the signals of the H6′_pro*R*_ and H6′_pro*S*_ protons, enabling the identification of a stronger NOE for H4′/H6′_pro*S*_ than for H4′/H6′_pro*R*_ (Figure S17). This confirmed the preference of the 6′-*gt* over 6′-*gg* conformation, which was also in agreement with the DFT analysis of both ^13^C chemical shifts and *J*-couplings. Furthermore, the LNs **3**−**8** showed a stronger NOE correlation between the protons (N)H and H1/H3 than between the protons (N)H and H2, indicating the expected preference for the (*Z*)-*anti* over (*Z*)-*syn* acetamide conformation. The observation of weak (N)H-(O3)H contacts indicated that the acetamide Ω3 torsion angle slightly deviated from the ideal arrangement (the antiperiplanar geometry of H2 and NH protons), in accordance with DFT analysis with explicit DMSO solvation (Figure S18).

### Analysis of Hydroxyl-Solvent Interactions by ^1^H NMR and HSQC-TOCSY

The ^1^H chemical shifts of exchangeable protons exhibit a linear dependence on temperature, which can be employed for the identification of intra- and intermolecular hydrogen bonds in oligosaccharides.^43,44^ Strongly solvated hydroxyl protons show higher values of −Δδ/Δ*T* temperature coefficients than those engaged in intramolecular hydrogen bonds.^62,63^

The nonfluorinated LN **2** shows marked differences in the temperature coefficients −Δδ/Δ*T* of individual hydroxyls as determined by variable temperature NMR in DMSO-*d*_6_ (Table 5). The lowest coefficient (2.0 ppb/K) has been observed for O3−H, which corresponds to the presence of the O5′···H−O3 hydrogen bond in agreement with X-ray analysis. On the other hand, the protons residing at the hydroxyls O2′−H and O3′−H have higher temperature coefficients (6.8 and 7.2 ppb/K respectively), which confirms their significant solvation. Such results are consistent with previous reports describing that the hydroxyl groups in vicinal equatorial diols cannot form direct intramolecular hydrogen bonds with each other, although they can form strong bidentate hydrogen bonds with solvent molecules.^64,65^ The temperature coefficients of the primary hydroxyls O6−H and O6′−H (5.9 and 4.9 ppb/K, respectively) are quite similar, although the observed values indicate a higher engagement of the hydroxyl O6′−H in intramolecular hydrogen bonding.

Our series **3**−**8** provides a unique opportunity to probe intramolecular hydrogen bonding patterns in the LN system (Table 5). First of all, the NH protons on acetamido groups show only negligible differences in −Δδ/Δ*T* coefficients, suggesting that NHAc solvation is not affected by monofluorination. The presence of fluorine generally results in a decrease in the −Δδ/Δ*T* temperature-dependence coefficients of the vicinal hydroxyls. Taking into consideration that DMSO-*d*_6_ could only act as a hydrogen-bond acceptor,^66^ deoxyfluorination possibly decreases the hydrogen-bond-donating capacity of the corresponding equatorial vicinal hydroxyl, decreasing the stability of the O−H···O=SMe_2_ hydrogen bond, in accordance with previous reports.^67−69^ When compared to the nonfluorinated LN **2**, both 2′F-LN **5** and 4′F-LN **7** show basically the same decrease of the O3′−H coefficients −Δδ/Δ*T* by 1.2 ppb/K, indicating that the fluorine configuration—axial or equatorial—does not play a role in reducing the hydrogen-bond-donating capacity of the equatorial vicinal hydroxyls.

**Figure 11:**
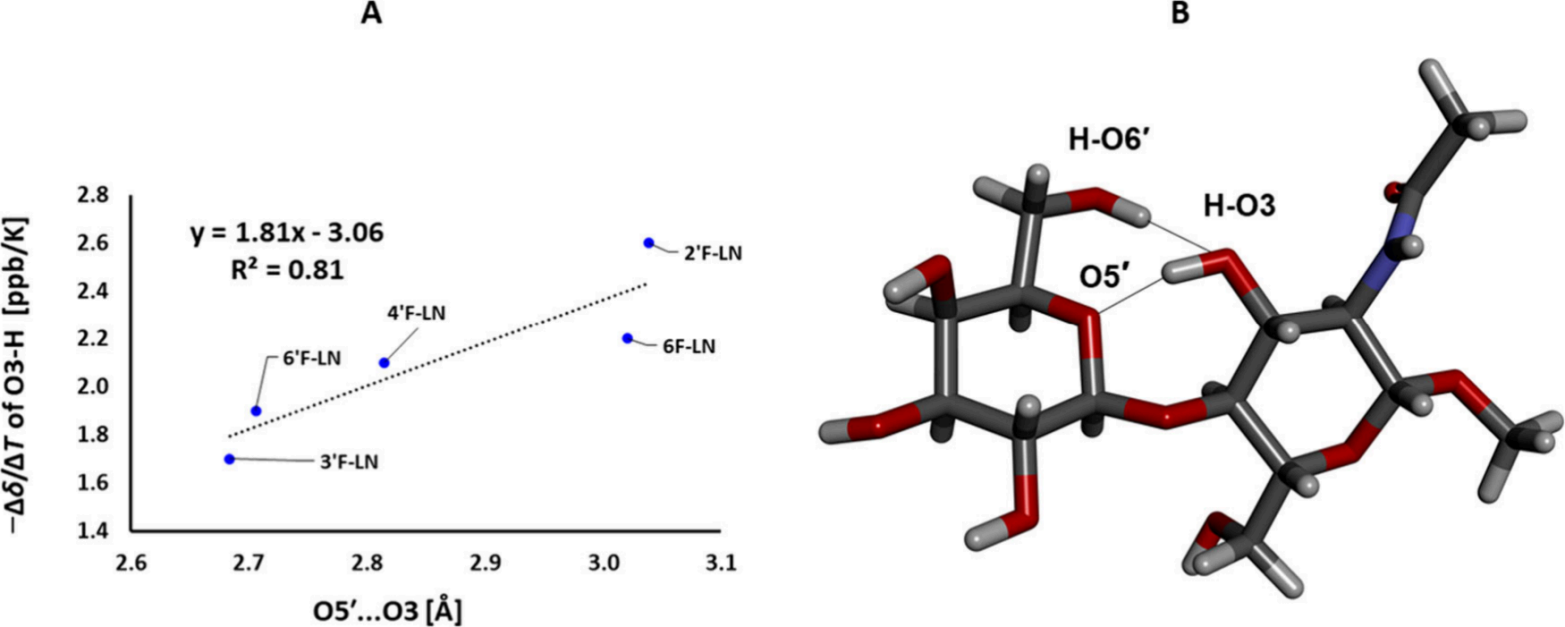
A) The correlation between the O3−H temperature coefficients −Δδ/Δ*T* and the X-ray-determined O5′···O3 distance. B) The structure of LN **2** with a depiction of O5′···H−O3 and O6′−H···O3 hydrogen bonds.

Apart from the vicinal effect, the fluorine-induced changes of −Δδ/Δ*T* coefficients also exhibited small, yet detectable, long-range effects, reflecting the subtle alteration of the LN molecular structure. Primarily, the coefficients of O3−H showed a dependence on the position of fluorine. The highest coefficient was observed for 2′F-LN **5** (2.6 ppb/K) whereas the lowest one for 3′F-LN **6** (1.7 ppb/K). The remaining fluorinated disaccharides **4**, **7** and **8** were in between these values, with their coefficients comparable to LN **2** (2.0 ppb/K). Fittingly, the −Δδ/Δ*T* coefficients of O3−H correlated with the X-ray-determined O5′···O3 distance (Figure 11, R^2^ = 0.81), strongly indicating that the trends observed in the crystal structures are also relevant for DMSO-*d*_6_ solution. Accordingly, 2′F-LN **5**, with the longest O5′···O3 distance according to the X-ray, showed the highest temperature coefficient for O3−H, suggesting a weaker inter-residual O5′···H−O3 hydrogen bond than LN **2** also in solution. Conversely, both 3′F-LN **6** and 6′F-LN **8**, with the shortest O5′···O3 distance in solid state, seem to have stronger O5′···H−O3 hydrogen bonds in solution than LN **2** according to their lower −Δδ/Δ*T*. The linear relationship between the −Δδ/Δ*T* coefficients of O3−H and the O5′···O3 distance (Figure 11A) may be employed for an estimation of the intramolecular hydrogen-bond length in other β-1,4-linked oligosaccharides, in cases where an X-ray analysis is not possible.

**Figure 12:**
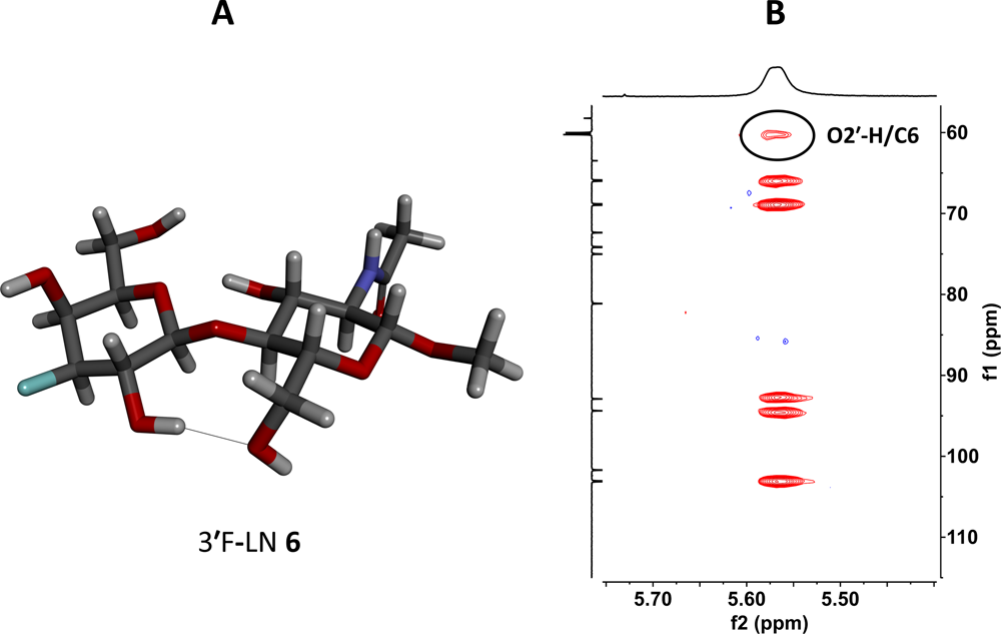
A) The DFT-optimized geometry of 3′F-LN **6** showing the O2′−H···O6 hydrogen bond. B) The HSQC-TOCSY spectrum of 3′F-LN **6**, depicting the O2′−H/C6 correlation.

Valuable information has also been extracted from the temperature coefficients of O2′−H, with the lowest −Δδ/Δ*T* coefficient (4.9 ppb/K) observed for 3′F-LN **6**, which corresponds to a decrease by 1.9 ppb/K in comparison with LN **2**. This decrease is higher than one would expect from solely the fluorine vicinal effect (decrease by 1.2 ppb/K for 3′−OH in **5** or **7**). Similarly, 6′F-LN **8** features the second lowest −Δδ/Δ*T* coefficient of O2′−H (5.5 ppb/K), which is decreased by 1.3 ppb/K relative to LN **2**, despite the separation of the proton concerned by seven bonds from fluorine. Such a decrease strongly suggests the presence of a transient O2′−H···O6 hydrogen bond in both 3′F-LN **6** and 6′F-LN **8** in DMSO-*d*_6_ solution, which can also be correlated with stronger O5′···H−O3 hydrogen bonds implying a slight reduction of glycoside linkage conformational flexibility in these two analogs. Fittingly, the presence of the O2′−H···O6 hydrogen bond in 3′F-LN **6** (Figure 12A) has been independently confirmed by the observation of HSQC-TOCSY cross correlation between O2′−H and C6 (Figure 12B). In this experiment, no correlation between O2′−H and C4 or C5 has been observed, confirming the presence of a proton/proton coupling transferred through the hydrogen bond.

The formation of a strong O6′−H···O4′ or O4′−H···O6′ hydrogen bond, which is in principle possible in *galacto*-configured carbohydrates, has been excluded because the fluorination of the 4′-position (**7**) or the 6′-position (**8**) did not cause any significant increase in the solvation of O6′-H or O4′-H, respectively. This is in accordance with a very minor population of the 6′-*gg* conformation, as determined by both ^13^C- and *J*-coupling-based NMR analyses (Table 3), which would be stabilized by these hydrogen bonds. Conversely, the DFT calculations suggested the existence of a weak O6′−H···O3 hydrogen bond (Figure 11B), which could explain the somehow lower solvation of O6′−H (−Δδ/Δ*T* = 4.9 ppb/K) in comparison with that of O6−H (−Δδ/Δ*T* = 5.9 ppb/K) in LN **2**. The presence of such a hydrogen bond is further supported by the increased O6′−H solvation in 3F-LN **3** (−Δδ/Δ*T* = 5.7, Table 5), where this hydrogen bond is disabled by 3-deoxyfluorination. Such an observation is also consistent with the prevalence of the 6′-*gt* conformer in **4**−**8** and the preference for the 6′-*tg* in 3F-LN **3** (Tables 2, 3 and 4).

### Relationships between Molecular Structure and Lipophilicity

**Figure 13:**
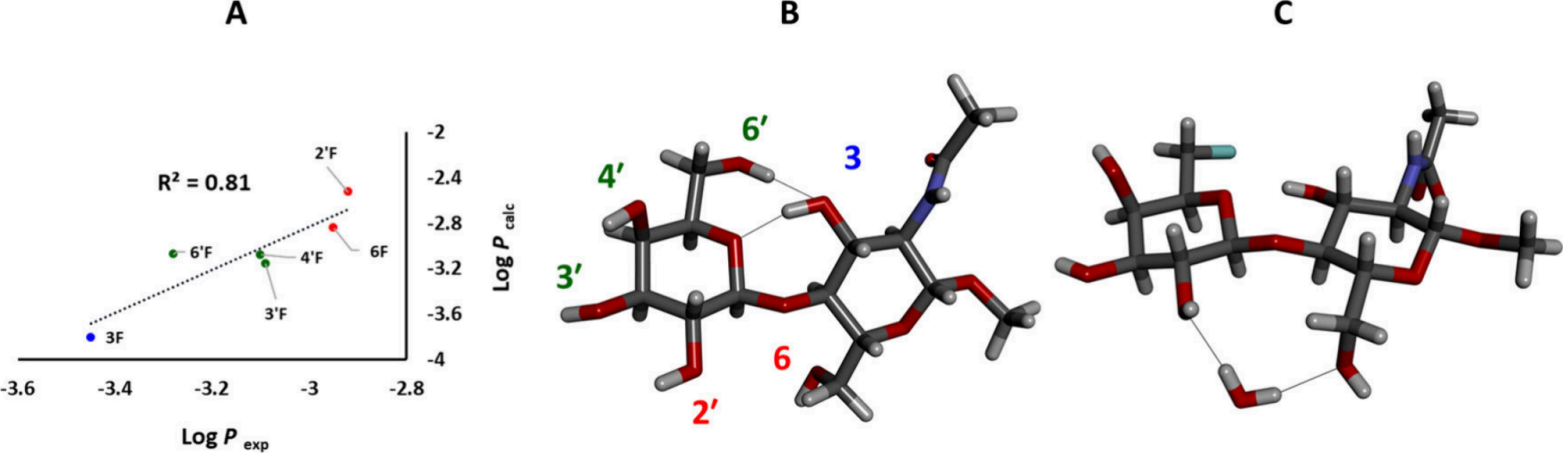
A) The correlation between the experimentally determined and the calculated values of log *P*. B) The structure of the LN **2** with the colored labeling of individual hydroxyls illustrating the lipophilicity trends. C) The crystal structure of 6′F-LN **8** with a bridging water molecule.

Lipophilicity is a key parameter for the design of new pharmacologically active compounds.^70^ The lipophilicity of the fluorinated LNs **3**−**8**, expressed as an octanol/water partition coefficient (log *P*), was previously determined by the shake-flask procedure using the measurement of ^19^F NMR resonances in the octanol and water phases (Table 6).^45^ In this work, the log *P* values of the compounds **3**−**8** have been estimated via the COSMO-RS methodology,^46^ using the stablest conformers of the LNs **3**−**8**. The calculation has furnished data in excellent agreement with the shake-flask experiment (Table 6, Figure 13A).

Both the experimental and computational COSMO-RS analysis has identified 2′F-LN **5** as the most lipophilic and 6F-LN **4** as the second most lipophilic molecule in the series **3**−**8**. Conversely, 3F-LN **3** has been identified as the least lipophilic in the series. The remaining compounds **6**, **7** and **8** have shown similar intermediate lipophilicity (Figures 13A and 13B). Although DMSO and water solvation should be different, it is tempting to analyze the lipophilicity data by means of the determined −Δδ/Δ*T* temperature coefficient. Nevertheless, this analysis should be taken with caution. The relatively low lipophilicity of 3F-LN **3** can be rationalized by the previously discussed temperature dependence of exchangeable-proton chemical shifts. Such analysis has confirmed the involvement of the hydroxyl O3−H in the inter-residual O5′···H−O3 hydrogen bond, which should decrease its hydration. Therefore, 3-deoxyfluorination to provide 3F-LN **3** does not increase lipophilicity to the same extent as deoxyfluorination in other positions. This observation also indirectly indicates that the inter-residual O5′···H−O3 hydrogen bond persists even in water solution.^71^

Conversely, the relatively high lipophilicity of the compounds 2′F-LN **5** and 6F-LN **4** is consistent with the high −Δδ/Δ*T* temperature coefficients of the protons O2′−H and O6−H in the LN **2** (6.8 and 5.9 ppb/K, respectively), indicating their strong solvation and explaining the significant increase in lipophilicity upon their deoxyfluorination. Interestingly, the relatively high increase in lipophilicity found for 2′F-LN **5** and 6F-LN **4** is also reflected in their crystal structures because only those two LN analogs have crystallized without bound water molecules (Figures S8−S13). Moreover, the crystal structure of 6′F-LN **8** contained crystal-bound water tethering the hydroxyls O2′−H and O6−H (Figure 13C). Such bidentate O2′−H/O6−H solvation is obviously weakened in both 2′F-LN **5** and 6F-LN **4**, consistent with their relatively high lipophilicity.

## Conclusions

We have herein analyzed the complete series of monofluorinated LacNAcβ1-OMe analogs to investigate the role of individual hydroxyls in the conformational behavior and lipophilicity of *N*-acetyllactosamine. These analogs have been studied by molecular and quantum mechanics, X-ray crystallography and NMR-based techniques, which have revealed that some intramolecular inter-residue hydrogen bonds (O5′···H−O3, O2′−H···O6 and O6′−H···O3) fine-tune *N*-acetyllactosamine conformational behavior. The deoxyfluorination of LacNAcβ1-OMe at the 6- and 4′-positions has no significant effect on the conformation. The deoxyfluorination of the 2′-position has resulted in the weakening of the O5′···H−O3 inter-residual hydrogen bond, likely leading to a subtle flexibilization of the glycosidic linkage. The most significant glycosidic-linkage flexibilization was observed upon the deoxyfluorination of the 3-position, which was attributed to the disruption of the O5′···H−O3 hydrogen bond. The 3-fluoro-*N*-acetyllactosamine analog also exhibited an anomaly in the conformational behavior of the exocyclic hydroxyl at position 6′, likely caused by a disruption of the O6′−H···O3 hydrogen bond. In contrast, 3′- and 6′- deoxyfluorination resulted in a subtle glycosidic-linkage conformational rigidification, probably associated with the strengthening of the O5′···H−O3 and O2′−H···O6 hydrogen bonds in solution. Such conformational effects probably influence biological activity. For example, we previously reported that 2′F-LacNAcβ1-OMe binds human galectin-1 with significantly higher affinity than nonfluorinated LacNAcβ1-OMe.^45^ As 2′F-LacNAcβ1-OMe showed the weakening of the O5′···H−O3 hydrogen bond, it is tempting to speculate that glycoside bond conformational flexibilization accounts for the affinity increase. However, as protein−ligand recognition is a complex process, further investigation would be required to confirm such speculation.

COSMO-RS *in-silico* calculations have confirmed the previously published lipophilicity of the investigated deoxyfluorinated LacNAcβ1-OMe analogs.^45^ NMR and X-ray analysis have enabled a partial rationalization of the observed lipophilicity trends, indicating that hydroxyls at 2′- and 6-positions are significantly more solvated in water solution then the hydroxyl at 3-position. Our detailed conformational analysis and lipophilicity rationalization could serve as a guideline for the synthesis of novel tailored LacNAc-derived glycomimetics.

## Experimental Section

### Molecular Mechanics

The systematic relaxed scan of glycoside torsions Φ (H1′−C1′−O4−C4) and Ψ (C1′−O4−C4−H4) was performed using the MacroModel^72,73^ software implemented in Maestro modeling environment (Maestro, Schrödinger, LLC, New York, version 13.8.135, MMshare Version 6.4.135, Release 2023−4, Platform Linux-x86_64). For the discussion, the glycosidic angles were described following the definition based on heavy atoms, Φ (O5′−C1′−O4−C4) and Ψ (C1′−O4−C4−C3) for comparison with experimental data derived from crystallographic structures. The topology of investigated systems **2**−**8** was constructed by automatic approach using MM3 force field^74^ with the use of implicit GBSA^75^ water solvation. This solvation model was selected to allow comparison with the literature data.^48^ Each scan generated a grid of torsions Φ and Ψ in an interval −300° to 60° with a step of 10°, therefore 1369 structures. Four combinations of exocyclic chain conformations 6/6′: *gt*/*gg*, *tg*/*gg*, *gt*/*gt*, *tg*/*gt* were considered, generating the 5476 structures in total for each investigated system **2**−**8**. Every generated structure was minimized using the default PRCG (Polak-Ribiere conjugate Gradient) minimization^76^ with maximally 2500 iterations and 0.05 convergence threshold. The final adiabatic Φ/Ψ energy diagrams were built using the lowest energy structure for each obtained Φ/Ψ point. The potential energy maps were transformed into the Φ/Ψ probability distribution by Boltzmann distribution. To compare individual compounds **2**−**8**, the obtained data were statistically analyzed in Microsoft excel Professional Plus 2019 and plotted through GNU Octave 8.3.0.^77^

### X-ray Crystallography

Crystals of compounds **3**−**8**, suitable for X-ray analysis, were prepared by the vapor diffusion technique^78^ using the demineralized water as the solvent and *tert*-butanol as the antisolvent. The starting concentration of the compound was approximately 20 mM. Demineralized water with conductivity ≤0.1 μS was obtained using ROTFM-5SV reverse osmosis. All crystals showed melting points of approximately 350 °C accompanied by decomposition, preventing their precise determination. The diffraction data was collected on a Bruker D8 VENTURE Kappa Duo PHOTON 100 CMOS with monochromated Mo/Cu−Kα radiation. Structures were solved by direct methods (SHELXT^79^) and refined by full-matrix least-squares on F^2^ values (SHELXL^79^ or CRYSTALS^80^). All heavy atoms were refined anisotropically. Hydrogen atoms were localized from the expected geometry and difference electron density maps and usually were refined isotropically. ORTEP-3^81^ was used for the presentation of the structure. The crystallographic data for the structures reported in this paper have been deposited in the Cambridge Crystallographic Data Center as a supplementary publication. Copies of the data can be obtained free of charge on application to CCDC, e-mail: deposit@ccdc.cam.ac.uk. BIOVIA Discovery Studio Visualizer v21.1.0.20298 was used to generate the associated graphics.

### NMR Analysis

NMR spectra for the characterization of compounds **2**−**8** in DMSO-*d*_6_ were acquired using Bruker Avance 400 (^1^H at 400.1 MHz, ^19^F at 376.4 MHz, ^13^C at 100.6 MHz) or Bruker Avance 500 (^1^H at 500.1 MHz, ^19^F at 470.5 MHz, ^13^C at 125.8 MHz) at 25 °C. The ^1^H and ^13^C NMR spectra were referenced to the DMSO-*d*_6_, 2.50/39.52 ppm. The ^19^F NMR spectra were referenced to the line of the external standard hexafluorobenzene (δ/ppm −163.86 in DMSO-*d*_6_). Structural assignments were made with additional information from gCOSY, gHSQC, gHMBC and H,C-gHSQC-TOSCY). Diagnostic *J*_H6′pro*R*-H5_ and *J*_H6′pro*S*-H5_ couplings were extracted by 1D selective proton gradient ROESY, 1D selective gradient TOCSY or selective homonuclear decoupling using Bruker Avance 400 MHz, Bruker Avance III HD 600 MHz or Bruker Avance III HD 850 MHz. Bruker Avance III HD NMR spectrometers (600 and 850 MHz) were equipped with the inverse triple resonance cryo-probe with ATM module (5 mm CPTCI ^1^H/^13^C/^15^N/D Z-GRD). The analysis of the spatial proton contacts and the exchange contacts with residual water in DMSO-*d*_6_ was performed on Bruker Avance 400 via H,H-ROESY (roesyphpp.2) at 25 °C with 2 s of relaxation delay D1 and 200 ms pulse for ROESY spinlock (P15). The ^1^H temperature dependence experiments of exchangeable protons were performed on Bruker Avance 400 in the temperature range of 25−50 °C using a common zg30 pulse sequence for proton acquisition. All experiments were processed using Topspin 4.1.3 or MestreNova 12.0.3 software.

### DFT Calculations

DFT calculations were performed in Gaussian16-B.01^82^ at the B3LYP-D3/6-311++g(d,p)^83−86^ level of theory with diffuse and polarization functions on all atoms and D3 empirical dispersion correction according to Grimme.^87,88^ All calculations were performed with implicit DMSO solvation utilizing a polarizable conductor calculation model (C-PCM).^89,90^ All additional calculation variables such as convergence criteria were set to default. In all optimized structures, we calculated vibration frequencies to evaluate the character of the stationary points (confirmation of the minimum). The optimized geometries were used for the calculation of ^13^C NMR shifts and *J*-couplings using the Gauge Independent Atomic Orbital (GIAO) method^91−95^ including the Fermi contact contribution^96^ to the nuclear spin−spin coupling. The isotropic parts of the ^13^C magnetic shielding tensors (σ_*ISO*_) were converted into chemical shifts (δ_*theor*_) using the linear regression method.^97,98^ The method is based on the linear dependence between experimentally measured chemical shifts (δ_*exp*_) and theoretically obtained σ_*ISO*_. First, the linear regression coefficients (the slope and the intercept) between σ_*ISO*_ and δ_*exp*_ have been obtained using the slope and intercept functions in Microsoft Excel Professional Plus 2019. The values of δ_*exp*_ were used as x_array and σ_*ISO*_ as y_array. The slope and intercept values were later used for the calculation of δ_*theor*_ according to the equation. 1.
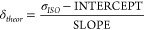
1

Subsequently, the mean square deviations (MSD) between the experimental ^13^C chemical shifts δ_exp_ and the theoretically obtained ^13^C chemical shifts δ_*th*eor_ conformer were calculated. BIOVIA Discovery Studio Visualizer v21.1.0.20298 was used to generate the associated graphics.

### COSMO-RS Calculations

Selected DFT-minimized geometries representing the most stable conformers of compounds **3**−**8** (See ESI II) were utilized for the calculation of Log *P* using the conductor-like screening model for real solvents (COSMO-RS) method,^46^ which is a semiempirical approach that shows high accuracy for partition prediction.^47^ Each conformer selected by the previous approach was used for the subsequent DFT/B−P/TZVP vacuum^99^ and COSMO water optimization with fine grid option using Turbomole 6.3.^100^ The .cosmo files for each conformer were obtained from this procedure and subsequently used for the partitioning calculation. Using the COSMOtherm X18 software and .cosmo files of all calculated conformers, the chemical potential of the molecules in water and water-saturated octanol (27.4 molar % of water in octanol) was calculated, furnishing the partition coefficients.

**Table 1:** MM3 Molecular-Mechanics Conformational Analysis of the LacNAc Analogs **2**−**8**

		Distributions [%]^b^		
Compound	D_90_^a^	Φ/Ψ*g−*/*g+*^c^	Φ/Ψ*g−*/*g−*d	Φ/Ψ*g+*/*g+*^e^
LN **2**	2.56%	98.99	0.07	0.22
3F-LN **3**	4.38%	92.45	1.16	1.23
6F-LN **4**	2.48%	98.84	0.09	0.32
2′F-LN **5**	2.41%	97.31	0.15	0.34
3′F-LN **6**	2.19%	88.87	0.56	9.38
4′F-LN **7**	2.70%	98.52	0.18	0.15
6′F-LN **8**	2.05%	97.15	0.19	0.62

a D_90_ = relative Φ/Ψ conformational space corresponding to the 90% Boltzmann distribution of conformers.

b The distribution of defined Φ/Ψ: *g−*/*g+, g−*/*g−*and *g+*/*g+* conformations.

c Φ/Ψ = (−110°− −30°)/(60°−160°).

d Φ/Ψ = (−110°− −30°)/(−90°− −30°).

e Φ/Ψ = (30°−90°)/(60°−160°).

**Table 2:**
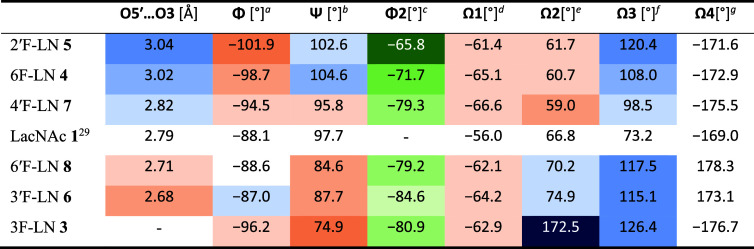
Comparison of O5′···O3 Distances and Torsions Φ, Ψ, Φ2, and Ω1−Ω4 in the Series of **3**−**8** with LacNAc **1**^h^

a [O5′−C1′−O4−C4].

b [C1′−O4−C4−C3].

c [O5−C1−O−C].

d [O5−C5−C6−O6/F6].

e [O5′−C5′−C6′−O6′/′F6′].

f [C1−C2−N−C].

g [C2−N−C−C].

h The color code indicates the relative value of a given descriptor: blue = the value is higher in comparison with α-d-LacNAc **1**,^29^ orange = the value is lower in comparison with α-d-LacNAc **1**,^29^ green = a depiction of the trend in Φ2 cannot be compared to α-d-LacNAc **1**(29) (α-configured hydroxyl group at C1).

**Table 3:** Experimental ^3^*J*_H5−H6pro*R*/H6pro*S*_, ^3^*J*_H5′-H6′pro*R*/H6′pro*S*_, and ^3^*J*_H2-NH_ Couplings [Hz] and the Calculated Distribution *f* [%] of Side Chain Conformers *gg*/*gt*/*tg*^18^

	GlcNAc					Gal					NHAc
Compound	^3^*J*_H5-__H6pro*R*_	^3^*J*_H5-__H6pro*S*_	*f*_*gg*_	*f*_*gt*_	*f*_*tg*_	^3^*J*_H5′_-_H6′pro*R*_	^3^*J*_H5′-__H6′pro*S*_	*f*_*gg*_	*f*_*gt*_	*f*_*tg*_	^3^*J*_H2-NH_
LN **2**	4.7^a^	2.2^a^	65	30	5	-b	-b	-b	-b	-b	8.6
3F-LN **3**	4.0^c^	2.4^c^	71	29	0	5.9^d^	7.1^d^	21	29	50	9.1
6F-LN **4**	4.6	1.8	64	35	1	6.7^e^	5.2^e^	23	54	23	8.5
2′F-LN **5**	5.1	1.9	65	35	0	6.8^e^	5.4^e^	20	53	27	8.6
3′F-LN **6**	5.0^a^	2.3^a^	63	34	4	7.3^f^	5.7^f^	11	56	33	8.6
4′F-LN **7**	4.8^a^	2.4^a^	65	30	5	7.3	5.9	12	51	37	8.6
6′F-LN **8**	4.8	2.2	66	32	1	7.9^g^	3.2^g^	17	76	7	8.6

a Obtained using 1D selective homonuclear decoupling of O6-H.

b The couplings could not be determined due to a spectral overlap.

c Obtained using 1D selective NH−H5 TOCSY transfer.

d Obtained using 1D selective homonuclear decoupling of O6′-H.

e Estimated from deconvolution of H5′ using 1D selective H1′−H5′ ROESY transfer.

f Estimated from deconvolution of H5′ using 1D selective O4′H−H5′ TOCSY transfer.

g Confirmed by ^1^H measurement at 31 °C.

**Table 4:** Diagnostic ROESY Contacts^a^

contact	H1′-H4	H1′-H3/H5	H2′-H4	H4-H6_pro*R*_	H4-H6_pro*S*_	H4-H6′_pro*R*_	H4-H6′_pro*S*_	NH-H1/H3	NH-H2	NH-O3H
conformation	Φ/Ψ*g*−/*g*+	Φ/Ψ *g*−/*g*−	Φ/Ψ *g*+/*g*+	6-*gt*	6-*tg*	6′-*gg*	6′-*gt*	(*Z*)-*anti*	(*Z*)-*syn*	(*Z*)-*anti*^c^
LN **2**	-b	-b	-b	-b	-b	-b	-b	S	W	W
3F-LN **3**	S	W	-b	-b	-b	VW	W	S	W	-d
6F-LN **4**	S	W	-	W	No	-b	-b	S	W	W
2′F-LN **5**	S	W	No	W	No	-b	-b	S	W	W
3′F-LN **6**	S	W	No	W	VW	-b	-b	S	W	W
4′F-LN **7**	S	W	-b	W	VW	VW	S	S	W	W
6′F-LN **8**	S	W	-b	M	W	No	S	S	W	W

a Contact strength: S = strong, M = medium, W = weak, VW = very weak, No = contact not observed, - = analysis could not be performed.

b The detection of the contact was not possible due to the signal overlap.

c The contact indicates that the acetamide (*Z*)-*anti* geometry is slightly deviated from the ideal antiperiplanar arrangement.

d O3H is substituted by fluorine.

**Table 5:** Temperature Coefficients −Δδ/Δ*T* ppb/K of Exchangeable Protons in **2**−**8**^a^

Compound	NHAc	OH-C3	OH-C2′	OH-C3′	OH-C4′	OH-C6′	OH-C6
LN **2**	4.2	2.0	6.8	7.2	-b	4.9	5.9
3F-LN **3**	4.0	-	6.0	6.8	5.8	5.7	5.3
6F-LN **4**	4.2	2.2	6.2	7.5	5.9	4.7	-
2′F-LN **5**	4.2	2.6	-	6.0	6.1	4.4	5.9
3′F-LN **6**	4.1	1.7	4.9	-	5.2	4.9	5.6
4′F-LN **7**	4.1	2.1	6.0	6.0	-	4.4	5.5
6′F-LN **8**	4.1	1.9	5.5	7.2	5.7	-	5.4

a The temperature coefficients were determined in DMSO-*d*_6_, ppb = ppm·10^−3^.

b The analysis could not be performed due to the signal overlap.

**Table 6:** Lipophilicities of the LN Analogs **3**−**8**^a^

Compound	3F-LN 3	6F-LN 4	2′F-LN 5	3′F-LN 6	4′F-LN 7	6′F-LN 8
log *P*_exp._	−3.45	−2.95	−2.92	−3.09	−3.10	−3.28
log *P*_calc_	−3.62	−2.84	−2.53	−3.16	−3.08	−3.07

a The lipophilicity of **3**−**8** has been calculated from DFT-optimized global-minimum geometries using the COSMO-RS methodology.

## Data Availability

The data underlying this study are available in the published article and its Supporting Information

## Supporting Information Available

The Supporting Information I contains outputs of molecular-mechanics calculations (energy and population diagrams); X-ray data of compounds **3**−**8**; NMR assignment of compounds **3**−**8** in DMSO-*d*_6_; and DFT-calculated NMR parameters of compounds **3**−**8** (PDF).
The Supporting Information II contains copies of ^1^H, ^13^C, ^19^F, ^1^H−^1^H gCOSY, ^1^H−^13^C gHSQC, ^1^H−^13^C gHMBC, ^1^H−^13^C gHSQC-TOCSY, ^1^H−^1^H gROESY and selective 1D proton NMR-spectra for all investigated compounds, cartesian coordinates, computed total energy values, computed sum of electronic and thermal free energies, and number of imaginary frequencies of all optimized geometries (DOCX).
