# Supplementary material for: Influence of Selective Deoxyfluorination on the Molecular Structure of Type-2 N-Acetyllactosamine
Source: J Org Chem. 2024 Aug 23;89(17):11875–90. doi: 10.1021/acs.joc.4c00879 (PMC11382267; doi:10.1021/acs.joc.4c00879)
Supplement: Supplementary file 1 — jo4c00879_si_001.pdf [file jo4c00879_si_001.pdf]

Supporting Information

The Influence of Selective Deoxyfluorination on the Molecular Structure of Type-2 *N*-Acetyllactosamine

Martin Kurfiřt,[a,e*] Lucie Červenková Šťastná,[a] Martin Dračínský,[b] Radek Pohl,[b] Ivana Císařová,[c] Jan Sýkora,[f] Martin Balouch,[g] Michal Baka,[d,h] Vojtěch Hamala,[a,e] F. Javier Cañada,[i,m] Ana Ardá,[j,k] Jesús Jiménez-Barbero,[j,k,l,m] Jindřich Karban[a]

[a] Institute of Chemical Process Fundamentals, Czech Academy of Sciences, Rozvojová 1/135, CZ-165 00 Praha 6, Czech Republic.
[b] Institute of Organic Chemistry and Biochemistry, Czech Academy of Sciences, Flemingovo náměstí 542/2, CZ-160 00 Praha 6, Czech Republic
[c] Department of Inorganic Chemistry, Faculty of Science, Charles University in Prague, Hlavova 8, CZ-128 43 Praha 2, Czech Republic
[d] Institute of Entomology, Biology Centre of the Czech Academy of Sciences, Branišovská 31, 370 05 České Budějovice, Czech Republic
[e] Department of Organic Chemistry. [f] Department of Analytical Chemistry. [g] Department of Chemical Engineering, [h] Department of Food Analysis and Nutrition, University of Chemistry and Technology, Prague, Technická 5, 166 28 Prague 6, Czech Republic
[i] Centro de Investigaciones Biológicas Margarita Salas, Ramiro de Maeztu 9, 28040 Madrid, Spain
[j] CICbioGUNE, Basque Research & Technology Alliance (BRTA), Bizkaia Technology Park, Building 800, 48162 Derio Bizkaia, Spain
[k] Ikerbasque, Basque Foundation for Science, Plaza Euskadi 2, 48013 Bilbao Bizkaia, Spain
[l] Department of Organic and Inorganic Chemistry, Faculty of Science and Technology, University of the Basque Country, EHU-UPV, 48940 Leioa, Spain
[m] CIBER de Enfermedades Respiratorias (CIBERES), Avda Monforte de Lemos 3-5, 28029 Madrid, Spain

*Corresponding author. E-mail address: kurfirt@icpf.cas.cz

Table of Contents

Table of Contents S2
Molecular Mechanics Calculation S3
X-ray crystallography S5
NMR analysis S9
Temperature Dependent ^{1}H NMR data S12
DFT calculations S15
Optimization of Molecular Geometries S15
Calculation of ^{13}C chemical shifts S16
Analysis of the exocyclic groups conformations using *J*-couplings S24
Assignment of $H6_{proR}$ and $H6_{proS}$ protons (GlcNAc rings) S24
Assignment of $H6'_{proR}$ and $H6'_{proS}$ protons (Gal rings) S25
Analysis of the acetamido group conformations using *J*-couplings S29
References S31

Molecular Mechanics Calculation

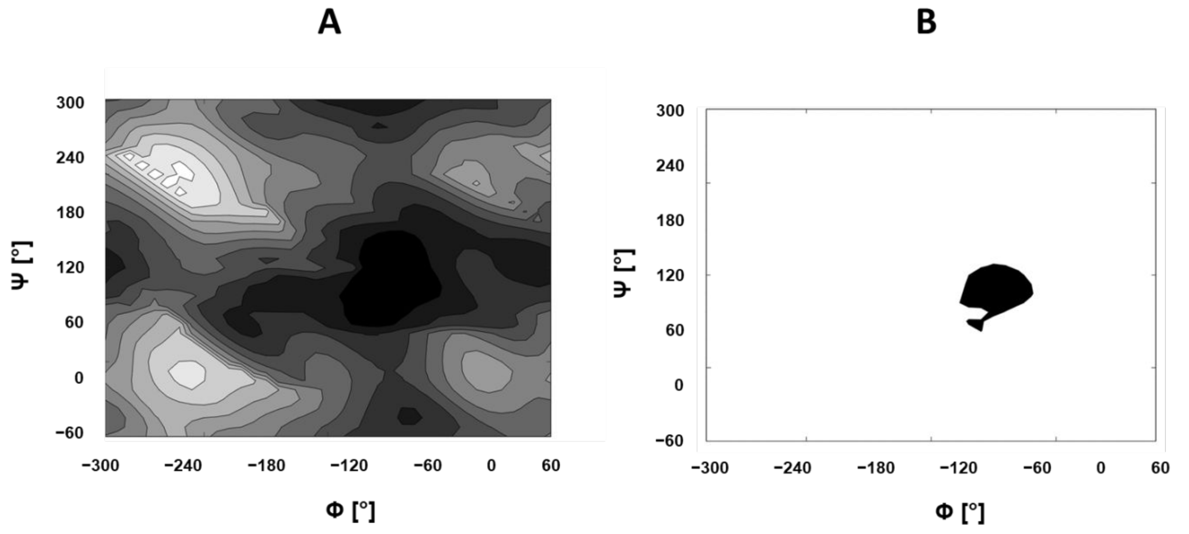

Figure S1. A) The adiabatic Φ/Ψ energy diagram of LN **2**, contour interval = 3 kcal/mol. B) The adiabatic Φ/Ψ population diagram of LN **2** with a cutoff of 90%.

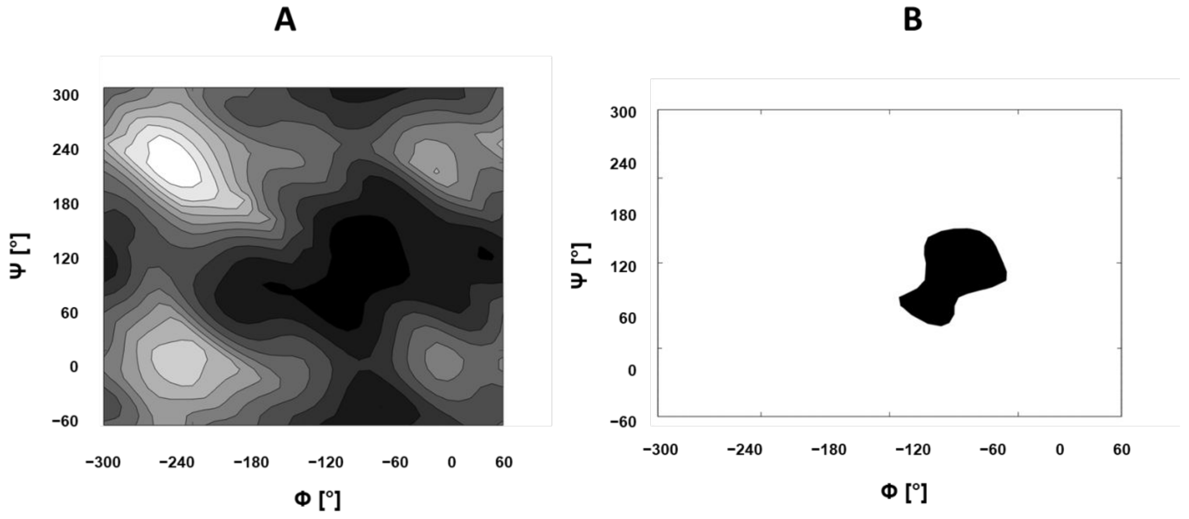

Figure S2. A) The adiabatic Φ/Ψ energy diagram of 3F-LN **3**, contour interval = 2.6 kcal/mol. B) The adiabatic Φ/Ψ population diagram of 3F-LN **3** with a cutoff of 90%.

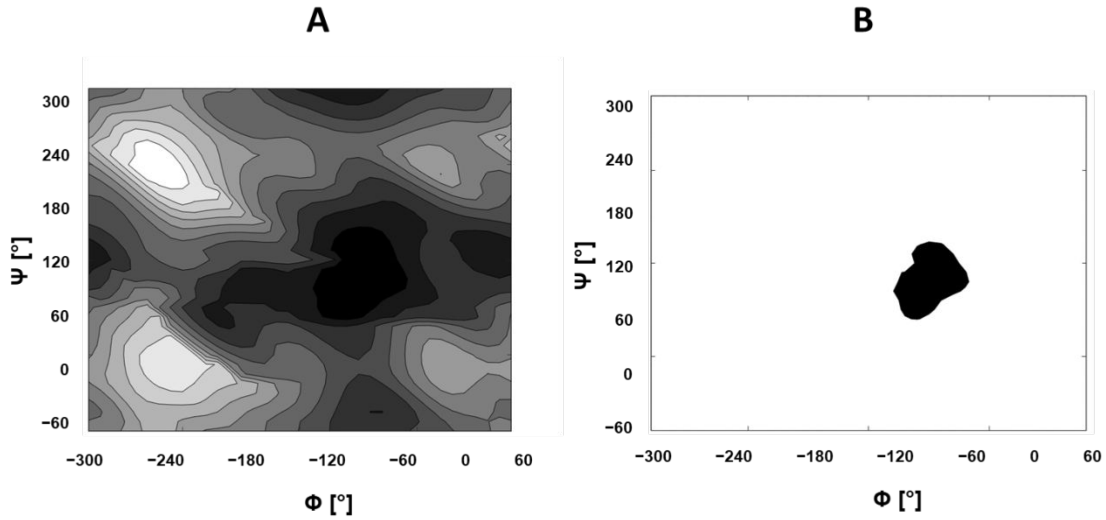

Figure S3. A) The adiabatic Φ/Ψ energy diagram of 6F-LN **4**, contour interval = 2.7 kcal/mol. B) The adiabatic Φ/Ψ population diagram of 6F-LN **4** with a cutoff of 90%.

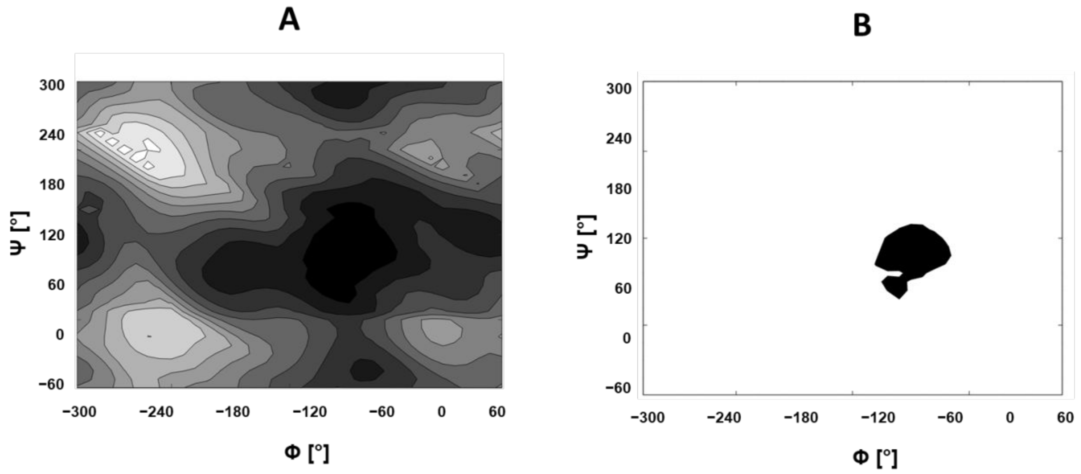

Figure S4. A) The adiabatic Φ/Ψ energy diagram of 2′F-LN **5**, contour interval = 2.9 kcal/mol. B) The adiabatic Φ/Ψ population diagram of 2′F-LN **5** with a cutoff of 90%.

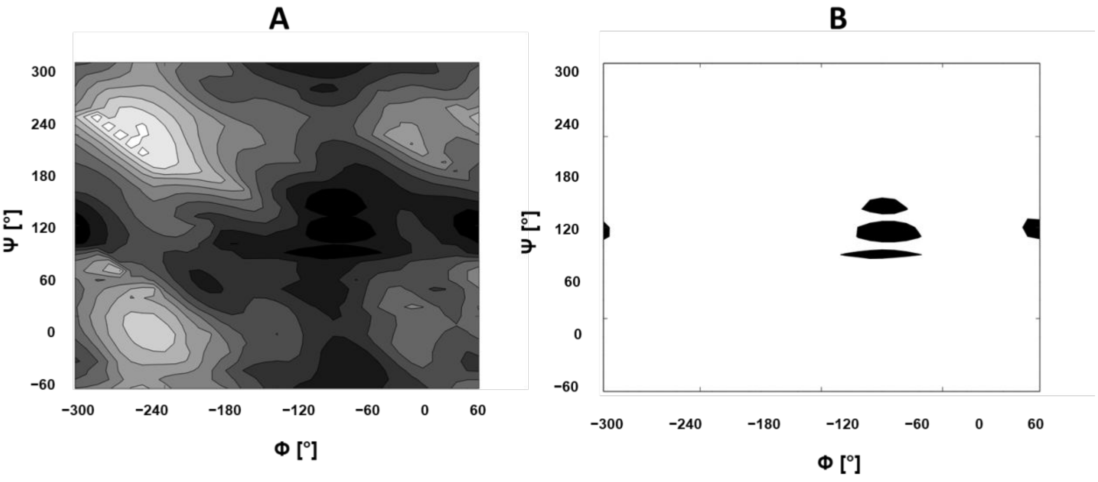

Figure S5. A) The adiabatic Φ/Ψ energy diagram of 3′F-LN **6**, contour interval = 2.9 kcal/mol. B) The adiabatic Φ/Ψ population diagram of 3′F-LN **6** with a cutoff of 90%.

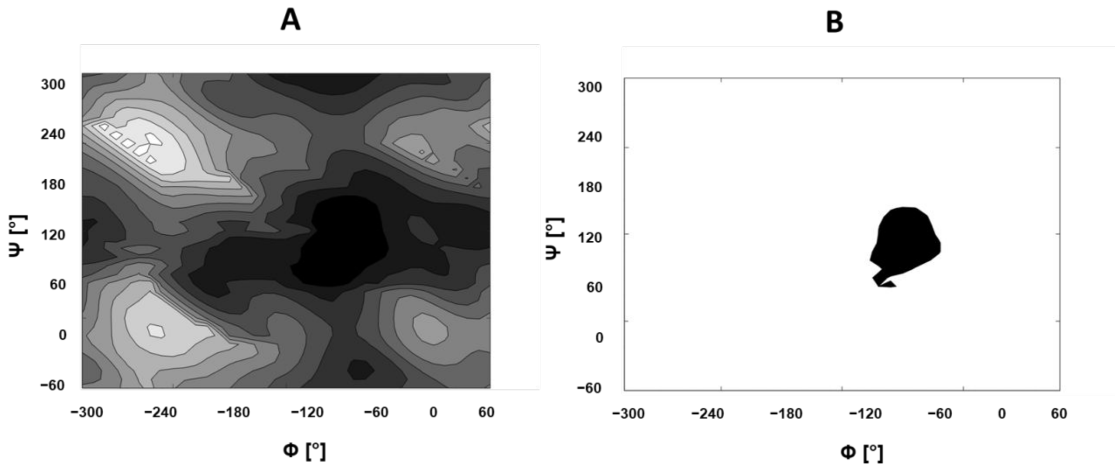

Figure S6. A) The adiabatic Φ/Ψ energy diagram of 4′F-LN **7**, contour interval = 2.9 kcal/mol. B) The adiabatic Φ/Ψ population diagram of 4′F-LN **7** with a cutoff of 90%.

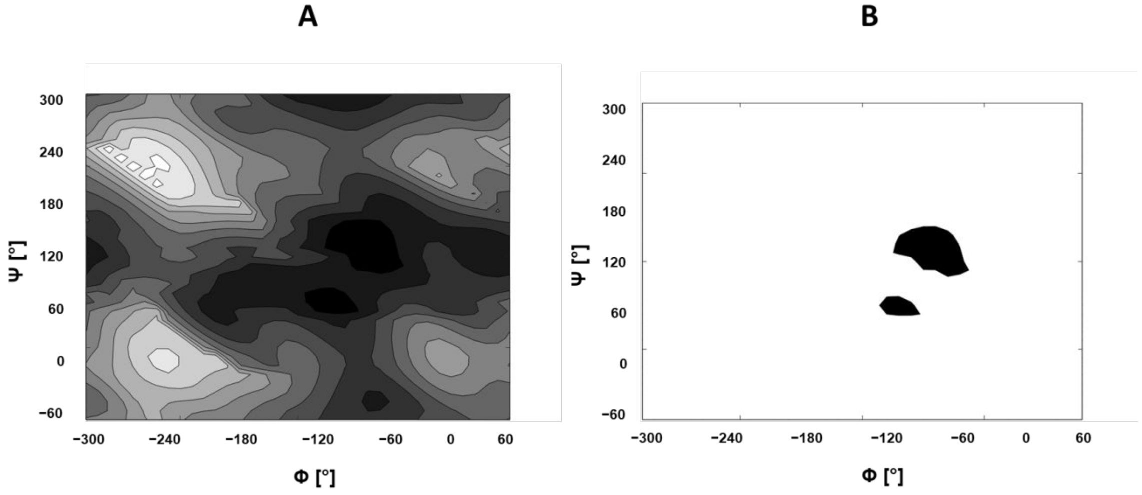

Figure S7. A) The adiabatic Φ/Ψ energy diagram of 6′F-LN **8**, contour interval = 2.9 kcal/mol. B) The adiabatic Φ/Ψ population diagram of 6′F-LN **8** with a cutoff of 90%.

X-ray crystallography

X-ray data of compound 3F-LN **3**: $C_{15}H_{26}F_1N_1O_{10} \cdot H_2O$, M= 417.39 g/mol, monoclinic system, space group *P2₁*, a=10.8089(5), b=4.7787(2), c=18.4334(9) Å, β=90.467(2)°, Z=2, V=952.10(8) Å^3, Dc=1.46 g.cm^{-3}, μ(Cu Kα)=1.131 mm^{-1}, T=120 K, crystal dimensions of 0.05 x 0.07 x 0.32 mm. The structure converged to the final R=0.0355 and R_w=0.0939 using 366

parameters for 3628 independent reflections (θ_{max}=72.04°). CCDC registration number 2327978.

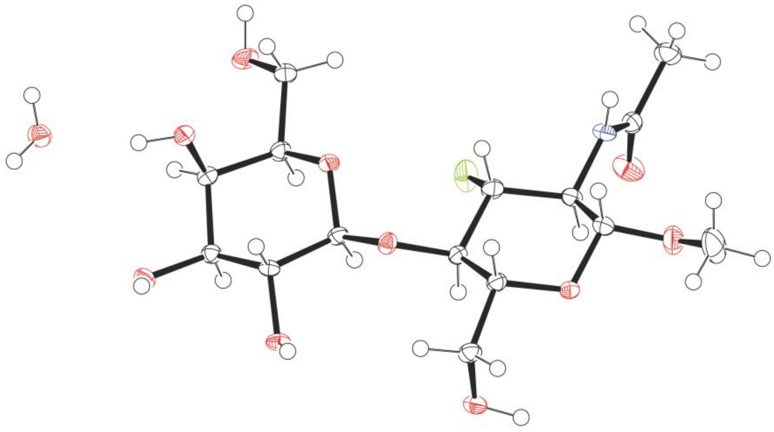

Figure S8. ORTEP projection of compound 3F-LN **3**, ellipsoid probability 50%.

X-ray data of compound 6F-LN **4**: $C_{15}H_{26}F_1N_1O_{10}$, M= 399.37 g/mol, monoclinic system, space group *P2₁*, a=4.6997(2), b=13.8563(4), c=13.2557(5) Å, β =92.605(2)°, Z=2, V=862.33(6) Å^3, Dc=1.54 g.cm^{-3}, μ(Cu Kα)=1.177 mm^{-1}, T=120 K, crystal dimensions of 0.02 x 0.04 x 0.24 mm. The structure converged to the final R=0.0451 and R_w=0.1056 using 323 parameters for 2998 independent reflections (θ_{max}=72.12°). CCDC registration number 2327977.

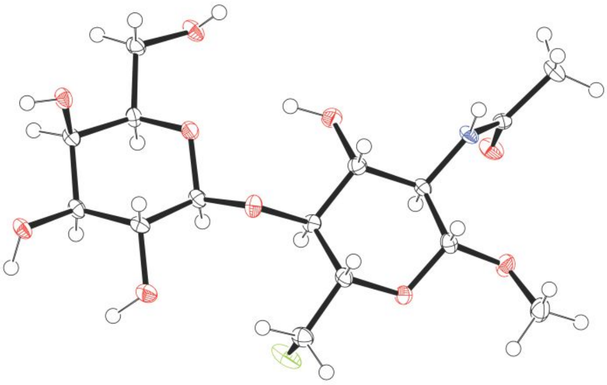

Figure S9. ORTEP projection of compound 6F-LN **4**, ellipsoid probability 50 %.

X-ray data of compound 2′F-LN **5**: $C_{15}H_{26}F_1N_1O_{10}$, M= 399.37 g/mol, monoclinic system, space group *P2₁*, a=4.7219(5), b=14.0984(15), c=13.1562(14) Å, β=93.889(3)°, Z=2, V=873.81(16) Å^3, Dc=1.52 g.cm^{-3}, μ(Cu Kα)=1.162 mm^{-1}, T=120 K, crystal dimensions of 0.09 x 0.16 x 0.46 mm. The structure converged to the final R=0.0359 and R_w=0.0944 using

349 parameters for 3378 independent reflections (θ_{max}=74.66°). CCDC registration number 2327980.

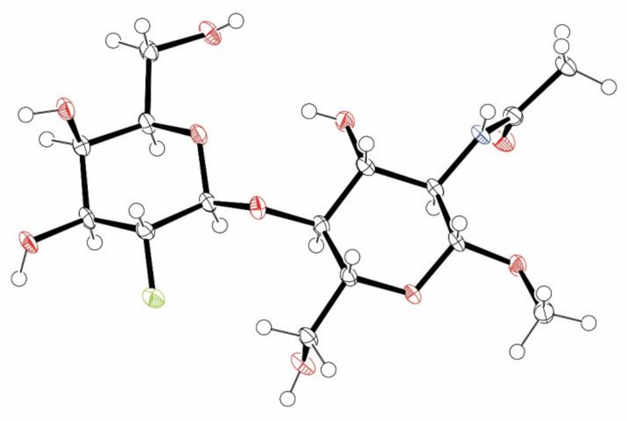

Figure S10. ORTEP projection of compound 2′F-LN **5**, ellipsoid probability 50 %.

X-ray data of compound 3′F-LN **6**: $C_{15}H_{26}F_1N_1O_{10} \cdot H_2O$, M= 417.39 g/mol, monoclinic system, space group *C2*, a=15.6474(8), b=4.7659(2), c=25.0579(13) Å, β=97.845(3)°, Z=4, V=1851.18(16) Å^3, Dc=1.50 g.cm^{-3}, μ(Cu Kα)=1.163 mm^{-1}, T=120 K, crystal dimensions of 0.08 x 0.09 x 0.38 mm. The structure converged to the final R=0.0827 and R_w=0.2128 using 255 parameters for 3517 independent reflections (θ_{max}=72.23°). CCDC registration number 2327975.

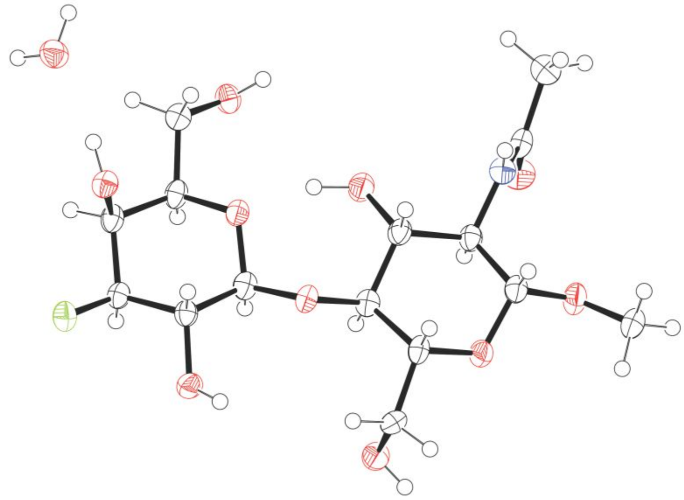

Figure S11. ORTEP projection of compound 3′F-LN **6**, ellipsoid probability 50 %.

X-ray data of compound 4′F-LN **7**: $C_{15}H_{26}F_1N_1O_{10} \cdot H_2O$, M= 417.39 g/mol, triclinic system, space group *P1*, a=4.6767(4), b=7.9281(7), c=13.5686(12) Å, α=74.144(3), β=81.721(3)°

γ=75.308(3)°, Z=1, V=466.61(7) $Å^3$, Dc=1.49 $g.cm^{-3}$, μ(Cu Kα)=1.153 mm^{-1}, T=120 K, crystal dimensions of 0.10 x 0.23 x 0.37 mm. The structure converged to the final R=0.0327 and R_w=0.0920 using 366 parameters for 3423 independent reflections (θ_{max}=74.65°). CCDC registration number 2327976.

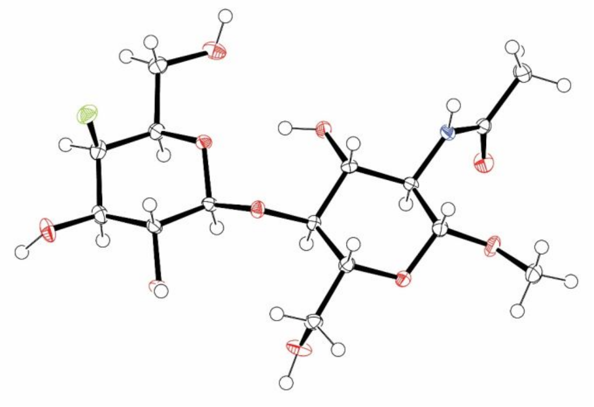

Figure S12. ORTEP projection of compound 4′F-LN **7** ellipsoid probability 50 %.

X-ray data of compound 6′F-LN **8**: $C_{15}H_{26}F_1N_1O_{10} \cdot 2.25\ H_2O$ M= 439.90 g/mol, orthorhombic system, space group *P2₁2₁2₁*, a=4.7145(2), b=9.6101(4), c=44.9300(16) Å, Z=4, V=2035.64(14) $Å^3$, Dc=1.44 $g.cm^{-3}$, μ(Cu Kα)=1.13 mm^{-1}, T=200 K, crystal dimensions of 0.05 x 0.07 x 0.79 mm. The structure converged to the final R=0.0286 and R_w=0.0807 using 359 parameters for 3932 independent reflections (θ_{max}=72.13°). CCDC registration number 2327979.

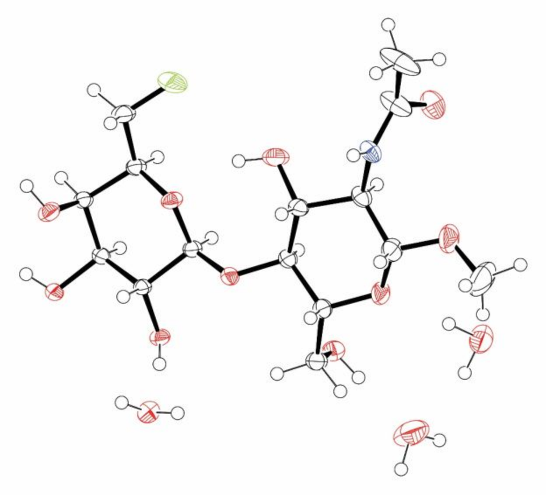

Figure S13. ORTEP projection of compound 6′F-LN **8**, ellipsoid probability 50 %.

NMR analysis

Table S1A. LacNAc ^{1}H chemical shifts δ [ppm] of unexchangeable protons in DMSO-d_6

	**1**	**2**	**3**	**4**	**5**	**6_{proS}**	**6_{proR}**	**1′**	**2′**	**3′**	**4′**	**5'**	**$6'_{proS}$**	**$6'_{proR}$**	**OMe**	**CH_3CO**
LN **2**	4.21	3.48	3.46	3.29	3.26	3.76	3.62	4.20	3.30	3.30	3.61	3.44	3.49	3.46	3.32	1.79
3F-LN **3**	4.27	3.70	4.40	3.70	3.26	3.77	3.74	4.22	3.25	3.25	3.63	3.25	3.39	3.52	3.33	1.83
6F-LN **4**	4.29	3.50	3.50	3.30	3.55	4.77	4.67	4.14	3.32	3.31	3.60	3.46	3.49	3.49	3.32	1.80
2′F-LN **5**	4.22	3.50	3.47	3.38	3.27	3.69	3.53	4.55	4.17	3.63	3.67	3.53	3.50	3.50	3.33	1.79
3′F-LN **6**	4.22	3.49	3.47	3.33	3.27	3.77	3.63	4.26	3.59	4.35	3.88	3.48	3.51	3.48	3.33	1.79
4′F-LN **7**	4.21	3.48	3.45	3.34	3.26	3.77	3.63	4.32	3.27	3.49	4.58	3.62	3.51	3.46	3.33	1.79
6′F-LN **8**	4.23	3.48	3.47	3.34	3.27	3.77	3.63	4.29	3.34	3.35	3.64	3.85	4.57	4.46	3.33	1.79

Table S1B. LacNAc ^{13}C chemical shifts δ [ppm] in DMSO-d_6

	**1**	**2**	**3**	**4**	**5**	**6**	**1′**	**2′**	**3′**	**4′**	**5′**	**6′**	**CO**	**OMe**	**CH_3CO**
LN **2**	101.8	54.5	72.4	81.4	75.1	60.5	104.1	70.7	73.3	68.2	75.7	60.5	168.9	55.8	23.1
3F-LN **3**	100.8	53.5	92.7	75.5	74.4	59.6	103.4	71.0	73.5	67.7	75.1	59.9	169.2	56.0	23.0
6F-LN **4**	101.6	54.3	72.1	80.0	73.0	82.0	104.2	70.5	73.2	68.2	75.7	60.5	168.9	55.8	23.1
2′F-LN **5**	101.7	54.5	72.3	80.7	75.0	59.8	100.7	92.1	71.1	68.9	75.7	60.2	168.8	55.8	23.1
3′F-LN **6**	101.7	54.5	72.4	81.1	75.0	60.3	103.1	68.9	93.6	65.9	74.2	60.0	168.8	55.8	23.1
4′F-LN **7**	101.7	54.6	72.3	81.0	75.0	60.3	103.4	70.8	71.4	89.2	73.6	59.2	168.8	55.8	23.1
6′F-LN **8**	101.7	54.5	72.2	81.1	75.0	60.3	103.7	70.3	72.7	67.9	73.5	83.4	168.8	55.8	23.1

Table S2A. LacNAc ^{1}H chemical shifts differences Δδ [ppm] of unexchangeable protons in DMSO-d_6

	1	2	3	4	5	6_{proS}	6_{proR}	1′	2′	3′	4′	5′	$6'_{proS}$	$6'_{proR}$	OMe	CH_3CO
3F-LN **3**	0.06	0.22	0.94	0.41	0.00	0.01	0.12	0.02	−0.05	−0.05	0.02	−0.19	−0.07	0.03	0.01	0.04
6F-LN **4**	0.08	0.02	0.04	0.01	0.29	1.01	1.05	−0.10	0.02	0.01	−0.01	0.02	0.00	0.03	0.00	0.01
2′F-LN **5**	0.01	0.02	0.01	0.09	0.01	−0.07	−0.09	0.35	0.87	0.33	0.06	0.09	0.01	0.04	0.01	0.00
3′F-LN **6**	0.01	0.01	0.01	0.04	0.01	0.01	0.01	0.06	0.29	1.05	0.27	0.04	0.02	0.02	0.01	0.00
4′F-LN **7**	0.00	0.00	−0.01	0.05	0.00	0.01	0.01	0.12	−0.03	0.19	0.97	0.18	0.02	0.00	0.01	0.00
6′F-LN **8**	0.02	0.00	0.01	0.05	0.01	0.01	0.01	0.09	0.04	0.05	0.03	0.41	1.08	1.00	0.01	0.00

$\Delta\delta = \delta_{\text{LNs } (\mathbf{3}\text{–}\mathbf{8})} - \delta_{\text{LN } \mathbf{2}}$. Color code indicates the number of bonds separating fluorine and the concerned proton, green: separation by 2 bonds (geminal), blue: separation by 3 bonds (vicinal), yellow: separation by 6 or more bonds (long-range).

Table S2B. LacNAc ^{13}C chemical shifts differences Δδ [ppm] in DMSO-d_6

	1	2	3	4	5	6	1′	2′	3′	4′	5′	6′	CO	OMe	MeAc
3F-LN **3**	−1.0	−1.0	20.3	−5.9	−0.7	−0.9	−0.7	0.3	0.2	−0.5	−0.6	−0.6	0.3	0.2	−0.1
6F-LN **4**	−0.2	−0.2	−0.3	−1.4	−2.1	21.5	0.1	−0.2	−0.1	0.0	0.0	0.0	0.0	0.0	0.0
2′F-LN **5**	−0.1	0.0	−0.1	−0.7	0.6	−0.7	−3.4	21.4	−2.2	0.7	−0.7	−0.3	−0.1	0.0	1.0
3′F-LN **6**	−0.1	0.0	0.0	−0.3	−0.1	−0.2	−1.0	−1.8	20.3	−2.3	−1.5	−0.5	−0.1	0.0	0.0
4′F-LN **7**	−0.1	0.1	−0.1	−0.4	−0.1	−0.2	−0.7	0.1	−1.9	21.0	−2.1	−1.3	−0.1	0.0	0.0
6′F-LN **8**	−0.1	0.0	−0.2	−0.3	−0.1	−0.2	−0.4	−0.4	−0.6	−0.3	−2.2	22.9	−0.1	0.0	0.0

$\Delta\delta = \delta_{\text{LNs } (\mathbf{1}\text{–}\mathbf{6})} - \delta_{\text{LacNAc LN } \mathbf{2}}$. Color code indicates the number of bonds separating fluorine and the concerned carbon, green: separation by 1 bond, blue: separation by 2 or 3 bonds, yellow: separation by 5 or more bonds (long-range).

Table S3. LacNAc ^{1}H chemical shifts δ [ppm] of exchangeable protons in DMSO-d_6

**Compound**	NH	O3H	O6H	O2′H	O3′H	O4′H	O6′H
LN **2**	7.78	4.65	-[a]	5.31	5.13	-[a]	4.75
3F-LN **3**	8.00	-	4.79	4.99	4.74	4.41	4.47
6F-LN **4**	7.79	4.71	-	5.17	4.88	4.55	4.67
2′F-LN **5**	7.75	4.45	4.70	-	5.32	4.88	4.73
3′F-LN **6**	7.76	4.54	4.66	5.58	-	5.14	4.78
4′F-LN **7**	7.74	4.46	4.65	5.36	5.32	-	4.92
6′F-LN **8**	7.74	4.44	4.62	5.17	4.94	4.77	-

[a] overlapped at 298 K

Table S4A. Coupling constant ^{3}J(H-n, H-m) [Hz] of protons on GlcNAc ring in DMSO-d_6

Compound	1,2	2,3	3,4	4,5	$5,6_{proR}$	$5,6_{proS}$	$6_{proS},6_{proR}$
LN **2**	8.0	*a*	*a*	9.6	4.7[*b*]	2.2[*b*]	*a*
3F-LN **3**	8.5	10.0	8.5	*a*	4.0[*c*]	2.4[*c*]	*a*
6F-LN **4**	8.1	*a*	*a*	*a*	4.6	1.8	10.2
2′F-LN **5**	8.0	*a*	8.5	9.6	5.1	1.9	*a*
3′F-LN **6**	7.8	*a*	*a*	9.8	5.0[*b*]	2.3[*b*]	11.9
4′F-LN **7**	8.0	*a*	*a*	*a*	4.8[*b*]	2.4[*b*]	11.9
6′F-LN **8**	8.0	*a*	*a*	9.5	4.8	2.2	11.8

[*a*] couplings could not be determined due to spectral overlap; [*b*] Obtained using 1D selective homonuclear decoupling of O6-H; [*c*] Obtained using 1D selective NH–H5 TOCSY transfer;

Table S4B. Coupling constant ^{3}J(H-n, H-m) [Hz] of protons on Gal ring in DMSO-d_6

Compound	1′,2′	2′,3′	3′,4′	4′,5′	$5',6'_{proR}$	$5',6'_{proS}$	$6'_{proS},6'_{proR}$
LN **2**	8.5	10.2	*a*	*a*	*a*	*a*	*a*
3F-LN **3**	*m*	*a*	*a*	*a*	5.9[*b*]	7.1[*b*]	*a*
6F-LN **4**	7.5	*a*	*a*	*a*	6.7[*c*]	5.2[*c*]	*a*
2′F-LN **5**	7.7	9.3	*a*	*a*	6.8[*c*]	5.4[*c*]	*a*
3′F-LN **6**	7.8	9.5	3.5	–	7.3[*d*]	5.7[*d*]	*a*
4′F-LN **7**	7.9	*a*	2.7	*a*	7.3	5.9	*a*
6′F-LN **8**	7.5	*a*	3.3	*a*	7.9[*e*]	3.2[*e*]	9.9

[*a*] couplings could not be determined due to spectral overlap; [*b*] Obtained using 1D selective homonuclear decoupling of O6′-H; [*c*] Obtained using 1D selective H1′–H5′ ROESY transfer; [*d*] Estimated using 1D selective O4′H–H5′ TOCSY transfer; [*e*] Confirmed by ^{1}H measurement at 31 °C. [*m*] signals are multiplet;

Table S5. Coupling constant ^3J(H-n, OH/NH) [Hz] of protons in DMSO-d_6

Compound	2,NH	3,OH	6_{proR},OH	6_{proS},OH	2′,NH	2′,OH	3′,OH	4′,OH	$6'_{proR}$,OH	$6'_{proS}$,OH
LN **2**	8.6	1.8	6.1	6.1	9.3	–	6.4	4.2	5.1	5.1
3F-LN **3**	9.0	–	*br s*	*br s*	–	*br s*	*br s*	4.3	*br s*	*br s*
6F-LN **4**	8.8	*br s*	–	–	–	*br s*	*br s*	4.4	4.8	4.8
2′F-LN **5**	8.4	2.1	5.2	5.2	–	–	5.9	4.7	5.8	5.8
3′F-LN **6**	8.3	1.9	6.0	6.0	–	4.5	–	5.2	5.3	5.3
4′F-LN **7**	8.5	2.1	5.7	5.7	–	5.0	5.6	–	5.4	5.4
6′F-LN **8**	8.4	1.1	6.7	5.8	–	3.8	*br s*	4.5	–	–

br s Signals of protons are broad singlet

Table S6. Coupling constant ^nJ(H-m, F) [Hz] of protons in DMSO-d_6

Compound	1′,F	2′,F	3′,F	4′,F	6_{proR},F	6_{proS},F	5′,F	$6'_{proR}$,F	$6'_{proS}$,F	3,F	2,F	4,F
3F-LN **3**	–	–	–	–	–	–	–	–	–	51.8	13.5	13.5
6F-LN **4**	–	–	–	–	47.8	47.5	29.1	–	–	–	–	–
2′F-LN **5**	3.3	52.1	14.3	3.3	–	–	–	–	–	–	–	–
3′F-LN **6**	–	12.6	48.6	6.4	–	–	–	–	–	–	–	–
4′F-LN **7**	–	–	28.5	50.6	–	–	31.1	–	–	–	–	–
6′F-LN **8**	–	–	–	–	–	–	14.7	49.6	45.5	–	–	–

Temperature Dependent ^{1}H NMR data

Table S7. LN **2**

	^{1}H chemical shift δ [ppm]						
**T [K]**	**NH**	**O3H**	**O6H**	**O2′H**	**O3′H**	**O4′H**	**O6′H**
**298.1**	7.784	4.653	-[a]	5.309	5.133	-[a]	4.749
**300.1**	7.776	4.650	-[a]	5.296	5.115	-[a]	4.740
**302.1**	7.768	4.645	-[a]	5.282	5.099	-[a]	4.731
**304.1**	7.759	4.641	-[a]	5.268	5.082	-[a]	4.721
**306.1**	7.751	4.637	-[a]	5.255	5.066	-[a]	4.712
**308.1**	7.743	4.633	-[a]	5.242	5.052	-[a]	4.702
**310.1**	7.733	4.629	4.598	5.228	5.035	-[a]	4.690
**312.1**	7.726	4.625	4.589	5.215	5.025	-[a]	-[a]
**314.1**	7.716	4.621	4.576	5.200	5.018	-[a]	-[a]
**316.1**	7.709	4.617	4.564	5.186	5.004	-[a]	-[a]
**318.1**	7.700	4.612	4.551	5.173	4.983	-[a]	-[a]
**−ppb/K**	**4.2**	**2.0**	**5.9**	**6.8**	**7.2**	-[a]	**4.9**

[a] Signals could not have been elucidated due to overlap.

Table S8. 3F-LN **3**

	^{1}H chemical shift δ [ppm]						
**T [K]**	**NH**	**O3H**	**O6H**	**O2′H**	**O3′H**	**O4′H**	**O6′H**
**298.1**	7.998	-	4.789	4.988	4.736	4.408	4.467
**300.1**	7.990	-	4.778	4.976	4.722	4.394	4.456
**302.1**	7.982	-	4.767	4.964	4.709	4.383	4.446
**304.1**	7.974	-	4.755	4.952	4.694	4.373	4.433
**306.1**	7.966	-	4.747	4.940	4.681	4.363	4.423
**308.1**	7.958	-	4.736	4.928	4.668	4.348	4.409
**−ppb/K**	**4.0**	**-**	**5.3**	**6.0**	**6.8**	**5.8**	**5.7**

Table S9. 6F-LN **4**

	^{1}H chemical shift δ [ppm]						
**T [K]**	**NH**	**O3H**	**O6H**	**O2′H**	**O3′H**	**O4′H**	**O6′H**
**298.1**	7.793	4.707	-	5.171	4.876	4.550	-[a]
**300.1**	7.784	4.703	-	5.158	4.861	4.538	4.661
**302.1**	7.777	4.699	-	5.146	4.846	4.526	4.652
**304.1**	7.768	4.694	-	5.133	4.831	4.514	4.643
**306.1**	7.760	4.690	-	5.120	4.815	4.502	4.633
**308.1**	7.751	4.686	-	5.108	4.802	4.490	4.624
**323.1**	7.689	4.653	-	5.015	-[a]	4.402	4.554
**−ppb/K**	**4.2**	**2.2**	**-**	**6.2**	**7.5**	**5.9**	**4.7**

[a] Signals could not have been elucidated due to overlap.

Table S10. 2′F-LN **5**

	^{1}H chemical shift δ [ppm]						
**T [K]**	**NH**	**O3H**	**O6H**	**O2′H**	**O3′H**	**O4′H**	**O6′H**
**298.1**	7.745	4.454	4.702	-	5.321	4.884	4.726
**300.1**	7.737	4.449	4.690	-	5.309	4.873	4.717
**302.1**	7.729	4.444	4.679	-	5.298	4.86	4.709
**304.1**	7.721	4.438	4.667	-	5.285	4.848	4.700
**306.1**	7.712	4.433	4.655	-	5.274	4.836	4.691
**308.1**	7.703	4.428	4.643	-	5.261	4.823	4.682
**−ppb/K**	**4.2**	**2.6**	**5.9**	**-**	**6.0**	**6.1**	**4.4**

Table S11. 3′F-LN **6**

	^{1}H chemical shift δ [ppm]						
**T [K]**	**NH**	**O3H**	**O6H**	**O2′H**	**O3′H**	**O4′H**	**O6′H**
**298.1**	7.762	4.549	4.672	5.592	-	5.155	4.796
**300.1**	7.754	4.546	4.661	5.583	-	5.145	4.787
**302.1**	7.747	4.542	4.650	5.573	-	5.134	4.777
**304.1**	7.739	4.539	4.639	5.563	-	5.124	4.767
**306.1**	7.730	4.535	4.628	5.553	-	5.114	4.758
**308.1**	7.721	4.532	4.617	5.543	-	5.103	4.748
**323.1**	7.659	4.506	4.533	5.469	-	5.025	4.675
**−ppb/K**	**4.1**	**1.7**	**5.6**	**4.9**	**-**	**5.2**	**4.9**

Table S12. 4′F-LN **7**

	^{1}H chemical shift δ [ppm]						
**T [K]**	**NH**	**O3H**	**O6H**	**O2′H**	**O3′H**	**O4′H**	**O6′H**
**298.1**	7.744	4.461	4.648	5.363	5.324	-	4.917
**300.1**	7.736	4.456	4.636	5.352	5.312	-	4.908
**302.1**	7.728	4.453	4.625	5.341	5.300	-	4.899
**304.1**	7.720	4.449	4.614	5.327	5.288	-	4.890
**306.1**	7.711	4.444	4.604	5.316	5.276	-	4.882
**308.1**	7.703	4.440	4.593	5.304	5.264	-	4.873
**−ppb/K**	**4.1**	**2.1**	**5.5**	**6.0**	**6.0**	**-**	**4.4**

Table S13. 6′F-LN **8**

	^{1}H chemical shift δ [ppm]						
**T [K]**	**NH**	**O3H**	**O6H**	**O2′H**	**O3′H**	**O4′H**	**O6′H**
**298.1**	7.744	4.441	4.619	5.170	4.937	4.767	-
**300.1**	7.736	4.437	4.608	5.159	4.923	4.755	-
**302.1**	7.729	4.433	4.597	5.148	4.908	4.745	-
**304.1**	7.721	4.429	4.586	5.137	4.893	4.733	-
**306.1**	7.712	4.426	4.576	5.126	4.879	4.721	-
**308.1**	7.703	4.422	4.565	5.115	4.865	4.710	-
**310.1**	7.695	4.418	4.554	5.104	4.851	4.698	-
**−ppb/K**	**4.1**	**1.9**	**5.4**	**5.5**	**7.2**	**5.7**	

DFT calculations

Optimization of Molecular Geometries

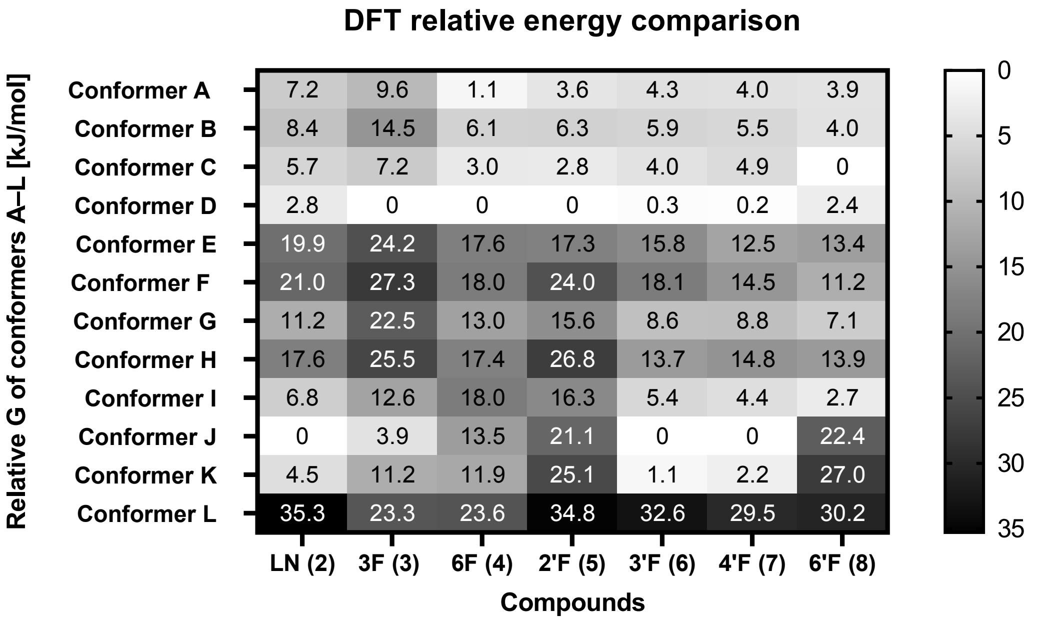

Figure S14. Comparison of the relative zero-point corrected energies calculated by DFT of the conformers A–L in compounds **2**–**8**. The energies are referenced to the most stable conformer of each compound.

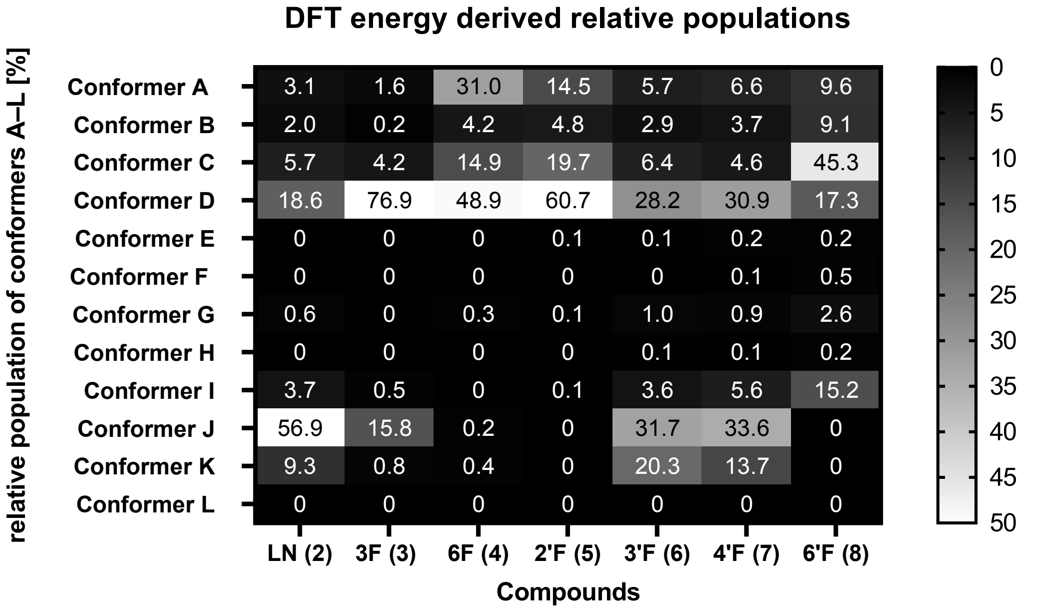

Figure S15. Comparison of the Boltzmann distribution-derived populations of conformers A–L in compounds **2**–**8**.

Calculation of ^{13}C chemical shifts

Table S14. Experimental and DFT-calculated ^{13}C NMR parameters (δ_{exp}, σ_{ISO} and δ_{theor}) corresponding to conformers **A–L** of LN **2**.

Conformer		A		B		C		D		E		F		G		H		I		J		K		L	
carbon	δ_{exp}	σ_{ISO}	δ_{teor}	σ_{ISO}	δ_{teor}	σ_{ISO}	δ_{teor}	σ_{ISO}	δ_{teor}	σ_{ISO}	δ_{teor}	σ_{ISO}	δ_{teor}	σ_{ISO}	δ_{teor}	σ_{ISO}	δ_{teor}	σ_{ISO}	δ_{teor}	σ_{ISO}	δ_{teor}	σ_{ISO}	δ_{teor}	σ_{ISO}	δ_{teor}
C-6′	60.5	119.4	60.1	119.1	59.7	115.7	62.6	115.6	63.2	114.6	63.9	114.7	63.3	119.3	60.0	119.0	61.3	119.5	60.7	115.0	62.7	119.6	59.1	115.0	63.1
C-5′	75.7	105.2	73.6	104.0	74.1	101.7	76.1	102.1	76.3	105.2	72.9	105.1	72.5	105.2	73.6	105.1	74.7	103.9	75.7	101.4	75.8	105.0	73.0	105.9	72.1
C-1′	104.1	74.1	103.2	73.6	103.2	75.9	101.0	76.5	100.9	74.3	102.6	73.9	102.6	74.4	103.4	82.5	96.6	77.0	101.5	73.8	102.3	73.5	103.0	78.1	99.2
C-4	81.4	97.4	81.0	93.4	84.3	97.1	80.6	100.7	77.6	97.6	80.2	93.8	83.4	96.5	82.0	101.5	78.2	106.9	72.8	87.8	88.8	87.7	89.5	95.9	81.9
C-3	72.4	105.1	73.7	104.6	73.5	104.6	73.3	104.9	73.5	104.9	73.2	104.6	73.0	110.1	68.9	108.7	71.2	103.9	75.7	104.2	73.1	104.3	73.7	103.7	74.1
C-2	54.5	124.5	55.2	125.2	53.9	125.0	53.6	124.6	54.6	124.4	54.4	125.0	53.3	117.9	61.4	125.3	55.1	125.1	55.4	125.2	53.0	125.1	53.8	124.6	53.8
CO	168.9	5.3	168.8	5.3	168.4	5.3	169.2	5.4	169.5	5.3	169.0	5.3	168.7	4.7	170.6	5.3	171.4	5.4	170.3	5.3	168.1	5.3	168.1	5.3	170.3
MeAc	23.1	158.9	22.4	158.9	21.7	158.9	20.9	158.9	21.5	158.9	21.3	158.9	20.7	158.9	21.8	158.9	22.6	158.9	22.9	158.9	20.6	158.9	21.6	158.9	20.3
C-1	101.8	75.0	102.4	75.8	101.0	75.7	101.3	74.9	102.5	75.0	101.9	75.8	100.8	79.4	98.5	74.9	104.0	75.3	103.2	75.1	101.0	75.1	101.5	76.4	100.9
OMe	55.8	124.4	55.3	124.4	54.7	124.2	54.4	124.2	54.9	124.3	54.5	124.3	54.0	124.6	55.0	124.3	56.1	124.4	56.1	124.3	53.8	124.4	54.5	124.5	53.8
C-5	75.1	104.5	74.2	103.6	74.5	103.6	74.4	104.7	73.7	104.5	73.6	103.5	74.1	104.4	74.4	106.6	73.3	103.3	76.3	107.8	69.6	107.8	70.3	106.9	71.0
C-6	60.5	118.6	60.8	115.0	63.7	115.4	62.9	118.5	60.5	118.5	60.1	114.9	63.0	118.6	60.7	117.8	62.4	118.5	61.7	112.8	64.8	112.9	65.5	111.2	66.9
C-2′	70.7	108.0	70.9	108.0	70.3	107.9	70.2	107.9	70.7	108.5	69.7	108.5	69.2	108.0	70.9	106.5	73.3	111.0	68.9	108.0	69.4	107.6	70.4	107.7	70.3
C-3′	73.3	102.3	76.4	102.4	75.7	102.1	75.8	102.0	76.4	102.1	75.9	102.2	75.3	102.2	76.5	102.7	77.1	103.4	76.2	103.2	74.0	103.0	74.9	102.2	75.6
C-4′	68.2	111.1	68.0	111.2	67.3	108.5	69.6	108.5	70.1	105.3	72.8	105.5	72.1	110.9	68.1	111.2	68.8	111.2	68.7	108.6	68.9	111.2	67.1	105.2	72.7
	SLOPE	−1.0492		−1.0469		−1.0357		−1.0372		−1.0398		−1.0381		−1.0367		−1.0325		−1.0414		−1.0418		−1.0490		−1.0231	
	INTERCEPT	182.4124		181.6135		180.5783		181.1880		181.0147		180.3652		181.5501		182.2296		182.7412		180.3555		181.5456		179.6032	
	**MSD [ppm^2]**	**1.3**		**2.3**		**2.5**		**3.5**		**4.0**		**4.4**		**6.2**		**7.3**		**7.4**		**8.6**		**8.9**		**9.7**	

δ_{exp} = experimentally obtained ^{13}C chemical shifts, σ_{ISO} = DFT-calculated isotropic magnetic shielding, δ_{teor} = theoretical ^{13}C chemical shifts obtained by the linear regression approach. SLOPE and INTERCEPT = coefficients of linear regression according to the equation 1. MSD = mean square deviations between δ_{exp} and δ_{teor} corresponding to the conformers **A–L** (Figure 9).

Table S15. Experimental and DFT-calculated ^{13}C NMR parameters (δ_{exp}, σ_{ISO} and δ_{theor}) corresponding to conformers **A**–**L** of 3F-LN **3**.

Conformer		A		B		C		D		E		F		G		H		I		J		K		L	
carbon	δ_{exp}	σ_{ISO}	δ_{teor}	σ_{ISO}	δ_{teor}	σ_{ISO}	δ_{teor}	σ_{ISO}	δ_{teor}	σ_{ISO}	δ_{teor}	σ_{ISO}	δ_{teor}	σ_{ISO}	δ_{teor}	σ_{ISO}	δ_{teor}	σ_{ISO}	δ_{teor}	σ_{ISO}	δ_{teor}	σ_{ISO}	δ_{teor}	σ_{ISO}	δ_{teor}
**C-6′**	59.9	119.0	60.3	119.0	59.7	116.8	61.2	115.6	62.6	113.7	64.4	114.0	63.7	118.8	60.0	118.9	60.7	119.6	60.2	116.4	60.8	118.9	58.9	113.6	63.1
**C-5′**	75.1	105.2	73.4	105.1	73.0	103.2	74.3	102.1	75.5	104.8	73.0	104.8	72.5	105.8	72.6	105.9	73.4	104.3	74.8	102.8	73.7	104.8	72.3	104.4	71.9
**C-1′**	103.4	76.1	101.1	76.2	100.7	74.3	102.0	75.0	101.4	75.5	101.1	75.9	100.5	75.8	101.5	81.6	96.9	76.6	101.2	71.8	103.4	70.6	104.9	70.0	105.0
**C-4**	75.5	103.5	75.0	100.3	77.6	99.0	78.3	102.1	75.5	103.4	74.3	100.3	76.9	102.6	75.6	103.9	75.3	110.3	69.0	91.3	84.8	91.5	85.0	91.3	84.5
**C-3**	92.7	80.3	97.1	80.3	96.8	81.0	95.5	79.1	97.4	80.2	96.6	80.5	96.1	83.6	94.0	85.8	92.8	81.5	96.5	80.5	95.1	80.7	95.3	80.9	94.5
**C-2**	53.5	126.2	53.4	126.3	52.7	125.8	52.6	126.3	52.4	126.2	52.5	126.3	51.8	119.6	59.2	125.3	54.5	125.5	54.5	126.0	51.5	126.1	52.0	126.0	51.2
**CO**	169.2	5.1	168.7	5.2	168.8	5.1	168.3	5.1	168.2	5.1	168.7	5.1	168.9	4.2	170.6	5.1	171.0	5.1	169.3	5.1	167.3	5.1	167.2	5.1	167.2
**MeAc**	23.0	159.0	22.1	159.0	21.4	159.0	20.7	159.0	21.1	159.0	20.9	159.0	20.2	159.0	21.2	158.9	22.0	159.0	22.6	159.0	19.9	159.0	20.8	159.0	19.5
**C-1**	100.8	76.5	100.8	76.8	100.1	76.7	99.7	76.5	100.0	76.3	100.3	76.8	99.6	79.9	97.6	76.7	101.6	76.3	101.5	75.9	99.5	76.2	99.5	76.2	99.0
**OMe**	56.0	124.2	55.3	124.2	54.7	124.1	54.2	124.1	54.4	124.2	54.4	124.3	53.8	124.5	54.5	124.2	55.6	124.2	55.8	124.0	53.5	124.0	54.1	124.0	53.1
**C-5**	74.4	105.2	73.4	104.5	73.6	103.9	73.5	105.0	72.7	105.3	72.5	104.4	72.9	105.2	73.1	106.0	73.2	103.9	75.2	106.6	70.1	106.9	70.3	106.8	69.6
**C-6**	59.6	118.2	61.0	114.6	64.0	115.3	62.6	118.0	60.3	118.2	60.1	114.5	63.2	118.4	60.4	118.1	61.5	118.9	60.9	111.4	65.5	111.5	66.0	111.6	65.0
**C-2′**	71.0	105.8	72.9	105.7	72.4	105.8	71.8	105.5	72.3	106.2	71.6	106.2	71.2	107.7	70.7	108.2	71.1	110.2	69.1	106.5	70.2	106.4	70.9	107.1	69.4
**C-3′**	73.5	104.4	74.2	104.5	73.6	104.5	73.0	104.6	73.1	104.3	73.5	104.3	73.0	101.8	76.5	102.0	77.1	102.1	76.9	103.4	73.2	104.2	72.9	104.0	72.3
**C-4′**	67.7	112.1	66.8	112.2	66.3	110.0	67.8	109.5	68.4	106.4	71.5	106.4	71.0	110.9	67.7	111.0	68.4	111.4	68.0	110.0	66.8	112.4	65.1	106.7	69.7
	**SLOPE**	−1.0501		−1.0441		−1.0432		−1.0469		−1.0419		−1.0357		−1.0364		−1.0318		−1.0493		−1.0439		−1.0506		−1.0419	
	**INTERCEPT**	182.2648		181.3527		180.6444		181.1276		180.8306		179.9541		181.0070		181.5750		182.7402		179.7814		180.8138		179.3533	
	**MSD [ppm²]**	**2.5**		**4.1**		**2.8**		**3.2**		**4.9**		**5.7**		**4.9**		**4.7**		**5.4**		**11.7**		**12.6**		**13.3**	

δ_{exp} = experimentally obtained ^{13}C chemical shifts, σ_{ISO} = DFT-calculated isotropic magnetic shielding, δ_{teor} = theoretical ^{13}C chemical shifts obtained via linear regression approach. SLOPE and INTERCEPT = coefficients of linear regression according to the equation 1. MSD = mean square deviations between δ_{exp} and δ_{teor} corresponding to the conformers **A**–**L** (Figure 9).

Table S16. Experimental and DFT-calculated ^{13}C NMR parameters (δ_{exp}, σ_{ISO} and δ_{theor}) corresponding to conformers **A**–**L** of 6F-LN **4**.

Conformer		A		B		C		D		E		F		G		H		I		J		K		L	
carbon	δ_{exp}	σ_{ISO}	δ_{teor}	σ_{ISO}	δ_{teor}	σ_{ISO}	δ_{teor}	σ_{ISO}	δ_{teor}	σ_{ISO}	δ_{teor}	σ_{ISO}	δ_{teor}	σ_{ISO}	δ_{teor}	σ_{ISO}	δ_{teor}	σ_{ISO}	δ_{teor}	σ_{ISO}	δ_{teor}	σ_{ISO}	δ_{teor}	σ_{ISO}	δ_{teor}
**C-6’**	60.5	119.3	60.0	119.4	59.6	115.8	62.6	115.7	62.9	114.9	63.4	115.0	63.1	119.4	59.6	119.0	61.0	119.0	61.1	115.4	62.6	119.2	59.5	114.4	63.3
**C-5’**	75.7	103.4	75.2	103.3	74.9	101.0	76.8	101.2	76.9	104.6	73.4	104.6	73.2	105.1	73.4	105.0	74.5	104.4	75.1	100.6	76.7	103.5	74.3	104.4	72.8
**C-1’**	104.2	74.7	102.5	74.5	102.4	75.9	100.9	76.2	101.0	74.6	102.2	74.7	101.9	73.9	103.4	82.5	96.2	77.8	100.7	75.0	101.2	72.8	103.3	72.7	103.0
**C-4**	80.0	98.5	79.8	96.3	81.7	98.6	79.2	100.6	77.5	98.0	79.8	96.5	80.9	95.9	82.2	101.5	77.9	107.6	72.1	91.1	85.8	89.0	88.0	89.0	87.5
**C-3**	72.1	105.2	73.4	104.9	73.5	104.7	73.3	105.0	73.3	105.1	72.9	104.7	73.0	110.1	68.6	109.0	70.6	103.8	75.7	104.8	72.7	104.5	73.4	104.3	72.9
**C-2**	54.3	124.8	54.8	125.3	54.0	125.4	53.4	124.9	54.1	124.8	53.9	125.2	53.4	118.4	60.6	125.5	54.7	125.7	54.7	125.2	53.2	125.7	53.4	125.5	52.7
**CO**	168.9	5.3	168.7	5.3	168.3	5.3	168.8	5.3	169.2	5.3	168.9	5.3	168.6	4.6	170.1	5.3	170.8	5.3	170.2	5.3	167.8	5.2	167.1	5.2	167.3
**MeAc**	23.1	159.0	22.2	159.0	21.9	159.0	21.1	158.9	21.3	158.9	21.2	159.0	20.9	159.0	21.6	158.9	22.5	158.9	22.8	159.0	20.9	158.9	22.0	158.9	20.9
**C-1**	101.6	75.2	102.0	76.0	101.0	75.7	101.1	75.0	102.1	75.2	101.6	76.0	100.7	79.3	98.2	74.8	103.6	75.4	102.9	74.9	101.3	74.9	101.4	74.7	101.1
**OMe**	55.8	124.2	55.3	124.2	55.0	124.1	54.6	124.1	54.9	124.2	54.6	124.3	54.3	124.4	54.8	124.1	56.1	124.3	56.0	124.1	54.3	124.2	54.9	124.1	54.1
**C-5**	73.0	105.5	73.2	104.9	73.5	105.0	73.0	105.5	72.8	105.3	72.7	104.8	72.9	105.1	73.4	107.1	72.5	104.6	75.0	110.3	67.5	110.5	67.7	110.4	67.1
**C-6**	82.0	95.4	82.8	92.6	85.1	92.9	84.6	95.4	82.5	95.3	82.3	92.5	84.8	95.3	82.9	94.7	84.5	96.4	82.8	91.1	85.8	90.1	87.0	90.2	86.4
**C-2’**	70.5	105.9	72.8	105.9	72.5	105.9	72.1	105.9	72.4	106.4	71.6	106.4	71.4	108.0	70.6	106.7	72.9	110.8	69.0	106.2	71.3	106.6	71.5	107.0	70.3
**C-3’**	73.2	105.2	73.5	105.2	73.2	105.1	72.9	105.0	73.3	105.2	72.9	105.2	72.5	102.4	76.0	102.7	76.7	103.3	76.2	103.8	73.7	104.3	73.6	104.1	73.1
**C-4’**	68.2	112.1	66.9	112.2	66.5	109.5	68.6	109.5	68.9	106.3	71.7	106.4	71.5	111.0	67.7	111.2	68.6	110.9	68.9	109.4	68.3	112.5	65.9	106.7	70.6
	**SLOPE**	−1.0485		−1.0500		−1.0402		−1.0392		−1.0401		−1.0404		−1.0391		−1.0363		−1.0420		−1.0460		−1.0598		−1.0500	
	**INTERCEPT**	182.1997		181.9974		180.9230		181.1271		180.9320		180.7158		181.3563		182.2123		182.6867		180.8515		182.2963		180.8517	
	**MSD [ppm^2]**	**1.0**		**1.9**		**2.3**		**2.3**		**2.5**		**3.3**		**5.9**		**7.0**		**7.2**		**7.0**		**9.0**		**9.8**	

δ_{exp} = experimentally obtained ^{13}C chemical shifts, σ_{ISO} = DFT-calculated isotropic magnetic shielding, δ_{teor} = theoretical ^{13}C chemical shifts obtained via linear regression approach. SLOPE and INTERCEPT = coefficients of linear regression according to the equation 1. MSD = mean square deviations between δ_{exp} and δ_{teor} corresponding to the conformers **A**–**L** (Figure 9).

Table S17. Experimental and DFT-calculated ^{13}C NMR parameters (δ_{exp}, σ_{ISO} and δ_{theor}) corresponding to conformers **A–L** of 2′F-LN **5**.

Conformer		A		B		C		D		E		F		G		H		I		J		K		L	
carbon	δ_{exp}	σ_{ISO}	δ_{teor}	σ_{ISO}	δ_{teor}	σ_{ISO}	δ_{teor}	σ_{ISO}	δ_{teor}	σ_{ISO}	δ_{teor}	σ_{ISO}	δ_{teor}	σ_{ISO}	δ_{teor}	σ_{ISO}	δ_{teor}	σ_{ISO}	δ_{teor}	σ_{ISO}	δ_{teor}	σ_{ISO}	δ_{teor}	σ_{ISO}	δ_{teor}
C-6′	60.2	119.9	59.4	119.9	58.8	116.1	62.0	116.1	62.5	115.1	63.1	115.1	62.4	119.6	59.6	119.2	60.9	119.7	60.2	116.3	61.7	119.7	59.1	115.4	62.5
C-5′	75.7	104.7	73.8	104.7	73.2	101.0	76.4	101.3	76.7	104.7	73.1	104.7	72.5	105.4	73.3	105.5	74.1	104.3	75.0	101.8	75.7	104.9	73.3	105.3	72.3
C-1′	100.7	76.7	100.3	76.5	100.1	78.6	98.0	78.9	98.2	77.0	99.6	76.8	99.3	76.7	100.9	84.1	94.6	78.7	99.5	79.7	97.1	79.5	97.8	80.1	96.8
C-4	80.7	96.7	81.4	92.8	84.6	96.3	80.9	100.4	77.6	97.2	80.3	93.4	83.3	96.5	81.8	101.4	78.0	106.6	72.7	94.0	83.3	94.2	83.6	94.6	82.7
C-3	72.3	105.1	73.4	104.6	73.4	104.7	72.9	105.1	73.1	105.2	72.7	104.6	72.5	110.2	68.6	108.9	70.8	103.8	75.4	104.2	73.4	104.5	73.7	104.1	73.5
C-2	54.5	125.1	54.4	125.3	53.6	125.1	53.3	124.9	54.1	124.8	53.9	125.2	52.8	117.9	61.2	125.5	54.8	125.6	54.6	125.5	52.8	124.9	54.1	124.8	53.3
CO	168.8	5.3	168.0	5.3	167.8	5.3	168.4	5.4	168.7	5.3	168.3	5.3	168.0	4.7	170.2	5.3	170.3	5.3	169.7	5.3	169.0	5.3	169.0	5.3	169.3
MeAc	23.1	158.9	22.4	158.9	21.6	158.9	20.8	158.9	21.5	158.9	21.2	158.9	20.4	158.9	21.7	158.9	22.7	158.9	22.7	158.9	20.5	158.9	21.4	158.9	20.3
C-1	101.7	75.4	101.5	75.7	100.9	75.4	101.0	75.2	101.8	75.3	101.2	75.6	100.4	79.4	98.2	75.5	102.9	75.4	102.6	76.4	100.3	76.4	100.7	76.4	100.3
OMe	55.8	124.3	55.2	124.2	54.7	124.1	54.3	124.1	54.8	124.2	54.4	124.3	53.7	124.6	54.8	124.3	56.0	124.3	55.8	124.4	53.9	124.4	54.5	124.5	53.7
C-5	75.0	104.6	73.9	104.1	73.8	104.0	73.6	104.6	73.6	104.5	73.3	102.3	74.8	104.7	73.9	106.3	73.3	103.5	75.7	107.5	70.3	107.3	71.0	107.3	70.4
C-6	59.8	118.6	60.6	114.7	63.8	115.2	62.8	118.6	60.1	118.6	59.9	114.3	63.2	118.5	60.6	118.0	62.0	118.7	61.2	111.5	66.3	111.5	66.9	111.4	66.4
C-2′	92.1	82.8	94.5	82.9	94.0	82.9	93.8	82.8	94.4	83.2	93.7	83.2	93.1	85.2	92.6	83.4	95.2	86.8	91.7	83.1	93.8	82.9	94.5	83.0	94.0
C-3′	71.1	105.2	73.3	105.3	72.7	105.2	72.4	105.1	73.1	105.1	72.7	105.3	71.9	104.8	73.8	104.9	74.6	105.3	74.0	105.1	72.6	105.1	73.1	105.2	72.4
C-4′	68.9	110.8	68.0	110.9	67.4	108.1	69.6	108.1	70.2	105.0	72.8	105.3	71.9	109.5	69.3	109.4	70.3	109.8	69.6	108.1	69.6	110.6	67.9	104.9	72.7
	SLOPE	−1.0545		−1.0506		−1.0402		−1.0433		−1.0444		−1.0406		−1.0392		−1.0411		−1.0453		−1.0347		−1.0402		−1.0304	
	INTERCEPT	182.5160		181.6627		180.5324		181.3376		181.0765		180.1155		181.4926		182.5866		182.6451		180.1590		181.1612		179.7788	
	**MSD [ppm^2]**	**1.4**		**3.7**		**2.5**		**2.7**		**3.1**		**4.3**		**6.2**		**5.7**		**5.9**		**7.3**		**7.3**		**9.2**	

δ_{exp} = experimentally obtained ^{13}C chemical shifts, σ_{ISO} = DFT calculated isotropic magnetic shielding, δ_{teor} = theoretical ^{13}C chemical shifts obtained via linear regression approach. SLOPE and INTERCEPT = coefficients of linear regression according to the equation 1. MSD = mean square deviations between δ_{exp} and δ_{teor} corresponding to the conformers **A**–**L** (Figure 9).

Table S18. Experimental and DFT-calculated ^{13}C NMR parameters (δ_{exp}, σ_{ISO} and δ_{theor}) corresponding to conformers **A**–**L** of 3′F-LN **6**.

Conformer		A		B		C		D		E		F		G		H		I		J		K		L	
carbon	δ_{exp}	σ_{ISO}	δ_{teor}	σ_{ISO}	δ_{teor}	σ_{ISO}	δ_{teor}	σ_{ISO}	δ_{teor}	σ_{ISO}	δ_{teor}	σ_{ISO}	δ_{teor}	σ_{ISO}	δ_{teor}	σ_{ISO}	δ_{teor}	σ_{ISO}	δ_{teor}	σ_{ISO}	δ_{teor}	σ_{ISO}	δ_{teor}	σ_{ISO}	δ_{teor}
C-6′	60.0	119.6	59.4	119.7	58.8	115.9	62.0	115.9	62.6	114.8	63.3	114.9	62.6	119.9	59.1	119.5	60.4	120.1	59.8	115.2	62.1	119.8	58.4	115.3	62.4
C-5′	74.2	105.2	73.1	105.2	72.6	103.0	74.5	103.4	74.6	106.5	71.2	106.5	70.8	106.3	72.2	106.3	73.2	105.0	74.3	102.3	74.4	105.0	72.5	107.2	70.4
C-1′	103.1	75.0	101.7	74.5	101.7	76.7	99.7	77.3	99.6	75.3	101.1	74.8	101.1	75.3	101.9	83.3	95.3	77.7	100.2	74.7	100.9	74.3	101.6	79.1	97.7
C-4	81.1	97.3	80.6	93.1	84.0	96.6	80.6	100.3	77.5	97.4	80.0	93.4	83.3	96.2	81.9	101.4	77.9	106.7	72.6	87.5	88.7	87.3	89.3	95.6	81.7
C-3	72.4	105.1	73.2	104.5	73.2	104.7	72.9	104.9	73.1	104.9	72.7	104.5	72.6	110.1	68.5	109.1	70.5	104.1	75.1	104.3	72.6	104.3	73.1	103.7	73.7
C-2	54.5	124.6	54.7	125.3	53.5	125.1	53.2	124.6	54.2	124.5	54.0	125.1	52.9	117.9	61.0	125.4	54.8	125.2	55.0	125.1	52.6	125.2	53.3	124.7	53.3
CO	168.8	5.3	167.8	5.3	167.5	5.3	168.3	5.4	168.6	5.3	168.1	5.3	167.9	4.7	169.5	5.3	170.3	5.3	169.3	5.3	167.5	5.2	167.3	5.3	169.5
MeAc	23.1	158.9	22.2	159.0	21.5	158.9	20.7	158.9	21.3	158.9	21.1	158.9	20.4	158.9	21.7	158.9	22.5	158.9	22.8	158.9	20.2	158.9	21.2	158.9	20.0
C-1	101.7	75.0	101.7	75.9	100.4	75.7	100.7	74.9	101.9	75.0	101.4	75.8	100.2	79.4	97.9	75.0	103.2	75.3	102.5	75.1	100.5	75.2	100.8	76.4	100.3
OMe	55.8	124.3	54.9	124.4	54.4	124.2	54.1	124.2	54.6	124.3	54.2	124.3	53.6	124.5	54.7	124.3	55.9	124.3	55.8	124.3	53.4	124.4	54.0	124.5	53.5
C-5	75.0	104.6	73.6	103.6	74.1	103.6	73.8	104.8	73.2	104.5	73.1	103.5	73.6	104.5	73.9	106.5	72.9	103.4	75.7	107.9	69.1	107.9	69.8	107.0	70.5
C-6	60.3	118.7	60.3	115.0	63.2	115.5	62.5	118.5	60.1	118.6	59.6	115.0	62.6	118.7	60.3	117.9	62.0	118.5	61.3	112.8	64.4	112.8	65.1	111.3	66.4
C-2′	68.9	107.6	70.8	107.6	70.3	107.6	70.0	107.6	70.5	108.2	69.6	108.2	69.1	107.7	70.8	106.3	73.2	110.7	68.8	107.8	69.2	107.4	70.2	107.4	70.2
C-3′	93.6	78.7	98.2	78.9	97.6	79.0	97.5	78.8	98.1	78.9	97.7	79.1	97.0	78.6	98.6	79.2	99.2	80.0	98.1	80.4	95.5	79.8	96.5	79.0	97.8
C-4′	65.9	112.3	66.4	112.5	65.6	109.8	67.9	109.7	68.5	106.5	71.2	106.7	70.6	112.2	66.5	112.4	67.3	112.6	67.0	109.9	67.2	112.5	65.3	106.3	71.2
	SLOPE	−1.0547		−1.0523		−1.0405		−1.0426		−1.0440		−1.0417		−1.0436		−1.0401		−1.0482		−1.0430		−1.0524		−1.0276	
	INTERCEPT	182.2663		181.5558		180.4670		181.1340		180.8644		180.1781		181.5854		182.3669		182.8071		179.9735		181.2680		179.4690	
	MSD [ppm^2]	**2.3**		**3.5**		**3.6**		**4.7**		**5.5**		**5.7**		**7.5**		**9.3**		**7.4**		**9.7**		**9.8**		**11.6**	

δ_{exp} = experimentally obtained ^{13}C chemical shifts, σ_{ISO} = DFT-calculated isotropic magnetic shielding, δ_{teor} = theoretical ^{13}C chemical shifts obtained via linear regression approach. SLOPE and INTERCEPT = coefficients of linear regression according to the equation 1. MSD = mean square deviations between δ_{exp} and δ_{teor} corresponding to the conformers **A**–**L** (Figure 9).

Table S19. Experimental and DFT-calculated ^{13}C NMR parameters (δ_{exp}, σ_{ISO} and δ_{theor}) corresponding to conformers **A**–**L** of 4′F-LN **7**.

Conformer		A		B		C		D		E		F		G		H		I		J		K		L	
carbon	δ_{exp}	σ_{ISO}	δ_{teor}	σ_{ISO}	δ_{teor}	σ_{ISO}	δ_{teor}	σ_{ISO}	δ_{teor}	σ_{ISO}	δ_{teor}	σ_{ISO}	δ_{teor}	σ_{ISO}	δ_{teor}	σ_{ISO}	δ_{teor}	σ_{ISO}	δ_{teor}	σ_{ISO}	δ_{teor}	σ_{ISO}	δ_{teor}	σ_{ISO}	δ_{teor}
**C-6′**	59.2	119.8	59.4	119.6	59.0	116.5	61.7	116.3	62.3	114.6	63.7	114.6	63.1	119.7	59.3	119.4	60.6	120.0	60.0	115.9	61.7	120.0	58.4	115.0	62.9
**C-5′**	73.6	105.8	72.7	104.8	73.1	102.6	75.0	103.0	75.2	105.8	72.1	105.7	71.6	105.8	72.7	105.7	73.8	104.4	74.9	102.4	74.6	105.6	72.1	106.4	71.3
**C-1′**	103.4	74.2	102.8	73.5	102.9	76.0	100.7	76.6	100.5	74.5	102.0	73.8	102.2	74.4	103.0	82.5	96.2	77.1	101.1	73.8	102.0	73.6	102.7	77.9	98.9
**C-4**	81.0	97.3	80.9	93.1	84.2	96.9	80.5	100.6	77.4	97.8	79.7	93.6	83.2	96.3	81.9	101.4	78.0	106.7	72.7	87.7	88.7	87.5	89.4	95.4	81.9
**C-3**	72.3	105.1	73.4	104.7	73.2	104.6	73.1	104.9	73.3	105.0	72.9	104.5	72.8	110.1	68.6	108.7	70.9	103.9	75.4	104.2	72.9	104.3	73.4	103.9	73.7
**C-2**	54.6	124.6	54.8	125.2	53.5	125.1	53.3	124.7	54.3	124.4	54.3	125.1	53.1	117.9	61.1	125.4	54.8	125.1	55.1	125.2	52.7	125.1	53.5	124.7	53.5
**CO**	168.8	5.3	168.4	5.3	168.1	5.3	168.7	5.4	169.0	5.3	168.2	5.3	167.9	4.7	170.2	5.3	170.8	5.3	169.9	5.3	167.7	5.3	167.8	5.3	169.3
**MeAc**	23.1	158.9	22.1	159.0	21.3	158.9	20.8	158.9	21.4	158.9	21.3	158.9	20.7	158.9	21.6	158.9	22.4	158.9	22.7	158.9	20.4	158.9	21.2	158.9	20.3
**C-1**	101.7	75.0	102.0	75.9	100.7	75.7	101.0	74.9	102.2	75.0	101.5	75.8	100.4	79.4	98.2	74.9	103.6	75.3	102.8	75.1	100.7	75.1	101.2	76.5	100.3
**OMe**	55.8	124.4	55.0	124.4	54.4	124.2	54.2	124.2	54.7	124.3	54.4	124.3	53.8	124.5	54.7	124.2	55.9	124.3	55.8	124.3	53.6	124.4	54.2	124.5	53.7
**C-5**	75.0	104.6	73.9	103.6	74.2	103.6	74.0	104.8	73.4	104.5	73.3	103.5	73.8	104.4	74.1	106.6	72.9	103.4	75.9	107.8	69.4	107.8	70.0	107.0	70.7
**C-6**	60.3	118.6	60.5	115.0	63.3	115.5	62.7	118.5	60.3	118.6	59.9	115.0	62.8	118.7	60.4	117.9	62.0	118.5	61.4	112.8	64.6	112.9	65.1	111.2	66.6
**C-2′**	70.8	108.0	70.6	108.0	70.0	107.8	70.0	107.8	70.5	108.3	69.7	108.3	69.2	108.0	70.7	106.6	72.9	111.1	68.5	108.1	69.2	107.7	70.1	107.6	70.1
**C-3′**	71.4	104.0	74.4	104.0	73.8	103.6	74.1	103.5	74.7	103.5	74.3	103.7	73.6	104.0	74.5	104.3	75.2	105.0	74.3	104.7	72.4	104.7	73.0	103.9	73.7
**C-4′**	89.2	88.6	89.1	88.6	88.5	86.7	90.4	86.5	90.9	84.1	92.9	84.4	92.1	88.5	89.5	88.6	90.4	88.8	89.9	86.7	89.6	88.7	88.2	83.8	93.2
	**SLOPE**	−1.0497		−1.0467		−1.0384		−1.0405		−1.0454		−1.0433		−1.0377		−1.0348		−1.0433		−1.0434		−1.0481		−1.0306	
	**INTERCEPT**	182.1306		181.2662		180.5262		181.1728		181.1681		180.4667		181.2976		182.0638		182.5589		180.2297		181.1590		179.8380	
	**MSD [ppm^2]**	**1.0**		**2.4**		**2.8**		**3.6**		**3.9**		**4.3**		**5.7**		**6.7**		**7.0**		**9.4**		**9.0**		**9.2**	

δ_{exp} = experimentally obtained ^{13}C chemical shifts, σ_{ISO} = DFT calculated isotropic magnetic shielding, δ_{teor} = theoretical ^{13}C chemical shifts obtained via linear regression approach. SLOPE and INTERCEPT = coefficients of linear regression according to the equation 1. MSD = mean square deviations between δ_{exp} and δ_{teor} corresponding to the conformers **A**–**L** (Figure 9).

Table S20. Experimental and DFT-calculated ^{13}C NMR parameters (δ_{exp}, σ_{ISO} and δ_{theor}) corresponding to conformers **A**–**L** of 6′F-LN **8**.

Conformer		A		B		C		D		E		F		G		H		I		J		K		L	
carbon	δ_{exp}	σ_{ISO}	δ_{teor}	σ_{ISO}	δ_{teor}	σ_{ISO}	δ_{teor}	σ_{ISO}	δ_{teor}	σ_{ISO}	δ_{teor}	σ_{ISO}	δ_{teor}	σ_{ISO}	δ_{teor}	σ_{ISO}	δ_{teor}	σ_{ISO}	δ_{teor}	σ_{ISO}	δ_{teor}	σ_{ISO}	δ_{teor}	σ_{ISO}	δ_{teor}
**C-6′**	83.4	98.3	80.2	98.4	79.6	92.3	84.7	92.2	85.2	93.2	84.3	93.4	83.5	92.2	85.5	91.7	86.9	93.0	85.5	92.1	85.3	98.2	80.2	93.4	84.1
**C-5′**	73.5	107.4	71.6	107.4	70.9	104.6	72.9	104.5	73.5	105.4	72.7	105.3	72.2	104.5	73.6	104.2	74.9	102.9	76.0	104.3	73.5	107.6	71.1	105.9	72.0
**C-1′**	103.7	73.5	103.8	73.4	103.5	74.0	102.2	74.1	102.5	74.2	102.5	73.2	102.9	74.4	102.6	82.8	95.5	77.4	100.5	77.9	99.1	77.3	100.5	77.7	99.4
**C-4**	81.1	96.7	81.7	93.1	84.7	92.8	84.3	96.5	81.2	97.6	80.2	92.8	84.2	95.9	81.9	101.4	77.5	106.8	72.3	95.5	82.1	95.5	82.8	95.8	81.8
**C-3**	72.2	105.2	73.6	104.7	73.5	104.4	73.1	104.6	73.4	104.9	73.1	104.7	72.8	109.7	68.6	108.7	70.5	103.9	75.0	103.9	73.9	104.2	74.3	103.9	73.9
**C-2**	54.5	125.0	54.7	125.2	54.0	125.3	53.1	124.8	54.2	124.5	54.4	125.2	53.1	118.1	60.5	125.3	54.4	125.0	54.7	124.8	53.6	125.0	54.2	124.7	53.7
**CO**	168.8	5.4	168.7	5.4	168.6	5.3	168.0	5.4	168.1	5.3	168.3	5.3	167.9	4.7	169.9	5.3	170.6	5.3	169.7	5.4	169.5	5.3	170.1	5.3	169.7
**MeAc**	23.1	158.9	22.5	158.9	21.7	158.9	21.0	158.9	21.7	158.9	21.6	158.9	20.9	158.9	21.1	158.9	21.9	158.9	22.2	158.9	20.5	158.9	21.4	158.9	20.5
**C-1**	101.7	75.4	102.0	75.6	101.4	75.5	100.7	75.1	101.6	75.0	101.7	75.5	100.7	79.4	97.8	74.9	103.3	75.2	102.6	76.6	100.4	76.6	101.1	76.4	100.6
**OMe**	55.8	124.3	55.5	124.2	54.9	124.2	54.2	124.4	54.6	124.3	54.6	124.2	54.1	124.5	54.3	124.2	55.5	124.3	55.4	124.5	53.9	124.5	54.7	124.5	53.9
**C-5**	75.0	104.4	74.4	103.8	74.5	103.8	73.7	104.6	73.5	104.5	73.6	103.7	73.7	104.4	73.7	106.5	72.7	103.3	75.6	107.0	70.9	107.1	71.6	107.1	70.9
**C-6**	60.3	118.8	60.6	114.7	64.0	114.4	63.6	118.6	60.1	118.6	60.0	114.5	63.4	118.6	60.0	117.9	61.7	118.4	61.1	111.5	66.6	111.5	67.3	111.5	66.6
**C-2′**	70.3	108.2	70.8	108.2	70.2	108.4	69.3	108.4	69.8	108.7	69.6	108.8	68.8	108.4	69.8	106.9	72.2	111.3	67.9	107.6	70.3	107.5	71.2	107.9	70.0
**C-3′**	72.7	102.8	76.0	102.8	75.3	102.9	74.6	102.8	75.2	102.6	75.4	102.8	74.6	102.7	75.3	103.2	75.8	103.8	75.1	102.8	74.9	102.7	75.8	102.7	75.1
**C-4′**	67.9	111.1	68.0	111.2	67.3	109.1	68.6	108.9	69.3	106.1	72.0	106.3	71.3	108.8	69.4	108.7	70.5	108.9	70.2	108.6	69.3	111.1	67.7	106.0	71.8
	**SLOPE**	−1.0496		−1.0455		−1.0450		−1.0486		−1.0461		−1.0445		−1.0365		−1.0332		−1.0410		−1.0304		−1.0323		−1.0294	
	**INTERCEPT**	182.4795		181.5912		180.8225		181.6270		181.4363		180.6853		180.7742		181.5596		182.0085		180.0481		180.9721		180.0030	
	**MSD [ppm²]**	**1.9**		**4.0**		**2.9**		**1.4**		**2.4**		**3.5**		**5.9**		**8.8**		**8.5**		**6.9**		**7.4**		**7.7**	

δ_{exp} = experimentally obtained ^{13}C chemical shifts, σ_{ISO} = DFT calculated isotropic magnetic shielding, δ_{teor} = theoretical ^{13}C chemical shifts obtained via linear regression approach. SLOPE and INTERCEPT = coefficients of linear regression according to the equation 1. MSD = mean square deviations between δ_{exp} and δ_{teor} corresponding to the conformers **A**–**L** (Figure 9).

Table S21. DFT-calculated limiting values of diagnostic $^3J_{H,H}$ couplings [Hz] for individual conformers *gg*/*gt*/*tg* of compound **3**–**8**.

Compound	$^3J_{H5\text{-}H6proR}$			$^3J_{H5\text{-}H6proS}$			$^3J_{H5'\text{-}H6'proR}$			$^3J_{H5'\text{-}H6'proS}$		
	gg	*gt*	*tg*	*gg*	*gt*	*tg*	*gg*	*gt*	*tg*	*gg*	*gt*	*tg*
LN **2**	2.7	9.2	4.8	2.2	1.8	10.0	-[a]	-[a]	-[a]	-[a]	-[a]	-[a]
3F-LN **3**	1.8	9.3	2.9	2.7	2.0	9.2	1.5	9.8	5.3	4.7	2.6	10.7
6F-LN **4**	2.1	9.2	4.9	1.6	1.9	10.5	1.1	9.8	5.2	5.4	2.7	11.0
2′F-LN **5**	2.9	9.3	4.7	2.0	1.8	10.1	1.1	9.8	5.2	5.3	2.7	10.7
3′F-LN **6**	2.8	9.2	4.8	2.1	1.8	10.0	1.2	9.7	5.2	5.2	2.8	10.9
4′F-LN **7**	2.7	9.2	4.8	2.2	1.8	10.0	1.6	9.9	5.5	4.3	2.8	10.7
6′F-LN **8**	2.7	9.1	5.0	2.2	1.8	10.1	0.9	9.6	5.5	3.9	2.3	10.7

[a] Limiting value of coupling was not calculated as the corresponding experimental couplings were not available.

Table S22. DFT calculated limiting values of diagnostic $^3J_{H,H}$ couplings [Hz] for NHAc conformers (Z)-*anti* and (Z)-*syn* of compounds **2**–**8**.[a]

**Compound**	$^3J_{H2\text{-}NH}$	
	(Z)-*anti*	(Z)-*syn*
LN **2**	11.57	8.30
3F-LN **3**	11.70	8.12
6F-LN **4**	11.56	8.32
2′F-LN **5**	11.57	8.31
3′F-LN **6**	11.56	8.29
4′F-LN **7**	11.57	8.30
6′F-LN **8**	11.55	8.29

[a] The values were obtained using implicit C-PCM DMSO solvation.

Analysis of the exocyclic groups conformations using *J*-couplings

The ratios of the staggered conformers (*gg*, *gt*, *tg*) were calculated according to the system of equations S1–S3 using the experimentally determined $^3J_{\mathrm{H6pro}R/\mathrm{H6pro}S\text{-H5}}$ or $^3J_{\mathrm{H6'pro}R/\mathrm{H6'pro}S\text{-H5'}}$ couplings and the corresponding DFT-calculated limiting couplings (Table S21).[1]

$$J_{\exp\_proR} = f_{gg} \cdot J_{calc\_gg\_proR} + f_{gt} \cdot J_{cal\_gt\_proR} + f_{tg} \cdot J_{cal\_tg\_proR} \qquad \text{(S1)}$$

$$J_{\exp\_proS} = f_{gg} \cdot J_{calc\_gg\_proS} + f_{gt} \cdot J_{cal\_gt\_proS} + f_{tg} \cdot J_{cal\_tg\_proS} \qquad \text{(S2)}$$

$$f_{gg} + f_{gt} + f_{tg} = 1 \qquad \text{(S3)}$$

$J_{\exp\_proR}$ = Experimentally determined $^3J_{\mathrm{H6pro}R\text{-H5}}$ or $^3J_{\mathrm{H6'pro}R\text{-H5'}}$.

$J_{\exp\_proS}$= Experimentally determined $^3J_{\mathrm{H6pro}S\text{-H5}}$ or $^3J_{\mathrm{H6'pro}S\text{-H5'}}$.

f_{gg}= Calculated population of *gg* conformer.

f_{gt}= Calculated population of *gt* conformer.

f_{tg}= Calculated population of *tg* conformer.

$J_{calc\_gg\_proR}$= Calculated limiting $^3J_{\mathrm{H6pro}R\text{-H5}}$ or $^3J_{\mathrm{H6'pro}R\text{-H5'}}$ coupling in *gg* conformation.

$J_{cal\_gt\_proR}$ = Calculated limiting $^3J_{\mathrm{H6pro}R\text{-H5}}$ or $^3J_{\mathrm{H6'pro}R\text{-H5'}}$ coupling in *gt* conformation.

$J_{cal\_tg\_proR}$ = Calculated limiting $^3J_{\mathrm{H6pro}R\text{-H5}}$ or $^3J_{\mathrm{H6'pro}R\text{-H5'}}$ coupling in *tg* conformation.

$J_{calc\_gg\_proS}$= Calculated limiting $^3J_{\mathrm{H6pro}S\text{-H5}}$ or $^3J_{\mathrm{H6'pro}S\text{-H5'}}$ coupling in *gg* conformation.

$J_{cal\_gt\_proS}$= Calculated limiting $^3J_{\mathrm{H6pro}S\text{-H5}}$ or $^3J_{\mathrm{H6'pro}S\text{-H5'}}$ coupling in *gt* conformation.

$J_{cal\_tg\_proS}$ = Calculated limiting $^3J_{\mathrm{H6pro}S\text{-H5}}$ or $^3J_{\mathrm{H6'pro}S\text{-H5'}}$ coupling in *tg* conformation.

Assignment of $\mathrm{H6}_{\mathrm{pro}R}$ and $\mathrm{H6}_{\mathrm{pro}S}$ protons (GlcNAc rings)

The $\mathrm{H6}_{\mathrm{pro}R}$ and $\mathrm{H6}_{\mathrm{pro}S}$ protons were assigned taking into account the ratios of the conformers *gg*, *gt*, and *tg*, which were calculated for both assignment possibilities (Table S23). In the first possibility, the proton that showed a higher coupling with H5 was assigned as $\mathrm{H6}_{\mathrm{pro}R}$, while the remaining proton was assigned as $\mathrm{H6}_{\mathrm{pro}S}$. This resulted in the expected preference of the *gg* conformation, with a minor population of the *gt* conformation and only a negligible population

of the *tg* conformation. The second (reversed) assignment possibility also showed a preference for the *gg* conformation, but with relatively high populations of *tg* conformers and very low or negative populations of *gt* conformers. As the population of *tg* conformers is generally considered negligible in gluco-configured systems due to the unfavorable steric 1,3-interaction (syn-pentane-type) between the hydroxyl groups O6H and O4H and the absence of a stabilizing stereoelectronic gauche effect, assignment possibility 1 was considered proper for all compounds **2**–**8**. This $H6_{proR}$ and $H6_{proS}$ assignment is in accordance with our ^{13}C chemical shift-based conformational analysis.

Table S23. Experimental $^3J_{H6proR/H6proS\text{-}H5}$ couplings [Hz] and calculated distribution *f* [%] of side chain conformers *gg*/*gt*/*tg* corresponding to the two possible assignments of $H6_{proR}$ and $H6_{proS}$ protons.[a]

Compound	Assignment possibility 1					Assignment possibility 2				
	$^3J_{H5\text{-}H6proR}$	$^3J_{H5\text{-}H6proS}$	f_{gg}	f_{gt}	f_{tg}	$^3J_{H5\text{-}H6proR}$	$^3J_{H5\text{-}H6proS}$	f_{gg}	f_{gt}	f_{tg}
LN **2**	**4.7**	**2.2**	**65**	**30**	**5**	2.2	4.7	83	-15	32
3F-LN **3**	**4.0**	**2.4**	**71**	**29**	**0**	2.4	4.0	74	5	21
6F-LN **4**	**4.6**	**1.8**	**64**	**35**	**1**	1.8	4.6	83	-18	35
2′F-LN **5**	**5.1**	**1.9**	**65**	**35**	**0**	1.9	5.1	89	-26	37
3′F-LN **6**	**5.0**	**2.3**	**63**	**34**	**4**	2.3	5.0	82	-18	36
4′F-LN **7**	**4.8**	**2.4**	**65**	**30**	**5**	2.4	4.8	83	-15	32
6′F-LN **8**	**4.8**	**2.2**	**66**	**32**	**1**	2.2	4.8	77	-3	26

[a] Green color represents the proper assignment $^3J_{H6proR\text{-}H5}$ and $^3J_{H6proS\text{-}H5}$ couplings.

Assignment of $H6'_{proR}$ and $H6'_{proS}$ protons (Gal rings)

The assignment of $H6'_{proR}$ and $H6'_{proS}$ protons was carried out by calculating the ratios of conformers *gg*, *gt*, and *tg*, followed by analyzing the ROESY spectra and comparing them with previously reported assignments.[2] This was done considering two different assignment possibilities, similarly as in the previous case of GlcNAc units. In the first possibility, proton that displayed higher coupling with H5′ was assigned as $H6'_{proR}$, while the remaining proton was assigned as $H6'_{proS}$. Such assignment favored *gt* conformers with minor populations of *gg* and *tg* conformers. In contrast, the second (reversed) assignment possibility showed a preference for the *tg* conformation accompanied by minor *gg* and *gt* conformers (Table S24). From such a simple comparison, the evaluation of proper assignment was possible only for 6′F-LN **8**. This compound showed the highest difference between the $^3J_{H6proR\text{-}H5}$ and $^3J_{H6proS\text{-}H5}$ couplings, leading to the significant preference of the *gt* conformer in the assignment possibility

1. The second assignment possibility resulted in a high population of both *gg* and *tg* conformers, accompanied by a negative population of *gt* conformer. As previously reported, fluorine at the 6′-position of galacto-configured carbohydrates strengthens the gauche effect with pyranose oxygen leading to an increase in the *gt* population and suppression of the *tg* population, consistent with the assignment possibility 1 and our ^{13}C chemical shift-based conformational analysis.[1]

Table S24. Experimental $^{3}J_{H6'proR/H6'proS\text{-}H5}$ couplings [Hz] and calculated distribution *f* [%] of side chain conformers *gg*/*gt*/*tg* corresponding to the two possible assignments of protons H6′$_{proR}$ and H6′$_{proS}$. [a]

Compound	Assignment possibility 1					Assignment possibility 2				
	$^{3}J_{H5'\text{-}H6'proR}$	$^{3}J_{H5'\text{-}H6'proS}$	f_{gg}	f_{gt}	f_{tg}	$^{3}J_{H5'\text{-}H6'proR}$	$^{3}J_{H5'\text{-}H6'proS}$	f_{gg}	f_{gt}	f_{tg}
LN **2**	-[b]	-[b]	-[b]	-[b]	-[b]	-[b]	-[b]	-[b]	-[b]	-[b]
3F-LN **3**	7.1	5.9	12	50	38	**5.9**	**7.1**	**21**	**29**	**50**
6F-LN **4**	**6.7**	**5.2**	**23**	**54**	**23**	5.2	6.7	32	30	38
2′F-LN **5**	**6.8**	**5.4**	**20**	**53**	**27**	5.4	6.8	28	30	42
3′F-LN **6**	**7.3**	**5.7**	**11**	**56**	**33**	5.7	7.3	21	30	50
4′F-LN **7**	**7.3**	**5.9**	**12**	**51**	**37**	5.9	7.3	20	27	53
6′F-LN **8**	**7.9**	**3.2**	**17**	**76**	**7**	3.2	7.9	47	-5	58

[a] Green color represents the proper assignment $^{3}J_{H6proR\text{-}H5}$ and $^{3}J_{H6proS\text{-}H5}$ couplings; [b] coupling could not be determined due to spectral overlap.

Compounds **3** and **7** showed the separation of H6′$_{proR}$ and H6′$_{proS}$ signals in the ^{1}H NMR spectra allowing us to analyze their spatial ROE contacts with H4′. To interpret ROESY spectra, we calculated H6′$_{proR}$-H4′ and H6′$_{proS}$-H4′ distances from DFT-optimized geometries of individual possible conformers (*gg*/*gt*/*tg*) at 6′ exocyclic group (Table S25).

Table S25. H6′$_{proR}$-H4′ and H6′$_{proS}$-H4′ distances from DFT-optimized geometries of possible 6′-conformers (*gg*/*gt*/*tg*) in compounds **3** and **7**.

	**6′-*gg***		**6′-*gt***		**6′-*tg***	
**Distances** [Å]	H6′$_{proR}$-H4′	H6′$_{proS}$-H4′	H6′$_{proR}$-H4′	H6′$_{proS}$-H4′	H6′$_{proR}$-H4′	H6′$_{proS}$-H4′
3F-LN **3**	2.60	3.76	3.31	2.66	3.82	3.21
4′F-LN **7**	2.62	3.80	3.25	2.66	3.82	3.17

These H6′$_{proR}$-H4′ and H6′$_{proS}$-H4′ distances corresponding to the individual 6′-conformers (Table S25) were subsequently weight-averaged on the basis of *gg*/*gt*/*tg* conformer populations

(Table S26). This provided two sets of population-averaged H6′$_{proR}$-H4′ and H6′$_{proS}$-H4′ distances corresponding to both H6′ proton assignment possibilities 1 and 2, for each compound **3** and **7** (Table S26, columns 3 and 4, eight distances in total). The ratio of population-averaged H6′$_{proR}$-H4′/H6′$_{proS}$-H4′ distances was subsequently used for the estimation of relative H6′$_{proS}$-H4′/H6′$_{proR}$-H4′ ROESY correlations intensity (Table S26, columns 5 and 6).

Table S26. Population averaged H6′$_{proR}$-H4′ and H6′$_{proS}$-H4′ distances from DFT-optimized 6′-*gg*/*gt*/*tg* conformers and expected ratios of H6′$_{proS}$-H4′ / H6′$_{proR}$-H4′ ROE intensities.

	***gg*/*gt*/*tg* Population**	**Population averaged H6′$_{proR}$-H4′ distance**	**Population averaged H6′$_{proS}$-H4′ distance**	**Ratio of Population averaged distances H6′$_{proR}$-H4′ / H6′$_{proS}$-H4′**	**Relative H6′$_{proS}$-H4′ / H6′$_{proR}$-H4′ ROE intensities[a]**
3F-LN **3** assignment possibility 1	12/50/38	3.42 Å	3.00 Å	1.14	2.18
3F-LN **3** assignment possibility 2	21/29/50	3.41 Å	3.17 Å	1.08	1.57
4′F-LN **7** assignment possibility 1	12/51/37	3.39 Å	2.99 Å	1.13	2.13
4′F-LN **7** assignment possibility 2	20/27/53	3.43 Å	3.16 Å	1.08	1.63

[a] The relative H6′$_{proS}$-H4′ / H6′$_{proR}$-H4′ ROE intensities were estimated as sixth power of ratio of population averaged distances H6′$_{proR}$-H4′ / H6′$_{proS}$-H4′.

Both compounds **3** and **7** displayed lower population-averaged H6′$_{proS}$-H4′ distance than H6′$_{proR}$-H4′ distance regardless of *gg*/*gt*/*tg* population used in the averaging methodology (Table S26). Accordingly, proton H6′$_{proS}$ always showed a stronger theoretically calculated NOE/ROE correlation with the proton H4′ than the corresponding proton H6′$_{proR}$, enabling the interpretation of ROESY spectra of compounds **3** and **7**. In compound **3**, the more downfield-shifted H6′ proton displayed a stronger ROESY correlation with H4′ than the more upfield-shifted H6′ proton (Figure S16). Therefore, we assigned the more downfield-shifted H6′ proton as H6′$_{proS}$ and the more upfield-shifted proton as H6′$_{proR}$. Consequently, compound **3** displayed a larger $J_{H6'proS\text{-}H5'}$ coupling (7.1 Hz) than $J_{H6'proR\text{-}H5'}$ coupling (5.9 Hz), indicating the preference for 6′-*tg* conformer in DMSO-d_6 solution. In compound **7**, the more downfield-shifted H6′

proton also displayed a stronger ROESY correlation with H4′ than the more upfield-shifted H6′ proton (Figure S17). Therefore, we assigned the more downfield-shifted H6′ proton as H6′$_{\mathrm{pro}S}$ and the more upfield-shifted proton as H6′$_{\mathrm{pro}R}$. In this case, compound **7** showed higher $J_{\mathrm{H6'pro}R\text{-}\mathrm{H5'}}$ coupling (7.3 Hz) than $J_{\mathrm{H6'pro}S\text{-}\mathrm{H5'}}$ coupling (5.9 Hz), indicating the slight preference of 6′-*gt* conformer over 6′-*tg* in DMSO-d_6 solution. To exclude the influence of offset dependence of ROESY cross peak intensities, we also investigated both compounds using NOESY, which provided analogical results as ROESY. The H6′$_{\mathrm{pro}R}$ and H6′$_{\mathrm{pro}S}$ protons of the remaining compounds **4**, **5**, and **6** were tentatively assigned according to the previously reported assignment of lactose analogues, consistent with the assignment possibility 1 (Table S24), indicating the slight preference of 6′-*gt* conformer over 6′-*tg* in DMSO-d_6 solution.[2]

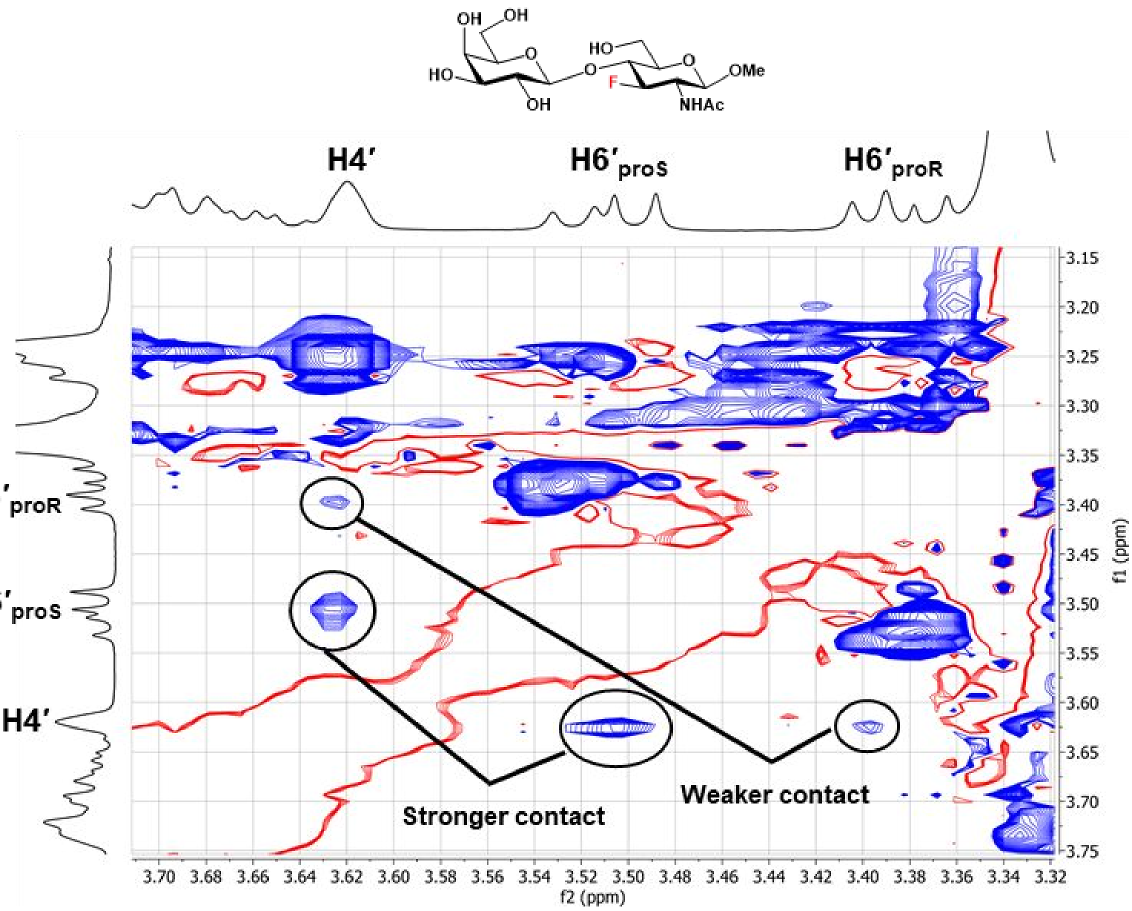

Figure S16. ^{1}H-^{1}H ROESY spectrum of 3F-LN **3** in DMSO-d_6, showing correlations of protons H6′$_{\mathrm{pro}S}$ and H6′$_{\mathrm{pro}R}$ with proton H4′. 1D selective homonuclear O6′-H decoupled spectrum was used as an external projection of both x- and y-axes.

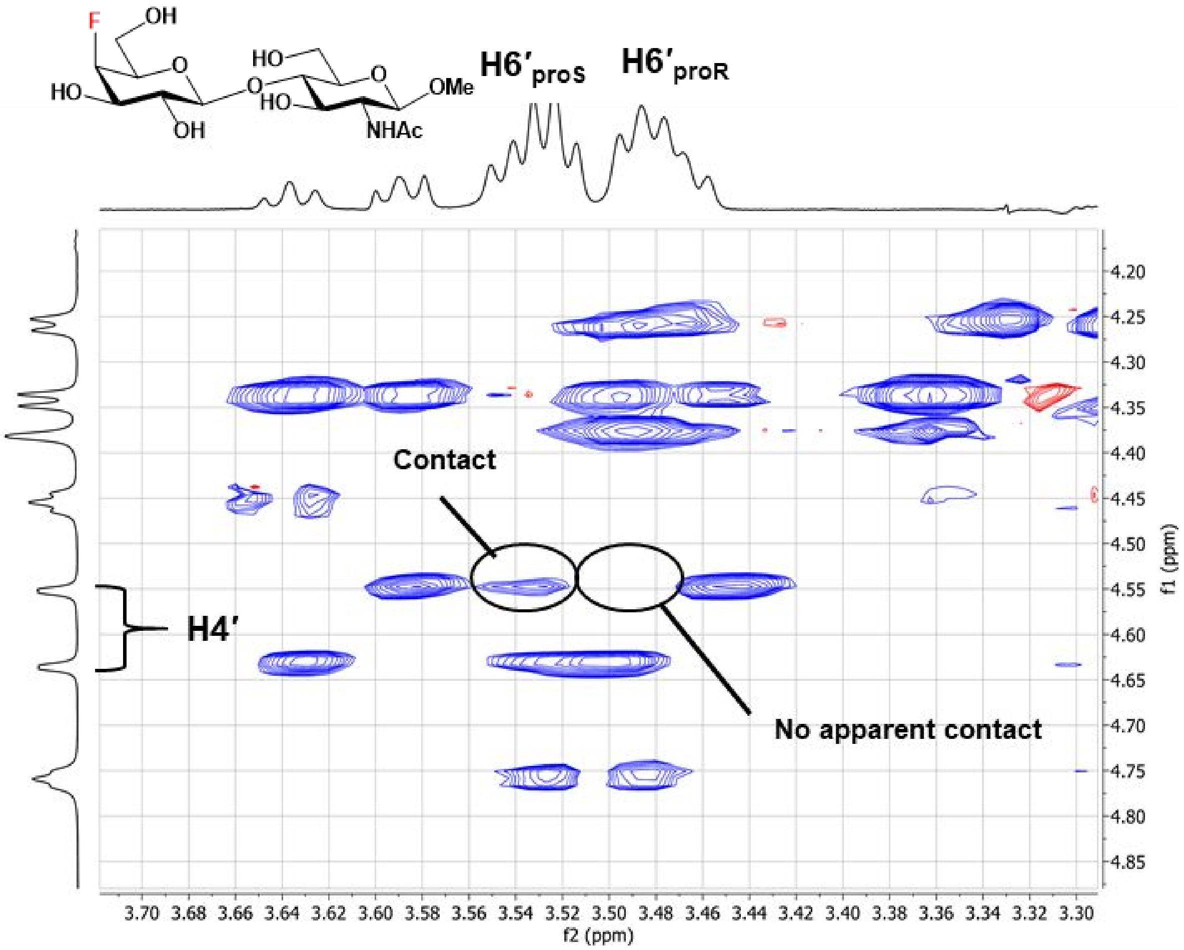

Figure S17. ^{1}H-^{1}H ROESY spectrum of 4′F-LN **7** in DMSO-d_6 at 60 °C, showing correlations of protons H6′$_{proS}$ with proton H4′. 1D selective homonuclear O6′-H TOCSY spectrum was used as an external projection of the x-axis.

Analysis of the acetamido group conformations using *J*-couplings

The conformational behavior of an acetamido group was investigated by DFT in the presence of an explicit DMSO solution using a simplified system—methyl *N*-acetyl-β-D-glucosaminide (Figure S18). The explicit DMSO solvation altered the acetamide geometry, which influenced the $J_{H2\text{-}NH}$ couplings. Mododentate solvation with $(CH_3)S{=}O\cdots H\text{-}N$ hydrogen bond (Figure S18B) decreased the observed $J_{H2\text{-}NH}$ coupling constant to 10.51 Hz. Bidentate solvation with two hydrogen bonds: $(CH_3)S{=}O\cdots H\text{-}N$ and $(CH_3)S{=}O\cdots H\text{-}O3$ (Figure S18C) decreased the $J_{H2\text{-}NH}$ to the 8.32 Hz.

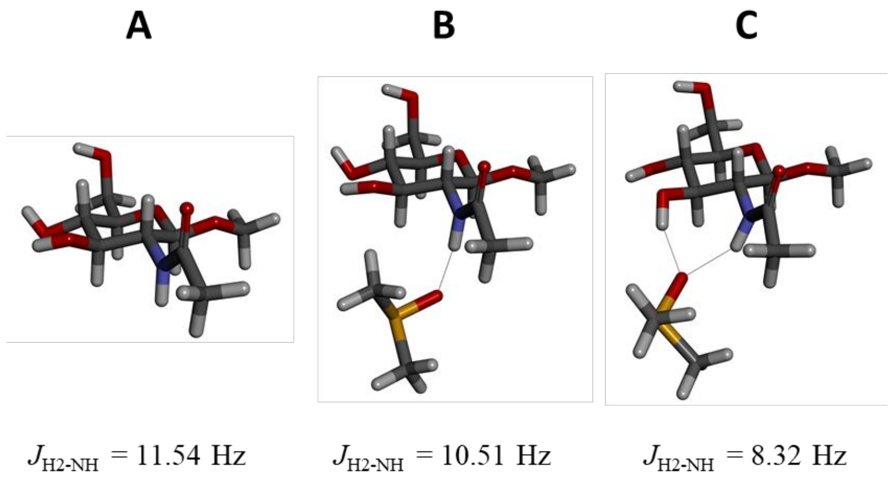

Figure S18. DFT-optimized geometries of methyl *N*-acetyl-β-D-glucosaminide. A) C-PCM implicit DMSO solvation; B) C-PCM implicit DMSO solvation + one explicit DMSO molecule in monodentate solvation. C) C-PCM implicit DMSO solvation + one explicit DMSO molecule in bidentate solvation.

References

(1) Bock, K.; Duus, J. Ø. A Conformational Study of Hydroxymethyl Groups in Carbohydrates Investigated by ^{1}H NMR Spectroscopy. *J. Carbohydr. Chem.* **1994**, *13* (4), 513–543. DOI: 10.1080/07328309408011662

(2) Fernández, P.; Jiménez-Barbero, J. The Conformation of Some Halodeoxy Analogues of Methyl β-Lactoside in D_2O and DMSO-D_6 Solutions. *J. Carbohydr. Chem.* **1994**, *13* (2), 207–233. DOI: 10.1080/07328309408009189
