# Supplementary material for: Influence of Selective Deoxyfluorination on the Molecular Structure of Type-2 N-Acetyllactosamine
Source: J Org Chem. 2024 Aug 23;89(17):11875–90. doi: 10.1021/acs.joc.4c00879 (PMC11382267; doi:10.1021/acs.joc.4c00879)
Supplement: Supplementary file 2 — jo4c00879_si_002.docx [file jo4c00879_si_002.docx]

**Supporting Information II**

**The Influence of Selective Deoxyfluorination on the Molecular Structure of Type-2 *N*-Acetyllactosamine**

Martin Kurfiřt,*^a^*^,^*^e^*^*^ Lucie Červenková Šťastná,*^a^* Martin Dračínský,*^b^* Radek Pohl,*^b^* Ivana Císařová,*^c^* Jan Sýkora,*^f^* Martin Balouch,*^g^* Michal Baka,*^d,h^* Vojtěch Hamala,*^a^*^,^*^e^* F. Javier Cañada,*^i,m^* Ana Ardá,*^j,k^* Jesús Jiménez-Barbero, *^j,k,l,m^* Jindřich Karban*^a^*

*^a^* Institute of Chemical Process Fundamentals, Czech Academy of Sciences, Rozvojová 1/135, CZ-165 00 Praha 6, Czech Republic.

*^b^* Institute of Organic Chemistry and Biochemistry, Czech Academy of Sciences, Flemingovo náměstí 542/2, CZ-160 00 Praha 6, Czech Republic

*^c^* Department of Inorganic Chemistry, Faculty of Science, Charles University in Prague, Hlavova 8, CZ-128 43 Praha 2, Czech Republic

*^d^* Institute of Entomology, Biology Centre of the Czech Academy of Sciences, Branišovská 31, 370 05 České Budějovice, Czech Republic

*^e^* Department of Organic Chemistry. *^f^* Department of Analytical Chemistry. *^g^* Department of Chemical Engineering, *^h^* Department of Food Analysis and Nutrition, University of Chemistry and Technology, Prague, Technická 5, 166 28 Prague 6, Czech Republic

*^i^* Centro de Investigaciones Biológicas Margarita Salas, Ramiro de Maeztu 9, 28040 Madrid, Spain

*^j^* CICbioGUNE, Basque Research & Technology Alliance (BRTA), Bizkaia Technology Park, Building 800, 48162 Derio Bizkaia, Spain

*^k^* Ikerbasque, Basque Foundation for Science, Plaza Euskadi 2, 48013 Bilbao Bizkaia, Spain

*^l^* Department of Organic and Inorganic Chemistry, Faculty of Science and Technology, University of the Basque Country, EHU-UPV, 48940 Leioa, Spain

*^m^* CIBER de Enfermedades Respiratorias (CIBERES), Avda Monforte de Lemos 3-5, 28029 Madrid, Spain

*Corresponding author. E-mail address: [kurfirt@icpf.cas.cz](mailto:kurfirt@icpf.cas.cz)

# Table of Contents

[Table of Contents S2](#_Toc167438400)

[Copies of NMR spectra S8](#_Toc167438401)

[^1^H NMR (400 MHz, DMSO-*d*_6_) LN **2** S8](#_Toc167438402)

[^13^C{^1^H} NMR (101 MHz, DMSO-*d*_6_) LN **2** S8](#_Toc167438403)

[^1^H-^1^H COSY NMR (DMSO-*d*_6_) LN **2** S9](#_Toc167438404)

[^1^H-^13^C HSQC NMR (DMSO-*d*_6_) LN **2** S9](#_Toc167438405)

[^1^H-^13^C HMBC NMR (DMSO-*d*_6_) LN **2** S10](#_Toc167438406)

[^1^H-^1^H ROESY NMR (DMSO-*d*_6_) LN **2** S10](#_Toc167438407)

[^1^H-^1^H ROESY NMR (DMSO-*d*_6_) LN **2** S11](#_Toc167438408)

[^1^H-^1^H ROESY NMR (DMSO-*d*_6_) LN **2** S11](#_Toc167438409)

[Selective Homonuclear Decoupled ^1^H-NMR (DMSO-*d*_6_) LN **2** S12](#_Toc167438410)

[Temperature Dependent ^1^H-NMR (DMSO-*d*_6_) LN **2** S12](#_Toc167438411)

[^1^H NMR (400 MHz, DMSO-*d*_6_) 3F-LN **3** S13](#_Toc167438412)

[^13^C{^1^H} APT NMR (126 MHz, DMSO-*d*_6_) 3F-LN **3** S13](#_Toc167438413)

[^19^F NMR (376 MHz, DMSO-*d*_6_) 3F-LN **3** S14](#_Toc167438414)

[^1^H-^1^H COSY NMR (DMSO-*d*_6_) 3F-LN **3** S14](#_Toc167438415)

[^1^H-^13^C HSQC NMR (DMSO-*d*_6_) 3F-LN **3** S15](#_Toc167438416)

[^1^H-^13^C HMBC NMR (DMSO-*d*_6_) 3F-LN **3** S15](#_Toc167438417)

[^1^H-^1^H ROESY NMR (DMSO-*d*_6_) 3F-LN **3** S16](#_Toc167438418)

[^1^H-^1^H ROESY NMR (DMSO-*d*_6_) 3F-LN **3** S16](#_Toc167438419)

[^1^H-^1^H ROESY NMR (DMSO-*d*_6_) 3F-LN **3** S17](#_Toc167438420)

[^1^H-^1^H ROESY NMR (DMSO-*d*_6_) 3F-LN **3** S17](#_Toc167438421)

[^1^H-^1^H ROESY NMR (DMSO-*d*_6_) 3F-LN **3** S18](#_Toc167438422)

[Selective Homonuclear Decoupled ^1^H-NMR (DMSO-*d*_6_) 3F-LN **3** S18](#_Toc167438423)

[1D Selective Gradient ^1^H-^1^H TOCSY NMR (DMSO-*d*_6_) 3F-LN **3** S19](#_Toc167438424)

[Temperature Dependent ^1^H-NMR (DMSO-*d*_6_) 3F-LN **3** S19](#_Toc167438425)

[^1^H NMR (400 MHz, DMSO-*d*_6_) 6F-LN **4** S20](#_Toc167438426)

[^13^C{^1^H} APT NMR (126 MHz, DMSO-*d*_6_) 6F-LN **4** S20](#_Toc167438427)

[^19^F NMR (376 MHz, DMSO-*d*_6_) 6F-LN **4** S21](#_Toc167438428)

[^1^H-^1^H COSY NMR (DMSO-*d*_6_) 6F-LN **4** S21](#_Toc167438429)

[^1^H-^13^C HSQC NMR (DMSO-*d*_6_) 6F-LN **4** S22](#_Toc167438430)

[^1^H-^13^C HMBC NMR (DMSO-*d*_6_) 6F-LN **4** S22](#_Toc167438431)

[^1^H-^1^H ROESY NMR (DMSO-*d*_6_) 6F-LN **4** S23](#_Toc167438432)

[^1^H-^1^H ROESY NMR (DMSO-*d*_6_) 6F-LN **4** S23](#_Toc167438433)

[^1^H-^1^H ROESY NMR (DMSO-*d*_6_) 6F-LN **4** S24](#_Toc167438434)

[^1^H-^1^H ROESY NMR (DMSO-*d*_6_) 6F-LN **4** S24](#_Toc167438435)

[^1^H-^1^H ROESY NMR (DMSO-*d*_6_) 6F-LN **4** S25](#_Toc167438436)

[^1^H NMR (400 MHz, DMSO-*d*_6_) 6F-LN **4** S25](#_Toc167438437)

[1D Selective Gradient ^1^H-^1^H ROESY NMR (DMSO-*d*_6_) 6F-LN **4** S26](#_Toc167438438)

[Temperature Dependent ^1^H-NMR (DMSO-*d*_6_) 6F-LN **4** S26](#_Toc167438439)

[^1^H NMR (400 MHz, DMSO-*d*_6_) 2′F-LN **5** S27](#_Toc167438440)

[^13^C{^1^H} NMR (101 MHz, DMSO-*d*_6_) 2′F-LN **5** S27](#_Toc167438441)

[^19^F NMR (376 MHz, DMSO-*d*_6_) 2′F-LN **5** S28](#_Toc167438442)

[^1^H-^1^H COSY NMR (DMSO-*d*_6_) 2′F-LN **5** S28](#_Toc167438443)

[^1^H-^13^C HSQC NMR (DMSO-*d*_6_) 2′F-LN **5** S29](#_Toc167438444)

[^1^H-^13^C HMBC NMR (DMSO-*d*_6_) 2′F-LN **5** S29](#_Toc167438445)

[^1^H-^1^H ROESY NMR (DMSO-*d*_6_) 2′F-LN **5** S30](#_Toc167438446)

[^1^H-^1^H ROESY NMR (DMSO-*d*_6_) 2′F-LN **5** S30](#_Toc167438447)

[^1^H-^1^H ROESY NMR (DMSO-*d*_6_) 2′F-LN **5** S31](#_Toc167438448)

[^1^H-^1^H ROESY NMR (DMSO-*d*_6_) 2′F-LN **5** S31](#_Toc167438449)

[^1^H-^1^H ROESY NMR (DMSO-*d*_6_) 2′F-LN **5** S32](#_Toc167438450)

[^1^H NMR (DMSO-*d*_6_) 2′F-LN **5** S32](#_Toc167438451)

[^1^H NMR (DMSO-*d*_6_) 2′F-LN **5** S33](#_Toc167438452)

[Selective Gradient ^1^H-^1^H ROESY NMR (DMSO-*d*_6_) 2′F-LN **5** S33](#_Toc167438453)

[Temperature Dependent ^1^H-NMR (DMSO-*d*_6_) 2′F-LN **5** S34](#_Toc167438454)

[^1^H NMR (400 MHz, DMSO-*d*_6_) 3′F-LN **6** S34](#_Toc167438455)

[^13^C{^1^H} APT NMR (126 MHz, DMSO-*d*_6_) 3′F-LN **6** S35](#_Toc167438456)

[^19^F NMR (376 MHz, DMSO-*d*_6_) 3′F-LN **6** S35](#_Toc167438457)

[^1^H-^1^H COSY NMR (DMSO-*d*_6_) 3′F-LN **6** S36](#_Toc167438458)

[^1^H-^13^C HSQC NMR (DMSO-*d*_6_) 3′F-LN **6** S36](#_Toc167438459)

[^1^H-^13^C HMBC NMR (DMSO-*d*_6_) 3′F-LN **6** S37](#_Toc167438460)

[^1^H-^1^H ROESY NMR (DMSO-*d*_6_) 3′F-LN **6** S37](#_Toc167438461)

[^1^H-^1^H ROESY NMR (DMSO-*d*_6_) 3′F-LN **6** S38](#_Toc167438462)

[^1^H-^1^H ROESY NMR (DMSO-*d*_6_) 3′F-LN **6** S38](#_Toc167438463)

[^1^H-^1^H ROESY NMR (DMSO-*d*_6_) 3′F-LN **6** S39](#_Toc167438464)

[Selective Homonuclear Decoupled ^1^H-NMR (DMSO-*d*_6_) 3′F-LN **6** S39](#_Toc167438465)

[1D Selective Gradient ^1^H-^1^H TOCSY NMR (DMSO-*d*_6_) 3′F-LN **6** S40](#_Toc167438466)

[Temperature Dependent ^1^H-NMR (DMSO-*d*_6_) 3′F-LN **6** S41](#_Toc167438467)

[^1^H NMR (500 MHz, DMSO-*d*_6_) 4′F-LN **7** S41](#_Toc167438468)

[^13^C{^1^H} APT NMR (126 MHz, DMSO-*d*_6_) 4′F-LN **7** S42](#_Toc167438469)

[^19^F NMR (376 MHz, DMSO-*d*_6_) 4′F-LN **7** S42](#_Toc167438470)

[^1^H-^1^H COSY NMR (DMSO-*d*_6_) 4′F-LN **7** S43](#_Toc167438471)

[^1^H-^13^C HSQC NMR (DMSO-*d*_6_) 4′F-LN **7** S43](#_Toc167438472)

[^1^H-^13^C HMBC NMR (DMSO-*d*_6_) 4′F-LN **7** S44](#_Toc167438473)

[^1^H-^1^H ROESY NMR (DMSO-*d*_6_) 4′F-LN **7** S44](#_Toc167438474)

[^1^H-^1^H ROESY NMR (DMSO-*d*_6_) 4′F-LN **7** S45](#_Toc167438475)

[^1^H-^1^H ROESY NMR (DMSO-*d*_6_) 4′F-LN **7** S45](#_Toc167438476)

[^1^H-^1^H ROESY NMR (DMSO-*d*_6_) 4′F-LN **7** S46](#_Toc167438477)

[^1^H-^1^H ROESY NMR (DMSO-*d*_6_, 60 °C) 4′F-LN **7** S46](#_Toc167438478)

[Selective Homonuclear Decoupled ^1^H-NMR (DMSO-*d*_6_) 4′F-LN **7** S47](#_Toc167438479)

[1D Selective Gradient ^1^H-^1^H TOCSY NMR (DMSO-*d*_6_) 4′F-LN **7** S47](#_Toc167438480)

[Temperature Dependent ^1^H-NMR (DMSO-*d*_6_) 4′F-LN **7** S48](#_Toc167438481)

[^1^H NMR (400 MHz, DMSO-*d*_6_) 6′F-LN **8** S48](#_Toc167438482)

[^13^C{^1^H} NMR (101 MHz, DMSO-*d*_6_) 6′F-LN **8** S49](#_Toc167438483)

[^19^F NMR (376 MHz, DMSO-*d*_6_) 6′F-LN **8** S49](#_Toc167438484)

[^1^H-^1^H COSY NMR (DMSO-*d*_6_) 6′F-LN **8** S50](#_Toc167438485)

[^1^H-^13^C HSQC NMR (DMSO-*d*_6_) 6′F-LN **8** S50](#_Toc167438486)

[^1^H-^13^C HMBC NMR (DMSO-*d*_6_) 6′F-LN **8** S51](#_Toc167438487)

[^1^H-^1^H ROESY NMR (DMSO-*d*_6_) 6′F-LN **8** S51](#_Toc167438488)

[^1^H-^1^H ROESY NMR (DMSO-*d*_6_) 6′F-LN **8** S52](#_Toc167438489)

[^1^H-^1^H ROESY NMR (DMSO-*d*_6_) 6′F-LN **8** S52](#_Toc167438490)

[^1^H-^1^H ROESY NMR (DMSO-*d*_6_) 6′F-LN **8** S53](#_Toc167438491)

[^1^H-^1^H ROESY NMR (DMSO-*d*_6_) 6′F-LN **8** S53](#_Toc167438492)

[^1^H NMR (400 MHz, DMSO-*d*_6_) 6′F-LN **8** S54](#_Toc167438493)

[^1^H NMR (400 MHz, DMSO-*d*_6_) 6′F-LN **8** S54](#_Toc167438494)

[^1^H NMR (400 MHz, DMSO-*d*_6_) 6′F-LN **8** S55](#_Toc167438495)

[^1^H NMR (400 MHz, DMSO-*d*_6_) 6′F-LN **8** S55](#_Toc167438496)

[^1^H NMR (400 MHz, DMSO-*d*_6_, 31 °C) 6′F-LN **8** S56](#_Toc167438497)

[Temperature Dependent ^1^H-NMR (DMSO-*d*_6_) 6′F-LN **8** S56](#_Toc167438498)

[Cartesian coordinates (Å), computed total energy values E(B3LYP), computed sum of electronic and thermal free energies (G) and number of imaginary frequencies of optimized geometries S57](#_Toc167438499)

[LN **2** Conformer A S58](#_Toc167438500)

[LN **2** Conformer B S59](#_Toc167438501)

[LN **2** Conformer C S60](#_Toc167438502)

[LN **2** Conformer D S61](#_Toc167438503)

[LN **2** Conformer E S62](#_Toc167438504)

[LN **2** Conformer F S63](#_Toc167438505)

[LN **2** Conformer G S64](#_Toc167438506)

[LN **2** Conformer H S65](#_Toc167438507)

[LN **2** Conformer I S66](#_Toc167438508)

[LN **2** Conformer J S67](#_Toc167438509)

[LN **2** Conformer K S68](#_Toc167438510)

[LN **2** Conformer L S69](#_Toc167438511)

[3F-LN **3** Conformer A S70](#_Toc167438512)

[3F-LN **3** Conformer B S71](#_Toc167438513)

[3F-LN **3** Conformer C S72](#_Toc167438514)

[3F-LN **3** Conformer D S73](#_Toc167438515)

[3F-LN **3** Conformer E S74](#_Toc167438516)

[3F-LN **3** Conformer F S75](#_Toc167438517)

[3F-LN **3** Conformer G S76](#_Toc167438518)

[3F-LN **3** Conformer H S77](#_Toc167438519)

[3F-LN **3** Conformer I S78](#_Toc167438520)

[3F-LN **3** Conformer J S79](#_Toc167438521)

[3F-LN **3** Conformer K S80](#_Toc167438522)

[3F-LN **3** Conformer L S81](#_Toc167438523)

[6F-LN **4** Conformer A S82](#_Toc167438524)

[6F-LN **4** Conformer B S83](#_Toc167438525)

[6F-LN **4** Conformer C S84](#_Toc167438526)

[6F-LN **4** Conformer D S85](#_Toc167438527)

[6F-LN **4** Conformer E S86](#_Toc167438528)

[6F-LN **4** Conformer F S87](#_Toc167438529)

[6F-LN **4** Conformer G S88](#_Toc167438530)

[6F-LN **4** Conformer H S89](#_Toc167438531)

[6F-LN **4** Conformer I S90](#_Toc167438532)

[6F-LN **4** Conformer J S91](#_Toc167438533)

[6F-LN **4** Conformer K S92](#_Toc167438534)

[6F-LN **4** Conformer L S93](#_Toc167438535)

[2′F-LN **5** Conformer A S94](#_Toc167438536)

[2′F-LN **5** Conformer B S95](#_Toc167438537)

[2′F-LN **5** Conformer C S96](#_Toc167438538)

[2′F-LN **5** Conformer D S97](#_Toc167438539)

[2′F-LN **5** Conformer E S98](#_Toc167438540)

[2′F-LN **5** Conformer F S99](#_Toc167438541)

[2′F-LN **5** Conformer G S100](#_Toc167438542)

[2′F-LN **5** Conformer H S101](#_Toc167438543)

[2′F-LN **5** Conformer I S102](#_Toc167438544)

[2′F-LN **5** Conformer J S103](#_Toc167438545)

[2′F-LN **5** Conformer K S104](#_Toc167438546)

[2′F-LN **5** Conformer L S105](#_Toc167438547)

[3′F-LN **6** Conformer A S106](#_Toc167438548)

[3′F-LN **6** Conformer B S107](#_Toc167438549)

[3′F-LN **6** Conformer C S108](#_Toc167438550)

[3′F-LN **6** Conformer D S109](#_Toc167438551)

[3′F-LN **6** Conformer E S110](#_Toc167438552)

[3′F-LN **6** Conformer F S111](#_Toc167438553)

[3′F-LN **6** Conformer G S112](#_Toc167438554)

[3′F-LN **6** Conformer H S113](#_Toc167438555)

[3′F-LN **6** Conformer I S114](#_Toc167438556)

[3′F-LN **6** Conformer J S115](#_Toc167438557)

[3′F-LN **6** Conformer K S116](#_Toc167438558)

[3′F-LN **6** Conformer L S117](#_Toc167438559)

[3′F-LN **6** O2′-H…O6 Hydrogen Bond S118](#_Toc167438560)

[4′F-LN **7** Conformer A S119](#_Toc167438561)

[4′F-LN **7** Conformer B S120](#_Toc167438562)

[4′F-LN **7** Conformer C S121](#_Toc167438563)

[4′F-LN **7** Conformer D S122](#_Toc167438564)

[4′F-LN **7** Conformer E S123](#_Toc167438565)

[4′F-LN **7** Conformer F S124](#_Toc167438566)

[4′F-LN **7** Conformer G S125](#_Toc167438567)

[4′F-LN **7** Conformer H S126](#_Toc167438568)

[4′F-LN **7** Conformer I S127](#_Toc167438569)

[4′F-LN **7** Conformer J S128](#_Toc167438570)

[4′F-LN **7** Conformer K S129](#_Toc167438571)

[4′F-LN **7** Conformer L S130](#_Toc167438572)

[6′F-LN **8** Conformer A S131](#_Toc167438573)

[6′F-LN **8** Conformer B S132](#_Toc167438574)

[6′F-LN **8** Conformer C S133](#_Toc167438575)

[6′F-LN **8** Conformer D S134](#_Toc167438576)

[6′F-LN **8** Conformer E S135](#_Toc167438577)

[6′F-LN **8** Conformer F S136](#_Toc167438578)

[6′F-LN **8** Conformer G S137](#_Toc167438579)

[6′F-LN **8** Conformer H S138](#_Toc167438580)

[6′F-LN **8** Conformer I S139](#_Toc167438581)

[6′F-LN **8** Conformer J S140](#_Toc167438582)

[6′F-LN **8** Conformer K S141](#_Toc167438583)

[6′F-LN **8** Conformer L S142](#_Toc167438584)

[GlcNAcβ-OMe (implicit DMSO solvation) S143](#_Toc167438585)

[GlcNAcβ-OMe (explicit monodentate DMSO solvation) S144](#_Toc167438586)

[GlcNAcβ-OMe (explicit bidentate DMSO solvation) S145](#_Toc167438587)

[3F-LN **3** Geometry for Log *P* calculation S146](#_Toc167438588)

[6F-LN **4** Geometry for Log *P* calculation S147](#_Toc167438589)

[2′F-LN **5** Geometry for Log *P* calculation S148](#_Toc167438590)

[3′F-LN **6** Geometry for Log *P* calculation S149](#_Toc167438591)

[4′F-LN **7** Geometry for Log *P* calculation S150](#_Toc167438592)

[6′F-LN **8** Geometry for Log *P* calculation S151](#_Toc167438593)

# Copies of NMR spectra

## ^1^H NMR (400 MHz, DMSO-*d*_6_) LN **2**

**
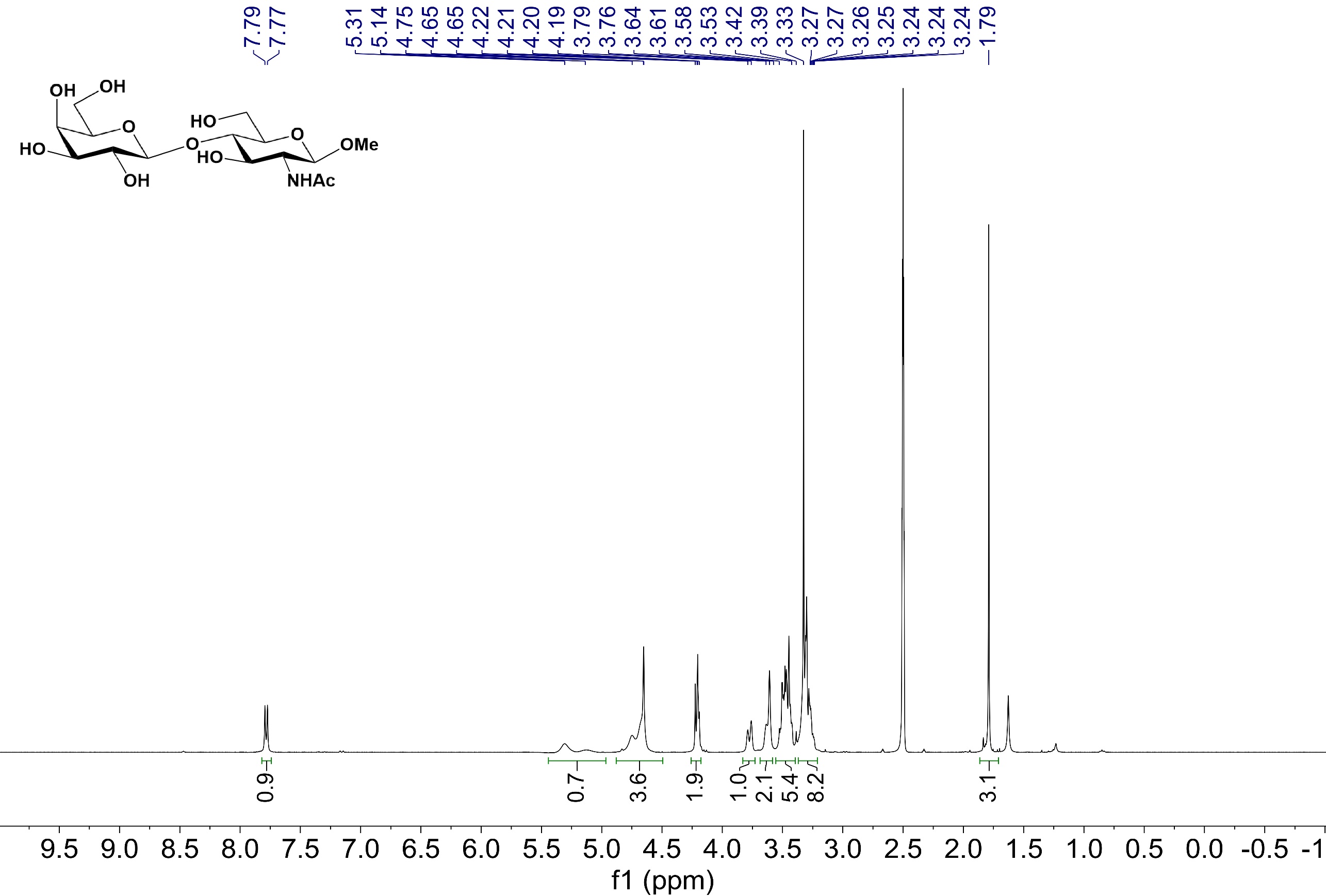
**

## ^13^C{^1^H} NMR (101 MHz, DMSO-*d*_6_) LN **2**

**
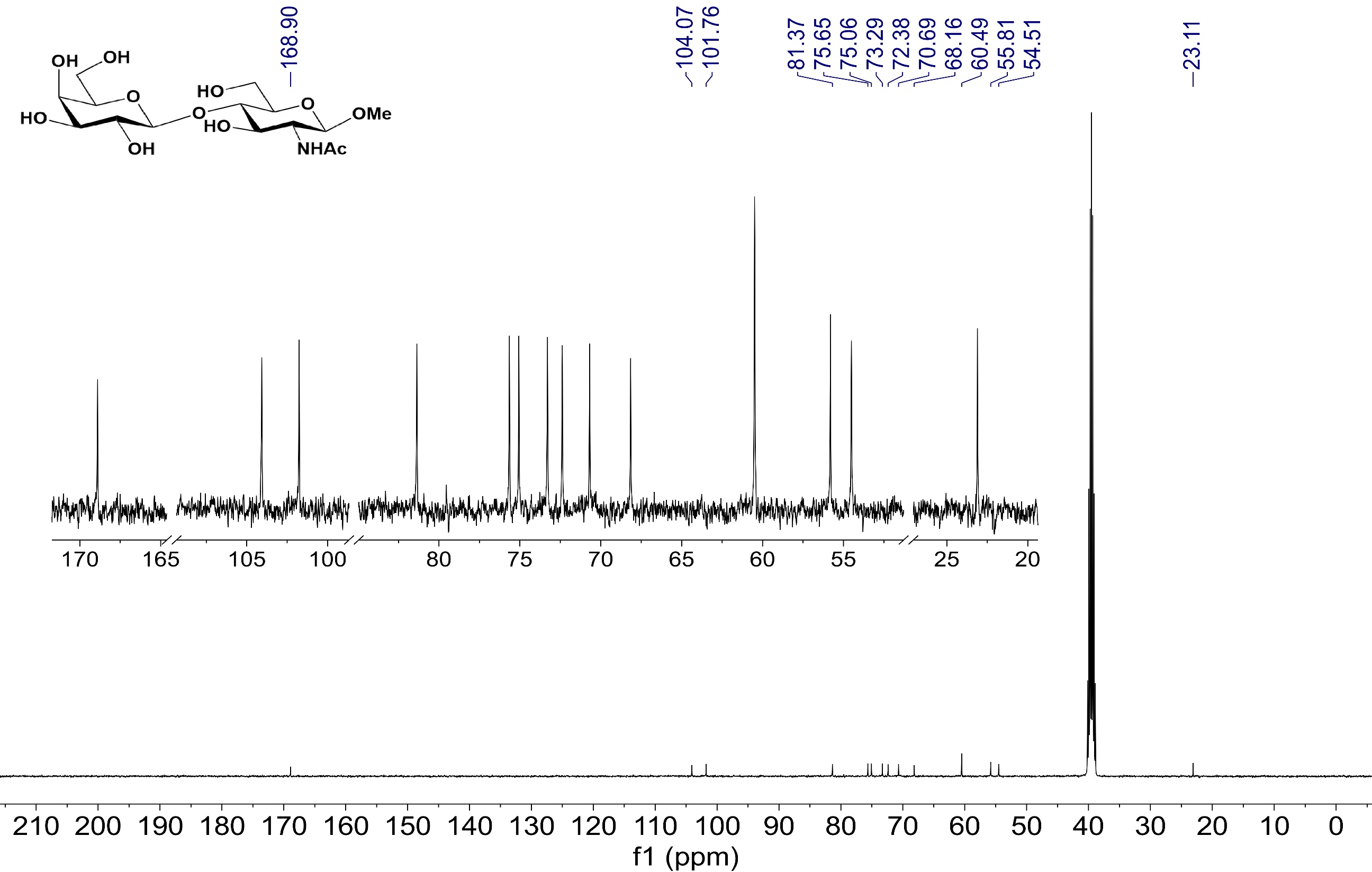
**

## ^1^H-^1^H COSY NMR (DMSO-*d*_6_) LN **2**


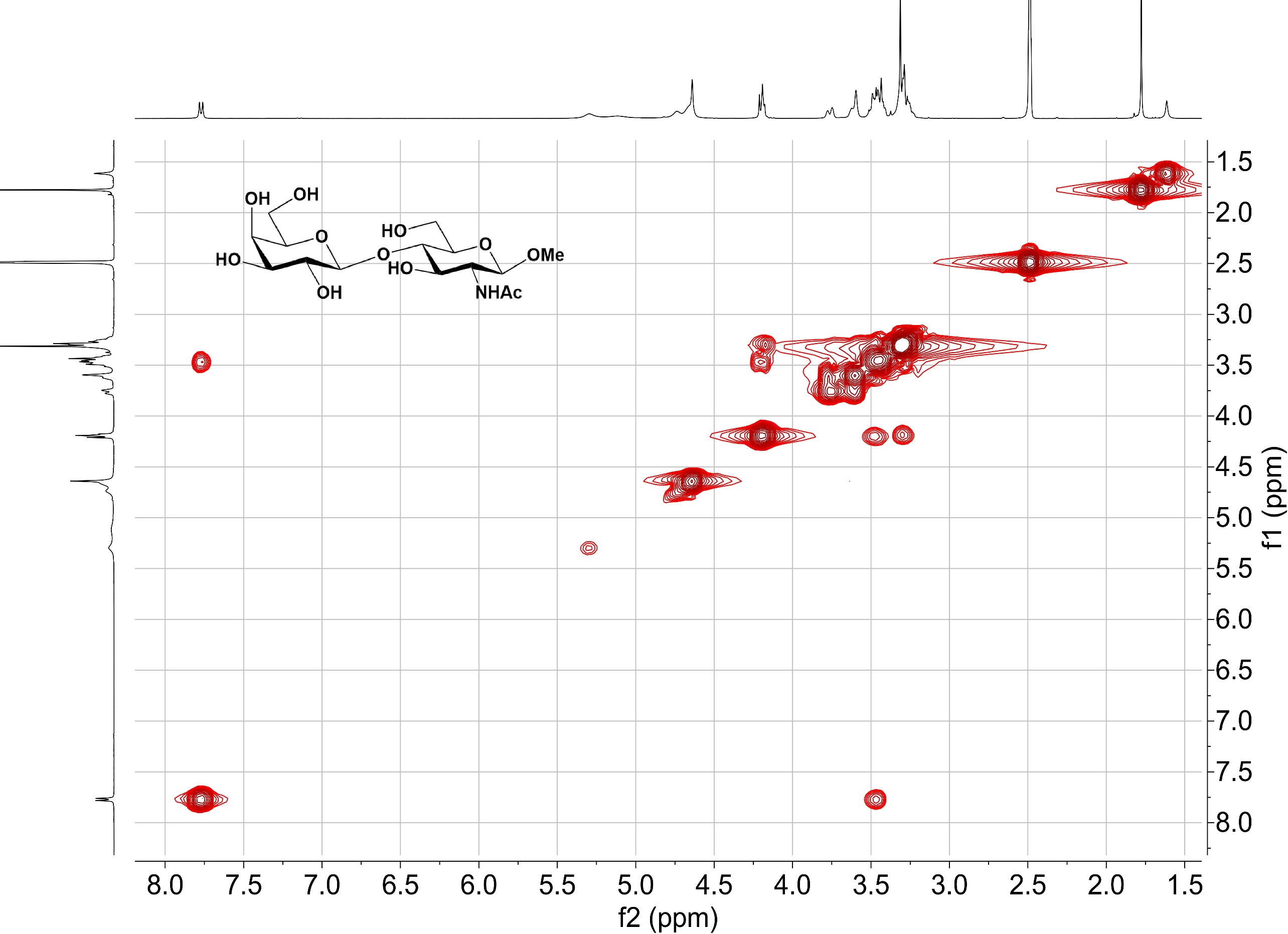


## ^1^H-^13^C HSQC NMR (DMSO-*d*_6_) LN **2**

**
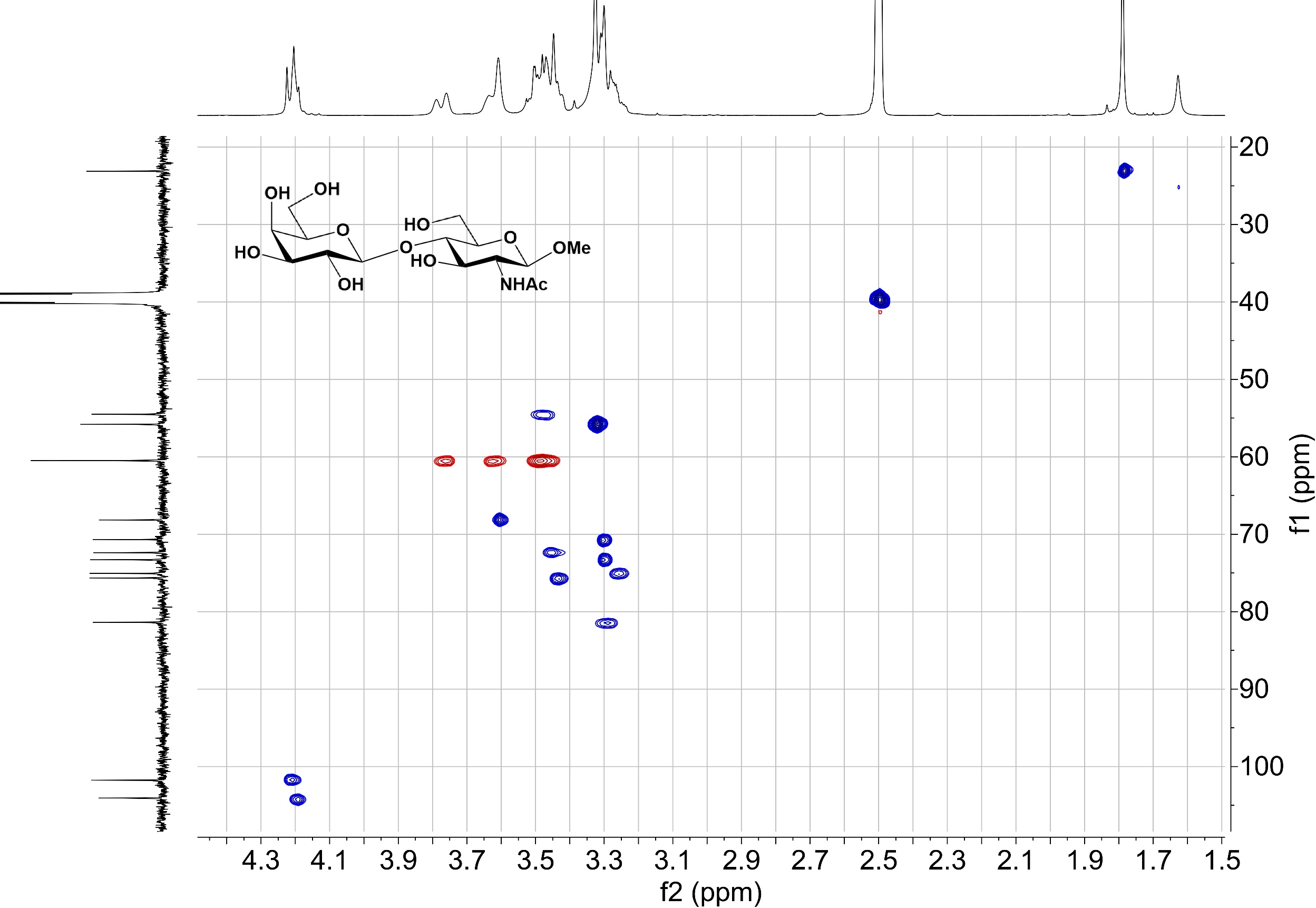
**

## ^1^H-^13^C HMBC NMR (DMSO-*d*_6_) LN **2**


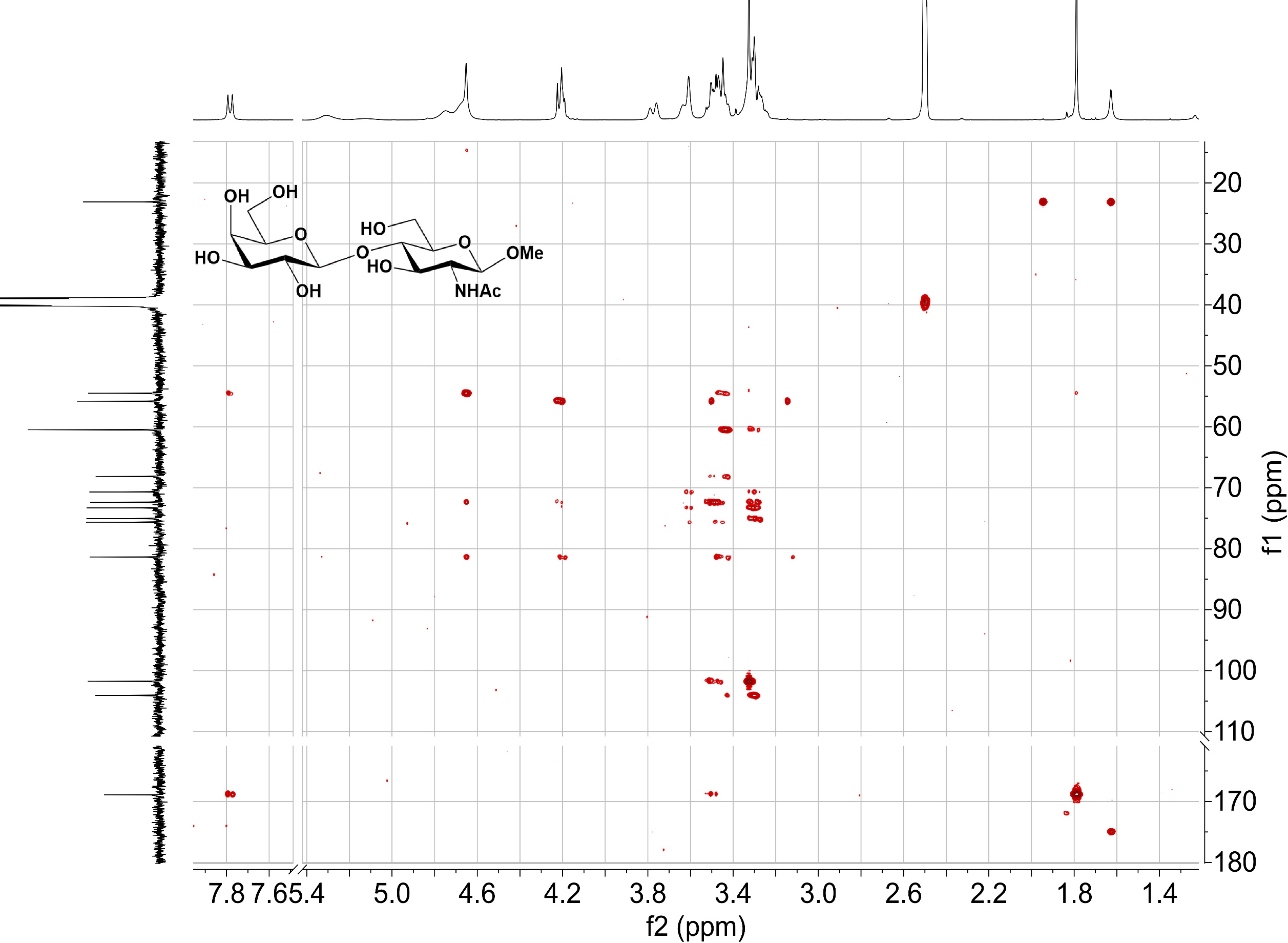


## ^1^H-^1^H ROESY NMR (DMSO-*d*_6_) LN **2**

^
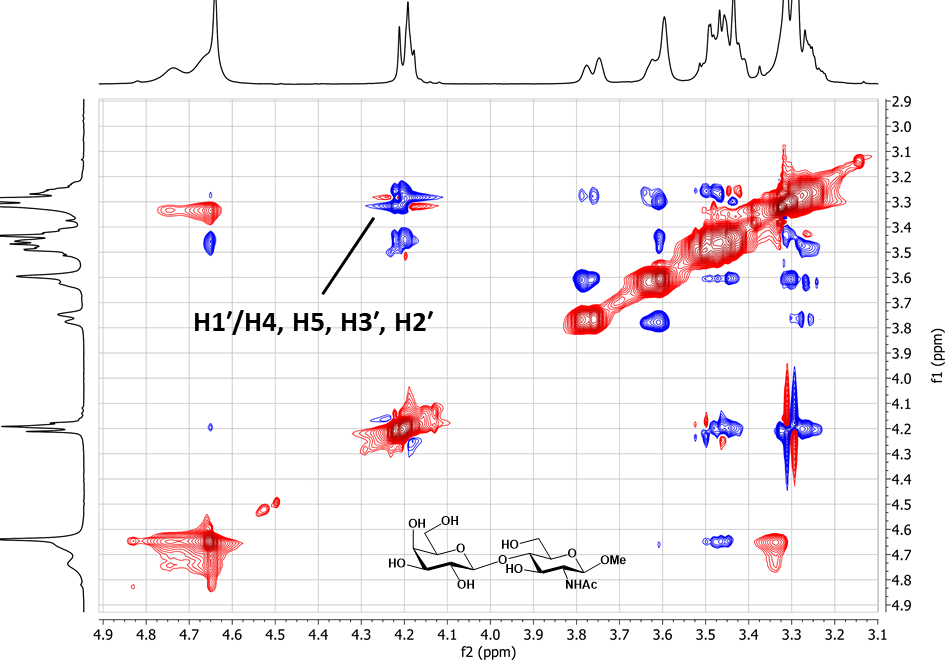
^

## ^1^H-^1^H ROESY NMR (DMSO-*d*_6_) LN **2**

^
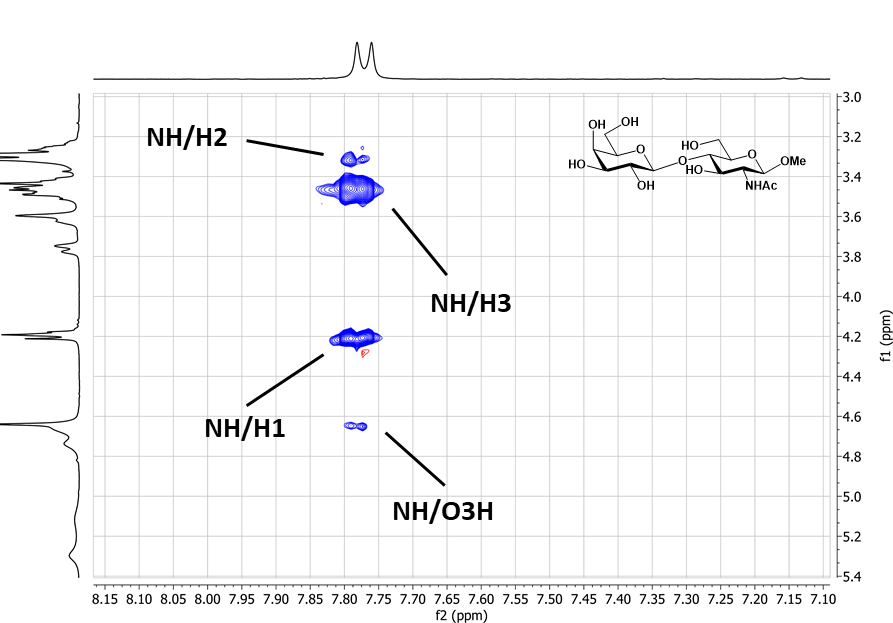
^

## ^1^H-^1^H ROESY NMR (DMSO-*d*_6_) LN **2**

^
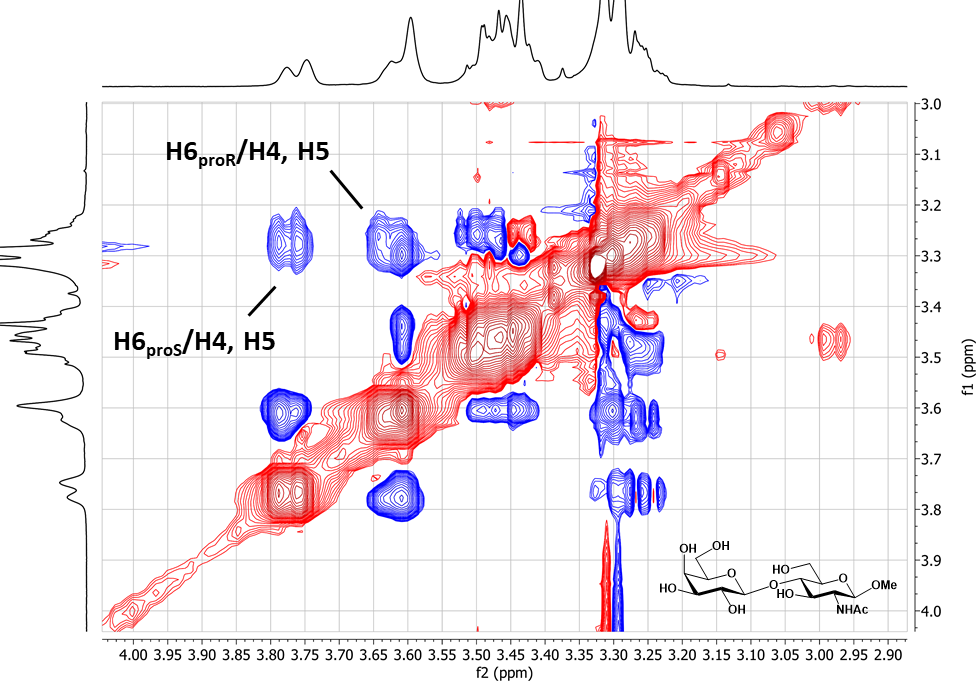
^

## Selective Homonuclear Decoupled ^1^H-NMR (DMSO-*d*_6_) LN **2**

(irradiation frequency: 4.695 ppm)

^
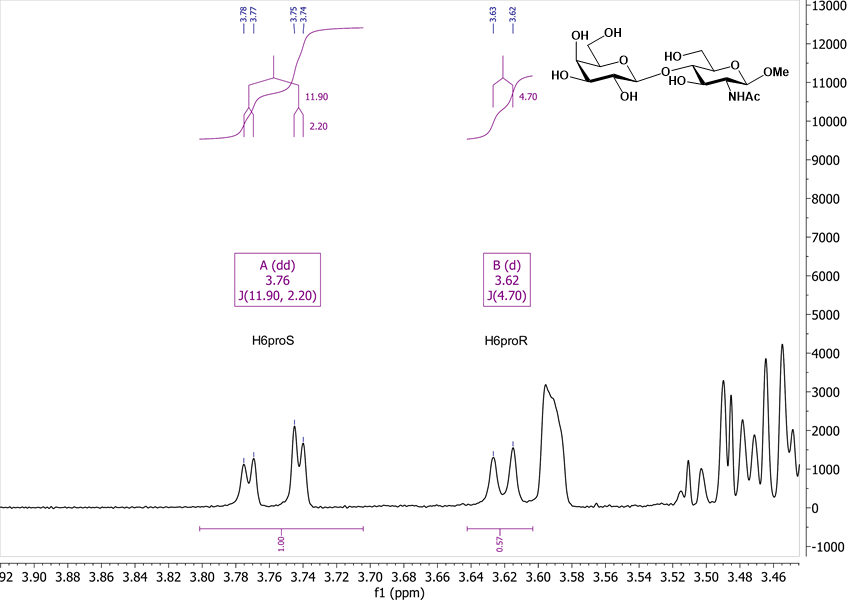
^

## Temperature Dependent ^1^H-NMR (DMSO-*d*_6_) LN **2**

^
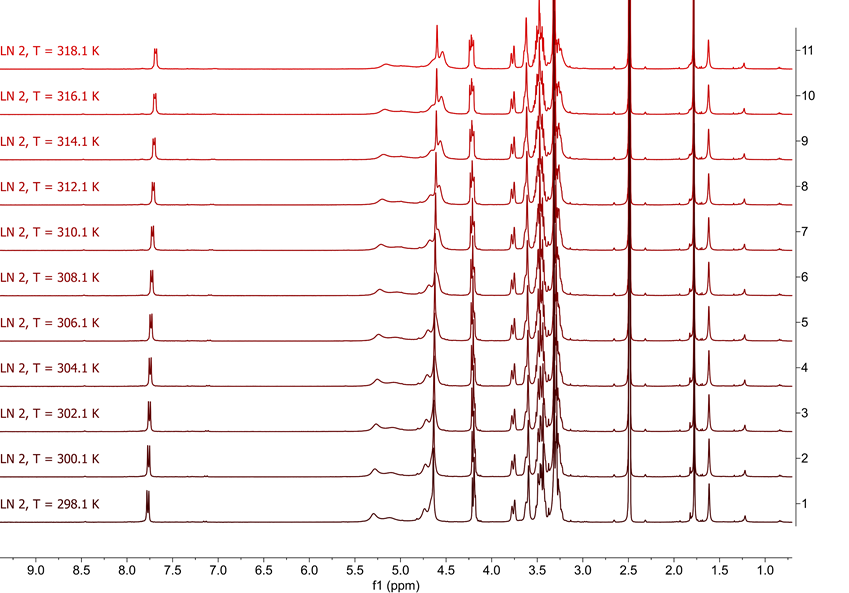
^

## ^1^H NMR (400 MHz, DMSO-*d*_6_) 3F-LN **3**

**
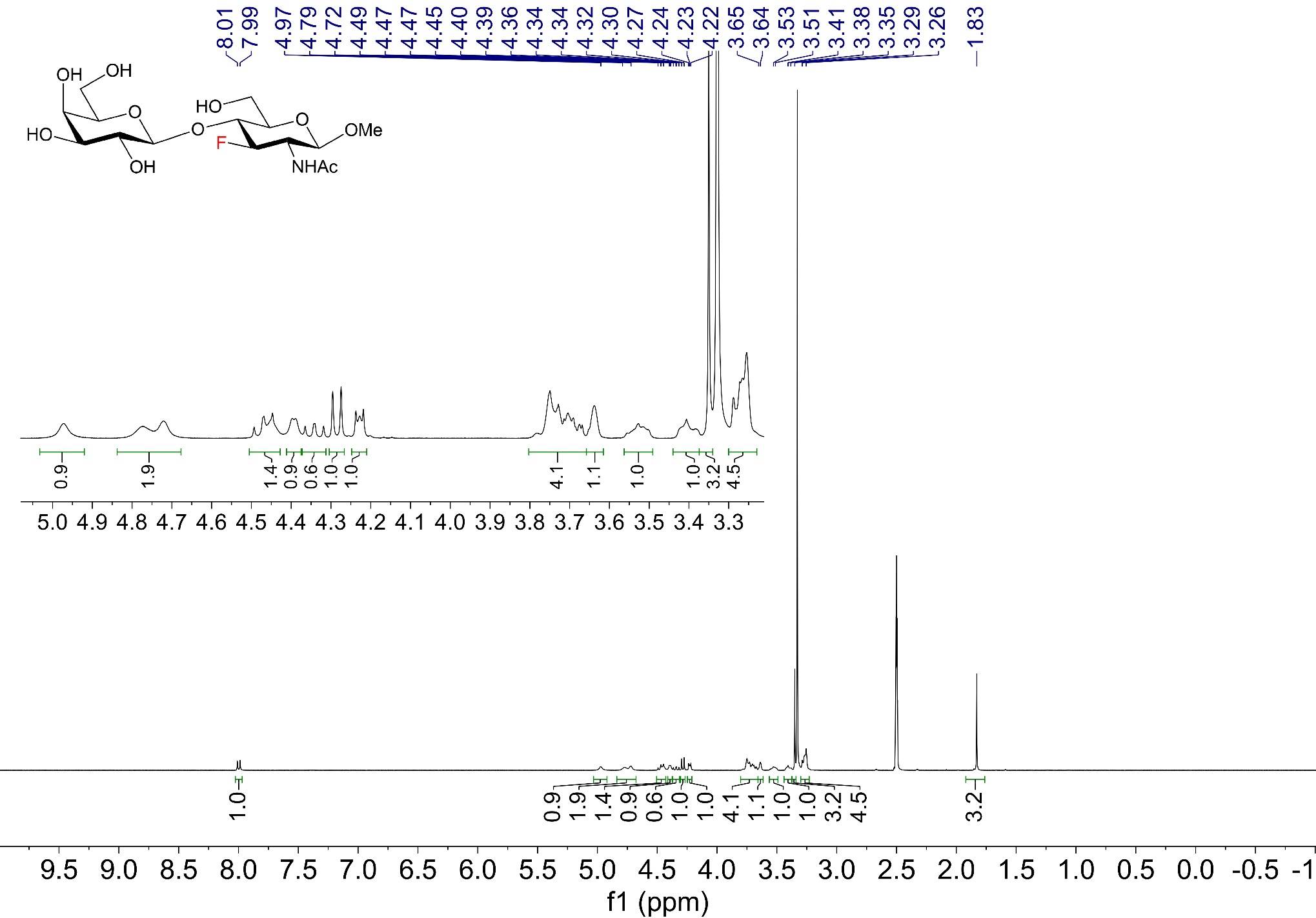
**

## ^13^C{^1^H} APT NMR (126 MHz, DMSO-*d*_6_) 3F-LN **3**

**
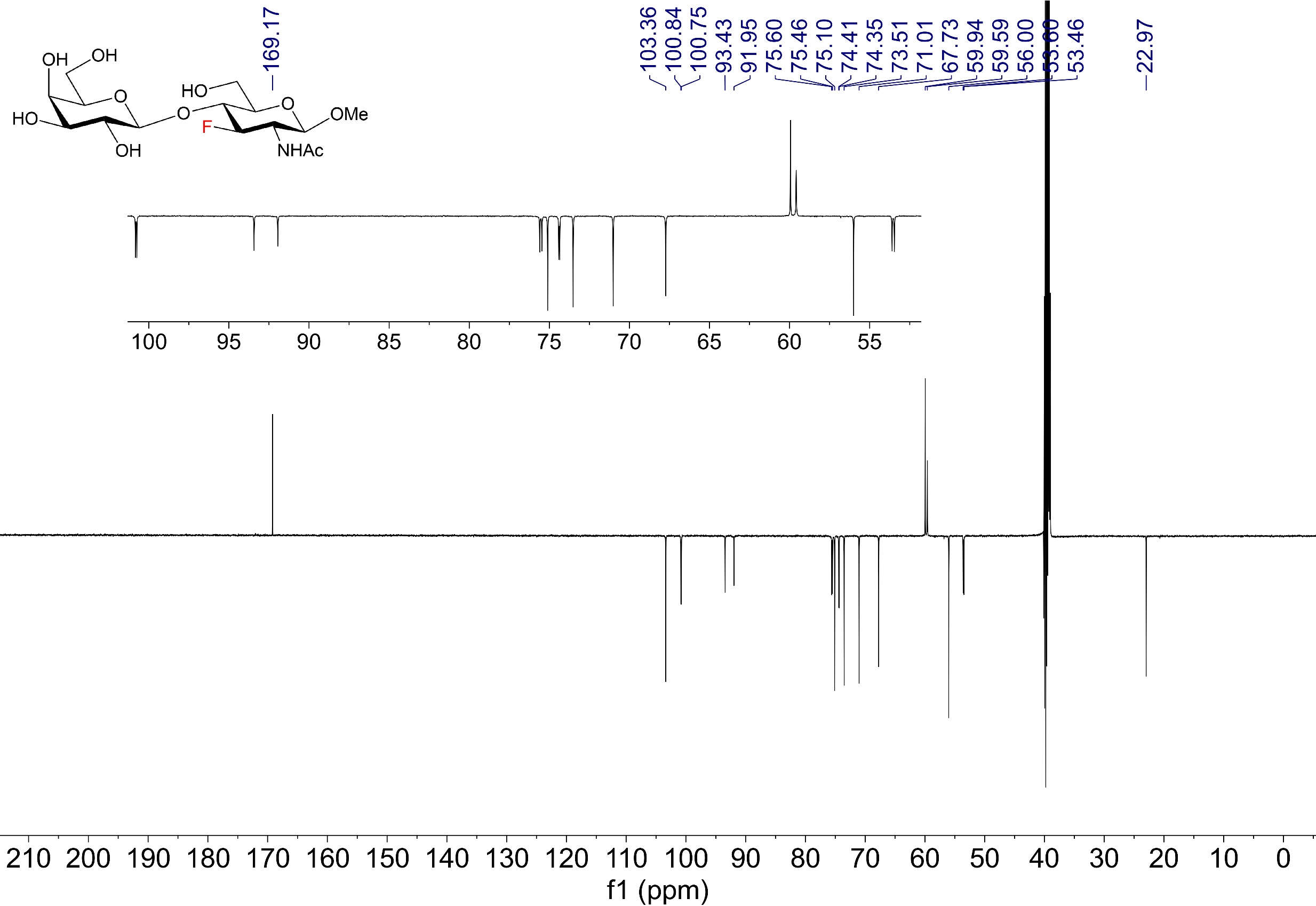
**

## ^19^F NMR (376 MHz, DMSO-*d*_6_) 3F-LN **3**

**
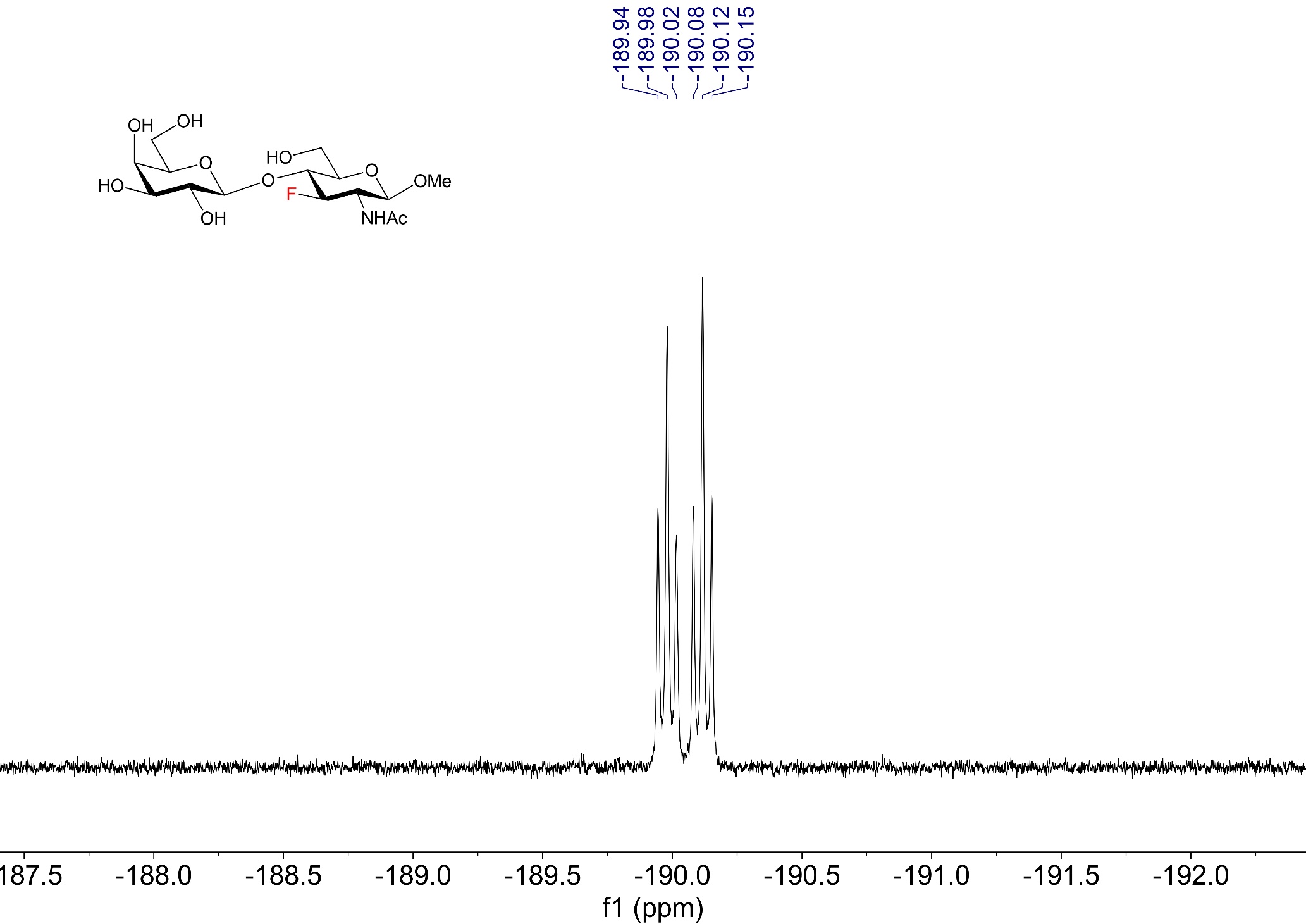
**

## ^1^H-^1^H COSY NMR (DMSO-*d*_6_) 3F-LN **3**


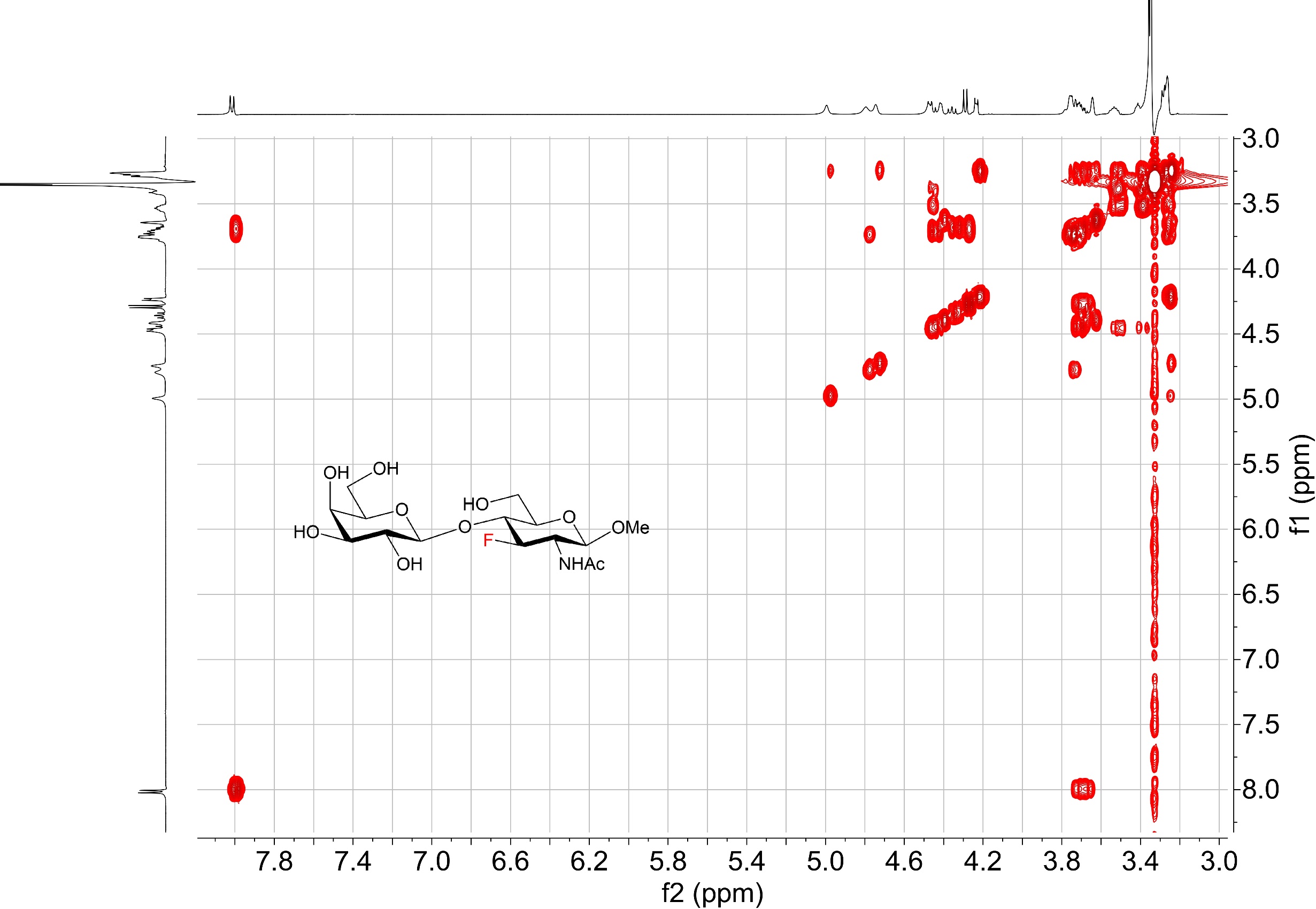


## ^1^H-^13^C HSQC NMR (DMSO-*d*_6_) 3F-LN **3**

**
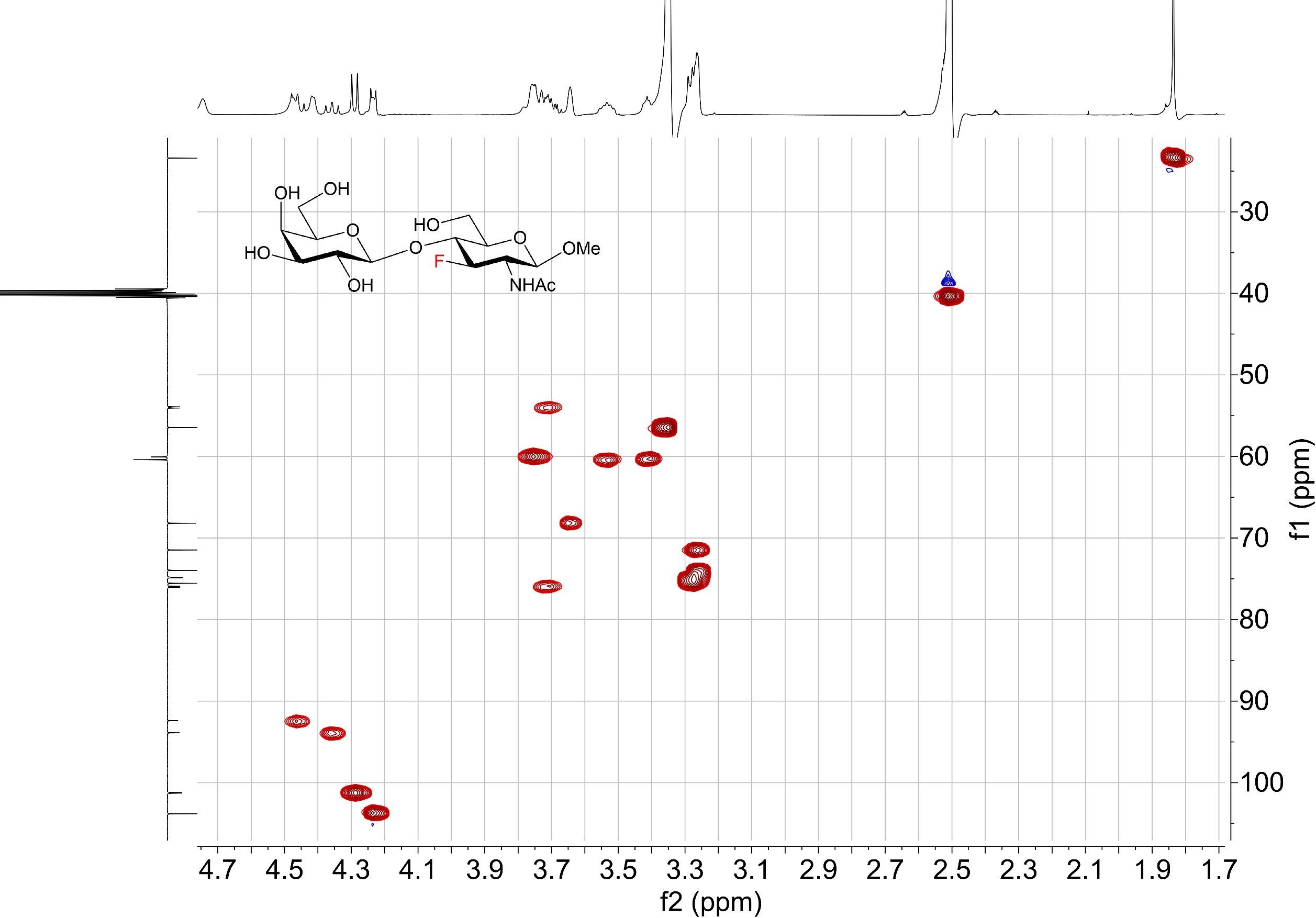
**

## ^1^H-^13^C HMBC NMR (DMSO-*d*_6_) 3F-LN **3**


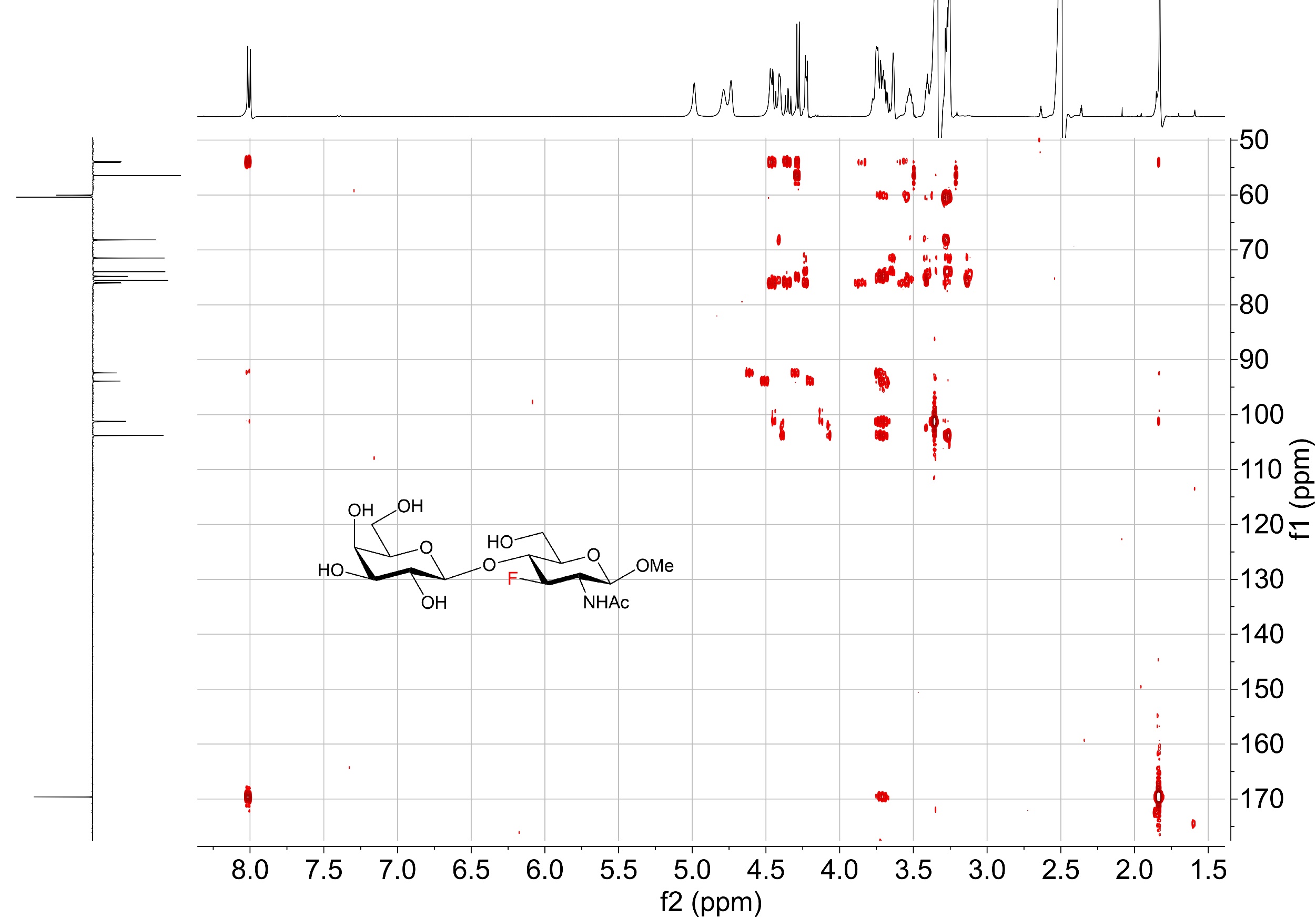


## ^1^H-^1^H ROESY NMR (DMSO-*d*_6_) 3F-LN **3**

**
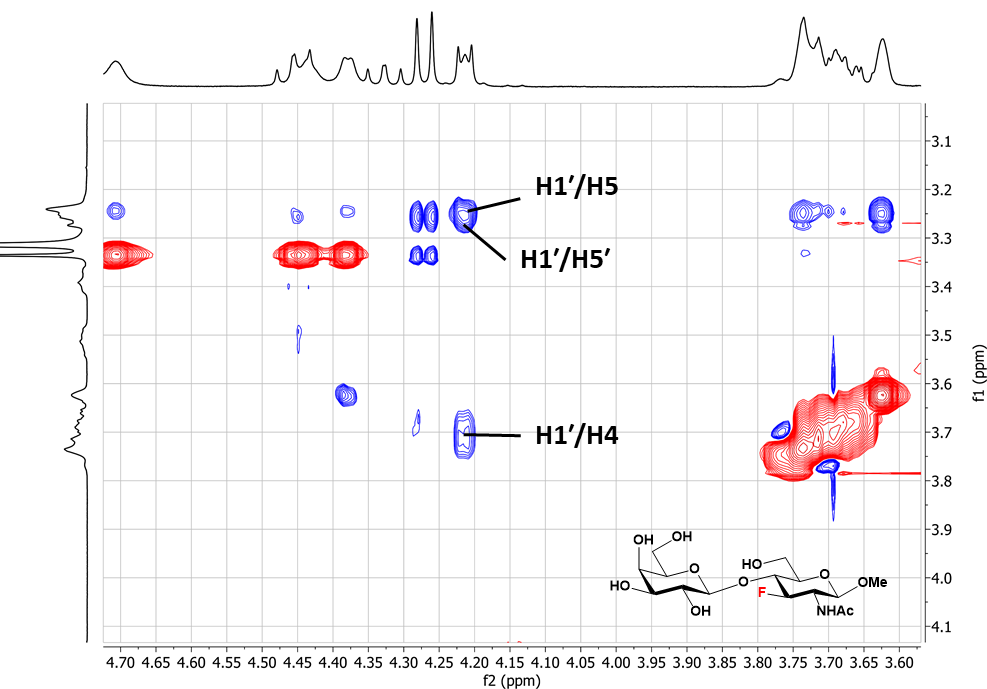
**

## ^1^H-^1^H ROESY NMR (DMSO-*d*_6_) 3F-LN **3**

**
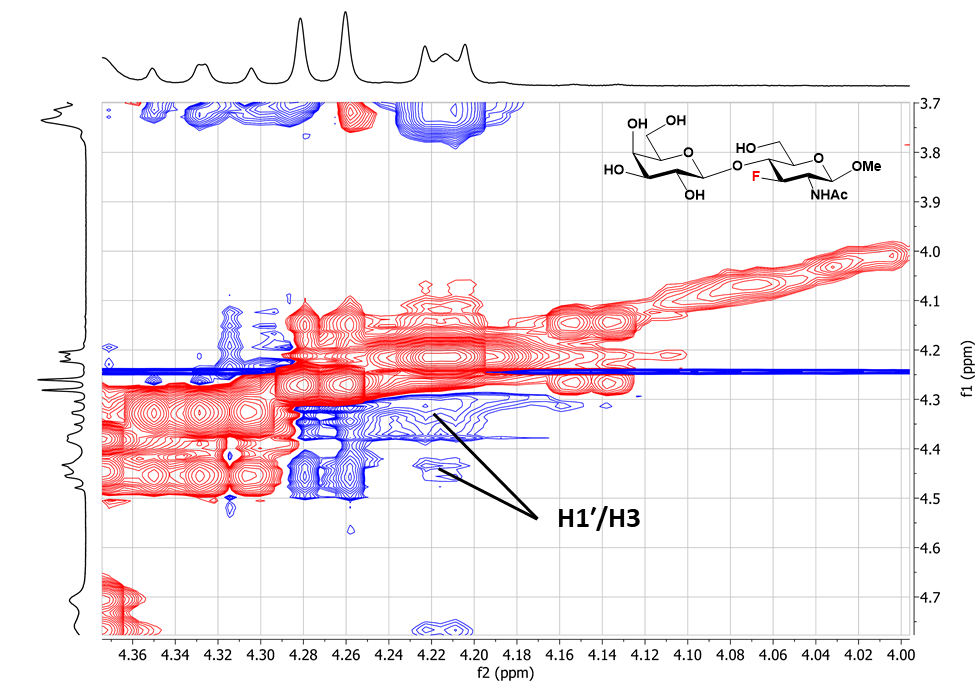
**

## ^1^H-^1^H ROESY NMR (DMSO-*d*_6_) 3F-LN **3**


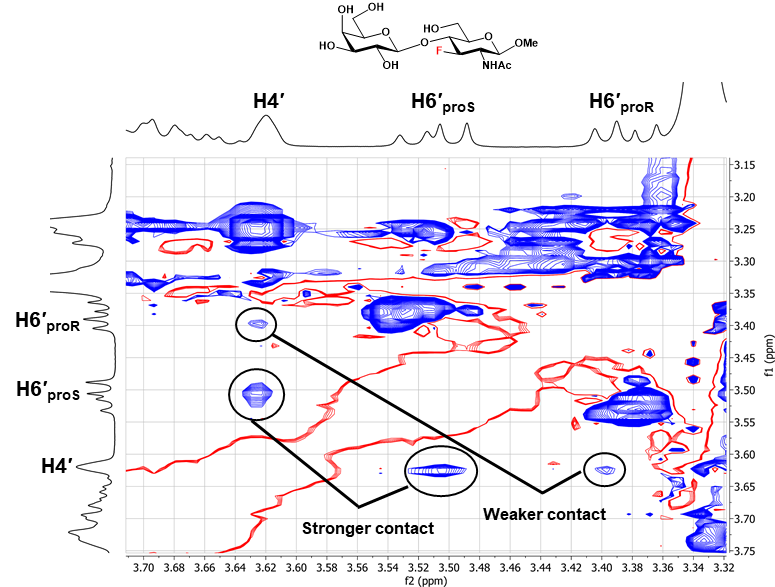


^1^H-^1^H ROESY NMR (DMSO-*d*_6_) 3F-LN **3
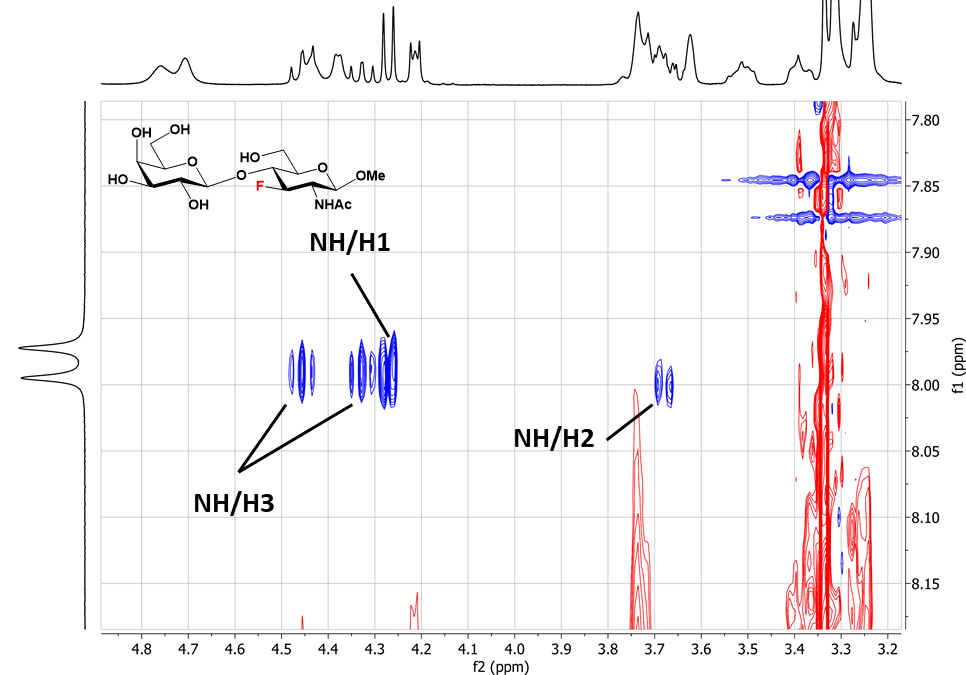
**

## ^1^H-^1^H ROESY NMR (DMSO-*d*_6_) 3F-LN **3**

**
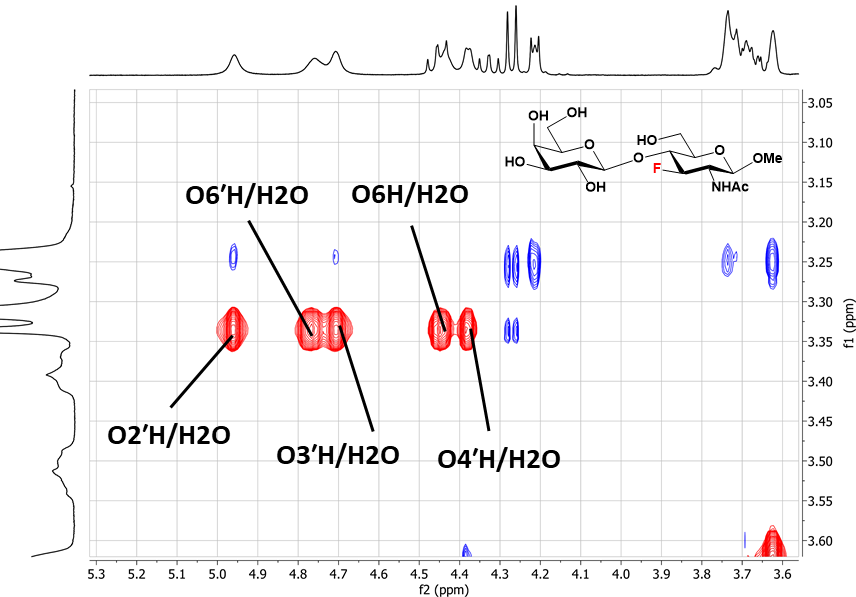
**

## Selective Homonuclear Decoupled ^1^H-NMR (DMSO-*d*_6_) 3F-LN **3**

(irradiation frequency: 4.475 ppm)

**
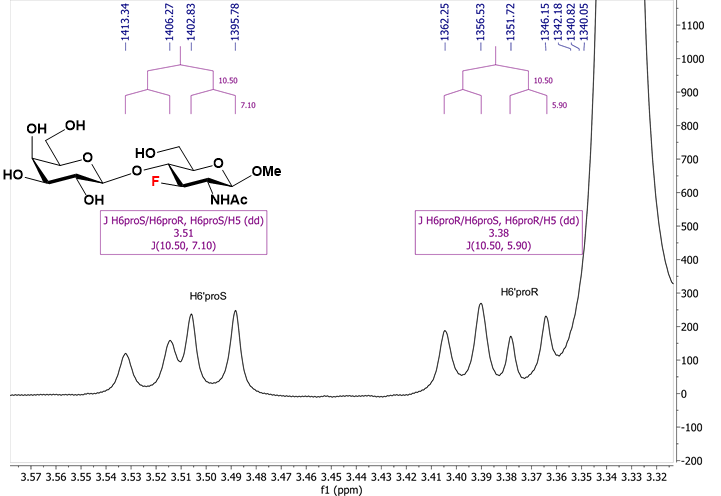
**

## 1D Selective Gradient ^1^H-^1^H TOCSY NMR (DMSO-*d*_6_) 3F-LN **3**

irradiation frequency: 7.997 ppm, Bruker Avance III™ HD 850 MHz, mixing time 120 ms)


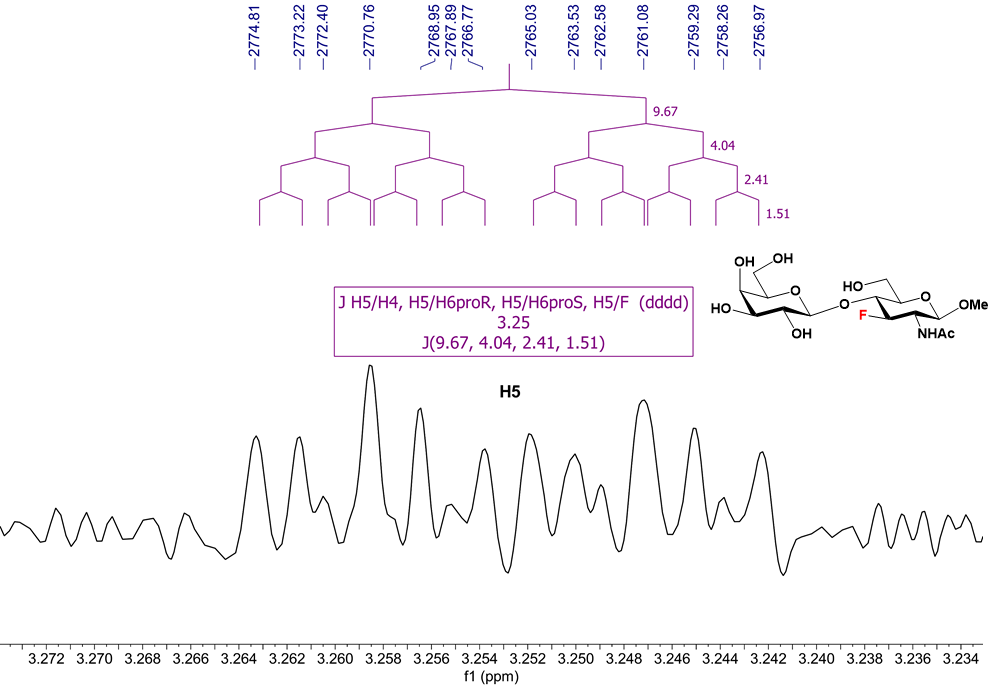


## Temperature Dependent ^1^H-NMR (DMSO-*d*_6_) 3F-LN **3**

^
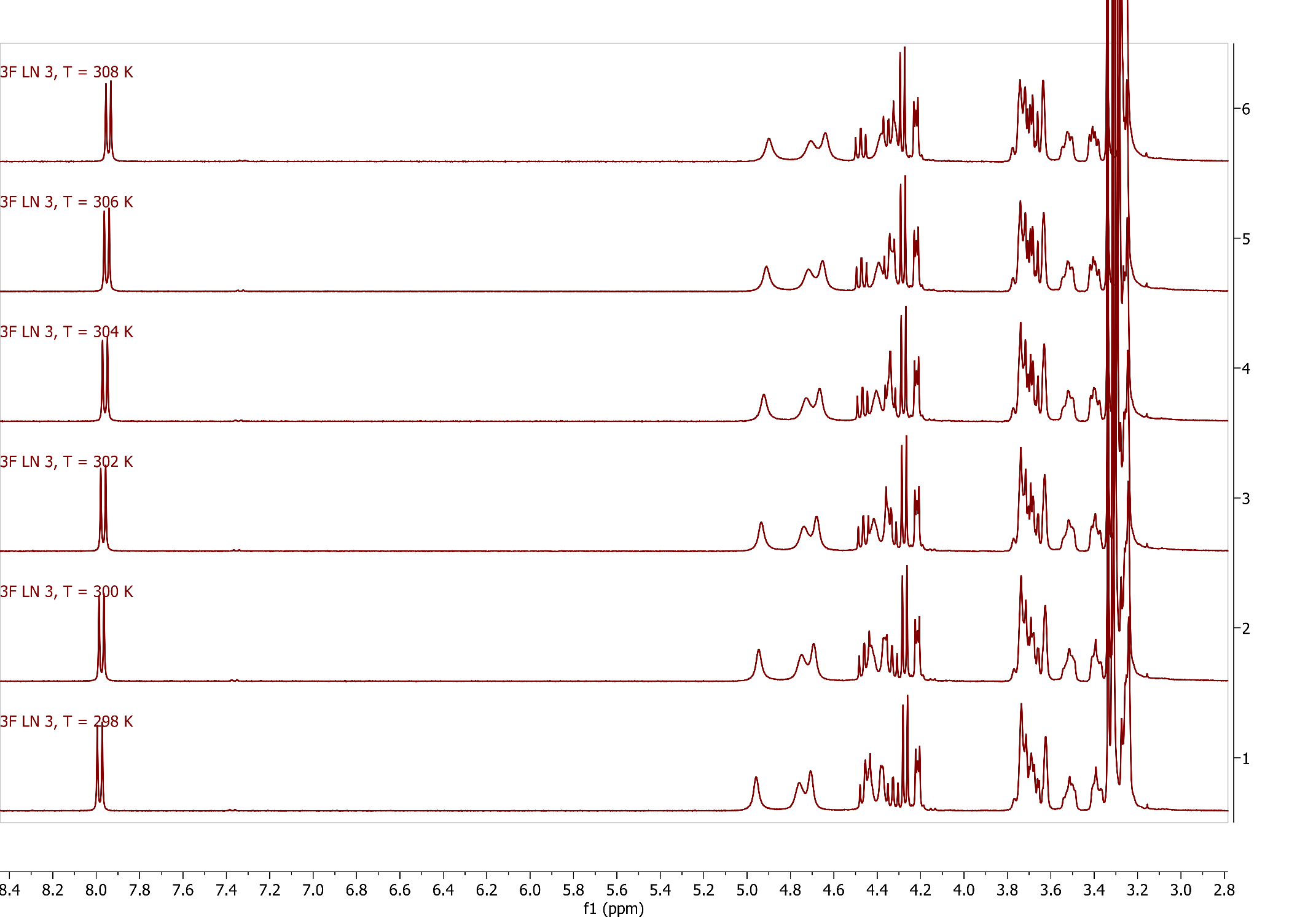
^

## ^1^H NMR (400 MHz, DMSO-*d*_6_) 6F-LN **4**

**
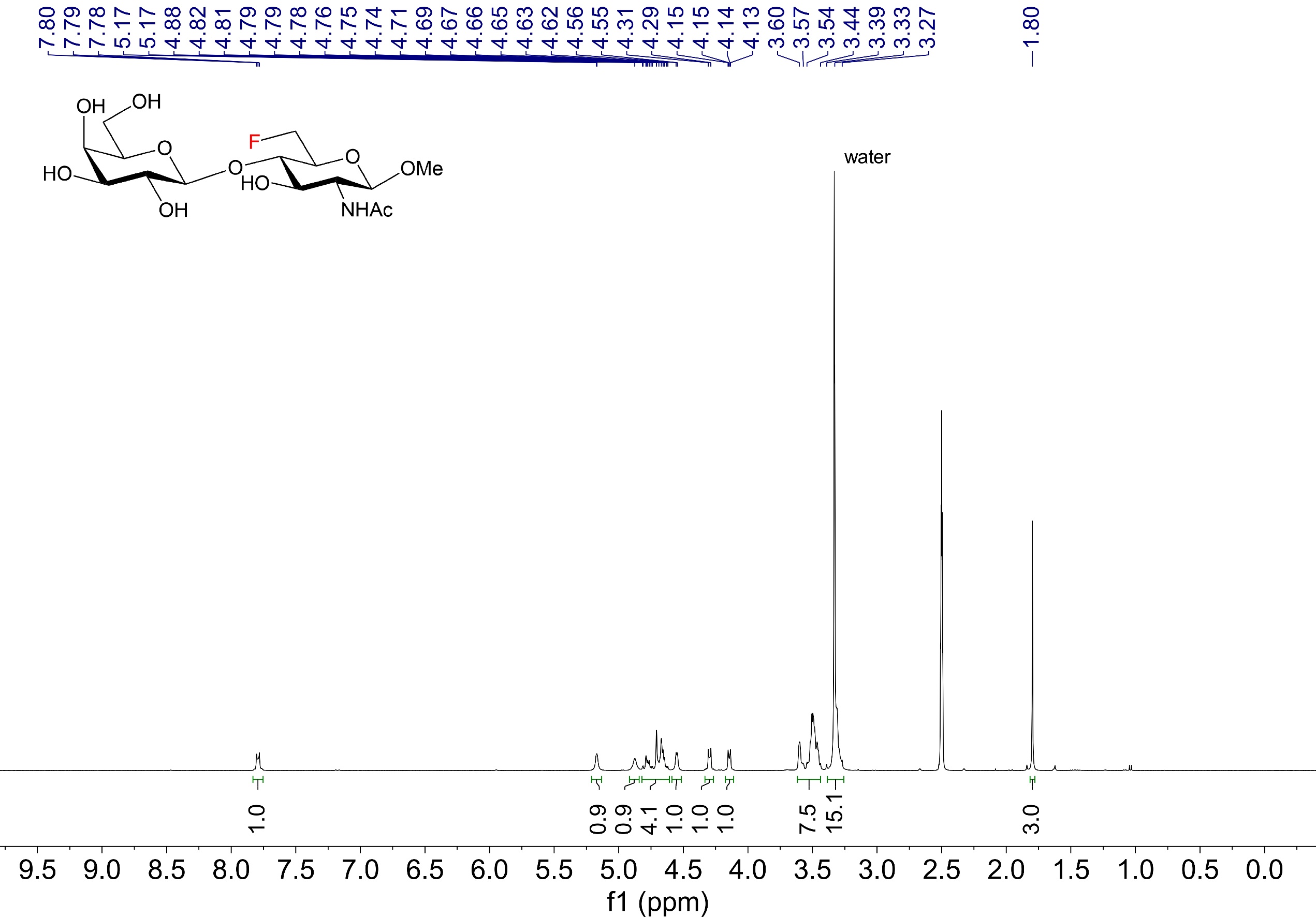
**

## ^13^C{^1^H} APT NMR (126 MHz, DMSO-*d*_6_) 6F-LN **4**

**
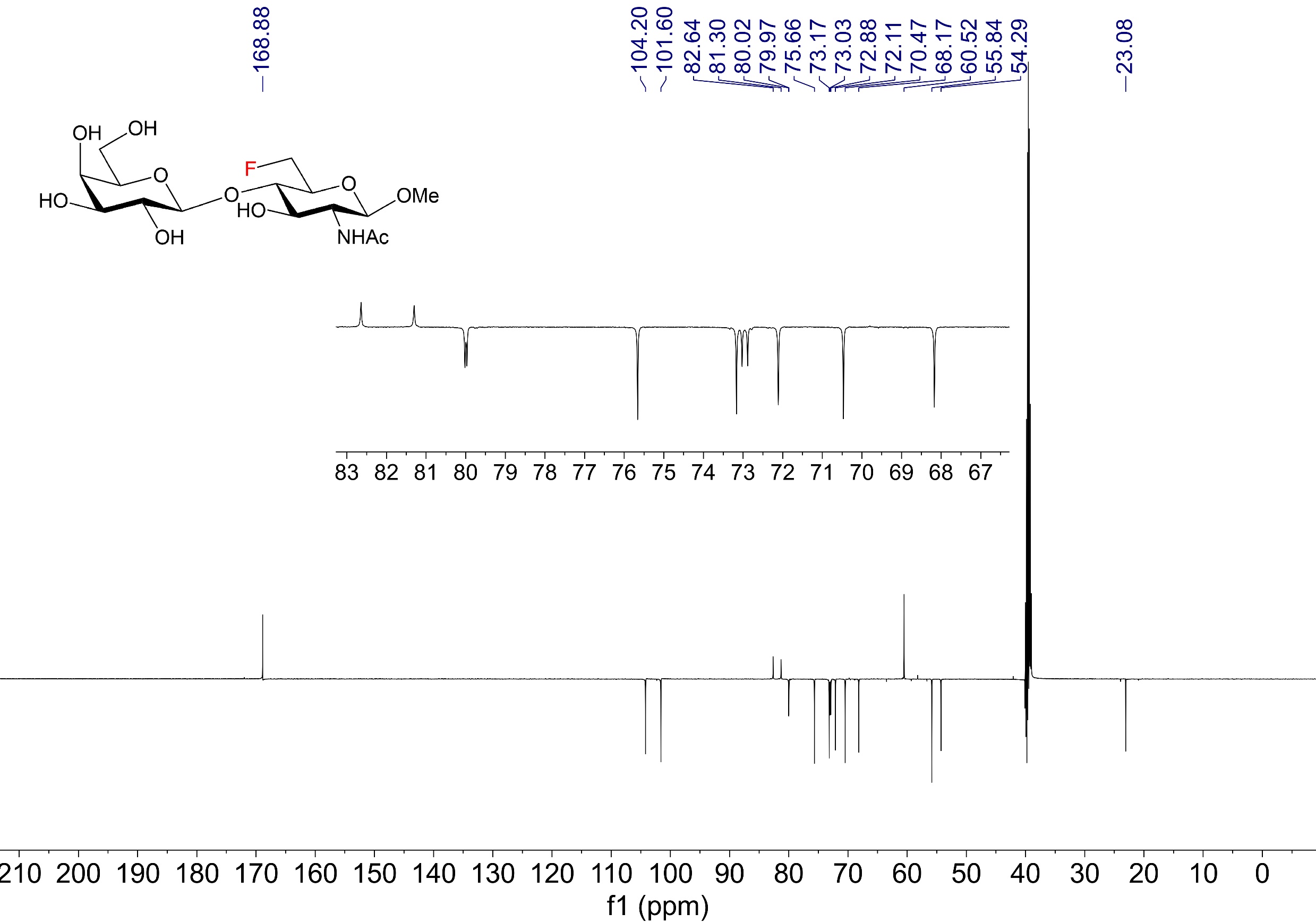
**

## ^19^F NMR (376 MHz, DMSO-*d*_6_) 6F-LN **4**

**
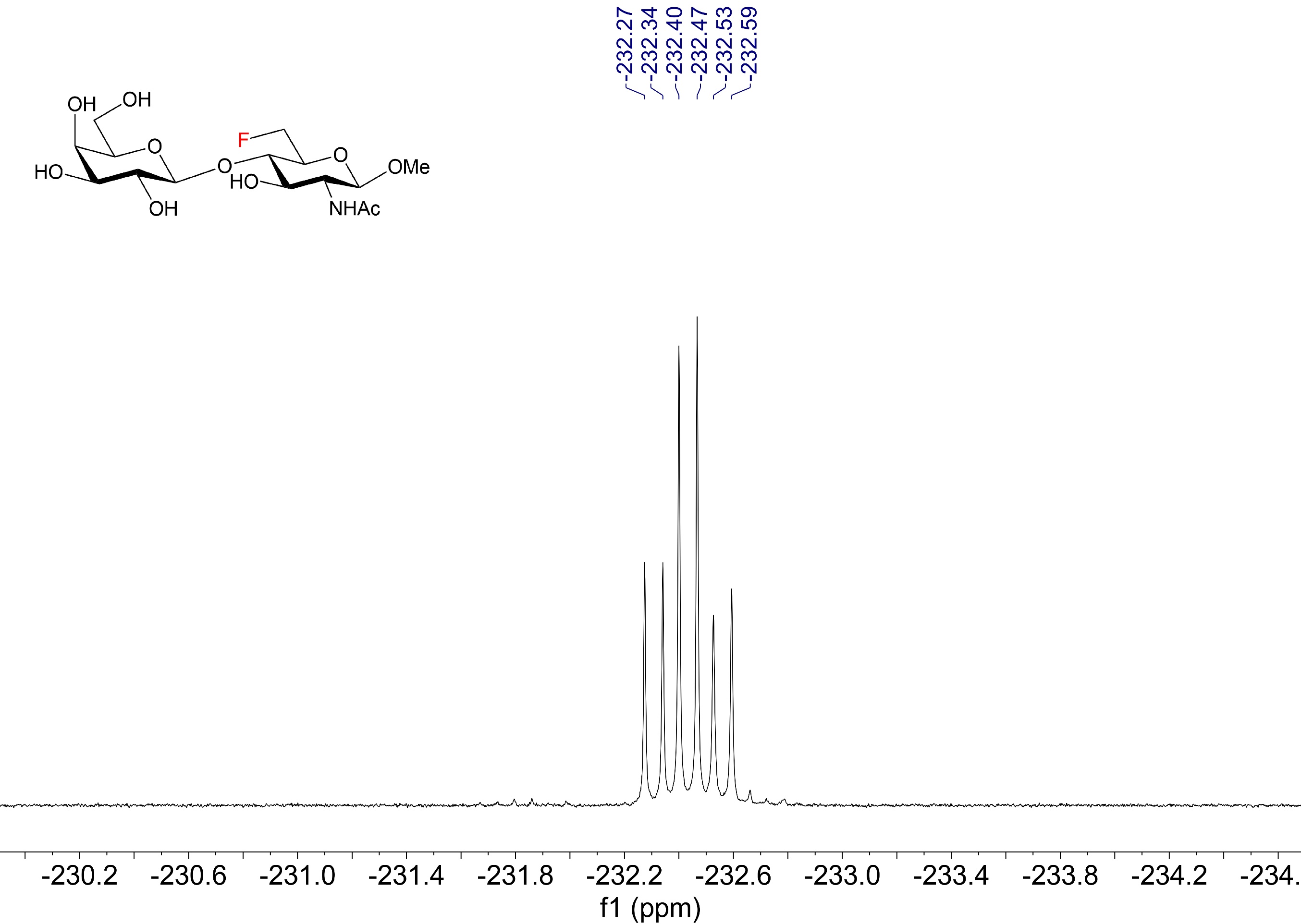
**

## ^1^H-^1^H COSY NMR (DMSO-*d*_6_) 6F-LN **4**


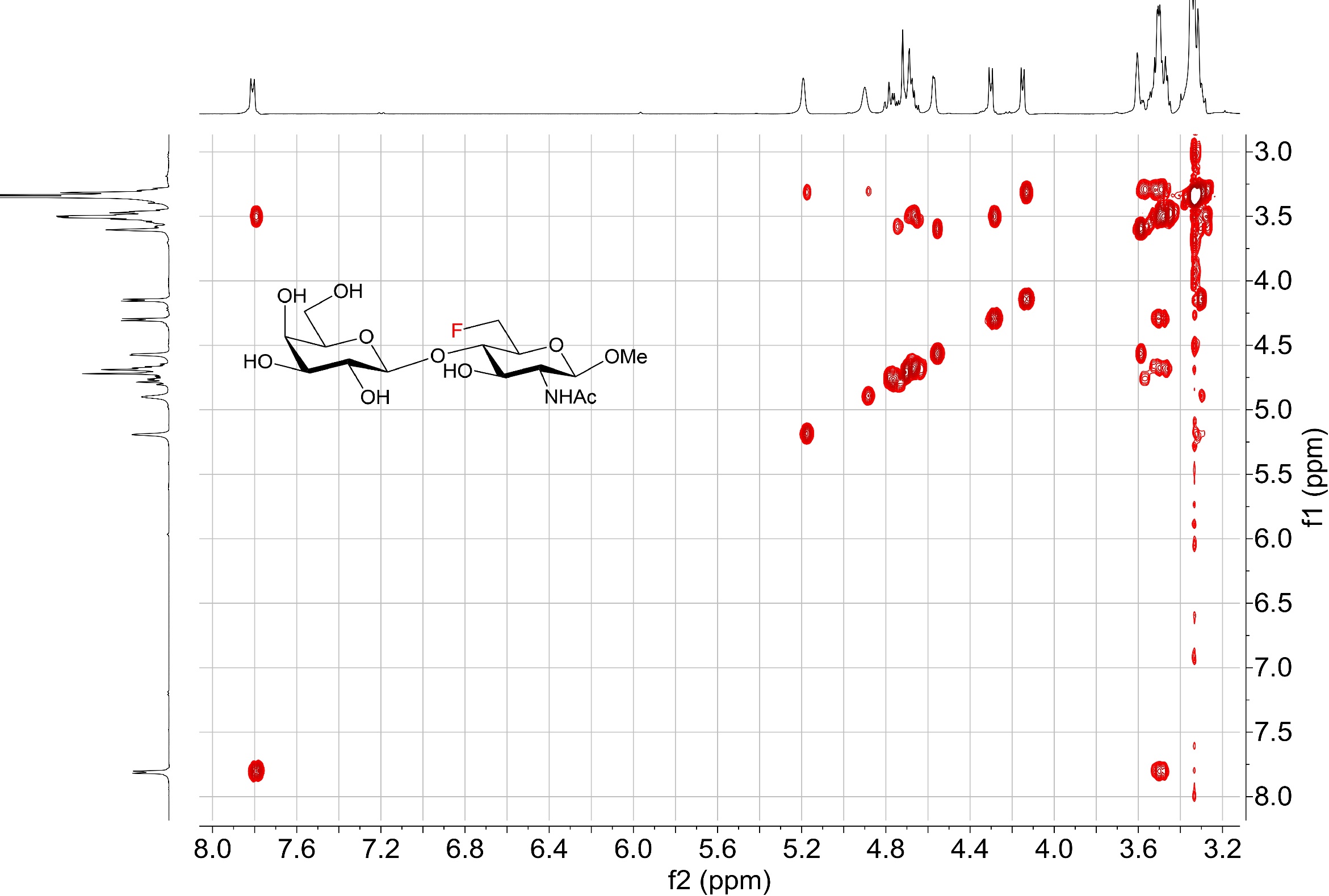


^1^H-^13^C HSQC NMR (DMSO-*d*_6_) 6F-LN **4
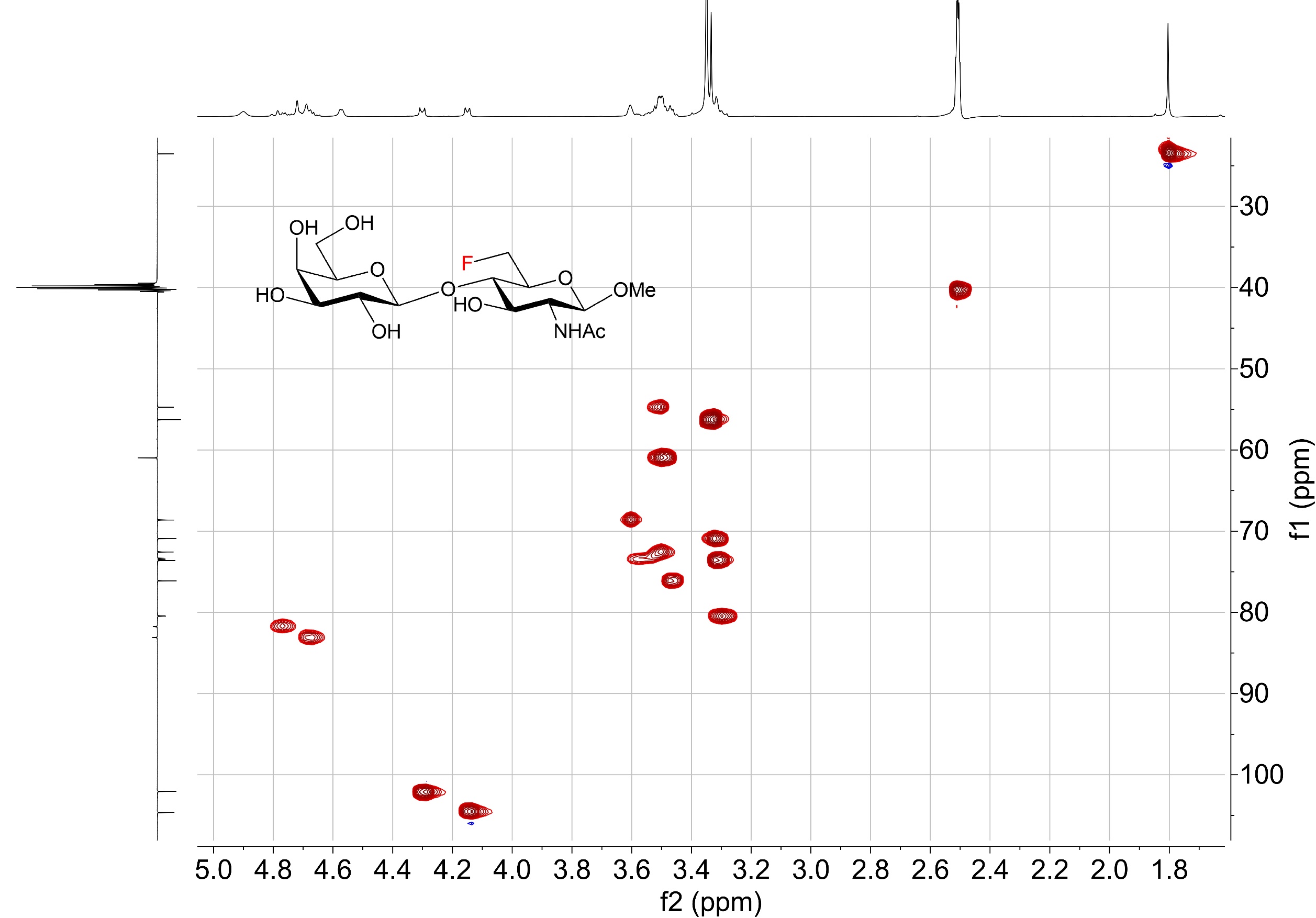
**

^1^H-^13^C HMBC NMR (DMSO-*d*_6_) 6F-LN **4**
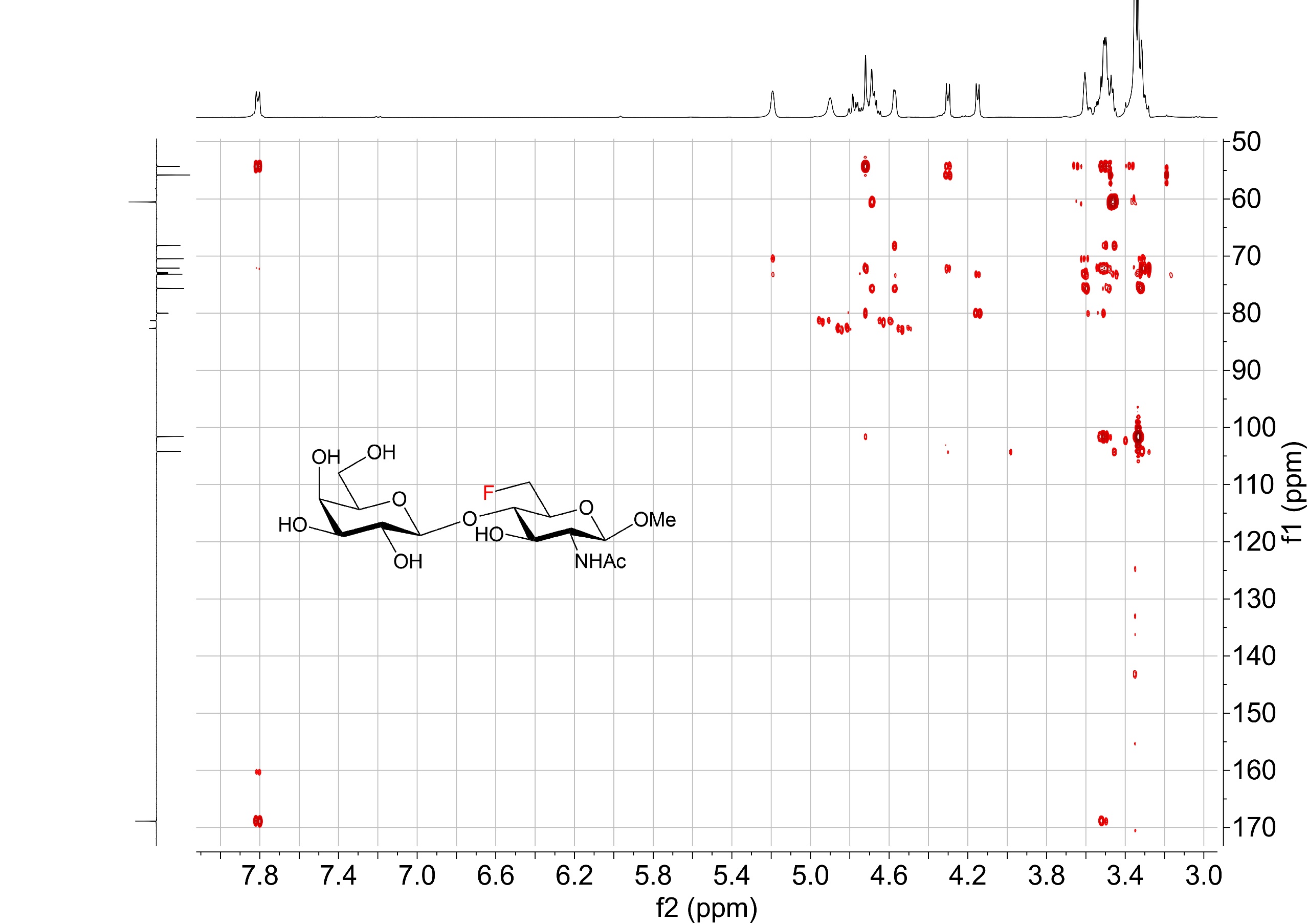


^1^H-^1^H ROESY NMR (DMSO-*d*_6_) 6F-LN **4**
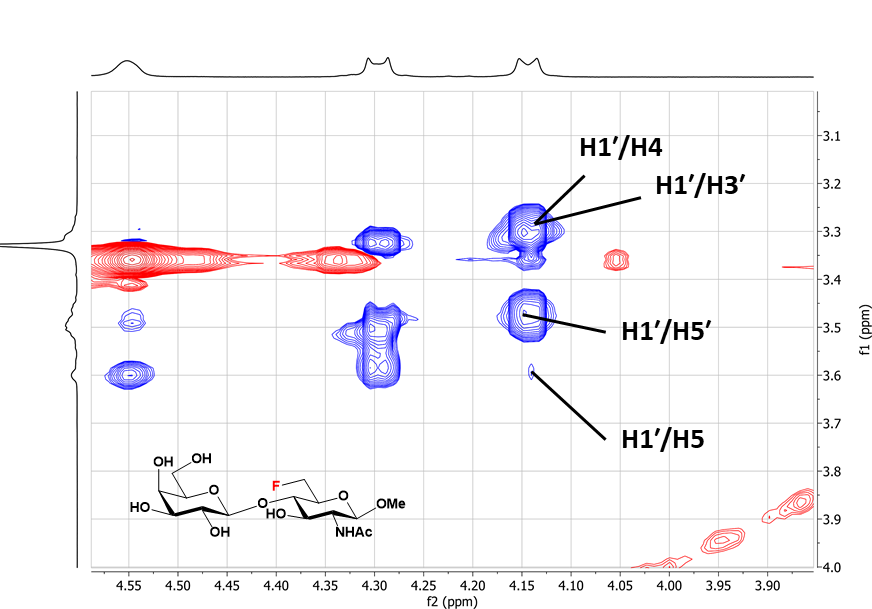


## ^1^H-^1^H ROESY NMR (DMSO-*d*_6_) 6F-LN **4**

^
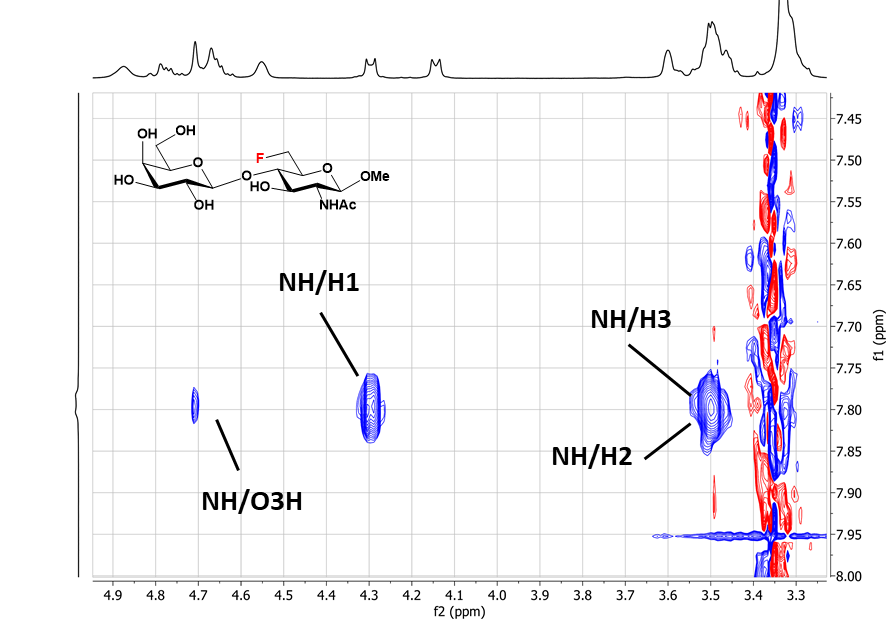
^

## ^1^H-^1^H ROESY NMR (DMSO-*d*_6_) 6F-LN **4**

^
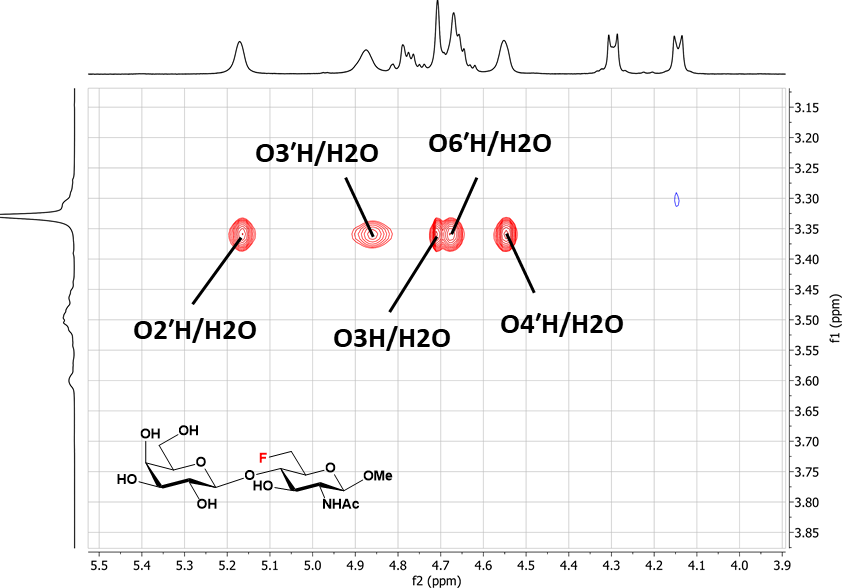
^

## ^1^H-^1^H ROESY NMR (DMSO-*d*_6_) 6F-LN **4**

^
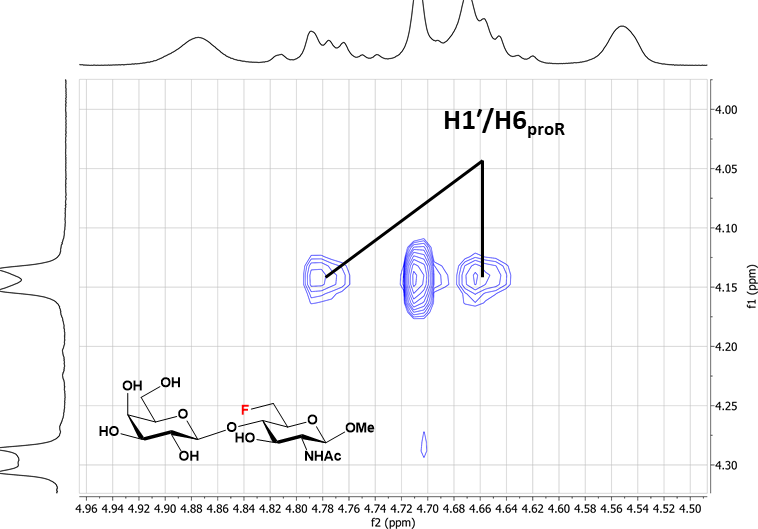
^

## ^1^H-^1^H ROESY NMR (DMSO-*d*_6_) 6F-LN **4**

^
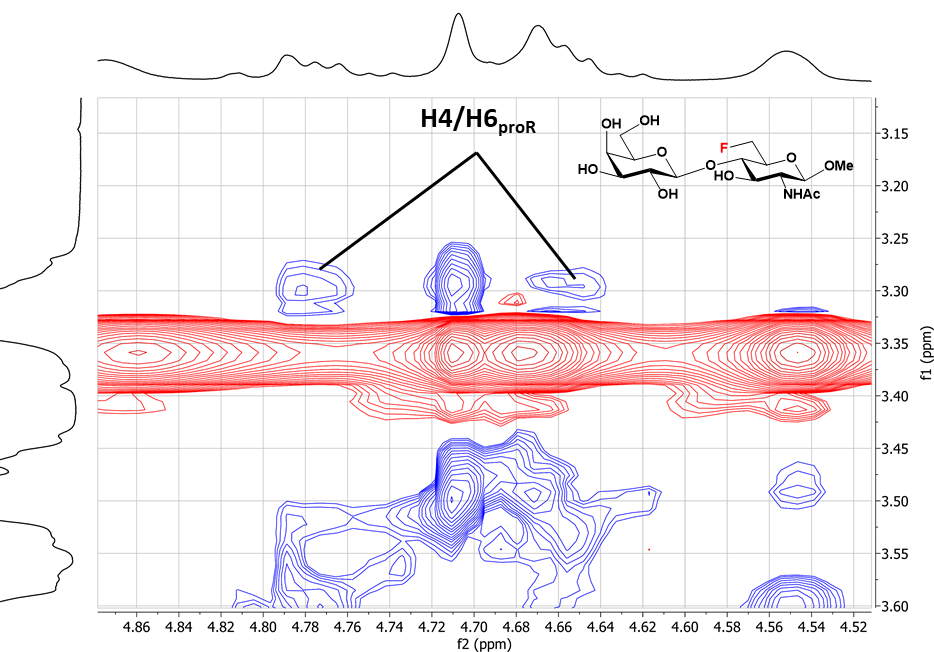
^

## ^1^H NMR (400 MHz, DMSO-*d*_6_) 6F-LN **4**

^
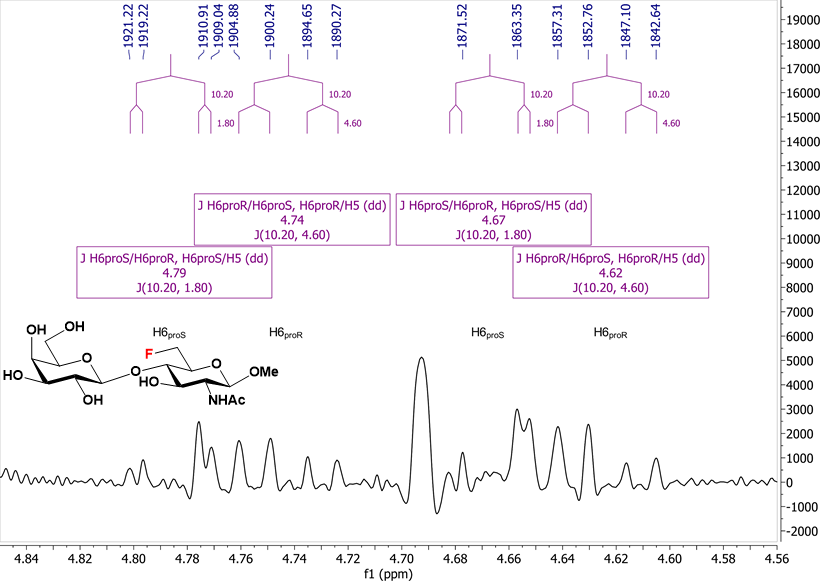
^

## 1D Selective Gradient ^1^H-^1^H ROESY NMR (DMSO-*d*_6_) 6F-LN **4**

(irradiation frequency: 4.144 ppm, Bruker Avance III™ HD 850 MHz)

^
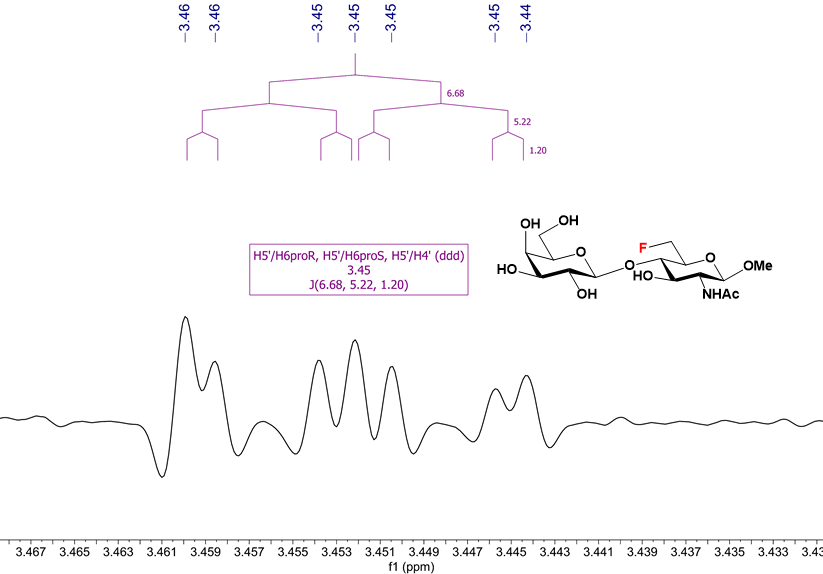
^

## Temperature Dependent ^1^H-NMR (DMSO-*d*_6_) 6F-LN **4**


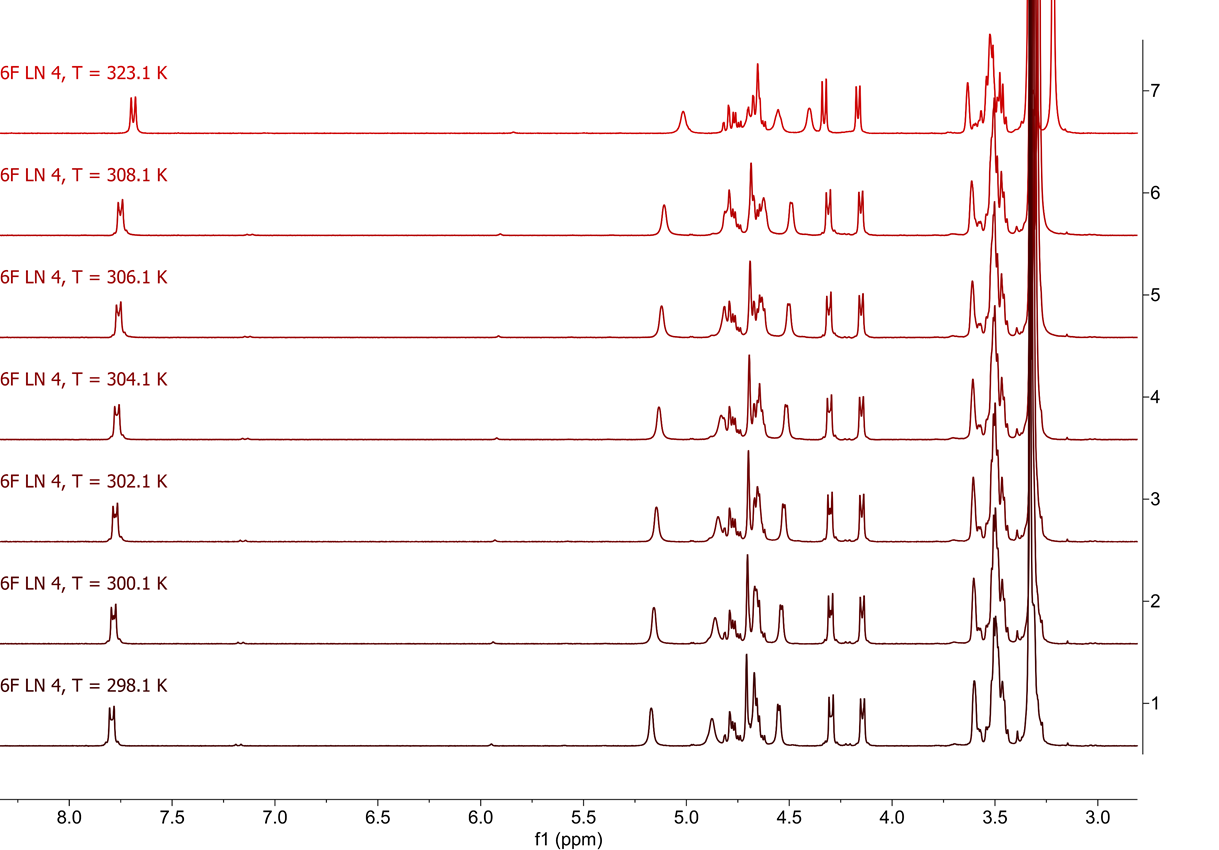


## ^1^H NMR (400 MHz, DMSO-*d*_6_) 2′F-LN **5**

**
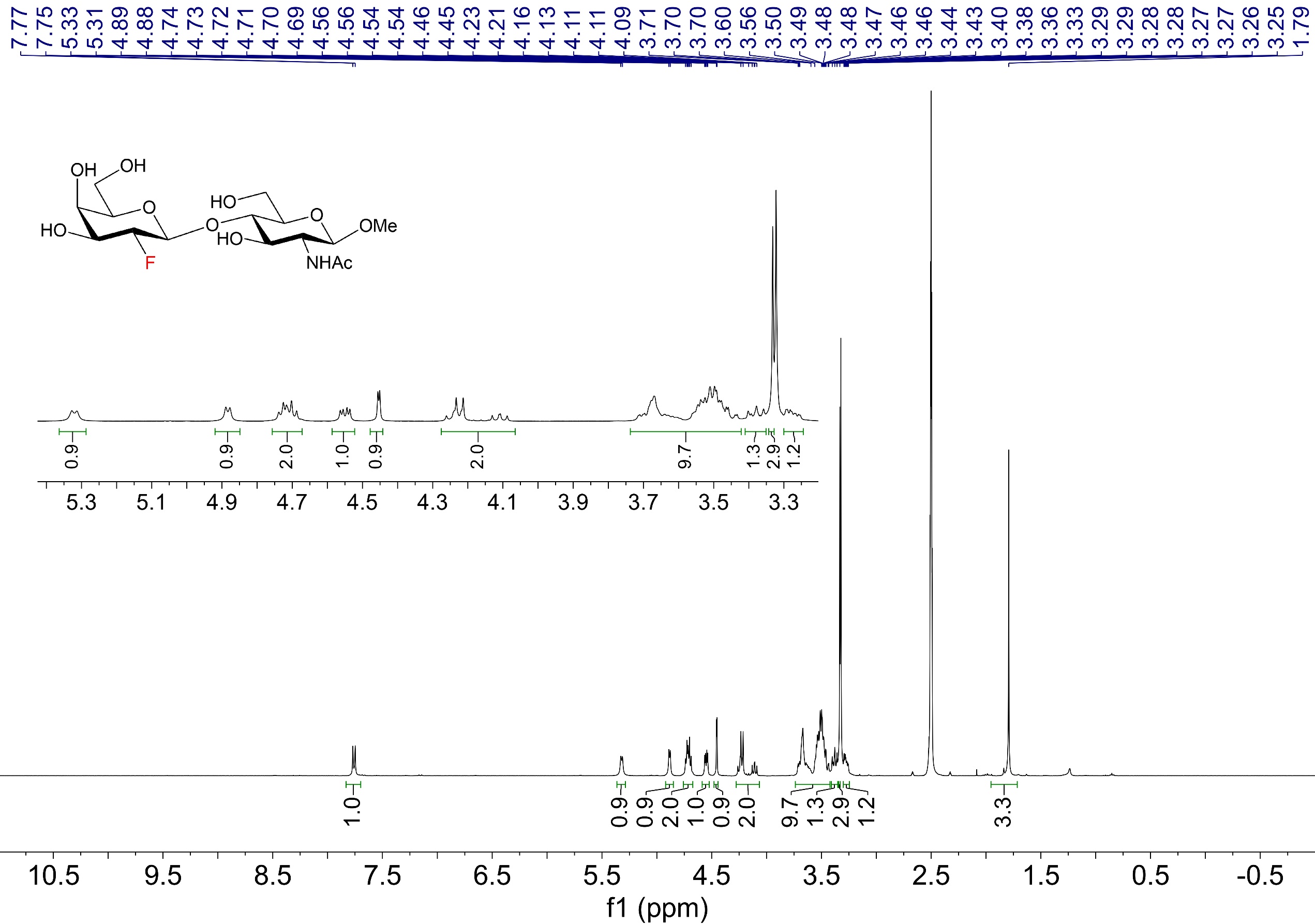
**

## ^13^C{^1^H} NMR (101 MHz, DMSO-*d*_6_) 2′F-LN **5**


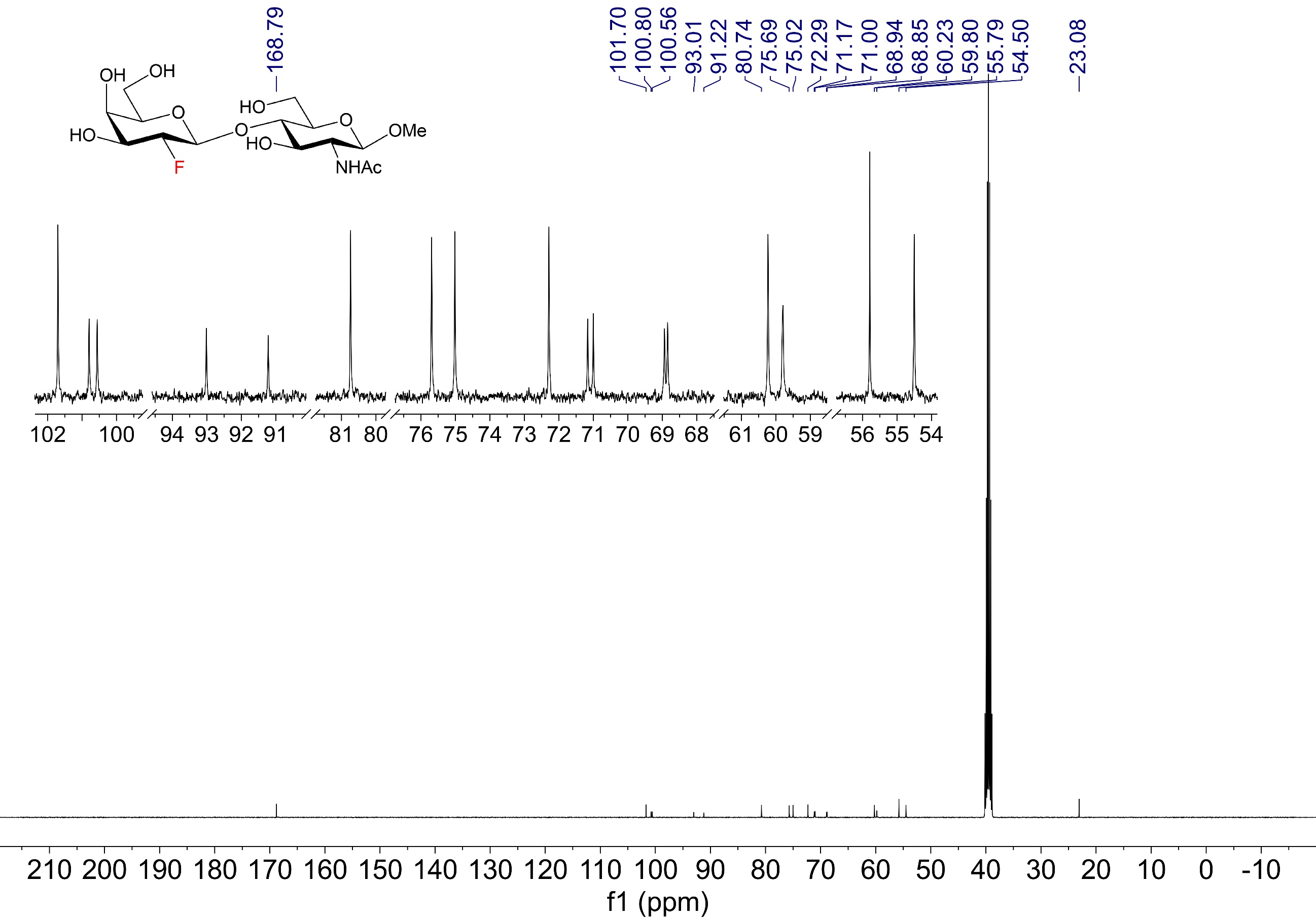


## ^19^F NMR (376 MHz, DMSO-*d*_6_) 2′F-LN **5**

**
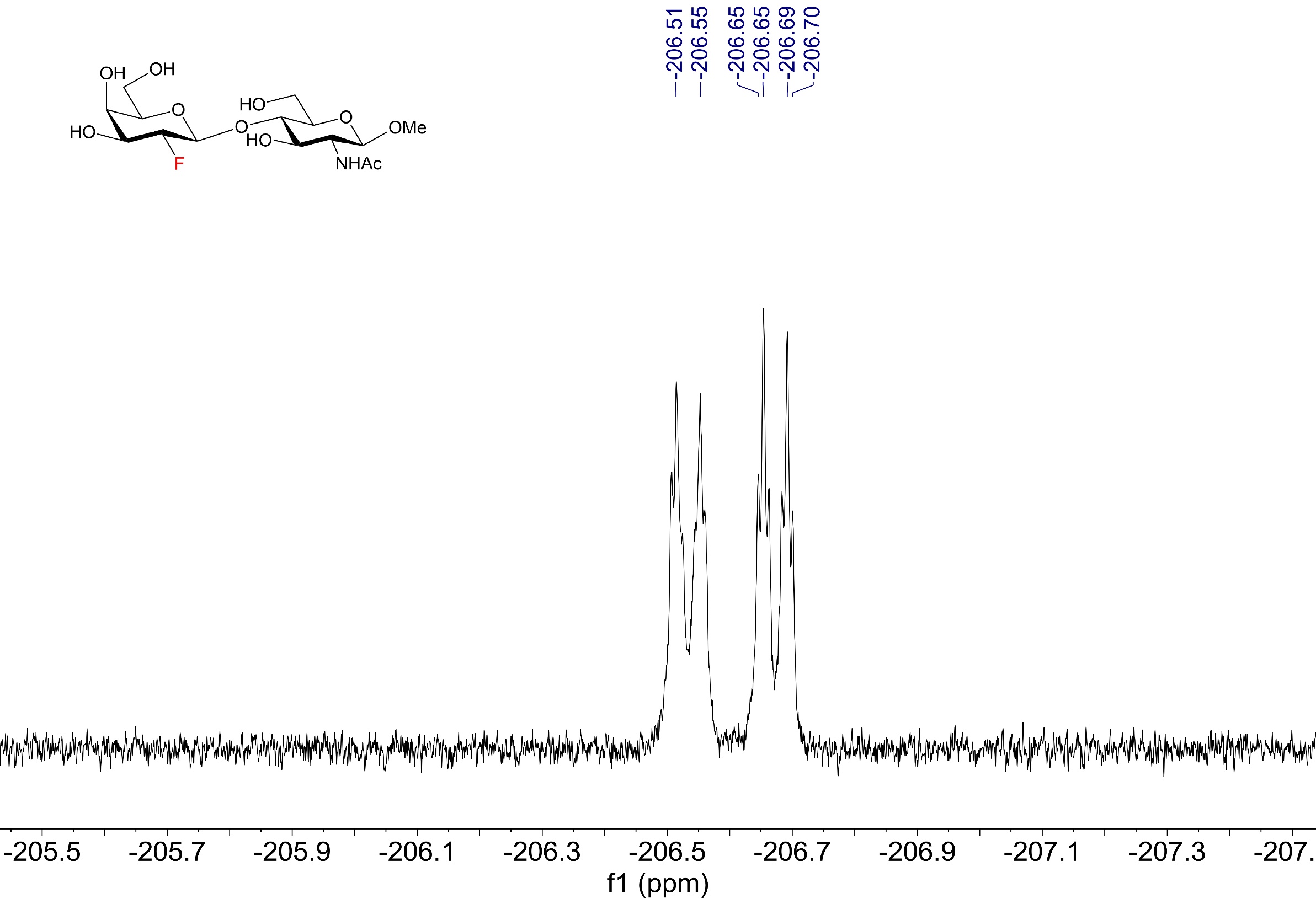
**

## ^1^H-^1^H COSY NMR (DMSO-*d*_6_) 2′F-LN **5**


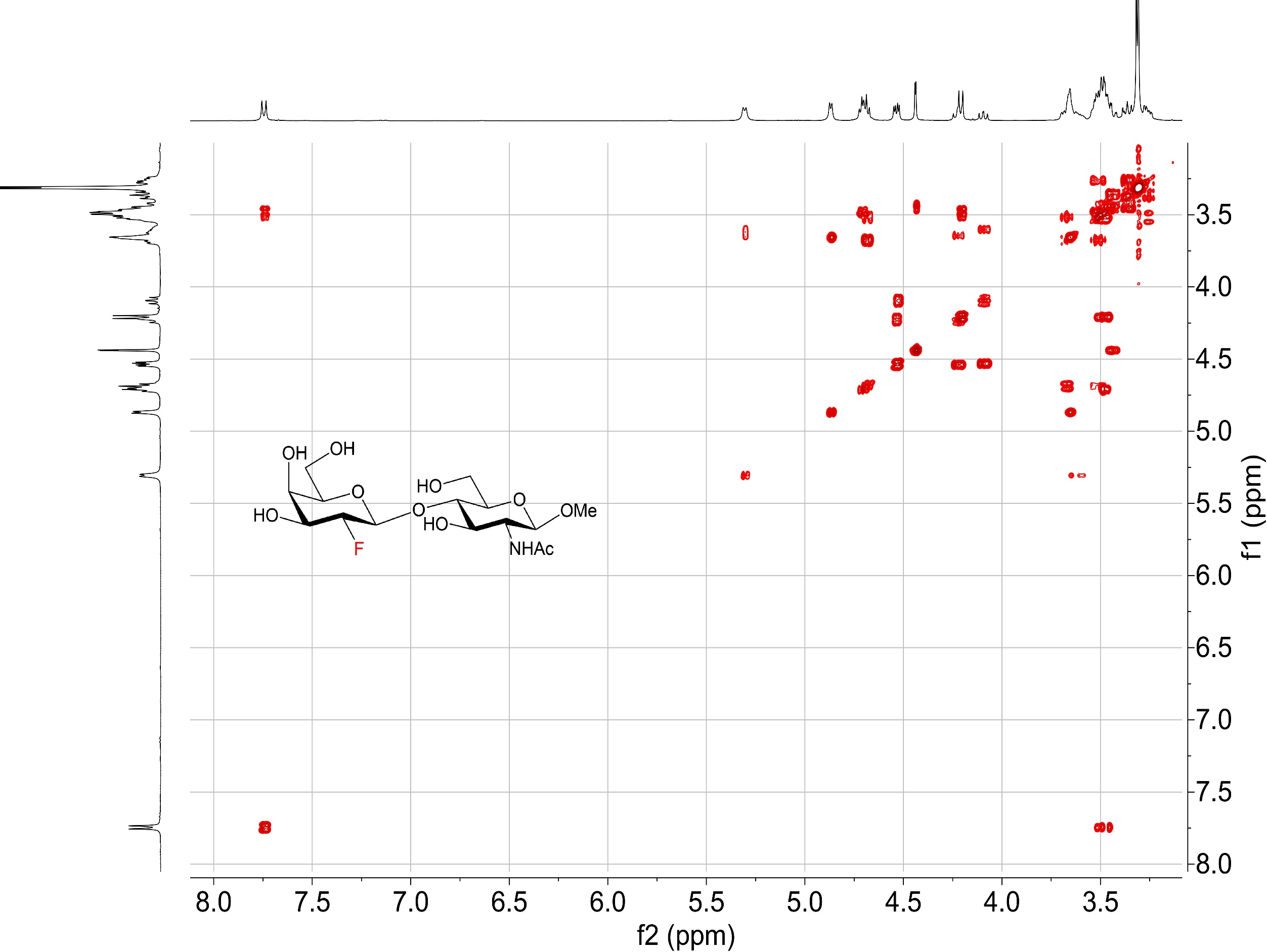


## ^1^H-^13^C HSQC NMR (DMSO-*d*_6_) 2′F-LN **5**

**
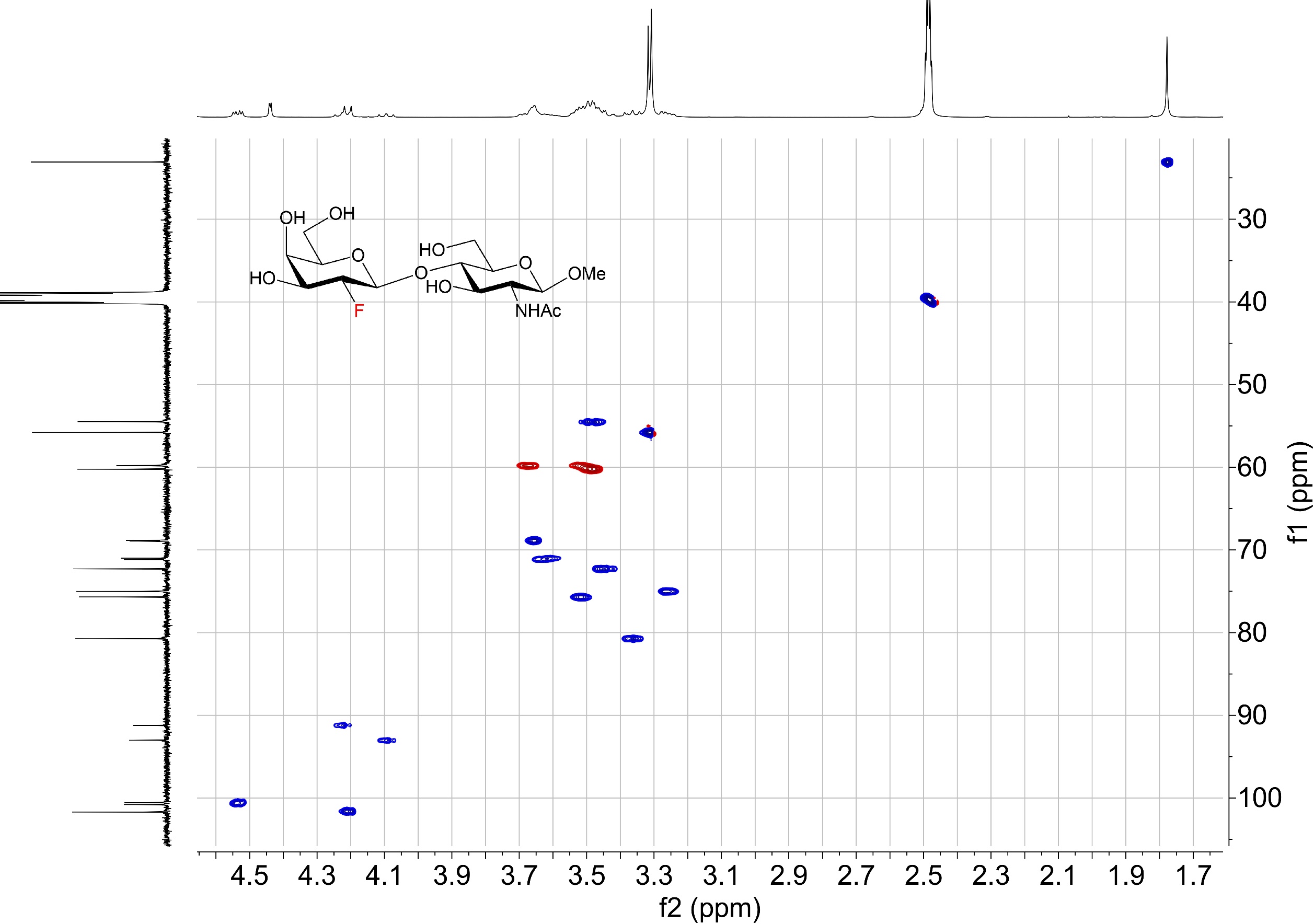
**

## ^1^H-^13^C HMBC NMR (DMSO-*d*_6_) 2′F-LN **5**


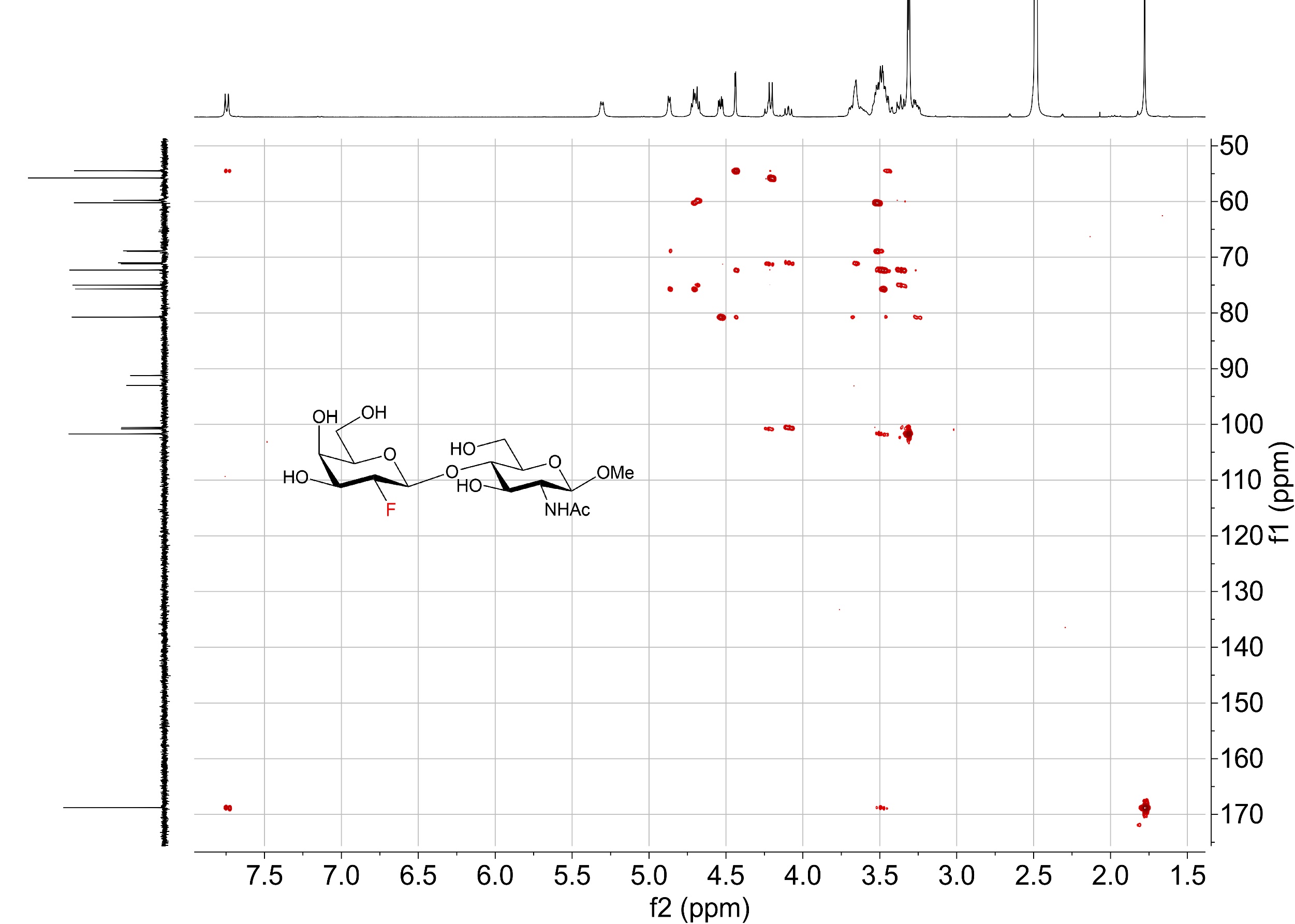


## ^1^H-^1^H ROESY NMR (DMSO-*d*_6_) 2′F-LN **5**


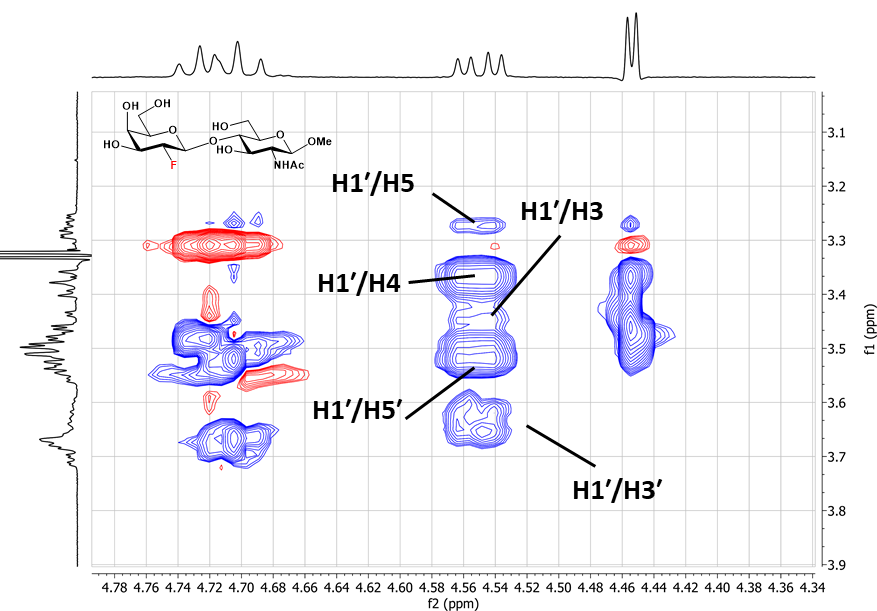


## ^1^H-^1^H ROESY NMR (DMSO-*d*_6_) 2′F-LN **5**

^
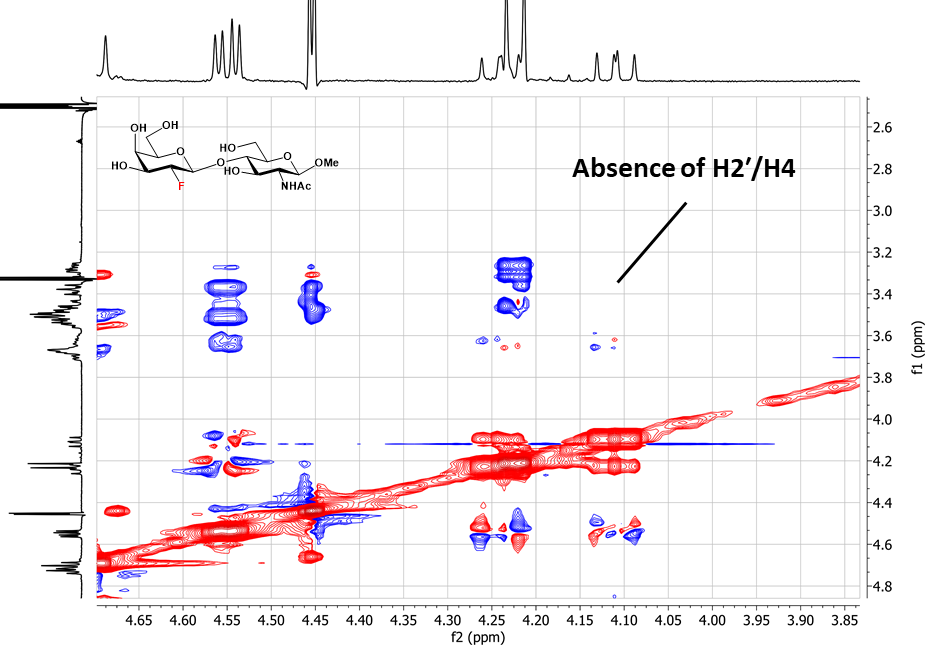
^

## ^1^H-^1^H ROESY NMR (DMSO-*d*_6_) 2′F-LN **5**

^
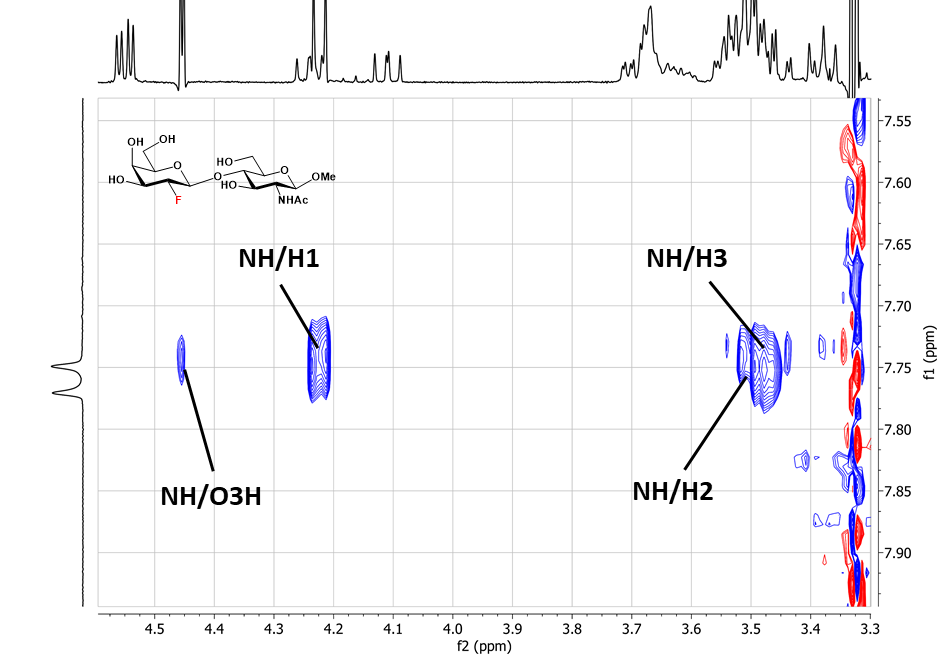
^

## ^1^H-^1^H ROESY NMR (DMSO-*d*_6_) 2′F-LN **5**

^
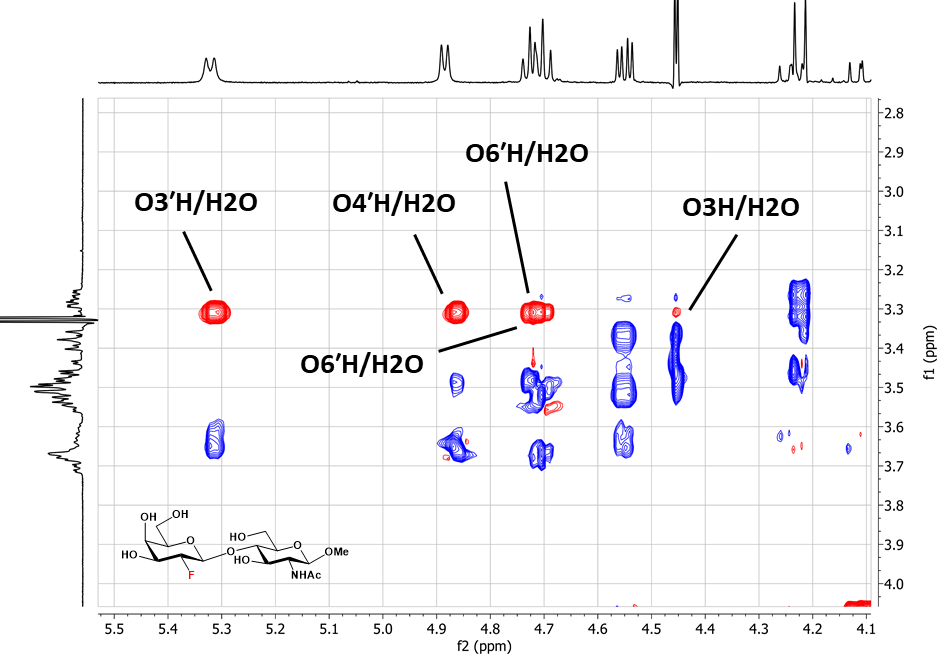
^

## ^1^H-^1^H ROESY NMR (DMSO-*d*_6_) 2′F-LN **5**

^
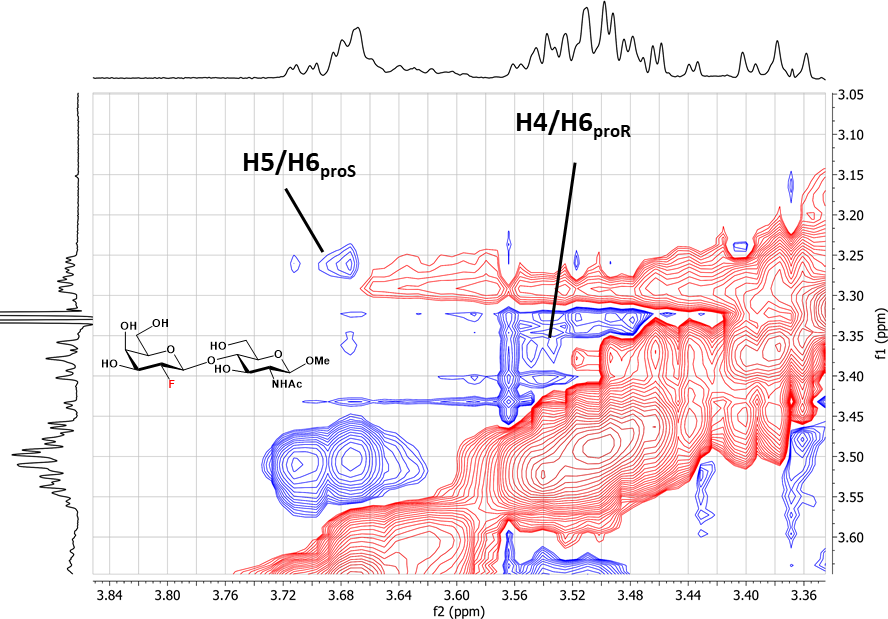
^

## ^1^H NMR (DMSO-*d*_6_) 2′F-LN **5**

^
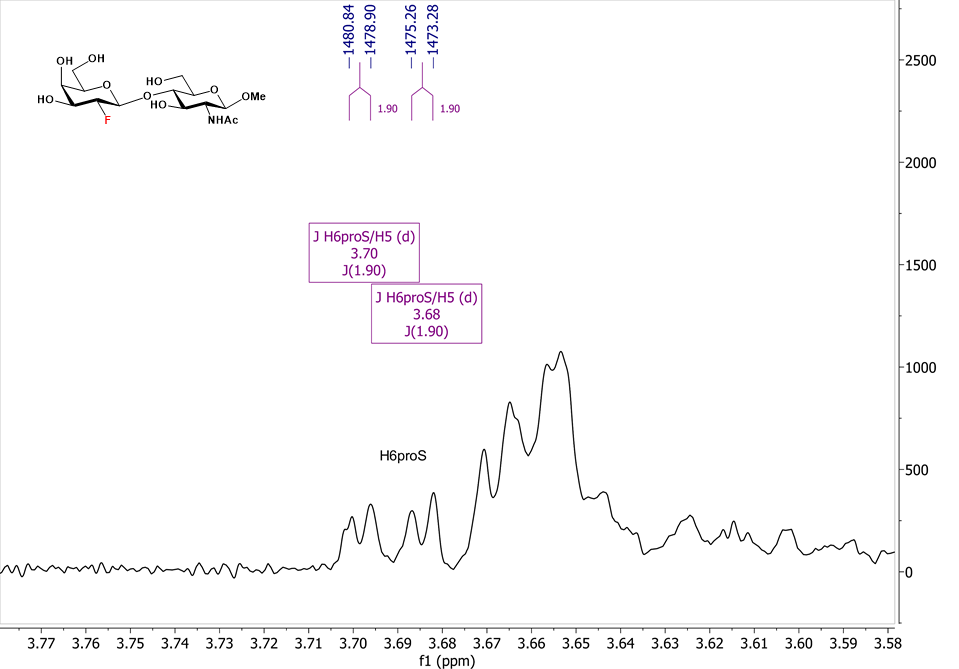
^

## ^1^H NMR (DMSO-*d*_6_) 2′F-LN **5**

^
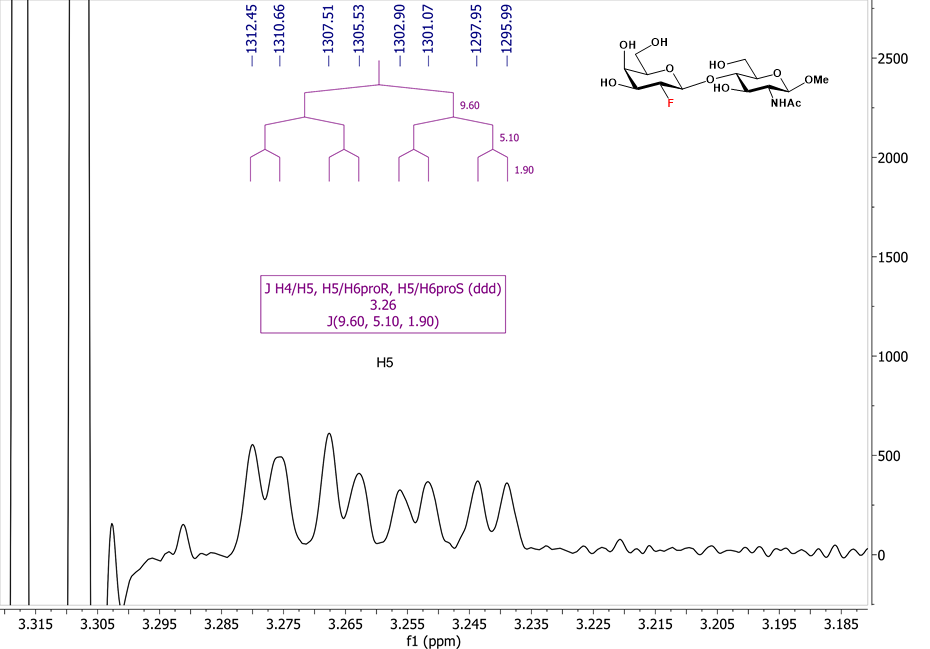
^

## Selective Gradient ^1^H-^1^H ROESY NMR (DMSO-*d*_6_) 2′F-LN **5**

(irradiation frequency: 4.557 ppm)

^
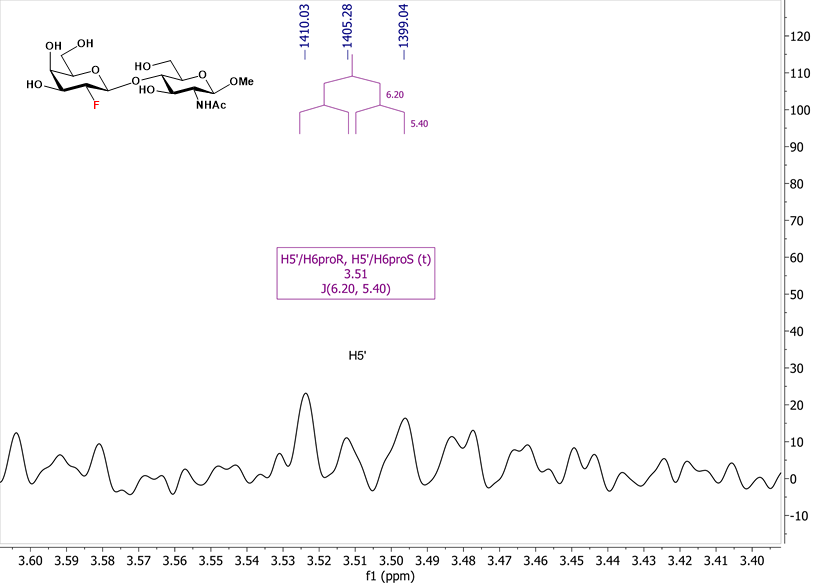
^

## Temperature Dependent ^1^H-NMR (DMSO-*d*_6_) 2′F-LN **5**

^
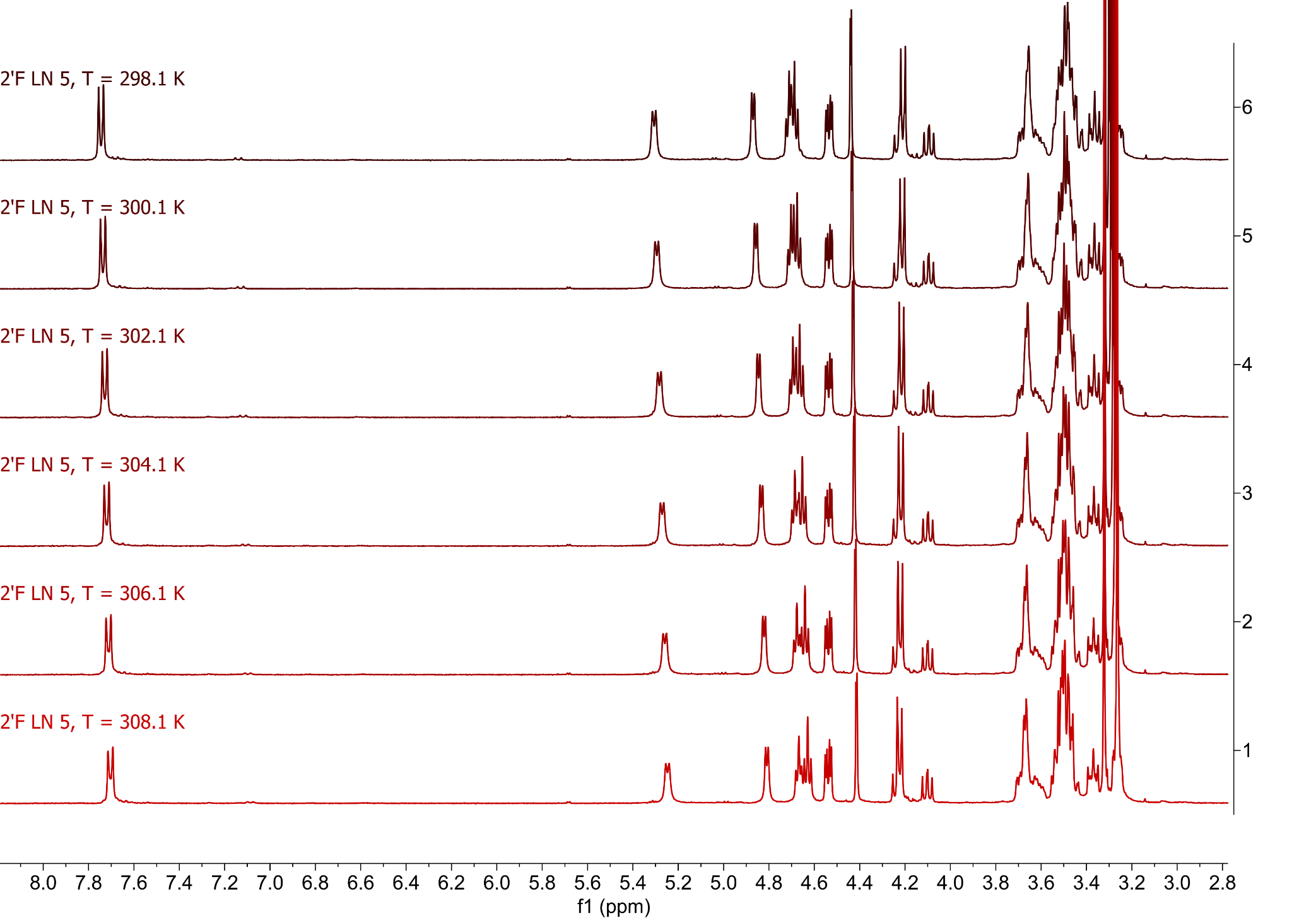
^

## ^1^H NMR (400 MHz, DMSO-*d*_6_) 3′F-LN **6**

**
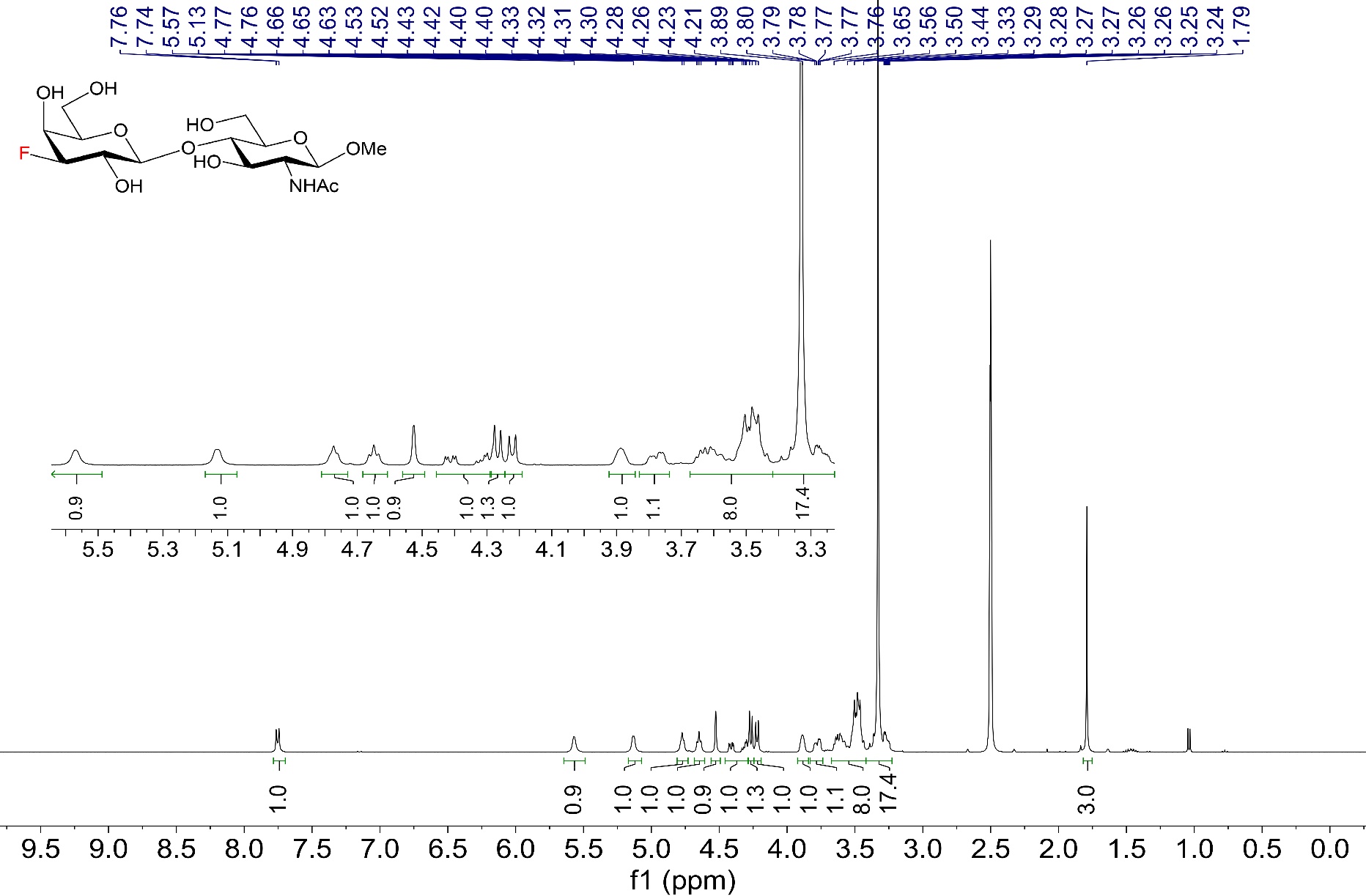
**

## ^13^C{^1^H} APT NMR (126 MHz, DMSO-*d*_6_) 3′F-LN **6**

**
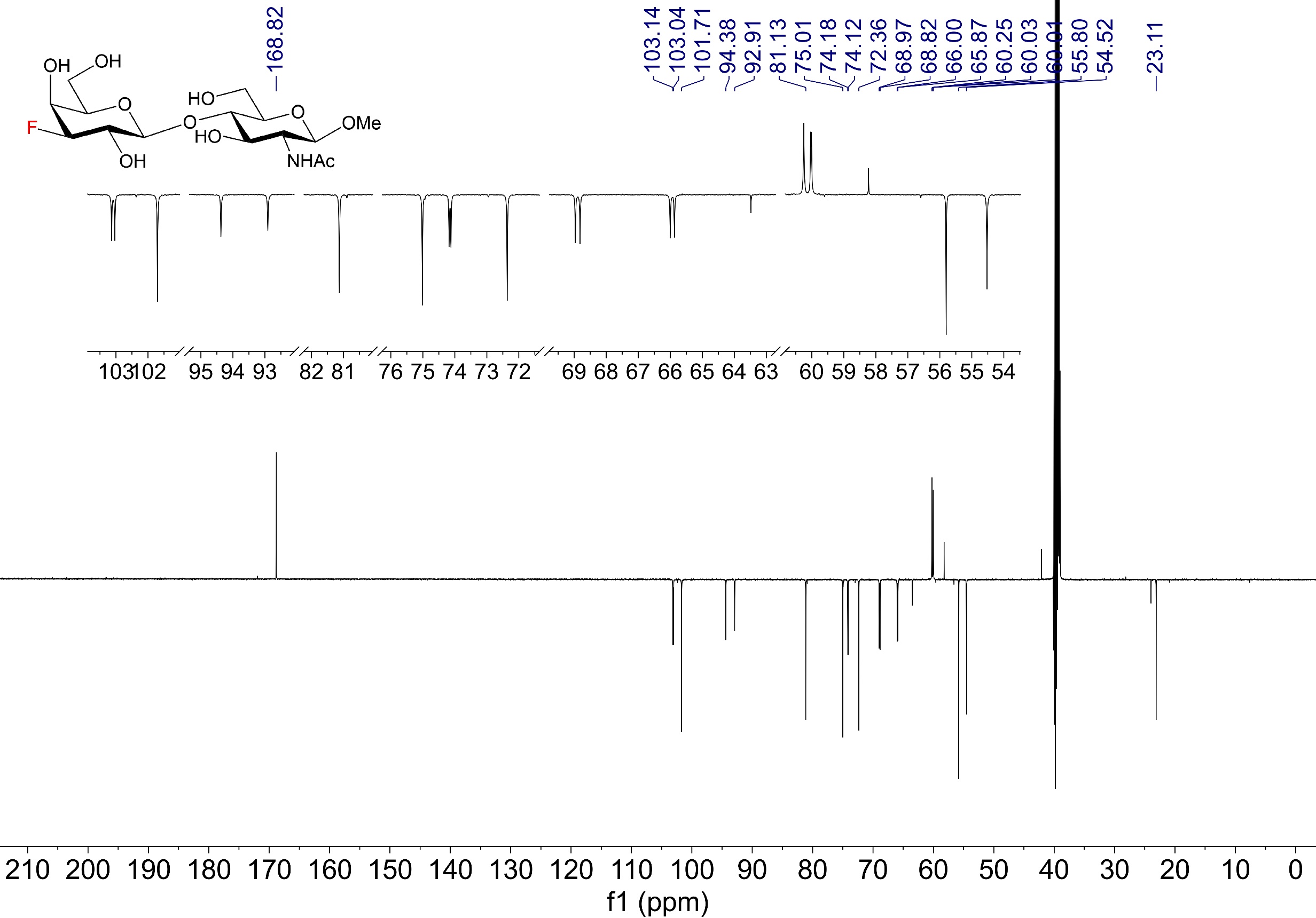
**

## ^19^F NMR (376 MHz, DMSO-*d*_6_) 3′F-LN **6**

**
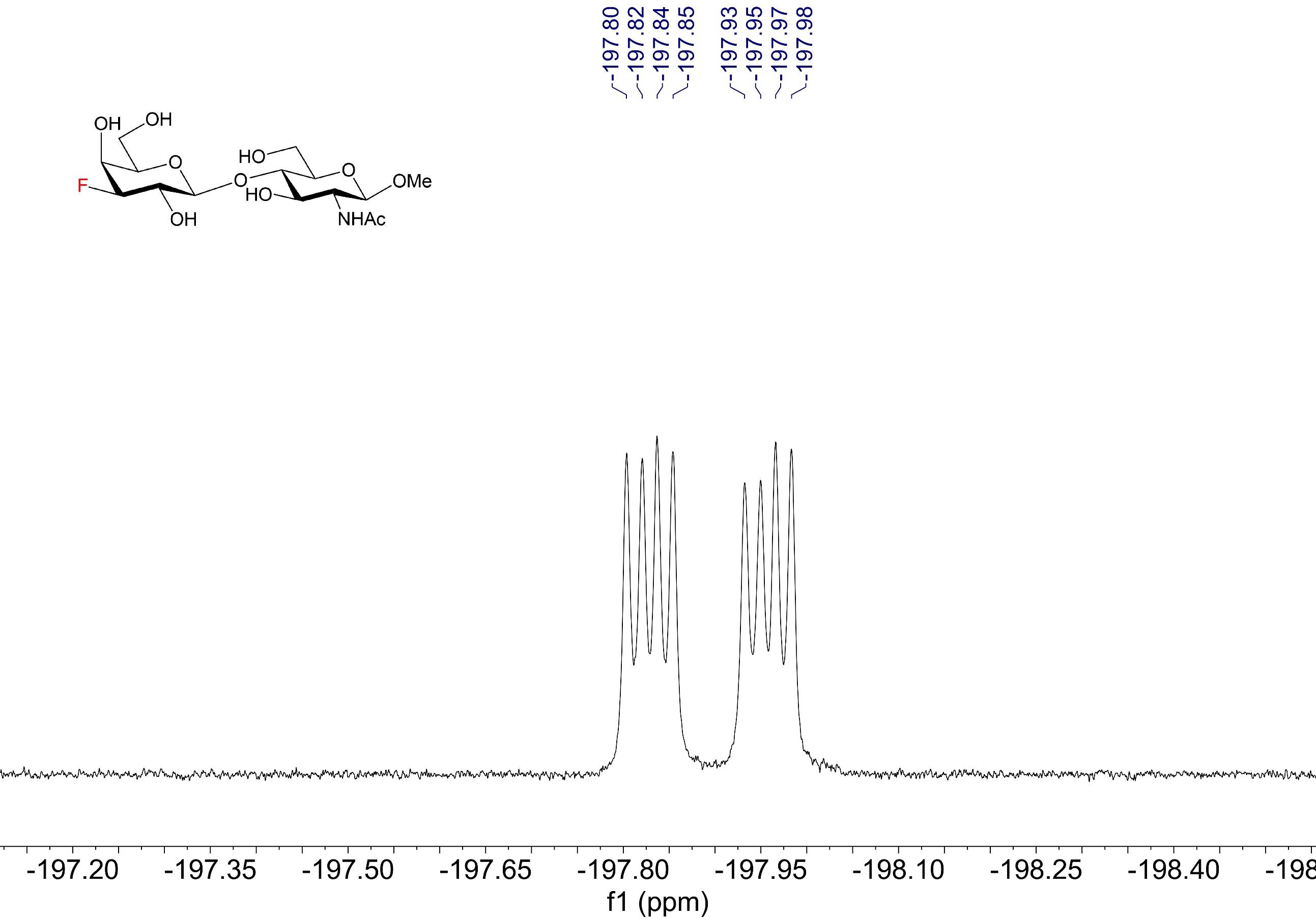
**

## ^1^H-^1^H COSY NMR (DMSO-*d*_6_) 3′F-LN **6**


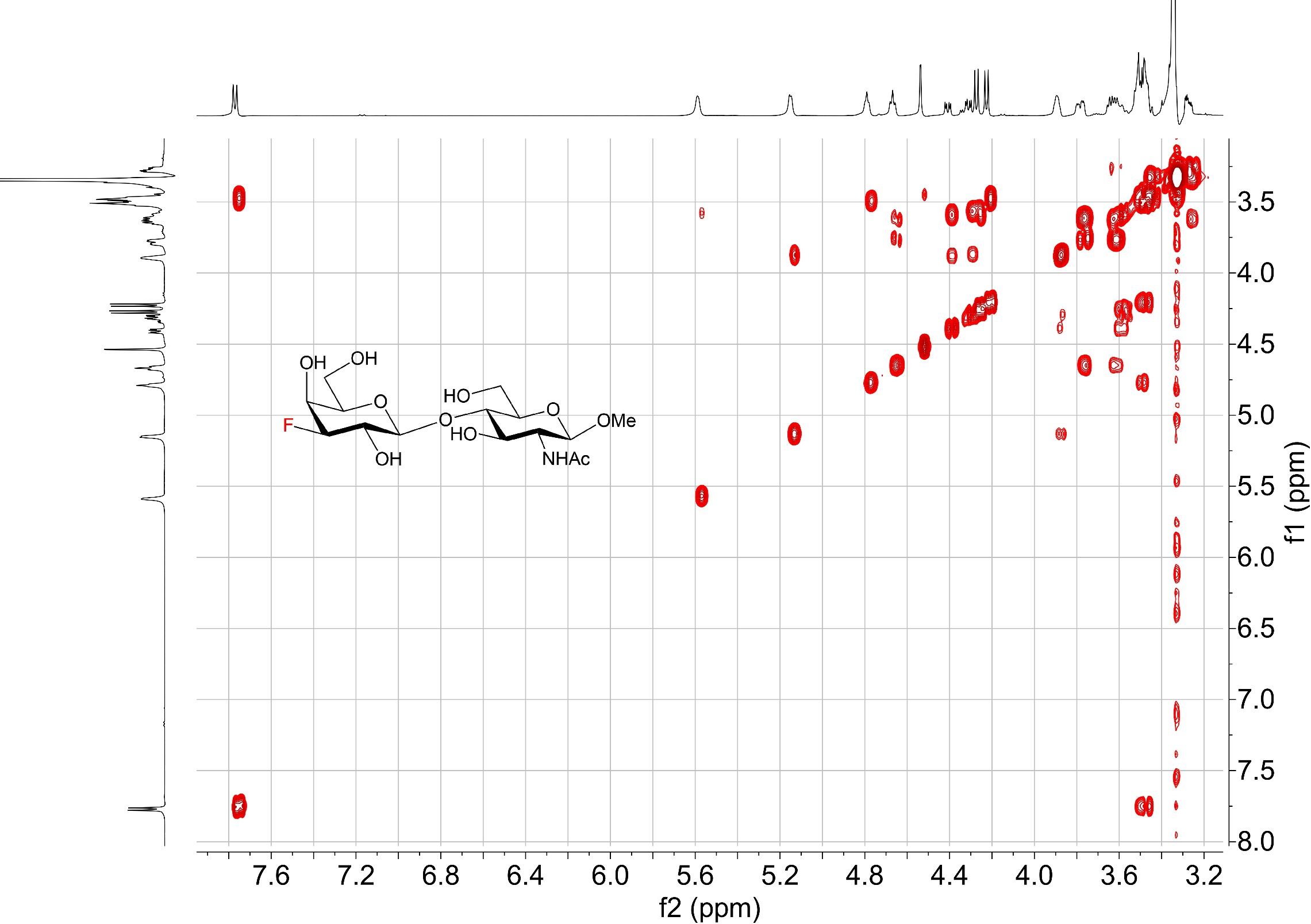


## ^1^H-^13^C HSQC NMR (DMSO-*d*_6_) 3′F-LN **6**

**
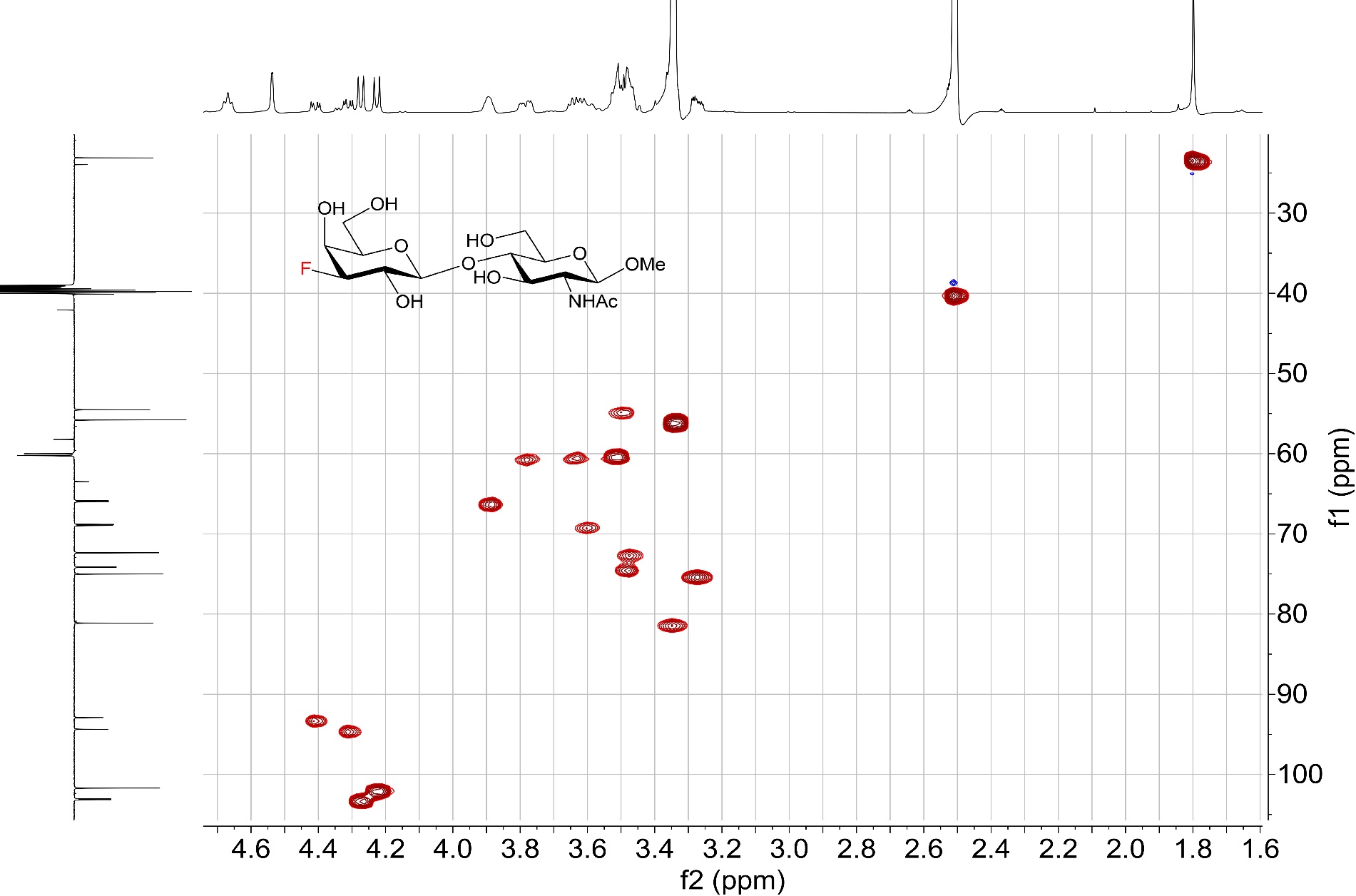
**

## ^1^H-^13^C HMBC NMR (DMSO-*d*_6_) 3′F-LN **6**


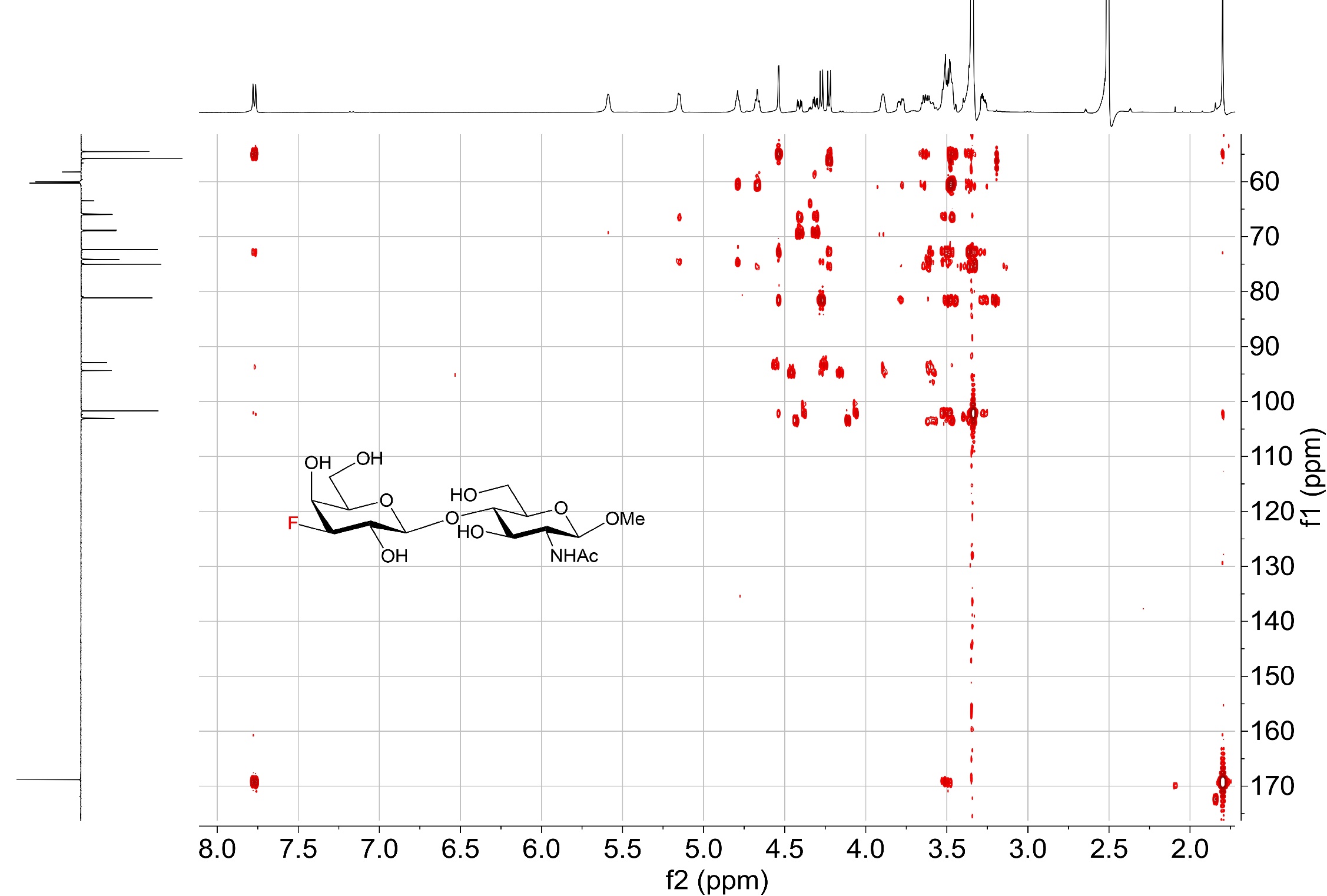


## ^1^H-^1^H ROESY NMR (DMSO-*d*_6_) 3′F-LN **6**

^
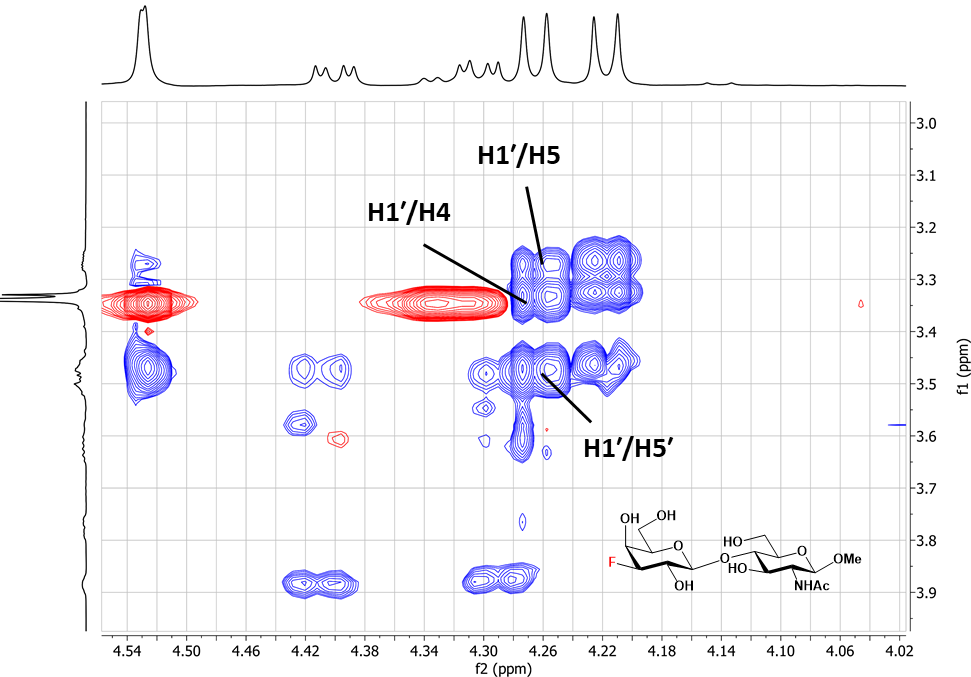
^

## ^1^H-^1^H ROESY NMR (DMSO-*d*_6_) 3′F-LN **6**

^
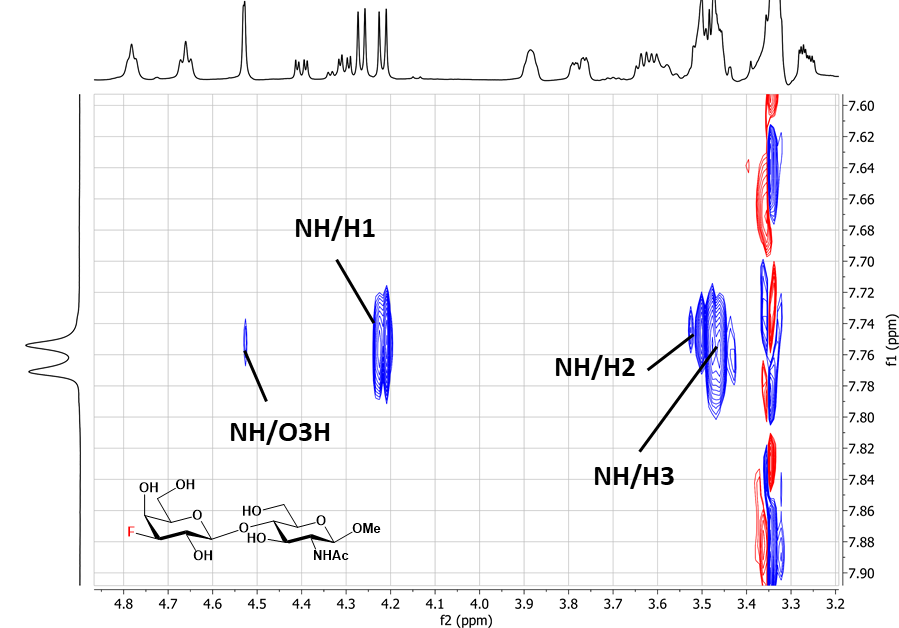
^

## ^1^H-^1^H ROESY NMR (DMSO-*d*_6_) 3′F-LN **6**

^
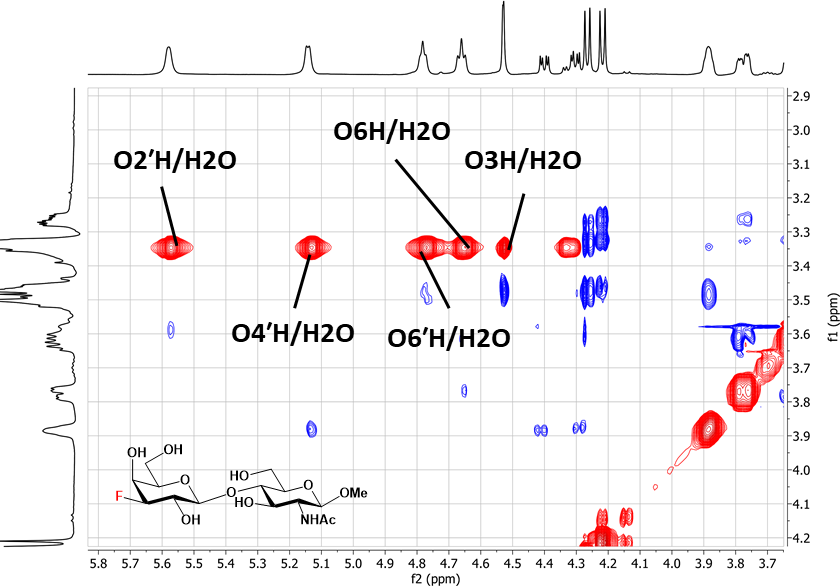
^

## ^1^H-^1^H ROESY NMR (DMSO-*d*_6_) 3′F-LN **6**

^
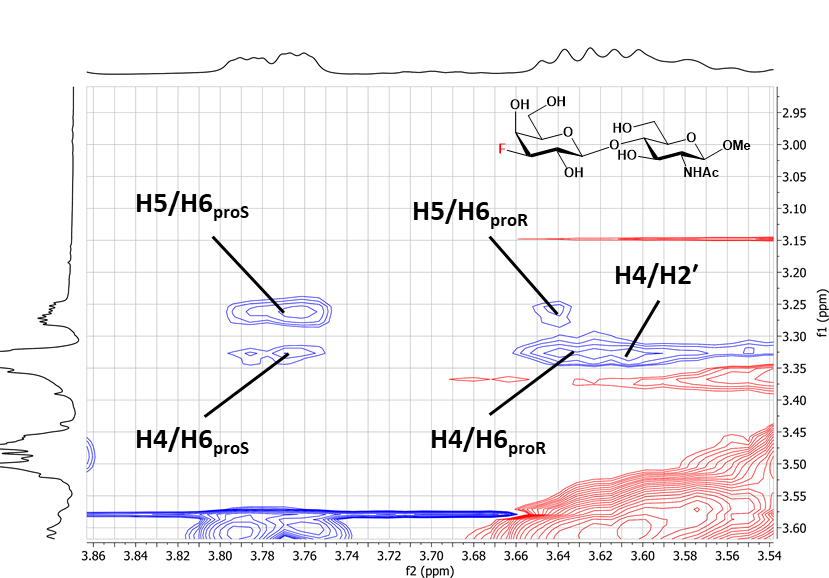
^

## Selective Homonuclear Decoupled ^1^H-NMR (DMSO-*d*_6_) 3′F-LN **6**

(irradiation frequency: 4.660 ppm)

^
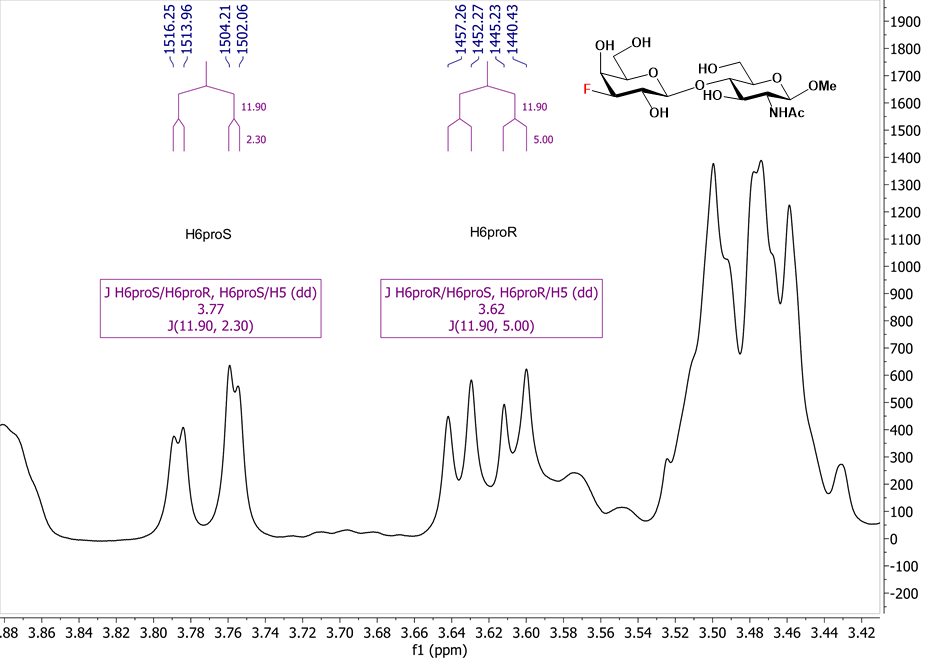
^

## 1D Selective Gradient ^1^H-^1^H TOCSY NMR (DMSO-*d*_6_) 3′F-LN **6**

(irradiation frequency: 5.136 ppm)

**
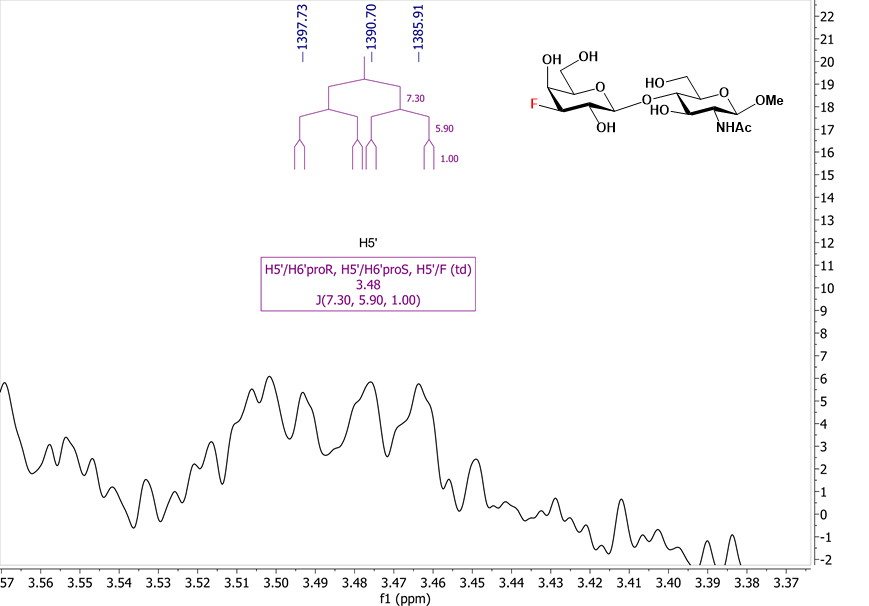
**

^
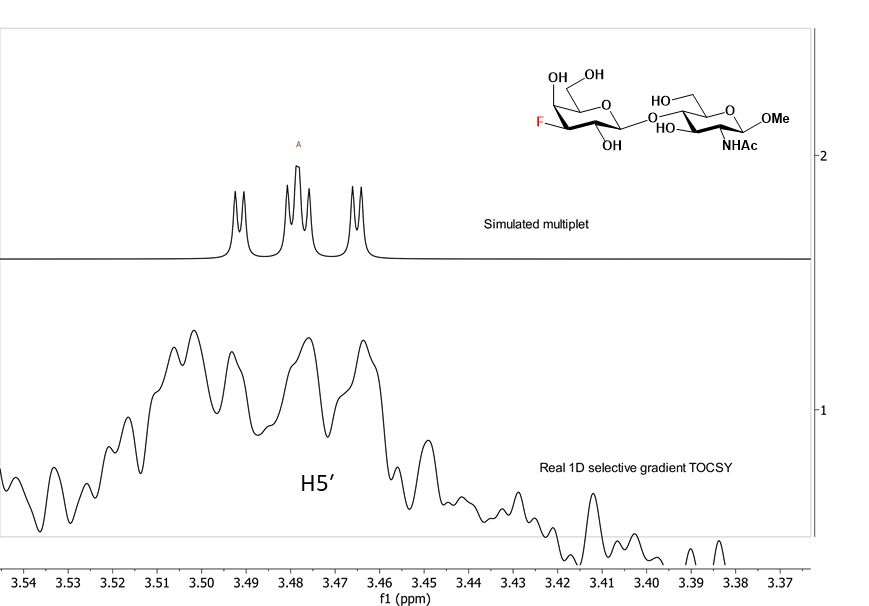
^

## Temperature Dependent ^1^H-NMR (DMSO-*d*_6_) 3′F-LN **6**

^
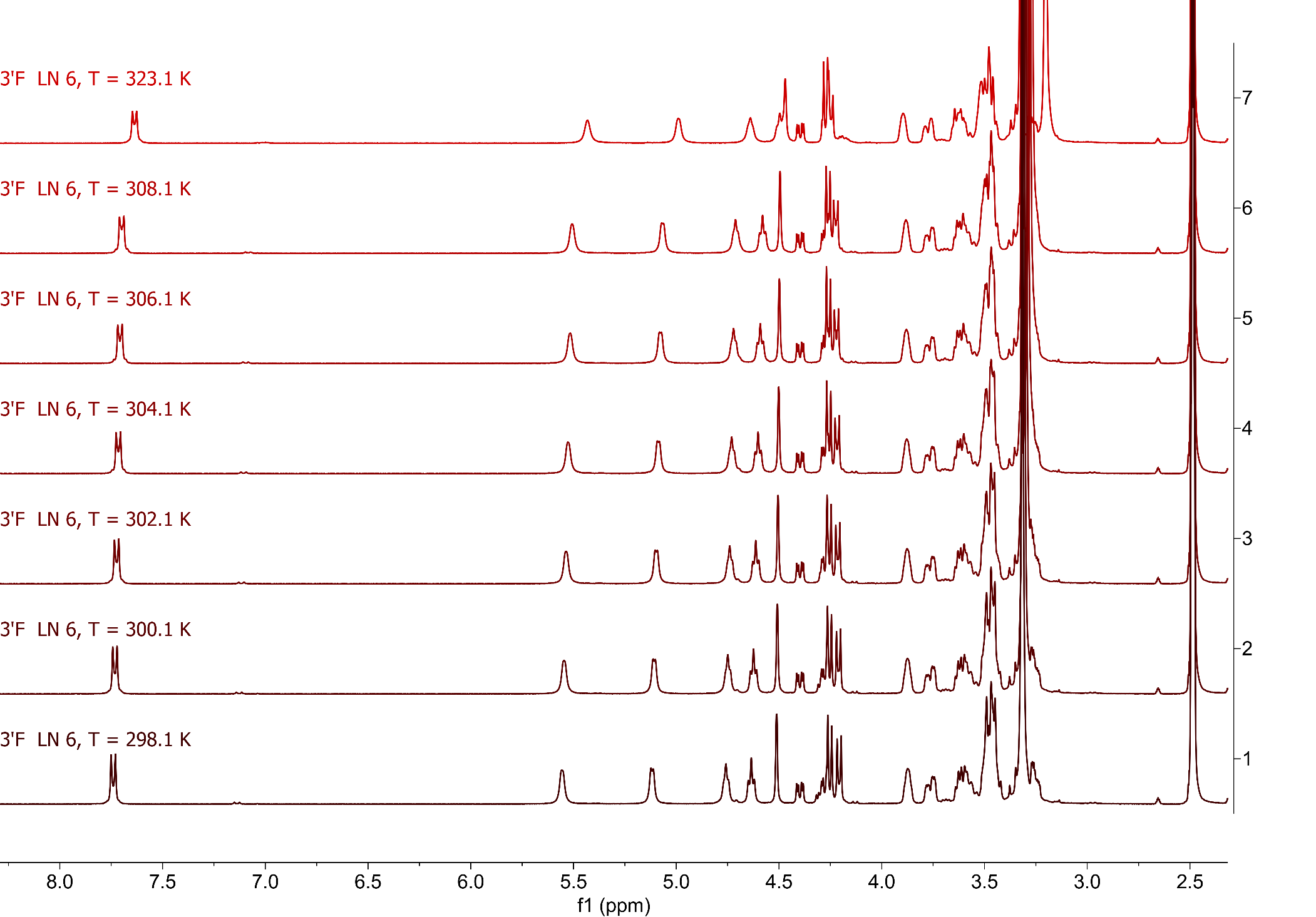
^

## ^1^H NMR (500 MHz, DMSO-*d*_6_) 4′F-LN **7**

**
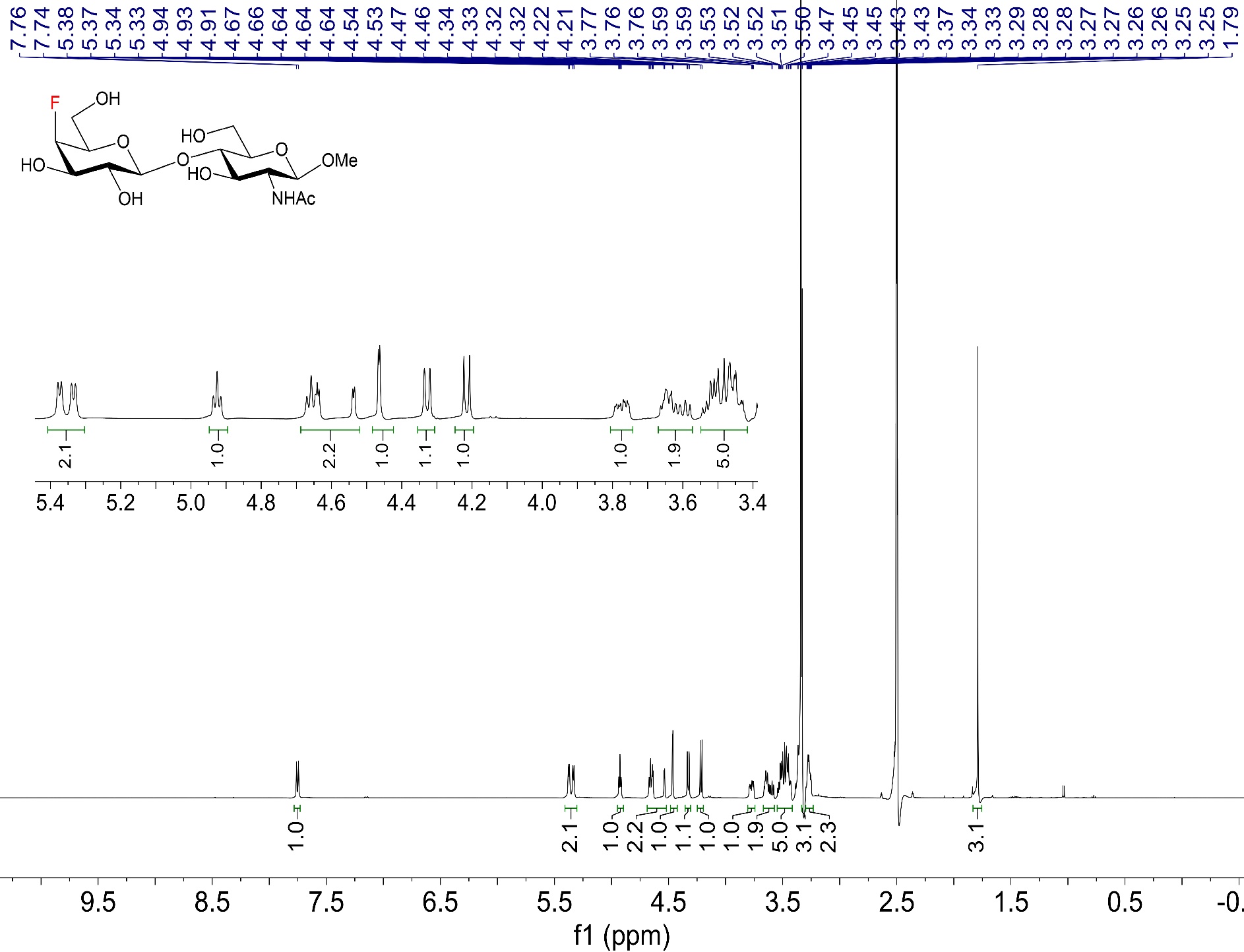
**

## ^13^C{^1^H} APT NMR (126 MHz, DMSO-*d*_6_) 4′F-LN **7**

**
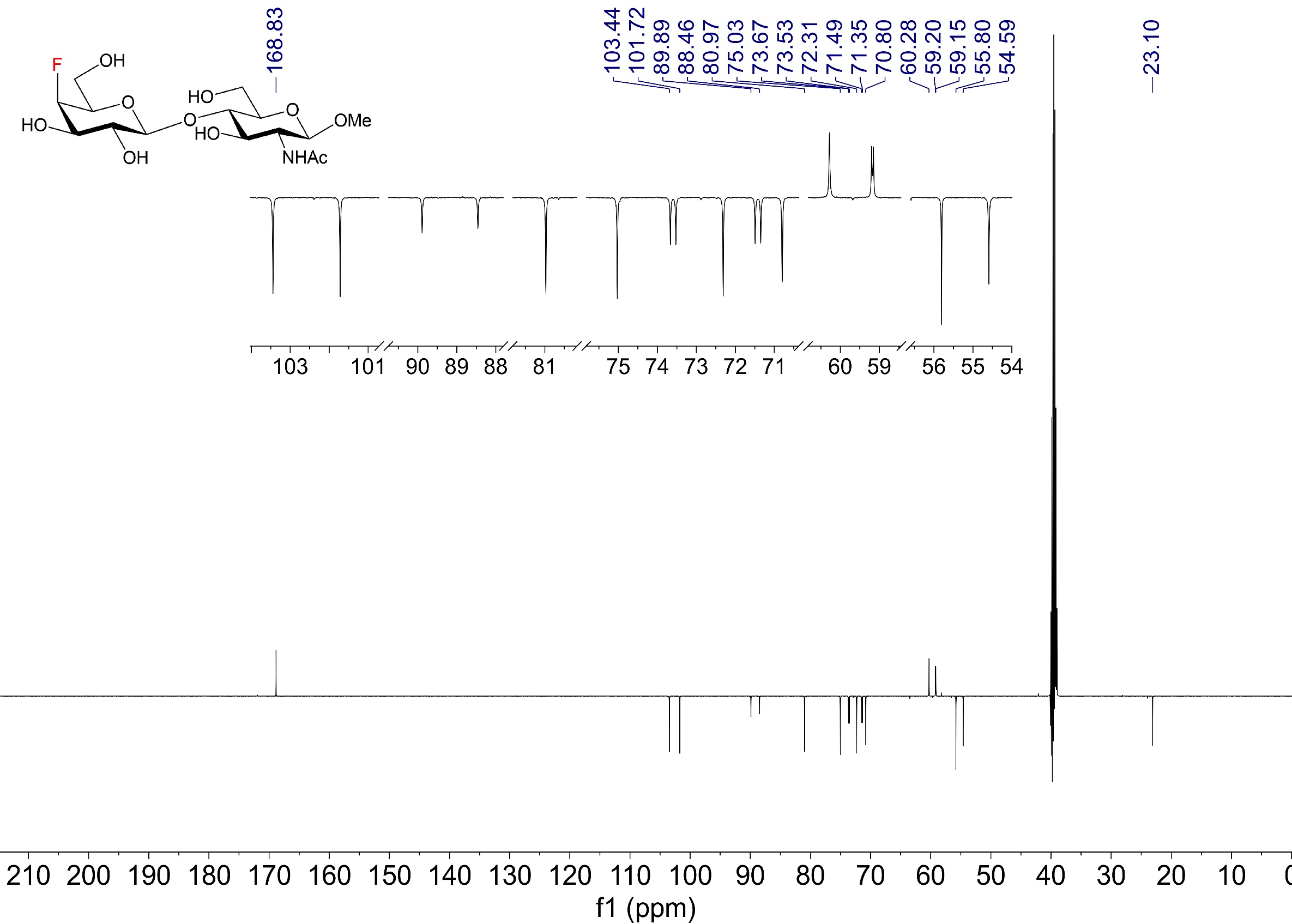
**

## ^19^F NMR (376 MHz, DMSO-*d*_6_) 4′F-LN **7**

**
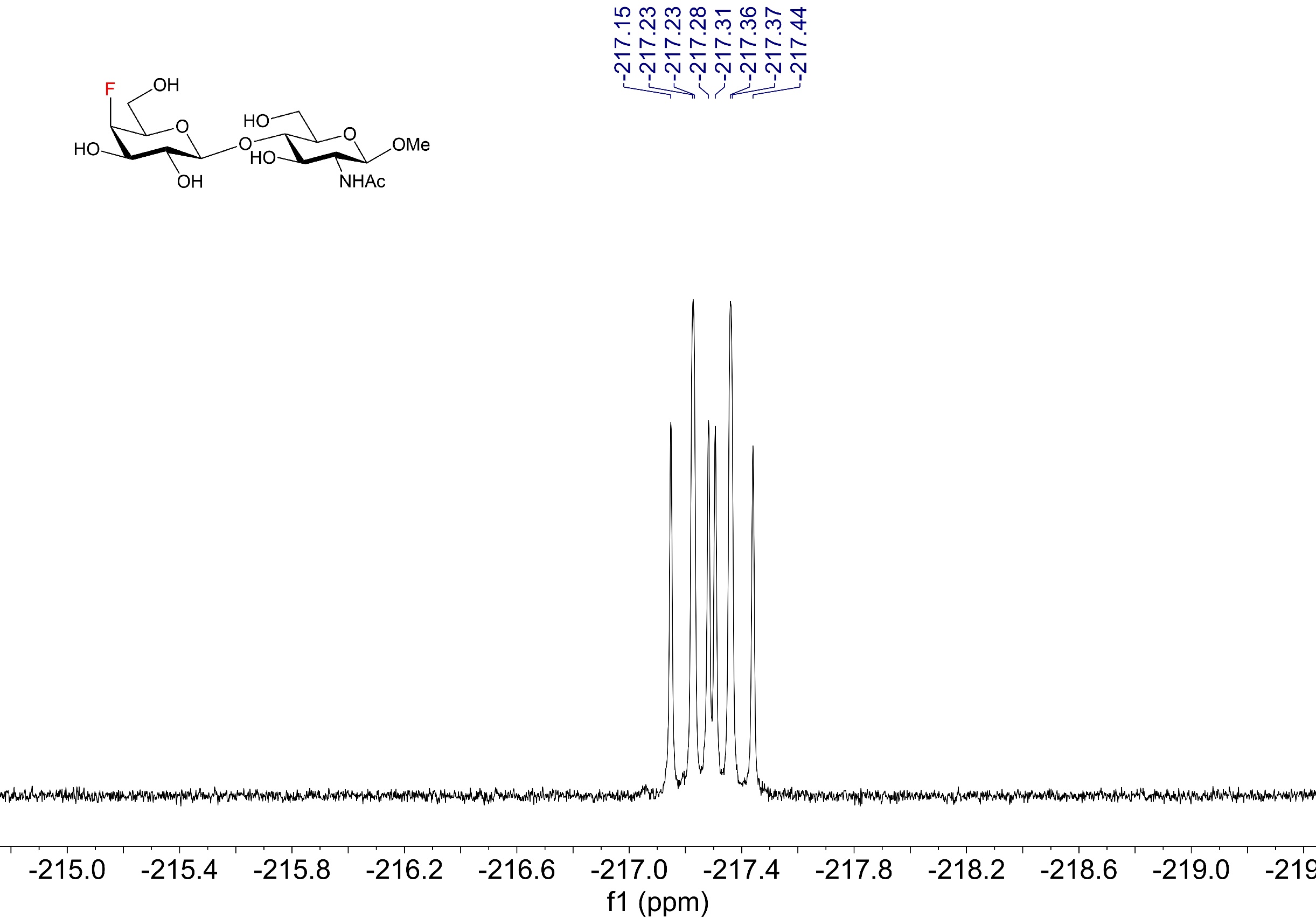
**

## ^1^H-^1^H COSY NMR (DMSO-*d*_6_) 4′F-LN **7**


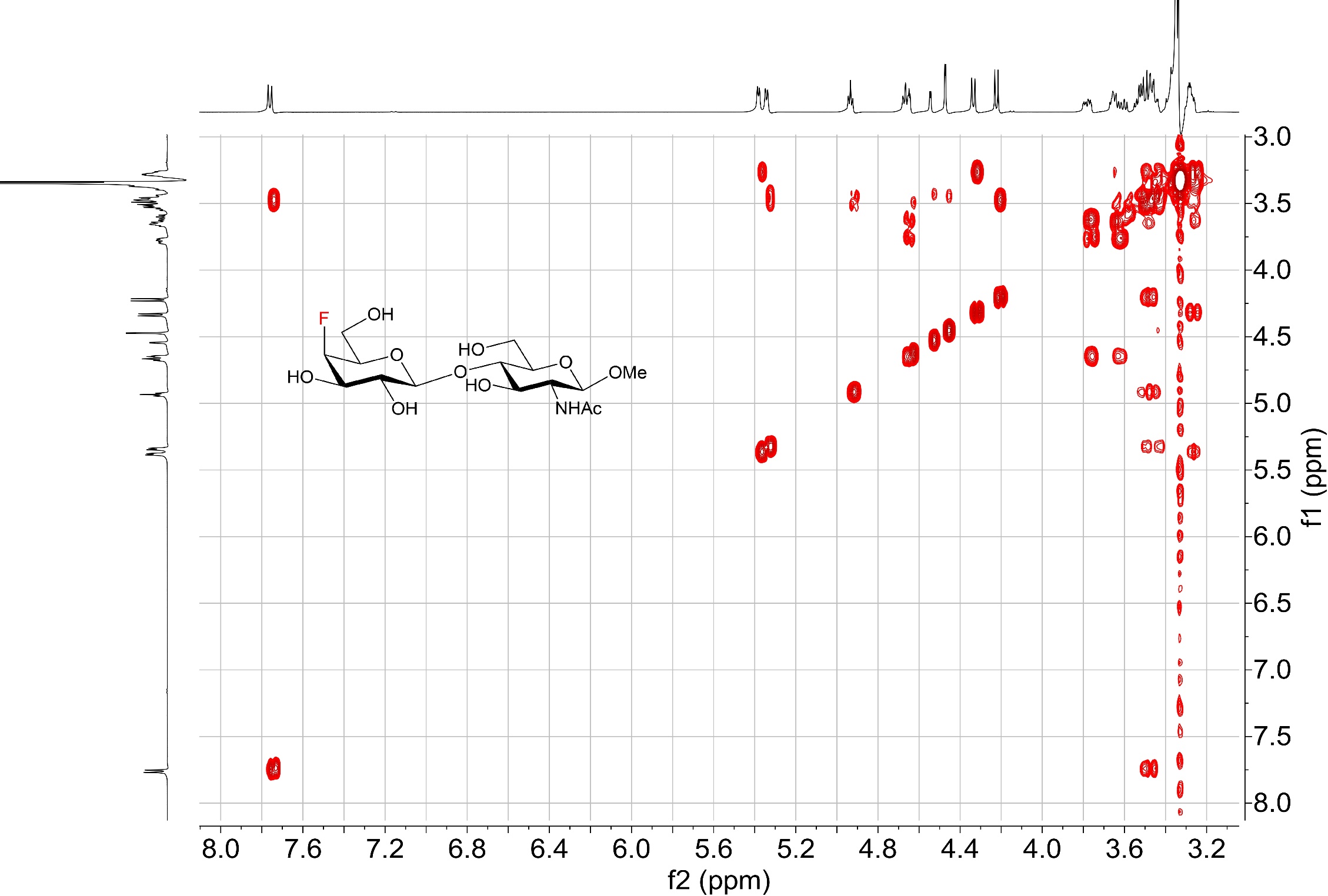


## ^1^H-^13^C HSQC NMR (DMSO-*d*_6_) 4′F-LN **7**

**
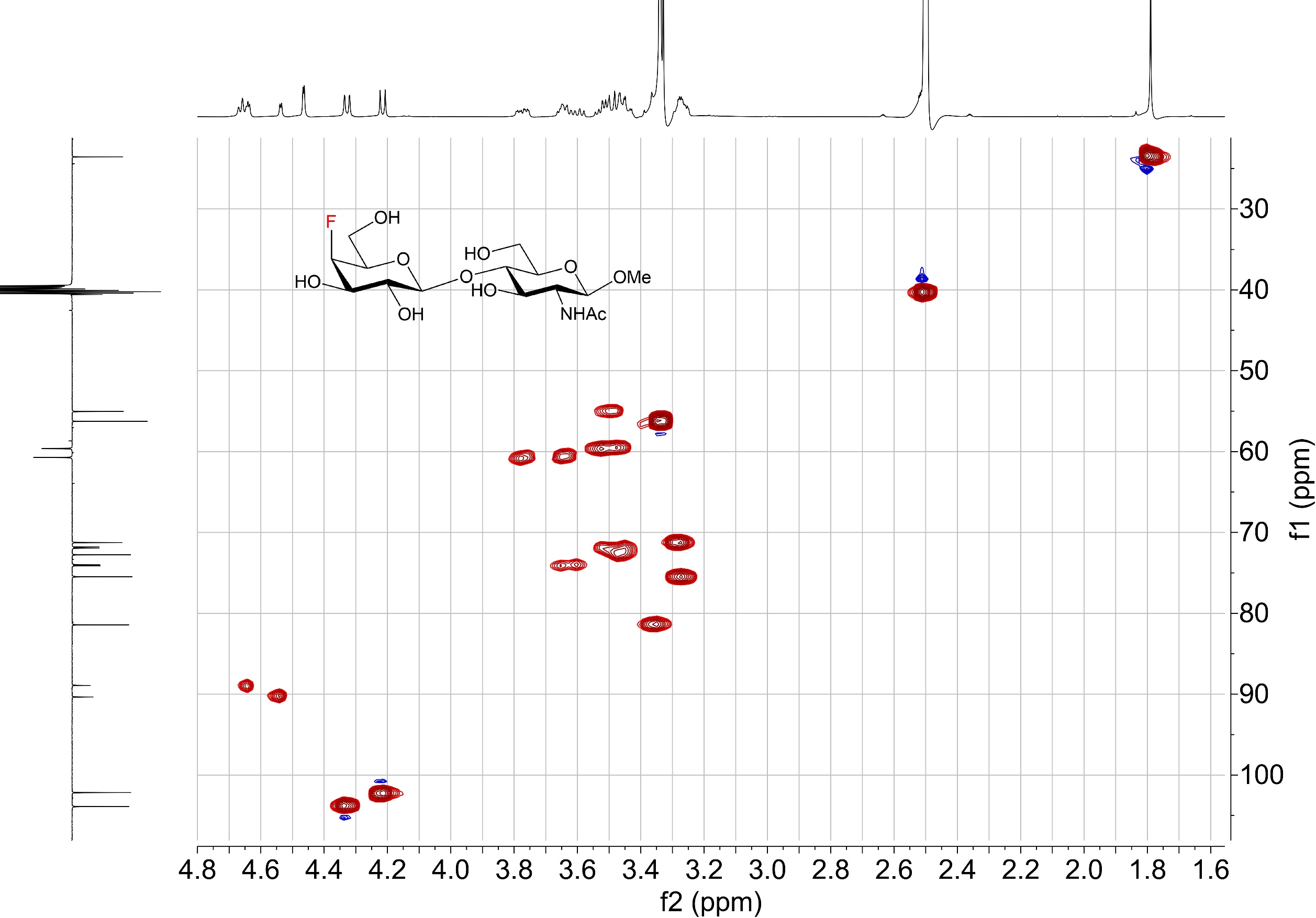
**

## ^1^H-^13^C HMBC NMR (DMSO-*d*_6_) 4′F-LN **7**


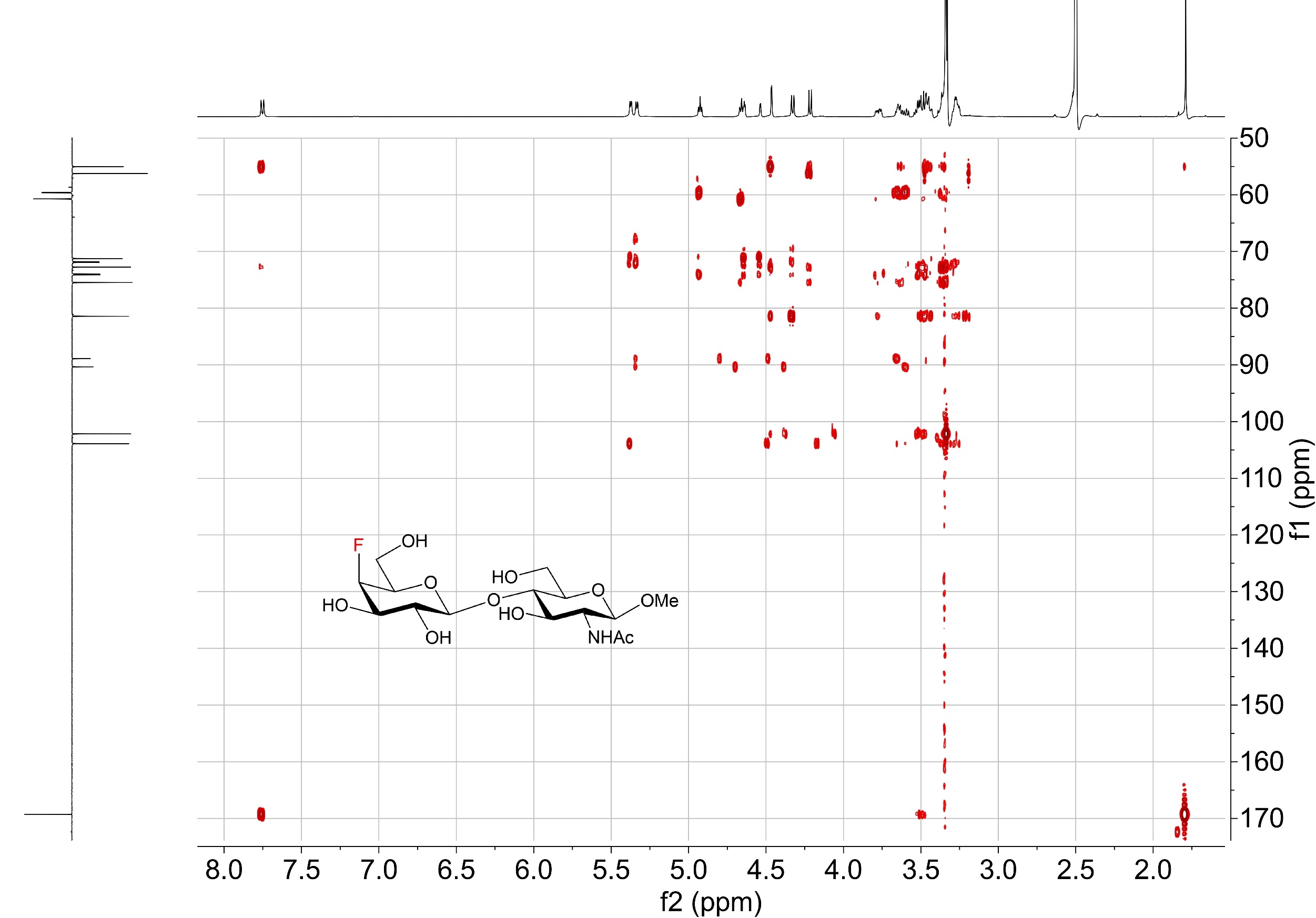


## ^1^H-^1^H ROESY NMR (DMSO-*d*_6_) 4′F-LN **7**

^
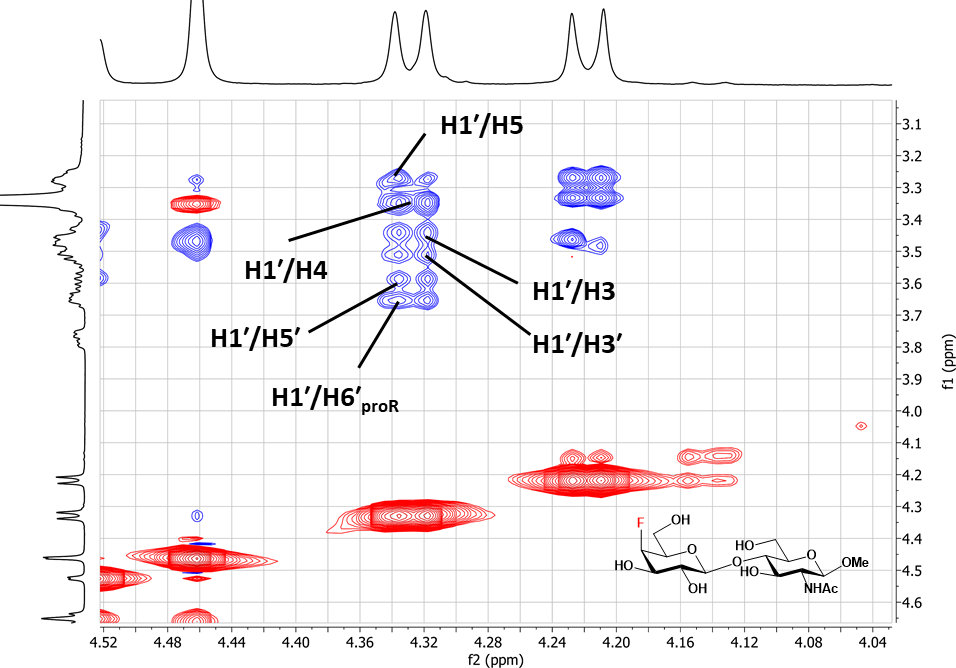
^

## ^1^H-^1^H ROESY NMR (DMSO-*d*_6_) 4′F-LN **7**

^
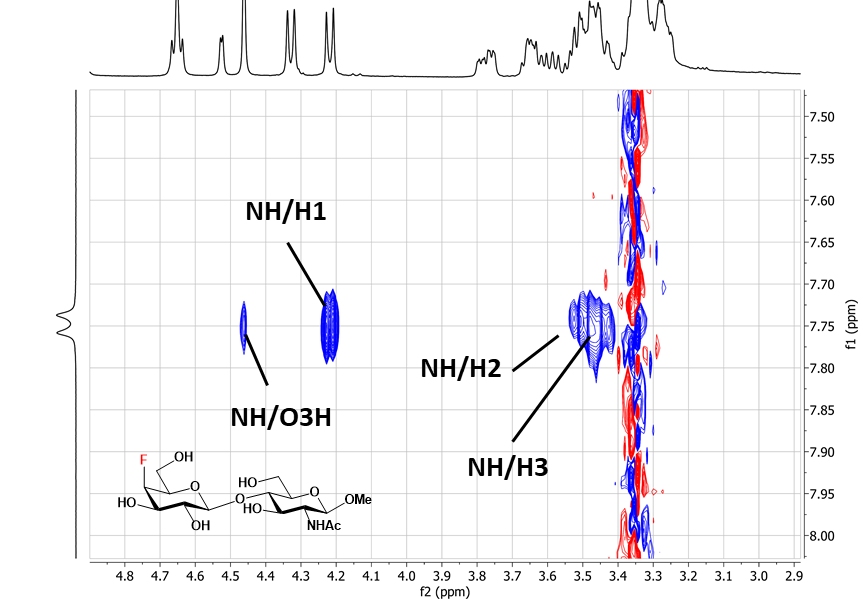
^

## ^1^H-^1^H ROESY NMR (DMSO-*d*_6_) 4′F-LN **7**

^
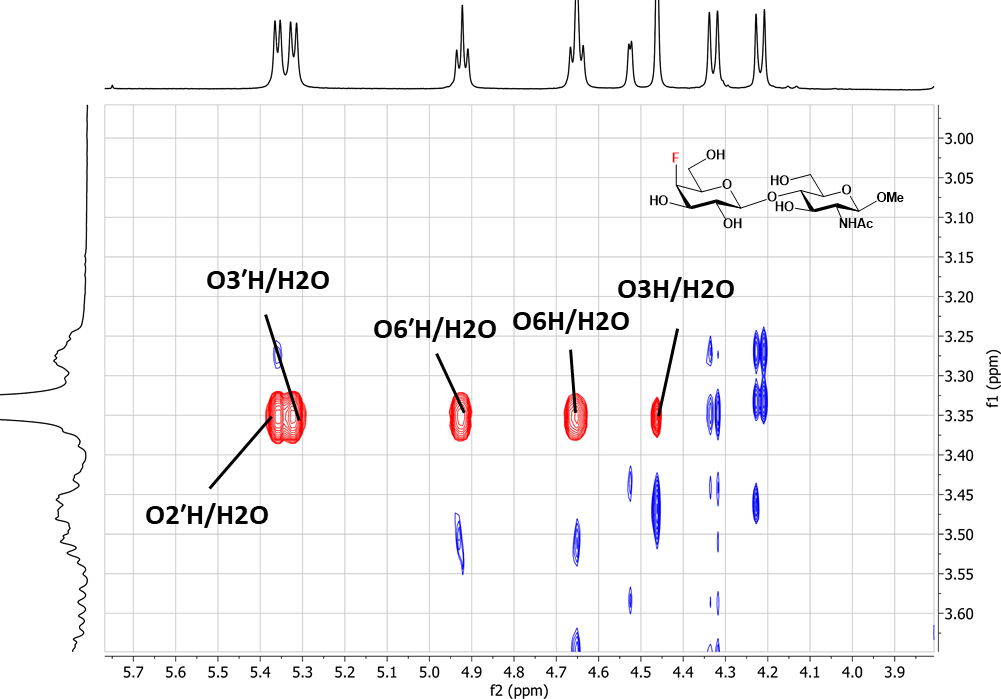
^

## ^1^H-^1^H ROESY NMR (DMSO-*d*_6_) 4′F-LN **7**

^
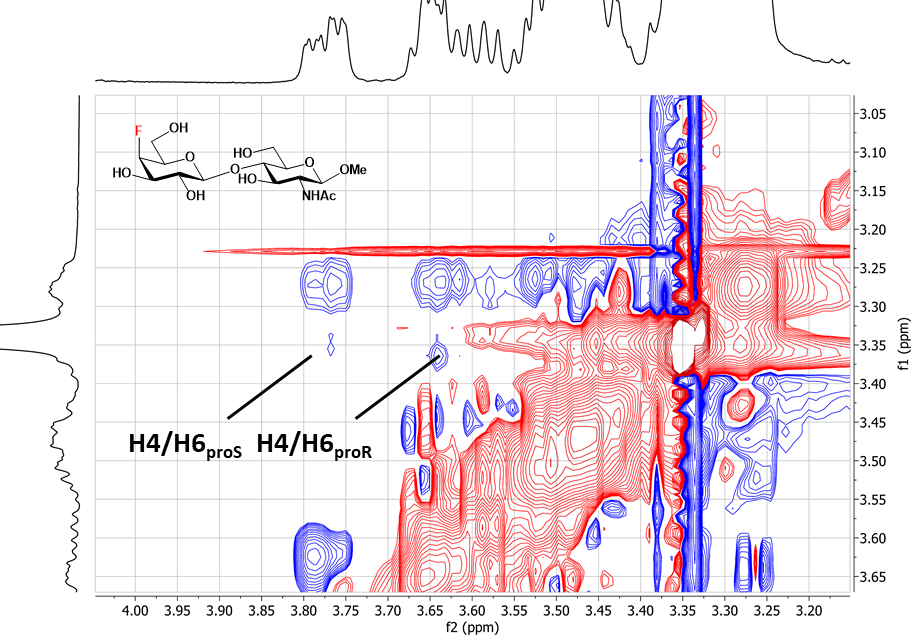
^

## ^1^H-^1^H ROESY NMR (DMSO-*d*_6_, 60 °C) 4′F-LN **7**

^
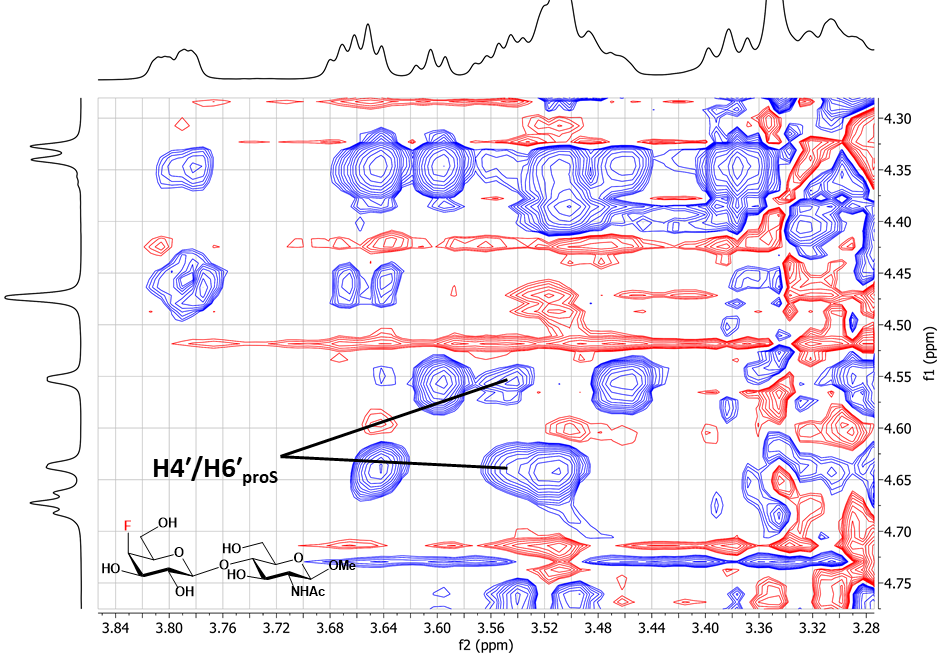
^

## Selective Homonuclear Decoupled ^1^H-NMR (DMSO-*d*_6_) 4′F-LN **7**

(irradiation frequency: 4.659 ppm)

^
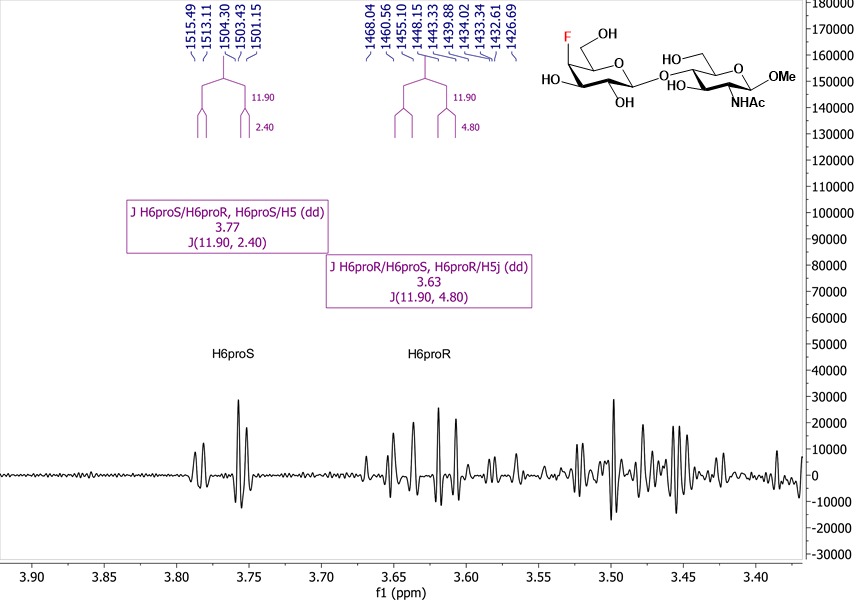
^

## 1D Selective Gradient ^1^H-^1^H TOCSY NMR (DMSO-*d*_6_) 4′F-LN **7**

(irradiation frequency: 4.943 ppm) ^
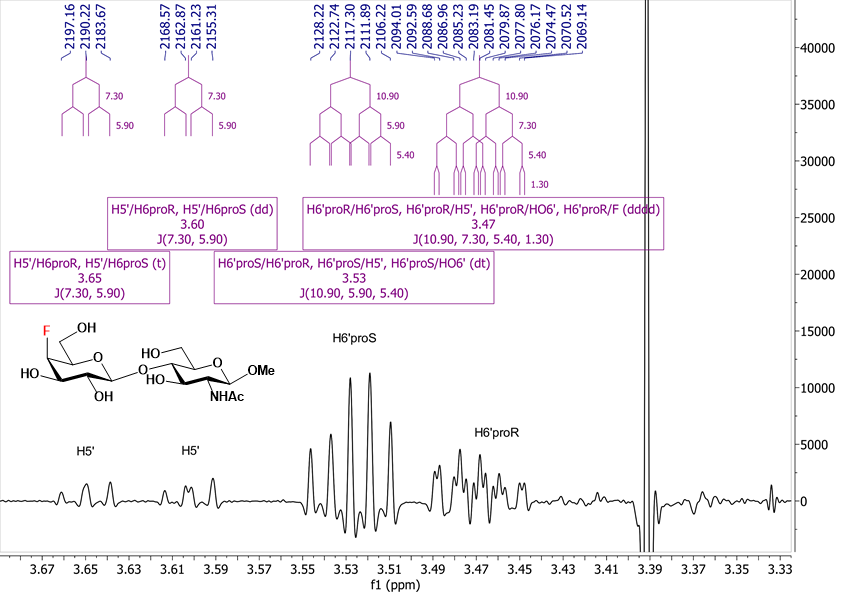
^

## Temperature Dependent ^1^H-NMR (DMSO-*d*_6_) 4′F-LN **7**

^
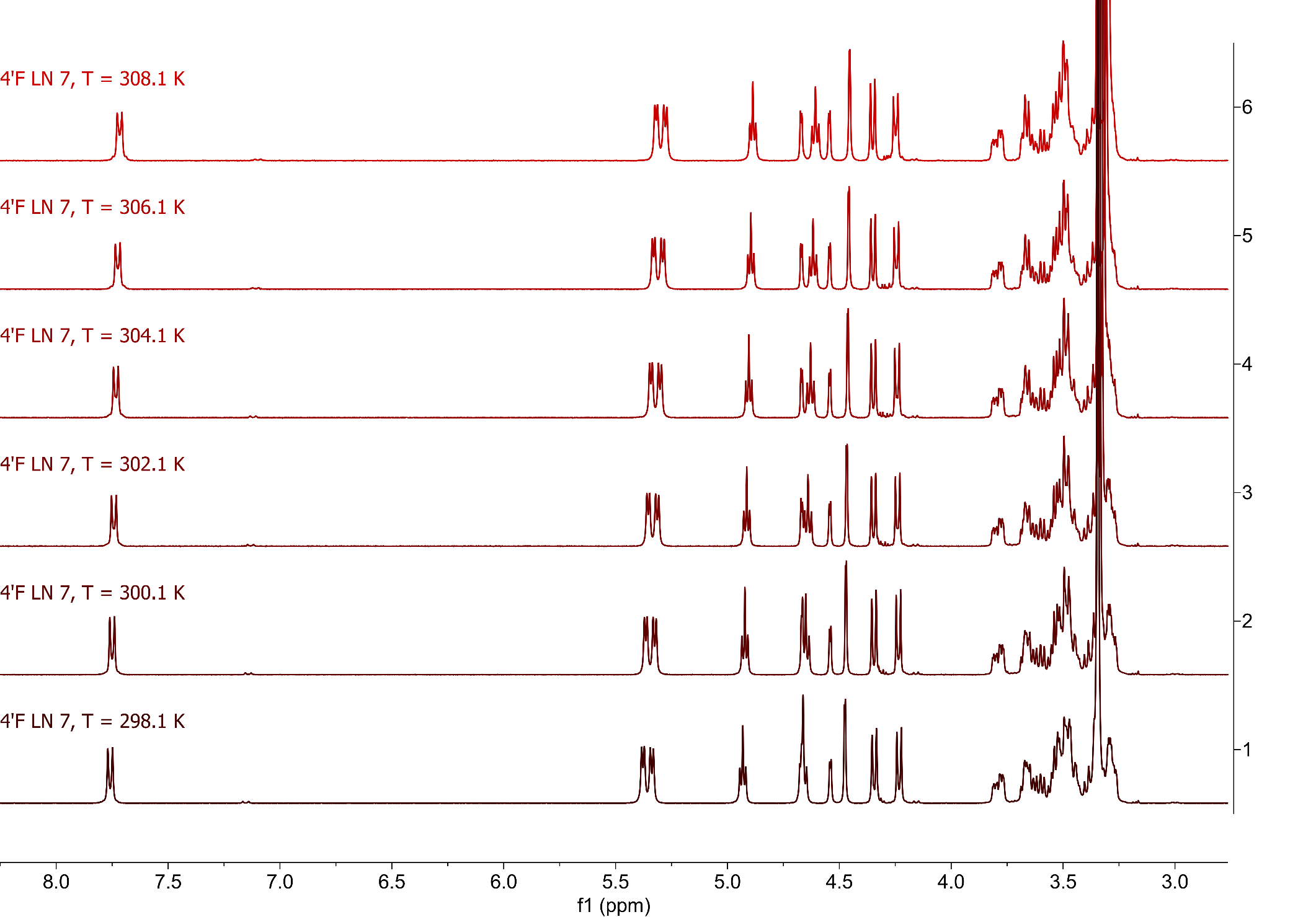
^

## ^1^H NMR (400 MHz, DMSO-*d*_6_) 6′F-LN **8**

**
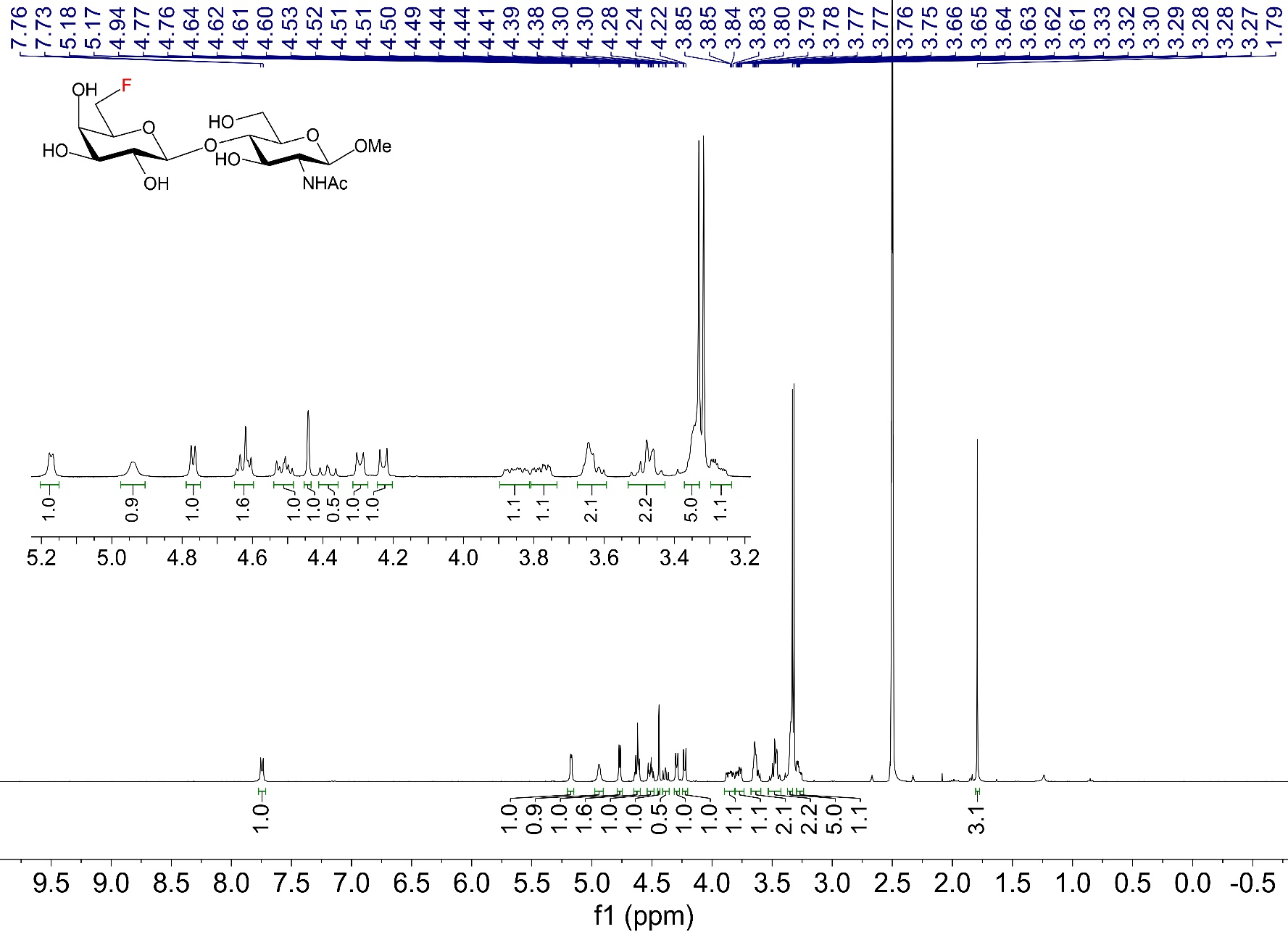
**

## ^13^C{^1^H} NMR (101 MHz, DMSO-*d*_6_) 6′F-LN **8**

**
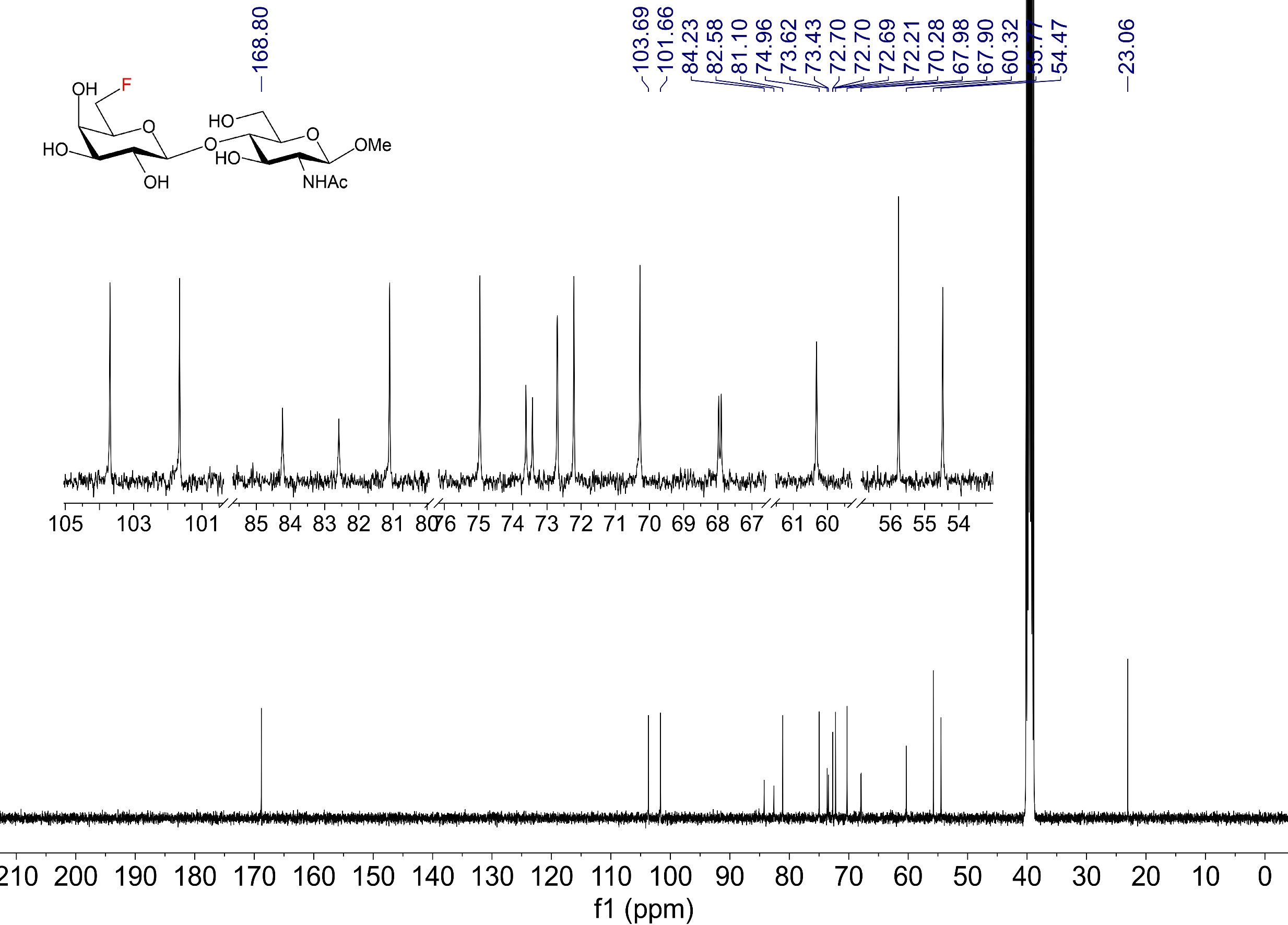
**

## ^19^F NMR (376 MHz, DMSO-*d*_6_) 6′F-LN **8**

**
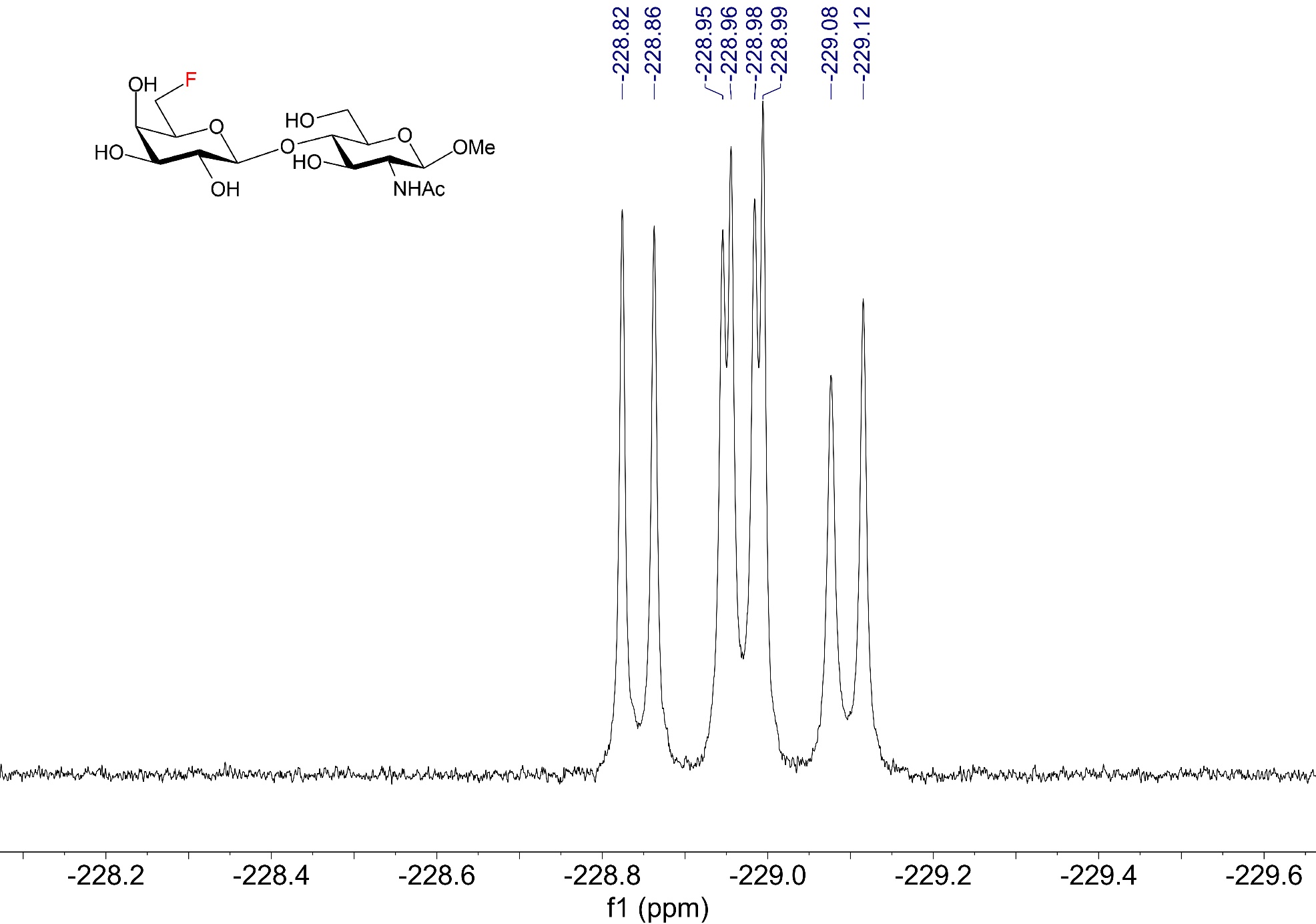
**

## ^1^H-^1^H COSY NMR (DMSO-*d*_6_) 6′F-LN **8**


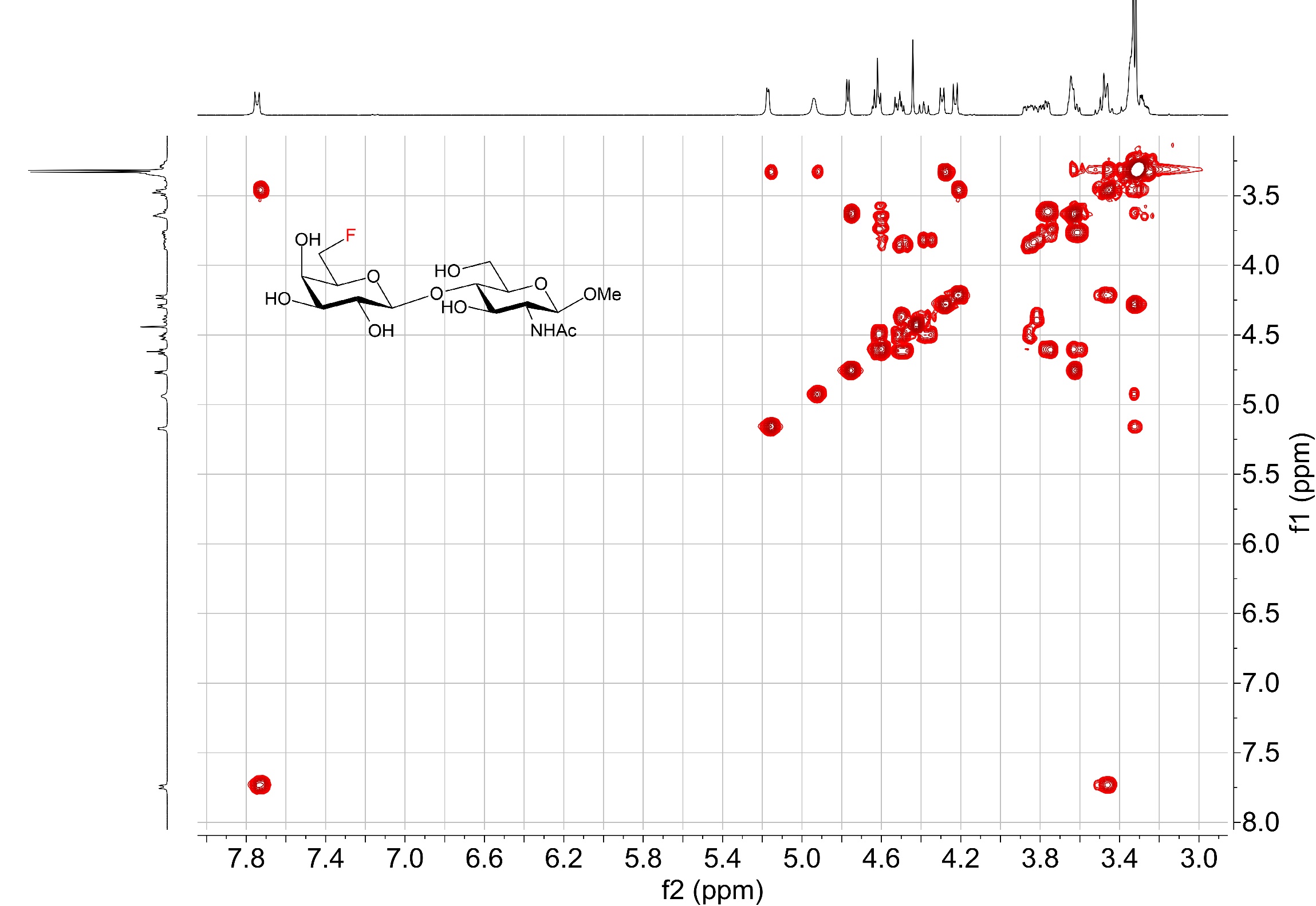


## ^1^H-^13^C HSQC NMR (DMSO-*d*_6_) 6′F-LN **8**

**
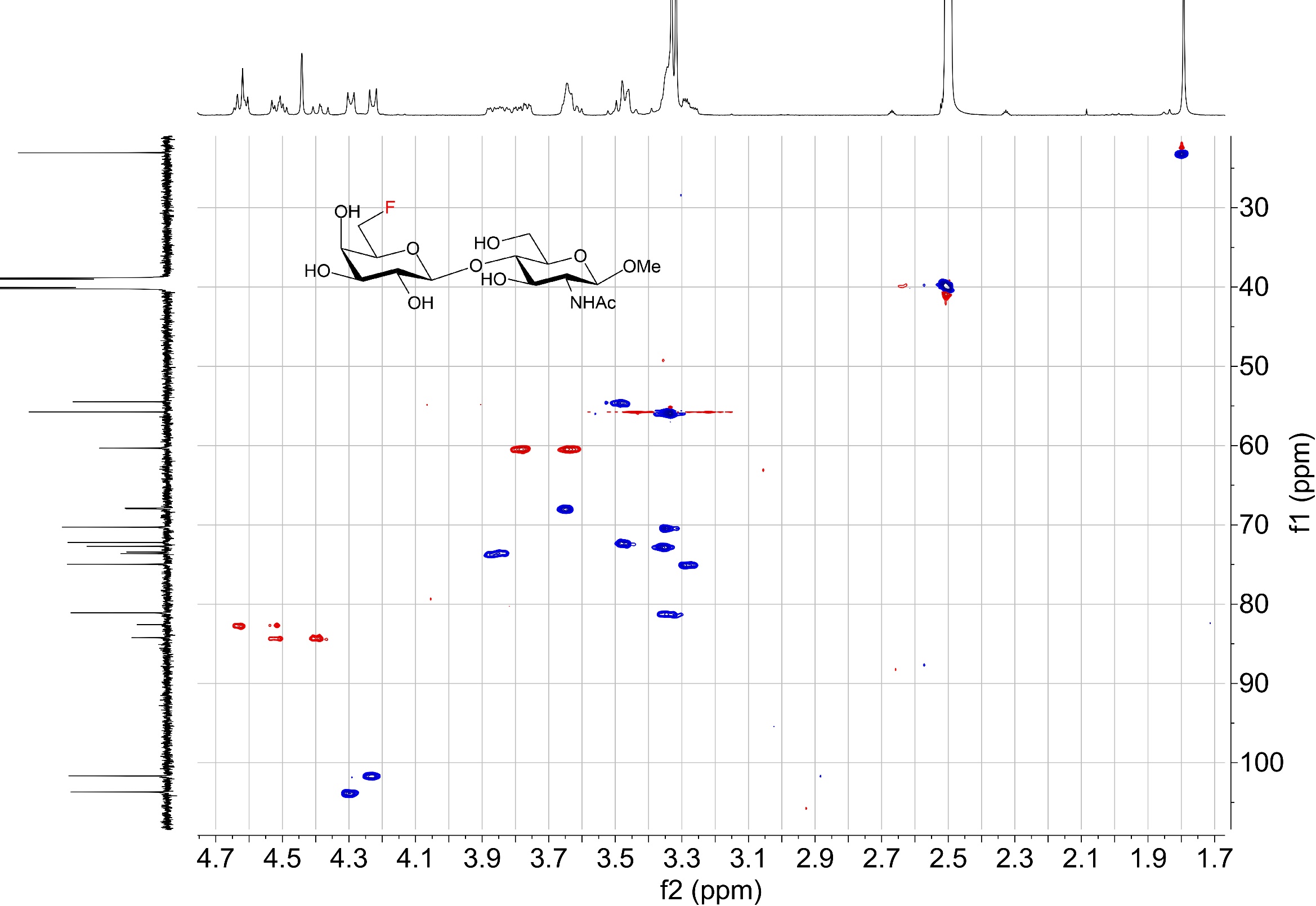
**

## ^1^H-^13^C HMBC NMR (DMSO-*d*_6_) 6′F-LN **8**


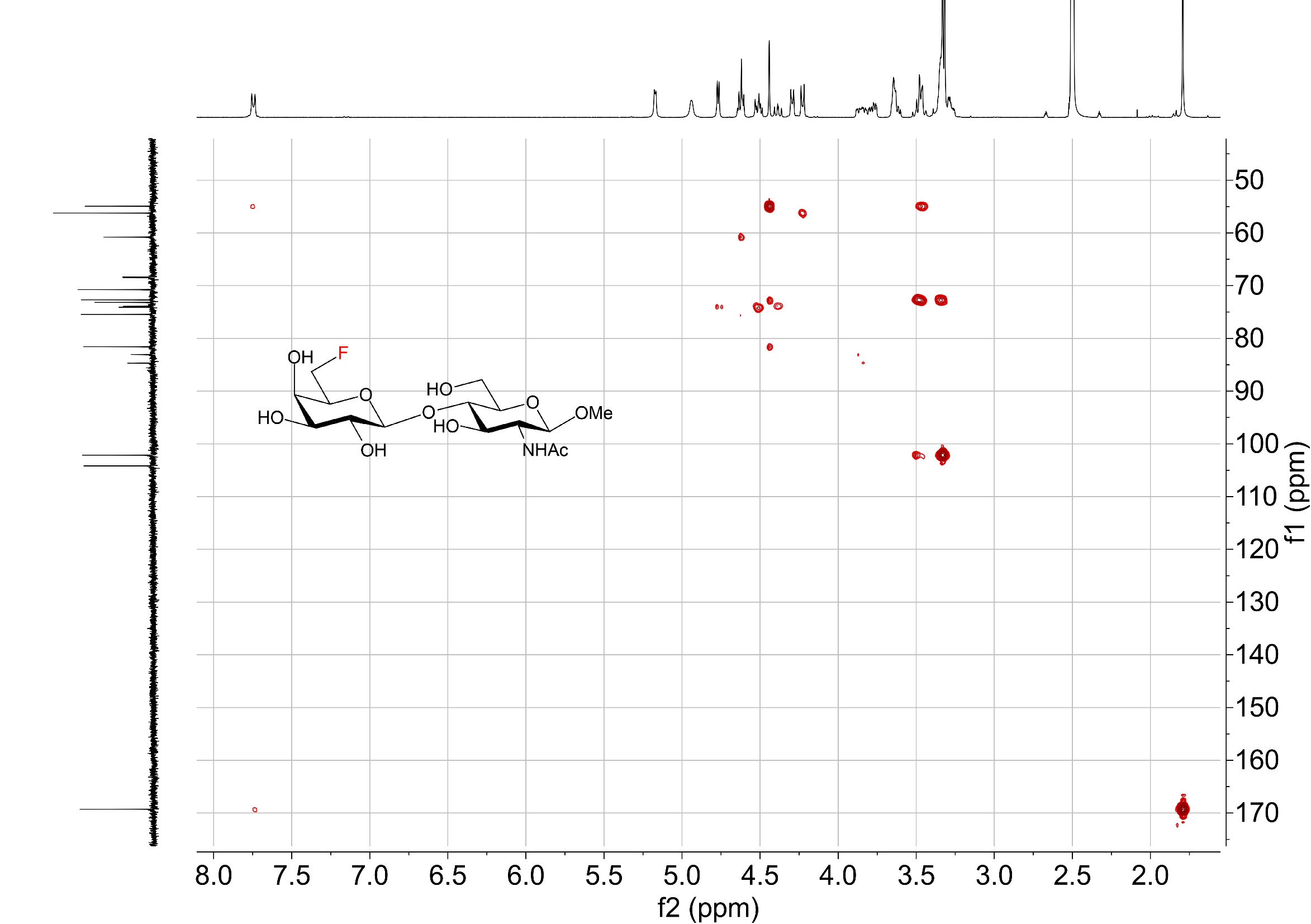


## ^1^H-^1^H ROESY NMR (DMSO-*d*_6_) 6′F-LN **8**

^
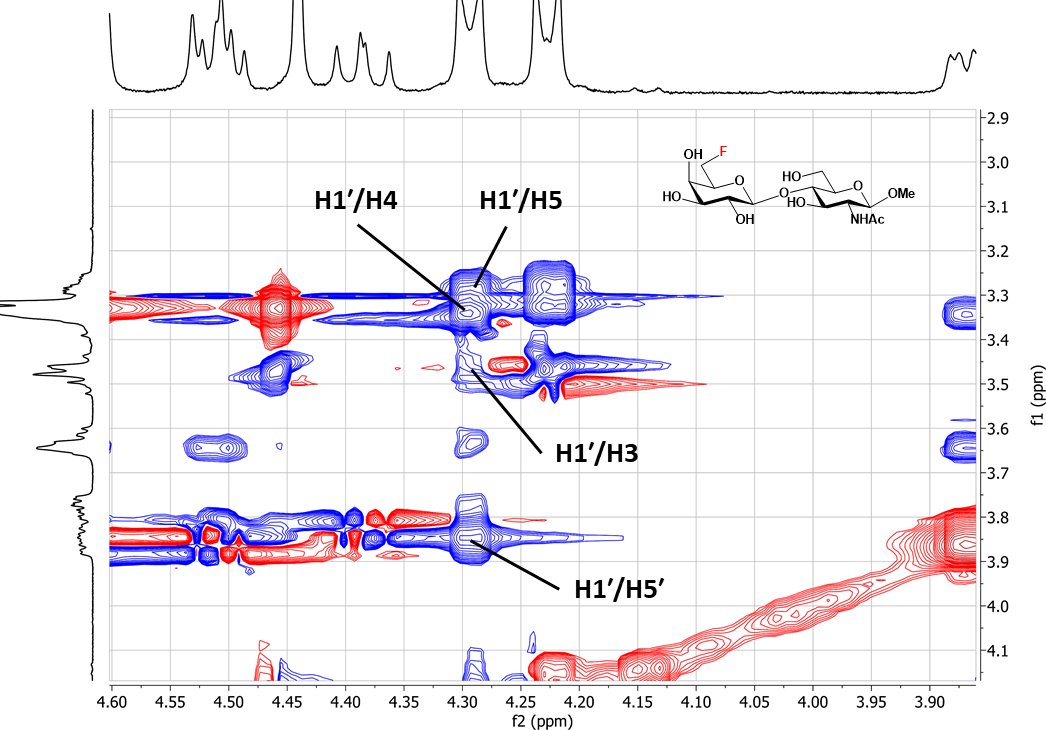
^

## ^1^H-^1^H ROESY NMR (DMSO-*d*_6_) 6′F-LN **8**

^
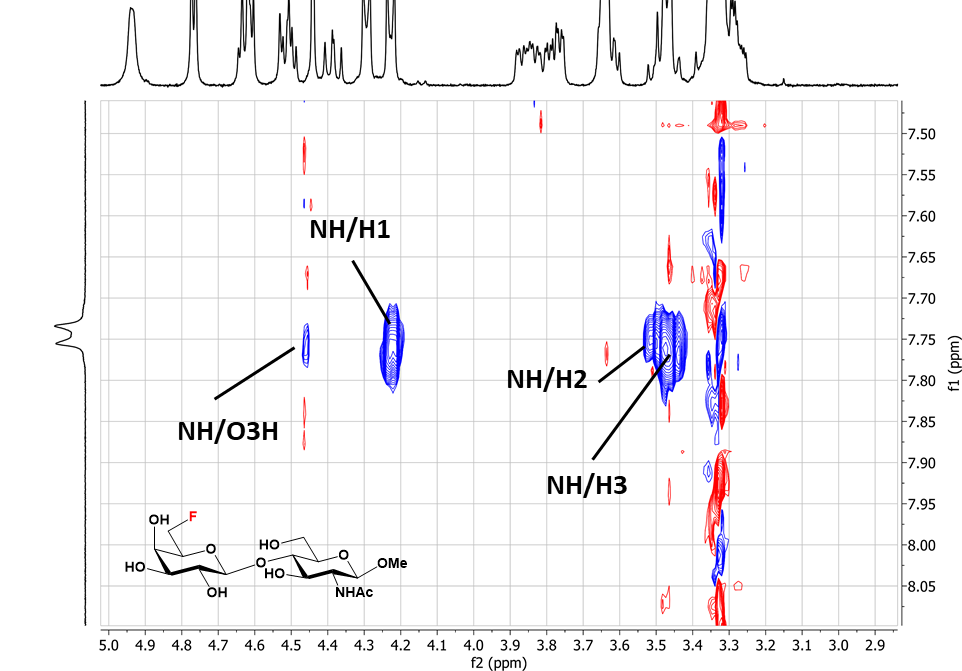
^

## ^1^H-^1^H ROESY NMR (DMSO-*d*_6_) 6′F-LN **8**

^
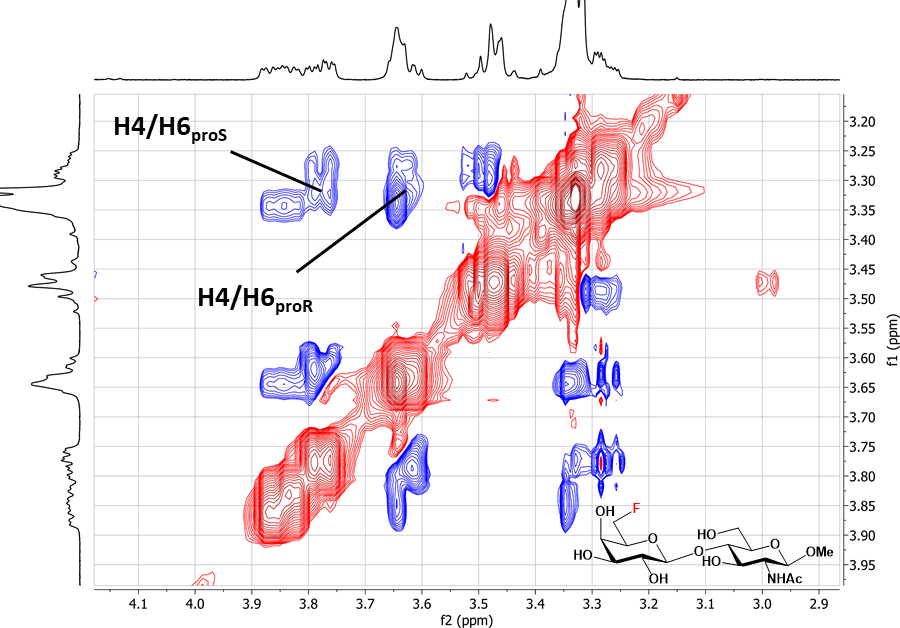
^

## ^1^H-^1^H ROESY NMR (DMSO-*d*_6_) 6′F-LN **8**

^
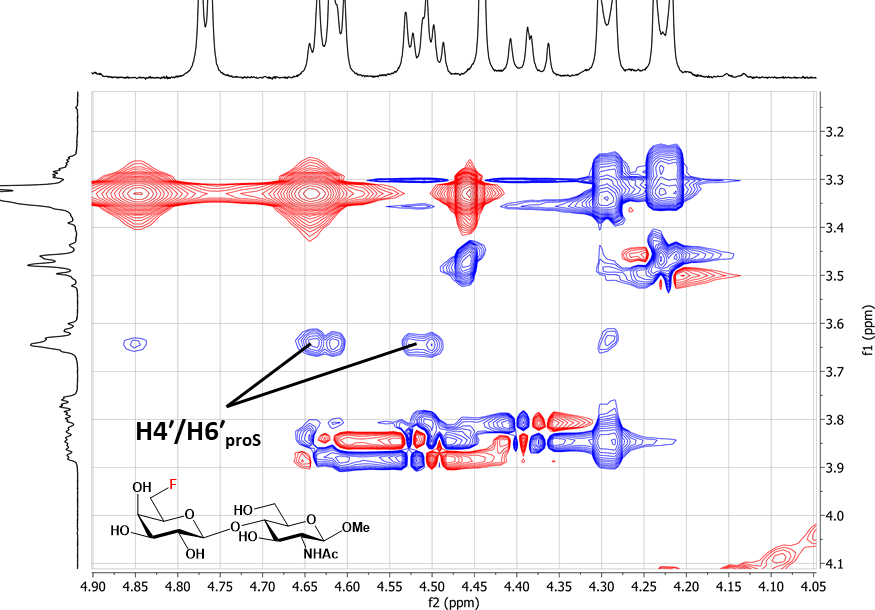
^

## ^1^H-^1^H ROESY NMR (DMSO-*d*_6_) 6′F-LN **8**

^
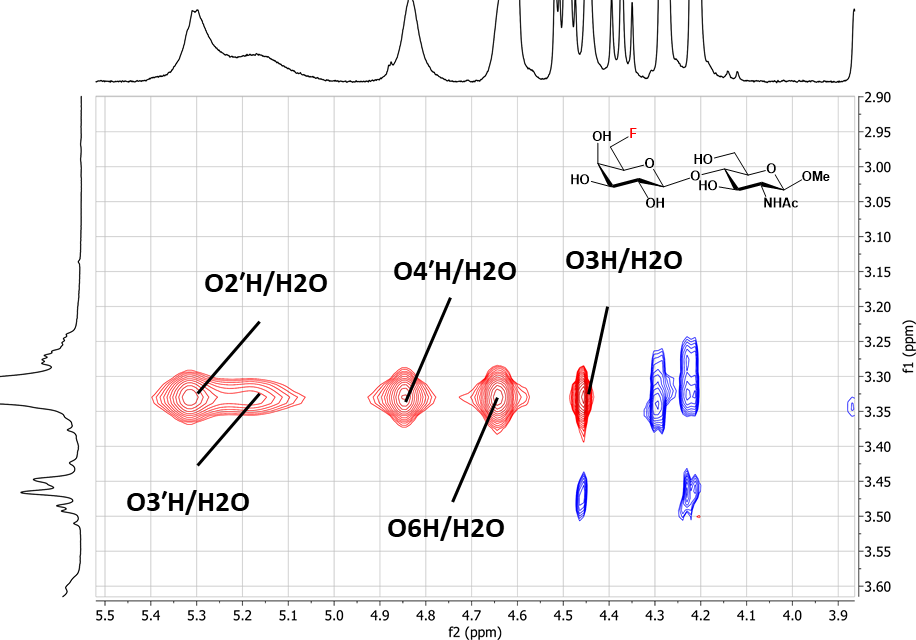
^

## ^1^H NMR (400 MHz, DMSO-*d*_6_) 6′F-LN **8**

^
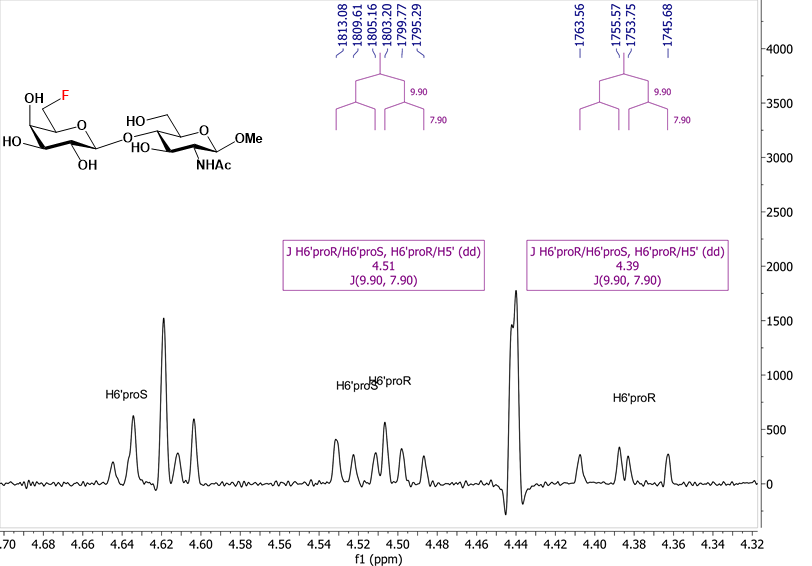
^

## ^1^H NMR (400 MHz, DMSO-*d*_6_) 6′F-LN **8**

^
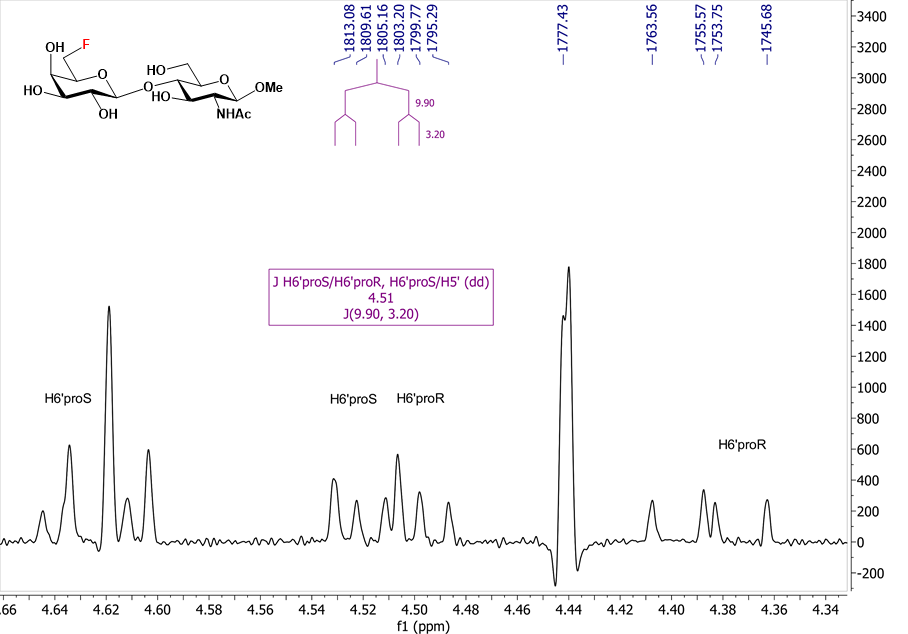
^

## ^1^H NMR (400 MHz, DMSO-*d*_6_) 6′F-LN **8**

^
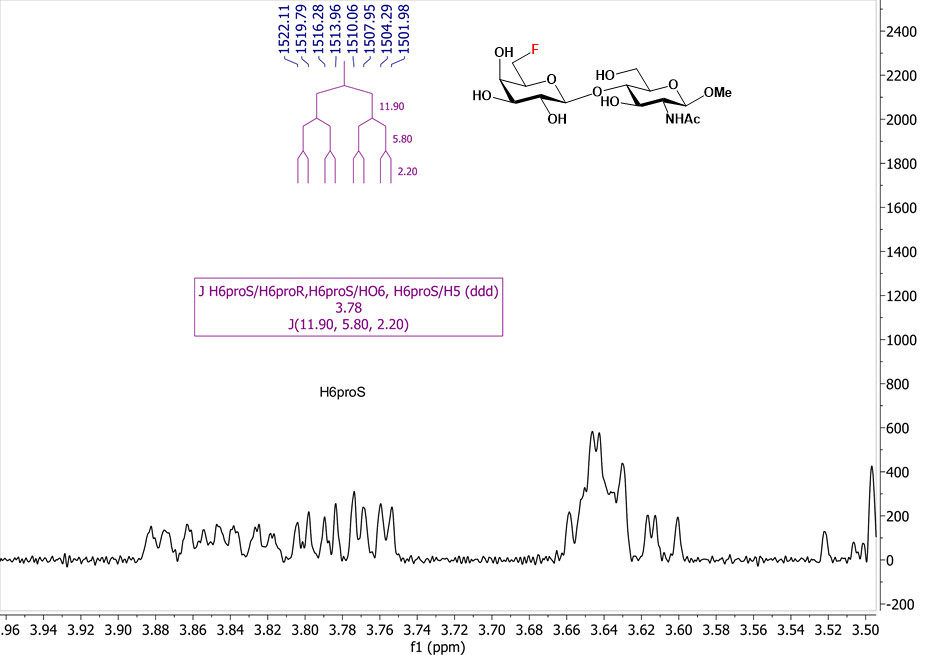
^

## ^1^H NMR (400 MHz, DMSO-*d*_6_) 6′F-LN **8**

^
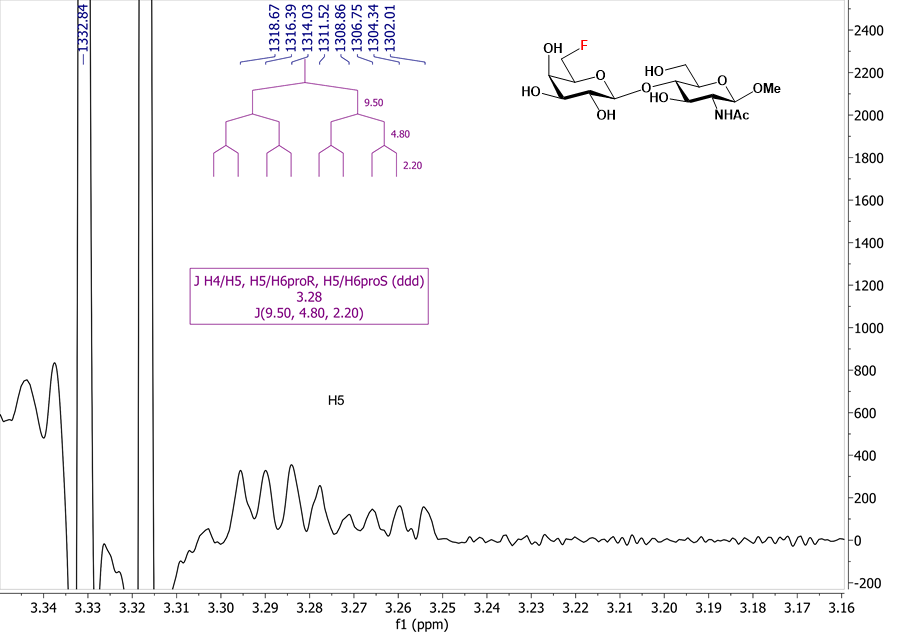
^

## ^1^H NMR (400 MHz, DMSO-*d*_6_, 31 °C) 6′F-LN **8**

^
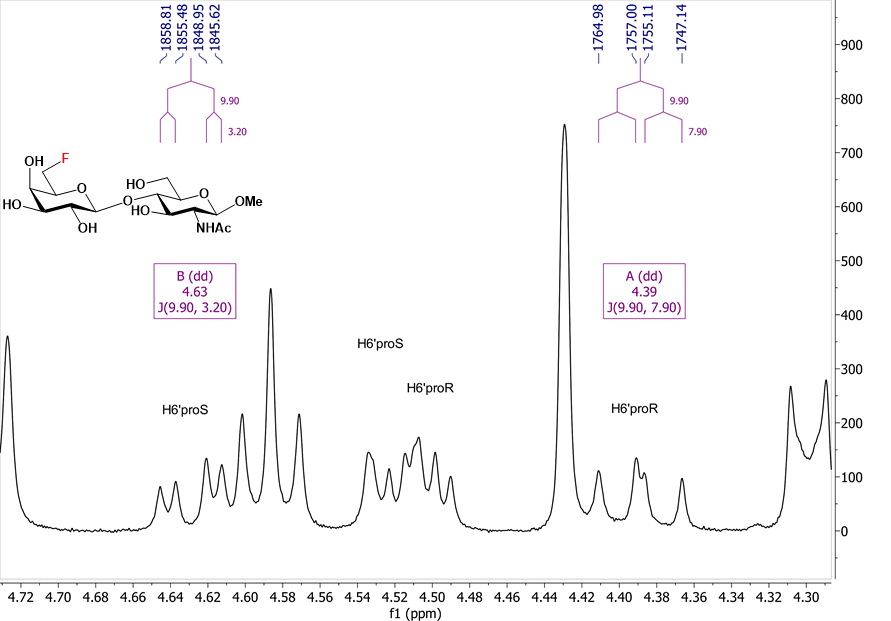
^

## Temperature Dependent ^1^H-NMR (DMSO-*d*_6_) 6′F-LN **8**

^
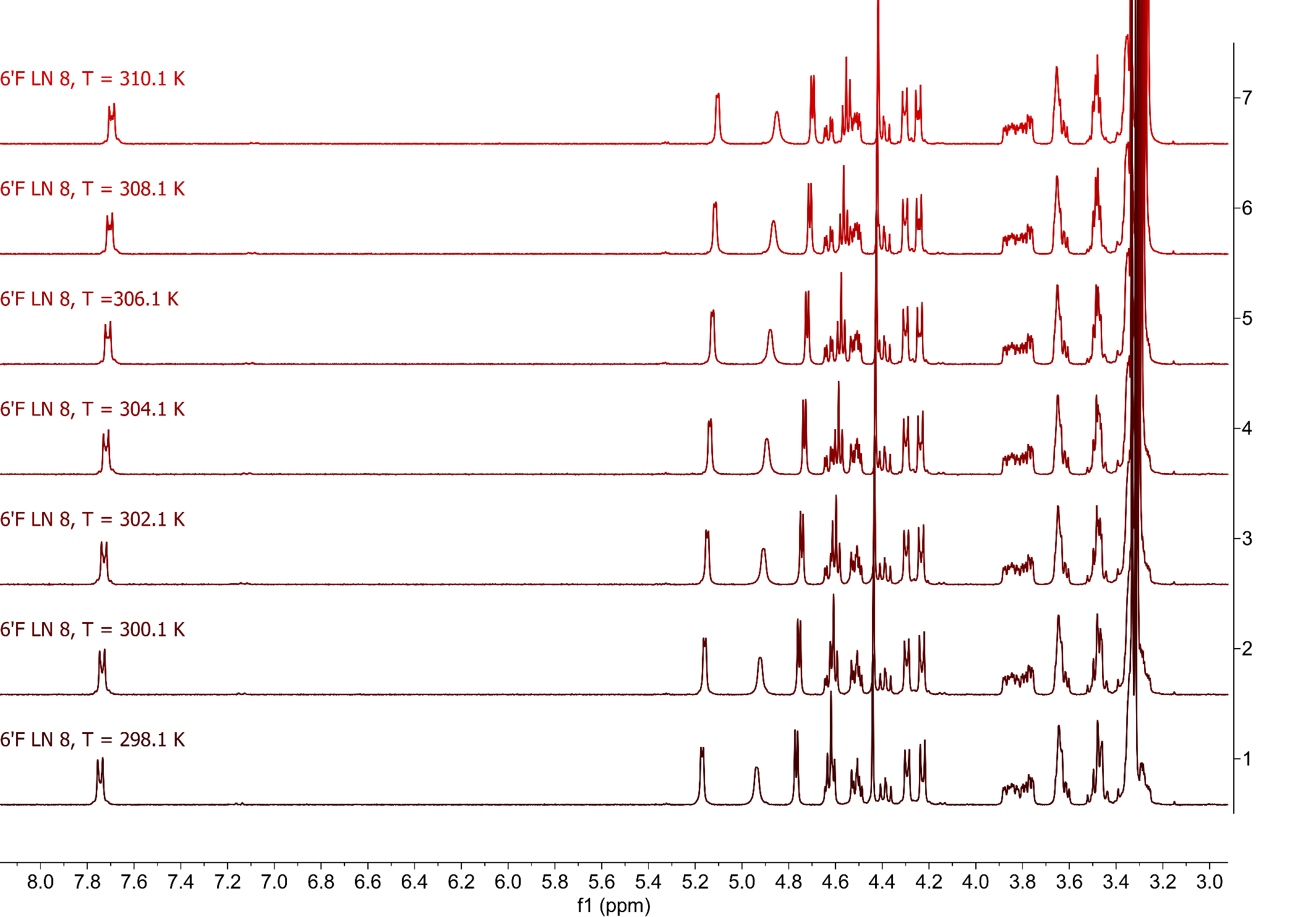
^

# Cartesian coordinates (Å), computed total energy values E(B3LYP), computed sum of electronic and thermal free energies (G) and number of imaginary frequencies of optimized geometries

## LN **2** Conformer A

**
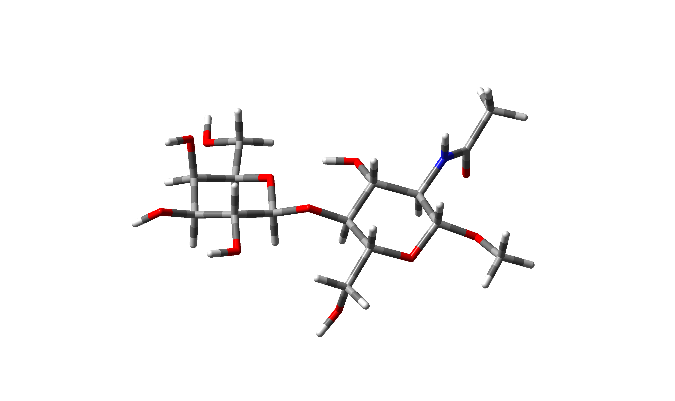
**

| **Atom** | **x** | **y** | **z** |
| --- | --- | --- | --- |
| H | 4.12088700 | 4.37388900 | 1.22033200 |
| O | 4.11328500 | 3.41756300 | 1.33137700 |
| C | 3.05100100 | 2.87823800 | 0.54281800 |
| H | 3.14150900 | 3.17552500 | -0.50580700 |
| H | 2.07398400 | 3.19838200 | 0.92144700 |
| C | 3.13772100 | 1.36254000 | 0.64695200 |
| H | 3.08193600 | 1.07720100 | 1.70637900 |
| O | 1.99355000 | 0.84785900 | -0.04993900 |
| C | 1.84321300 | -0.57470600 | 0.04263100 |
| H | 1.74291600 | -0.86278000 | 1.09958600 |
| O | 0.70840600 | -0.91785400 | -0.67603300 |
| C | -0.55369500 | -0.79048200 | 0.00005100 |
| H | -0.39401400 | -0.66990400 | 1.07480400 |
| C | -1.30706000 | 0.42655600 | -0.53986600 |
| H | -1.29828000 | 0.35591500 | -1.63845400 |
| O | -0.71065400 | 1.64888800 | -0.12730500 |
| H | 0.25573200 | 1.55663900 | -0.19837100 |
| C | -2.76147200 | 0.42961000 | -0.04971200 |
| H | -2.77538500 | 0.61474200 | 1.02499700 |
| N | -3.51612100 | 1.49317300 | -0.68482900 |
| H | -3.53080500 | 1.51825700 | -1.69385700 |
| C | -4.18364600 | 2.46295100 | -0.00894800 |
| O | -4.20881000 | 2.52849900 | 1.22077300 |
| C | -4.90860100 | 3.48119200 | -0.86504000 |
| H | -4.79526100 | 3.31403400 | -1.93692400 |
| H | -5.97013900 | 3.45479800 | -0.61002200 |
| H | -4.53157600 | 4.47526400 | -0.61613300 |
| C | -3.41492100 | -0.93951700 | -0.29529500 |
| H | -3.48700000 | -1.14699600 | -1.37797600 |
| O | -4.67219100 | -0.94155500 | 0.28746800 |
| C | -5.49081400 | -2.05579300 | -0.09028900 |
| H | -5.04654800 | -2.99831500 | 0.23918300 |
| H | -5.63377000 | -2.07790000 | -1.17677200 |
| H | -6.45298900 | -1.91295600 | 0.39924500 |
| O | -2.63584400 | -1.96651000 | 0.31576500 |
| C | -1.32907000 | -2.08912100 | -0.24783200 |
| H | -1.40284300 | -2.25531800 | -1.33298800 |
| C | -0.64111300 | -3.30141700 | 0.35124800 |
| H | 0.30215700 | -3.45349200 | -0.18165600 |
| C | 3.05488800 | -1.25984300 | -0.58101500 |
| H | 3.07801400 | -0.99949300 | -1.64537400 |
| C | 4.33407700 | -0.75776200 | 0.08897700 |
| H | 4.34044100 | -1.10232100 | 1.12942200 |
| C | 4.42494000 | 0.77470600 | 0.06577600 |
| H | 5.26125600 | 1.09488300 | 0.69903000 |
| O | 2.90258500 | -2.66359200 | -0.40345900 |
| H | 3.73067900 | -3.07630300 | -0.67954100 |
| O | -0.40012200 | -3.07287200 | 1.74485600 |
| H | 0.15470600 | -3.78675500 | 2.07538400 |
| H | -1.28522100 | -4.17564500 | 0.20441100 |
| O | 5.42199100 | -1.35020200 | -0.63296200 |
| H | 6.19713000 | -1.39402100 | -0.06127500 |
| O | 4.60280700 | 1.25298100 | -1.26476700 |
| H | 5.28262200 | 0.70189300 | -1.67419300 |
| E(RB3LYP):-1470.58351978 | | | |
| Sum of electronic and thermal free energies: -1470.199267 | | | |
| Number of imaginary frequencies: 0 | | | |

## LN **2** Conformer B

**
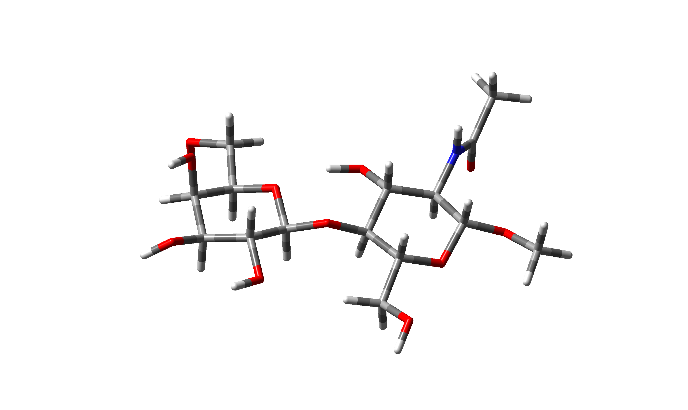
**

| **Atom** | **x** | **y** | **z** |
| --- | --- | --- | --- |
| H | 4.12248000 | 3.62884500 | 1.81026700 |
| O | 4.27473100 | 3.59119900 | 0.85915000 |
| C | 3.18643100 | 2.90586800 | 0.24432500 |
| H | 3.28350300 | 3.05988600 | -0.82994900 |
| H | 2.22219900 | 3.30941900 | 0.57078600 |
| C | 3.22258100 | 1.40783300 | 0.54607500 |
| H | 3.16127700 | 1.25907300 | 1.63371700 |
| O | 2.05667900 | 0.84464500 | -0.07670500 |
| C | 1.86264200 | -0.55076800 | 0.19102100 |
| H | 1.75371500 | -0.70025800 | 1.27549400 |
| O | 0.71946800 | -0.95168600 | -0.48483300 |
| C | -0.54738000 | -0.76167500 | 0.16667800 |
| H | -0.40118800 | -0.63494200 | 1.24557800 |
| C | -1.23652800 | 0.48838800 | -0.39598200 |
| H | -1.18882900 | 0.41179700 | -1.49305900 |
| O | -0.60564400 | 1.68415100 | 0.03781800 |
| H | 0.35446600 | 1.58564300 | -0.09252600 |
| C | -2.70744900 | 0.56088000 | 0.03554300 |
| H | -2.75737700 | 0.75217900 | 1.10810900 |
| N | -3.38682400 | 1.65375300 | -0.63347900 |
| H | -3.37216300 | 1.66663600 | -1.64278000 |
| C | -4.02542400 | 2.66202000 | 0.01343900 |
| O | -4.08205700 | 2.74157800 | 1.24128900 |
| C | -4.67518700 | 3.70562400 | -0.87179800 |
| H | -4.54697700 | 3.51864700 | -1.93868200 |
| H | -5.74200600 | 3.73936300 | -0.64101800 |
| H | -4.25101800 | 4.68141200 | -0.62624600 |
| C | -3.40902300 | -0.77829900 | -0.23117100 |
| H | -3.44395900 | -0.99190100 | -1.31430200 |
| O | -4.68806900 | -0.72369000 | 0.29942100 |
| C | -5.53457900 | -1.80871500 | -0.10187900 |
| H | -5.14010000 | -2.76475400 | 0.25099100 |
| H | -5.63672200 | -1.83442500 | -1.19284600 |
| H | -6.50838300 | -1.62459500 | 0.34935500 |
| O | -2.69709400 | -1.82710800 | 0.42089300 |
| C | -1.38038100 | -2.02430800 | -0.09504900 |
| H | -1.42311200 | -2.20244900 | -1.17925100 |
| C | -0.78351300 | -3.24373900 | 0.57813100 |
| H | -0.91868200 | -3.14949200 | 1.66277900 |
| C | 3.05378800 | -1.34536100 | -0.33668300 |
| H | 3.08213400 | -1.22439300 | -1.42551600 |
| C | 4.35034300 | -0.80232700 | 0.26427100 |
| H | 4.35126100 | -1.01057800 | 1.34046600 |
| C | 4.48699800 | 0.71040500 | 0.04380300 |
| H | 5.33666100 | 1.08370300 | 0.62879000 |
| O | 2.85815900 | -2.70915300 | 0.01932500 |
| H | 3.67170600 | -3.18040800 | -0.20067600 |
| O | -1.45167300 | -4.40830600 | 0.07917700 |
| H | -1.06336100 | -5.18272200 | 0.49977900 |
| H | 0.28659800 | -3.27932000 | 0.35416300 |
| O | 5.41605400 | -1.51593100 | -0.37638000 |
| H | 6.19178800 | -1.51101300 | 0.19616300 |
| O | 4.67065900 | 1.00826100 | -1.33739300 |
| H | 5.33424000 | 0.39177300 | -1.67367100 |
| E(RB3LYP):-1470.58328816 | | | |
| Sum of electronic and thermal free energies: -1470.198820 | | | |
| Number of imaginary frequencies: 0 | | | |
| LN **2** Conformer C | | | |
| **Atom** | **x** | **y** | **z** |
| H | -0.95422500 | -2.71655400 | 1.37351900 |
| O | -1.70829600 | -3.13310800 | 1.81081100 |
| C | -2.88011100 | -2.87379600 | 1.03571600 |
| H | -3.71294700 | -3.34626700 | 1.55883200 |
| H | -2.79948100 | -3.31466700 | 0.03726300 |
| C | -3.14407100 | -1.38179000 | 0.90888900 |
| H | -3.14984800 | -0.93996700 | 1.91460600 |
| O | -2.03589500 | -0.84339900 | 0.16894300 |
| C | -2.01238200 | 0.58031500 | 0.09331400 |
| H | -1.98868800 | 0.99919900 | 1.11124400 |
| O | -0.87404500 | 0.93699300 | -0.62141600 |
| C | 0.38058300 | 0.76688500 | 0.06435900 |
| H | 0.20569200 | 0.49943300 | 1.11196100 |
| C | 1.16181800 | -0.36375900 | -0.61639000 |
| H | 1.17083400 | -0.14265000 | -1.69453400 |
| O | 0.56339600 | -1.63508400 | -0.39489300 |
| H | -0.39492900 | -1.52768100 | -0.51003900 |
| C | 2.60672800 | -0.43046600 | -0.10647600 |
| H | 2.60576100 | -0.75679500 | 0.93427700 |
| N | 3.37618100 | -1.39820500 | -0.86467800 |
| H | 3.41368100 | -1.28707700 | -1.86742500 |
| C | 4.02454900 | -2.45505800 | -0.31078900 |
| O | 4.01720600 | -2.68693500 | 0.89866600 |
| C | 4.76776600 | -3.35202100 | -1.27907500 |
| H | 4.71148300 | -3.02010700 | -2.31653200 |
| H | 5.81528700 | -3.39748000 | -0.97449400 |
| H | 4.35556200 | -4.36053300 | -1.20371500 |
| C | 3.25371900 | 0.96213900 | -0.15738000 |
| H | 3.34789900 | 1.31119900 | -1.20063500 |
| O | 4.49616900 | 0.89599000 | 0.45193400 |
| C | 5.31422300 | 2.05417800 | 0.23996000 |
| H | 4.85107000 | 2.94529200 | 0.67070600 |
| H | 5.48774100 | 2.21200600 | -0.83061900 |
| H | 6.26329900 | 1.85748800 | 0.73623900 |
| O | 2.44927500 | 1.89229500 | 0.56521200 |
| C | 1.15561500 | 2.08947800 | -0.00698100 |
| H | 1.24972300 | 2.39326700 | -1.05952900 |
| C | 0.46067800 | 3.19675500 | 0.75916600 |
| H | 0.52988600 | 2.98437100 | 1.83304300 |
| C | -3.25155900 | 1.06041600 | -0.65412200 |
| H | -3.19354300 | 0.67370800 | -1.67811800 |
| C | -4.51127400 | 0.50803300 | 0.02141500 |
| H | -4.61615200 | 0.98701400 | 1.00133100 |
| C | -4.45515600 | -1.01792800 | 0.21421000 |
| H | -5.28940700 | -1.32079700 | 0.86148900 |
| O | -3.25461100 | 2.48229800 | -0.64426500 |
| H | -4.09453100 | 2.76943700 | -1.02422000 |
| O | 1.09779000 | 4.43716500 | 0.43956000 |
| H | 0.64912400 | 5.14090300 | 0.92016600 |
| H | -0.59390700 | 3.21472700 | 0.46691800 |
| O | -5.60647700 | 0.87493000 | -0.82671900 |
| H | -6.41197200 | 0.93423100 | -0.30033600 |
| O | -4.53011400 | -1.69536800 | -1.03621800 |
| H | -5.23261800 | -1.27077300 | -1.54646800 |
| E(RB3LYP): -1470.58515412 | | | |
| Sum of electronic and thermal free energies: -1470.199826 | | | |
| Number of imaginary frequencies: 0 | | | |

## LN **2** Conformer D

| **Atom** | **x** | **y** | **z** |
| --- | --- | --- | --- |
| H | 0.80705200 | 2.59530300 | 1.40018200 |
| O | 1.53324400 | 3.01017200 | 1.88406600 |
| C | 2.73519100 | 2.82820800 | 1.13328900 |
| H | 3.53842900 | 3.29081400 | 1.70895900 |
| H | 2.67184800 | 3.32483100 | 0.16010600 |
| C | 3.04355300 | 1.35354300 | 0.92717400 |
| H | 3.04381100 | 0.85367000 | 1.90566900 |
| O | 1.96564600 | 0.83090200 | 0.13569900 |
| C | 1.98347600 | -0.58728100 | -0.02565000 |
| H | 1.94837600 | -1.07135900 | 0.96178300 |
| O | 0.86444400 | -0.92272100 | -0.78039200 |
| C | -0.39242000 | -0.81822700 | -0.08642400 |
| H | -0.22118800 | -0.58458000 | 0.96690800 |
| C | -1.22067600 | 0.30104300 | -0.71929700 |
| H | -1.25384800 | 0.11609700 | -1.80376200 |
| O | -0.65745900 | 1.58531100 | -0.47170200 |
| H | 0.30467700 | 1.50333700 | -0.57340200 |
| C | -2.65003200 | 0.29774800 | -0.16341700 |
| H | -2.62571200 | 0.59313900 | 0.88623000 |
| N | -3.47881600 | 1.25793500 | -0.86671900 |
| H | -3.53281900 | 1.18335500 | -1.87208600 |
| C | -4.15784100 | 2.26503900 | -0.25959400 |
| O | -4.13474500 | 2.45089900 | 0.95760200 |
| C | -4.95826500 | 3.16569900 | -1.17766000 |
| H | -4.88926100 | 2.89154200 | -2.23111600 |
| H | -6.00496100 | 3.13352200 | -0.86807100 |
| H | -4.60552500 | 4.19133300 | -1.05104300 |
| C | -3.25048800 | -1.11619000 | -0.23634500 |
| H | -3.36791000 | -1.43443600 | -1.28740200 |
| O | -4.47486100 | -1.10943100 | 0.41202300 |
| C | -5.26590000 | -2.28266900 | 0.18261200 |
| H | -4.76717100 | -3.17424400 | 0.57073600 |
| H | -5.46407600 | -2.41005100 | -0.88776100 |
| H | -6.20628900 | -2.12975000 | 0.70991800 |
| O | -2.39596400 | -2.04633700 | 0.42824600 |
| C | -1.11157800 | -2.16707200 | -0.18689600 |
| H | -1.22735900 | -2.43419700 | -1.24780100 |
| C | -0.34360200 | -3.29119000 | 0.48255600 |
| H | 0.56425800 | -3.47711900 | -0.10001400 |
| C | 3.24761200 | -0.98445300 | -0.77894100 |
| H | 3.19672400 | -0.53493300 | -1.77741800 |
| C | 4.47902700 | -0.44148700 | -0.04577900 |
| H | 4.57952000 | -0.97647800 | 0.90517100 |
| C | 4.37753700 | 1.06757200 | 0.23895200 |
| H | 5.19089000 | 1.35179200 | 0.92043500 |
| O | 3.29280500 | -2.40368500 | -0.85996500 |
| H | 4.14791800 | -2.64101000 | -1.24032300 |
| O | -0.01401800 | -2.91081500 | 1.82310800 |
| H | 0.51521300 | -3.61291500 | 2.21544500 |
| H | -0.96595900 | -4.19259200 | 0.47101100 |
| O | 5.60000900 | -0.72614000 | -0.89240900 |
| H | 6.39756100 | -0.79196000 | -0.35477300 |
| O | 4.45891700 | 1.82242500 | -0.96629200 |
| H | 5.17979300 | 1.44498600 | -1.48790800 |
| E(RB3LYP): -1470.58635350 | | | |
| Sum of electronic and thermal free energies: -1470.200949 | | | |
| Number of imaginary frequencies: 0 | | | |

## LN **2** Conformer E

| **Atom** | **x** | **y** | **z** |
| --- | --- | --- | --- |
| H | 2.45785400 | 4.43111100 | 0.00131400 |
| O | 2.76106400 | 3.53549000 | -0.18038500 |
| C | 2.93370200 | 2.87267400 | 1.07100600 |
| H | 2.05165500 | 3.02249500 | 1.70388800 |
| H | 3.80912700 | 3.25750600 | 1.60927300 |
| C | 3.11926900 | 1.37360700 | 0.88931500 |
| H | 3.09348500 | 0.95145700 | 1.90584100 |
| O | 2.00268400 | 0.86756200 | 0.14500200 |
| C | 1.90897600 | -0.56015200 | 0.12466200 |
| H | 1.79884300 | -0.93529600 | 1.15329000 |
| O | 0.80175200 | -0.88906400 | -0.64367500 |
| C | -0.47925500 | -0.82027400 | 0.00500000 |
| H | -0.34937300 | -0.76478400 | 1.08923300 |
| C | -1.24085800 | 0.41668100 | -0.47822100 |
| H | -1.20183400 | 0.41447700 | -1.57852800 |
| O | -0.68328100 | 1.62262700 | 0.02518200 |
| H | 0.28769200 | 1.55598900 | -0.02063200 |
| C | -2.70795400 | 0.36239600 | -0.02917700 |
| H | -2.75309300 | 0.47525700 | 1.05463400 |
| N | -3.46443500 | 1.45444100 | -0.61181200 |
| H | -3.44473000 | 1.55520900 | -1.61603100 |
| C | -4.16564600 | 2.36550900 | 0.10989100 |
| O | -4.23045300 | 2.34234100 | 1.33975500 |
| C | -4.87692300 | 3.43426500 | -0.69443400 |
| H | -4.73062600 | 3.34270500 | -1.77144100 |
| H | -5.94500400 | 3.38109900 | -0.47327100 |
| H | -4.51777600 | 4.41189500 | -0.36637800 |
| C | -3.32984300 | -0.99805100 | -0.38027200 |
| H | -3.36781900 | -1.13833400 | -1.47535900 |
| O | -4.60248900 | -1.05753200 | 0.16524600 |
| C | -5.39008100 | -2.15968800 | -0.30291400 |
| H | -4.93799600 | -3.11338900 | -0.01936400 |
| H | -5.50332700 | -2.11714400 | -1.39227600 |
| H | -6.36758400 | -2.06320400 | 0.16723800 |
| O | -2.54921300 | -2.04831800 | 0.18785100 |
| C | -1.22541000 | -2.11310700 | -0.34429200 |
| H | -1.26620900 | -2.21261800 | -1.43930800 |
| C | -0.53330100 | -3.34990500 | 0.19737900 |
| H | 0.42490000 | -3.45635500 | -0.31983400 |
| C | 3.15805400 | -1.14964300 | -0.52245500 |
| H | 3.18412000 | -0.81732500 | -1.56655900 |
| C | 4.40815000 | -0.64112200 | 0.19759500 |
| H | 4.42039500 | -1.05307600 | 1.21290200 |
| C | 4.43706800 | 0.89628000 | 0.27011600 |
| H | 5.25162700 | 1.20047900 | 0.94229500 |
| O | 3.06486300 | -2.56738900 | -0.44168800 |
| H | 3.91172800 | -2.92502300 | -0.73697200 |
| O | -0.33079600 | -3.20390800 | 1.60799500 |
| H | 0.21292500 | -3.93788900 | 1.91186900 |
| H | -1.15980000 | -4.22236500 | -0.01931800 |
| O | 5.52643600 | -1.14006700 | -0.54810200 |
| H | 6.29451100 | -1.20157000 | 0.03140400 |
| O | 4.63638600 | 1.44870700 | -1.02457800 |
| H | 5.32604400 | 0.91718400 | -1.44526700 |
| E(RB3LYP): -1470.57927808 | | | |
| Sum of electronic and thermal free energies: -1470.194442 | | | |
| Number of imaginary frequencies: 0 | | | |

## LN **2** Conformer F

| **Atom** | **x** | **y** | **z** |
| --- | --- | --- | --- |
| H | -2.64096000 | -4.40370800 | -0.40002100 |
| O | -2.90629100 | -3.48201100 | -0.48525300 |
| C | -3.06373700 | -2.95102200 | 0.82909800 |
| H | -2.18938300 | -3.19333100 | 1.44406500 |
| H | -3.95217400 | -3.36458700 | 1.32270200 |
| C | -3.20585000 | -1.43671600 | 0.80724000 |
| H | -3.18163000 | -1.12619200 | 1.86300700 |
| O | -2.06530800 | -0.88475500 | 0.13253200 |
| C | -1.93175600 | 0.53307100 | 0.27215200 |
| H | -1.82314700 | 0.78586000 | 1.33791300 |
| O | -0.81074000 | 0.91975100 | -0.44877900 |
| C | 0.47139000 | 0.78650100 | 0.18716300 |
| H | 0.34711400 | 0.70197000 | 1.27299400 |
| C | 1.18069300 | -0.47026000 | -0.33641700 |
| H | 1.10509900 | -0.44205900 | -1.43433300 |
| O | 0.59550300 | -1.66315200 | 0.16289800 |
| H | -0.37201000 | -1.59158600 | 0.06901000 |
| C | 2.66349200 | -0.48496700 | 0.05983800 |
| H | 2.74518600 | -0.62394000 | 1.13842500 |
| N | 3.35318300 | -1.59185300 | -0.57492900 |
| H | 3.29797900 | -1.66402500 | -1.58028100 |
| C | 4.04563800 | -2.54449600 | 0.10017400 |
| O | 4.15265800 | -2.55372700 | 1.32726200 |
| C | 4.69055800 | -3.61783100 | -0.75261900 |
| H | 4.51391900 | -3.49445200 | -1.82186100 |
| H | 5.76632700 | -3.60917700 | -0.56516700 |
| H | 4.30587500 | -4.58967200 | -0.43649300 |
| C | 3.32159300 | 0.85773300 | -0.28708000 |
| H | 3.31735300 | 1.02411500 | -1.37903900 |
| O | 4.61714100 | 0.85990300 | 0.20481900 |
| C | 5.42188400 | 1.94653900 | -0.27094100 |
| H | 5.01116700 | 2.90762500 | 0.04816600 |
| H | 5.49313000 | 1.92318200 | -1.36440000 |
| H | 6.41251400 | 1.80966900 | 0.16004200 |
| O | 2.60132900 | 1.91523500 | 0.34169000 |
| C | 1.26648500 | 2.05766100 | -0.14395600 |
| H | 1.27490400 | 2.19523100 | -1.23488300 |
| C | 0.65526900 | 3.28594700 | 0.49975100 |
| H | 0.82090500 | 3.23587000 | 1.58310900 |
| C | -3.15776300 | 1.22626600 | -0.31476600 |
| H | -3.17841700 | 1.01480100 | -1.38994300 |
| C | -4.43110200 | 0.67563200 | 0.32905600 |
| H | -4.44722800 | 0.97143800 | 1.38393900 |
| C | -4.50104100 | -0.85873300 | 0.22839700 |
| H | -5.33361600 | -1.21312900 | 0.85224300 |
| O | -3.02625100 | 2.62234100 | -0.07277000 |
| H | -3.85781900 | 3.03659600 | -0.33579000 |
| O | 1.27752800 | 4.44832200 | -0.05923300 |
| H | 0.87777100 | 5.22723000 | 0.34197000 |
| H | -0.42094400 | 3.28502000 | 0.30309500 |
| O | -5.52398000 | 1.28576500 | -0.36954100 |
| H | -6.30056300 | 1.29830400 | 0.20175700 |
| O | -4.69340800 | -1.25822100 | -1.12222000 |
| H | -5.36358200 | -0.66562900 | -1.48950100 |
| E(RB3LYP): -1470.57844290 | | | |
| Sum of electronic and thermal free energies: -1470.194018 | | | |
| Number of imaginary frequencies: 0 | | | |

## LN **2** Conformer G

| **Atom** | **x** | **y** | **z** |
| --- | --- | --- | --- |
| H | -4.46596400 | 3.91277300 | -1.87679800 |
| O | -4.40535100 | 2.95448400 | -1.80632300 |
| C | -3.24274600 | 2.62902800 | -1.04209700 |
| H | -3.25352000 | 3.12602900 | -0.06789100 |
| H | -2.32715100 | 2.90802800 | -1.57522500 |
| C | -3.24444300 | 1.12081300 | -0.83923900 |
| H | -3.27196300 | 0.63230700 | -1.82297300 |
| O | -2.00989400 | 0.80451100 | -0.18022600 |
| C | -1.78155100 | -0.60058500 | -0.00926900 |
| H | -1.76848400 | -1.09009700 | -0.99445300 |
| O | -0.56365700 | -0.74626100 | 0.63532400 |
| C | 0.61639500 | -0.68998400 | -0.18557600 |
| H | 0.34178700 | -0.77137600 | -1.24087300 |
| C | 1.34252000 | 0.63499100 | 0.05058300 |
| H | 1.44203600 | 0.77626300 | 1.13247400 |
| O | 0.64015800 | 1.73277300 | -0.52573900 |
| H | -0.30777900 | 1.61581200 | -0.34050300 |
| C | 2.74402800 | 0.60458000 | -0.57942700 |
| H | 2.62480300 | 0.55372000 | -1.66387400 |
| N | 3.49834400 | 1.82293600 | -0.30395800 |
| H | 3.72102100 | 2.40764700 | -1.09315000 |
| C | 3.89287600 | 2.23409000 | 0.92577300 |
| O | 3.66633000 | 1.58446400 | 1.94947900 |
| C | 4.63795400 | 3.55216300 | 0.97883300 |
| H | 4.78728000 | 4.01302600 | 0.00178600 |
| H | 4.07722000 | 4.23979100 | 1.61566100 |
| H | 5.60958000 | 3.38252200 | 1.44719600 |
| C | 3.49999800 | -0.66869600 | -0.16570300 |
| H | 3.67991800 | -0.68304100 | 0.91802200 |
| O | 4.69478000 | -0.71825500 | -0.86920800 |
| C | 5.61384600 | -1.70596500 | -0.38650900 |
| H | 5.20262900 | -2.71246400 | -0.49823600 |
| H | 5.85636000 | -1.52441300 | 0.66675000 |
| H | 6.51664500 | -1.61057900 | -0.98814300 |
| O | 2.72142600 | -1.81242700 | -0.52436800 |
| C | 1.49003600 | -1.89035500 | 0.19525700 |
| H | 1.68551300 | -1.85780500 | 1.27738800 |
| C | 0.81931100 | -3.21795400 | -0.10358800 |
| H | -0.05201800 | -3.31267600 | 0.55147400 |
| C | -2.88268800 | -1.19640300 | 0.86195500 |
| H | -2.81949200 | -0.72943000 | 1.85141300 |
| C | -4.24855900 | -0.89260300 | 0.24616200 |
| H | -4.33262800 | -1.43941600 | -0.70000000 |
| C | -4.43183300 | 0.60792100 | -0.02205600 |
| H | -5.34288700 | 0.75860700 | -0.61406800 |
| O | -2.66224500 | -2.60002900 | 0.94643000 |
| H | -3.43328800 | -2.98278300 | 1.38377800 |
| O | 0.42001700 | -3.25268200 | -1.47936100 |
| H | -0.10234700 | -4.04874100 | -1.62103100 |
| H | 1.52790700 | -4.02393800 | 0.11773800 |
| O | -5.22409600 | -1.37247300 | 1.18107100 |
| H | -6.04583500 | -1.56188600 | 0.71384100 |
| O | -4.51210900 | 1.33662100 | 1.19997200 |
| H | -5.11149100 | 0.84951800 | 1.78040000 |
| E(RB3LYP): -1470.58204920 | | | |
| Sum of electronic and thermal free energies: -1470.197734 | | | |
| Number of imaginary frequencies: 0 | | | |

## LN **2** Conformer H

| **Atom** | **x** | **y** | **z** |
| --- | --- | --- | --- |
| H | -5.87552600 | 2.52225600 | -1.87846500 |
| O | -5.27144300 | 1.77357800 | -1.91494300 |
| C | -4.37700000 | 1.85739600 | -0.80274400 |
| H | -4.92241100 | 1.91723800 | 0.14341500 |
| H | -3.71562000 | 2.72620300 | -0.88878000 |
| C | -3.52194900 | 0.59859200 | -0.81393300 |
| H | -3.01523600 | 0.52823500 | -1.78738500 |
| O | -2.54952900 | 0.77205100 | 0.22384200 |
| C | -1.59338300 | -0.27519900 | 0.27570300 |
| H | -1.06052900 | -0.33011500 | -0.68357700 |
| O | -0.72934500 | 0.00059100 | 1.33698200 |
| C | 0.61250000 | 0.42092000 | 1.04856200 |
| H | 1.05060000 | 0.57981900 | 2.03401800 |
| C | 1.40519300 | -0.68745500 | 0.34100000 |
| H | 0.94317400 | -0.89478200 | -0.63484800 |
| O | 1.42675300 | -1.87483600 | 1.11499000 |
| H | 0.52797400 | -2.24777600 | 1.08266300 |
| C | 2.84534000 | -0.22530000 | 0.08879800 |
| H | 3.36049400 | -0.10309400 | 1.04241100 |
| N | 3.58188500 | -1.20798500 | -0.68159400 |
| H | 3.19794700 | -1.49293500 | -1.57079100 |
| C | 4.73880500 | -1.78558600 | -0.26791000 |
| O | 5.27293500 | -1.52040800 | 0.80979800 |
| C | 5.34905800 | -2.79265900 | -1.22082100 |
| H | 4.76671400 | -2.94532800 | -2.13036500 |
| H | 6.35020200 | -2.45035700 | -1.49137800 |
| H | 5.45363800 | -3.74563900 | -0.69824500 |
| C | 2.83378500 | 1.13990400 | -0.61994400 |
| H | 2.37942400 | 1.04809100 | -1.62288300 |
| O | 4.13711000 | 1.60113500 | -0.71330500 |
| C | 4.30045300 | 2.72607100 | -1.58646600 |
| H | 3.74169200 | 3.59111700 | -1.22087000 |
| H | 3.96876400 | 2.47744500 | -2.60107500 |
| H | 5.36483900 | 2.95568100 | -1.59915000 |
| O | 2.07996000 | 2.08489300 | 0.13698000 |
| C | 0.69542100 | 1.74619600 | 0.27479000 |
| H | 0.24448800 | 1.63525700 | -0.72256500 |
| C | -0.01585300 | 2.89589000 | 0.96256800 |
| H | -1.08855000 | 2.68486000 | 0.94852600 |
| C | -2.28261600 | -1.61245400 | 0.54610500 |
| H | -2.74590200 | -1.56060100 | 1.53666200 |
| C | -3.35143800 | -1.87576600 | -0.50838300 |
| H | -2.85985800 | -2.02314000 | -1.47684600 |
| C | -4.32289700 | -0.69119000 | -0.61177800 |
| H | -4.97003600 | -0.83117000 | -1.48608400 |
| O | -1.27111200 | -2.62254400 | 0.51562000 |
| H | -1.70130900 | -3.48209100 | 0.60579600 |
| O | 0.46683200 | 3.02517100 | 2.30420100 |
| H | -0.06215800 | 3.69399500 | 2.75077400 |
| H | 0.17672300 | 3.81033600 | 0.38963900 |
| O | -4.02917400 | -3.07461400 | -0.11069700 |
| H | -4.43146400 | -3.48459300 | -0.88517700 |
| O | -5.11001600 | -0.57696200 | 0.57105200 |
| H | -5.42755900 | -1.46362900 | 0.78630000 |
| E(RB3LYP): -1470.58028905 | | | |
| Sum of electronic and thermal free energies: -1470.195311 | | | |
| Number of imaginary frequencies: 0 | | | |

## LN **2** Conformer I

| **Atom** | **x** | **y** | **z** |
| --- | --- | --- | --- |
| H | -6.14162000 | 2.33800300 | 1.15783000 |
| O | -5.80882400 | 1.58741200 | 0.65488100 |
| C | -4.39983300 | 1.74460200 | 0.48396600 |
| H | -3.89568400 | 1.86988200 | 1.44706000 |
| H | -4.16833000 | 2.60331000 | -0.15527400 |
| C | -3.89027700 | 0.48227500 | -0.19744100 |
| H | -4.44309800 | 0.34264500 | -1.13591200 |
| O | -2.50413500 | 0.71717300 | -0.49905800 |
| C | -1.89480800 | -0.33184000 | -1.25383500 |
| H | -2.43889600 | -0.47157000 | -2.19516300 |
| O | -0.61528500 | 0.06062100 | -1.64372600 |
| C | 0.37595600 | 0.37318400 | -0.64243000 |
| H | -0.03253100 | 0.26385900 | 0.36456800 |
| C | 1.55554700 | -0.59004100 | -0.82295400 |
| H | 1.83674200 | -0.56092300 | -1.88678500 |
| O | 1.23232900 | -1.91690400 | -0.44062400 |
| H | 0.45849000 | -2.22284700 | -0.94615800 |
| C | 2.75865400 | -0.15648300 | 0.02709600 |
| H | 2.51796600 | -0.29828300 | 1.08149000 |
| N | 3.92009100 | -0.97416700 | -0.26612000 |
| H | 4.23461300 | -1.01672600 | -1.22443200 |
| C | 4.58925700 | -1.70521700 | 0.66142700 |
| O | 4.26809500 | -1.73755200 | 1.85013700 |
| C | 5.77462000 | -2.49543600 | 0.14615800 |
| H | 5.95089900 | -2.37507400 | -0.92349200 |
| H | 6.66552100 | -2.17919500 | 0.69267300 |
| H | 5.60839200 | -3.55240500 | 0.36438900 |
| C | 3.06290600 | 1.33316600 | -0.18346000 |
| H | 3.39708000 | 1.51992800 | -1.21973600 |
| O | 4.04011900 | 1.71730100 | 0.72191600 |
| C | 4.60461600 | 3.00854100 | 0.46101600 |
| H | 3.84652300 | 3.79160100 | 0.54116400 |
| H | 5.05667900 | 3.03685400 | -0.53707700 |
| H | 5.37612100 | 3.16686100 | 1.21305800 |
| O | 1.89202200 | 2.10603300 | 0.06420400 |
| C | 0.82797200 | 1.82691900 | -0.84862200 |
| H | 1.18408100 | 1.94034200 | -1.88402000 |
| C | -0.27191200 | 2.85901300 | -0.62620800 |
| H | -0.99792300 | 2.77278400 | -1.44014200 |
| C | -1.94806700 | -1.62820200 | -0.43684000 |
| H | -1.38301400 | -1.49548100 | 0.49107000 |
| C | -3.39468200 | -1.95933100 | -0.08454700 |
| H | -3.94643100 | -2.17034100 | -1.00757900 |
| C | -4.05744000 | -0.78297300 | 0.64489900 |
| H | -5.13022300 | -0.98167100 | 0.75271600 |
| O | -1.35828600 | -2.66100200 | -1.22969800 |
| H | -1.56341100 | -3.50429200 | -0.80285000 |
| O | -0.91717300 | 2.74044200 | 0.63799000 |
| H | -1.55906800 | 2.02047800 | 0.54371300 |
| H | 0.18256500 | 3.85084000 | -0.66901300 |
| O | -3.34691200 | -3.12820200 | 0.74206900 |
| H | -4.18924300 | -3.59408200 | 0.68545800 |
| O | -3.45904700 | -0.57404600 | 1.92062000 |
| H | -3.40513400 | -1.43458500 | 2.35625000 |
| E(RB3LYP): -1470.58754668 | | | |
| Sum of electronic and thermal free energies: -1470.199427 | | | |
| Number of imaginary frequencies: 0 | | | |

## LN **2** Conformer J

| **Atom** | **x** | **y** | **z** |
| --- | --- | --- | --- |
| H | 1.12510900 | 3.60921700 | -0.41475900 |
| O | 1.82191800 | 3.83088300 | 0.21160700 |
| C | 3.02042200 | 3.15287100 | -0.16992300 |
| H | 3.85422200 | 3.72104500 | 0.24735400 |
| H | 3.13064900 | 3.12407200 | -1.25640400 |
| C | 3.07442900 | 1.73665000 | 0.38541000 |
| H | 2.89249100 | 1.77355600 | 1.46785800 |
| O | 2.00796600 | 0.99467000 | -0.24211900 |
| C | 1.87408300 | -0.34887100 | 0.23320600 |
| H | 1.66476300 | -0.33691000 | 1.31293700 |
| O | 0.83648100 | -0.95279600 | -0.46734600 |
| C | -0.48032500 | -0.91261100 | 0.10481600 |
| H | -0.40651500 | -0.91399600 | 1.19887700 |
| C | -1.22886800 | 0.35140400 | -0.34558700 |
| H | -1.16972100 | 0.39136100 | -1.44356300 |
| O | -0.66860400 | 1.52997800 | 0.21729100 |
| H | 0.28067700 | 1.54469600 | -0.00014600 |
| C | -2.70225900 | 0.30294100 | 0.08078300 |
| H | -2.75957200 | 0.39326400 | 1.16619400 |
| N | -3.44057900 | 1.41453900 | -0.48696200 |
| H | -3.41622800 | 1.53174600 | -1.48934300 |
| C | -4.12259400 | 2.32944700 | 0.24902500 |
| O | -4.18751700 | 2.28820200 | 1.47817600 |
| C | -4.81133200 | 3.42458800 | -0.53891400 |
| H | -4.67012100 | 3.34350800 | -1.61743900 |
| H | -5.87965400 | 3.39360000 | -0.31495700 |
| H | -4.42825200 | 4.38916300 | -0.19944000 |
| C | -3.33170100 | -1.04231500 | -0.30485100 |
| H | -3.38038500 | -1.15221200 | -1.40224800 |
| O | -4.59770100 | -1.11552900 | 0.25298900 |
| C | -5.39476600 | -2.19959300 | -0.24190200 |
| H | -4.94053600 | -3.16317200 | 0.00182800 |
| H | -5.52367600 | -2.11862600 | -1.32723000 |
| H | -6.36493500 | -2.11774700 | 0.24574600 |
| O | -2.54423000 | -2.10714700 | 0.22606100 |
| C | -1.22575700 | -2.17634000 | -0.33569900 |
| H | -1.31553900 | -2.22186700 | -1.42969100 |
| C | -0.66033000 | -3.50688600 | 0.18089100 |
| H | -1.34876400 | -4.29393100 | -0.13166500 |
| C | 3.15673800 | -1.12210100 | -0.04574800 |
| H | 3.29668200 | -1.17241500 | -1.13056400 |
| C | 4.34747300 | -0.41410200 | 0.59215100 |
| H | 4.23784000 | -0.45384500 | 1.68141300 |
| C | 4.42280800 | 1.05424400 | 0.14488900 |
| H | 5.17867900 | 1.56952000 | 0.75280300 |
| O | 2.97623700 | -2.42633800 | 0.50324100 |
| H | 3.79838700 | -2.91881500 | 0.38451700 |
| O | 0.62066800 | -3.85365500 | -0.31797400 |
| H | 1.29988100 | -3.23892500 | 0.00496300 |
| H | -0.66413200 | -3.47396600 | 1.27911300 |
| O | 5.51304700 | -1.13749100 | 0.18238600 |
| H | 6.21421300 | -1.00848700 | 0.83143700 |
| O | 4.75426300 | 1.14696600 | -1.23749700 |
| H | 5.49015600 | 0.54048200 | -1.39467100 |
| E(RB3LYP): -1470.58865009 | | | |
| Sum of electronic and thermal free energies: -1470.202004 | | | |
| Number of imaginary frequencies: 0 | | | |

## LN **2** Conformer K

| **Atom** | **x** | **y** | **z** |
| --- | --- | --- | --- |
| H | 4.14318900 | 4.60243900 | 0.16109800 |
| O | 4.08277900 | 3.71814900 | 0.53712900 |
| C | 3.06560600 | 2.99741200 | -0.16009300 |
| H | 3.24849600 | 2.98920900 | -1.23825000 |
| H | 2.07559900 | 3.42646000 | 0.03041300 |
| C | 3.08783200 | 1.56967400 | 0.36685100 |
| H | 2.94204700 | 1.59189000 | 1.45538200 |
| O | 1.97536000 | 0.89882900 | -0.25202000 |
| C | 1.77703400 | -0.43957600 | 0.21416700 |
| H | 1.57870000 | -0.42410200 | 1.29587600 |
| O | 0.70299200 | -0.98425500 | -0.48018200 |
| C | -0.60593900 | -0.87494800 | 0.10117400 |
| H | -0.52243900 | -0.84998000 | 1.19416900 |
| C | -1.30543800 | 0.40653000 | -0.37939500 |
| H | -1.26083200 | 0.40825100 | -1.47891500 |
| O | -0.69062100 | 1.57786400 | 0.13679400 |
| H | 0.25011800 | 1.56445700 | -0.11305200 |
| C | -2.77365400 | 0.43463300 | 0.06806400 |
| H | -2.81031700 | 0.56119500 | 1.15074700 |
| N | -3.47450600 | 1.55767200 | -0.52398500 |
| H | -3.47097100 | 1.63565800 | -1.53043200 |
| C | -4.10448100 | 2.52416300 | 0.19197800 |
| O | -4.14273400 | 2.53034000 | 1.42301600 |
| C | -4.76948700 | 3.61595000 | -0.62078500 |
| H | -4.65692300 | 3.49074400 | -1.69844400 |
| H | -5.83271500 | 3.63407600 | -0.37243000 |
| H | -4.34270600 | 4.57680100 | -0.32581500 |
| C | -3.46658600 | -0.89294300 | -0.26602500 |
| H | -3.53943100 | -1.03280900 | -1.35885400 |
| O | -4.72484800 | -0.89461800 | 0.31393000 |
| C | -5.57526000 | -1.95828200 | -0.13400800 |
| H | -5.15897800 | -2.93235300 | 0.13447700 |
| H | -5.71741600 | -1.90664300 | -1.21950200 |
| H | -6.53335200 | -1.81898800 | 0.36438400 |
| O | -2.71639600 | -1.97451500 | 0.28349500 |
| C | -1.41179400 | -2.11556600 | -0.29827900 |
| H | -1.52526000 | -2.18123900 | -1.38908700 |
| C | -0.90103900 | -3.46042700 | 0.23713900 |
| H | -1.63336700 | -4.21884300 | -0.04503300 |
| C | 3.01419500 | -1.27923800 | -0.08243200 |
| H | 3.13131300 | -1.34194400 | -1.16920300 |
| C | 4.25393600 | -0.63463200 | 0.52870800 |
| H | 4.16581100 | -0.66917400 | 1.62028300 |
| C | 4.39381200 | 0.82676000 | 0.07893600 |
| H | 5.19097600 | 1.30702900 | 0.65901300 |
| O | 2.77521500 | -2.57019800 | 0.47661300 |
| H | 3.56846800 | -3.10536800 | 0.34655200 |
| O | 0.35109100 | -3.87985100 | -0.27778700 |
| H | 1.06747700 | -3.29673900 | 0.02334400 |
| H | -0.88179700 | -3.40433100 | 1.33432300 |
| O | 5.36947500 | -1.42137900 | 0.09501900 |
| H | 6.09364500 | -1.32198000 | 0.72357100 |
| O | 4.67984000 | 0.90337700 | -1.31441900 |
| H | 5.38256900 | 0.26556900 | -1.49542300 |
| E(RB3LYP): -1470.58695239 | | | |
| Sum of electronic and thermal free energies: -1470.200291 | | | |
| Number of imaginary frequencies: 0 | | | |

## LN **2** Conformer L

| **Atom** | **x** | **y** | **z** |
| --- | --- | --- | --- |
| H | 1.82325900 | 4.08923100 | 1.27724300 |
| O | 2.21796000 | 3.34389700 | 0.81294600 |
| C | 2.74548400 | 2.44965300 | 1.79198400 |
| H | 2.00790900 | 2.28089200 | 2.58422300 |
| H | 3.65706900 | 2.85196000 | 2.25128300 |
| C | 3.07943100 | 1.09769900 | 1.17791600 |
| H | 3.29204500 | 0.42997300 | 2.02683300 |
| O | 1.91603300 | 0.62806600 | 0.49187000 |
| C | 1.97012700 | -0.73917300 | 0.07248200 |
| H | 2.07871600 | -1.39282400 | 0.94939400 |
| O | 0.78197400 | -0.99792700 | -0.60005400 |
| C | -0.43089900 | -0.84910800 | 0.16486000 |
| H | -0.20015200 | -0.58189900 | 1.20068400 |
| C | -1.20594400 | 0.31089800 | -0.48238700 |
| H | -1.15757200 | 0.14535500 | -1.56971800 |
| O | -0.63492200 | 1.57303700 | -0.16293100 |
| H | 0.32653500 | 1.46813700 | -0.04761900 |
| C | -2.67993900 | 0.34728900 | -0.06483900 |
| H | -2.75662200 | 0.62650100 | 0.98636800 |
| N | -3.40389900 | 1.34117000 | -0.83535900 |
| H | -3.35070600 | 1.28453800 | -1.84202100 |
| C | -4.11962000 | 2.35581000 | -0.28763100 |
| O | -4.22427100 | 2.52392300 | 0.92831000 |
| C | -4.79143800 | 3.29279700 | -1.27052100 |
| H | -4.64775000 | 3.01073300 | -2.31434000 |
| H | -5.86055300 | 3.31747100 | -1.05048100 |
| H | -4.39676300 | 4.29928400 | -1.11526500 |
| C | -3.29322300 | -1.04813900 | -0.22112900 |
| H | -3.25833300 | -1.38127300 | -1.27320600 |
| O | -4.59784300 | -1.02483300 | 0.24404100 |
| C | -5.35434800 | -2.19982400 | -0.07518700 |
| H | -4.92003100 | -3.08564900 | 0.39496400 |
| H | -5.39956000 | -2.34559000 | -1.16054600 |
| H | -6.35921800 | -2.03534500 | 0.31078800 |
| O | -2.55155400 | -1.96763000 | 0.57660400 |
| C | -1.20693000 | -2.17708500 | 0.14091200 |
| H | -1.19055300 | -2.57424700 | -0.88327100 |
| C | -0.67373100 | -3.22278900 | 1.11715400 |
| H | -1.33290200 | -4.09665500 | 1.06697500 |
| C | 3.13717700 | -0.91572500 | -0.89739800 |
| H | 2.92548800 | -0.29686300 | -1.77720200 |
| C | 4.43261300 | -0.42544900 | -0.24477200 |
| H | 4.67454500 | -1.08746700 | 0.59410800 |
| C | 4.30618300 | 1.02081200 | 0.26483600 |
| H | 5.19269700 | 1.25943400 | 0.86930000 |
| O | 3.24549400 | -2.28681500 | -1.25797800 |
| H | 4.06180500 | -2.37787800 | -1.76582900 |
| O | 0.66674800 | -3.56623600 | 0.77488200 |
| H | 0.97684000 | -4.22413400 | 1.40592900 |
| H | -0.72852300 | -2.80753000 | 2.13114000 |
| O | 5.44567700 | -0.52259300 | -1.25554400 |
| H | 6.30746300 | -0.61925000 | -0.83442400 |
| O | 4.19887000 | 1.92474300 | -0.82808800 |
| H | 4.85916400 | 1.64787300 | -1.47794600 |
| E(RB3LYP): -1470.57318481 | | | |
| Sum of electronic and thermal free energies:-1470.188573 | | | |
| Number of imaginary frequencies: 0 | | | |

## 3F-LN **3** Conformer A

| **Atom** | **x** | **y** | **z** |
| --- | --- | --- | --- |
| H | 3.89725300 | 4.45444400 | -0.90683400 |
| O | 3.85519000 | 3.68274500 | -0.33280400 |
| C | 2.92496500 | 2.75161300 | -0.89205200 |
| H | 3.18517100 | 2.50128400 | -1.92452300 |
| H | 1.90371400 | 3.14671400 | -0.86740800 |
| C | 2.97209700 | 1.49255400 | -0.03916000 |
| H | 2.74698700 | 1.76658900 | 1.00158700 |
| O | 1.94477500 | 0.63327100 | -0.54329000 |
| C | 1.78156400 | -0.54015900 | 0.24058200 |
| H | 1.53138100 | -0.26992300 | 1.27595000 |
| O | 0.75939400 | -1.30051100 | -0.33427600 |
| C | -0.57355700 | -1.09791100 | 0.14443300 |
| H | -0.63416400 | -1.37035200 | 1.20365800 |
| C | -1.06786300 | 0.33614100 | -0.01914000 |
| H | -0.73644400 | 0.73646600 | -0.97998400 |
| C | -2.58268300 | 0.46356400 | 0.15938000 |
| H | -2.82931700 | 0.26431200 | 1.20306000 |
| N | -3.02558800 | 1.80908700 | -0.14149500 |
| H | -2.85991300 | 2.16738900 | -1.07076600 |
| C | -3.68214100 | 2.60203300 | 0.74857500 |
| O | -3.93225900 | 2.24652200 | 1.89942700 |
| C | -4.09053100 | 3.96660100 | 0.23602500 |
| H | -3.78986500 | 4.15539000 | -0.79514900 |
| H | -5.17641400 | 4.05334400 | 0.31291700 |
| H | -3.64918700 | 4.72704700 | 0.88324700 |
| C | -3.31556200 | -0.58857400 | -0.68536100 |
| H | -3.15348000 | -0.41765700 | -1.76346700 |
| O | -4.66164500 | -0.54029500 | -0.37366500 |
| C | -5.49818800 | -1.29108500 | -1.26541100 |
| H | -5.25527600 | -2.35564200 | -1.22536900 |
| H | -5.38919900 | -0.92691500 | -2.29302900 |
| H | -6.52246700 | -1.13512500 | -0.93105400 |
| O | -2.81692100 | -1.88222300 | -0.34794900 |
| C | -1.44081400 | -2.06743700 | -0.67633300 |
| H | -1.27221500 | -1.87530600 | -1.74521700 |
| C | -1.11967200 | -3.52901000 | -0.39101500 |
| H | -1.75704200 | -4.15153300 | -1.02886500 |
| H | -0.07759000 | -3.72556600 | -0.64070500 |
| O | -1.28867300 | -3.86508000 | 0.98470600 |
| H | -2.20898400 | -3.69332500 | 1.21877500 |
| C | 3.06531000 | -1.36947900 | 0.20125400 |
| H | 3.25113300 | -1.65965100 | -0.83840000 |
| C | 4.23104600 | -0.53018200 | 0.71468700 |
| H | 4.05627000 | -0.30615600 | 1.77555700 |
| O | 5.47490200 | -1.21521200 | 0.56172600 |
| H | 5.35765700 | -2.11923000 | 0.87942800 |
| C | 4.33415500 | 0.79029400 | -0.05394500 |
| H | 5.06160300 | 1.43787800 | 0.44862400 |
| O | 4.73010900 | 0.56460200 | -1.40603200 |
| H | 5.46432800 | -0.06245500 | -1.38357700 |
| O | 2.97422300 | -2.51677500 | 1.04298300 |
| H | 2.31713500 | -3.11544000 | 0.66920700 |
| F | -0.46927600 | 1.14663000 | 0.97456400 |
| E(RB3LYP): -1494.60345697 | | | |
| Sum of electronic and thermal free energies: -1494.230878 | | | |
| Number of imaginary frequencies: 0 | | | |

## 3F-LN **3** Conformer B

| **Atom** | **x** | **y** | **z** |
| --- | --- | --- | --- |
| H | 3.88197200 | 4.35540500 | -1.20723700 |
| O | 3.86084100 | 3.62558000 | -0.57969500 |
| C | 2.90945100 | 2.66144400 | -1.03828500 |
| H | 3.13292900 | 2.33675400 | -2.05856400 |
| H | 1.89065500 | 3.06234200 | -1.00684000 |
| C | 2.98228000 | 1.46613400 | -0.09987300 |
| H | 2.79164300 | 1.81438400 | 0.92556100 |
| O | 1.93740400 | 0.57798100 | -0.50887600 |
| C | 1.79480400 | -0.53458300 | 0.36241100 |
| H | 1.58246900 | -0.18905800 | 1.38389000 |
| O | 0.74875300 | -1.32703500 | -0.11975600 |
| C | -0.56958100 | -1.06586900 | 0.37153900 |
| H | -0.60416900 | -1.23329400 | 1.45509400 |
| C | -1.04030400 | 0.36066400 | 0.08948200 |
| H | -0.70069400 | 0.66507800 | -0.90305000 |
| C | -2.55032200 | 0.54846600 | 0.25111200 |
| H | -2.80281000 | 0.47506600 | 1.30956400 |
| N | -2.95133100 | 1.86436900 | -0.20231500 |
| H | -2.78355200 | 2.10183900 | -1.16935500 |
| C | -3.58062100 | 2.77509500 | 0.58900000 |
| O | -3.83614800 | 2.56388700 | 1.77375400 |
| C | -3.95512400 | 4.08033000 | -0.08017900 |
| H | -3.59766900 | 4.16506400 | -1.10704100 |
| H | -5.04376000 | 4.17081500 | -0.07398700 |
| H | -3.54931200 | 4.90297700 | 0.51124000 |
| C | -3.31168900 | -0.56973400 | -0.47404200 |
| H | -3.15174000 | -0.51677000 | -1.56489200 |
| O | -4.65563800 | -0.45452000 | -0.16628200 |
| C | -5.51237000 | -1.27941000 | -0.96878000 |
| H | -5.28850500 | -2.33814000 | -0.81771400 |
| H | -5.40428400 | -1.02821400 | -2.02986000 |
| H | -6.53124600 | -1.06958300 | -0.64750300 |
| O | -2.84141700 | -1.82739300 | 0.00222800 |
| C | -1.48194400 | -2.08402700 | -0.33894100 |
| H | -1.33964200 | -1.98500200 | -1.42419000 |
| C | -1.15861300 | -3.50893500 | 0.06726400 |
| H | -0.07855000 | -3.65976600 | -0.01218200 |
| H | -1.46328700 | -3.65779400 | 1.11022200 |
| O | -1.86257600 | -4.39349900 | -0.80717900 |
| H | -1.66073700 | -5.29937700 | -0.54972700 |
| C | 3.07308300 | -1.37300300 | 0.33655200 |
| H | 3.22231000 | -1.73340700 | -0.68675200 |
| C | 4.25827000 | -0.50656100 | 0.75188000 |
| H | 4.11960000 | -0.20927900 | 1.79997600 |
| O | 5.49368400 | -1.20769900 | 0.60545400 |
| H | 5.38358500 | -2.08723300 | 0.98794500 |
| C | 4.34077500 | 0.75711200 | -0.10945400 |
| H | 5.08669100 | 1.43378000 | 0.32289100 |
| O | 4.69113100 | 0.43534000 | -1.45452700 |
| H | 5.42360700 | -0.19278400 | -1.41221800 |
| O | 3.00874700 | -2.46029400 | 1.25662700 |
| H | 2.35495500 | -3.09186000 | 0.93525900 |
| F | -0.41755200 | 1.23732800 | 1.00817400 |
| E(RB3LYP): -1494.59998689 | | | |
| Sum of electronic and thermal free energies: -1494.229006 | | | |
| Number of imaginary frequencies: 0 | | | |

## 3F-LN **3** Conformer C

| **Atom** | **x** | **y** | **z** |
| --- | --- | --- | --- |
| H | -1.13785300 | -2.79622200 | 0.97756400 |
| O | -1.81850000 | -3.29816000 | 1.44828300 |
| C | -3.07451700 | -2.94105400 | 0.86825900 |
| H | -3.85505500 | -3.39498900 | 1.48104300 |
| H | -3.15727300 | -3.32857800 | -0.15177100 |
| C | -3.24428000 | -1.42858500 | 0.84708600 |
| H | -3.19706500 | -1.04972400 | 1.87805600 |
| O | -2.11198700 | -0.94054600 | 0.11510600 |
| C | -2.01521600 | 0.46966000 | 0.10155700 |
| H | -1.97105100 | 0.85387100 | 1.13382600 |
| O | -0.86470100 | 0.82309500 | -0.60378700 |
| C | 0.37773600 | 0.68492800 | 0.09754500 |
| H | 0.20036900 | 0.43293300 | 1.14806500 |
| C | 1.21139400 | -0.42397100 | -0.53546400 |
| H | 1.14003100 | -0.34598700 | -1.62397800 |
| C | 2.67073700 | -0.42017600 | -0.07578500 |
| H | 2.71051500 | -0.71139800 | 0.97469800 |
| N | 3.44867200 | -1.38563100 | -0.82414000 |
| H | 3.50993000 | -1.27193300 | -1.82557000 |
| C | 4.13414100 | -2.40948900 | -0.24632100 |
| O | 4.12395200 | -2.61683200 | 0.96623100 |
| C | 4.91795500 | -3.29341100 | -1.19285700 |
| H | 4.82622200 | -3.00460400 | -2.24048500 |
| H | 5.97056300 | -3.25986000 | -0.90395500 |
| H | 4.57272400 | -4.32221600 | -1.07279400 |
| C | 3.25760200 | 0.99674800 | -0.17625800 |
| H | 3.30683100 | 1.33216200 | -1.22648100 |
| O | 4.51640700 | 0.98945700 | 0.39784300 |
| C | 5.28098200 | 2.17703800 | 0.14592600 |
| H | 4.79429800 | 3.05431600 | 0.57883000 |
| H | 5.41646400 | 2.32559800 | -0.93121600 |
| H | 6.25076000 | 2.02487800 | 0.61678300 |
| O | 2.42891000 | 1.89478100 | 0.55598900 |
| C | 1.12045400 | 2.03220400 | 0.00582700 |
| H | 1.18225600 | 2.32817000 | -1.05107600 |
| C | 0.40153000 | 3.12221600 | 0.77629900 |
| H | -0.66192100 | 3.09566300 | 0.52063500 |
| H | 0.51263600 | 2.92889900 | 1.85013000 |
| O | 0.97898300 | 4.37947000 | 0.41700100 |
| H | 0.52085900 | 5.07380000 | 0.90249800 |
| C | -3.22893800 | 1.05005100 | -0.62337300 |
| H | -3.22474100 | 0.67150900 | -1.65058500 |
| C | -4.50358000 | 0.59007900 | 0.08488800 |
| H | -4.51726800 | 1.02913600 | 1.09151000 |
| O | -5.66810000 | 0.98939300 | -0.63578800 |
| H | -5.55841700 | 1.91482600 | -0.88850800 |
| C | -4.55003400 | -0.93878700 | 0.21694900 |
| H | -5.38291000 | -1.20545300 | 0.88038200 |
| O | -4.72043200 | -1.55607200 | -1.05697700 |
| H | -5.41205000 | -1.06411600 | -1.51863700 |
| O | -3.23333800 | 2.47559600 | -0.60241200 |
| H | -2.56540600 | 2.79237500 | -1.22119300 |
| F | 0.66672000 | -1.68108900 | -0.19293000 |
| E(RB3LYP): -1494.60435144 | | | |
| Sum of electronic and thermal free energies: -1494.231784 | | | |
| Number of imaginary frequencies: 0 | | | |

## 3F-LN **3** Conformer D

| **Atom** | **x** | **y** | **z** |
| --- | --- | --- | --- |
| H | 0.85317000 | 2.84731700 | 0.02997200 |
| O | 1.50296800 | 3.55588700 | 0.13793300 |
| C | 2.76829500 | 3.05106200 | -0.29424200 |
| H | 3.53280800 | 3.73306200 | 0.08241200 |
| H | 2.82860500 | 3.02517300 | -1.38720400 |
| C | 3.00595500 | 1.65483600 | 0.25382100 |
| H | 2.85496600 | 1.68116600 | 1.34283000 |
| O | 1.99518500 | 0.82830100 | -0.34171100 |
| C | 1.94663100 | -0.47039000 | 0.22487300 |
| H | 1.77808900 | -0.40508300 | 1.31076000 |
| O | 0.91558300 | -1.18164200 | -0.39128900 |
| C | -0.40168600 | -1.05349700 | 0.15375300 |
| H | -0.37940400 | -1.22081400 | 1.23596800 |
| C | -1.03208500 | 0.30934800 | -0.12220700 |
| H | -0.75832800 | 0.64295700 | -1.12586500 |
| C | -2.54803000 | 0.33039600 | 0.08487100 |
| H | -2.75662100 | 0.22880300 | 1.15070500 |
| N | -3.10455100 | 1.59517400 | -0.34832600 |
| H | -2.97860800 | 1.86297200 | -1.31383600 |
| C | -3.80837500 | 2.42633700 | 0.46795400 |
| O | -4.01356100 | 2.17658500 | 1.65482200 |
| C | -4.33024300 | 3.69303700 | -0.17571900 |
| H | -4.04231500 | 3.80382800 | -1.22171000 |
| H | -5.42002000 | 3.69317500 | -0.10364800 |
| H | -3.95905000 | 4.54872700 | 0.39151700 |
| C | -3.20760500 | -0.86394100 | -0.61804700 |
| H | -3.08578500 | -0.80182800 | -1.71309400 |
| O | -4.54531500 | -0.89194800 | -0.27047700 |
| C | -5.33645400 | -1.79867400 | -1.05209800 |
| H | -5.00490600 | -2.82988300 | -0.90859000 |
| H | -5.28144300 | -1.53850500 | -2.11496900 |
| H | -6.36226900 | -1.69080400 | -0.70385200 |
| O | -2.59189800 | -2.06502700 | -0.15620800 |
| C | -1.21545400 | -2.17771100 | -0.51325800 |
| H | -1.09409100 | -2.10353400 | -1.60310800 |
| C | -0.76961400 | -3.56486400 | -0.06759000 |
| H | -1.37053500 | -4.30766200 | -0.60377000 |
| H | 0.27762300 | -3.71124700 | -0.32868600 |
| O | -0.87503600 | -3.74236400 | 1.34308500 |
| H | -1.80005900 | -3.61543300 | 1.58719800 |
| C | 3.26562800 | -1.18498200 | -0.07714400 |
| H | 3.36075000 | -1.26364200 | -1.16540000 |
| C | 4.43495700 | -0.37234900 | 0.47796400 |
| H | 4.36236100 | -0.36891800 | 1.57382400 |
| O | 5.68680600 | -0.92612700 | 0.07515000 |
| H | 5.64913000 | -1.88056300 | 0.21588000 |
| C | 4.39650100 | 1.08005600 | -0.01538200 |
| H | 5.13453500 | 1.66039700 | 0.55352400 |
| O | 4.67801300 | 1.16102400 | -1.41030400 |
| H | 5.44165700 | 0.59353400 | -1.57824600 |
| O | 3.33165300 | -2.47026600 | 0.53541300 |
| H | 2.71940000 | -3.05715600 | 0.07764100 |
| F | -0.49483400 | 1.26670800 | 0.77571400 |
| E(RB3LYP): -1494.60767110 | | | |
| Sum of electronic and thermal free energies: -1494.234537 | | | |
| Number of imaginary frequencies: 0 | | | |

## 3F-LN **3** Conformer E

| **Atom** | **x** | **y** | **z** |
| --- | --- | --- | --- |
| H | 2.68720000 | 3.86666800 | -2.16815700 |
| O | 2.95124600 | 3.00018400 | -1.84155700 |
| C | 2.81574900 | 3.01371800 | -0.42048100 |
| H | 1.82755500 | 3.39353000 | -0.13759800 |
| H | 3.57536100 | 3.65709500 | 0.04174600 |
| C | 2.95608500 | 1.62395000 | 0.17997500 |
| H | 2.73926500 | 1.75701300 | 1.25230100 |
| O | 1.96009900 | 0.77139800 | -0.39381800 |
| C | 1.83150800 | -0.45591300 | 0.30758100 |
| H | 1.55718400 | -0.26522500 | 1.35505800 |
| O | 0.84590500 | -1.21538800 | -0.33031500 |
| C | -0.49943300 | -1.08965600 | 0.13949200 |
| H | -0.56196600 | -1.41764200 | 1.18272300 |
| C | -1.04664000 | 0.33082500 | 0.03992500 |
| H | -0.71587600 | 0.79062400 | -0.89404900 |
| C | -2.56783600 | 0.39444900 | 0.19836400 |
| H | -2.82162000 | 0.14552800 | 1.22966400 |
| N | -3.05517800 | 1.73378600 | -0.05846100 |
| H | -2.88967700 | 2.13074600 | -0.97195800 |
| C | -3.75740900 | 2.46705500 | 0.84764600 |
| O | -4.01437700 | 2.05871000 | 1.97930900 |
| C | -4.20935200 | 3.83361100 | 0.37861600 |
| H | -3.87891500 | 4.08242300 | -0.63051400 |
| H | -5.30040300 | 3.86741800 | 0.41514700 |
| H | -3.82966600 | 4.58291300 | 1.07585000 |
| C | -3.25224000 | -0.64785300 | -0.69796600 |
| H | -3.09292200 | -0.42167500 | -1.76611700 |
| O | -4.60094500 | -0.66737100 | -0.39362200 |
| C | -5.40235300 | -1.40736200 | -1.32585800 |
| H | -5.11832400 | -2.46243900 | -1.33368100 |
| H | -5.30158600 | -0.99142000 | -2.33449500 |
| H | -6.43389300 | -1.30706300 | -0.99251300 |
| O | -2.70424000 | -1.93468300 | -0.41694300 |
| C | -1.31911300 | -2.04844200 | -0.74010400 |
| H | -1.14916900 | -1.79389500 | -1.79563300 |
| C | -0.94270600 | -3.50925500 | -0.52804400 |
| H | -1.54599500 | -4.12162400 | -1.20750000 |
| H | 0.10955100 | -3.65054700 | -0.77230100 |
| O | -1.11581300 | -3.92488000 | 0.82517500 |
| H | -2.04512300 | -3.80129500 | 1.05413500 |
| C | 3.14504200 | -1.23524600 | 0.23628600 |
| H | 3.35358300 | -1.45358700 | -0.81655100 |
| C | 4.27569900 | -0.39056300 | 0.81558500 |
| H | 4.08195100 | -0.23395000 | 1.88487500 |
| O | 5.54227300 | -1.02664400 | 0.64138700 |
| H | 5.44498200 | -1.95546700 | 0.88603900 |
| C | 4.34582500 | 0.97440900 | 0.11661500 |
| H | 5.04437600 | 1.61406700 | 0.67260900 |
| O | 4.78687100 | 0.81983100 | -1.22810000 |
| H | 5.51761300 | 0.18740600 | -1.20357300 |
| O | 3.08593900 | -2.43527800 | 1.00433200 |
| H | 2.45973100 | -3.03487800 | 0.58221200 |
| F | -0.49551000 | 1.11215500 | 1.08321600 |
| E(RB3LYP): -1494.59866034 | | | |
| Sum of electronic and thermal free energies: -1494.225323 | | | |
| Number of imaginary frequencies: 0 | | | |

## 3F-LN **3** Conformer F

| **Atom** | **x** | **y** | **z** |
| --- | --- | --- | --- |
| H | 2.55893800 | -3.57872200 | 2.52935300 |
| O | 2.84716100 | -2.75851600 | 2.11551100 |
| C | 2.77336100 | -2.93732800 | 0.70140200 |
| H | 1.79563200 | -3.34550300 | 0.42190000 |
| H | 3.54776700 | -3.63112500 | 0.34983100 |
| C | 2.94750700 | -1.62526000 | -0.04706200 |
| H | 2.76083100 | -1.87513200 | -1.10399800 |
| O | 1.94436500 | -0.70784100 | 0.39896900 |
| C | 1.84640400 | 0.43402100 | -0.43824400 |
| H | 1.60537300 | 0.12923700 | -1.46662700 |
| O | 0.84534100 | 1.26248600 | 0.07937900 |
| C | -0.49201200 | 1.06971900 | -0.39177400 |
| H | -0.54261300 | 1.28046500 | -1.46716300 |
| C | -1.01249600 | -0.34838300 | -0.15676000 |
| H | -0.66452600 | -0.70809900 | 0.81398100 |
| C | -2.53161000 | -0.46938600 | -0.29272300 |
| H | -2.80338400 | -0.33349200 | -1.34012700 |
| N | -2.97589800 | -1.78995900 | 0.10344600 |
| H | -2.78415800 | -2.09113800 | 1.04808100 |
| C | -3.66514200 | -2.62950500 | -0.71620500 |
| O | -3.94692100 | -2.34245700 | -1.87892100 |
| C | -4.06979700 | -3.95618500 | -0.10972800 |
| H | -3.73302900 | -4.08950700 | 0.91888800 |
| H | -5.15881100 | -4.03189300 | -0.14131800 |
| H | -3.66244300 | -4.75887900 | -0.72751000 |
| C | -3.23022400 | 0.64302600 | 0.50154200 |
| H | -3.04184600 | 0.53982400 | 1.58439300 |
| O | -4.58559500 | 0.59071200 | 0.22901200 |
| C | -5.38718100 | 1.41610000 | 1.08603300 |
| H | -5.12966500 | 2.47100000 | 0.96467900 |
| H | -5.25634000 | 1.12372100 | 2.13393000 |
| H | -6.42222800 | 1.25386300 | 0.78984700 |
| O | -2.72497200 | 1.90081200 | 0.06251700 |
| C | -1.34878400 | 2.09362000 | 0.37767200 |
| H | -1.18393800 | 1.94924300 | 1.45464100 |
| C | -0.98153900 | 3.51957300 | 0.01410300 |
| H | 0.10468700 | 3.62874600 | 0.07567400 |
| H | -1.30140700 | 3.71553900 | -1.01654500 |
| O | -1.63508400 | 4.39798500 | 0.93285300 |
| H | -1.40610500 | 5.30438800 | 0.70140300 |
| C | 3.16293100 | 1.21065200 | -0.40892300 |
| H | 3.33996300 | 1.54079700 | 0.62021400 |
| C | 4.30341100 | 0.30116600 | -0.85699400 |
| H | 4.14115900 | 0.03167800 | -1.90889000 |
| O | 5.56955400 | 0.94514500 | -0.71237500 |
| H | 5.48876300 | 1.84218100 | -1.06005500 |
| C | 4.34085100 | -0.98154600 | -0.01382800 |
| H | 5.04861400 | -1.68143100 | -0.47803200 |
| O | 4.74622400 | -0.68684500 | 1.31847200 |
| H | 5.48244200 | -0.06431100 | 1.24739700 |
| O | 3.14000500 | 2.32049900 | -1.30400500 |
| H | 2.51611100 | 2.97204300 | -0.96360300 |
| F | -0.44479900 | -1.20903600 | -1.12520400 |
| E(RB3LYP): -1494.59519307 | | | |
| Sum of electronic and thermal free energies: -1494.224152 | | | |
| Number of imaginary frequencies: 0 | | | |

## 3F-LN **3** Conformer G

| **Atom** | **x** | **y** | **z** |
| --- | --- | --- | --- |
| H | 2.55893800 | -3.57872200 | 2.52935300 |
| O | 2.84716100 | -2.75851600 | 2.11551100 |
| C | 2.77336100 | -2.93732800 | 0.70140200 |
| H | 1.79563200 | -3.34550300 | 0.42190000 |
| H | 3.54776700 | -3.63112500 | 0.34983100 |
| C | 2.94750700 | -1.62526000 | -0.04706200 |
| H | 2.76083100 | -1.87513200 | -1.10399800 |
| O | 1.94436500 | -0.70784100 | 0.39896900 |
| C | 1.84640400 | 0.43402100 | -0.43824400 |
| H | 1.60537300 | 0.12923700 | -1.46662700 |
| O | 0.84534100 | 1.26248600 | 0.07937900 |
| C | -0.49201200 | 1.06971900 | -0.39177400 |
| H | -0.54261300 | 1.28046500 | -1.46716300 |
| C | -1.01249600 | -0.34838300 | -0.15676000 |
| H | -0.66452600 | -0.70809900 | 0.81398100 |
| C | -2.53161000 | -0.46938600 | -0.29272300 |
| H | -2.80338400 | -0.33349200 | -1.34012700 |
| N | -2.97589800 | -1.78995900 | 0.10344600 |
| H | -2.78415800 | -2.09113800 | 1.04808100 |
| C | -3.66514200 | -2.62950500 | -0.71620500 |
| O | -3.94692100 | -2.34245700 | -1.87892100 |
| C | -4.06979700 | -3.95618500 | -0.10972800 |
| H | -3.73302900 | -4.08950700 | 0.91888800 |
| H | -5.15881100 | -4.03189300 | -0.14131800 |
| H | -3.66244300 | -4.75887900 | -0.72751000 |
| C | -3.23022400 | 0.64302600 | 0.50154200 |
| H | -3.04184600 | 0.53982400 | 1.58439300 |
| O | -4.58559500 | 0.59071200 | 0.22901200 |
| C | -5.38718100 | 1.41610000 | 1.08603300 |
| H | -5.12966500 | 2.47100000 | 0.96467900 |
| H | -5.25634000 | 1.12372100 | 2.13393000 |
| H | -6.42222800 | 1.25386300 | 0.78984700 |
| O | -2.72497200 | 1.90081200 | 0.06251700 |
| C | -1.34878400 | 2.09362000 | 0.37767200 |
| H | -1.18393800 | 1.94924300 | 1.45464100 |
| C | -0.98153900 | 3.51957300 | 0.01410300 |
| H | 0.10468700 | 3.62874600 | 0.07567400 |
| H | -1.30140700 | 3.71553900 | -1.01654500 |
| O | -1.63508400 | 4.39798500 | 0.93285300 |
| H | -1.40610500 | 5.30438800 | 0.70140300 |
| C | 3.16293100 | 1.21065200 | -0.40892300 |
| H | 3.33996300 | 1.54079700 | 0.62021400 |
| C | 4.30341100 | 0.30116600 | -0.85699400 |
| H | 4.14115900 | 0.03167800 | -1.90889000 |
| O | 5.56955400 | 0.94514500 | -0.71237500 |
| H | 5.48876300 | 1.84218100 | -1.06005500 |
| C | 4.34085100 | -0.98154600 | -0.01382800 |
| H | 5.04861400 | -1.68143100 | -0.47803200 |
| O | 4.74622400 | -0.68684500 | 1.31847200 |
| H | 5.48244200 | -0.06431100 | 1.24739700 |
| O | 3.14000500 | 2.32049900 | -1.30400500 |
| H | 2.51611100 | 2.97204300 | -0.96360300 |
| F | -0.44479900 | -1.20903600 | -1.12520400 |
| E(RB3LYP): -1494.59787610 | | | |
| Sum of electronic and thermal free energies: -1494.225982 | | | |
| Number of imaginary frequencies: 0 | | | |

## 3F-LN **3** Conformer H

| **Atom** | **x** | **y** | **z** |
| --- | --- | --- | --- |
| H | -5.00965800 | 3.06346300 | -2.12840500 |
| O | -4.58798300 | 2.19799100 | -2.12940800 |
| C | -3.76233300 | 2.09497100 | -0.96677500 |
| H | -4.33345200 | 2.28889000 | -0.05433500 |
| H | -2.91935900 | 2.79283600 | -1.01422700 |
| C | -3.21045600 | 0.67743200 | -0.92482700 |
| H | -2.67431200 | 0.48222700 | -1.86508100 |
| O | -2.29235800 | 0.64621500 | 0.17196600 |
| C | -1.57367500 | -0.57704100 | 0.28498700 |
| H | -1.01979400 | -0.75937900 | -0.64761600 |
| O | -0.71955000 | -0.46929800 | 1.38384200 |
| C | 0.59048300 | 0.08527300 | 1.18697600 |
| H | 1.00201000 | 0.15757600 | 2.19408000 |
| C | 1.48557900 | -0.85776600 | 0.38801200 |
| H | 0.99327100 | -1.18904600 | -0.52961600 |
| C | 2.84943400 | -0.25010600 | 0.06126100 |
| H | 3.41942400 | -0.13777700 | 0.98426600 |
| N | 3.60632500 | -1.11860500 | -0.81635500 |
| H | 3.21177200 | -1.34228600 | -1.71837400 |
| C | 4.84490200 | -1.59676800 | -0.51806300 |
| O | 5.41158500 | -1.35997200 | 0.54791300 |
| C | 5.49587000 | -2.44671400 | -1.58850100 |
| H | 6.39259100 | -1.93094100 | -1.93978100 |
| H | 5.80900300 | -3.39131900 | -1.14048600 |
| H | 4.84666400 | -2.65006400 | -2.44095900 |
| C | 2.65793200 | 1.15502100 | -0.53049100 |
| H | 2.10417800 | 1.11110100 | -1.48536700 |
| O | 3.90478000 | 1.72788100 | -0.71148000 |
| C | 3.88733400 | 2.93674700 | -1.48323700 |
| H | 3.31092100 | 3.71526300 | -0.97766400 |
| H | 3.46377300 | 2.75268600 | -2.47699200 |
| H | 4.92413900 | 3.25371500 | -1.58322300 |
| O | 1.92302400 | 1.94785200 | 0.39526300 |
| C | 0.57516500 | 1.50885600 | 0.58541300 |
| H | 0.04719100 | 1.50780400 | -0.37643100 |
| C | -0.11752900 | 2.51743200 | 1.48535000 |
| H | -1.17387400 | 2.25220800 | 1.54675300 |
| C | -2.54945200 | -1.72722800 | 0.53089800 |
| H | -3.04611000 | -1.54346400 | 1.49064000 |
| C | -3.59608600 | -1.76087100 | -0.58360100 |
| H | -3.09548800 | -2.03655500 | -1.51885600 |
| C | -4.28355700 | -0.40159400 | -0.76464400 |
| H | -4.88965300 | -0.42292700 | -1.67858600 |
| O | -1.81207300 | -2.94340700 | 0.56336900 |
| H | -2.45422500 | -3.66436500 | 0.57444400 |
| O | 0.49709900 | 2.50330900 | 2.77875100 |
| H | -0.05243300 | 3.01769000 | 3.37855000 |
| H | -0.02281700 | 3.50582400 | 1.02101200 |
| O | -4.54122500 | -2.77500500 | -0.21451600 |
| H | -4.97403300 | -3.11059900 | -1.00801000 |
| O | -5.10149300 | -0.09158700 | 0.36090100 |
| H | -5.60171700 | -0.89010500 | 0.57431400 |
| F | 1.69795500 | -2.02443700 | 1.15389600 |
| E(RB3LYP): -1494.59690012 | | | |
| Sum of electronic and thermal free energies: -1494.224813 | | | |
| Number of imaginary frequencies: 0 | | | |

## 3F-LN **3** Conformer I

| **Atom** | **x** | **y** | **z** |
| --- | --- | --- | --- |
| H | -5.83231800 | 2.29950300 | 1.77838300 |
| O | -5.58117600 | 1.59380400 | 1.17341800 |
| C | -4.19669100 | 1.73965900 | 0.85635000 |
| H | -3.58159000 | 1.75520600 | 1.76155800 |
| H | -4.01442200 | 2.65571100 | 0.28386300 |
| C | -3.80513600 | 0.54401800 | -0.00129900 |
| H | -4.45673500 | 0.51792100 | -0.88486500 |
| O | -2.44971500 | 0.77585900 | -0.41733100 |
| C | -1.93253400 | -0.19377000 | -1.33119700 |
| H | -2.51658200 | -0.17720000 | -2.25874400 |
| O | -0.64687300 | 0.20230200 | -1.71578300 |
| C | 0.33868100 | 0.44750900 | -0.70011600 |
| H | -0.10018200 | 0.40128800 | 0.29886100 |
| C | 1.46602300 | -0.57140100 | -0.79904900 |
| H | 1.73738400 | -0.71107000 | -1.84942500 |
| C | 2.68321800 | -0.18828600 | 0.04660900 |
| H | 2.41338100 | -0.26164500 | 1.10098000 |
| N | 3.78222700 | -1.10303800 | -0.18410400 |
| H | 4.16194000 | -1.16241700 | -1.11777700 |
| C | 4.35967300 | -1.84861300 | 0.79665100 |
| O | 3.97106500 | -1.83013800 | 1.96386100 |
| C | 5.52015800 | -2.71882500 | 0.36340000 |
| H | 5.75925900 | -2.63386700 | -0.69724400 |
| H | 6.39775800 | -2.44206700 | 0.95120600 |
| H | 5.27946900 | -3.75858000 | 0.59420700 |
| C | 3.08660900 | 1.27021200 | -0.21979600 |
| H | 3.44647800 | 1.39544000 | -1.25634900 |
| O | 4.07059400 | 1.62107300 | 0.68839600 |
| C | 4.72545700 | 2.86253600 | 0.39332400 |
| H | 4.01993100 | 3.69601000 | 0.43272900 |
| H | 5.19457600 | 2.82436100 | -0.59621700 |
| H | 5.49282200 | 2.99478800 | 1.15432100 |
| O | 1.95820900 | 2.11533300 | -0.01932000 |
| C | 0.89842200 | 1.86325100 | -0.94196100 |
| H | 1.27917200 | 1.91524100 | -1.97304600 |
| C | -0.14957500 | 2.96034900 | -0.78353100 |
| H | -0.85665200 | 2.88537500 | -1.61532900 |
| C | -1.99822700 | -1.59654500 | -0.71544600 |
| H | -1.30281700 | -1.64756200 | 0.12678300 |
| C | -3.40795000 | -1.90087700 | -0.21009400 |
| H | -4.07750800 | -1.98807100 | -1.07327000 |
| C | -3.92636300 | -0.79745700 | 0.71783200 |
| H | -4.98682200 | -0.97336800 | 0.93524200 |
| O | -1.62282700 | -2.51383100 | -1.73633000 |
| H | -1.64429500 | -3.39715700 | -1.34931200 |
| O | -0.82990000 | 2.92031500 | 0.46570000 |
| H | -1.48691200 | 2.21117600 | 0.39285000 |
| H | 0.35996400 | 3.92359500 | -0.84763000 |
| O | -3.32047700 | -3.15598400 | 0.47795900 |
| H | -4.18865500 | -3.57507900 | 0.48593400 |
| O | -3.17367500 | -0.74947800 | 1.92718600 |
| H | -3.08707400 | -1.65851500 | 2.24260600 |
| F | 1.01582800 | -1.82581800 | -0.33596500 |
| E(RB3LYP): -1494.60383938 | | | |
| Sum of electronic and thermal free energies: -1494.229739 | | | |
| Number of imaginary frequencies: 0 | | | |

## 3F-LN **3** Conformer J

| **Atom** | **x** | **y** | **z** |
| --- | --- | --- | --- |
| H | 1.10950900 | 3.09011600 | 0.11561600 |
| O | 1.78767500 | 3.72697500 | 0.38305300 |
| C | 3.05083000 | 3.18132200 | -0.00486200 |
| H | 3.82465600 | 3.78365800 | 0.47415800 |
| H | 3.18345400 | 3.23519600 | -1.09015100 |
| C | 3.16437500 | 1.73346500 | 0.44444900 |
| H | 3.02321500 | 1.69283700 | 1.53445000 |
| O | 2.07264500 | 1.05890300 | -0.19573500 |
| C | 1.91634100 | -0.27822600 | 0.24040400 |
| H | 1.74464400 | -0.30584100 | 1.32769900 |
| O | 0.83790800 | -0.83966600 | -0.44282700 |
| C | -0.44828300 | -0.79932500 | 0.16732300 |
| H | -0.35521700 | -0.73666600 | 1.25751900 |
| C | -1.26151100 | 0.39414300 | -0.32340400 |
| H | -1.13567900 | 0.49924500 | -1.40427700 |
| C | -2.73885700 | 0.30205500 | 0.06078100 |
| H | -2.82997500 | 0.41895500 | 1.14149400 |
| N | -3.50095000 | 1.36637100 | -0.55819600 |
| H | -3.50482900 | 1.42148000 | -1.56627800 |
| C | -4.24049700 | 2.26962900 | 0.14133800 |
| O | -4.30219400 | 2.27087200 | 1.36988100 |
| C | -4.99201800 | 3.28712800 | -0.69032300 |
| H | -4.80840100 | 3.19883000 | -1.76174400 |
| H | -6.06080200 | 3.16628000 | -0.50053300 |
| H | -4.70589000 | 4.28679300 | -0.35802500 |
| C | -3.29651700 | -1.08557300 | -0.29597200 |
| H | -3.29855300 | -1.24276300 | -1.38843200 |
| O | -4.57433800 | -1.18648200 | 0.22099400 |
| C | -5.31437600 | -2.31979400 | -0.25551800 |
| H | -4.83451400 | -3.25411200 | 0.04552500 |
| H | -5.40632100 | -2.28744300 | -1.34678600 |
| H | -6.30320500 | -2.25279900 | 0.19498900 |
| O | -2.48257700 | -2.09188100 | 0.30767900 |
| C | -1.14921500 | -2.11963600 | -0.19834100 |
| H | -1.15181900 | -2.23439400 | -1.29094200 |
| C | -0.49704700 | -3.35858600 | 0.41680200 |
| H | -0.24314500 | -3.17232100 | 1.46604100 |
| H | -1.23316900 | -4.16459700 | 0.37359600 |
| O | 0.67861600 | -3.72175300 | -0.32529900 |
| H | 0.82124300 | -4.66870100 | -0.21786500 |
| C | 3.16238900 | -1.08752900 | -0.12120700 |
| H | 3.25299400 | -1.08129400 | -1.21427400 |
| C | 4.39805300 | -0.43173400 | 0.49159300 |
| H | 4.32178900 | -0.50836700 | 1.58443500 |
| O | 5.59787000 | -1.06472600 | 0.04377300 |
| H | 5.46613300 | -2.01961900 | 0.09720800 |
| C | 4.49396600 | 1.05263600 | 0.11455300 |
| H | 5.28518100 | 1.51553900 | 0.71887000 |
| O | 4.77837700 | 1.21703200 | -1.27319400 |
| H | 5.47611300 | 0.58636500 | -1.49422800 |
| O | 3.08712300 | -2.41280900 | 0.38105400 |
| H | 2.26728100 | -2.83247800 | 0.05198800 |
| F | -0.74807600 | 1.57888100 | 0.25295600 |
| E(RB3LYP): -1494.60795978 | | | |
| Sum of electronic and thermal free energies: -1494.233045 | | | |
| Number of imaginary frequencies: 0 | | | |

## 3F-LN **3** Conformer K

| **Atom** | **x** | **y** | **z** |
| --- | --- | --- | --- |
| H | 4.57333400 | 4.49182200 | -0.23107300 |
| O | 4.41731900 | 3.66223900 | 0.23170800 |
| C | 3.36957200 | 2.95813000 | -0.44075300 |
| H | 3.59757100 | 2.82583700 | -1.50228400 |
| H | 2.41327300 | 3.48317900 | -0.34421100 |
| C | 3.23872500 | 1.59423400 | 0.22156300 |
| H | 3.05505500 | 1.74414600 | 1.29558000 |
| O | 2.09818800 | 0.97084600 | -0.37970500 |
| C | 1.77903900 | -0.27931300 | 0.21170500 |
| H | 1.54801700 | -0.14537700 | 1.27908500 |
| O | 0.67964600 | -0.81466600 | -0.46698800 |
| C | -0.60449800 | -0.76244700 | 0.14717700 |
| H | -0.50985500 | -0.76637700 | 1.23898900 |
| C | -1.36382300 | 0.49344100 | -0.26848400 |
| H | -1.22702400 | 0.66135300 | -1.34043000 |
| C | -2.84650800 | 0.44771800 | 0.10030900 |
| H | -2.94374200 | 0.48628700 | 1.18600300 |
| N | -3.55058900 | 1.59157300 | -0.44058500 |
| H | -3.53771100 | 1.72733100 | -1.44090300 |
| C | -4.24835900 | 2.47642100 | 0.32235700 |
| O | -4.32210700 | 2.38683300 | 1.54707200 |
| C | -4.93665100 | 3.59355000 | -0.43249700 |
| H | -4.75551800 | 3.57183400 | -1.50777800 |
| H | -6.01112000 | 3.52278400 | -0.24998500 |
| H | -4.59291500 | 4.54812300 | -0.02947000 |
| C | -3.45795700 | -0.88233600 | -0.36493900 |
| H | -3.43574000 | -0.96662400 | -1.46501700 |
| O | -4.75282000 | -0.96188400 | 0.11186600 |
| C | -5.52615200 | -2.02576100 | -0.46196700 |
| H | -5.09142700 | -2.99874500 | -0.22073700 |
| H | -5.59051800 | -1.91032500 | -1.54956200 |
| H | -6.52203300 | -1.95063900 | -0.02861300 |
| O | -2.70395200 | -1.95985000 | 0.19093600 |
| C | -1.36220500 | -2.02988300 | -0.29012300 |
| H | -1.34635800 | -2.09729100 | -1.38631700 |
| C | -0.80221700 | -3.32868100 | 0.28995000 |
| H | -0.61603800 | -3.21101300 | 1.36304300 |
| H | -1.56485700 | -4.09810900 | 0.15098200 |
| O | 0.40736400 | -3.70214100 | -0.38882100 |
| H | 0.49431600 | -4.66089800 | -0.34640000 |
| C | 2.94583600 | -1.25594800 | 0.03935700 |
| H | 3.10017400 | -1.39715000 | -1.03698800 |
| C | 4.20788300 | -0.66511900 | 0.65602400 |
| H | 4.05640400 | -0.57503500 | 1.74023700 |
| O | 5.34820000 | -1.48637100 | 0.39421100 |
| H | 5.08800900 | -2.40362100 | 0.54730200 |
| C | 4.49324800 | 0.72477700 | 0.08325300 |
| H | 5.29858500 | 1.19238000 | 0.66140800 |
| O | 4.86194800 | 0.64548100 | -1.29329600 |
| H | 5.49394700 | -0.08066300 | -1.37232300 |
| O | 2.68877600 | -2.49239300 | 0.68607900 |
| H | 1.91829700 | -2.91583000 | 0.25655600 |
| F | -0.79267700 | 1.60490500 | 0.38610100 |
| E(RB3LYP): -1494.60352495 | | | |
| Sum of electronic and thermal free energies: -1494.230258 | | | |
| Number of imaginary frequencies: 0 | | | |

## 3F-LN **3** Conformer L

| **Atom** | **x** | **y** | **z** |
| --- | --- | --- | --- |
| H | 3.28745700 | -4.27352200 | 1.47174100 |
| O | 3.43028800 | -3.33863200 | 1.29124700 |
| C | 3.29110400 | -3.14362700 | -0.11602600 |
| H | 2.36645700 | -3.61011600 | -0.47410400 |
| H | 4.13287000 | -3.59069300 | -0.66047600 |
| C | 3.23066300 | -1.66960300 | -0.48492400 |
| H | 3.04097200 | -1.65884400 | -1.57078800 |
| O | 2.11583000 | -1.07288900 | 0.18790600 |
| C | 1.82210100 | 0.22767200 | -0.29612300 |
| H | 1.56730500 | 0.18579700 | -1.36616200 |
| O | 0.74981400 | 0.73939400 | 0.44287300 |
| C | -0.54720900 | 0.75777900 | -0.14558100 |
| H | -0.47378300 | 0.80895700 | -1.23784100 |
| C | -1.34150400 | -0.48907300 | 0.22946100 |
| H | -1.19916700 | -0.70317000 | 1.29267000 |
| C | -2.82572000 | -0.38009300 | -0.12074900 |
| H | -2.93401200 | -0.37504000 | -1.20613400 |
| N | -3.56209800 | -1.52010500 | 0.38443200 |
| H | -3.54177900 | -1.69552800 | 1.37864900 |
| C | -4.29526400 | -2.35225600 | -0.40412600 |
| O | -4.37672700 | -2.21404500 | -1.62386800 |
| C | -5.01167500 | -3.47554000 | 0.31439600 |
| H | -4.84680500 | -3.48107200 | 1.39243500 |
| H | -6.08177900 | -3.38484400 | 0.11670700 |
| H | -4.67527300 | -4.42596000 | -0.10478800 |
| C | -3.39206300 | 0.94995200 | 0.39909800 |
| H | -3.36872400 | 0.98834300 | 1.50160600 |
| O | -4.68345200 | 1.09109600 | -0.07320000 |
| C | -5.42302300 | 2.15349600 | 0.54587400 |
| H | -4.95762700 | 3.12164100 | 0.34590300 |
| H | -5.49158700 | 1.99407700 | 1.62763900 |
| H | -6.42057100 | 2.12845000 | 0.11055700 |
| O | -2.60335600 | 2.02488600 | -0.11213000 |
| C | -1.25767900 | 2.02574900 | 0.36350600 |
| H | -1.23449200 | 2.03484200 | 1.46159500 |
| C | -0.65481800 | 3.33398500 | -0.14823100 |
| H | -0.45835900 | 3.26367800 | -1.22358600 |
| H | -1.39671000 | 4.11764000 | 0.01987200 |
| O | 0.55709100 | 3.63857700 | 0.56070100 |
| H | 0.66588900 | 4.59588300 | 0.57688400 |
| C | 3.01926500 | 1.15669500 | -0.07495500 |
| H | 3.19714900 | 1.21187900 | 1.00562200 |
| C | 4.25388300 | 0.58169800 | -0.75945000 |
| H | 4.08128900 | 0.57387500 | -1.84383500 |
| O | 5.41819600 | 1.35559600 | -0.46272900 |
| H | 5.17304900 | 2.28745600 | -0.52729400 |
| C | 4.51614600 | -0.85344800 | -0.28642100 |
| H | 5.29544600 | -1.29479500 | -0.92248300 |
| O | 4.93502200 | -0.85944900 | 1.07426100 |
| H | 5.55881500 | -0.12672000 | 1.16791900 |
| O | 2.78844700 | 2.44535400 | -0.62212800 |
| H | 2.03870500 | 2.85828300 | -0.14789300 |
| F | -0.81605800 | -1.59213200 | -0.47482200 |
| E(RB3LYP): -1494.59861370 | | | |
| Sum of electronic and thermal free energies: -1494.225656 | | | |
| Number of imaginary frequencies: 0 | | | |

## 6F-LN **4** Conformer A

| **Atom** | **x** | **y** | **z** |
| --- | --- | --- | --- |
| H | 3.99471600 | 3.28267100 | 2.35426000 |
| O | 4.11415900 | 3.42637600 | 1.40860900 |
| C | 3.03355600 | 2.81541100 | 0.70802100 |
| H | 3.08349400 | 3.17197100 | -0.32035800 |
| H | 2.06545700 | 3.10147300 | 1.13217000 |
| C | 3.14387800 | 1.29125200 | 0.72148200 |
| H | 3.12685300 | 0.93854900 | 1.76239200 |
| O | 1.97875500 | 0.79862600 | 0.03771000 |
| C | 1.86895400 | -0.62796200 | 0.05101200 |
| H | 1.82738300 | -0.98074000 | 1.09270900 |
| O | 0.71565600 | -0.98053700 | -0.64017900 |
| C | -0.53875600 | -0.81065300 | 0.04567600 |
| H | -0.36694900 | -0.65237200 | 1.11438800 |
| C | -1.27874600 | 0.39964000 | -0.53445900 |
| H | -1.25408300 | 0.29539900 | -1.63012100 |
| O | -0.68099300 | 1.62792000 | -0.14845800 |
| H | 0.28625500 | 1.52491200 | -0.18399800 |
| C | -2.74122700 | 0.43547400 | -0.06778700 |
| H | -2.76932400 | 0.65757600 | 0.99961800 |
| N | -3.47061000 | 1.48652800 | -0.75052200 |
| H | -3.47166600 | 1.47689800 | -1.76006200 |
| C | -4.13228500 | 2.48832500 | -0.11563400 |
| O | -4.17017300 | 2.59474400 | 1.11057700 |
| C | -4.83258600 | 3.48736800 | -1.01346600 |
| H | -4.71571700 | 3.27930100 | -2.07773200 |
| H | -5.89593700 | 3.48963700 | -0.76491900 |
| H | -4.43877600 | 4.48279900 | -0.79815200 |
| C | -3.41054600 | -0.93033000 | -0.27538200 |
| H | -3.46873600 | -1.18061100 | -1.34916100 |
| O | -4.67246000 | -0.89917100 | 0.29111100 |
| C | -5.50204200 | -2.01461200 | -0.06114500 |
| H | -5.07339700 | -2.95163200 | 0.30270000 |
| H | -5.63360800 | -2.06811800 | -1.14777500 |
| H | -6.46695300 | -1.84382700 | 0.41354300 |
| O | -2.64811400 | -1.94275300 | 0.38638100 |
| C | -1.34056900 | -2.10407700 | -0.15303700 |
| H | -1.39209500 | -2.32719200 | -1.22978200 |
| C | -0.69918500 | -3.29964600 | 0.51446700 |
| H | -1.35874600 | -4.16621900 | 0.45739000 |
| H | 0.26581800 | -3.52146000 | 0.05958400 |
| C | 3.06847300 | -1.23020200 | -0.67996000 |
| H | 3.04665600 | -0.87317500 | -1.71420300 |
| C | 4.36060800 | -0.76708000 | -0.01086300 |
| H | 4.39054300 | -1.17173600 | 1.00959200 |
| O | 5.50453600 | -1.20436100 | -0.74189800 |
| H | 5.37987500 | -2.13750000 | -0.95690000 |
| C | 4.41936700 | 0.76181700 | 0.06287900 |
| H | 5.27230300 | 1.05482600 | 0.68586200 |
| O | 4.54105000 | 1.33063600 | -1.23860700 |
| H | 5.22704100 | 0.83300600 | -1.70191500 |
| O | 3.05578300 | -2.65416800 | -0.62917800 |
| H | 2.42123400 | -2.97804200 | -1.27807300 |
| F | -0.46736700 | -3.03210200 | 1.88258100 |
| E(RB3LYP): -1494.60971399 | | | |
| Sum of electronic and thermal free energies: -1494.237782 | | | |
| Number of imaginary frequencies: 0 | | | |

## 6F-LN **4** Conformer B

| **Atom** | **x** | **y** | **z** |
| --- | --- | --- | --- |
| H | 4.14358800 | 3.42248200 | 2.14004700 |
| O | 4.26881600 | 3.47519200 | 1.18571400 |
| C | 3.16563400 | 2.84592300 | 0.53859500 |
| H | 3.22141300 | 3.11758000 | -0.51503400 |
| H | 2.20971400 | 3.19663300 | 0.94120000 |
| C | 3.22742800 | 1.32473700 | 0.67326900 |
| H | 3.20864400 | 1.05633600 | 1.73894200 |
| O | 2.04084700 | 0.81600900 | 0.03839700 |
| C | 1.88757900 | -0.60035200 | 0.17204400 |
| H | 1.84354200 | -0.86162800 | 1.24018600 |
| O | 0.72109700 | -0.97813900 | -0.48277200 |
| C | -0.52963100 | -0.75042000 | 0.19219600 |
| H | -0.35661300 | -0.56233000 | 1.25778300 |
| C | -1.22703700 | 0.47005500 | -0.42566700 |
| H | -1.17723900 | 0.34182300 | -1.51786900 |
| O | -0.60325800 | 1.68663600 | -0.04701200 |
| H | 0.36114800 | 1.57195200 | -0.11608200 |
| C | -2.69950600 | 0.55977600 | -0.00030100 |
| H | -2.75119100 | 0.80237000 | 1.06175900 |
| N | -3.37592000 | 1.62018900 | -0.72167300 |
| H | -3.34762500 | 1.59458500 | -1.73059500 |
| C | -4.01739800 | 2.65595700 | -0.12142600 |
| O | -4.08429600 | 2.78415700 | 1.10145000 |
| C | -4.65559600 | 3.66491000 | -1.05362400 |
| H | -4.52915800 | 3.42807500 | -2.11073700 |
| H | -5.72186100 | 3.72107000 | -0.82505200 |
| H | -4.22043300 | 4.64608500 | -0.85252200 |
| C | -3.40199100 | -0.78961200 | -0.20122700 |
| H | -3.43141400 | -1.06484200 | -1.26976500 |
| O | -4.68047000 | -0.71003500 | 0.32198000 |
| C | -5.52869300 | -1.81108800 | -0.03149900 |
| H | -5.13692800 | -2.75051100 | 0.36609200 |
| H | -5.62820600 | -1.88588100 | -1.12027000 |
| H | -6.50277700 | -1.60418900 | 0.40884300 |
| O | -2.68833800 | -1.80205100 | 0.51189400 |
| C | -1.37322300 | -2.02282600 | 0.01405600 |
| H | -1.40405100 | -2.27805200 | -1.05462500 |
| C | -0.78357800 | -3.18699700 | 0.77765200 |
| H | 0.28072800 | -3.28956900 | 0.56702600 |
| H | -0.95108700 | -3.07011900 | 1.85011100 |
| C | 3.06191800 | -1.30120200 | -0.51065500 |
| H | 3.04158100 | -1.03636000 | -1.57232400 |
| C | 4.37407500 | -0.82364900 | 0.10682000 |
| H | 4.40178600 | -1.14120000 | 1.15773700 |
| O | 5.49694100 | -1.35586400 | -0.59302600 |
| H | 5.34330600 | -2.29979700 | -0.72660100 |
| C | 4.47927900 | 0.70346300 | 0.05077500 |
| H | 5.34716100 | 1.02125700 | 0.64001500 |
| O | 4.60320300 | 1.15763800 | -1.29476300 |
| H | 5.27255600 | 0.60594800 | -1.71955100 |
| O | 3.00272700 | -2.71404600 | -0.33377400 |
| H | 2.35096800 | -3.07315000 | -0.94622200 |
| F | -1.41442400 | -4.38421400 | 0.37871800 |
| E(RB3LYP): -1494.60900078 | | | |
| Sum of electronic and thermal free energies: -1494.235891 | | | |
| Number of imaginary frequencies: 0 | | | |

## 6F-LN **4** Conformer C

| **Atom** | **x** | **y** | **z** |
| --- | --- | --- | --- |
| H | 1.03280600 | 2.74797800 | 1.41285500 |
| O | 1.80602300 | 3.15080000 | 1.82829900 |
| C | 2.95185500 | 2.86168500 | 1.02575800 |
| H | 3.80715700 | 3.32089400 | 1.52374500 |
| H | 2.85481200 | 3.29635800 | 0.02619900 |
| C | 3.18271900 | 1.36383600 | 0.90557500 |
| H | 3.19886700 | 0.92937500 | 1.91393900 |
| O | 2.04581800 | 0.84103100 | 0.19313200 |
| C | 2.01895800 | -0.58108000 | 0.12060000 |
| H | 2.01411100 | -0.99979500 | 1.13876200 |
| O | 0.87357800 | -0.95427000 | -0.57908600 |
| C | -0.38212600 | -0.76660500 | 0.10026700 |
| H | -0.20934400 | -0.49632800 | 1.14734900 |
| C | -1.15210200 | 0.36710000 | -0.59073300 |
| H | -1.14424700 | 0.14553100 | -1.66870100 |
| O | -0.55525200 | 1.63539100 | -0.35758600 |
| H | 0.40408500 | 1.53236900 | -0.46810000 |
| C | -2.60524800 | 0.43854700 | -0.10320300 |
| H | -2.61909700 | 0.76305400 | 0.93805600 |
| N | -3.35784600 | 1.41110000 | -0.87115800 |
| H | -3.38096300 | 1.30275600 | -1.87463900 |
| C | -4.01121800 | 2.46864400 | -0.32330900 |
| O | -4.02098900 | 2.69586700 | 0.88673800 |
| C | -4.73684900 | 3.37095700 | -1.29967800 |
| H | -4.66428000 | 3.04323200 | -2.33744100 |
| H | -5.78911200 | 3.41691600 | -1.01196400 |
| H | -4.32418900 | 4.37835900 | -1.21328500 |
| C | -3.25989800 | -0.94951200 | -0.16647900 |
| H | -3.33694700 | -1.30059400 | -1.21008300 |
| O | -4.50916900 | -0.87915200 | 0.42358900 |
| C | -5.33209900 | -2.03152900 | 0.19534900 |
| H | -4.88215400 | -2.92652600 | 0.63188400 |
| H | -5.48910200 | -2.18524300 | -0.87820300 |
| H | -6.28737400 | -1.82880200 | 0.67683800 |
| O | -2.46906300 | -1.88433700 | 0.57156600 |
| C | -1.16954400 | -2.08319100 | 0.02422900 |
| H | -1.23775500 | -2.39876800 | -1.02656300 |
| C | -0.49728800 | -3.17964700 | 0.81827800 |
| H | 0.55451400 | -3.26478800 | 0.54486900 |
| H | -0.60161400 | -3.00308300 | 1.89062800 |
| C | 3.23889800 | -1.06366000 | -0.66160600 |
| H | 3.17028200 | -0.65592900 | -1.67525800 |
| C | 4.51442600 | -0.54943900 | 0.00618200 |
| H | 4.60307500 | -1.01985100 | 0.99453000 |
| O | 5.66363500 | -0.85057200 | -0.78193100 |
| H | 5.60479800 | -1.77508300 | -1.05426700 |
| C | 4.47670200 | 0.97394400 | 0.19106300 |
| H | 5.32574300 | 1.26705600 | 0.82173300 |
| O | 4.53805100 | 1.64746600 | -1.06309400 |
| H | 5.24166500 | 1.22913900 | -1.57625800 |
| O | 3.32495900 | -2.48533800 | -0.68452200 |
| H | 2.66079600 | -2.82404900 | -1.29577100 |
| F | -1.11171800 | -4.41636100 | 0.53073900 |
| E(RB3LYP): -1494.61089392 | | | |
| Sum of electronic and thermal free energies: -1494.237088 | | | |
| Number of imaginary frequencies: 0 | | | |

## 6F-LN **4** Conformer D

| **Atom** | **x** | **y** | **z** |
| --- | --- | --- | --- |
| H | 0.88468100 | 2.61711900 | 1.45521300 |
| O | 1.63314700 | 3.01924600 | 1.91470200 |
| C | 2.80780900 | 2.81210100 | 1.12867200 |
| H | 3.63561000 | 3.26632500 | 1.67535000 |
| H | 2.72271700 | 3.30167700 | 0.15372300 |
| C | 3.08778200 | 1.33126000 | 0.92751100 |
| H | 3.10326100 | 0.83914300 | 1.90926900 |
| O | 1.97878800 | 0.81718400 | 0.16811800 |
| C | 1.99582100 | -0.59889000 | 0.01177900 |
| H | 1.99164800 | -1.07847000 | 1.00261500 |
| O | 0.86696900 | -0.95882500 | -0.72086000 |
| C | -0.39318200 | -0.83076400 | -0.03577700 |
| H | -0.22636400 | -0.60068900 | 1.01988300 |
| C | -1.19939400 | 0.30199900 | -0.67849500 |
| H | -1.20794100 | 0.11975900 | -1.76381500 |
| O | -0.63287000 | 1.57877400 | -0.41188300 |
| H | 0.32969800 | 1.49853100 | -0.51129600 |
| C | -2.64278900 | 0.31408500 | -0.15817700 |
| H | -2.64141000 | 0.60692900 | 0.89249800 |
| N | -3.44099700 | 1.28594600 | -0.87963800 |
| H | -3.47604300 | 1.21085700 | -1.88583300 |
| C | -4.11625300 | 2.30448200 | -0.28636100 |
| O | -4.11322000 | 2.49066600 | 0.93069600 |
| C | -4.88413700 | 3.21660800 | -1.22048900 |
| H | -4.81212900 | 2.93076800 | -2.27059800 |
| H | -5.93406400 | 3.21467100 | -0.92066300 |
| H | -4.50583200 | 4.23392000 | -1.10011100 |
| C | -3.26094500 | -1.08988900 | -0.24955300 |
| H | -3.35763700 | -1.40746900 | -1.30243300 |
| O | -4.49571000 | -1.07158600 | 0.37396200 |
| C | -5.29679100 | -2.23508400 | 0.12596500 |
| H | -4.81610300 | -3.13311600 | 0.52165200 |
| H | -5.47562900 | -2.35692100 | -0.94826300 |
| H | -6.24483300 | -2.07140900 | 0.63579100 |
| O | -2.42922000 | -2.03235100 | 0.43304300 |
| C | -1.13631700 | -2.16719000 | -0.14956000 |
| H | -1.21836500 | -2.44371800 | -1.21195300 |
| C | -0.41551500 | -3.30040000 | 0.54429500 |
| H | -1.03974700 | -4.19433800 | 0.56290100 |
| H | 0.53501400 | -3.50677200 | 0.05229000 |
| C | 3.23891800 | -0.99388600 | -0.78268400 |
| H | 3.16958400 | -0.52482400 | -1.76939800 |
| C | 4.48926900 | -0.48345200 | -0.06539200 |
| H | 4.57913600 | -1.01047700 | 0.89377600 |
| O | 5.65775500 | -0.70186300 | -0.85264100 |
| H | 5.63022000 | -1.60938400 | -1.18127500 |
| C | 4.40344000 | 1.02387200 | 0.21197700 |
| H | 5.23418400 | 1.30210700 | 0.87306800 |
| O | 4.46336100 | 1.77484700 | -0.99761500 |
| H | 5.18564600 | 1.40815900 | -1.52416800 |
| O | 3.37244500 | -2.40780300 | -0.89525900 |
| H | 2.73202200 | -2.72656500 | -1.54134600 |
| F | -0.12979800 | -2.95365300 | 1.88447000 |
| E(RB3LYP): -1494.61188344 | | | |
| Sum of electronic and thermal free energies: -1494.238213 | | | |
| Number of imaginary frequencies: 0 | | | |

## 6F-LN **4** Conformer E

| **Atom** | **x** | **y** | **z** |
| --- | --- | --- | --- |
| H | 2.40065500 | 4.38725300 | 0.05053800 |
| O | 2.70897400 | 3.49567800 | -0.14205000 |
| C | 2.93719200 | 2.83668800 | 1.10240100 |
| H | 2.07839600 | 2.97766400 | 1.76818400 |
| H | 3.82818600 | 3.23115000 | 1.60682400 |
| C | 3.13116600 | 1.33925700 | 0.91322700 |
| H | 3.12763200 | 0.91152400 | 1.92706600 |
| O | 1.99941700 | 0.82964000 | 0.18971200 |
| C | 1.93369600 | -0.59673800 | 0.15356600 |
| H | 1.86745000 | -0.98813000 | 1.18040100 |
| O | 0.81442600 | -0.95801700 | -0.58855200 |
| C | -0.46727900 | -0.85083700 | 0.05834000 |
| H | -0.33810900 | -0.78543700 | 1.14288800 |
| C | -1.20416200 | 0.39861500 | -0.44193800 |
| H | -1.13739400 | 0.39067400 | -1.54083900 |
| O | -0.64748600 | 1.59760600 | 0.07291100 |
| H | 0.32400700 | 1.53138300 | 0.03111900 |
| C | -2.68391700 | 0.37137700 | -0.03071400 |
| H | -2.75507700 | 0.48547600 | 1.05156600 |
| N | -3.40291100 | 1.47750000 | -0.63241200 |
| H | -3.35236800 | 1.58084500 | -1.63529200 |
| C | -4.10738500 | 2.39939600 | 0.07315500 |
| O | -4.20636900 | 2.37295700 | 1.30041300 |
| C | -4.77705500 | 3.48292000 | -0.74665100 |
| H | -4.60001100 | 3.39406400 | -1.81923200 |
| H | -5.85200500 | 3.44561600 | -0.55784200 |
| H | -4.41260000 | 4.45310300 | -0.40283800 |
| C | -3.32242000 | -0.97500300 | -0.39746900 |
| H | -3.32637800 | -1.12321800 | -1.49166900 |
| O | -4.60944000 | -1.01240700 | 0.10940500 |
| C | -5.40379900 | -2.10029200 | -0.38227300 |
| H | -4.97646800 | -3.06194000 | -0.08764000 |
| H | -5.48634400 | -2.05395400 | -1.47403100 |
| H | -6.39152100 | -1.98653000 | 0.06163300 |
| O | -2.57489700 | -2.03533400 | 0.20300100 |
| C | -1.24159800 | -2.13124900 | -0.28586800 |
| H | -1.23984700 | -2.26161400 | -1.37908400 |
| C | -0.61328600 | -3.37250100 | 0.30656700 |
| H | -1.25425500 | -4.23908600 | 0.14090800 |
| H | 0.37643800 | -3.54075000 | -0.11668300 |
| C | 3.17451100 | -1.13960300 | -0.55451000 |
| H | 3.17255900 | -0.75591900 | -1.57942300 |
| C | 4.43040000 | -0.65329400 | 0.16592900 |
| H | 4.44172800 | -1.07829100 | 1.17834800 |
| O | 5.60888900 | -1.04238700 | -0.53648400 |
| H | 5.51160000 | -1.96978500 | -0.78766400 |
| C | 4.44238400 | 0.87921000 | 0.26625600 |
| H | 5.26477700 | 1.17571000 | 0.93080000 |
| O | 4.61589800 | 1.46092700 | -1.01966000 |
| H | 5.31532100 | 0.95636200 | -1.45642500 |
| O | 3.20902100 | -2.56423100 | -0.54163400 |
| H | 2.60350800 | -2.89133200 | -1.21608700 |
| F | -0.45418400 | -3.22338400 | 1.70275100 |
| E(RB3LYP): -1494.60496340 | | | |
| Sum of electronic and thermal free energies: -1494.231519 | | | |
| Number of imaginary frequencies: 0 | | | |

## 6F-LN **4** Conformer F

| **Atom** | **x** | **y** | **z** |
| --- | --- | --- | --- |
| H | -2.50821800 | -4.37973900 | -0.06177500 |
| O | -2.79239500 | -3.47450600 | -0.22582900 |
| C | -3.04470700 | -2.86221300 | 1.03755400 |
| H | -2.20740400 | -3.04513800 | 1.72029300 |
| H | -3.95591600 | -3.26075700 | 1.50094300 |
| C | -3.20802300 | -1.35512200 | 0.90470700 |
| H | -3.22069100 | -0.96915700 | 1.93504800 |
| O | -2.05024100 | -0.83726300 | 0.22939900 |
| C | -1.95749300 | 0.58666700 | 0.26132300 |
| H | -1.90621400 | 0.92777900 | 1.30693200 |
| O | -0.81765200 | 0.96278800 | -0.44160200 |
| C | 0.45384800 | 0.79094600 | 0.21096700 |
| H | 0.31178100 | 0.66016300 | 1.28974200 |
| C | 1.16122900 | -0.44894800 | -0.35843400 |
| H | 1.08095200 | -0.37870600 | -1.45414500 |
| O | 0.57939800 | -1.65829700 | 0.09895700 |
| H | -0.39031700 | -1.57427600 | 0.04931300 |
| C | 2.64682500 | -0.48392400 | 0.03008800 |
| H | 2.73257200 | -0.66400700 | 1.10228600 |
| N | 3.32715300 | -1.56949300 | -0.64897500 |
| H | 3.27817700 | -1.59626600 | -1.65692200 |
| C | 4.00530300 | -2.55788200 | -0.01090500 |
| O | 4.10301900 | -2.62032300 | 1.21513100 |
| C | 4.64507900 | -3.60062600 | -0.90422500 |
| H | 4.48586600 | -3.42493300 | -1.96882700 |
| H | 5.71793200 | -3.61858100 | -0.70164500 |
| H | 4.24031800 | -4.57957900 | -0.63909700 |
| C | 3.31356000 | 0.86491400 | -0.26896300 |
| H | 3.30321600 | 1.07742400 | -1.35254500 |
| O | 4.60886300 | 0.84172400 | 0.21729800 |
| C | 5.42077300 | 1.93935800 | -0.22158100 |
| H | 5.01922900 | 2.89083600 | 0.13555000 |
| H | 5.48697500 | 1.95583300 | -1.31529700 |
| H | 6.41171400 | 1.77807500 | 0.19983600 |
| O | 2.60000900 | 1.90158900 | 0.40790000 |
| C | 1.26652000 | 2.06717200 | -0.06091100 |
| H | 1.26072300 | 2.26343600 | -1.14250500 |
| C | 0.67408500 | 3.25884300 | 0.65627400 |
| H | -0.39648800 | 3.33193200 | 0.46682300 |
| H | 0.86909300 | 3.19975200 | 1.72883700 |
| C | -3.17085800 | 1.18790000 | -0.44728000 |
| H | -3.15103000 | 0.85361000 | -1.48922100 |
| C | -4.45331800 | 0.69266600 | 0.21786500 |
| H | -4.48408800 | 1.07118200 | 1.24799400 |
| O | -5.60587800 | 1.13482600 | -0.49601000 |
| H | -5.48703800 | 2.07105700 | -0.70084900 |
| C | -4.49445700 | -0.84261400 | 0.24735400 |
| H | -5.33831300 | -1.15355400 | 0.87750500 |
| O | -4.64557700 | -1.36214400 | -1.06770300 |
| H | -5.32483300 | -0.82543600 | -1.49791300 |
| O | -3.17669100 | 2.61059900 | -0.36577200 |
| H | -2.54819900 | 2.95765000 | -1.00884400 |
| F | 1.27301100 | 4.44478700 | 0.18157400 |
| E(RB3LYP): -1494.60419847 | | | |
| Sum of electronic and thermal free energies: -1494.231356 | | | |
| Number of imaginary frequencies: 0 | | | |

## 6F-LN **4** Conformer G

| **Atom** | **x** | **y** | **z** |
| --- | --- | --- | --- |
| H | -4.48517000 | 3.99653300 | -1.67421700 |
| O | -4.41715700 | 3.03627600 | -1.65926800 |
| C | -3.25649300 | 2.67562000 | -0.90840000 |
| H | -3.27461600 | 3.11723900 | 0.09202300 |
| H | -2.34016000 | 2.98948200 | -1.42061900 |
| C | -3.25046200 | 1.15834600 | -0.79060900 |
| H | -3.27073800 | 0.72555300 | -1.80011200 |
| O | -2.01528100 | 0.81268000 | -0.14507900 |
| C | -1.78337500 | -0.59788700 | -0.05023800 |
| H | -1.76376300 | -1.03171900 | -1.06131900 |
| O | -0.56874200 | -0.77879100 | 0.59387000 |
| C | 0.62064000 | -0.71785500 | -0.20900000 |
| H | 0.36664200 | -0.83231600 | -1.26745300 |
| C | 1.32619000 | 0.62382700 | 0.00067500 |
| H | 1.40077100 | 0.79754400 | 1.07987100 |
| O | 0.62233700 | 1.69162000 | -0.62440400 |
| H | -0.32088400 | 1.60242500 | -0.40192100 |
| C | 2.74130200 | 0.59387300 | -0.59867800 |
| H | 2.64723500 | 0.50522000 | -1.68315600 |
| N | 3.47144200 | 1.83122100 | -0.34676400 |
| H | 3.70154500 | 2.39484000 | -1.14906700 |
| C | 3.83249600 | 2.28591800 | 0.87825200 |
| O | 3.59136100 | 1.66415300 | 1.91566300 |
| C | 4.55957100 | 3.61447000 | 0.90692300 |
| H | 4.71511800 | 4.05265100 | -0.07952500 |
| H | 3.98267800 | 4.31022100 | 1.51989000 |
| H | 5.52764800 | 3.46906600 | 1.39070400 |
| C | 3.50760600 | -0.65199100 | -0.12511200 |
| H | 3.66171900 | -0.62881500 | 0.96206200 |
| O | 4.71589200 | -0.70913800 | -0.80053900 |
| C | 5.64129500 | -1.66234400 | -0.26193700 |
| H | 5.24948500 | -2.67921800 | -0.34528700 |
| H | 5.85755500 | -1.43754200 | 0.78852900 |
| H | 6.55478300 | -1.57273900 | -0.84792700 |
| O | 2.75214500 | -1.82159200 | -0.46196900 |
| C | 1.50647600 | -1.89213800 | 0.22420200 |
| H | 1.66203300 | -1.83735500 | 1.31264100 |
| C | 0.86776400 | -3.23282200 | -0.05845600 |
| H | -0.05751700 | -3.33670600 | 0.50893800 |
| C | -2.88521300 | -1.24699600 | 0.78100100 |
| H | -2.83026100 | -0.83409700 | 1.79461100 |
| C | -4.24944500 | -0.91871200 | 0.17514600 |
| H | -4.32535800 | -1.41190400 | -0.80060000 |
| C | -4.43840200 | 0.59395300 | -0.00908200 |
| H | -5.34728000 | 0.77370500 | -0.59613500 |
| O | -2.65193500 | -2.65105400 | 0.78967500 |
| H | -3.42086600 | -3.06544300 | 1.20110100 |
| H | 1.56158700 | -4.04339700 | 0.16751100 |
| O | -5.22699900 | -1.45515900 | 1.07609600 |
| H | -6.04290100 | -1.62940000 | 0.59304000 |
| O | -4.52735400 | 1.25193100 | 1.25163200 |
| H | -5.13091100 | 0.73314300 | 1.79934100 |
| F | 0.53196600 | -3.33605600 | -1.42990300 |
| E(RB3LYP): -1494.60654827 | | | |
| Sum of electronic and thermal free energies: -1494.233279 | | | |
| Number of imaginary frequencies: 0 | | | |

## 6F-LN **4** Conformer H

| **Atom** | **x** | **y** | **z** |
| --- | --- | --- | --- |
| H | -5.85447400 | 2.56464100 | -1.85658100 |
| O | -5.25693500 | 1.81103400 | -1.89913600 |
| C | -4.35902000 | 1.88032400 | -0.78890200 |
| H | -4.90121300 | 1.94010300 | 0.15907600 |
| H | -3.68940500 | 2.74311600 | -0.87194400 |
| C | -3.51636000 | 0.61335300 | -0.80973400 |
| H | -3.01340700 | 0.54307300 | -1.78501700 |
| O | -2.53802600 | 0.77245400 | 0.22576900 |
| C | -1.59527100 | -0.28587700 | 0.27141900 |
| H | -1.06210100 | -0.34202500 | -0.68758000 |
| O | -0.72794900 | -0.02377300 | 1.33499000 |
| C | 0.61070000 | 0.39842400 | 1.04706500 |
| H | 1.05456300 | 0.54522300 | 2.03261400 |
| C | 1.40628700 | -0.69659800 | 0.32180600 |
| H | 0.94730800 | -0.88662100 | -0.65895400 |
| O | 1.42667500 | -1.89538600 | 1.07586000 |
| H | 0.52714800 | -2.26666000 | 1.04186200 |
| C | 2.84625400 | -0.22486900 | 0.08391700 |
| H | 3.35558900 | -0.11515600 | 1.04221700 |
| N | 3.59117900 | -1.19273200 | -0.69634400 |
| H | 3.21432900 | -1.46695700 | -1.59195300 |
| C | 4.74589800 | -1.77431600 | -0.28048400 |
| O | 5.26974700 | -1.52246600 | 0.80517200 |
| C | 5.36571100 | -2.76735500 | -1.24164200 |
| H | 4.79265100 | -2.90649500 | -2.15919900 |
| H | 6.36965000 | -2.42148200 | -1.49671700 |
| H | 5.46450400 | -3.72791500 | -0.73197100 |
| C | 2.83590100 | 1.15070800 | -0.60546100 |
| H | 2.38937000 | 1.07366400 | -1.61252700 |
| O | 4.13569200 | 1.61953800 | -0.67781600 |
| C | 4.30533700 | 2.75414800 | -1.53828900 |
| H | 3.73860500 | 3.61301900 | -1.17064300 |
| H | 3.98727600 | 2.51376600 | -2.55903000 |
| H | 5.36883900 | 2.98761000 | -1.53496300 |
| O | 2.06859600 | 2.08473100 | 0.15909000 |
| C | 0.69075600 | 1.73222700 | 0.28742700 |
| H | 0.23267000 | 1.63720000 | -0.70902800 |
| C | -0.02837200 | 2.86341900 | 0.98599200 |
| H | -1.09886200 | 2.66334200 | 1.01507900 |
| C | -2.29409400 | -1.61848100 | 0.53565300 |
| H | -2.75342600 | -1.57031700 | 1.52818600 |
| C | -3.36861800 | -1.86515000 | -0.51702200 |
| H | -2.88175000 | -2.01181300 | -1.48793300 |
| C | -4.32839900 | -0.66984900 | -0.61021300 |
| H | -4.98049000 | -0.79894500 | -1.48247900 |
| O | -1.28830500 | -2.63387200 | 0.49429700 |
| H | -1.72197900 | -3.49177300 | 0.58323400 |
| H | 0.17783900 | 3.81011400 | 0.48457600 |
| O | -4.05733200 | -3.05887900 | -0.12382700 |
| H | -4.46531600 | -3.46099800 | -0.89944100 |
| O | -5.10896500 | -0.55354000 | 0.57662000 |
| H | -5.43622300 | -1.43760700 | 0.78800800 |
| F | 0.42380400 | 2.98662600 | 2.31979600 |
| E(RB3LYP): -1494.60503216 | | | |
| Sum of electronic and thermal free energies: -1494.231605 | | | |
| Number of imaginary frequencies: 0 | | | |

## 6F-LN **4** Conformer I

| **Atom** | **x** | **y** | **z** |
| --- | --- | --- | --- |
| H | -6.07518400 | 2.41722200 | 1.19315000 |
| O | -5.77394100 | 1.67080600 | 0.66508400 |
| C | -4.36091000 | 1.78682100 | 0.48013800 |
| H | -3.84054500 | 1.88564800 | 1.43671300 |
| H | -4.11132600 | 2.64603100 | -0.15145700 |
| C | -3.89201400 | 0.52030800 | -0.22140700 |
| H | -4.45911300 | 0.40457000 | -1.15630300 |
| O | -2.50819600 | 0.72851800 | -0.52531000 |
| C | -1.91605100 | -0.32437900 | -1.26528500 |
| H | -2.43797400 | -0.46449200 | -2.22066600 |
| O | -0.61981100 | 0.05242700 | -1.63050600 |
| C | 0.34859700 | 0.36088100 | -0.60912600 |
| H | -0.08108800 | 0.26117000 | 0.38945600 |
| C | 1.51966200 | -0.61672900 | -0.77920500 |
| H | 1.78480100 | -0.61292200 | -1.84756500 |
| O | 1.18090200 | -1.93027900 | -0.36683700 |
| H | 0.41470300 | -2.24352100 | -0.88006700 |
| C | 2.74438900 | -0.18124600 | 0.03519600 |
| H | 2.52122900 | -0.26862500 | 1.09928100 |
| N | 3.88085800 | -1.03779300 | -0.24264800 |
| H | 4.16477600 | -1.14462600 | -1.20551600 |
| C | 4.55920900 | -1.73081600 | 0.70713500 |
| O | 4.27002200 | -1.68838800 | 1.90371700 |
| C | 5.71176300 | -2.57694600 | 0.20655500 |
| H | 5.86794300 | -2.51219300 | -0.87098600 |
| H | 6.62226700 | -2.26025300 | 0.71932200 |
| H | 5.52197900 | -3.61750600 | 0.47819800 |
| C | 3.07361300 | 1.28914600 | -0.25329700 |
| H | 3.36129900 | 1.42554900 | -1.31094000 |
| O | 4.09627400 | 1.69166800 | 0.58932600 |
| C | 4.67423800 | 2.95859600 | 0.24867400 |
| H | 3.93709800 | 3.76106900 | 0.33166200 |
| H | 5.07912400 | 2.93599200 | -0.76948900 |
| H | 5.48337500 | 3.12949900 | 0.95705800 |
| O | 1.92888300 | 2.09633200 | 0.01722500 |
| C | 0.81129200 | 1.81298700 | -0.82167600 |
| H | 1.08408600 | 1.93485900 | -1.88139000 |
| C | -0.25032700 | 2.84647000 | -0.52038900 |
| H | -1.14016500 | 2.66880000 | -1.12156100 |
| C | -1.99336900 | -1.62252200 | -0.45248800 |
| H | -1.44513300 | -1.49426800 | 0.48598500 |
| C | -3.44954100 | -1.93399100 | -0.12703700 |
| H | -3.99173300 | -2.12433400 | -1.06024100 |
| C | -4.09750600 | -0.75147200 | 0.60611300 |
| H | -5.17567400 | -0.92867500 | 0.69731500 |
| O | -1.39700200 | -2.66343200 | -1.23109600 |
| H | -1.62963200 | -3.50408700 | -0.81330100 |
| H | 0.14455600 | 3.84977500 | -0.68769600 |
| O | -3.43736000 | -3.11353700 | 0.68691200 |
| H | -4.28889600 | -3.55995800 | 0.61418300 |
| O | -3.51395400 | -0.57517500 | 1.89443600 |
| H | -3.48594300 | -1.44398400 | 2.31582800 |
| F | -0.63284900 | 2.77218300 | 0.83672900 |
| E(RB3LYP): -1494.60576722 | | | |
| Sum of electronic and thermal free energies: -1494.231348 | | | |
| Number of imaginary frequencies: 0 | | | |

## 6F-LN **4** Conformer J

| **Atom** | **x** | **y** | **z** |
| --- | --- | --- | --- |
| H | 0.92975600 | 2.95658200 | 1.21705900 |
| O | 1.71338600 | 3.39457100 | 1.57172900 |
| C | 2.84018200 | 3.03763600 | 0.76919600 |
| H | 3.70196200 | 3.55124300 | 1.19799100 |
| H | 2.71330600 | 3.37320000 | -0.26465900 |
| C | 3.08888400 | 1.53826900 | 0.78739400 |
| H | 3.10038600 | 1.19918800 | 1.83185200 |
| O | 1.96877400 | 0.93411500 | 0.11300400 |
| C | 1.95767800 | -0.49076900 | 0.17560100 |
| H | 1.91868800 | -0.81260000 | 1.22701300 |
| O | 0.84538700 | -0.94563100 | -0.52900500 |
| C | -0.43363600 | -0.84010100 | 0.11912700 |
| H | -0.30099300 | -0.61403900 | 1.18272800 |
| C | -1.23405900 | 0.29048100 | -0.54794600 |
| H | -1.21581800 | 0.09640000 | -1.63113200 |
| O | -0.67745100 | 1.56984300 | -0.28222200 |
| H | 0.28507300 | 1.50021000 | -0.40083500 |
| C | -2.69027500 | 0.30885300 | -0.06584600 |
| H | -2.71681100 | 0.60573200 | 0.98338000 |
| N | -3.46830200 | 1.27869300 | -0.81160400 |
| H | -3.47979100 | 1.19884900 | -1.81800200 |
| C | -4.14898800 | 2.30592400 | -0.23965400 |
| O | -4.17061800 | 2.50005500 | 0.97594900 |
| C | -4.89011600 | 3.21711400 | -1.19590300 |
| H | -4.80963100 | 2.91534100 | -2.24091400 |
| H | -5.94343500 | 3.23678100 | -0.90920700 |
| H | -4.49631500 | 4.22969800 | -1.08505600 |
| C | -3.30631600 | -1.09324100 | -0.16834900 |
| H | -3.38119000 | -1.41606100 | -1.22085900 |
| O | -4.55023200 | -1.07918300 | 0.43413100 |
| C | -5.34373600 | -2.24566500 | 0.17349200 |
| H | -4.86635500 | -3.14176000 | 0.57734300 |
| H | -5.50472600 | -2.36808200 | -0.90337700 |
| H | -6.30019900 | -2.08473400 | 0.66809200 |
| O | -2.48079900 | -2.02891800 | 0.53364200 |
| C | -1.17647600 | -2.17380300 | -0.02862400 |
| H | -1.25404100 | -2.44498900 | -1.09053000 |
| C | -0.58711500 | -3.34618100 | 0.74630500 |
| H | -0.48586900 | -3.10094400 | 1.80514000 |
| H | -1.22254900 | -4.22323700 | 0.62235700 |
| C | 3.20322500 | -1.03690300 | -0.51946700 |
| H | 3.14284000 | -0.75149600 | -1.57606500 |
| C | 4.46090900 | -0.42893700 | 0.09984400 |
| H | 4.54614200 | -0.79170500 | 1.13245700 |
| O | 5.62661300 | -0.78831900 | -0.64089000 |
| H | 5.60529100 | -1.74084300 | -0.79515700 |
| C | 4.39647700 | 1.10310100 | 0.12631500 |
| H | 5.23317800 | 1.47350700 | 0.73266500 |
| O | 4.46006400 | 1.65024000 | -1.18791700 |
| H | 5.17483300 | 1.19471100 | -1.65139600 |
| O | 3.30854700 | -2.44796100 | -0.37451000 |
| H | 2.45898500 | -2.84495700 | -0.60877400 |
| F | 0.69735600 | -3.67689100 | 0.26654500 |
| E(RB3LYP): -1494.60860699 | | | |
| Sum of electronic and thermal free energies: -1494.233078 | | | |
| Number of imaginary frequencies: 0 | | | |

## 6F-LN **4** Conformer K

| **Atom** | **x** | **y** | **z** |
| --- | --- | --- | --- |
| H | 3.95249900 | 3.95596800 | 1.32787500 |
| O | 4.14208400 | 3.77419200 | 0.40042600 |
| C | 3.08075500 | 2.99358500 | -0.14381500 |
| H | 3.21592600 | 2.98812600 | -1.22485800 |
| H | 2.10336800 | 3.43136500 | 0.08449400 |
| C | 3.11350400 | 1.55684400 | 0.37725700 |
| H | 3.01227200 | 1.56832900 | 1.47189300 |
| O | 1.96935400 | 0.89834000 | -0.19751000 |
| C | 1.79557500 | -0.45045500 | 0.25453100 |
| H | 1.62752100 | -0.45368500 | 1.34140500 |
| O | 0.70718500 | -0.99691600 | -0.42020300 |
| C | -0.59611100 | -0.87217100 | 0.16455900 |
| H | -0.51541800 | -0.84177200 | 1.25741900 |
| C | -1.28601200 | 0.41269300 | -0.32528500 |
| H | -1.20941600 | 0.42198000 | -1.42300400 |
| O | -0.68367600 | 1.57678300 | 0.21773500 |
| H | 0.26141900 | 1.56776500 | -0.01820700 |
| C | -2.76742700 | 0.43907700 | 0.07588100 |
| H | -2.84189000 | 0.54125600 | 1.15921200 |
| N | -3.44275000 | 1.57750100 | -0.51502600 |
| H | -3.38841900 | 1.68936500 | -1.51681300 |
| C | -4.10645900 | 2.52149900 | 0.20139900 |
| O | -4.20261700 | 2.48722100 | 1.42854800 |
| C | -4.73166500 | 3.63999400 | -0.60626500 |
| H | -4.57354700 | 3.54587400 | -1.68134300 |
| H | -5.80437000 | 3.65663300 | -0.40287500 |
| H | -4.31370100 | 4.58939100 | -0.26508300 |
| C | -3.45168100 | -0.87749600 | -0.31305700 |
| H | -3.46525700 | -1.00663000 | -1.40895500 |
| O | -4.73390000 | -0.88597200 | 0.20279200 |
| C | -5.56800100 | -1.93482300 | -0.30915700 |
| H | -5.16927600 | -2.91679400 | -0.04319900 |
| H | -5.65834700 | -1.85705200 | -1.39834800 |
| H | -6.54727700 | -1.80048900 | 0.14730500 |
| O | -2.73114200 | -1.97235000 | 0.26315900 |
| C | -1.40389300 | -2.10939200 | -0.24586900 |
| H | -1.43680700 | -2.19890200 | -1.34017700 |
| C | -0.94085100 | -3.43343300 | 0.35213500 |
| H | -0.88688200 | -3.37111200 | 1.44033800 |
| H | -1.62236900 | -4.22894900 | 0.05219500 |
| C | 3.02369100 | -1.28698600 | -0.10118800 |
| H | 3.09973400 | -1.32117100 | -1.19385000 |
| C | 4.28183600 | -0.64749500 | 0.47571900 |
| H | 4.21689600 | -0.67727900 | 1.57144600 |
| O | 5.45769500 | -1.32907400 | 0.03857900 |
| H | 5.31329400 | -2.27679700 | 0.15199000 |
| C | 4.40354300 | 0.81134000 | 0.03110800 |
| H | 5.22568500 | 1.28330600 | 0.58150400 |
| O | 4.63367800 | 0.90396900 | -1.37306200 |
| H | 5.32855600 | 0.26885200 | -1.58861200 |
| O | 2.92387300 | -2.59417400 | 0.44854500 |
| H | 2.10557400 | -3.00140900 | 0.12890500 |
| F | 0.34759800 | -3.78572400 | -0.11169800 |
| E(RB3LYP): -1494.60806663 | | | |
| Sum of electronic and thermal free energies: -1494.233665 | | | |
| Number of imaginary frequencies: 0 | | | |

## 6F-LN **4** Conformer L

| **Atom** | **x** | **y** | **z** |
| --- | --- | --- | --- |
| H | 2.63831200 | 4.41111100 | -0.95883400 |
| O | 2.88858200 | 3.48238200 | -0.91351300 |
| C | 2.96408000 | 3.11905800 | 0.46284500 |
| H | 2.05808000 | 3.43783100 | 0.99122700 |
| H | 3.82557900 | 3.59036700 | 0.95259400 |
| C | 3.09756700 | 1.61541500 | 0.64465800 |
| H | 3.03050300 | 1.45056300 | 1.73083400 |
| O | 1.97716800 | 0.97529800 | 0.00780200 |
| C | 1.85731100 | -0.41249500 | 0.33606300 |
| H | 1.68605400 | -0.52034900 | 1.41747200 |
| O | 0.79418800 | -0.94118200 | -0.39153100 |
| C | -0.52063100 | -0.88572400 | 0.17771200 |
| H | -0.45717500 | -0.90436200 | 1.27203300 |
| C | -1.24330700 | 0.39904600 | -0.26414000 |
| H | -1.14291700 | 0.46375300 | -1.35821000 |
| O | -0.69425400 | 1.55526000 | 0.34715500 |
| H | 0.26065900 | 1.57958900 | 0.15204600 |
| C | -2.73376400 | 0.35937800 | 0.10233800 |
| H | -2.83528400 | 0.41688400 | 1.18678300 |
| N | -3.43233300 | 1.49819100 | -0.46061400 |
| H | -3.35560700 | 1.65216800 | -1.45531300 |
| C | -4.14011800 | 2.39444700 | 0.27439800 |
| O | -4.26432900 | 2.31011100 | 1.49658500 |
| C | -4.78022500 | 3.52354900 | -0.50648200 |
| H | -4.58250900 | 3.48187600 | -1.57835700 |
| H | -5.85913400 | 3.49146800 | -0.34087600 |
| H | -4.41101400 | 4.47196400 | -0.11094700 |
| C | -3.36794200 | -0.96109300 | -0.35204800 |
| H | -3.36113200 | -1.04484700 | -1.45228700 |
| O | -4.65723100 | -1.03243300 | 0.14158200 |
| C | -5.44856800 | -2.08426000 | -0.42891700 |
| H | -5.02159500 | -3.06360900 | -0.19960200 |
| H | -5.52539800 | -1.96154100 | -1.51500200 |
| H | -6.43839800 | -2.00202400 | 0.01702200 |
| O | -2.62065800 | -2.05534700 | 0.19009500 |
| C | -1.28250300 | -2.12643500 | -0.30384600 |
| H | -1.29771200 | -2.16413100 | -1.40159300 |
| C | -0.78586900 | -3.46324100 | 0.23586200 |
| H | -0.74807900 | -3.45315100 | 1.32646500 |
| H | -1.43807000 | -4.26360300 | -0.11249300 |
| C | 3.11717600 | -1.16792600 | -0.08494000 |
| H | 3.18803100 | -1.11370300 | -1.17714300 |
| C | 4.35523400 | -0.52757700 | 0.53383700 |
| H | 4.30668200 | -0.65087800 | 1.62355100 |
| O | 5.55102500 | -1.12395400 | 0.03113500 |
| H | 5.43953500 | -2.08258900 | 0.05282900 |
| C | 4.41511900 | 0.96898900 | 0.20173900 |
| H | 5.22436300 | 1.42313800 | 0.78898600 |
| O | 4.64579900 | 1.16266600 | -1.18886400 |
| H | 5.33965900 | 0.53835700 | -1.44026500 |
| O | 3.07304600 | -2.51764700 | 0.36022100 |
| H | 2.26353000 | -2.92715800 | 0.02185300 |
| F | 0.51903600 | -3.75249700 | -0.22536600 |
| E(RB3LYP): -1494.60304885 | | | |
| Sum of electronic and thermal free energies: -1494.229225 | | | |
| Number of imaginary frequencies: 0 | | | |

## 2′F-LN **5** Conformer A

| **Atom** | **x** | **y** | **z** |
| --- | --- | --- | --- |
| H | 4.24507600 | 4.35987500 | 1.03787400 |
| O | 4.21371400 | 3.40976700 | 1.19060100 |
| C | 3.13239900 | 2.86440500 | 0.43385700 |
| H | 3.22009600 | 3.11455500 | -0.62707900 |
| H | 2.16663000 | 3.22240500 | 0.80703300 |
| C | 3.18696700 | 1.35306100 | 0.60283300 |
| H | 3.12861400 | 1.11359000 | 1.67273400 |
| O | 2.02724300 | 0.83291900 | -0.07031100 |
| C | 1.84819300 | -0.57878400 | 0.08751700 |
| H | 1.74339300 | -0.81869600 | 1.15579400 |
| O | 0.72264100 | -0.95323000 | -0.62572500 |
| C | -0.55494700 | -0.81329800 | 0.02363300 |
| H | -0.41860700 | -0.73938100 | 1.10683800 |
| C | -1.26673800 | 0.44305300 | -0.48640700 |
| H | -1.22366200 | 0.41826600 | -1.58590500 |
| O | -0.66418300 | 1.63452000 | -0.00137400 |
| H | 0.29884800 | 1.55900700 | -0.11715100 |
| C | -2.73583600 | 0.45764900 | -0.04166200 |
| H | -2.78011600 | 0.58771500 | 1.04032300 |
| N | -3.44493600 | 1.57046100 | -0.64294600 |
| H | -3.42187500 | 1.65357600 | -1.64870500 |
| C | -4.11211300 | 2.51860500 | 0.06403100 |
| O | -4.17824400 | 2.51631100 | 1.29378400 |
| C | -4.78162100 | 3.60081300 | -0.75775000 |
| H | -4.63958200 | 3.48611200 | -1.83308900 |
| H | -5.85083200 | 3.59270100 | -0.53592200 |
| H | -4.38462900 | 4.56900300 | -0.44573600 |
| C | -3.41299000 | -0.87918900 | -0.37793500 |
| H | -3.45111900 | -1.03402300 | -1.47049800 |
| O | -4.68687000 | -0.88125500 | 0.16405300 |
| C | -5.51919400 | -1.95435700 | -0.29582500 |
| H | -5.10811700 | -2.92301200 | -0.00104900 |
| H | -5.62745700 | -1.91776800 | -1.38574700 |
| H | -6.49272500 | -1.81179400 | 0.17060700 |
| O | -2.67610500 | -1.95402600 | 0.20684000 |
| C | -1.34666000 | -2.08504200 | -0.29993500 |
| H | -1.36331500 | -2.22926800 | -1.38987000 |
| C | -0.75133200 | -3.32905200 | 0.34597500 |
| H | -1.34449000 | -4.19884300 | 0.04203600 |
| H | 0.27274200 | -3.46106200 | -0.00354000 |
| O | -0.69166800 | -3.22500300 | 1.76894400 |
| H | -1.59001700 | -3.07728000 | 2.08929000 |
| C | 3.05956900 | -1.29025500 | -0.50457400 |
| H | 3.07720100 | -1.15094600 | -1.58600900 |
| C | 4.35251200 | -0.80809900 | 0.13963600 |
| H | 4.34056300 | -1.09197400 | 1.19968700 |
| O | 5.49642400 | -1.35797000 | -0.50644000 |
| H | 5.43617600 | -2.32040400 | -0.48646900 |
| C | 4.45956100 | 0.71789200 | 0.03886200 |
| H | 5.30892700 | 1.05177100 | 0.64468600 |
| O | 4.62017900 | 1.12927500 | -1.31565000 |
| H | 5.34496800 | 0.61349400 | -1.69069500 |
| F | 2.93513000 | -2.67508000 | -0.25890400 |
| E(RB3LYP): -1494.60737132 | | | |
| Sum of electronic and thermal free energies: -1494.235461 | | | |
| Number of imaginary frequencies: 0 | | | |

## 2′F-LN **5** Conformer B

| **Atom** | **x** | **y** | **z** |
| --- | --- | --- | --- |
| H | 4.39252600 | 4.38124600 | 0.62967800 |
| O | 4.33116000 | 3.45646700 | 0.89041600 |
| C | 3.23368400 | 2.86159900 | 0.19723700 |
| H | 3.32271100 | 2.99588700 | -0.88436100 |
| H | 2.27868400 | 3.28142000 | 0.53198400 |
| C | 3.25173900 | 1.37552800 | 0.52488500 |
| H | 3.19216300 | 1.25126800 | 1.61405300 |
| O | 2.07492900 | 0.81785000 | -0.08748200 |
| C | 1.86111200 | -0.56551600 | 0.21460100 |
| H | 1.74962000 | -0.69118900 | 1.30158200 |
| O | 0.72696200 | -0.98637200 | -0.45936400 |
| C | -0.55024400 | -0.77574100 | 0.17017900 |
| H | -0.42229200 | -0.67649200 | 1.25413000 |
| C | -1.21117100 | 0.49653500 | -0.37715300 |
| H | -1.14834200 | 0.44430800 | -1.47463500 |
| O | -0.57639800 | 1.67519600 | 0.09455700 |
| H | 0.38069800 | 1.58667400 | -0.05816000 |
| C | -2.68823300 | 0.57725500 | 0.03724100 |
| H | -2.74808900 | 0.74020800 | 1.11402300 |
| N | -3.34278600 | 1.69846700 | -0.60802200 |
| H | -3.32157500 | 1.73767700 | -1.61647000 |
| C | -3.95389400 | 2.70899000 | 0.06228200 |
| O | -4.01160300 | 2.75959700 | 1.29141900 |
| C | -4.57042900 | 3.79210400 | -0.79866300 |
| H | -4.45175100 | 3.62313100 | -1.86963500 |
| H | -5.63464200 | 3.85789600 | -0.56311300 |
| H | -4.11187200 | 4.74736100 | -0.53464300 |
| C | -3.41084700 | -0.74166300 | -0.27266000 |
| H | -3.43587700 | -0.92966000 | -1.36035100 |
| O | -4.69210000 | -0.68021100 | 0.24737600 |
| C | -5.55551700 | -1.73742300 | -0.19196000 |
| H | -5.18815300 | -2.70970800 | 0.14558400 |
| H | -5.64058000 | -1.73634200 | -1.28449100 |
| H | -6.53190800 | -1.54191800 | 0.24851800 |
| O | -2.71885800 | -1.81909900 | 0.36043200 |
| C | -1.39515800 | -2.01455900 | -0.14064300 |
| H | -1.42564100 | -2.16605700 | -1.22897700 |
| C | -0.86092100 | -3.27443600 | 0.52238700 |
| H | 0.16347000 | -3.45122300 | 0.19582700 |
| H | -0.86370200 | -3.13392600 | 1.61102400 |
| O | -1.62422500 | -4.42241000 | 0.15612100 |
| H | -2.54318600 | -4.24545300 | 0.39211900 |
| C | 3.05424000 | -1.36452700 | -0.29844400 |
| H | 3.07351600 | -1.33890900 | -1.38848900 |
| C | 4.36030600 | -0.85060400 | 0.29242900 |
| H | 4.34401200 | -1.01827000 | 1.37703100 |
| O | 5.48820300 | -1.49565700 | -0.29047300 |
| H | 5.40344000 | -2.44866600 | -0.16804900 |
| C | 4.50532000 | 0.65248100 | 0.02878400 |
| H | 5.36519700 | 1.02751500 | 0.59430700 |
| O | 4.67004100 | 0.91333600 | -1.36196300 |
| H | 5.38297100 | 0.34580200 | -1.68073100 |
| F | 2.89481200 | -2.71239800 | 0.08967500 |
| E(RB3LYP): -1494.60677691 | | | |
| Sum of electronic and thermal free energies: -1494.234420 | | | |
| Number of imaginary frequencies: 0 | | | |

## 2′F-LN **5** Conformer C

| **Atom** | **x** | **y** | **z** |
| --- | --- | --- | --- |
| H | -1.03440700 | -2.72607400 | 1.39280600 |
| O | -1.80505300 | -3.12254500 | 1.81938600 |
| C | -2.95791400 | -2.84066700 | 1.02531000 |
| H | -3.80807900 | -3.29942800 | 1.53234600 |
| H | -2.86870700 | -3.27897000 | 0.02667000 |
| C | -3.19320300 | -1.34353500 | 0.90150000 |
| H | -3.20012900 | -0.90468700 | 1.90775900 |
| O | -2.06564600 | -0.82310700 | 0.17234200 |
| C | -2.01500200 | 0.59882500 | 0.10759500 |
| H | -1.99280200 | 1.01409800 | 1.12658200 |
| O | -0.88044500 | 0.95442700 | -0.60863900 |
| C | 0.38112600 | 0.77018300 | 0.06531900 |
| H | 0.21368600 | 0.52187700 | 1.11873200 |
| C | 1.14267400 | -0.37841200 | -0.60808200 |
| H | 1.14341900 | -0.17264600 | -1.68909200 |
| O | 0.53796700 | -1.64159600 | -0.36191300 |
| H | -0.41765700 | -1.53686300 | -0.49519300 |
| C | 2.59261100 | -0.45123000 | -0.10910800 |
| H | 2.59610700 | -0.76347400 | 0.93602800 |
| N | 3.34537000 | -1.43739200 | -0.85952700 |
| H | 3.37545800 | -1.34112900 | -1.86404100 |
| C | 3.98556100 | -2.49420000 | -0.29513300 |
| O | 3.98413700 | -2.70826700 | 0.91739600 |
| C | 4.71176200 | -3.41294700 | -1.25562900 |
| H | 4.64557700 | -3.09926700 | -2.29813400 |
| H | 5.76251700 | -3.45943200 | -0.96240700 |
| H | 4.29439800 | -4.41727700 | -1.15725500 |
| C | 3.25981300 | 0.93136200 | -0.18335100 |
| H | 3.35452900 | 1.26579200 | -1.23089600 |
| O | 4.50064000 | 0.85774600 | 0.42491200 |
| C | 5.33761700 | 1.99873100 | 0.19187800 |
| H | 4.89523000 | 2.90300500 | 0.61704300 |
| H | 5.50391600 | 2.14113500 | -0.88182700 |
| H | 6.28724700 | 1.79031300 | 0.68206000 |
| O | 2.46602100 | 1.88345800 | 0.52759800 |
| C | 1.16871700 | 2.07857600 | -0.04002300 |
| H | 1.26053100 | 2.36343200 | -1.09775000 |
| C | 0.52603400 | 3.22286400 | 0.72732200 |
| H | -0.48172000 | 3.39409900 | 0.34863000 |
| H | 0.46278500 | 2.95274700 | 1.78924700 |
| O | 1.24869300 | 4.43986400 | 0.55419500 |
| H | 2.15848000 | 4.27765500 | 0.83279500 |
| C | -3.24970700 | 1.07879500 | -0.64562700 |
| H | -3.18802700 | 0.76477500 | -1.68835900 |
| C | -4.52866900 | 0.56942300 | 0.01367300 |
| H | -4.61437000 | 1.03415400 | 1.00409000 |
| O | -5.67503700 | 0.87005700 | -0.77474100 |
| H | -5.72028600 | 1.82315300 | -0.91597900 |
| C | -4.49000000 | -0.95629000 | 0.19225500 |
| H | -5.33948000 | -1.24858100 | 0.82191500 |
| O | -4.54708800 | -1.62456500 | -1.06372500 |
| H | -5.28899200 | -1.25268800 | -1.55753600 |
| F | -3.28431700 | 2.48926500 | -0.62927000 |
| E(RB3LYP): -1494.60894994 | | | |
| Sum of electronic and thermal free energies: -1494.235751 | | | |
| Number of imaginary frequencies: 0 | | | |

## 2′F-LN **5** Conformer D

| **Atom** | **x** | **y** | **z** |
| --- | --- | --- | --- |
| H | 0.88204600 | 2.59756300 | 1.43666800 |
| O | 1.62468300 | 2.99188100 | 1.91247100 |
| C | 2.80986500 | 2.79607000 | 1.14038300 |
| H | 3.62966300 | 3.24582400 | 1.70253200 |
| H | 2.73712000 | 3.29487500 | 0.16922700 |
| C | 3.09463800 | 1.31734700 | 0.92863500 |
| H | 3.09934700 | 0.81534900 | 1.90524200 |
| O | 1.99641700 | 0.81180900 | 0.14825300 |
| C | 1.99013000 | -0.60409500 | -0.00907000 |
| H | 1.96313800 | -1.08716900 | 0.97937300 |
| O | 0.87404400 | -0.93962000 | -0.76308500 |
| C | -0.39089700 | -0.82640600 | -0.07998600 |
| H | -0.22606800 | -0.61268300 | 0.97941100 |
| C | -1.19643200 | 0.31414100 | -0.70573400 |
| H | -1.21592800 | 0.14549300 | -1.79304300 |
| O | -0.62552500 | 1.58839700 | -0.42981100 |
| H | 0.33399600 | 1.51004100 | -0.55091300 |
| C | -2.63384800 | 0.32136600 | -0.16996100 |
| H | -2.62029800 | 0.59985500 | 0.88457400 |
| N | -3.43894100 | 1.30368100 | -0.86958800 |
| H | -3.48137800 | 1.24390500 | -1.87652200 |
| C | -4.11020100 | 2.31247800 | -0.25590800 |
| O | -4.09847700 | 2.48008700 | 0.96388300 |
| C | -4.88594900 | 3.23814200 | -1.17008900 |
| H | -4.81271200 | 2.97526100 | -2.22609400 |
| H | -5.93565800 | 3.22153700 | -0.86972800 |
| H | -4.51566700 | 4.25559100 | -1.02825900 |
| C | -3.25594200 | -1.08038200 | -0.27457200 |
| H | -3.36968700 | -1.37877400 | -1.33118700 |
| O | -4.48238100 | -1.06832000 | 0.36712700 |
| C | -5.29118700 | -2.22402900 | 0.10920100 |
| H | -4.80810100 | -3.13119700 | 0.48047000 |
| H | -5.48643500 | -2.32499300 | -0.96437900 |
| H | -6.23111000 | -2.06707300 | 0.63597300 |
| O | -2.41874000 | -2.03758500 | 0.37720800 |
| C | -1.12275200 | -2.16461900 | -0.21314100 |
| H | -1.20877500 | -2.42881700 | -1.27699600 |
| C | -0.41379400 | -3.29990800 | 0.51182300 |
| H | -0.99242700 | -4.21969700 | 0.37182600 |
| H | 0.57633800 | -3.44134000 | 0.07763800 |
| O | -0.21940700 | -3.02342900 | 1.89892200 |
| H | -1.08783400 | -2.88151100 | 2.29522700 |
| C | 3.24926500 | -0.99542500 | -0.77219100 |
| H | 3.19302900 | -0.61445400 | -1.79271200 |
| C | 4.50236500 | -0.49256800 | -0.05982300 |
| H | 4.58934400 | -1.01941200 | 0.89878000 |
| O | 5.66894800 | -0.70406100 | -0.84804300 |
| H | 5.74265700 | -1.64236800 | -1.05878700 |
| C | 4.41422500 | 1.01609700 | 0.21919500 |
| H | 5.24432600 | 1.29061300 | 0.88191200 |
| O | 4.47195300 | 1.76764100 | -0.98897400 |
| H | 5.22925600 | 1.44625500 | -1.49478200 |
| F | 3.32837400 | -2.40245600 | -0.84938900 |
| E(RB3LYP): -1494.60980005 | | | |
| Sum of electronic and thermal free energies: -1494.236813 | | | |
| Number of imaginary frequencies: 0 | | | |

## 2′F-LN **5** Conformer E

| **Atom** | **x** | **y** | **z** |
| --- | --- | --- | --- |
| H | 2.51194800 | 4.39725100 | -0.05199200 |
| O | 2.80466200 | 3.49611600 | -0.22360100 |
| C | 2.99491000 | 2.85350600 | 1.03489300 |
| H | 2.12717900 | 3.02243100 | 1.68232500 |
| H | 3.88435900 | 3.23700500 | 1.55044400 |
| C | 3.16100900 | 1.34952500 | 0.87515800 |
| H | 3.13469300 | 0.94130000 | 1.89637200 |
| O | 2.03153100 | 0.84728100 | 0.14183700 |
| C | 1.91769700 | -0.57667800 | 0.15122400 |
| H | 1.81249400 | -0.93232600 | 1.18726100 |
| O | 0.81613400 | -0.92466600 | -0.61291600 |
| C | -0.47527900 | -0.83937600 | 0.01928400 |
| H | -0.35875900 | -0.80943300 | 1.10695200 |
| C | -1.20623600 | 0.42308900 | -0.44922000 |
| H | -1.14444100 | 0.44539200 | -1.54807100 |
| O | -0.64394000 | 1.60811300 | 0.09506100 |
| H | 0.32557100 | 1.55123700 | 0.02390800 |
| C | -2.68314200 | 0.38528200 | -0.03065000 |
| H | -2.74784100 | 0.46845000 | 1.05491700 |
| N | -3.40624800 | 1.50796100 | -0.59607100 |
| H | -3.36363400 | 1.63888100 | -1.59613200 |
| C | -4.09877300 | 2.41444300 | 0.14031100 |
| O | -4.18529100 | 2.35671000 | 1.36750800 |
| C | -4.77063100 | 3.52226400 | -0.64451100 |
| H | -4.61075900 | 3.45539200 | -1.72141100 |
| H | -5.84280900 | 3.49114500 | -0.43978400 |
| H | -4.39119200 | 4.48111200 | -0.28531100 |
| C | -3.32588600 | -0.94979000 | -0.43259300 |
| H | -3.34394100 | -1.06084900 | -1.53068000 |
| O | -4.60762900 | -1.00127800 | 0.08859200 |
| C | -5.41002000 | -2.07047100 | -0.42980700 |
| H | -4.98152600 | -3.04234700 | -0.17275400 |
| H | -5.50524600 | -1.98753400 | -1.51839400 |
| H | -6.39235900 | -1.96996100 | 0.02907800 |
| O | -2.57435400 | -2.03172900 | 0.11976800 |
| C | -1.23498200 | -2.11203100 | -0.37131400 |
| H | -1.23271500 | -2.20945300 | -1.46653200 |
| C | -0.62245400 | -3.37042900 | 0.22888200 |
| H | -1.19436800 | -4.23787100 | -0.11928800 |
| H | 0.40792300 | -3.46793300 | -0.11337900 |
| O | -0.58128900 | -3.32628600 | 1.65557300 |
| H | -1.48618400 | -3.21107700 | 1.97078200 |
| C | 3.16672900 | -1.16702200 | -0.49309400 |
| H | 3.18421200 | -0.92146900 | -1.55557000 |
| C | 4.43135500 | -0.68701800 | 0.20849700 |
| H | 4.42924000 | -1.07217700 | 1.23592400 |
| O | 5.60370100 | -1.11707900 | -0.47627700 |
| H | 5.58163600 | -2.07706800 | -0.56770900 |
| C | 4.46805900 | 0.84933200 | 0.25086600 |
| H | 5.29517600 | 1.15333800 | 0.90533200 |
| O | 4.64739900 | 1.37946600 | -1.05547800 |
| H | 5.37071600 | 0.88448400 | -1.46222500 |
| F | 3.11253700 | -2.57384200 | -0.38551800 |
| E(RB3LYP): -1494.60305296 | | | |
| Sum of electronic and thermal free energies: -1494.230211 | | | |
| Number of imaginary frequencies: 0 | | | |

## 2′F-LN **5** Conformer F

| **Atom** | **x** | **y** | **z** |
| --- | --- | --- | --- |
| H | -2.64964300 | -4.37926400 | -0.46612900 |
| O | -2.91030900 | -3.45508800 | -0.53823100 |
| C | -3.09272100 | -2.95009800 | 0.78236400 |
| H | -2.23385800 | -3.21018500 | 1.41145400 |
| H | -3.99426600 | -3.36632500 | 1.24907500 |
| C | -3.22387400 | -1.43453100 | 0.78802400 |
| H | -3.20706900 | -1.14212000 | 1.84830000 |
| O | -2.07062400 | -0.87950200 | 0.13200700 |
| C | -1.92784200 | 0.53248900 | 0.30059600 |
| H | -1.82778800 | 0.76840900 | 1.37071600 |
| O | -0.81291000 | 0.94312600 | -0.41194800 |
| C | 0.47651800 | 0.79945000 | 0.21363000 |
| H | 0.36075800 | 0.72870200 | 1.30133200 |
| C | 1.16920300 | -0.46968400 | -0.30425600 |
| H | 1.07678400 | -0.45562500 | -1.40101400 |
| O | 0.58804600 | -1.65320500 | 0.22066000 |
| H | -0.37732700 | -1.59724800 | 0.10458600 |
| C | 2.65809500 | -0.48880100 | 0.06976400 |
| H | 2.75496800 | -0.61485600 | 1.14871700 |
| N | 3.33017400 | -1.60848700 | -0.56076600 |
| H | 3.26163800 | -1.69231500 | -1.56440500 |
| C | 4.01981700 | -2.56091900 | 0.11784600 |
| O | 4.14038300 | -2.55669200 | 1.34359900 |
| C | 4.64302400 | -3.65137200 | -0.72922100 |
| H | 4.46061800 | -3.53547200 | -1.79832700 |
| H | 5.72004200 | -3.65694100 | -0.54918200 |
| H | 4.24620000 | -4.61441000 | -0.40127300 |
| C | 3.32075100 | 0.84434300 | -0.30296900 |
| H | 3.30098200 | 0.99899500 | -1.39630800 |
| O | 4.62282300 | 0.84282100 | 0.16984900 |
| C | 5.42927100 | 1.91651300 | -0.33217000 |
| H | 5.03071200 | 2.88501700 | -0.02024700 |
| H | 5.48449600 | 1.87764300 | -1.42606600 |
| H | 6.42481400 | 1.77739400 | 0.08650900 |
| O | 2.61648300 | 1.91437700 | 0.32432000 |
| C | 1.27745700 | 2.06083000 | -0.14173100 |
| H | 1.26985600 | 2.18046400 | -1.23582800 |
| C | 0.68809900 | 3.30890800 | 0.50284200 |
| H | -0.39142200 | 3.32279700 | 0.32795200 |
| H | 0.86706300 | 3.26705500 | 1.57955200 |
| O | 1.30048000 | 4.51343800 | 0.04075100 |
| H | 1.02100500 | 4.67084500 | -0.86823800 |
| C | -3.15643100 | 1.21810200 | -0.28706600 |
| H | -3.16192100 | 1.09537100 | -1.37069400 |
| C | -4.44191700 | 0.68963800 | 0.33672100 |
| H | -4.45026800 | 0.95472100 | 1.40128800 |
| O | -5.59294000 | 1.22042700 | -0.31226300 |
| H | -5.54887000 | 2.18381900 | -0.29410900 |
| C | -4.50878100 | -0.84004100 | 0.20224700 |
| H | -5.35392900 | -1.19925900 | 0.80361900 |
| O | -4.67178900 | -1.21375200 | -1.15909400 |
| H | -5.37937000 | -0.66247100 | -1.51846700 |
| F | -3.07517700 | 2.60201100 | -0.01937700 |
| E(RB3LYP): -1494.60000198 | | | |
| Sum of electronic and thermal free energies: -1494.227669 | | | |
| Number of imaginary frequencies: 0 | | | |

## 2′F-LN **5** Conformer G

| **Atom** | **x** | **y** | **z** |
| --- | --- | --- | --- |
| H | -4.51275100 | 3.83342400 | -1.96082000 |
| O | -4.44412100 | 2.87835700 | -1.85999300 |
| C | -3.26811500 | 2.58548500 | -1.10396800 |
| H | -3.26772200 | 3.11171800 | -0.14531300 |
| H | -2.36221000 | 2.85293900 | -1.65894100 |
| C | -3.25751000 | 1.08405700 | -0.85596100 |
| H | -3.28970300 | 0.56544100 | -1.82366500 |
| O | -2.01542800 | 0.79487400 | -0.19611400 |
| C | -1.77983500 | -0.60237400 | -0.00777100 |
| H | -1.77673200 | -1.11015100 | -0.98376000 |
| O | -0.56375700 | -0.74744600 | 0.63663800 |
| C | 0.61751100 | -0.68940200 | -0.18668300 |
| H | 0.34074500 | -0.77214100 | -1.24109100 |
| C | 1.33953300 | 0.63822500 | 0.04899600 |
| H | 1.43658100 | 0.78105300 | 1.13081500 |
| O | 0.63726400 | 1.73393200 | -0.53101700 |
| H | -0.30971000 | 1.62302800 | -0.34124100 |
| C | 2.74232400 | 0.61263100 | -0.57926300 |
| H | 2.62391700 | 0.56701300 | -1.66400500 |
| N | 3.49328800 | 1.83121100 | -0.29661300 |
| H | 3.71635900 | 2.42025600 | -1.08247100 |
| C | 3.88443600 | 2.23677900 | 0.93621600 |
| O | 3.65653000 | 1.58129300 | 1.95583400 |
| C | 4.62667100 | 3.55595700 | 0.99784000 |
| H | 4.77888800 | 4.02123900 | 0.02334200 |
| H | 4.06197000 | 4.23974000 | 1.63532100 |
| H | 5.59673600 | 3.38624100 | 1.46934800 |
| C | 3.50155900 | -0.66052000 | -0.17222900 |
| H | 3.68534300 | -0.67875900 | 0.91073200 |
| O | 4.69328200 | -0.70607900 | -0.88056100 |
| C | 5.61598000 | -1.69395100 | -0.40480800 |
| H | 5.20586700 | -2.70068700 | -0.51833100 |
| H | 5.86242100 | -1.51546000 | 0.64802400 |
| H | 6.51609400 | -1.59500300 | -1.00983800 |
| O | 2.72263100 | -1.80393900 | -0.53218700 |
| C | 1.49580800 | -1.88663600 | 0.19373800 |
| H | 1.69630900 | -1.85196500 | 1.27475300 |
| C | 0.83180100 | -3.21855700 | -0.10181400 |
| H | -0.02012900 | -3.33326200 | 0.57543700 |
| C | -2.87509900 | -1.18470500 | 0.88157400 |
| H | -2.77997400 | -0.78732200 | 1.89315000 |
| C | -4.25620300 | -0.90932100 | 0.30757200 |
| H | -4.36500700 | -1.48677300 | -0.61727600 |
| C | -4.42904600 | 0.58925700 | -0.00777400 |
| H | -5.35128000 | 0.72207300 | -0.58591800 |
| O | 0.39874100 | -3.24352000 | -1.46654200 |
| H | -0.07762100 | -4.06639800 | -1.61740200 |
| H | 1.55493000 | -4.01762100 | 0.09525600 |
| O | -5.21609300 | -1.32834700 | 1.27886900 |
| H | -6.04958000 | -1.52008600 | 0.83363600 |
| O | -4.48377900 | 1.34788900 | 1.19607100 |
| H | -5.09022100 | 0.88897700 | 1.79227100 |
| F | -2.67751100 | -2.57707200 | 0.95136700 |
| E(RB3LYP): -1494.60304842 | | | |
| Sum of electronic and thermal free energies: -1494.230877 | | | |
| Number of imaginary frequencies: 0 | | | |

## 2′F-LN **5** Conformer H

| **Atom** | **x** | **y** | **z** |
| --- | --- | --- | --- |
| H | -5.53961800 | 2.70294800 | -2.07344000 |
| O | -5.00460500 | 1.90261000 | -2.06421300 |
| C | -4.14206800 | 1.94586700 | -0.92537000 |
| H | -4.70926200 | 2.08191600 | -0.00020700 |
| H | -3.40502600 | 2.75114500 | -1.01397200 |
| C | -3.39917200 | 0.61880100 | -0.87317400 |
| H | -2.86788600 | 0.47706900 | -1.82510600 |
| O | -2.45115700 | 0.73612900 | 0.19592700 |
| C | -1.58580700 | -0.38198500 | 0.28330500 |
| H | -1.04553200 | -0.50124700 | -0.66625500 |
| O | -0.71941300 | -0.18670200 | 1.35670400 |
| C | 0.61094400 | 0.29572900 | 1.09295200 |
| H | 1.04474700 | 0.40956300 | 2.08627200 |
| C | 1.42978500 | -0.75416600 | 0.33112400 |
| H | 0.95978000 | -0.95500500 | -0.64182000 |
| O | 1.51845300 | -1.96395100 | 1.07269000 |
| H | 0.62395900 | -2.32001200 | 1.16108000 |
| C | 2.84841300 | -0.23730500 | 0.06974600 |
| H | 3.37627700 | -0.12905900 | 1.01801600 |
| N | 3.60307700 | -1.16864000 | -0.74502000 |
| H | 3.22113200 | -1.42736800 | -1.64307800 |
| C | 4.78341600 | -1.72343200 | -0.36548500 |
| O | 5.32029900 | -1.48140200 | 0.71598400 |
| C | 5.41414800 | -2.67386800 | -1.36189700 |
| H | 4.82884000 | -2.80717300 | -2.27255100 |
| H | 6.40295900 | -2.29261400 | -1.62550500 |
| H | 5.54980600 | -3.64339800 | -0.87833900 |
| C | 2.77280000 | 1.15089700 | -0.58730500 |
| H | 2.28431800 | 1.08488100 | -1.57591500 |
| O | 4.05759900 | 1.65414300 | -0.70746700 |
| C | 4.15615000 | 2.81885400 | -1.53755100 |
| H | 3.58777000 | 3.65089700 | -1.11475600 |
| H | 3.79296100 | 2.60282400 | -2.54875400 |
| H | 5.21247700 | 3.07980600 | -1.57959400 |
| O | 2.02066000 | 2.03973900 | 0.23600800 |
| C | 0.64828600 | 1.66685000 | 0.39890100 |
| H | 0.16027300 | 1.61208200 | -0.58487900 |
| C | -0.04827700 | 2.76122500 | 1.18685800 |
| H | -1.11815100 | 2.54085500 | 1.20118400 |
| C | -2.39149100 | -1.64972700 | 0.56039300 |
| H | -2.81617600 | -1.60828100 | 1.56399200 |
| C | -3.46432200 | -1.86813600 | -0.49066900 |
| H | -2.97535200 | -2.10207900 | -1.44278300 |
| C | -4.31157200 | -0.59068100 | -0.65745300 |
| H | -4.94730100 | -0.70440600 | -1.54351300 |
| O | 0.48753000 | 2.81039100 | 2.51357200 |
| H | -0.04061700 | 3.42860300 | 3.02869300 |
| H | 0.11398900 | 3.71191900 | 0.66591600 |
| O | -4.27943600 | -2.95955800 | -0.06088000 |
| H | -4.70368400 | -3.35680500 | -0.83018100 |
| O | -5.10974100 | -0.37013800 | 0.50124200 |
| H | -5.52124200 | -1.21495700 | 0.72662500 |
| F | -1.48221100 | -2.73502700 | 0.53347200 |
| E(RB3LYP): -1494.59837809 | | | |
| Sum of electronic and thermal free energies: -1494.226604 | | | |
| Number of imaginary frequencies: 0 | | | |

## 2′F-LN **5** Conformer I

| **Atom** | **x** | **y** | **z** |
| --- | --- | --- | --- |
| H | -6.10836400 | 2.28362000 | 1.25866900 |
| O | -5.77723000 | 1.53673800 | 0.74908900 |
| C | -4.37827900 | 1.71995500 | 0.53176400 |
| H | -3.84549300 | 1.86038600 | 1.47719800 |
| H | -4.18361700 | 2.57854200 | -0.11973100 |
| C | -3.86871200 | 0.46240200 | -0.15874300 |
| H | -4.44599300 | 0.30889400 | -1.07987400 |
| O | -2.49415400 | 0.71559100 | -0.49852200 |
| C | -1.90163200 | -0.31810800 | -1.27985200 |
| H | -2.46039100 | -0.44516800 | -2.21423500 |
| O | -0.62476400 | 0.06990200 | -1.67917100 |
| C | 0.36516600 | 0.38805900 | -0.67793400 |
| H | -0.04574800 | 0.28102900 | 0.32842100 |
| C | 1.54498400 | -0.57439600 | -0.85263900 |
| H | 1.84538500 | -0.54043800 | -1.90935500 |
| O | 1.20066900 | -1.90530300 | -0.48909000 |
| H | 0.59618600 | -2.26301600 | -1.15133100 |
| C | 2.73271800 | -0.15543000 | 0.02449500 |
| H | 2.47090400 | -0.30062900 | 1.07353800 |
| N | 3.89610700 | -0.97576300 | -0.24907200 |
| H | 4.24477300 | -0.99760100 | -1.19619400 |
| C | 4.53020000 | -1.72890900 | 0.68615500 |
| O | 4.16879600 | -1.78119600 | 1.86227600 |
| C | 5.72744800 | -2.51632700 | 0.19545200 |
| H | 5.93809200 | -2.37876500 | -0.86584400 |
| H | 6.60197800 | -2.21409100 | 0.77531600 |
| H | 5.54927400 | -3.57587700 | 0.39034500 |
| C | 3.04668300 | 1.33458000 | -0.17522900 |
| H | 3.40530500 | 1.52119600 | -1.20329600 |
| O | 4.00424800 | 1.71094300 | 0.75336300 |
| C | 4.58236900 | 2.99969600 | 0.50921100 |
| H | 3.82721800 | 3.78687400 | 0.57477300 |
| H | 5.05701800 | 3.02795300 | -0.47827600 |
| H | 5.33749700 | 3.15081600 | 1.27908100 |
| O | 1.87485100 | 2.11359300 | 0.04750900 |
| C | 0.82297500 | 1.84047400 | -0.88097200 |
| H | 1.19261500 | 1.95275500 | -1.91166100 |
| C | -0.27457200 | 2.87738900 | -0.67092500 |
| H | -1.00169000 | 2.78264400 | -1.48315800 |
| C | -1.93955300 | -1.63472900 | -0.49108500 |
| H | -1.26389000 | -1.60401400 | 0.36307500 |
| C | -3.34839300 | -1.98783900 | -0.04815300 |
| H | -3.94824600 | -2.21952400 | -0.93518400 |
| C | -3.98172200 | -0.79808200 | 0.69551900 |
| H | -5.04493000 | -1.00856600 | 0.85932500 |
| O | -0.91660300 | 2.77709500 | 0.59619800 |
| H | -1.55233100 | 2.05048800 | 0.51876500 |
| H | 0.18173000 | 3.86761500 | -0.72686200 |
| O | -3.25428900 | -3.12523400 | 0.80973600 |
| H | -4.10135700 | -3.58599800 | 0.81504600 |
| O | -3.31829200 | -0.57317900 | 1.93495100 |
| H | -3.24208000 | -1.42855600 | 2.37789700 |
| F | -1.46005900 | -2.64713700 | -1.35622800 |
| E(RB3LYP): -1494.60504636 | | | |
| Sum of electronic and thermal free energies: -1494.230615 | | | |
| Number of imaginary frequencies: 0 | | | |

## 2′F-LN **5** Conformer J

| **Atom** | **x** | **y** | **z** |
| --- | --- | --- | --- |
| H | -0.84526500 | -2.45981800 | 1.69025300 |
| O | -1.59107300 | -2.83664200 | 2.17491700 |
| C | -2.75072300 | -2.73894500 | 1.34743600 |
| H | -3.57784000 | -3.17645400 | 1.90862600 |
| H | -2.62120800 | -3.30295100 | 0.41896000 |
| C | -3.07414900 | -1.28955900 | 1.01712900 |
| H | -3.16528700 | -0.72263600 | 1.95296300 |
| O | -1.94514400 | -0.79299000 | 0.27929600 |
| C | -1.98058600 | 0.61051800 | 0.01010800 |
| H | -2.05867900 | 1.17399200 | 0.94956400 |
| O | -0.81695400 | 0.92812000 | -0.67265000 |
| C | 0.40438600 | 0.80053600 | 0.08708600 |
| H | 0.18297200 | 0.46568300 | 1.10515900 |
| C | 1.23564000 | -0.28419800 | -0.61276500 |
| H | 1.22655800 | -0.04905400 | -1.68758200 |
| O | 0.68052700 | -1.57768100 | -0.39985000 |
| H | -0.28252300 | -1.50442900 | -0.49200100 |
| C | 2.68709700 | -0.30977100 | -0.12716300 |
| H | 2.72084400 | -0.64602600 | 0.90967200 |
| N | 3.47790400 | -1.23766600 | -0.91241100 |
| H | 3.49033400 | -1.11523700 | -1.91452100 |
| C | 4.18019300 | -2.27215000 | -0.38277300 |
| O | 4.20793300 | -2.51508100 | 0.82431400 |
| C | 4.93861700 | -3.12902000 | -1.37521400 |
| H | 4.84342000 | -2.79227100 | -2.40827100 |
| H | 5.99407300 | -3.13074900 | -1.09555000 |
| H | 4.57268500 | -4.15509400 | -1.29857100 |
| C | 3.26504100 | 1.11014800 | -0.17548900 |
| H | 3.27750700 | 1.49630000 | -1.20972400 |
| O | 4.54184700 | 1.09464600 | 0.35974500 |
| C | 5.28300400 | 2.30280300 | 0.14334700 |
| H | 4.80367000 | 3.15076600 | 0.63871400 |
| H | 5.37733700 | 2.50958000 | -0.92874900 |
| H | 6.27147000 | 2.13968000 | 0.56994100 |
| O | 2.45603200 | 1.96918200 | 0.62579300 |
| C | 1.12521700 | 2.15640800 | 0.13972100 |
| H | 1.13896500 | 2.59089400 | -0.86925200 |
| C | 0.51448800 | 3.14776200 | 1.12676100 |
| H | 0.54035800 | 2.70004500 | 2.12790100 |
| H | 1.14264400 | 4.04530700 | 1.13416700 |
| O | -0.82201600 | 3.45391800 | 0.73551800 |
| H | -1.17929300 | 4.09114000 | 1.36290400 |
| C | -3.18739200 | 0.86027900 | -0.88741800 |
| H | -3.03408100 | 0.36608200 | -1.84786900 |
| C | -4.46855500 | 0.37708100 | -0.21144700 |
| H | -4.63211300 | 0.98331400 | 0.68880800 |
| O | -5.58744000 | 0.47980000 | -1.08634900 |
| H | -5.65201200 | 1.38696600 | -1.40792200 |
| C | -4.35451000 | -1.09698200 | 0.20450600 |
| H | -5.21676100 | -1.34456000 | 0.83625100 |
| O | -4.31354800 | -1.94953500 | -0.93607900 |
| H | -5.03560200 | -1.68532200 | -1.52052200 |
| F | -3.33504900 | 2.23879600 | -1.14296100 |
| E(RB3LYP): -1494.60267384 | | | |
| Sum of electronic and thermal free energies: -1494.228788 | | | |
| Number of imaginary frequencies: 0 | | | |

## 2′F-LN **5** Conformer K

| **Atom** | **x** | **y** | **z** |
| --- | --- | --- | --- |
| H | 3.94776500 | 4.00849300 | 2.14900100 |
| O | 4.01608400 | 3.05749600 | 2.01522800 |
| C | 2.89084000 | 2.62538200 | 1.24843300 |
| H | 2.80500200 | 3.18709500 | 0.31391100 |
| H | 1.95891100 | 2.72935200 | 1.81448800 |
| C | 3.10147300 | 1.15158200 | 0.93446400 |
| H | 3.21847300 | 0.60127500 | 1.87743400 |
| O | 1.91011800 | 0.71105600 | 0.26737600 |
| C | 1.86430700 | -0.69768100 | 0.00821300 |
| H | 1.93839100 | -1.25502100 | 0.95177700 |
| O | 0.67031700 | -0.96043700 | -0.64225300 |
| C | -0.53163200 | -0.77784200 | 0.13540800 |
| H | -0.28115300 | -0.45104800 | 1.14970200 |
| C | -1.32496700 | 0.33744700 | -0.56041500 |
| H | -1.32963500 | 0.09682100 | -1.63447500 |
| O | -0.72374400 | 1.60996500 | -0.35595700 |
| H | 0.23948600 | 1.49180800 | -0.31160300 |
| C | -2.77429400 | 0.41791500 | -0.07027900 |
| H | -2.79166600 | 0.76420300 | 0.96353500 |
| N | -3.53194600 | 1.36868900 | -0.86153700 |
| H | -3.54619800 | 1.24160000 | -1.86301800 |
| C | -4.20573500 | 2.42527100 | -0.33917200 |
| O | -4.23168200 | 2.67451500 | 0.86679400 |
| C | -4.93314500 | 3.30087400 | -1.33864200 |
| H | -4.85196300 | 2.95173800 | -2.36879500 |
| H | -5.98731800 | 3.34478500 | -1.05780200 |
| H | -4.52886500 | 4.31323500 | -1.27183300 |
| C | -3.40650000 | -0.97830500 | -0.10402400 |
| H | -3.43990000 | -1.37270700 | -1.13464200 |
| O | -4.67845500 | -0.91050600 | 0.43907900 |
| C | -5.46492600 | -2.09256300 | 0.23950500 |
| H | -5.01154800 | -2.95353400 | 0.73691400 |
| H | -5.57753200 | -2.30388000 | -0.82995900 |
| H | -6.44253200 | -1.89051700 | 0.67443600 |
| O | -2.62499900 | -1.86025200 | 0.69954600 |
| C | -1.30813400 | -2.10288100 | 0.20142700 |
| H | -1.35002300 | -2.53863400 | -0.80628400 |
| C | -0.72886300 | -3.11762600 | 1.18330600 |
| H | -0.71106800 | -2.66396200 | 2.18200400 |
| H | -1.40235900 | -3.98138600 | 1.20921900 |
| O | 0.58112800 | -3.49448000 | 0.76605600 |
| H | 0.92167800 | -4.14045100 | 1.39375900 |
| C | 3.02438700 | -1.03427900 | -0.92359600 |
| H | 2.86435200 | -0.55394100 | -1.88983500 |
| C | 4.35386300 | -0.61188900 | -0.30823700 |
| H | 4.51691800 | -1.20130800 | 0.60324700 |
| O | 5.43215800 | -0.80338800 | -1.21948200 |
| H | 5.43133100 | -1.72127300 | -1.51617100 |
| C | 4.32894400 | 0.87448700 | 0.06440400 |
| H | 5.22795100 | 1.10772100 | 0.64544800 |
| O | 4.26316300 | 1.69129300 | -1.10131800 |
| H | 4.95280500 | 1.38855900 | -1.70532600 |
| F | 3.08065800 | -2.42458600 | -1.14996600 |
| E(RB3LYP): -1494.59947899 | | | |
| Sum of electronic and thermal free energies: -1494.227256 | | | |
| Number of imaginary frequencies: 0 | | | |

## 2′F-LN **5** Conformer L

| **Atom** | **x** | **y** | **z** |
| --- | --- | --- | --- |
| H | 1.94463000 | 4.18677500 | 1.06571400 |
| O | 2.31168500 | 3.40289600 | 0.64412500 |
| C | 2.80346600 | 2.54478700 | 1.67231600 |
| H | 2.05490900 | 2.43797300 | 2.46488100 |
| H | 3.72306000 | 2.94176700 | 2.11998700 |
| C | 3.10204100 | 1.15319200 | 1.13242500 |
| H | 3.28027600 | 0.52185500 | 2.01561200 |
| O | 1.93160500 | 0.68670800 | 0.45061200 |
| C | 1.95160700 | -0.69873500 | 0.09877300 |
| H | 2.03264500 | -1.31672300 | 1.00357100 |
| O | 0.77907700 | -0.96329600 | -0.59043300 |
| C | -0.44358200 | -0.83317800 | 0.16487900 |
| H | -0.22234800 | -0.57866900 | 1.20620600 |
| C | -1.22438200 | 0.32835500 | -0.47065300 |
| H | -1.17308600 | 0.17845800 | -1.55999400 |
| O | -0.66508500 | 1.59014300 | -0.13082900 |
| H | 0.30125300 | 1.50070600 | -0.05614000 |
| C | -2.69846900 | 0.34473700 | -0.05189700 |
| H | -2.77604200 | 0.60427900 | 1.00432300 |
| N | -3.43231400 | 1.34599900 | -0.80259600 |
| H | -3.38450300 | 1.30645600 | -1.81035700 |
| C | -4.15520900 | 2.34386800 | -0.23309600 |
| O | -4.25475600 | 2.48886300 | 0.98612100 |
| C | -4.84320600 | 3.29030900 | -1.19534100 |
| H | -4.68675900 | 3.03940400 | -2.24523000 |
| H | -5.91401700 | 3.28295800 | -0.98188300 |
| H | -4.47432900 | 4.30146900 | -1.01069400 |
| C | -3.30011900 | -1.05338600 | -0.23221600 |
| H | -3.26212600 | -1.36793400 | -1.28979100 |
| O | -4.60444300 | -1.04849900 | 0.23314200 |
| C | -5.35278700 | -2.22258300 | -0.10853900 |
| H | -4.91164000 | -3.11422200 | 0.34388500 |
| H | -5.39789700 | -2.34720900 | -1.19649100 |
| H | -6.35839000 | -2.07286200 | 0.28142300 |
| O | -2.55094000 | -1.98103800 | 0.54997900 |
| C | -1.20418100 | -2.16844600 | 0.11286800 |
| H | -1.18263100 | -2.54401800 | -0.91934100 |
| C | -0.65862800 | -3.22751100 | 1.06780800 |
| H | -0.73866700 | -2.84149100 | 2.09154800 |
| H | -1.29331000 | -4.11658900 | 0.98390900 |
| O | 0.69614300 | -3.52520000 | 0.73675700 |
| H | 1.01138000 | -4.20029800 | 1.34678300 |
| C | 3.13497900 | -0.93148800 | -0.83495900 |
| H | 2.95950100 | -0.41370800 | -1.77889200 |
| C | 4.43892900 | -0.47541000 | -0.19011200 |
| H | 4.62667000 | -1.09589500 | 0.69530200 |
| O | 5.53013000 | -0.57384200 | -1.10065500 |
| H | 5.56693600 | -1.47237000 | -1.44970200 |
| C | 4.33849600 | 0.99595100 | 0.24150600 |
| H | 5.22534900 | 1.23923200 | 0.84108600 |
| O | 4.26035700 | 1.84440000 | -0.89677000 |
| H | 4.95628100 | 1.56242300 | -1.50484500 |
| F | 3.25723600 | -2.30617800 | -1.12685100 |
| E(RB3LYP): -1494.59536611 | | | |
| Sum of electronic and thermal free energies: -1494.223554 | | | |
| Number of imaginary frequencies: 0 | | | |

## 3′F-LN **6** Conformer A

| **Atom** | **x** | **y** | **z** |
| --- | --- | --- | --- |
| H | 4.03080900 | 3.39855000 | 2.21590800 |
| O | 4.17047000 | 3.48129100 | 1.26572600 |
| C | 3.08237200 | 2.86567400 | 0.58199600 |
| H | 3.14864100 | 3.17527900 | -0.46046600 |
| H | 2.11711600 | 3.18952400 | 0.98424700 |
| C | 3.15914000 | 1.34118800 | 0.66138700 |
| H | 3.12174300 | 1.03254900 | 1.71562600 |
| O | 2.00363200 | 0.84043100 | -0.02527300 |
| C | 1.84351700 | -0.58176900 | 0.05099900 |
| H | 1.74169800 | -0.88143500 | 1.10437300 |
| O | 0.71033200 | -0.91133800 | -0.67272200 |
| C | -0.55290800 | -0.78509900 | 0.00359300 |
| H | -0.39324200 | -0.66092300 | 1.07778800 |
| C | -1.30533700 | 0.42991100 | -0.54106500 |
| H | -1.29531300 | 0.35612000 | -1.63933200 |
| O | -0.70918900 | 1.65347400 | -0.13126600 |
| H | 0.25660100 | 1.56295000 | -0.20290700 |
| C | -2.76015600 | 0.43491400 | -0.05206900 |
| H | -2.77479400 | 0.62636300 | 1.02153000 |
| N | -3.51469000 | 1.49436900 | -0.69394600 |
| H | -3.53076900 | 1.51202400 | -1.70312900 |
| C | -4.18156100 | 2.46884100 | -0.02395900 |
| O | -4.20518500 | 2.54266000 | 1.20526100 |
| C | -4.90752300 | 3.48115800 | -0.88612700 |
| H | -4.79639600 | 3.30606700 | -1.95697000 |
| H | -5.96855800 | 3.45730200 | -0.62881000 |
| H | -4.52939200 | 4.47679800 | -0.64528500 |
| C | -3.41331400 | -0.93584700 | -0.28989800 |
| H | -3.48697800 | -1.14884800 | -1.37134100 |
| O | -4.66946700 | -0.93511600 | 0.29494300 |
| C | -5.48883000 | -2.05116400 | -0.07605900 |
| H | -5.04400200 | -2.99207100 | 0.25721700 |
| H | -5.63365800 | -2.07843700 | -1.16215600 |
| H | -6.45010700 | -1.90586500 | 0.41448800 |
| O | -2.63297000 | -1.95983200 | 0.32501600 |
| C | -1.32733000 | -2.08482100 | -0.24037100 |
| H | -1.40269700 | -2.25401800 | -1.32490700 |
| C | -0.63785100 | -3.29496800 | 0.36129300 |
| H | 0.30266300 | -3.45104200 | -0.17540600 |
| C | 3.05173100 | -1.27467600 | -0.58342200 |
| H | 3.06404900 | -1.02040000 | -1.64820500 |
| C | 4.32658000 | -0.75804900 | 0.07221500 |
| H | 4.39918600 | -1.12700500 | 1.09818700 |
| C | 4.43367300 | 0.76303600 | 0.03854300 |
| H | 5.29049700 | 1.08325200 | 0.64091100 |
| O | 4.55019200 | 1.24169300 | -1.29734900 |
| H | 5.35679000 | 0.87453300 | -1.67758200 |
| O | 2.89558700 | -2.67348100 | -0.38444300 |
| H | 3.61448600 | -3.12663600 | -0.84016200 |
| F | 5.42916700 | -1.31059600 | -0.62932600 |
| O | -0.38944600 | -3.05921500 | 1.75236100 |
| H | 0.16091900 | -3.77534600 | 2.08554000 |
| H | -1.28383000 | -4.16899700 | 0.22201800 |
| E(RB3LYP): -1494.60501293 | | | |
| Sum of electronic and thermal free energies: -1494.232745 | | | |
| Number of imaginary frequencies: 0 | | | |

## 3′F-LN **6** Conformer B

| **Atom** | **x** | **y** | **z** |
| --- | --- | --- | --- |
| H | 4.19743200 | 3.61565600 | 1.77591400 |
| O | 4.32984700 | 3.56557500 | 0.82235500 |
| C | 3.21827600 | 2.89492200 | 0.23553600 |
| H | 3.29027300 | 3.04586700 | -0.84100500 |
| H | 2.26695700 | 3.30827300 | 0.58598200 |
| C | 3.24473900 | 1.39703300 | 0.53905000 |
| H | 3.20380500 | 1.25020000 | 1.62739300 |
| O | 2.06768100 | 0.83926500 | -0.06466000 |
| C | 1.86082600 | -0.55138200 | 0.21623800 |
| H | 1.74576500 | -0.68919100 | 1.30144700 |
| O | 0.72219500 | -0.95220600 | -0.46297600 |
| C | -0.54950800 | -0.76244700 | 0.18095600 |
| H | -0.41052200 | -0.64496000 | 1.26172700 |
| C | -1.22939800 | 0.49453700 | -0.37747100 |
| H | -1.17192100 | 0.42739600 | -1.47457100 |
| O | -0.59904400 | 1.68448500 | 0.07291300 |
| H | 0.35958500 | 1.59314400 | -0.06837800 |
| C | -2.70394500 | 0.56793500 | 0.04129900 |
| H | -2.76336600 | 0.74862800 | 1.11522100 |
| N | -3.37368100 | 1.66950800 | -0.62283800 |
| H | -3.35181200 | 1.69157300 | -1.63182300 |
| C | -4.01216000 | 2.67483500 | 0.02888400 |
| O | -4.07694900 | 2.74325700 | 1.25697000 |
| C | -4.65106400 | 3.72941500 | -0.85118900 |
| H | -4.51594000 | 3.55183300 | -1.91882500 |
| H | -5.71937900 | 3.76554100 | -0.62780000 |
| H | -4.22455700 | 4.70103300 | -0.59349300 |
| C | -3.40646900 | -0.76657900 | -0.24543400 |
| H | -3.42955900 | -0.97029200 | -1.33084400 |
| O | -4.69098800 | -0.71305700 | 0.27147000 |
| C | -5.53634000 | -1.79160500 | -0.14947300 |
| H | -5.14826500 | -2.75223900 | 0.19796400 |
| H | -5.62708700 | -1.80613700 | -1.24163100 |
| H | -6.51427000 | -1.60908800 | 0.29338100 |
| O | -2.70458900 | -1.82309900 | 0.40528100 |
| C | -1.38338300 | -2.02070400 | -0.09839100 |
| H | -1.41519200 | -2.19084000 | -1.18423200 |
| C | -0.79706600 | -3.24653800 | 0.57250100 |
| H | -0.94528600 | -3.16004900 | 1.65609000 |
| C | 3.04825600 | -1.36974500 | -0.29895800 |
| H | 3.06720300 | -1.28231200 | -1.38995600 |
| C | 4.34107600 | -0.80200000 | 0.27316900 |
| H | 4.40536700 | -1.01118800 | 1.34399300 |
| C | 4.49534700 | 0.69150300 | 0.00673500 |
| H | 5.36606300 | 1.07279400 | 0.55065300 |
| O | 4.61494300 | 0.95780100 | -1.38671800 |
| H | 5.41151800 | 0.51982600 | -1.70861700 |
| O | 2.84507400 | -2.71528900 | 0.11187700 |
| H | 3.53745500 | -3.26101800 | -0.27869600 |
| F | 5.42229200 | -1.49092600 | -0.33380100 |
| O | -1.46169400 | -4.40561000 | 0.05654300 |
| H | -1.08174900 | -5.18403000 | 0.47740800 |
| H | 0.27569300 | -3.28270100 | 0.36161400 |
| E(RB3LYP): -1494.60414604 | | | |
| Sum of electronic and thermal free energies: -1494.232105 | | | |
| Number of imaginary frequencies: 0 | | | |

## 3′F-LN **6** Conformer C

| **Atom** | **x** | **y** | **z** |
| --- | --- | --- | --- |
| H | -1.00246400 | -2.74032800 | 1.38171400 |
| O | -1.76791700 | -3.14348200 | 1.81153900 |
| C | -2.92531500 | -2.87257000 | 1.02090400 |
| H | -3.77022100 | -3.33868600 | 1.53000500 |
| H | -2.83476200 | -3.30921300 | 0.02172600 |
| C | -3.17234500 | -1.37682100 | 0.89786300 |
| H | -3.18794000 | -0.93983100 | 1.90509200 |
| O | -2.05303000 | -0.84555900 | 0.17103800 |
| C | -2.01035700 | 0.57792700 | 0.10488900 |
| H | -1.97801400 | 0.99009500 | 1.12504600 |
| O | -0.87481300 | 0.92626800 | -0.61377600 |
| C | 0.38238900 | 0.76082200 | 0.06990900 |
| H | 0.21033300 | 0.49779900 | 1.11900700 |
| C | 1.16105400 | -0.37245500 | -0.60851700 |
| H | 1.16696400 | -0.15635700 | -1.68759000 |
| O | 0.56270500 | -1.64230800 | -0.37872700 |
| H | -0.39357400 | -1.54037400 | -0.50987700 |
| C | 2.60724800 | -0.43691500 | -0.10196000 |
| H | 2.60892400 | -0.75975000 | 0.93990000 |
| N | 3.37545800 | -1.40662400 | -0.85867300 |
| H | 3.41257200 | -1.29752300 | -1.86165500 |
| C | 4.02654900 | -2.46066300 | -0.30235900 |
| O | 4.02085500 | -2.68887400 | 0.90774600 |
| C | 4.77056100 | -3.35892800 | -1.26877800 |
| H | 4.71009500 | -3.03181200 | -2.30751600 |
| H | 5.81913000 | -3.39875600 | -0.96695400 |
| H | 4.36270200 | -4.36873700 | -1.18775200 |
| C | 3.25373200 | 0.95591400 | -0.15902900 |
| H | 3.34641400 | 1.30099000 | -1.20373700 |
| O | 4.49679100 | 0.89213500 | 0.44894700 |
| C | 5.31447600 | 2.04970700 | 0.23180300 |
| H | 4.85150600 | 2.94231700 | 0.65960500 |
| H | 5.48696700 | 2.20344200 | -0.83952100 |
| H | 6.26399800 | 1.85503100 | 0.72799500 |
| O | 2.45015500 | 1.88904100 | 0.56101400 |
| C | 1.15585600 | 2.08362600 | -0.01015800 |
| H | 1.24814100 | 2.38156000 | -1.06452900 |
| C | 0.46143100 | 3.19485900 | 0.75079500 |
| H | 0.53100600 | 2.98791000 | 1.82573300 |
| C | -3.24720600 | 1.08667800 | -0.63723600 |
| H | -3.19129200 | 0.72169200 | -1.66799900 |
| C | -4.49796200 | 0.51958800 | 0.03024100 |
| H | -4.65227000 | 0.99692900 | 1.00114500 |
| C | -4.47175900 | -1.00200500 | 0.18184800 |
| H | -5.32237400 | -1.31282400 | 0.80014400 |
| O | -4.50442900 | -1.65252300 | -1.08333100 |
| H | -5.31786700 | -1.39477000 | -1.53327600 |
| O | -3.23400900 | 2.50611700 | -0.58479500 |
| H | -3.96353800 | 2.83800700 | -1.12105400 |
| F | -5.61108500 | 0.87580600 | -0.77188900 |
| O | 1.09903300 | 4.43325700 | 0.42464100 |
| H | 0.64753600 | 5.14026700 | 0.89774300 |
| H | -0.59324300 | 3.21187900 | 0.45865500 |
| E(RB3LYP): -1494.60570090 | | | |
| Sum of electronic and thermal free energies: -1494.232863 | | | |
| Number of imaginary frequencies: 0 | | | |

## 3′F-LN **6** Conformer D

| **Atom** | **x** | **y** | **z** |
| --- | --- | --- | --- |
| H | 0.84915500 | 2.60533100 | 1.42189400 |
| O | 1.58513700 | 3.00782200 | 1.90132300 |
| C | 2.77543900 | 2.82208300 | 1.13513100 |
| H | 3.58939500 | 3.27712500 | 1.70152700 |
| H | 2.70341200 | 3.32066400 | 0.16385300 |
| C | 3.06980500 | 1.34484100 | 0.92309800 |
| H | 3.08110800 | 0.84387200 | 1.90051200 |
| O | 1.98094800 | 0.83117800 | 0.14233100 |
| C | 1.98155000 | -0.58686500 | -0.01768900 |
| H | 1.93856600 | -1.07042900 | 0.96957600 |
| O | 0.86488600 | -0.91056500 | -0.77689000 |
| C | -0.39355700 | -0.81075000 | -0.08361200 |
| H | -0.22357900 | -0.57916400 | 0.97026900 |
| C | -1.22102900 | 0.30931000 | -0.71473000 |
| H | -1.25215500 | 0.12836900 | -1.79983800 |
| O | -0.65884800 | 1.59275200 | -0.46044000 |
| H | 0.30148100 | 1.51702500 | -0.57723000 |
| C | -2.65115900 | 0.30293300 | -0.16081100 |
| H | -2.62867900 | 0.59726100 | 0.88918600 |
| N | -3.48074300 | 1.26232200 | -0.86406700 |
| H | -3.53627700 | 1.18657300 | -1.86926500 |
| C | -4.16158500 | 2.26806600 | -0.25659400 |
| O | -4.13808300 | 2.45396500 | 0.96055400 |
| C | -4.96453500 | 3.16684200 | -1.17423800 |
| H | -4.89433600 | 2.89365200 | -2.22786000 |
| H | -6.01122200 | 3.13107200 | -0.86497900 |
| H | -4.61512900 | 4.19349600 | -1.04673400 |
| C | -3.24951100 | -1.11201000 | -0.23583200 |
| H | -3.36624900 | -1.42896300 | -1.28734100 |
| O | -4.47381700 | -1.10752600 | 0.41235600 |
| C | -5.26359400 | -2.28138600 | 0.18126900 |
| H | -4.76386000 | -3.17288500 | 0.56822800 |
| H | -5.46153400 | -2.40746000 | -0.88928700 |
| H | -6.20413900 | -2.13015000 | 0.70875400 |
| O | -2.39388500 | -2.04237100 | 0.42754800 |
| C | -1.10977300 | -2.16052500 | -0.18829700 |
| H | -1.22549000 | -2.42486800 | -1.24986300 |
| C | -0.33862300 | -3.28454300 | 0.47781000 |
| H | 0.56670000 | -3.47044300 | -0.10877100 |
| C | 3.24401000 | -1.00723200 | -0.77099500 |
| H | 3.19405200 | -0.57473900 | -1.77569400 |
| C | 4.46626100 | -0.45048600 | -0.04424200 |
| H | 4.62044900 | -0.98798100 | 0.89459100 |
| C | 4.39278000 | 1.05575000 | 0.20982800 |
| H | 5.22296100 | 1.34760200 | 0.86424300 |
| O | 4.43027300 | 1.79126800 | -1.00810300 |
| H | 5.25346100 | 1.57684400 | -1.46301500 |
| O | 3.27469200 | -2.42705700 | -0.81617900 |
| H | 4.02581700 | -2.69831700 | -1.35666200 |
| F | 5.60309100 | -0.71813900 | -0.84872600 |
| O | -0.00242600 | -2.90316800 | 1.81652100 |
| H | 0.52456500 | -3.60712300 | 2.20859900 |
| H | -0.96097900 | -4.18592000 | 0.46957000 |
| E(RB3LYP): -1494.60698214 | | | |
| Sum of electronic and thermal free energies: -1494.234255 | | | |
| Number of imaginary frequencies: 0 | | | |

## 3′F-LN **6** Conformer E

| **Atom** | **x** | **y** | **z** |
| --- | --- | --- | --- |
| H | 2.47661000 | 4.42174700 | 0.04818200 |
| O | 2.78432800 | 3.53033000 | -0.14617800 |
| C | 2.97216700 | 2.85505700 | 1.09521200 |
| H | 2.10174400 | 3.00304700 | 1.74404300 |
| H | 3.85880700 | 3.22772700 | 1.62345600 |
| C | 3.14290300 | 1.35605200 | 0.89718500 |
| H | 3.12916200 | 0.92478500 | 1.90964200 |
| O | 2.01693700 | 0.86303500 | 0.16006500 |
| C | 1.90944100 | -0.56303400 | 0.13221900 |
| H | 1.79435100 | -0.94292600 | 1.15837400 |
| O | 0.80466000 | -0.87892300 | -0.64122700 |
| C | -0.47788900 | -0.81321200 | 0.00646600 |
| H | -0.34896600 | -0.75563500 | 1.09054400 |
| C | -1.23896800 | 0.42213000 | -0.47991500 |
| H | -1.19912900 | 0.41845100 | -1.58007000 |
| O | -0.68146200 | 1.62883100 | 0.02240600 |
| H | 0.28877600 | 1.56590100 | -0.02799700 |
| C | -2.70612000 | 0.36780900 | -0.03113400 |
| H | -2.75157100 | 0.48416500 | 1.05230600 |
| N | -3.46384000 | 1.45679800 | -0.61754700 |
| H | -3.44539700 | 1.55331200 | -1.62220500 |
| C | -4.16894000 | 2.36753000 | 0.10101900 |
| O | -4.23363000 | 2.34822800 | 1.33088500 |
| C | -4.88509000 | 3.43003100 | -0.70719600 |
| H | -4.73341700 | 3.33873800 | -1.78348100 |
| H | -5.95371300 | 3.36817400 | -0.49078500 |
| H | -4.53545500 | 4.41076900 | -0.37830100 |
| C | -3.32661200 | -0.99468700 | -0.37796600 |
| H | -3.36541400 | -1.13790100 | -1.47264700 |
| O | -4.59851800 | -1.05383400 | 0.16893600 |
| C | -5.38532300 | -2.15848600 | -0.29483000 |
| H | -4.93194400 | -3.11066800 | -0.00829300 |
| H | -5.49942000 | -2.11980700 | -1.38423600 |
| H | -6.36252700 | -2.06138300 | 0.17578600 |
| O | -2.54447500 | -2.04294700 | 0.19215100 |
| C | -1.22133100 | -2.10769500 | -0.34128400 |
| H | -1.26290600 | -2.20885100 | -1.43607900 |
| C | -0.52620700 | -3.34212800 | 0.20201100 |
| H | 0.43022100 | -3.44971600 | -0.31829800 |
| C | 3.15513700 | -1.16829500 | -0.51864000 |
| H | 3.17711000 | -0.84687700 | -1.56504000 |
| C | 4.39783600 | -0.64655400 | 0.19289400 |
| H | 4.46928200 | -1.07496400 | 1.19545400 |
| C | 4.44987900 | 0.88107500 | 0.24773800 |
| H | 5.28331000 | 1.18550100 | 0.89305800 |
| O | 4.59844700 | 1.42726300 | -1.05514700 |
| H | 5.39428400 | 1.04403700 | -1.44383900 |
| O | 3.05068100 | -2.58188400 | -0.41103500 |
| H | 3.79380000 | -2.97762000 | -0.88113300 |
| F | 5.53195500 | -1.11444600 | -0.52014300 |
| O | -0.31813900 | -3.19052000 | 1.61121500 |
| H | 0.22870300 | -3.92200800 | 1.91554000 |
| H | -1.15294200 | -4.21575500 | -0.00910700 |
| E(RB3LYP): -1494.59996399 | | | |
| Sum of electronic and thermal free energies: -1494.228347 | | | |
| Number of imaginary frequencies: 0 | | | |

## 3′F-LN **6** Conformer F

| **Atom** | **x** | **y** | **z** |
| --- | --- | --- | --- |
| H | -2.63774800 | -4.38531100 | -0.42797300 |
| O | -2.91153600 | -3.46566600 | -0.50872800 |
| C | -3.09487500 | -2.94894100 | 0.80710400 |
| H | -2.23606400 | -3.20213300 | 1.43894500 |
| H | -3.99639900 | -3.36139700 | 1.27717300 |
| C | -3.22522000 | -1.43288000 | 0.79879800 |
| H | -3.21002400 | -1.13138500 | 1.85683600 |
| O | -2.07847000 | -0.88195700 | 0.13609000 |
| C | -1.93139700 | 0.53256500 | 0.29006900 |
| H | -1.81468000 | 0.77346000 | 1.35752500 |
| O | -0.81573100 | 0.91906300 | -0.43514900 |
| C | 0.47081500 | 0.78733100 | 0.19402700 |
| H | 0.35253300 | 0.70988600 | 1.28092300 |
| C | 1.17410700 | -0.47401000 | -0.32630400 |
| H | 1.09305500 | -0.45245900 | -1.42387900 |
| O | 0.58911400 | -1.66259600 | 0.18359200 |
| H | -0.37701500 | -1.59834100 | 0.07769600 |
| C | 2.65862100 | -0.48934100 | 0.06339700 |
| H | 2.74467200 | -0.62093700 | 1.14258400 |
| N | 3.34317000 | -1.60212100 | -0.56627200 |
| H | 3.28389800 | -1.68167800 | -1.57083700 |
| C | 4.03646900 | -2.55106600 | 0.11329700 |
| O | 4.14831600 | -2.55113000 | 1.33996200 |
| C | 4.67590800 | -3.63204300 | -0.73384900 |
| H | 4.49563800 | -3.51615100 | -1.80331800 |
| H | 5.75235600 | -3.62444100 | -0.55029100 |
| H | 4.29020200 | -4.60064200 | -0.40911500 |
| C | 3.31778500 | 0.84978700 | -0.29541500 |
| H | 3.30876900 | 1.00915000 | -1.38831300 |
| O | 4.61540800 | 0.85248300 | 0.19059900 |
| C | 5.42052300 | 1.93386100 | -0.29654300 |
| H | 5.01317900 | 2.89814000 | 0.01717500 |
| H | 5.48712100 | 1.90221700 | -1.39006900 |
| H | 6.41262000 | 1.79799200 | 0.13134300 |
| O | 2.60243100 | 1.91289500 | 0.32979400 |
| C | 1.26568900 | 2.05498200 | -0.15034300 |
| H | 1.26888200 | 2.18481100 | -1.24222500 |
| C | 0.65990500 | 3.28900200 | 0.48760500 |
| H | 0.83139400 | 3.24664800 | 1.57038800 |
| C | -3.15644900 | 1.24901900 | -0.28390600 |
| H | -3.17536500 | 1.06507400 | -1.36292500 |
| C | -4.42018800 | 0.67462600 | 0.34499500 |
| H | -4.49189900 | 0.97347600 | 1.39340200 |
| C | -4.51226000 | -0.84566600 | 0.20496300 |
| H | -5.35966400 | -1.20628700 | 0.80131800 |
| O | -4.66130500 | -1.21974400 | -1.15700300 |
| H | -5.44470500 | -0.77196400 | -1.49920600 |
| O | -3.01580500 | 2.63342900 | 0.00548900 |
| H | -3.73329700 | 3.10927700 | -0.42872200 |
| F | -5.53284400 | 1.25966100 | -0.31235700 |
| O | 1.28148800 | 4.44577200 | -0.08356100 |
| H | 0.88543500 | 5.22856700 | 0.31371600 |
| H | -0.41738500 | 3.28857400 | 0.29691300 |
| E(RB3LYP): -1494.59906296 | | | |
| Sum of electronic and thermal free energies: -1494.227486 | | | |
| Number of imaginary frequencies: 0 | | | |

## 3′F-LN **6** Conformer G

| **Atom** | **x** | **y** | **z** |
| --- | --- | --- | --- |
| H | -4.52431400 | 3.88192800 | -1.86396200 |
| O | -4.45216400 | 2.92457100 | -1.79128500 |
| C | -3.27242100 | 2.61327500 | -1.04909700 |
| H | -3.26653900 | 3.11601300 | -0.07801900 |
| H | -2.36902500 | 2.89213800 | -1.60235700 |
| C | -3.26186900 | 1.10647200 | -0.83778700 |
| H | -3.30221700 | 0.61197200 | -1.81759900 |
| O | -2.02115300 | 0.79689300 | -0.18984200 |
| C | -1.78101800 | -0.60602600 | -0.01989900 |
| H | -1.76382100 | -1.09485300 | -1.00519500 |
| O | -0.56660200 | -0.74368900 | 0.62814300 |
| C | 0.61695800 | -0.69002100 | -0.18961700 |
| H | 0.34562600 | -0.77870100 | -1.24503800 |
| C | 1.33815700 | 0.63832600 | 0.04131100 |
| H | 1.43376900 | 0.78655000 | 1.12256700 |
| O | 0.63497900 | 1.73056700 | -0.54453900 |
| H | -0.31146600 | 1.62017700 | -0.35101900 |
| C | 2.74153100 | 0.60793400 | -0.58458200 |
| H | 2.62538300 | 0.55071200 | -1.66903900 |
| N | 3.49191700 | 1.82957300 | -0.31376900 |
| H | 3.71681800 | 2.40970400 | -1.10570900 |
| C | 3.88089600 | 2.24879800 | 0.91509800 |
| O | 3.65120700 | 1.60478300 | 1.94159300 |
| C | 4.62362500 | 3.56832900 | 0.96318900 |
| H | 4.77481200 | 4.02425600 | -0.01587300 |
| H | 4.06013600 | 4.25835200 | 1.59495700 |
| H | 5.59429700 | 3.40271200 | 1.43498800 |
| C | 3.49962100 | -0.66111600 | -0.16168300 |
| H | 3.67720600 | -0.66861000 | 0.92249300 |
| O | 4.69582200 | -0.71175200 | -0.86241600 |
| C | 5.61676500 | -1.69387000 | -0.37183100 |
| H | 5.20915400 | -2.70228400 | -0.47939500 |
| H | 5.85563500 | -1.50590700 | 0.68112700 |
| H | 6.52094400 | -1.59865000 | -0.97139900 |
| O | 2.72455000 | -1.80899200 | -0.51526000 |
| C | 1.49191600 | -1.88578600 | 0.20208200 |
| H | 1.68464800 | -1.84548000 | 1.28439700 |
| C | 0.82475200 | -3.21673900 | -0.08983100 |
| H | -0.04597700 | -3.31089700 | 0.56608700 |
| C | -2.87947200 | -1.21825600 | 0.85251300 |
| H | -2.81235100 | -0.76535500 | 1.84683200 |
| C | -4.23627300 | -0.89471400 | 0.23961500 |
| H | -4.37093100 | -1.45228000 | -0.69074200 |
| C | -4.43671000 | 0.59901800 | 0.00539100 |
| H | -5.36250600 | 0.76079000 | -0.55665600 |
| O | -2.65430400 | -2.62101300 | 0.90344600 |
| H | -3.29938200 | -3.00966300 | 1.50566700 |
| O | 0.42451600 | -3.25880200 | -1.46507900 |
| H | -0.09693400 | -4.05620700 | -1.60253500 |
| H | 1.53592400 | -4.01948700 | 0.13485000 |
| O | -4.46234400 | 1.31464700 | 1.23606400 |
| H | -5.20195800 | 0.98431900 | 1.75925400 |
| F | -5.23835200 | -1.35663500 | 1.13132000 |
| E(RB3LYP): -1494.60294908 | | | |
| Sum of electronic and thermal free energies: -1494.231081 | | | |
| Number of imaginary frequencies: 0 | | | |

## 3′F-LN **6** Conformer H

| **Atom** | **x** | **y** | **z** |
| --- | --- | --- | --- |
| H | -5.81577700 | 2.52931000 | -1.94935800 |
| O | -5.22445800 | 1.76984400 | -1.96874500 |
| C | -4.32350400 | 1.86670500 | -0.86390800 |
| H | -4.86176300 | 1.96614400 | 0.08279600 |
| H | -3.64116900 | 2.71562000 | -0.97830000 |
| C | -3.49812600 | 0.58855000 | -0.84380400 |
| H | -2.99417200 | 0.48132500 | -1.81482800 |
| O | -2.52546400 | 0.75664300 | 0.19276200 |
| C | -1.58946300 | -0.30733800 | 0.26184500 |
| H | -1.05198600 | -0.38182300 | -0.69332100 |
| O | -0.73022400 | -0.04504700 | 1.32649500 |
| C | 0.61091000 | 0.38783000 | 1.04728900 |
| H | 1.04714700 | 0.52503400 | 2.03667400 |
| C | 1.40775200 | -0.70101200 | 0.31533100 |
| H | 0.95132700 | -0.88644100 | -0.66771200 |
| O | 1.43024700 | -1.90616300 | 1.06136900 |
| H | 0.52808300 | -2.26996100 | 1.03720300 |
| C | 2.84673600 | -0.22729500 | 0.07935600 |
| H | 3.35662100 | -0.12022000 | 1.03758000 |
| N | 3.59266500 | -1.19111000 | -0.70537900 |
| H | 3.21670000 | -1.46034400 | -1.60287100 |
| C | 4.74817900 | -1.77298200 | -0.29312300 |
| O | 5.27241300 | -1.52627700 | 0.79373600 |
| C | 5.36928800 | -2.76022200 | -1.25956000 |
| H | 4.79546500 | -2.89659500 | -2.17706200 |
| H | 6.37211900 | -2.41089500 | -1.51435200 |
| H | 5.47099100 | -3.72286400 | -0.75440500 |
| C | 2.82893700 | 1.15137400 | -0.60283800 |
| H | 2.37489700 | 1.07713000 | -1.60724800 |
| O | 4.12981200 | 1.62056400 | -0.68640900 |
| C | 4.28812300 | 2.76301100 | -1.53765500 |
| H | 3.72462100 | 3.61791100 | -1.15586900 |
| H | 3.95848900 | 2.53220800 | -2.55709500 |
| H | 5.35132900 | 2.99817100 | -1.54489000 |
| O | 2.07027800 | 2.07762600 | 0.17267100 |
| C | 0.68730200 | 1.73109300 | 0.30497200 |
| H | 0.23336800 | 1.64257000 | -0.69321300 |
| C | -0.02486200 | 2.86184300 | 1.02294200 |
| H | -1.09717100 | 2.64977700 | 1.00584700 |
| C | -2.30298400 | -1.63619500 | 0.54245900 |
| H | -2.75648600 | -1.57305600 | 1.53563600 |
| C | -3.38352300 | -1.86102800 | -0.50215100 |
| H | -2.93371300 | -2.07884800 | -1.47394900 |
| C | -4.33639400 | -0.67367100 | -0.60351100 |
| H | -5.00623500 | -0.81398500 | -1.45817200 |
| O | -1.30727500 | -2.65971100 | 0.49568300 |
| H | -1.69628200 | -3.49243200 | 0.78802600 |
| O | 0.46086700 | 2.95782000 | 2.36615100 |
| H | -0.06978000 | 3.61210000 | 2.83185500 |
| H | 0.16498600 | 3.79061300 | 0.47263600 |
| O | -5.08079500 | -0.50920400 | 0.59930600 |
| H | -5.60395900 | -1.30705700 | 0.73935600 |
| F | -4.12219300 | -3.01537400 | -0.13526000 |
| E(RB3LYP): -1494.60120478 | | | |
| Sum of electronic and thermal free energies: -1494.229138 | | | |
| Number of imaginary frequencies: 0 | | | |

## 3′F-LN **6** Conformer I

| **Atom** | **x** | **y** | **z** |
| --- | --- | --- | --- |
| H | -6.16556900 | 2.29324300 | 1.13160900 |
| O | -5.82222200 | 1.54213300 | 0.63649800 |
| C | -4.41878700 | 1.72340700 | 0.45068300 |
| H | -3.90927800 | 1.88029800 | 1.40607000 |
| H | -4.20768500 | 2.56965100 | -0.21152100 |
| C | -3.89244400 | 0.45496300 | -0.20569500 |
| H | -4.44723600 | 0.28540800 | -1.13758300 |
| O | -2.51022500 | 0.69679500 | -0.51388200 |
| C | -1.89364900 | -0.35514600 | -1.25766800 |
| H | -2.43573300 | -0.50866600 | -2.19773200 |
| O | -0.61699600 | 0.03779300 | -1.64793200 |
| C | 0.37232800 | 0.36476100 | -0.64801700 |
| H | -0.03348100 | 0.25109200 | 0.35957800 |
| C | 1.56035000 | -0.58642800 | -0.83282800 |
| H | 1.84402400 | -0.54796100 | -1.89553100 |
| O | 1.24463600 | -1.91837500 | -0.46148700 |
| H | 0.48438200 | -2.22653300 | -0.98362100 |
| C | 2.75698500 | -0.14848100 | 0.02324400 |
| H | 2.51389500 | -0.29556600 | 1.07638800 |
| N | 3.92518600 | -0.95657500 | -0.26882300 |
| H | 4.24652400 | -0.98956100 | -1.22525300 |
| C | 4.59228500 | -1.69102200 | 0.65773500 |
| O | 4.26350000 | -1.73348600 | 1.84398100 |
| C | 5.78465400 | -2.47174700 | 0.14438000 |
| H | 5.96977300 | -2.33945600 | -0.92235400 |
| H | 6.66973700 | -2.15883200 | 0.70208300 |
| H | 5.61975600 | -3.53152200 | 0.34978100 |
| C | 3.05027800 | 1.34423800 | -0.18219600 |
| H | 3.38805700 | 1.53621600 | -1.21628400 |
| O | 4.01939100 | 1.73342900 | 0.72933900 |
| C | 4.57527800 | 3.02989800 | 0.47552000 |
| H | 3.81069100 | 3.80672300 | 0.55439900 |
| H | 5.03222200 | 3.06486800 | -0.52010500 |
| H | 5.34162800 | 3.19153200 | 1.23209500 |
| O | 1.87203100 | 2.10766400 | 0.06150900 |
| C | 0.81178400 | 1.82241300 | -0.85375600 |
| H | 1.16817900 | 1.93915300 | -1.88861100 |
| C | -0.29487800 | 2.84694800 | -0.63104100 |
| H | -1.02344100 | 2.75317800 | -1.44181900 |
| C | -1.93874700 | -1.65129900 | -0.42817000 |
| H | -1.36419200 | -1.51500800 | 0.49243200 |
| C | -3.38150200 | -1.95600600 | -0.06092000 |
| H | -3.95718300 | -2.22723600 | -0.94915500 |
| C | -4.03524800 | -0.79094300 | 0.67411800 |
| H | -5.10096900 | -0.99626700 | 0.81611200 |
| O | -1.36591100 | -2.68514300 | -1.22925300 |
| H | -1.49507800 | -3.52897100 | -0.77693100 |
| O | -0.93367900 | 2.72818600 | 0.63647400 |
| H | -1.57100700 | 2.00408500 | 0.54865400 |
| H | 0.15239000 | 3.84177300 | -0.67857400 |
| O | -3.39335800 | -0.54357100 | 1.91979600 |
| H | -3.51533600 | -1.31719300 | 2.48238100 |
| F | -3.38231200 | -3.09724100 | 0.78028700 |
| wE(RB3LYP): -1494.60775596 | | | |
| Sum of electronic and thermal free energies: -1494.232306 | | | |
| Number of imaginary frequencies: 0 | | | |

## 3′F-LN **6** Conformer J

| **Atom** | **x** | **y** | **z** |
| --- | --- | --- | --- |
| H | 1.09974400 | 3.62874700 | -0.23580200 |
| O | 1.81881600 | 3.80709400 | 0.37909300 |
| C | 3.00053800 | 3.15058700 | -0.08050900 |
| H | 3.85135000 | 3.70574900 | 0.31966200 |
| H | 3.06342400 | 3.15989000 | -1.17084000 |
| C | 3.08139700 | 1.71589400 | 0.42320300 |
| H | 2.93294200 | 1.71442900 | 1.51089900 |
| O | 2.00826200 | 0.98204300 | -0.20007500 |
| C | 1.88670600 | -0.37250400 | 0.24669200 |
| H | 1.69879600 | -0.38616700 | 1.33037100 |
| O | 0.84336500 | -0.96771900 | -0.44819300 |
| C | -0.47465500 | -0.91527500 | 0.12646600 |
| H | -0.39870000 | -0.90248900 | 1.22017400 |
| C | -1.21807500 | 0.34593500 | -0.34177500 |
| H | -1.14854600 | 0.37451700 | -1.43948100 |
| O | -0.66196500 | 1.52930000 | 0.21465000 |
| H | 0.29089800 | 1.53468800 | 0.01405500 |
| C | -2.69598900 | 0.30703400 | 0.07088800 |
| H | -2.76334400 | 0.41551800 | 1.15403000 |
| N | -3.42469800 | 1.41136800 | -0.52285400 |
| H | -3.38930800 | 1.51131300 | -1.52678200 |
| C | -4.11567100 | 2.33798700 | 0.18992200 |
| O | -4.19592500 | 2.31634500 | 1.41860000 |
| C | -4.79465400 | 3.42012400 | -0.62409300 |
| H | -4.63590200 | 3.32483300 | -1.69899800 |
| H | -5.86641800 | 3.38872800 | -0.41716700 |
| H | -4.42004400 | 4.39045300 | -0.29175500 |
| C | -3.32616700 | -1.04197000 | -0.29828400 |
| H | -3.36644600 | -1.16985900 | -1.39412300 |
| O | -4.59638900 | -1.10306000 | 0.25074600 |
| C | -5.39186800 | -2.19424500 | -0.23101600 |
| H | -4.94177600 | -3.15415500 | 0.03389400 |
| H | -5.51170800 | -2.13267000 | -1.31863900 |
| H | -6.36575700 | -2.10146500 | 0.24714200 |
| O | -2.54459500 | -2.09897600 | 0.25644200 |
| C | -1.22465300 | -2.18216900 | -0.29876500 |
| H | -1.31021000 | -2.24262200 | -1.39232800 |
| C | -0.66880400 | -3.50788200 | 0.23896400 |
| H | -1.35945700 | -4.29604800 | -0.06531100 |
| C | 3.17178900 | -1.13580200 | -0.07651800 |
| H | 3.28524000 | -1.16700100 | -1.16409700 |
| C | 4.35623300 | -0.40987800 | 0.54221100 |
| H | 4.32320500 | -0.48999400 | 1.63120200 |
| C | 4.42700200 | 1.05312600 | 0.11109300 |
| H | 5.20862900 | 1.55833200 | 0.69075900 |
| O | 4.68075600 | 1.16792300 | -1.28473300 |
| H | 5.52702800 | 0.74501700 | -1.47315400 |
| O | 3.01744800 | -2.44464700 | 0.46517000 |
| H | 3.76222300 | -2.99102700 | 0.18616500 |
| F | 5.53978100 | -1.06545000 | 0.12467900 |
| O | 0.61250300 | -3.86801400 | -0.24999000 |
| H | 1.28845100 | -3.23799900 | 0.04686600 |
| H | -0.67644100 | -3.45933300 | 1.33658200 |
| E(RB3LYP): -1494.60897803 | | | |
| Sum of electronic and thermal free energies:-1494.234368 | | | |
| Number of imaginary frequencies: 0 | | | |

## 3′F-LN **6** Conformer K

| **Atom** | **x** | **y** | **z** |
| --- | --- | --- | --- |
| H | 3.91923100 | 3.96781900 | 1.32806900 |
| O | 4.13119200 | 3.76777300 | 0.40925000 |
| C | 3.07603600 | 2.99219300 | -0.15119300 |
| H | 3.22859200 | 2.98235300 | -1.22976000 |
| H | 2.09614400 | 3.43169000 | 0.06106500 |
| C | 3.09741900 | 1.55657700 | 0.37419400 |
| H | 2.96880900 | 1.56912300 | 1.46556100 |
| O | 1.97779700 | 0.88744000 | -0.23215100 |
| C | 1.77944900 | -0.45270800 | 0.23005700 |
| H | 1.58737800 | -0.44130300 | 1.31283400 |
| O | 0.70573400 | -0.99654300 | -0.46100800 |
| C | -0.60593300 | -0.87969400 | 0.11794000 |
| H | -0.52429300 | -0.85450300 | 1.21096900 |
| C | -1.29690200 | 0.40566900 | -0.36565800 |
| H | -1.24181100 | 0.41035100 | -1.46459100 |
| O | -0.68419700 | 1.57359700 | 0.16026100 |
| H | 0.25543500 | 1.56477900 | -0.09161800 |
| C | -2.76943500 | 0.43868500 | 0.06752600 |
| H | -2.81640300 | 0.56051500 | 1.15035200 |
| N | -3.45863000 | 1.56787400 | -0.52604000 |
| H | -3.44639300 | 1.65012900 | -1.53207400 |
| C | -4.08751500 | 2.53566600 | 0.18928900 |
| O | -4.13514100 | 2.53660500 | 1.41999000 |
| C | -4.73856900 | 3.63569900 | -0.62355300 |
| H | -4.62054400 | 3.51344300 | -1.70095900 |
| H | -5.80305300 | 3.66186200 | -0.38144700 |
| H | -4.30551300 | 4.59187400 | -0.32253900 |
| C | -3.46397600 | -0.88432900 | -0.27888200 |
| H | -3.52167700 | -1.02286500 | -1.37275400 |
| O | -4.72988800 | -0.88192200 | 0.28354300 |
| C | -5.57836900 | -1.94153100 | -0.17775900 |
| H | -5.16921900 | -2.91767900 | 0.09409300 |
| H | -5.70613500 | -1.88702200 | -1.26487400 |
| H | -6.54231800 | -1.79963300 | 0.30842100 |
| O | -2.72507900 | -1.96888000 | 0.28028100 |
| C | -1.41512700 | -2.11750100 | -0.28558600 |
| H | -1.51407200 | -2.18597100 | -1.37761300 |
| C | -0.91751300 | -3.46224100 | 0.26170800 |
| H | -1.64833600 | -4.21922600 | -0.02774600 |
| C | 3.01898500 | -1.29621300 | -0.07639000 |
| H | 3.12703000 | -1.36112200 | -1.16294100 |
| C | 4.24835900 | -0.62911900 | 0.52030000 |
| H | 4.21805400 | -0.68250700 | 1.61113900 |
| C | 4.39870100 | 0.81517600 | 0.05353500 |
| H | 5.21369700 | 1.29521500 | 0.60531800 |
| O | 4.63019300 | 0.87990000 | -1.34915800 |
| H | 5.46211400 | 0.43017500 | -1.53775400 |
| O | 2.78778300 | -2.58061300 | 0.49731800 |
| H | 3.50819700 | -3.17188200 | 0.24724800 |
| F | 5.38748500 | -1.36635100 | 0.11258200 |
| O | 0.34003900 | -3.88781600 | -0.23504100 |
| H | 1.04961400 | -3.29575100 | 0.06242100 |
| H | -0.91258800 | -3.40211600 | 1.35877300 |
| E(RB3LYP): -1494.60810223 | | | |
| Sum of electronic and thermal free energies: -1494.233948 | | | |
| Number of imaginary frequencies: 0 | | | |

## 3′F-LN **6** Conformer L

| **Atom** | **x** | **y** | **z** |
| --- | --- | --- | --- |
| H | 1.79674400 | 4.06071700 | 1.27995100 |
| O | 2.20624100 | 3.32550200 | 0.81235100 |
| C | 2.76827900 | 2.44762200 | 1.78597000 |
| H | 2.05409900 | 2.27860800 | 2.59892800 |
| H | 3.68781300 | 2.86250500 | 2.21705000 |
| C | 3.09676400 | 1.09170000 | 1.17575600 |
| H | 3.32271200 | 0.42796400 | 2.02359300 |
| O | 1.92812700 | 0.61943200 | 0.50264800 |
| C | 1.97400800 | -0.74671700 | 0.08039300 |
| H | 2.08099500 | -1.40392000 | 0.95461400 |
| O | 0.78774900 | -0.99704300 | -0.59426800 |
| C | -0.42647200 | -0.84895300 | 0.17064400 |
| H | -0.19591600 | -0.58543600 | 1.20734500 |
| C | -1.19819400 | 0.31459500 | -0.47391700 |
| H | -1.14563800 | 0.15469700 | -1.56182300 |
| O | -0.62929000 | 1.57525300 | -0.14511800 |
| H | 0.33335700 | 1.47507200 | -0.03975100 |
| C | -2.67372000 | 0.34916200 | -0.06143600 |
| H | -2.75390800 | 0.62386800 | 0.99071200 |
| N | -3.39481400 | 1.34651200 | -0.82998600 |
| H | -3.33992400 | 1.29325000 | -1.83674500 |
| C | -4.11119600 | 2.35950500 | -0.27980900 |
| O | -4.21808100 | 2.52298900 | 0.93650900 |
| C | -4.78082700 | 3.30028200 | -1.26052000 |
| H | -4.63197000 | 3.02417300 | -2.30520400 |
| H | -5.85097400 | 3.32122300 | -1.04508800 |
| H | -4.38921400 | 4.30679500 | -1.09804000 |
| C | -3.28693800 | -1.04543900 | -0.22557100 |
| H | -3.24811500 | -1.37470400 | -1.27869800 |
| O | -4.59312400 | -1.02317600 | 0.23483800 |
| C | -5.34932700 | -2.19640400 | -0.09176800 |
| H | -4.91711900 | -3.08441300 | 0.37617400 |
| H | -5.39105900 | -2.33758500 | -1.17785500 |
| H | -6.35532400 | -2.03280600 | 0.29160700 |
| O | -2.54857200 | -1.96830300 | 0.57166600 |
| C | -1.20282500 | -2.17660900 | 0.14022400 |
| H | -1.18203100 | -2.57049900 | -0.88512200 |
| C | -0.67151400 | -3.22446200 | 1.11515300 |
| H | -1.32867600 | -4.09947600 | 1.05986300 |
| C | 3.14032100 | -0.93066700 | -0.89585200 |
| H | 2.92523900 | -0.32012000 | -1.77913800 |
| C | 4.42115000 | -0.42405700 | -0.24097300 |
| H | 4.71704800 | -1.08630700 | 0.57615400 |
| C | 4.31112800 | 1.02246200 | 0.24123500 |
| H | 5.20803000 | 1.27456900 | 0.82090000 |
| O | 4.15689000 | 1.91222500 | -0.85612600 |
| H | 4.90741100 | 1.78024400 | -1.44807500 |
| O | 3.25029200 | -2.30575500 | -1.23413100 |
| H | 3.95532700 | -2.39544800 | -1.88612100 |
| F | 5.45584000 | -0.50140800 | -1.21003200 |
| O | 0.67082100 | -3.56380400 | 0.77573600 |
| H | 0.98039800 | -4.22396300 | 1.40468800 |
| H | -0.73041500 | -2.81260200 | 2.13026500 |
| E(RB3LYP): -1494.59399566 | | | |
| Sum of electronic and thermal free energies: -1494.221936 | | | |
| Number of imaginary frequencies: 0 | | | |

## 3′F-LN **6** O2′-H…O6 Hydrogen Bond

| **Atom** | **x** | **y** | **z** |
| --- | --- | --- | --- |
| H | 1.52854800 | 3.58423500 | -0.92487700 |
| O | 2.17044900 | 3.83285700 | -0.25167600 |
| C | 3.34195000 | 3.03505400 | -0.42689500 |
| H | 4.16819900 | 3.57395000 | 0.04103500 |
| H | 3.57233800 | 2.89825100 | -1.48594800 |
| C | 3.20552400 | 1.67549000 | 0.24518300 |
| H | 2.94693800 | 1.82671300 | 1.30189300 |
| O | 2.12686800 | 0.98507600 | -0.41026900 |
| C | 1.83504100 | -0.29024200 | 0.15763200 |
| H | 1.48326100 | -0.16090600 | 1.19091000 |
| O | 0.84479400 | -0.87329200 | -0.63352200 |
| C | -0.48929400 | -0.86163700 | -0.12048600 |
| H | -0.46189400 | -1.02378400 | 0.96149700 |
| C | -1.21867000 | 0.45108900 | -0.41067600 |
| H | -1.20097500 | 0.61916400 | -1.49705600 |
| O | -0.62453900 | 1.55486400 | 0.26176200 |
| H | 0.28211900 | 1.65994100 | -0.06911200 |
| C | -2.67338100 | 0.34177700 | 0.07213300 |
| H | -2.67839400 | 0.30560700 | 1.16248400 |
| N | -3.44385600 | 1.50366600 | -0.32499800 |
| H | -3.48376800 | 1.72771600 | -1.30871000 |
| C | -4.08482400 | 2.32369900 | 0.54802700 |
| O | -4.07420400 | 2.14460500 | 1.76610100 |
| C | -4.82609900 | 3.49094300 | -0.06977900 |
| H | -4.74277400 | 3.53735100 | -1.15625400 |
| H | -5.88038500 | 3.41612600 | 0.20481900 |
| H | -4.43554300 | 4.41597700 | 0.35918800 |
| C | -3.33402100 | -0.95224000 | -0.44036700 |
| H | -3.46705700 | -0.90634000 | -1.53529700 |
| O | -4.55308600 | -1.10140000 | 0.19766000 |
| C | -5.40576400 | -2.09485300 | -0.38762100 |
| H | -4.95253000 | -3.08711500 | -0.32364300 |
| H | -5.61475800 | -1.85321700 | -1.43585400 |
| H | -6.33425100 | -2.07834900 | 0.18088700 |
| O | -2.52447700 | -2.09315200 | -0.13653700 |
| C | -1.23950500 | -2.02949200 | -0.76125800 |
| H | -1.36271700 | -1.85028900 | -1.83933200 |
| C | -0.52384700 | -3.36629900 | -0.60532300 |
| H | 0.27179200 | -3.42384500 | -1.34696900 |
| C | 3.04929800 | -1.22900200 | 0.14949800 |
| H | 3.28229400 | -1.46845200 | -0.89436000 |
| C | 4.23805600 | -0.52731800 | 0.78068100 |
| H | 4.09644600 | -0.44824100 | 1.86137700 |
| C | 4.49411600 | 0.85035000 | 0.17321500 |
| H | 5.26557500 | 1.35771300 | 0.76509200 |
| O | 4.89641700 | 0.74765700 | -1.18957100 |
| H | 5.68951100 | 0.19976500 | -1.22699900 |
| O | 2.76860400 | -2.39570600 | 0.90584000 |
| H | 1.87937400 | -2.73439800 | 0.69405400 |
| F | 5.39890700 | -1.31479200 | 0.58276600 |
| O | 0.12281200 | -3.54253100 | 0.66024900 |
| H | -0.53626500 | -3.58354100 | 1.36274600 |
| H | -1.23883200 | -4.17230700 | -0.79558600 |
| E(RB3LYP): -1494.60999019 | | | |
| Sum of electronic and thermal free energies: -1494.234984 | | | |
| Number of imaginary frequencies: 0 | | | |

## 4′F-LN **7** Conformer A

| **Atom** | **x** | **y** | **z** |
| --- | --- | --- | --- |
| H | 4.18282800 | 4.36393900 | 1.18184500 |
| O | 4.16612200 | 3.40744900 | 1.29137600 |
| C | 3.07685400 | 2.88129900 | 0.53382700 |
| H | 3.14552900 | 3.16792800 | -0.52002600 |
| H | 2.11323100 | 3.21765800 | 0.93098600 |
| C | 3.14455700 | 1.36511800 | 0.64952700 |
| H | 3.10137400 | 1.08522800 | 1.71099100 |
| O | 2.00020900 | 0.85428200 | -0.04328600 |
| C | 1.84629800 | -0.57160800 | 0.03651100 |
| H | 1.74851300 | -0.86793900 | 1.09099400 |
| O | 0.71120800 | -0.90273400 | -0.68357800 |
| C | -0.55050800 | -0.78319400 | -0.00348900 |
| H | -0.38831200 | -0.66351000 | 1.07086000 |
| C | -1.30930000 | 0.43161200 | -0.53996000 |
| H | -1.30269100 | 0.36332500 | -1.63860800 |
| O | -0.71657100 | 1.65546900 | -0.12604000 |
| H | 0.24901700 | 1.56978200 | -0.20481300 |
| C | -2.76238900 | 0.42861300 | -0.04597300 |
| H | -2.77398300 | 0.61240200 | 1.02900400 |
| N | -3.52259300 | 1.49014100 | -0.67763100 |
| H | -3.54098500 | 1.51608100 | -1.68657300 |
| C | -4.19100900 | 2.45712400 | 0.00155800 |
| O | -4.21213500 | 2.52127100 | 1.23137900 |
| C | -4.92214100 | 3.47374000 | -0.85113500 |
| H | -4.81171200 | 3.30793000 | -1.92353000 |
| H | -5.98274200 | 3.44360100 | -0.59267100 |
| H | -4.54757400 | 4.46881500 | -0.60252000 |
| C | -3.41133900 | -0.94277700 | -0.29118400 |
| H | -3.48623700 | -1.14932300 | -1.37381600 |
| O | -4.66641900 | -0.95015000 | 0.29593200 |
| C | -5.48231000 | -2.06703200 | -0.08015900 |
| H | -5.03347300 | -3.00821600 | 0.24690900 |
| H | -5.62886100 | -2.08856700 | -1.16615700 |
| H | -6.44329000 | -1.92810700 | 0.41281200 |
| O | -2.62621400 | -1.96757900 | 0.31613300 |
| C | -1.32091100 | -2.08466000 | -0.25169000 |
| H | -1.39713900 | -2.24940900 | -1.33686500 |
| C | -0.62640400 | -3.29498800 | 0.34386900 |
| H | 0.31461200 | -3.44486100 | -0.19363700 |
| C | 3.05608500 | -1.25526100 | -0.59514900 |
| H | 3.07906800 | -0.99217600 | -1.65918200 |
| C | 4.33857200 | -0.75815000 | 0.07627900 |
| H | 4.31720200 | -1.07253600 | 1.12764300 |
| C | 4.41772800 | 0.76631800 | 0.05699500 |
| H | 5.29403000 | 1.12620900 | 0.59867500 |
| O | 2.90275100 | -2.65707600 | -0.41886700 |
| H | 3.73437300 | -3.07024700 | -0.68413800 |
| O | -0.37906200 | -3.06525200 | 1.73613700 |
| H | 0.17662000 | -3.77923600 | 2.06504000 |
| H | -1.26890300 | -4.17089200 | 0.20027600 |
| F | 4.55201900 | 1.20364500 | -1.27921700 |
| O | 5.42235600 | -1.38445400 | -0.60340200 |
| H | 6.19882500 | -1.38839600 | -0.03235900 |
| E(RB3LYP): -1494.60498374 | | | |
| Sum of electronic and thermal free energies: -1494.233229 | | | |
| Number of imaginary frequencies: 0 | | | |

## 4′F-LN **7** Conformer B

| **Atom** | **x** | **y** | **z** |
| --- | --- | --- | --- |
| H | 4.17720000 | 3.65746100 | 1.74477800 |
| O | 4.32540900 | 3.58089700 | 0.79527000 |
| C | 3.21614900 | 2.90995100 | 0.20565200 |
| H | 3.30109600 | 3.04412400 | -0.87280600 |
| H | 2.26296500 | 3.33471900 | 0.53586000 |
| C | 3.22992100 | 1.41626400 | 0.53245800 |
| H | 3.17777300 | 1.28390700 | 1.62252100 |
| O | 2.06541100 | 0.85204500 | -0.08479000 |
| C | 1.86435100 | -0.54458100 | 0.18812000 |
| H | 1.75255700 | -0.68786400 | 1.27266700 |
| O | 0.72337500 | -0.94113300 | -0.48998400 |
| C | -0.54519100 | -0.75828800 | 0.16207400 |
| H | -0.39950400 | -0.63795900 | 1.24164500 |
| C | -1.23657700 | 0.49354900 | -0.39327400 |
| H | -1.19026100 | 0.42372300 | -1.49073500 |
| O | -0.60743200 | 1.68796300 | 0.04710400 |
| H | 0.35074500 | 1.59829100 | -0.09721900 |
| C | -2.70681800 | 0.56077200 | 0.04147900 |
| H | -2.75488500 | 0.74364900 | 1.11561100 |
| N | -3.38933500 | 1.65766900 | -0.61731600 |
| H | -3.37962100 | 1.67731400 | -1.62653900 |
| C | -4.02632200 | 2.66065700 | 0.03950400 |
| O | -4.07761700 | 2.73136100 | 1.26809300 |
| C | -4.68124800 | 3.70959100 | -0.83551000 |
| H | -4.55617100 | 3.53107600 | -1.90421200 |
| H | -5.74737900 | 3.73916300 | -0.60096300 |
| H | -4.25843900 | 4.68439800 | -0.58383500 |
| C | -3.40646900 | -0.77768300 | -0.23402500 |
| H | -3.44247900 | -0.98363900 | -1.31862400 |
| O | -4.68463900 | -0.72901600 | 0.29894600 |
| C | -5.52980400 | -1.81308700 | -0.10790000 |
| H | -5.13282900 | -2.77058700 | 0.23811200 |
| H | -5.63383500 | -1.83190500 | -1.19881500 |
| H | -6.50311100 | -1.63372000 | 0.34628900 |
| O | -2.69175300 | -1.82982400 | 0.40973900 |
| C | -1.37505200 | -2.02120900 | -0.10811600 |
| H | -1.41761500 | -2.19229000 | -1.19344200 |
| C | -0.77588500 | -3.24381800 | 0.55732000 |
| H | -0.91096100 | -3.15628300 | 1.64256300 |
| C | 3.05492400 | -1.34740100 | -0.33188400 |
| H | 3.08729100 | -1.23948800 | -1.42235400 |
| C | 4.35356000 | -0.80247200 | 0.26767600 |
| H | 4.32516600 | -0.96318600 | 1.35322200 |
| C | 4.48042500 | 0.70003500 | 0.03166800 |
| H | 5.37003900 | 1.10590700 | 0.51669000 |
| O | 2.85529800 | -2.70403400 | 0.04104100 |
| H | 3.67262100 | -3.17827200 | -0.15850800 |
| O | -1.44250800 | -4.40612500 | 0.05138900 |
| H | -1.05095400 | -5.18275400 | 0.46485400 |
| H | 0.29423500 | -3.27660000 | 0.33321100 |
| F | 4.62242900 | 0.93925600 | -1.35301500 |
| O | 5.41494100 | -1.55380300 | -0.31314600 |
| H | 6.19017800 | -1.50650100 | 0.25762200 |
| E(RB3LYP): -1494.60463970 | | | |
| Sum of electronic and thermal free energies: -1494.232689 | | | |
| Number of imaginary frequencies: 0 | | | |

## 4′F-LN **7** Conformer C

| **Atom** | **x** | **y** | **z** |
| --- | --- | --- | --- |
| H | -0.96309300 | -2.73772400 | 1.35304300 |
| O | -1.71793700 | -3.14863900 | 1.79463600 |
| C | -2.89066300 | -2.88642400 | 1.02614200 |
| H | -3.72324400 | -3.35762000 | 1.55063200 |
| H | -2.81495200 | -3.32366600 | 0.02513500 |
| C | -3.14989100 | -1.39183100 | 0.90563100 |
| H | -3.17237400 | -0.95425900 | 1.91311600 |
| O | -2.04256400 | -0.85205600 | 0.17144900 |
| C | -2.01860000 | 0.57358300 | 0.08631900 |
| H | -1.99684500 | 0.99768000 | 1.10175500 |
| O | -0.88079000 | 0.92247500 | -0.62964700 |
| C | 0.37387700 | 0.76143600 | 0.05976900 |
| H | 0.19789500 | 0.49207400 | 1.10655200 |
| C | 1.16289600 | -0.36394100 | -0.62015300 |
| H | 1.17496200 | -0.14176000 | -1.69793800 |
| O | 0.56993400 | -1.63858700 | -0.40205300 |
| H | -0.38534700 | -1.54094700 | -0.54128700 |
| C | 2.60587900 | -0.42446300 | -0.10421600 |
| H | 2.60204600 | -0.75282300 | 0.93591000 |
| N | 3.38359200 | -1.38666200 | -0.86082200 |
| H | 3.42743900 | -1.27183600 | -1.86290100 |
| C | 4.03305600 | -2.44246100 | -0.30600200 |
| O | 4.01898800 | -2.67790100 | 0.90266800 |
| C | 4.78615900 | -3.33329300 | -1.27226100 |
| H | 4.73475400 | -2.99855500 | -2.30906300 |
| H | 5.83198300 | -3.37501500 | -0.96136800 |
| H | 4.37795600 | -4.34380900 | -1.20238400 |
| C | 3.24616500 | 0.97158600 | -0.14955300 |
| H | 3.34461000 | 1.32253400 | -1.19173100 |
| O | 4.48525500 | 0.91041600 | 0.46679300 |
| C | 5.29956700 | 2.07229300 | 0.26027800 |
| H | 4.82992500 | 2.96115000 | 0.68863500 |
| H | 5.47884300 | 2.23132200 | -0.80915700 |
| H | 6.24643100 | 1.87944500 | 0.76223000 |
| O | 2.43307800 | 1.89710900 | 0.56957000 |
| C | 1.14160700 | 2.08828900 | -0.00937500 |
| H | 1.23954800 | 2.39144400 | -1.06175500 |
| C | 0.43785200 | 3.19330300 | 0.75200600 |
| H | 0.50072300 | 2.98159300 | 1.82641100 |
| C | -3.25727000 | 1.05185800 | -0.66519900 |
| H | -3.19753600 | 0.67066100 | -1.69145100 |
| C | -4.51894500 | 0.49473400 | 0.00671600 |
| H | -4.59596700 | 0.94147000 | 1.00596500 |
| C | -4.44692000 | -1.02248800 | 0.19401400 |
| H | -5.31571800 | -1.38609300 | 0.74795300 |
| O | -3.26425700 | 2.47182300 | -0.64576900 |
| H | -4.11004200 | 2.75949800 | -1.01259100 |
| O | 1.07297900 | 4.43547900 | 0.43590700 |
| H | 0.61733800 | 5.13833700 | 0.91122800 |
| H | -0.61489500 | 3.20788500 | 0.45285500 |
| F | -4.47411700 | -1.64970400 | -1.07001700 |
| O | -5.61748600 | 0.90316500 | -0.80136100 |
| H | -6.41875200 | 0.92361600 | -0.26613400 |
| E(RB3LYP): -1494.60585116 | | | |
| Sum of electronic and thermal free energies: -1494.232888 | | | |
| Number of imaginary frequencies: 0 | | | |

## 4′F-LN **7** Conformer D

| **Atom** | **x** | **y** | **z** |
| --- | --- | --- | --- |
| H | 0.81299700 | 2.61126700 | 1.38360500 |
| O | 1.53844700 | 3.02064800 | 1.87359500 |
| C | 2.74259000 | 2.83904800 | 1.13060300 |
| H | 3.54414700 | 3.29954300 | 1.71015400 |
| H | 2.68501500 | 3.33533300 | 0.15629900 |
| C | 3.04751000 | 1.36208000 | 0.92640900 |
| H | 3.06414100 | 0.86393200 | 1.90574800 |
| O | 1.97094900 | 0.83987300 | 0.13863100 |
| C | 1.98881700 | -0.57920500 | -0.03572600 |
| H | 1.95511700 | -1.07114300 | 0.94746400 |
| O | 0.86998000 | -0.90445700 | -0.79216400 |
| C | -0.38575000 | -0.81001000 | -0.09341400 |
| H | -0.21237100 | -0.57124900 | 0.95825800 |
| C | -1.22419400 | 0.30095200 | -0.72626400 |
| H | -1.26168700 | 0.11318600 | -1.80997600 |
| O | -0.66804600 | 1.58962200 | -0.48412400 |
| H | 0.29111500 | 1.51906400 | -0.61105900 |
| C | -2.65025600 | 0.28986300 | -0.16230000 |
| H | -2.62199900 | 0.59015900 | 0.88587300 |
| N | -3.49017800 | 1.24070300 | -0.86486700 |
| H | -3.55273300 | 1.15896100 | -1.86918300 |
| C | -4.17052600 | 2.24731900 | -0.25824200 |
| O | -4.13852100 | 2.44067500 | 0.95754400 |
| C | -4.98410700 | 3.13717400 | -1.17523700 |
| H | -4.91881500 | 2.85923100 | -2.22793600 |
| H | -6.02868700 | 3.09721300 | -0.85942700 |
| H | -4.63952800 | 4.16630700 | -1.05483800 |
| C | -3.24108900 | -1.12882800 | -0.22522900 |
| H | -3.36394800 | -1.45211500 | -1.27409500 |
| O | -4.46059600 | -1.12746400 | 0.43204100 |
| C | -5.24584200 | -2.30662600 | 0.21296300 |
| H | -4.73891300 | -3.19334900 | 0.60153600 |
| H | -5.45041700 | -2.43981200 | -0.85548300 |
| H | -6.18355400 | -2.15723300 | 0.74599600 |
| O | -2.37552100 | -2.05086700 | 0.43679800 |
| C | -1.09452500 | -2.16461200 | -0.18667300 |
| H | -1.21541300 | -2.43530700 | -1.24605700 |
| C | -0.31313800 | -3.28084500 | 0.48060300 |
| H | 0.58912800 | -3.46583600 | -0.11099100 |
| C | 3.25263900 | -0.97248000 | -0.79341300 |
| H | 3.20087600 | -0.52590400 | -1.79347800 |
| C | 4.48566800 | -0.42629000 | -0.06149700 |
| H | 4.55638400 | -0.93324500 | 0.90912600 |
| C | 4.36816900 | 1.07348000 | 0.22042700 |
| H | 5.21562700 | 1.42398000 | 0.81446100 |
| O | 3.30145400 | -2.39014100 | -0.86811200 |
| H | 4.16198700 | -2.62791100 | -1.23627800 |
| O | 0.02998600 | -2.89034100 | 1.81487700 |
| H | 0.56053700 | -3.59080000 | 2.20841400 |
| H | -0.93088300 | -4.18541400 | 0.48146500 |
| F | 4.40277800 | 1.78123400 | -1.00069400 |
| O | 5.61062100 | -0.75335000 | -0.87096900 |
| H | 6.40231700 | -0.78460100 | -0.32228900 |
| E(RB3LYP): -1494.60714895 | | | |
| Sum of electronic and thermal free energies: -1494.234689 | | | |
| Number of imaginary frequencies: 0 | | | |

## 4′F-LN **7** Conformer E

| **Atom** | **x** | **y** | **z** |
| --- | --- | --- | --- |
| H | 2.61419900 | 4.43974100 | 0.05522000 |
| O | 2.89230500 | 3.53529600 | -0.12364100 |
| C | 2.97419600 | 2.84764400 | 1.12251100 |
| H | 2.06330200 | 3.00516000 | 1.71077500 |
| H | 3.82897000 | 3.19724900 | 1.71499400 |
| C | 3.13298700 | 1.35056500 | 0.91546000 |
| H | 3.12591700 | 0.90858700 | 1.92378300 |
| O | 2.00909200 | 0.86632100 | 0.17155000 |
| C | 1.91181700 | -0.56298100 | 0.11094300 |
| H | 1.80926500 | -0.96513800 | 1.12965100 |
| O | 0.79949600 | -0.86509500 | -0.65796700 |
| C | -0.47741400 | -0.80394800 | 0.00104300 |
| H | -0.33965900 | -0.73564100 | 1.08335600 |
| C | -1.24890700 | 0.42260300 | -0.48999200 |
| H | -1.21561900 | 0.41039500 | -1.59028400 |
| O | -0.69352600 | 1.63609400 | -0.00118400 |
| H | 0.27664100 | 1.56862500 | -0.03927000 |
| C | -2.71306100 | 0.36492900 | -0.03244200 |
| H | -2.75351300 | 0.49292600 | 1.04986800 |
| N | -3.47971200 | 1.44318200 | -0.62722400 |
| H | -3.46713900 | 1.52755300 | -1.63306000 |
| C | -4.18666800 | 2.35826000 | 0.08387900 |
| O | -4.24538400 | 2.35325900 | 1.31419100 |
| C | -4.91245300 | 3.40718800 | -0.73350000 |
| H | -4.76761700 | 3.30240800 | -1.80949600 |
| H | -5.97933900 | 3.34408800 | -0.50910700 |
| H | -4.56453500 | 4.39356900 | -0.41996600 |
| C | -3.32801300 | -1.00448200 | -0.36148600 |
| H | -3.37360400 | -1.15875700 | -1.45445500 |
| O | -4.59585400 | -1.06493500 | 0.19460300 |
| C | -5.37968600 | -2.17855100 | -0.25244700 |
| H | -4.91956700 | -3.12521700 | 0.04151200 |
| H | -5.50056000 | -2.15219200 | -1.34149300 |
| H | -6.35451100 | -2.08141800 | 0.22307400 |
| O | -2.53621100 | -2.04272800 | 0.21362200 |
| C | -1.21649300 | -2.10562900 | -0.32859000 |
| H | -1.26515500 | -2.21765600 | -1.42202500 |
| C | -0.51093900 | -3.33095700 | 0.22182500 |
| H | 0.44205500 | -3.43927900 | -0.30473500 |
| C | 3.15342100 | -1.14183400 | -0.56260700 |
| H | 3.16950400 | -0.79341400 | -1.60190400 |
| C | 4.41511700 | -0.64845000 | 0.15099500 |
| H | 4.41408300 | -1.05075600 | 1.17192400 |
| C | 4.42748200 | 0.87852700 | 0.25135200 |
| H | 5.29439800 | 1.22404000 | 0.82017300 |
| O | 3.05821200 | -2.55876100 | -0.50082400 |
| H | 3.90520300 | -2.91435600 | -0.79879900 |
| O | -0.29316500 | -3.16403600 | 1.62785900 |
| H | 0.25473200 | -3.89286200 | 1.93667900 |
| H | -1.13463400 | -4.20986200 | 0.02418900 |
| F | 4.54612600 | 1.40949900 | -1.04603500 |
| O | 5.52184300 | -1.16858200 | -0.57940900 |
| H | 6.29726200 | -1.19750900 | -0.00777000 |
| E(RB3LYP): -1494.60150265 | | | |
| Sum of electronic and thermal free energies: -1494.229994 | | | |
| Number of imaginary frequencies: 0 | | | |

## 4′F-LN **7** Conformer F

| **Atom** | **x** | **y** | **z** |
| --- | --- | --- | --- |
| H | -2.80690700 | -4.39981600 | -0.45564200 |
| O | -3.04953700 | -3.46959300 | -0.51141100 |
| C | -3.10612500 | -2.95171400 | 0.81556000 |
| H | -2.19984100 | -3.21479600 | 1.37256800 |
| H | -3.96986300 | -3.35015100 | 1.36244000 |
| C | -3.21798300 | -1.43608300 | 0.81039500 |
| H | -3.20276600 | -1.13180100 | 1.86800700 |
| O | -2.07396600 | -0.89196000 | 0.13908200 |
| C | -1.93197000 | 0.52905600 | 0.27044300 |
| H | -1.82385100 | 0.78529000 | 1.33501900 |
| O | -0.81006700 | 0.90320900 | -0.45221600 |
| C | 0.47218100 | 0.77768300 | 0.18659300 |
| H | 0.34641100 | 0.69619100 | 1.27234800 |
| C | 1.18453000 | -0.47852400 | -0.33286900 |
| H | 1.10935600 | -0.45427200 | -1.43079000 |
| O | 0.60076300 | -1.67085000 | 0.17025600 |
| H | -0.36559500 | -1.60369100 | 0.07073500 |
| C | 2.66699800 | -0.48925100 | 0.06431800 |
| H | 2.74847700 | -0.62631500 | 1.14316200 |
| N | 3.35956200 | -1.59538700 | -0.56847300 |
| H | 3.30684900 | -1.66800300 | -1.57393800 |
| C | 4.05422200 | -2.54523900 | 0.10839100 |
| O | 4.15954200 | -2.55303900 | 1.33560700 |
| C | 4.70418200 | -3.61677300 | -0.74276300 |
| H | 4.52507800 | -3.49703300 | -1.81200500 |
| H | 5.78021600 | -3.60092700 | -0.55717900 |
| H | 4.32610800 | -4.59016300 | -0.42356800 |
| C | 3.32212600 | 0.85457100 | -0.28448800 |
| H | 3.31941600 | 1.01850300 | -1.37678900 |
| O | 4.61662100 | 0.86102100 | 0.20982600 |
| C | 5.41968900 | 1.94854500 | -0.26694000 |
| H | 5.00596200 | 2.90933400 | 0.04912600 |
| H | 5.49312800 | 1.92274700 | -1.36018600 |
| H | 6.40976300 | 1.81507000 | 0.16634700 |
| O | 2.59800100 | 1.91177000 | 0.34051700 |
| C | 1.26386900 | 2.04993600 | -0.14803300 |
| H | 1.27369300 | 2.18358600 | -1.23941900 |
| C | 0.64866000 | 3.27903900 | 0.49027300 |
| H | 0.81164200 | 3.23271500 | 1.57421500 |
| C | -3.15305500 | 1.23021700 | -0.32066600 |
| H | -3.17281000 | 1.02463400 | -1.39726400 |
| C | -4.43370200 | 0.68527400 | 0.31685200 |
| H | -4.42758500 | 0.94694500 | 1.38240300 |
| C | -4.49269600 | -0.84002300 | 0.21162900 |
| H | -5.37396100 | -1.23112700 | 0.72630000 |
| O | -3.01434200 | 2.62224400 | -0.06929200 |
| H | -3.84739300 | 3.04192700 | -0.31936300 |
| O | 1.27022700 | 4.44060300 | -0.07094000 |
| H | 0.86590500 | 5.22015300 | 0.32441000 |
| H | -0.42707000 | 3.27568500 | 0.29097200 |
| F | -4.61764000 | -1.18864100 | -1.14495400 |
| O | -5.51819000 | 1.33206000 | -0.34149600 |
| H | -6.30050400 | 1.29680500 | 0.22035000 |
| E(RB3LYP): -1494.60061059 | | | |
| Sum of electronic and thermal free energies: -1494.229250 | | | |
| Number of imaginary frequencies: 0 | | | |

## 4′F-LN **7** Conformer G

| **Atom** | **x** | **y** | **z** |
| --- | --- | --- | --- |
| H | -4.52551200 | 3.91079400 | -1.82605800 |
| O | -4.45671500 | 2.95313200 | -1.75313200 |
| C | -3.27037400 | 2.63575900 | -1.02541500 |
| H | -3.25809100 | 3.12359000 | -0.04599200 |
| H | -2.37124900 | 2.92721000 | -1.57861300 |
| C | -3.25349000 | 1.12539700 | -0.83754600 |
| H | -3.29557700 | 0.64330200 | -1.82392700 |
| O | -2.01845800 | 0.81092200 | -0.18567800 |
| C | -1.78454500 | -0.59515000 | -0.00563500 |
| H | -1.77193200 | -1.08978100 | -0.98782200 |
| O | -0.56719000 | -0.72962100 | 0.63832700 |
| C | 0.61280300 | -0.68233300 | -0.18460800 |
| H | 0.33608800 | -0.76452300 | -1.23915700 |
| C | 1.34478000 | 0.63965100 | 0.04807400 |
| H | 1.44689400 | 0.78306900 | 1.12937400 |
| O | 0.64617700 | 1.73920000 | -0.52981100 |
| H | -0.30048900 | 1.63162900 | -0.33663700 |
| C | 2.74475600 | 0.60160100 | -0.58493900 |
| H | 2.62292700 | 0.54899700 | -1.66901200 |
| N | 3.50526600 | 1.81684500 | -0.31357400 |
| H | 3.73158300 | 2.39742300 | -1.10478100 |
| C | 3.90382700 | 2.22871600 | 0.91467800 |
| O | 3.67418500 | 1.58310300 | 1.94019400 |
| C | 4.65745400 | 3.54204500 | 0.96344800 |
| H | 4.80730400 | 4.00014700 | -0.01480800 |
| H | 4.10284200 | 4.23442100 | 1.60047500 |
| H | 5.62914900 | 3.36693400 | 1.42966800 |
| C | 3.49555600 | -0.67449300 | -0.17005600 |
| H | 3.67750900 | -0.68736600 | 0.91335500 |
| O | 4.68845800 | -0.73111100 | -0.87592400 |
| C | 5.60391600 | -1.72226700 | -0.39323100 |
| H | 5.18777400 | -2.72700800 | -0.50240800 |
| H | 5.84928600 | -1.53995900 | 0.65922200 |
| H | 6.50594000 | -1.63208400 | -0.99680700 |
| O | 2.71075300 | -1.81538100 | -0.52470900 |
| C | 1.48084200 | -1.88609900 | 0.19797100 |
| H | 1.67880200 | -1.85176300 | 1.27955100 |
| C | 0.80272700 | -3.21096500 | -0.09637800 |
| H | -0.06579100 | -3.30167800 | 0.56296800 |
| C | -2.88278600 | -1.19101100 | 0.87124600 |
| H | -2.81850000 | -0.72519400 | 1.86158100 |
| C | -4.25295900 | -0.89018400 | 0.25906000 |
| H | -4.31591400 | -1.40590900 | -0.70769300 |
| C | -4.42387700 | 0.60317600 | -0.00780800 |
| H | -5.36931000 | 0.81213100 | -0.51138400 |
| O | -2.66114900 | -2.59275100 | 0.95104300 |
| H | -3.43549900 | -2.97956700 | 1.37931400 |
| O | 0.39640900 | -3.24456500 | -1.47009000 |
| H | -0.12907100 | -4.03904000 | -1.60918300 |
| H | 1.50895500 | -4.01976800 | 0.12207300 |
| O | -5.22457600 | -1.41325300 | 1.16006600 |
| H | -6.04835200 | -1.57109500 | 0.68545500 |
| F | -4.45461900 | 1.29074500 | 1.22564500 |
| E(RB3LYP): -1494.60354768 | | | |
| Sum of electronic and thermal free energies: -1494.231401 | | | |
| Number of imaginary frequencies: 0 | | | |

## 4′F-LN **7** Conformer H

| **Atom** | **x** | **y** | **z** |
| --- | --- | --- | --- |
| H | -5.92876000 | 2.45843000 | -1.86191900 |
| O | -5.31366000 | 1.71858400 | -1.89658900 |
| C | -4.39948300 | 1.83346500 | -0.80522100 |
| H | -4.92519100 | 1.89647600 | 0.15243400 |
| H | -3.75289500 | 2.71036800 | -0.91347200 |
| C | -3.52574700 | 0.58715000 | -0.82061400 |
| H | -3.03262900 | 0.51308600 | -1.80067400 |
| O | -2.55276300 | 0.76621900 | 0.21152700 |
| C | -1.59376900 | -0.28174700 | 0.27461000 |
| H | -1.06113000 | -0.34432700 | -0.68384400 |
| O | -0.73405500 | 0.00461700 | 1.33372900 |
| C | 0.60822400 | 0.42687800 | 1.04507400 |
| H | 1.04351900 | 0.59122600 | 2.03074800 |
| C | 1.40380400 | -0.68361200 | 0.34429600 |
| H | 0.94257400 | -0.89821000 | -0.63030800 |
| O | 1.42907900 | -1.86605300 | 1.12554200 |
| H | 0.53285100 | -2.24435000 | 1.09377200 |
| C | 2.84287900 | -0.21938600 | 0.08909500 |
| H | 3.35838300 | -0.09238700 | 1.04189500 |
| N | 3.58048200 | -1.20395700 | -0.67777300 |
| H | 3.19671900 | -1.49263000 | -1.56584600 |
| C | 4.73788600 | -1.77910700 | -0.26187600 |
| O | 5.27160900 | -1.50942300 | 0.81488000 |
| C | 5.34893400 | -2.78905400 | -1.21120200 |
| H | 4.76722500 | -2.94435200 | -2.12071900 |
| H | 6.35026200 | -2.44757100 | -1.48209000 |
| H | 5.45304000 | -3.74049800 | -0.68575500 |
| C | 2.82882000 | 1.14281800 | -0.62527700 |
| H | 2.37524900 | 1.04620600 | -1.62809200 |
| O | 4.13113900 | 1.60630300 | -0.71945700 |
| C | 4.29292300 | 2.72828700 | -1.59682900 |
| H | 3.73226100 | 3.59359400 | -1.23482200 |
| H | 3.96240600 | 2.47512200 | -2.61068100 |
| H | 5.35687800 | 2.95981300 | -1.60960800 |
| O | 2.07245100 | 2.08930300 | 0.12755200 |
| C | 0.68861600 | 1.74862000 | 0.26523400 |
| H | 0.23892600 | 1.63223900 | -0.73210800 |
| C | -0.02548200 | 2.90002800 | 0.94739700 |
| H | -1.09802200 | 2.68807800 | 0.93274500 |
| C | -2.28230600 | -1.61823600 | 0.55618400 |
| H | -2.74289200 | -1.56379300 | 1.54828800 |
| C | -3.35481600 | -1.89011500 | -0.49492000 |
| H | -2.86194100 | -2.00096400 | -1.46930400 |
| C | -4.31184800 | -0.70471100 | -0.59929700 |
| H | -5.03884300 | -0.84890800 | -1.40024000 |
| O | -1.27055600 | -2.62514200 | 0.52398300 |
| H | -1.69944200 | -3.48654000 | 0.60433700 |
| O | 0.45510100 | 3.03499100 | 2.28906600 |
| H | -0.07203800 | 3.70840700 | 2.73093900 |
| H | 0.16675700 | 3.81231600 | 0.37101500 |
| O | -3.99634900 | -3.10660800 | -0.12518500 |
| H | -4.42932400 | -3.48784000 | -0.89741400 |
| F | -5.04278700 | -0.59725500 | 0.60498000 |
| E(RB3LYP): -1494.60187679 | | | |
| Sum of electronic and thermal free energies: -1494.229124 | | | |
| Number of imaginary frequencies: 0 | | | |

## 4′F-LN **7** Conformer I

| **Atom** | **x** | **y** | **z** |
| --- | --- | --- | --- |
| H | -6.13972800 | 2.30804500 | 1.20979200 |
| O | -5.80325100 | 1.56239700 | 0.70175800 |
| C | -4.40126400 | 1.73916400 | 0.50613400 |
| H | -3.87938800 | 1.86504100 | 1.46029300 |
| H | -4.18873300 | 2.60304400 | -0.13204900 |
| C | -3.89253000 | 0.48552000 | -0.19224800 |
| H | -4.45551600 | 0.34649700 | -1.12479400 |
| O | -2.50858400 | 0.71497000 | -0.49263300 |
| C | -1.89652700 | -0.33806400 | -1.24562200 |
| H | -2.44260700 | -0.48147600 | -2.18484200 |
| O | -0.62030000 | 0.05504900 | -1.63701700 |
| C | 0.37424000 | 0.37202900 | -0.63941800 |
| H | -0.03326800 | 0.27331100 | 0.36904100 |
| C | 1.55013700 | -0.59675900 | -0.81349200 |
| H | 1.82912700 | -0.57866800 | -1.87802300 |
| O | 1.22325300 | -1.91877100 | -0.41768400 |
| H | 0.45764500 | -2.23278700 | -0.93002000 |
| C | 2.75618000 | -0.15903100 | 0.03044400 |
| H | 2.51718400 | -0.29155100 | 1.08643900 |
| N | 3.91488000 | -0.98199800 | -0.25825200 |
| H | 4.22963900 | -1.03049200 | -1.21620000 |
| C | 4.58279400 | -1.70862300 | 0.67378800 |
| O | 4.26191500 | -1.73252900 | 1.86270400 |
| C | 5.76612900 | -2.50478500 | 0.16305900 |
| H | 5.94428500 | -2.38889300 | -0.90676900 |
| H | 6.65732500 | -2.18974300 | 0.70973800 |
| H | 5.59590100 | -3.56040800 | 0.38476300 |
| C | 3.06441900 | 1.32802600 | -0.19279800 |
| H | 3.39889200 | 1.50514500 | -1.23064800 |
| O | 4.04267700 | 1.71673700 | 0.70927400 |
| C | 4.61162900 | 3.00383900 | 0.43749400 |
| H | 3.85612700 | 3.79007400 | 0.51057000 |
| H | 5.06425000 | 3.02199500 | -0.56055900 |
| H | 5.38327900 | 3.16598100 | 1.18855800 |
| O | 1.89571800 | 2.10655800 | 0.04855300 |
| C | 0.83055800 | 1.82215100 | -0.86073300 |
| H | 1.18611500 | 1.92365600 | -1.89754300 |
| C | -0.26591200 | 2.85993800 | -0.64877400 |
| H | -0.99444500 | 2.76579000 | -1.45956500 |
| C | -1.94940100 | -1.63246700 | -0.42311200 |
| H | -1.38216700 | -1.50156300 | 0.50424400 |
| C | -3.39834800 | -1.96163100 | -0.06967000 |
| H | -3.95429700 | -2.13260700 | -1.00006800 |
| C | -4.04934000 | -0.78059100 | 0.64540200 |
| H | -5.10336100 | -0.97047600 | 0.85320200 |
| O | -1.36712000 | -2.66641900 | -1.21617100 |
| H | -1.58189600 | -3.50988400 | -0.79396000 |
| O | -0.90768700 | 2.75987100 | 0.61904500 |
| H | -1.55165400 | 2.04102400 | 0.53829600 |
| H | 0.19102000 | 3.84987100 | -0.70511700 |
| O | -3.36586400 | -3.15043800 | 0.71244100 |
| H | -4.22354700 | -3.58815700 | 0.66741000 |
| F | -3.40966500 | -0.58671800 | 1.88896600 |
| E(RB3LYP): -1494.60850030 | | | |
| Sum of electronic and thermal free energies: -1494.233081 | | | |
| Number of imaginary frequencies: 0 | | | |

## 4′F-LN **7** Conformer J

| **Atom** | **x** | **y** | **z** |
| --- | --- | --- | --- |
| H | 1.15234400 | 3.62032100 | -0.47151500 |
| O | 1.83408200 | 3.83195600 | 0.17467300 |
| C | 3.03795300 | 3.15581700 | -0.18349800 |
| H | 3.86546400 | 3.72547400 | 0.24420500 |
| H | 3.16562200 | 3.12014100 | -1.26833300 |
| C | 3.08170900 | 1.74145400 | 0.38166800 |
| H | 2.91511100 | 1.78472500 | 1.46651800 |
| O | 2.01524500 | 1.00054900 | -0.23900900 |
| C | 1.87948500 | -0.34716700 | 0.23260200 |
| H | 1.67261800 | -0.33683800 | 1.31242400 |
| O | 0.84200100 | -0.94484600 | -0.46930900 |
| C | -0.47475800 | -0.91071200 | 0.10562600 |
| H | -0.39879500 | -0.91775700 | 1.19939600 |
| C | -1.22561700 | 0.35451600 | -0.33712700 |
| H | -1.16480700 | 0.40266900 | -1.43456000 |
| O | -0.66972000 | 1.53059100 | 0.23551300 |
| H | 0.27635500 | 1.55815200 | 0.00958200 |
| C | -2.69962800 | 0.30051300 | 0.08672600 |
| H | -2.75870500 | 0.38496300 | 1.17252900 |
| N | -3.43929900 | 1.41362700 | -0.47605800 |
| H | -3.41609500 | 1.53483100 | -1.47799300 |
| C | -4.12088400 | 2.32534400 | 0.26438000 |
| O | -4.18452000 | 2.27886500 | 1.49337200 |
| C | -4.81058700 | 3.42352400 | -0.51841500 |
| H | -4.67216100 | 3.34553000 | -1.59752900 |
| H | -5.87834000 | 3.39303200 | -0.29177000 |
| H | -4.42554500 | 4.38667300 | -0.17707300 |
| C | -3.32571800 | -1.04391200 | -0.30663300 |
| H | -3.37270400 | -1.14859200 | -1.40457400 |
| O | -4.59206200 | -1.12265000 | 0.24942800 |
| C | -5.38651900 | -2.20564100 | -0.25211100 |
| H | -4.93035900 | -3.16958800 | -0.01352400 |
| H | -5.51494000 | -2.11875200 | -1.33702400 |
| H | -6.35713000 | -2.12860000 | 0.23542100 |
| O | -2.53628600 | -2.10945100 | 0.22009600 |
| C | -1.21760200 | -2.17387000 | -0.34121300 |
| H | -1.30621400 | -2.21426400 | -1.43548200 |
| C | -0.65088600 | -3.50617600 | 0.16970400 |
| H | -1.33804000 | -4.29234100 | -0.14777600 |
| C | 3.16149800 | -1.12268800 | -0.04958100 |
| H | 3.29921900 | -1.18094800 | -1.13463500 |
| C | 4.35669200 | -0.41272100 | 0.58201900 |
| H | 4.22243800 | -0.41356400 | 1.67079900 |
| C | 4.41758200 | 1.04575400 | 0.12929300 |
| H | 5.22751500 | 1.57598700 | 0.63578400 |
| O | 2.98220300 | -2.42048200 | 0.51001300 |
| H | 3.80773600 | -2.91074200 | 0.40471300 |
| O | 0.63108900 | -3.84896200 | -0.32939500 |
| H | 1.31001400 | -3.23954300 | 0.00329600 |
| H | -0.65613100 | -3.47880100 | 1.26800600 |
| F | 4.70194300 | 1.08582400 | -1.25308900 |
| O | 5.51291600 | -1.15900200 | 0.22228200 |
| H | 6.21722200 | -0.98672200 | 0.85737000 |
| E(RB3LYP): -1494.60920934 | | | |
| Sum of electronic and thermal free energies: -1494.234768 | | | |
| Number of imaginary frequencies: 0 | | | |

## 4′F-LN **7** Conformer K

| **Atom** | **x** | **y** | **z** |
| --- | --- | --- | --- |
| H | 4.20864900 | 4.57933700 | 0.13513900 |
| O | 4.13905600 | 3.69437300 | 0.50822200 |
| C | 3.09216000 | 2.99635400 | -0.16502200 |
| H | 3.25199600 | 2.97824100 | -1.24730000 |
| H | 2.11494700 | 3.44544800 | 0.04192000 |
| C | 3.09617400 | 1.57114600 | 0.37009500 |
| H | 2.96685800 | 1.59842900 | 1.46060700 |
| O | 1.98187500 | 0.90471100 | -0.24237600 |
| C | 1.78113200 | -0.43948900 | 0.21517000 |
| H | 1.58686600 | -0.43021900 | 1.29730000 |
| O | 0.70639100 | -0.97474000 | -0.48139600 |
| C | -0.60288500 | -0.87060400 | 0.10246300 |
| H | -0.51775700 | -0.84950700 | 1.19528500 |
| C | -1.30430000 | 0.41152400 | -0.37332400 |
| H | -1.25849700 | 0.41827400 | -1.47267300 |
| O | -0.69226800 | 1.58178200 | 0.14894600 |
| H | 0.24595700 | 1.57652400 | -0.10732000 |
| C | -2.77305600 | 0.43604300 | 0.07274300 |
| H | -2.81096400 | 0.55978000 | 1.15570900 |
| N | -3.47479700 | 1.55952900 | -0.51722700 |
| H | -3.47092800 | 1.63976700 | -1.52350400 |
| C | -4.10702600 | 2.52325700 | 0.20061000 |
| O | -4.14620500 | 2.52631200 | 1.43158500 |
| C | -4.77330300 | 3.61570400 | -0.61017800 |
| H | -4.65890800 | 3.49353600 | -1.68799200 |
| H | -5.83693000 | 3.63077300 | -0.36333400 |
| H | -4.34910500 | 4.57672200 | -0.31206800 |
| C | -3.46357600 | -0.89170200 | -0.26528800 |
| H | -3.53579600 | -1.02883500 | -1.35846600 |
| O | -4.72184900 | -0.89718900 | 0.31437700 |
| C | -5.57038600 | -1.96104900 | -0.13684000 |
| H | -5.15229900 | -2.93515600 | 0.12866600 |
| H | -5.71264000 | -1.90628600 | -1.22215600 |
| H | -6.52868800 | -1.82498800 | 0.36201900 |
| O | -2.71131600 | -1.97323200 | 0.28160300 |
| C | -1.40704400 | -2.11105600 | -0.30139500 |
| H | -1.52103300 | -2.17258100 | -1.39236500 |
| C | -0.89561400 | -3.45794000 | 0.22834500 |
| H | -1.62797200 | -4.21510600 | -0.05698000 |
| C | 3.01644800 | -1.28085300 | -0.09084700 |
| H | 3.12828800 | -1.34613100 | -1.17840700 |
| C | 4.26322300 | -0.63944900 | 0.51372800 |
| H | 4.15709600 | -0.64193500 | 1.60571900 |
| C | 4.38805900 | 0.81565600 | 0.06827500 |
| H | 5.23442500 | 1.30838300 | 0.54955700 |
| O | 2.77959000 | -2.56799000 | 0.47299000 |
| H | 3.57494700 | -3.10196600 | 0.34955100 |
| O | 0.35632900 | -3.87485000 | -0.28901600 |
| H | 1.07345400 | -3.29676000 | 0.01914600 |
| H | -0.87599100 | -3.40668700 | 1.32570400 |
| F | 4.62564500 | 0.85143200 | -1.32276400 |
| O | 5.36694200 | -1.44688800 | 0.11978900 |
| H | 6.09839700 | -1.31221700 | 0.73270800 |
| E(RB3LYP): -1494.60816640 | | | |
| Sum of electronic and thermal free energies: -1494.233925 | | | |
| Number of imaginary frequencies: 0 | | | |

## 4′F-LN **7** Conformer L

| **Atom** | **x** | **y** | **z** |
| --- | --- | --- | --- |
| H | 1.94494100 | 4.16009700 | 1.12360900 |
| O | 2.32462700 | 3.38047600 | 0.70521800 |
| C | 2.75604900 | 2.49494800 | 1.73705100 |
| H | 1.96756300 | 2.36715000 | 2.48656900 |
| H | 3.65266000 | 2.87546900 | 2.24185900 |
| C | 3.07655900 | 1.12093300 | 1.16953300 |
| H | 3.28650800 | 0.47440600 | 2.03532900 |
| O | 1.91940500 | 0.64470800 | 0.48134300 |
| C | 1.96935100 | -0.72901600 | 0.07176300 |
| H | 2.07118600 | -1.37486300 | 0.95476300 |
| O | 0.78428100 | -0.98438200 | -0.60383200 |
| C | -0.43114400 | -0.84345200 | 0.16023700 |
| H | -0.20232000 | -0.57547700 | 1.19630300 |
| C | -1.21216900 | 0.31247100 | -0.48655600 |
| H | -1.16797600 | 0.14637100 | -1.57389100 |
| O | -0.64389900 | 1.57680800 | -0.17033000 |
| H | 0.31891800 | 1.47498700 | -0.07136100 |
| C | -2.68379100 | 0.34319900 | -0.06086300 |
| H | -2.75514600 | 0.61620400 | 0.99237300 |
| N | -3.41536900 | 1.33929800 | -0.82080700 |
| H | -3.37066900 | 1.28800700 | -1.82816600 |
| C | -4.13050800 | 2.34797900 | -0.26102700 |
| O | -4.22541400 | 2.50824200 | 0.95673400 |
| C | -4.81495000 | 3.28782300 | -1.23225900 |
| H | -4.67252300 | 3.01738600 | -2.27928900 |
| H | -5.88345000 | 3.29966100 | -1.00800000 |
| H | -4.42977800 | 4.29657100 | -1.06831200 |
| C | -3.29279200 | -1.05374700 | -0.22116600 |
| H | -3.25931600 | -1.38270500 | -1.27455900 |
| O | -4.59604500 | -1.03712800 | 0.24758400 |
| C | -5.34933100 | -2.21333700 | -0.07509500 |
| H | -4.91036100 | -3.09983800 | 0.38940700 |
| H | -5.39729900 | -2.35388700 | -1.16100900 |
| H | -6.35356800 | -2.05433500 | 0.31475800 |
| O | -2.54557800 | -1.97345800 | 0.57131000 |
| C | -1.20045800 | -2.17545100 | 0.13419100 |
| H | -1.18223400 | -2.57026900 | -0.89085700 |
| C | -0.66158100 | -3.22024000 | 1.10820800 |
| H | -1.31608200 | -4.09748000 | 1.05620900 |
| C | 3.14220600 | -0.91965300 | -0.89022500 |
| H | 2.94072800 | -0.31139400 | -1.78020600 |
| C | 4.43614200 | -0.42635500 | -0.23223100 |
| H | 4.63970400 | -1.05701100 | 0.64222500 |
| C | 4.29657900 | 1.01681700 | 0.25497600 |
| H | 5.19852200 | 1.33819200 | 0.78220400 |
| O | 3.25043800 | -2.29411200 | -1.22971600 |
| H | 4.07892000 | -2.39681700 | -1.71558200 |
| O | 0.68038300 | -3.55512600 | 0.76424400 |
| H | 0.99442200 | -4.21435900 | 1.39193000 |
| H | -0.71833700 | -2.80745900 | 2.12308800 |
| F | 4.15430500 | 1.85593300 | -0.86596100 |
| O | 5.46716800 | -0.58547600 | -1.20282800 |
| H | 6.31991700 | -0.63902400 | -0.75716500 |
| E(RB3LYP): -1494.59538909 | | | |
| Sum of electronic and thermal free energies: -1494.223527 | | | |
| Number of imaginary frequencies: 0 | | | |

## 6′F-LN **8** Conformer A

| **Atom** | **x** | **y** | **z** |
| --- | --- | --- | --- |
| C | 3.11299500 | 2.87926200 | 0.43552200 |
| H | 3.23691000 | 3.15099200 | -0.61219200 |
| H | 2.17626600 | 3.27626500 | 0.82833300 |
| C | 3.17344700 | 1.37066200 | 0.62058700 |
| H | 3.09664100 | 1.13940500 | 1.69125600 |
| O | 2.02780700 | 0.85816900 | -0.07157400 |
| C | 1.85083300 | -0.56161500 | 0.06241000 |
| H | 1.73290200 | -0.81153800 | 1.12687700 |
| O | 0.72230000 | -0.90792900 | -0.66129700 |
| C | -0.55014200 | -0.78867200 | -0.00261200 |
| H | -0.40713300 | -0.70110600 | 1.07869500 |
| C | -1.28687000 | 0.44962200 | -0.51695000 |
| H | -1.26123400 | 0.41271700 | -1.61669300 |
| O | -0.68922300 | 1.65398000 | -0.05666000 |
| H | 0.27443600 | 1.57470200 | -0.16164700 |
| C | -2.74805100 | 0.44765800 | -0.04810100 |
| H | -2.77672700 | 0.58797600 | 1.03315600 |
| N | -3.48389200 | 1.54341300 | -0.64887400 |
| H | -3.47746800 | 1.61660100 | -1.65563300 |
| C | -4.15593100 | 2.48765200 | 0.05859200 |
| O | -4.20422700 | 2.49655300 | 1.28917500 |
| C | -4.85445500 | 3.55094200 | -0.76377100 |
| H | -4.72656400 | 3.42747900 | -1.83992200 |
| H | -5.91998800 | 3.52850200 | -0.52582000 |
| H | -4.46807600 | 4.52831800 | -0.46746200 |
| C | -3.41028900 | -0.90318600 | -0.36065600 |
| H | -3.46584700 | -1.06734600 | -1.45114900 |
| O | -4.67476900 | -0.91883800 | 0.20342000 |
| C | -5.49856500 | -2.00832600 | -0.23238600 |
| H | -5.06845100 | -2.96798900 | 0.06466300 |
| H | -5.62518800 | -1.98396500 | -1.32071200 |
| H | -6.46637200 | -1.87552600 | 0.24865300 |
| O | -2.64869500 | -1.96312700 | 0.21939700 |
| C | -1.32342600 | -2.07623300 | -0.30441500 |
| H | -1.35245400 | -2.23041500 | -1.39275100 |
| C | -0.69315200 | -3.30159100 | 0.34367900 |
| H | 0.33163500 | -3.40810200 | -0.01300300 |
| C | 3.06004600 | -1.28460600 | -0.52291000 |
| H | 3.10362200 | -1.05486500 | -1.59360400 |
| C | 4.33896900 | -0.78837600 | 0.15223100 |
| H | 4.32327800 | -1.10154400 | 1.20238900 |
| C | 4.45979200 | 0.74103000 | 0.08240500 |
| H | 5.29278800 | 1.06350700 | 0.71971600 |
| O | 2.87717900 | -2.67867200 | -0.30721800 |
| H | 3.69938900 | -3.11694800 | -0.56065600 |
| O | -0.62395800 | -3.18545100 | 1.76626000 |
| H | -1.52215800 | -3.05116300 | 2.09280400 |
| H | -1.26843000 | -4.18771600 | 0.05244900 |
| O | 5.42534300 | -1.41990100 | -0.53581600 |
| H | 6.18889800 | -1.47026400 | 0.05079800 |
| O | 4.66550500 | 1.17575700 | -1.25763700 |
| H | 5.34471900 | 0.60497200 | -1.64041900 |
| F | 4.16917100 | 3.46038800 | 1.16420500 |
| E(RB3LYP): -1494.60912881 | | | |
| Sum of electronic and thermal free energies: -1494.235278 | | | |
| Number of imaginary frequencies: 0 | | | |

## 6′F-LN **8** Conformer B

| **Atom** | **x** | **y** | **z** |
| --- | --- | --- | --- |
| C | -3.21456800 | 2.87690300 | -0.28147200 |
| H | -3.32814400 | 3.06726100 | 0.78519200 |
| H | -2.29343100 | 3.32415300 | -0.65683600 |
| C | -3.24414500 | 1.38513000 | -0.57750100 |
| H | -3.17575000 | 1.23560200 | -1.66311100 |
| O | -2.07805800 | 0.84891400 | 0.06173300 |
| C | -1.87127300 | -0.55227200 | -0.18080200 |
| H | -1.75743200 | -0.71755900 | -1.26210700 |
| O | -0.72957000 | -0.92994100 | 0.50680700 |
| C | 0.53915800 | -0.74234800 | -0.14219900 |
| H | 0.39616700 | -0.61781200 | -1.22164500 |
| C | 1.23387800 | 0.50388700 | 0.41892800 |
| H | 1.19200300 | 0.42806900 | 1.51612800 |
| O | 0.60608900 | 1.70252400 | -0.01188900 |
| H | -0.35352500 | 1.60430500 | 0.11422500 |
| C | 2.70311300 | 0.56745500 | -0.02326000 |
| H | 2.74537800 | 0.75526600 | -1.09684400 |
| N | 3.39200100 | 1.65956000 | 0.63656200 |
| H | 3.38376200 | 1.67732400 | 1.64585000 |
| C | 4.02751300 | 2.66433600 | -0.01957300 |
| O | 4.07457900 | 2.73837200 | -1.24794700 |
| C | 4.68565400 | 3.71021400 | 0.85659100 |
| H | 4.56758400 | 3.52600200 | 1.92511800 |
| H | 5.75018000 | 3.74343300 | 0.61549900 |
| H | 4.25897300 | 4.68529900 | 0.61257000 |
| C | 3.40647200 | -0.77242000 | 0.24209000 |
| H | 3.45297300 | -0.98269000 | 1.32495900 |
| O | 4.67727000 | -0.72344100 | -0.30505900 |
| C | 5.52939300 | -1.80575100 | 0.09292200 |
| H | 5.13544000 | -2.76364600 | -0.25566700 |
| H | 5.63898800 | -1.82887300 | 1.18307500 |
| H | 6.49935400 | -1.62001200 | -0.36566400 |
| O | 2.68173200 | -1.82462800 | -0.39610400 |
| C | 1.36372200 | -2.00359000 | 0.12756700 |
| H | 1.41245900 | -2.17682400 | 1.21204500 |
| C | 0.78674500 | -3.23712200 | -0.54795600 |
| H | 0.78046200 | -3.07820900 | -1.63419400 |
| C | -3.05828900 | -1.34559700 | 0.35786100 |
| H | -3.09403500 | -1.20026600 | 1.44350500 |
| C | -4.35643100 | -0.82825900 | -0.26261300 |
| H | -4.34900800 | -1.05882100 | -1.33405800 |
| C | -4.50899900 | 0.68794900 | -0.07326500 |
| H | -5.35712500 | 1.03987300 | -0.67396900 |
| O | -2.84677900 | -2.71411300 | 0.03309200 |
| H | -3.65588300 | -3.18966300 | 0.26016700 |
| O | 1.52415900 | -4.41187400 | -0.21180000 |
| H | 2.44387800 | -4.25635400 | -0.45944200 |
| H | -0.23878400 | -3.38937300 | -0.21172000 |
| O | -5.41945200 | -1.53518500 | 0.38743100 |
| H | -6.18866100 | -1.56001600 | -0.19339600 |
| O | -4.70507400 | 1.01407200 | 1.29853300 |
| H | -5.36874200 | 0.40269900 | 1.64406700 |
| F | -4.29465700 | 3.48502300 | -0.95021600 |
| E(RB3LYP): -1494.60849401 | | | |
| Sum of electronic and thermal free energies: -1494.235231 | | | |
| Number of imaginary frequencies: 0 | | | |

## 6′F-LN **8** Conformer C

| **Atom** | **x** | **y** | **z** |
| --- | --- | --- | --- |
| C | 3.18306200 | -3.02227700 | -0.11385800 |
| H | 4.04687000 | -3.56066500 | -0.50653500 |
| H | 3.12226800 | -3.13384700 | 0.96806800 |
| C | 3.23538300 | -1.56440100 | -0.50956200 |
| H | 3.13949600 | -1.48879700 | -1.60096600 |
| O | 2.11558200 | -0.91978200 | 0.11421900 |
| C | 1.96991200 | 0.46582300 | -0.22674700 |
| H | 1.84187900 | 0.56263100 | -1.31509600 |
| O | 0.86075900 | 0.94711300 | 0.45036000 |
| C | -0.42544000 | 0.79977400 | -0.17268400 |
| H | -0.30926600 | 0.72193000 | -1.26004400 |
| C | -1.13794100 | -0.45786900 | 0.34472600 |
| H | -1.05476200 | -0.44482400 | 1.44201300 |
| O | -0.57112000 | -1.64995200 | -0.17728900 |
| H | 0.38792700 | -1.63725000 | -0.00967100 |
| C | -2.62439200 | -0.45608100 | -0.04462700 |
| H | -2.71031200 | -0.58757700 | -1.12394500 |
| N | -3.32025400 | -1.56196900 | 0.58438400 |
| H | -3.25854100 | -1.64479400 | 1.58852300 |
| C | -4.02081200 | -2.50521000 | -0.09603100 |
| O | -4.13321100 | -2.50294300 | -1.32249400 |
| C | -4.66846700 | -3.58161700 | 0.75062800 |
| H | -4.48304300 | -3.47030800 | 1.81969800 |
| H | -5.74548300 | -3.56199200 | 0.57120100 |
| H | -4.29432200 | -4.55335800 | 0.42192500 |
| C | -3.27838700 | 0.88707900 | 0.31128500 |
| H | -3.27391600 | 1.04821900 | 1.40354900 |
| O | -4.57153700 | 0.89860800 | -0.18343600 |
| C | -5.37427700 | 1.98476600 | 0.29762900 |
| H | -4.96600900 | 2.94646500 | -0.02298300 |
| H | -5.44008000 | 1.96041500 | 1.39127100 |
| H | -6.36676800 | 1.84796600 | -0.12887900 |
| O | -2.54947400 | 1.94630700 | -0.30952600 |
| C | -1.21022800 | 2.06820700 | 0.17361400 |
| H | -1.21767000 | 2.19967000 | 1.26507400 |
| C | -0.62348400 | 3.31270100 | -0.47326800 |
| H | -0.65803000 | 3.19656700 | -1.56438800 |
| C | 3.20547100 | 1.23252400 | 0.23512700 |
| H | 3.25845800 | 1.15616500 | 1.32721300 |
| C | 4.46180800 | 0.61016600 | -0.37538400 |
| H | 4.43991800 | 0.76985900 | -1.45935500 |
| C | 4.54846400 | -0.89731600 | -0.09190700 |
| H | 5.36175900 | -1.32394300 | -0.69379700 |
| O | 3.05725400 | 2.58594300 | -0.17508000 |
| H | 3.89121400 | 3.03461400 | 0.01331100 |
| O | -1.31765500 | 4.49163900 | -0.06673400 |
| H | -2.24914400 | 4.36861700 | -0.28721700 |
| H | 0.41634600 | 3.42520700 | -0.16752800 |
| O | 5.57397800 | 1.30058300 | 0.20530700 |
| H | 6.32635600 | 1.25891400 | -0.39619100 |
| O | 4.77429700 | -1.14817800 | 1.29146900 |
| H | 5.47361400 | -0.54651300 | 1.57976300 |
| F | 2.02311200 | -3.60687600 | -0.66819300 |
| E(RB3LYP): -1494.61060368 | | | |
| Sum of electronic and thermal free energies: -1494.236744 | | | |
| Number of imaginary frequencies: 0 | | | |

## 6′F-LN **8** Conformer D

| **Atom** | **x** | **y** | **z** |
| --- | --- | --- | --- |
| C | 3.05682400 | 3.03670200 | 0.39612700 |
| H | 3.91424600 | 3.54597200 | 0.83855700 |
| H | 2.97923900 | 3.27078500 | -0.66496400 |
| C | 3.14784300 | 1.54432100 | 0.61903100 |
| H | 3.05779300 | 1.33996300 | 1.69435800 |
| O | 2.04606400 | 0.94590800 | -0.07690100 |
| C | 1.94064400 | -0.47494200 | 0.09333700 |
| H | 1.82439600 | -0.70810100 | 1.16197800 |
| O | 0.83780900 | -0.89559600 | -0.63077700 |
| C | -0.44311100 | -0.82776900 | 0.01849300 |
| H | -0.31117500 | -0.76502900 | 1.10212500 |
| C | -1.21809700 | 0.39919800 | -0.47013400 |
| H | -1.16579800 | 0.40139200 | -1.56969700 |
| O | -0.68599600 | 1.61092100 | 0.04679000 |
| H | 0.28185300 | 1.59600800 | -0.05747200 |
| C | -2.68987500 | 0.32716100 | -0.03891000 |
| H | -2.75032600 | 0.44999300 | 1.04311000 |
| N | -3.45399100 | 1.40245100 | -0.64235100 |
| H | -3.42279600 | 1.49228500 | -1.64730800 |
| C | -4.17912100 | 2.30975800 | 0.06014300 |
| O | -4.26014700 | 2.29821400 | 1.28916500 |
| C | -4.89658500 | 3.35831900 | -0.76509100 |
| H | -4.73136000 | 3.26015300 | -1.83877700 |
| H | -5.96698800 | 3.28733000 | -0.56070900 |
| H | -4.56075700 | 4.34519900 | -0.44016300 |
| C | -3.28950900 | -1.04447200 | -0.38368600 |
| H | -3.31544900 | -1.19511300 | -1.47783300 |
| O | -4.56626900 | -1.11600800 | 0.15061200 |
| C | -5.33432700 | -2.23353500 | -0.31352800 |
| H | -4.87253900 | -3.17801400 | -0.01511700 |
| H | -5.43704200 | -2.20416000 | -1.40435900 |
| H | -6.31774300 | -2.14512200 | 0.14574900 |
| O | -2.49985500 | -2.07821100 | 0.20126500 |
| C | -1.17276100 | -2.13221700 | -0.32287000 |
| H | -1.20630000 | -2.24192600 | -1.41718300 |
| C | -0.46948100 | -3.35602200 | 0.23387900 |
| H | 0.49601800 | -3.45136900 | -0.27133600 |
| C | 3.19357900 | -1.14300800 | -0.46389700 |
| H | 3.23935000 | -0.92914700 | -1.53791300 |
| C | 4.43336800 | -0.56531600 | 0.22005600 |
| H | 4.41779900 | -0.86073800 | 1.27537600 |
| C | 4.47836400 | 0.96776200 | 0.12721800 |
| H | 5.27987200 | 1.33777900 | 0.78043800 |
| O | 3.08862100 | -2.54123100 | -0.22589200 |
| H | 3.93587200 | -2.93592100 | -0.46811600 |
| O | -0.28519500 | -3.19936300 | 1.64579900 |
| H | 0.28473400 | -3.91056300 | 1.95563100 |
| H | -1.08161400 | -4.23835000 | 0.01574500 |
| O | 5.56344500 | -1.14598800 | -0.44096600 |
| H | 6.31702900 | -1.15440100 | 0.16040800 |
| O | 4.69860700 | 1.39415200 | -1.21366500 |
| H | 5.41136300 | 0.84865500 | -1.57232100 |
| F | 1.89278400 | 3.52747300 | 1.02686600 |
| E(RB3LYP): -1494.60840577 | | | |
| Sum of electronic and thermal free energies: -1494.235838 | | | |
| Number of imaginary frequencies: 0 | | | |

## 6′F-LN **8** Conformer E

| **Atom** | **x** | **y** | **z** |
| --- | --- | --- | --- |
| C | 2.98602500 | 2.85848300 | 1.08299800 |
| H | 2.07323000 | 3.07343900 | 1.64166800 |
| H | 3.85042100 | 3.26432900 | 1.61242100 |
| C | 3.13865100 | 1.36046000 | 0.91068200 |
| H | 3.10442600 | 0.95050600 | 1.93257400 |
| O | 2.01221200 | 0.88316600 | 0.16678700 |
| C | 1.90192000 | -0.54646500 | 0.12029900 |
| H | 1.78868900 | -0.93647100 | 1.14255800 |
| O | 0.79151700 | -0.84693800 | -0.65223300 |
| C | -0.48799600 | -0.79402400 | 0.00177500 |
| H | -0.35488100 | -0.73083800 | 1.08500200 |
| C | -1.26116500 | 0.43244900 | -0.48582200 |
| H | -1.22619600 | 0.42534900 | -1.58607300 |
| O | -0.70703100 | 1.64403700 | 0.00935100 |
| H | 0.26248800 | 1.57920100 | -0.03756800 |
| C | -2.72556300 | 0.37004100 | -0.03056200 |
| H | -2.76797100 | 0.49098300 | 1.05250000 |
| N | -3.49337900 | 1.45060100 | -0.61950600 |
| H | -3.47891900 | 1.54230000 | -1.62468000 |
| C | -4.20318600 | 2.35941700 | 0.09689600 |
| O | -4.26419400 | 2.34521800 | 1.32698600 |
| C | -4.92846600 | 3.41357500 | -0.71412700 |
| H | -4.78658300 | 3.31298800 | -1.79091200 |
| H | -5.99487000 | 3.35244900 | -0.48711400 |
| H | -4.57696100 | 4.39771000 | -0.39741800 |
| C | -3.33666000 | -0.99885900 | -0.36941500 |
| H | -3.37909900 | -1.14649700 | -1.46340300 |
| O | -4.60571900 | -1.06559800 | 0.18309600 |
| C | -5.38637400 | -2.17743700 | -0.27382300 |
| H | -4.92513200 | -3.12535300 | 0.01430600 |
| H | -5.50481800 | -2.14338000 | -1.36292000 |
| H | -6.36248000 | -2.08563500 | 0.20012400 |
| O | -2.54395100 | -2.03895700 | 0.20123600 |
| C | -1.22235700 | -2.09583500 | -0.33747400 |
| H | -1.26756500 | -2.20191900 | -1.43164200 |
| C | -0.51535800 | -3.32235100 | 0.20818900 |
| H | 0.44052400 | -3.42363800 | -0.31441500 |
| C | 3.14258700 | -1.13914300 | -0.54017200 |
| H | 3.17065300 | -0.78823000 | -1.57807700 |
| C | 4.40149000 | -0.66149400 | 0.18518300 |
| H | 4.41206100 | -1.09234900 | 1.19260500 |
| C | 4.44969000 | 0.87303400 | 0.28630100 |
| H | 5.26978200 | 1.15647400 | 0.96028500 |
| O | 3.02911600 | -2.55612900 | -0.48402900 |
| H | 3.86769700 | -2.92154000 | -0.79312600 |
| O | -0.30466700 | -3.16397800 | 1.61636100 |
| H | 0.25071600 | -3.88873500 | 1.92131400 |
| H | -1.13516000 | -4.20209300 | 0.00204300 |
| O | 5.51080200 | -1.15765900 | -0.57404400 |
| H | 6.27851700 | -1.24713100 | 0.00232900 |
| O | 4.64373400 | 1.45257700 | -0.99723200 |
| H | 5.33262000 | 0.93261400 | -1.43316700 |
| F | 2.87761000 | 3.52713900 | -0.14871900 |
| E(RB3LYP): -1494.60459625 | | | |
| Sum of electronic and thermal free energies: -1494.231652 | | | |
| Number of imaginary frequencies: 0 | | | |

## 6′F-LN **8** Conformer F

| **Atom** | **x** | **y** | **z** |
| --- | --- | --- | --- |
| C | -3.16197600 | -2.97587500 | 0.62306700 |
| H | -2.24931900 | -3.33977300 | 1.09881200 |
| H | -4.03373100 | -3.43025000 | 1.09804000 |
| C | -3.23589900 | -1.46635200 | 0.74204900 |
| H | -3.16085800 | -1.26026400 | 1.82128400 |
| O | -2.09662300 | -0.91211200 | 0.07072600 |
| C | -1.91875000 | 0.49691100 | 0.28041700 |
| H | -1.77035800 | 0.68711200 | 1.35375800 |
| O | -0.81108400 | 0.88996500 | -0.45446600 |
| C | 0.48200000 | 0.76696200 | 0.15923300 |
| H | 0.37807800 | 0.71827100 | 1.24931100 |
| C | 1.18542500 | -0.50383800 | -0.33329000 |
| H | 1.11176800 | -0.50845300 | -1.43143600 |
| O | 0.59636800 | -1.68015900 | 0.20099000 |
| H | -0.36646400 | -1.62339300 | 0.06933200 |
| C | 2.66718300 | -0.50465600 | 0.06980700 |
| H | 2.74267800 | -0.60122200 | 1.15356300 |
| N | 3.35984000 | -1.63587400 | -0.51566600 |
| H | 3.30611900 | -1.75383000 | -1.51678900 |
| C | 4.04846100 | -2.55925000 | 0.20346300 |
| O | 4.14997400 | -2.51499300 | 1.43006200 |
| C | 4.69662400 | -3.66780100 | -0.59991100 |
| H | 4.51659500 | -3.59427600 | -1.67315800 |
| H | 5.77285700 | -3.64485000 | -0.41605600 |
| H | 4.31784900 | -4.62580400 | -0.23797600 |
| C | 3.33268800 | 0.82288300 | -0.32447400 |
| H | 3.33483900 | 0.94948600 | -1.42100100 |
| O | 4.62332700 | 0.84113300 | 0.17620700 |
| C | 5.43740700 | 1.90289500 | -0.33932700 |
| H | 5.03423800 | 2.87877100 | -0.05744900 |
| H | 5.51003000 | 1.83733000 | -1.43082500 |
| H | 6.42606000 | 1.77484200 | 0.09861800 |
| O | 2.60861800 | 1.90732400 | 0.25856400 |
| C | 1.26932900 | 2.02155600 | -0.22795600 |
| H | 1.27631800 | 2.11921400 | -1.32291200 |
| C | 0.68799400 | 3.28774500 | 0.38012900 |
| H | 0.72402400 | 3.20651600 | 1.47435900 |
| C | -3.14201200 | 1.25426400 | -0.22760500 |
| H | -3.21011500 | 1.09519300 | -1.30978200 |
| C | -4.40609900 | 0.71382100 | 0.44084700 |
| H | -4.36754300 | 0.95076400 | 1.51007200 |
| C | -4.53145800 | -0.80814400 | 0.25831300 |
| H | -5.35086100 | -1.17304400 | 0.89300600 |
| O | -2.95323600 | 2.63130700 | 0.07475800 |
| H | -3.78073700 | 3.08444700 | -0.13078800 |
| O | 1.38519400 | 4.45090400 | -0.06520200 |
| H | 2.31654700 | 4.33284000 | 0.15849400 |
| H | -0.35211300 | 3.39383600 | 0.07261700 |
| O | -5.50580300 | 1.39348500 | -0.17598500 |
| H | -6.25492600 | 1.40600100 | 0.43091900 |
| O | -4.78174000 | -1.12753500 | -1.10390200 |
| H | -5.45456500 | -0.50601000 | -1.41377700 |
| F | -3.12452900 | -3.40484600 | -0.71479500 |
| E(RB3LYP): -1494.60653898 | | | |
| Sum of electronic and thermal free energies: -1494.232494 | | | |
| Number of imaginary frequencies: 0 | | | |

## 6′F-LN **8** Conformer G

| **Atom** | **x** | **y** | **z** |
| --- | --- | --- | --- |
| C | -3.23870100 | 2.79400800 | -0.93033900 |
| H | -4.17089600 | 3.16388200 | -1.36013200 |
| H | -3.06010900 | 3.24879100 | 0.04337200 |
| C | -3.25786200 | 1.28578500 | -0.83195700 |
| H | -3.27041500 | 0.86473500 | -1.84635900 |
| O | -2.05418700 | 0.88803800 | -0.16285200 |
| C | -1.87879200 | -0.53287400 | -0.06189900 |
| H | -1.86413300 | -0.97179400 | -1.07053400 |
| O | -0.68159800 | -0.75529500 | 0.59615500 |
| C | 0.51791400 | -0.73948700 | -0.19857000 |
| H | 0.26580000 | -0.85113300 | -1.25679800 |
| C | 1.26556800 | 0.57935300 | 0.01072300 |
| H | 1.32679000 | 0.76273400 | 1.08918500 |
| O | 0.61686900 | 1.66779000 | -0.64015400 |
| H | -0.33567100 | 1.61906000 | -0.44726200 |
| C | 2.68999200 | 0.49632500 | -0.56181700 |
| H | 2.61144900 | 0.41342000 | -1.64798100 |
| N | 3.46015700 | 1.70656300 | -0.29471400 |
| H | 3.71819100 | 2.26619800 | -1.09127800 |
| C | 3.82003500 | 2.14494100 | 0.93615400 |
| O | 3.54530600 | 1.52905800 | 1.96899300 |
| C | 4.58938800 | 3.44936500 | 0.97871100 |
| H | 4.78396100 | 3.87785700 | -0.00507800 |
| H | 4.01976200 | 4.16649700 | 1.57376500 |
| H | 5.53939600 | 3.27497600 | 1.48787800 |
| C | 3.40106100 | -0.77818000 | -0.08057900 |
| H | 3.53906100 | -0.76281500 | 1.00918600 |
| O | 4.62043000 | -0.87508800 | -0.73544500 |
| C | 5.49733700 | -1.86786800 | -0.18892300 |
| H | 5.06785300 | -2.86810900 | -0.28761300 |
| H | 5.70335400 | -1.66054700 | 0.86736100 |
| H | 6.42453400 | -1.81005200 | -0.75732500 |
| O | 2.61041200 | -1.91448200 | -0.43494400 |
| C | 1.35469800 | -1.94527700 | 0.24424100 |
| H | 1.51501700 | -1.88474700 | 1.33092600 |
| C | 0.66741000 | -3.26798300 | -0.03881300 |
| H | -0.22818800 | -3.32588100 | 0.58685800 |
| C | -3.02107100 | -1.12417600 | 0.75815500 |
| H | -2.96366900 | -0.70204200 | 1.76799700 |
| C | -4.36109000 | -0.74414900 | 0.12695600 |
| H | -4.44368000 | -1.24638600 | -0.84351600 |
| C | -4.48961500 | 0.77266700 | -0.08066200 |
| H | -5.37852000 | 0.97102000 | -0.69425300 |
| O | -2.85704900 | -2.53690200 | 0.78554600 |
| H | -3.64700500 | -2.90747000 | 1.19898800 |
| O | 0.31686500 | -3.33706700 | -1.42641500 |
| H | -0.22041700 | -4.12417400 | -1.56208600 |
| H | 1.35140200 | -4.08024500 | 0.23168100 |
| O | -5.37460100 | -1.22131700 | 1.01954800 |
| H | -6.18645400 | -1.38160000 | 0.52491500 |
| O | -4.58787500 | 1.45557200 | 1.16516200 |
| H | -5.21990700 | 0.96672200 | 1.70891900 |
| F | -2.18586800 | 3.18866800 | -1.78454000 |
| E(RB3LYP): -1494.60695711 | | | |
| Sum of electronic and thermal free energies: -1494.234058 | | | |
| Number of imaginary frequencies: 0 | | | |

## 6′F-LN **8** Conformer H

| **Atom** | **x** | **y** | **z** |
| --- | --- | --- | --- |
| C | -4.45580900 | 1.76485700 | -0.92287700 |
| H | -5.27189600 | 1.64094300 | -1.63661400 |
| H | -4.84850500 | 1.96598600 | 0.07329700 |
| C | -3.56033400 | 0.54648900 | -0.91760200 |
| H | -3.02805700 | 0.49992500 | -1.87846500 |
| O | -2.61825300 | 0.71469200 | 0.14674500 |
| C | -1.65754700 | -0.32844300 | 0.21697200 |
| H | -1.10419200 | -0.38109300 | -0.73062100 |
| O | -0.81918200 | -0.04992200 | 1.29656700 |
| C | 0.52641100 | 0.37880100 | 1.03761700 |
| H | 0.94179600 | 0.53914400 | 2.03257500 |
| C | 1.33972600 | -0.72463200 | 0.34631600 |
| H | 0.89973500 | -0.93363900 | -0.63936900 |
| O | 1.35120200 | -1.91262000 | 1.11968700 |
| H | 0.45463900 | -2.28869000 | 1.07164900 |
| C | 2.78241600 | -0.25462700 | 0.12470600 |
| H | 3.27740800 | -0.13170900 | 1.08884500 |
| N | 3.53911700 | -1.23224500 | -0.63237600 |
| H | 3.17371800 | -1.51835700 | -1.52895700 |
| C | 4.69090700 | -1.80419000 | -0.19715600 |
| O | 5.20246400 | -1.53781700 | 0.89114400 |
| C | 5.32492400 | -2.80612300 | -1.13988900 |
| H | 4.76086400 | -2.95997600 | -2.06071500 |
| H | 6.32916200 | -2.45781000 | -1.39056900 |
| H | 5.42461600 | -3.75958700 | -0.61725600 |
| C | 2.77830100 | 1.11179100 | -0.58200900 |
| H | 2.34569000 | 1.01888900 | -1.59431400 |
| O | 4.08065500 | 1.58038000 | -0.64732700 |
| C | 4.25583800 | 2.70688300 | -1.51624700 |
| H | 3.68440800 | 3.56835400 | -1.16198800 |
| H | 3.94697800 | 2.45701100 | -2.53772600 |
| H | 5.31889100 | 2.94268800 | -1.50639500 |
| O | 2.00334100 | 2.05155600 | 0.15992600 |
| C | 0.61808500 | 1.70515600 | 0.26681600 |
| H | 0.18979400 | 1.59256600 | -0.74035500 |
| C | -0.11475500 | 2.85001600 | 0.93975700 |
| H | -1.18553900 | 2.63267500 | 0.90274300 |
| C | -2.35178200 | -1.66564500 | 0.47212900 |
| H | -2.84219300 | -1.61141000 | 1.44951400 |
| C | -3.39074100 | -1.93347500 | -0.61130900 |
| H | -2.87164300 | -2.08746300 | -1.56408500 |
| C | -4.36146400 | -0.75066700 | -0.75473200 |
| H | -4.96857100 | -0.90013300 | -1.65752900 |
| O | -1.33962600 | -2.67442900 | 0.47016500 |
| H | -1.76899600 | -3.53369700 | 0.56648000 |
| O | 0.33861500 | 2.98134400 | 2.29136000 |
| H | -0.20548600 | 3.64486300 | 2.72760200 |
| H | 0.08430400 | 3.76590300 | 0.37140800 |
| O | -4.08584600 | -3.12443400 | -0.22609300 |
| H | -4.45070300 | -3.54986200 | -1.01074100 |
| O | -5.20051300 | -0.63330900 | 0.39022600 |
| H | -5.52574000 | -1.51945000 | 0.59781000 |
| F | -3.70913500 | 2.89433100 | -1.32374800 |
| E(RB3LYP): -1494.60498161D | | | |
| Sum of electronic and thermal free energies: -1494.231448 | | | |
| Number of imaginary frequencies: 0 | | | |

## 6′F-LN **8** Conformer I

| **Atom** | **x** | **y** | **z** |
| --- | --- | --- | --- |
| C | -4.49058700 | 1.58668300 | 0.68812400 |
| H | -5.51494400 | 1.40093500 | 1.01488500 |
| H | -3.86417300 | 1.83106500 | 1.54581600 |
| C | -3.95594400 | 0.39069100 | -0.06759900 |
| H | -4.51563700 | 0.29064600 | -1.00682500 |
| O | -2.57514300 | 0.64598200 | -0.37110800 |
| C | -1.96646800 | -0.37034900 | -1.17222000 |
| H | -2.52804500 | -0.48985200 | -2.10591800 |
| O | -0.70203800 | 0.05124400 | -1.57615500 |
| C | 0.30834100 | 0.35042800 | -0.58925900 |
| H | -0.07449700 | 0.20717200 | 0.42352700 |
| C | 1.49628100 | -0.59023600 | -0.82466900 |
| H | 1.74938400 | -0.52949500 | -1.89421200 |
| O | 1.20158400 | -1.93095600 | -0.46859600 |
| H | 0.41988100 | -2.23528200 | -0.96269200 |
| C | 2.71558600 | -0.16181600 | 0.00463400 |
| H | 2.50648100 | -0.33759000 | 1.06070400 |
| N | 3.87990800 | -0.95299900 | -0.34470800 |
| H | 4.16624000 | -0.96490800 | -1.31275400 |
| C | 4.58582400 | -1.69968900 | 0.54225300 |
| O | 4.30010300 | -1.76911500 | 1.73835500 |
| C | 5.76698700 | -2.45711100 | -0.02869200 |
| H | 5.89889100 | -2.31941400 | -1.10264000 |
| H | 6.67150300 | -2.12768300 | 0.48700900 |
| H | 5.63246900 | -3.51996900 | 0.18215400 |
| C | 2.99253300 | 1.33762200 | -0.17099700 |
| H | 3.29795800 | 1.55833200 | -1.20936100 |
| O | 3.98658900 | 1.71120500 | 0.72020200 |
| C | 4.52546800 | 3.01769300 | 0.48179300 |
| H | 3.75847300 | 3.78659900 | 0.60350200 |
| H | 4.95099000 | 3.08080700 | -0.52631400 |
| H | 5.31387100 | 3.16651200 | 1.21807500 |
| O | 1.81709000 | 2.08538700 | 0.12718300 |
| C | 0.73495400 | 1.81563600 | -0.76682900 |
| H | 1.06363400 | 1.96137900 | -1.80718900 |
| C | -0.37318700 | 2.82538200 | -0.48997300 |
| H | -1.11747500 | 2.75198100 | -1.28851600 |
| C | -1.98970800 | -1.68829600 | -0.38935400 |
| H | -1.41668200 | -1.57078200 | 0.53592000 |
| C | -3.42827600 | -2.05141700 | -0.03297200 |
| H | -3.98301000 | -2.25052000 | -0.95669000 |
| C | -4.11186500 | -0.90696400 | 0.72977400 |
| H | -5.18211700 | -1.13240500 | 0.82314800 |
| O | -1.39614400 | -2.69175300 | -1.21488500 |
| H | -1.57630100 | -3.54844200 | -0.80369800 |
| O | -0.98605000 | 2.66090900 | 0.78526400 |
| H | -1.62095400 | 1.93634800 | 0.68325400 |
| H | 0.06651500 | 3.82439500 | -0.51496100 |
| O | -3.35645400 | -3.23391900 | 0.76938800 |
| H | -4.18412500 | -3.72317400 | 0.69544800 |
| O | -3.52741800 | -0.72348600 | 2.01467600 |
| H | -3.45652400 | -1.59462600 | 2.42699600 |
| F | -4.50863900 | 2.70949100 | -0.16381900 |
| E(RB3LYP): -1494.61179609 | | | |
| Sum of electronic and thermal free energies: -1494.235713 | | | |
| Number of imaginary frequencies: 0 | | | |

## 6′F-LN **8** Conformer J

| **Atom** | **x** | **y** | **z** |
| --- | --- | --- | --- |
| C | -2.84572100 | 2.66133100 | -1.40561400 |
| H | -3.73984600 | 3.10250200 | -1.84902100 |
| H | -2.52404700 | 3.24292400 | -0.54220100 |
| C | -3.09555500 | 1.21870900 | -1.02924100 |
| H | -3.22360500 | 0.63122900 | -1.94846700 |
| O | -1.94382600 | 0.74999500 | -0.32108000 |
| C | -1.98137600 | -0.64693900 | 0.01067200 |
| H | -2.09191900 | -1.24199000 | -0.90576700 |
| O | -0.78979900 | -0.94175200 | 0.65791400 |
| C | 0.41682200 | -0.79311700 | -0.11886900 |
| H | 0.17910400 | -0.45281100 | -1.13153500 |
| C | 1.23757500 | 0.29702700 | 0.58774200 |
| H | 1.21701600 | 0.05622000 | 1.66154400 |
| O | 0.67842800 | 1.58770000 | 0.37606800 |
| H | -0.28451000 | 1.49321100 | 0.28619700 |
| C | 2.69749400 | 0.33359700 | 0.12607500 |
| H | 2.74707300 | 0.67571600 | -0.90797100 |
| N | 3.46754000 | 1.26290800 | 0.93105800 |
| H | 3.45991000 | 1.13596400 | 1.93267300 |
| C | 4.18313800 | 2.29761200 | 0.42118900 |
| O | 4.23934600 | 2.54526000 | -0.78417500 |
| C | 4.91959000 | 3.15030500 | 1.43385500 |
| H | 4.80318000 | 2.80806900 | 2.46296300 |
| H | 5.98067700 | 3.15454200 | 1.17639400 |
| H | 4.55425500 | 4.17645200 | 1.35508600 |
| C | 3.28257100 | -1.08173500 | 0.17691200 |
| H | 3.27251900 | -1.47804500 | 1.20743100 |
| O | 4.57211400 | -1.05740800 | -0.32807600 |
| C | 5.30880100 | -2.26803200 | -0.11149200 |
| H | 4.84217400 | -3.10910300 | -0.63027600 |
| H | 5.37769000 | -2.49046700 | 0.95947600 |
| H | 6.30715300 | -2.09850200 | -0.51186700 |
| O | 2.49496000 | -1.93322300 | -0.65157400 |
| C | 1.15671400 | -2.13994000 | -0.19256300 |
| H | 1.16036800 | -2.59781900 | 0.80603200 |
| C | 0.58139600 | -3.11497300 | -1.21675900 |
| H | 1.21071900 | -4.01178800 | -1.21841000 |
| C | -3.14753100 | -0.87661800 | 0.97047300 |
| H | -2.95431000 | -0.27526400 | 1.86672800 |
| C | -4.44669700 | -0.39818200 | 0.31566700 |
| H | -4.66546800 | -1.05018600 | -0.53732200 |
| C | -4.35360300 | 1.05550900 | -0.17244800 |
| H | -5.23183200 | 1.27718100 | -0.79348300 |
| O | -3.23505600 | -2.25667200 | 1.29593200 |
| H | -4.04931500 | -2.37344500 | 1.80181200 |
| O | -0.76775200 | -3.42943600 | -0.88301700 |
| H | -1.10282800 | -4.04732300 | -1.54114200 |
| H | 0.64404700 | -2.64777800 | -2.20742200 |
| O | -5.46763700 | -0.52869500 | 1.31291900 |
| H | -6.32155500 | -0.65055400 | 0.88236500 |
| O | -4.28400800 | 1.96201300 | 0.92404400 |
| H | -4.94951300 | 1.68228700 | 1.56676900 |
| F | -1.81619700 | 2.72209500 | -2.36924700 |
| E(RB3LYP): -1494.60177822 | | | |
| Sum of electronic and thermal free energies: -1494.228220 | | | |
| Number of imaginary frequencies: 0 | | | |

## 6′F-LN **8** Conformer K

| **Atom** | **x** | **y** | **z** |
| --- | --- | --- | --- |
| C | -2.83720500 | 2.54408900 | -1.36970100 |
| H | -2.74694400 | 3.17192900 | -0.48375800 |
| H | -1.94616300 | 2.62683400 | -1.99276900 |
| C | -3.08619900 | 1.09289300 | -0.98961500 |
| H | -3.22136900 | 0.50291200 | -1.90595400 |
| O | -1.90043000 | 0.67068300 | -0.31207800 |
| C | -1.88012500 | -0.72749800 | 0.02597600 |
| H | -1.97976100 | -1.32704200 | -0.88834900 |
| O | -0.66974800 | -0.97232200 | 0.65711300 |
| C | 0.52023000 | -0.78405400 | -0.13688000 |
| H | 0.25712100 | -0.44652700 | -1.14415900 |
| C | 1.31577900 | 0.32789900 | 0.56423200 |
| H | 1.32091800 | 0.07967100 | 1.63657400 |
| O | 0.71014600 | 1.60035600 | 0.37016400 |
| H | -0.25153300 | 1.47592400 | 0.31611300 |
| C | 2.76549400 | 0.41592000 | 0.07852900 |
| H | 2.78668100 | 0.76477200 | -0.95429300 |
| N | 3.51766300 | 1.36601600 | 0.87593000 |
| H | 3.52646900 | 1.23691300 | 1.87721100 |
| C | 4.19644600 | 2.42198600 | 0.35916400 |
| O | 4.22991000 | 2.67315100 | -0.84629900 |
| C | 4.92045300 | 3.29428700 | 1.36399800 |
| H | 4.82686100 | 2.94768800 | 2.39396100 |
| H | 5.97768100 | 3.32948000 | 1.09337700 |
| H | 4.52473100 | 4.30955900 | 1.29125600 |
| C | 3.39736000 | -0.97993700 | 0.11196300 |
| H | 3.41733000 | -1.38074100 | 1.14054100 |
| O | 4.67643500 | -0.91096600 | -0.41458200 |
| C | 5.45687000 | -2.09682300 | -0.21435900 |
| H | 5.00737500 | -2.95257400 | -0.72416900 |
| H | 5.55519200 | -2.31693500 | 0.85477900 |
| H | 6.44059400 | -1.89443700 | -0.63513000 |
| O | 2.62451800 | -1.85354000 | -0.70738900 |
| C | 1.30171300 | -2.10595800 | -0.22748300 |
| H | 1.33628600 | -2.56620600 | 0.76943500 |
| C | 0.74113800 | -3.09569900 | -1.24560500 |
| H | 1.39839300 | -3.97210000 | -1.26060600 |
| C | -3.02217800 | -1.00280700 | 1.00298700 |
| H | -2.83645900 | -0.39921400 | 1.89922900 |
| C | -4.34958200 | -0.56940100 | 0.37371700 |
| H | -4.55847700 | -1.22223900 | -0.48115400 |
| C | -4.31439700 | 0.89059700 | -0.10115600 |
| H | -5.21281600 | 1.09512800 | -0.69738900 |
| O | -3.05085200 | -2.38713000 | 1.32205100 |
| H | -3.85238900 | -2.53737000 | 1.83929300 |
| O | -0.59166800 | -3.45338800 | -0.89043400 |
| H | -0.91736600 | -4.08190000 | -1.54318700 |
| H | 0.77259100 | -2.62378500 | -2.23550600 |
| O | -5.34687200 | -0.74891700 | 1.38802500 |
| H | -6.20470000 | -0.89020800 | 0.97138300 |
| O | -4.24106000 | 1.78499300 | 1.00443400 |
| H | -4.88221200 | 1.47915300 | 1.65951800 |
| F | -3.93772000 | 3.00587500 | -2.11893400 |
| E(RB3LYP): -1494.60024186 | | | |
| Sum of electronic and thermal free energies: -1494.226472 | | | |
| Number of imaginary frequencies: 0 | | | |

## 6′F-LN **8** Conformer L

| **Atom** | **x** | **y** | **z** |
| --- | --- | --- | --- |
| C | 2.78297500 | 2.43822800 | 1.78865400 |
| H | 1.97022600 | 2.34867100 | 2.51149800 |
| H | 3.67214200 | 2.84576600 | 2.27370700 |
| C | 3.08693700 | 1.07863700 | 1.19086200 |
| H | 3.26764100 | 0.41703300 | 2.05262300 |
| O | 1.92125200 | 0.64166100 | 0.49013500 |
| C | 1.95667200 | -0.72812700 | 0.06073800 |
| H | 2.05574700 | -1.38576100 | 0.93500700 |
| O | 0.76816000 | -0.96727200 | -0.61477200 |
| C | -0.44624000 | -0.83410900 | 0.15182900 |
| H | -0.21746400 | -0.56264300 | 1.18699400 |
| C | -1.22998500 | 0.31668300 | -0.49947600 |
| H | -1.19301700 | 0.14146600 | -1.58558100 |
| O | -0.65152400 | 1.58055600 | -0.19808500 |
| H | 0.30916700 | 1.46642300 | -0.10073900 |
| C | -2.69830000 | 0.35617100 | -0.06501600 |
| H | -2.76297700 | 0.63952400 | 0.98590000 |
| N | -3.43183400 | 1.34624200 | -0.83098800 |
| H | -3.39340200 | 1.28393600 | -1.83797400 |
| C | -4.14210300 | 2.36221700 | -0.27795800 |
| O | -4.23007500 | 2.53528300 | 0.93846800 |
| C | -4.82914600 | 3.29340800 | -1.25567900 |
| H | -4.69769500 | 3.00786300 | -2.30014900 |
| H | -5.89548000 | 3.31538500 | -1.02224200 |
| H | -4.43583900 | 4.30169700 | -1.10904800 |
| C | -3.31125600 | -1.04104300 | -0.20909300 |
| H | -3.29026400 | -1.37667100 | -1.26070000 |
| O | -4.60909200 | -1.01849500 | 0.27407300 |
| C | -5.36859300 | -2.19493500 | -0.03297400 |
| H | -4.92671500 | -3.07944200 | 0.43255900 |
| H | -5.42845900 | -2.34231800 | -1.11738300 |
| H | -6.36821500 | -2.03097700 | 0.36655800 |
| O | -2.55715100 | -1.95737000 | 0.58075400 |
| C | -1.21648200 | -2.16516400 | 0.13041300 |
| H | -1.21277700 | -2.55920900 | -0.89501300 |
| C | -0.67599300 | -3.21419500 | 1.09889900 |
| H | -1.32174400 | -4.09695300 | 1.03443100 |
| C | 3.12424900 | -0.90847400 | -0.90775000 |
| H | 2.92828600 | -0.26861000 | -1.77618400 |
| C | 4.42523800 | -0.45617900 | -0.23869000 |
| H | 4.64842100 | -1.13821500 | 0.58925300 |
| C | 4.32585600 | 0.98222300 | 0.29705100 |
| H | 5.20933500 | 1.18961500 | 0.91684700 |
| O | 3.20930000 | -2.27348900 | -1.29351400 |
| H | 4.02459400 | -2.36949200 | -1.80213000 |
| O | 0.67238300 | -3.53317600 | 0.76491800 |
| H | 0.98493700 | -4.19914100 | 1.38620700 |
| H | -0.74518800 | -2.80975200 | 2.11638600 |
| O | 5.44224900 | -0.55200900 | -1.24429700 |
| H | 6.29865700 | -0.68149000 | -0.82099900 |
| O | 4.24718900 | 1.91229800 | -0.77574900 |
| H | 4.90707100 | 1.63816300 | -1.42716100 |
| F | 2.35904000 | 3.35839000 | 0.81310500 |
| E(RB3LYP): -1494.59839018 | | | |
| Sum of electronic and thermal free energies: -1494.225243 | | | |
| Number of imaginary frequencies: 0 | | | |

## GlcNAcβ-OMe (implicit DMSO solvation)

| **Atom** | **x** | **y** | **z** |
| --- | --- | --- | --- |
| C | -1.61569100 | -1.17951300 | -0.02390800 |
| H | -1.68992300 | -1.23287000 | 1.06691300 |
| C | -0.15503900 | -1.39108800 | -0.42058400 |
| H | -0.11683100 | -1.45958000 | -1.51862500 |
| O | 0.36211100 | -2.58776700 | 0.15113100 |
| H | -0.22043100 | -3.30739200 | -0.12304600 |
| C | 0.72161700 | -0.22382000 | 0.04000300 |
| H | 0.76513000 | -0.21105600 | 1.12986700 |
| N | 2.07980700 | -0.36579500 | -0.44818800 |
| H | 2.21262200 | -0.47568500 | -1.44290900 |
| C | 3.17669700 | -0.37482500 | 0.35208500 |
| O | 3.11320200 | -0.26739600 | 1.57729900 |
| C | 4.50680100 | -0.52874400 | -0.35606900 |
| H | 4.41930800 | -0.61927600 | -1.43946600 |
| H | 5.12702700 | 0.33828700 | -0.11922400 |
| H | 5.00938700 | -1.41448800 | 0.03781900 |
| C | 0.10239500 | 1.10627400 | -0.42016400 |
| H | 0.11279500 | 1.17636800 | -1.52240200 |
| O | 0.82577800 | 2.14631400 | 0.14215300 |
| C | 0.53174300 | 3.43342100 | -0.41570700 |
| H | -0.50940100 | 3.71141500 | -0.23383500 |
| H | 0.73011400 | 3.44039500 | -1.49363100 |
| H | 1.19255200 | 4.14351100 | 0.07917700 |
| O | -1.24600800 | 1.20341000 | 0.03148300 |
| C | -2.10701000 | 0.19356400 | -0.50259800 |
| H | -2.07445800 | 0.21177800 | -1.60300500 |
| C | -3.53374400 | 0.53126600 | -0.08769800 |
| H | -3.75875100 | 1.53903100 | -0.44223600 |
| H | -4.22075500 | -0.16457900 | -0.57862100 |
| O | -3.73067300 | 0.54316400 | 1.32847300 |
| H | -3.97498500 | -0.33789100 | 1.62775100 |
| O | -2.36166700 | -2.23913800 | -0.63705700 |
| H | -3.03060500 | -2.56530500 | -0.02721800 |
| E(RB3LYP): -859.59841881 | | | |
| Sum of electronic and thermal free energies-859.370233: | | | |
| Number of imaginary frequencies: 0 | | | |

## GlcNAcβ-OMe (explicit monodentate DMSO solvation)

| **Atom** | **x** | **y** | **z** |
| --- | --- | --- | --- |
| C | 1.95178100 | -1.42536700 | -0.61187500 |
| H | 2.46902300 | -1.10514500 | -1.52212000 |
| C | 0.53287600 | -0.86225600 | -0.63381100 |
| H | -0.00541800 | -1.27916200 | 0.23027500 |
| O | -0.14178300 | -1.22712900 | -1.83586000 |
| H | -0.05313400 | -2.18499600 | -1.92628700 |
| C | 0.53502700 | 0.66489100 | -0.52286600 |
| H | 0.99480900 | 1.09738000 | -1.41280900 |
| N | -0.81645300 | 1.17355300 | -0.41265400 |
| H | -1.43022800 | 0.72373400 | 0.27299200 |
| C | -1.32750900 | 2.12707500 | -1.22169300 |
| O | -0.69000400 | 2.67269300 | -2.12880600 |
| C | -2.77282900 | 2.49719100 | -0.94932800 |
| H | -3.19136400 | 1.97813900 | -0.08646400 |
| H | -2.83251300 | 3.57542700 | -0.78792700 |
| H | -3.36727600 | 2.26195400 | -1.83549000 |
| C | 1.37850200 | 1.07947000 | 0.69426200 |
| H | 0.90516200 | 0.72101900 | 1.62539500 |
| O | 1.49512800 | 2.46085000 | 0.70505800 |
| C | 2.01671600 | 2.99449300 | 1.92822400 |
| H | 3.03770600 | 2.64671300 | 2.10488100 |
| H | 1.38132300 | 2.70981200 | 2.77487100 |
| H | 2.01241800 | 4.07818600 | 1.82039000 |
| O | 2.68918400 | 0.52314500 | 0.60342700 |
| C | 2.71053600 | -0.90628500 | 0.61723300 |
| H | 2.20756100 | -1.28417100 | 1.52112300 |
| C | 4.16657100 | -1.35000300 | 0.69173200 |
| H | 4.61397200 | -0.89230200 | 1.57613900 |
| H | 4.20194800 | -2.43667100 | 0.81565700 |
| O | 4.95292200 | -0.92883300 | -0.42595400 |
| H | 4.89483500 | -1.58539000 | -1.12679100 |
| O | 1.82136500 | -2.85329200 | -0.57764600 |
| H | 2.49322800 | -3.25715900 | -1.13545900 |
| S | -3.26830900 | -1.50549200 | 0.90617500 |
| O | -2.42438800 | -0.30164100 | 1.38077200 |
| C | -4.98270200 | -1.12823800 | 1.39223700 |
| H | -5.23936000 | -0.13143100 | 1.03198600 |
| H | -5.63879200 | -1.88568600 | 0.96131000 |
| H | -5.02449900 | -1.16893200 | 2.47986300 |
| C | -3.46756000 | -1.30227800 | -0.89497200 |
| H | -4.08405800 | -2.12282900 | -1.26458900 |
| H | -3.93592300 | -0.33918200 | -1.09894400 |
| H | -2.46916400 | -1.34481000 | -1.33101400 |
| E(RB3LYP): -1412.90213416 | | | |
| Sum of electronic and thermal free energies: -1412.603879 | | | |
| Number of imaginary frequencies: 0 | | | |
|  | | | |

## GlcNAcβ-OMe (explicit bidentate DMSO solvation)

| **Atom** | **x** | **y** | **z** |
| --- | --- | --- | --- |
|  |  |  |  |
| C | -1.91768500 | -1.55420000 | 0.44541600 |
| H | -2.28389500 | -1.32537100 | 1.45505400 |
| C | -0.54641300 | -0.91061200 | 0.27670000 |
| H | -0.16832400 | -1.17318000 | -0.72089800 |
| O | 0.32595500 | -1.45614100 | 1.25723700 |
| H | 1.22322800 | -1.41555500 | 0.86894900 |
| C | -0.66453000 | 0.61981500 | 0.37903000 |
| H | -1.04206200 | 0.89732300 | 1.36550700 |
| N | 0.63681200 | 1.23868300 | 0.19666700 |
| H | 1.33273700 | 0.70862500 | -0.32237100 |
| C | 1.09812300 | 2.23093200 | 1.00046700 |
| O | 0.41564500 | 2.77528500 | 1.87002900 |
| C | 2.53939300 | 2.63962800 | 0.76668500 |
| H | 2.95764300 | 2.23424100 | -0.15473400 |
| H | 2.59473800 | 3.72870100 | 0.73845900 |
| H | 3.14031000 | 2.29724700 | 1.61310400 |
| C | -1.69284600 | 1.09346000 | -0.66149800 |
| H | -1.34900500 | 0.83934700 | -1.67990600 |
| O | -1.88469000 | 2.46042600 | -0.53999000 |
| C | -2.61560900 | 3.04510700 | -1.62467200 |
| H | -3.63122900 | 2.64457400 | -1.67594400 |
| H | -2.10319100 | 2.86448700 | -2.57686500 |
| H | -2.65375300 | 4.11631100 | -1.43168000 |
| O | -2.94610200 | 0.44482200 | -0.42598100 |
| C | -2.89549800 | -0.97580300 | -0.58081200 |
| H | -2.53422300 | -1.22903400 | -1.58957900 |
| C | -4.31096800 | -1.52698700 | -0.45646800 |
| H | -4.95678100 | -0.96707400 | -1.13535800 |
| H | -4.30528300 | -2.57528900 | -0.76927300 |
| O | -4.87621200 | -1.38700800 | 0.84891600 |
| H | -4.53051400 | -2.08777300 | 1.41212500 |
| O | -1.84974300 | -2.96496100 | 0.25240200 |
| H | -1.09753300 | -3.27959800 | 0.77111800 |
| S | 3.88981500 | -1.48627800 | -0.51238600 |
| O | 2.43932800 | -0.96467200 | -0.37961400 |
| C | 4.60091800 | -0.55983900 | -1.90763900 |
| H | 4.44382600 | 0.50654600 | -1.74385300 |
| H | 5.66325700 | -0.79789300 | -1.97410000 |
| H | 4.08403900 | -0.89421900 | -2.80595500 |
| C | 4.82664400 | -0.69628400 | 0.83494200 |
| H | 5.88012800 | -0.95406600 | 0.71938500 |
| H | 4.67719600 | 0.38202000 | 0.79282300 |
| H | 4.44134100 | -1.10400700 | 1.76859700 |
|  |  |  |  |
|  |  |  |  |
|  |  |  |  |
| E(RB3LYP): -1412.90585148 | | | |
| Sum of electronic and thermal free energies. -1412.606361 | | | |
| Number of imaginary frequencies: 0 | | | |

## 3F-LN **3** Geometry for Log *P* calculation

| **Atom** | **x** | **y** | **z** |
| --- | --- | --- | --- |
| H | 3.89730000 | 4.45440000 | -0.90680000 |
| O | 3.85520000 | 3.68270000 | -0.33280000 |
| C | 2.92500000 | 2.75160000 | -0.89210000 |
| H | 3.18520000 | 2.50130000 | -1.92450000 |
| H | 1.90370000 | 3.14670000 | -0.86740000 |
| C | 2.97210000 | 1.49260000 | -0.03920000 |
| H | 2.74700000 | 1.76660000 | 1.00160000 |
| O | 1.94480000 | 0.63330000 | -0.54330000 |
| C | 1.78160000 | -0.54020000 | 0.24060000 |
| H | 1.53140000 | -0.26990000 | 1.27590000 |
| O | 0.75940000 | -1.30050000 | -0.33430000 |
| C | -0.57360000 | -1.09790000 | 0.14440000 |
| H | -0.63420000 | -1.37040000 | 1.20370000 |
| C | -1.06790000 | 0.33610000 | -0.01910000 |
| H | -0.73640000 | 0.73650000 | -0.98000000 |
| C | -2.58270000 | 0.46360000 | 0.15940000 |
| H | -2.82930000 | 0.26430000 | 1.20310000 |
| N | -3.02560000 | 1.80910000 | -0.14150000 |
| H | -2.85990000 | 2.16740000 | -1.07080000 |
| C | -3.68210000 | 2.60200000 | 0.74860000 |
| O | -3.93230000 | 2.24650000 | 1.89940000 |
| C | -4.09050000 | 3.96660000 | 0.23600000 |
| H | -3.78990000 | 4.15540000 | -0.79510000 |
| H | -5.17640000 | 4.05330000 | 0.31290000 |
| H | -3.64920000 | 4.72700000 | 0.88320000 |
| C | -3.31560000 | -0.58860000 | -0.68540000 |
| H | -3.15350000 | -0.41770000 | -1.76350000 |
| O | -4.66160000 | -0.54030000 | -0.37370000 |
| C | -5.49820000 | -1.29110000 | -1.26540000 |
| H | -5.25530000 | -2.35560000 | -1.22540000 |
| H | -5.38920000 | -0.92690000 | -2.29300000 |
| H | -6.52250000 | -1.13510000 | -0.93110000 |
| O | -2.81690000 | -1.88220000 | -0.34790000 |
| C | -1.44080000 | -2.06740000 | -0.67630000 |
| H | -1.27220000 | -1.87530000 | -1.74520000 |
| C | -1.11970000 | -3.52900000 | -0.39100000 |
| H | -1.75700000 | -4.15150000 | -1.02890000 |
| H | -0.07760000 | -3.72560000 | -0.64070000 |
| O | -1.28870000 | -3.86510000 | 0.98470000 |
| H | -2.20900000 | -3.69330000 | 1.21880000 |
| C | 3.06530000 | -1.36950000 | 0.20130000 |
| H | 3.25110000 | -1.65970000 | -0.83840000 |
| C | 4.23100000 | -0.53020000 | 0.71470000 |
| H | 4.05630000 | -0.30620000 | 1.77560000 |
| O | 5.47490000 | -1.21520000 | 0.56170000 |
| H | 5.35770000 | -2.11920000 | 0.87940000 |
| C | 4.33420000 | 0.79030000 | -0.05390000 |
| H | 5.06160000 | 1.43790000 | 0.44860000 |
| O | 4.73010000 | 0.56460000 | -1.40600000 |
| H | 5.46430000 | -0.06250000 | -1.38360000 |
| O | 2.97420000 | -2.51680000 | 1.04300000 |
| H | 2.31710000 | -3.11540000 | 0.66920000 |
| F | -0.46930000 | 1.14660000 | 0.97460000 |
| E(RB3LYP): -1494.60345697 | | | |
| Sum of electronic and thermal free energies: -1494.230878 | | | |
| Number of imaginary frequencies: 0 | | | |

## 6F-LN **4** Geometry for Log *P* calculation

| **Atom** | **x** | **y** | **z** |
| --- | --- | --- | --- |
| H | 0.88470000 | 2.61710000 | 1.45520000 |
| O | 1.63310000 | 3.01920000 | 1.91470000 |
| C | 2.80780000 | 2.81210000 | 1.12870000 |
| H | 3.63560000 | 3.26630000 | 1.67530000 |
| H | 2.72270000 | 3.30170000 | 0.15370000 |
| C | 3.08780000 | 1.33130000 | 0.92750000 |
| H | 3.10330000 | 0.83910000 | 1.90930000 |
| O | 1.97880000 | 0.81720000 | 0.16810000 |
| C | 1.99580000 | -0.59890000 | 0.01180000 |
| H | 1.99160000 | -1.07850000 | 1.00260000 |
| O | 0.86700000 | -0.95880000 | -0.72090000 |
| C | -0.39320000 | -0.83080000 | -0.03580000 |
| H | -0.22640000 | -0.60070000 | 1.01990000 |
| C | -1.19940000 | 0.30200000 | -0.67850000 |
| H | -1.20790000 | 0.11980000 | -1.76380000 |
| O | -0.63290000 | 1.57880000 | -0.41190000 |
| H | 0.32970000 | 1.49850000 | -0.51130000 |
| C | -2.64280000 | 0.31410000 | -0.15820000 |
| H | -2.64140000 | 0.60690000 | 0.89250000 |
| N | -3.44100000 | 1.28590000 | -0.87960000 |
| H | -3.47600000 | 1.21090000 | -1.88580000 |
| C | -4.11630000 | 2.30450000 | -0.28640000 |
| O | -4.11320000 | 2.49070000 | 0.93070000 |
| C | -4.88410000 | 3.21660000 | -1.22050000 |
| H | -4.81210000 | 2.93080000 | -2.27060000 |
| H | -5.93410000 | 3.21470000 | -0.92070000 |
| H | -4.50580000 | 4.23390000 | -1.10010000 |
| C | -3.26090000 | -1.08990000 | -0.24960000 |
| H | -3.35760000 | -1.40750000 | -1.30240000 |
| O | -4.49570000 | -1.07160000 | 0.37400000 |
| C | -5.29680000 | -2.23510000 | 0.12600000 |
| H | -4.81610000 | -3.13310000 | 0.52170000 |
| H | -5.47560000 | -2.35690000 | -0.94830000 |
| H | -6.24480000 | -2.07140000 | 0.63580000 |
| O | -2.42920000 | -2.03240000 | 0.43300000 |
| C | -1.13630000 | -2.16720000 | -0.14960000 |
| H | -1.21840000 | -2.44370000 | -1.21200000 |
| C | -0.41550000 | -3.30040000 | 0.54430000 |
| H | -1.03970000 | -4.19430000 | 0.56290000 |
| H | 0.53500000 | -3.50680000 | 0.05230000 |
| C | 3.23890000 | -0.99390000 | -0.78270000 |
| H | 3.16960000 | -0.52480000 | -1.76940000 |
| C | 4.48930000 | -0.48350000 | -0.06540000 |
| H | 4.57910000 | -1.01050000 | 0.89380000 |
| O | 5.65780000 | -0.70190000 | -0.85260000 |
| H | 5.63020000 | -1.60940000 | -1.18130000 |
| C | 4.40340000 | 1.02390000 | 0.21200000 |
| H | 5.23420000 | 1.30210000 | 0.87310000 |
| O | 4.46340000 | 1.77480000 | -0.99760000 |
| H | 5.18560000 | 1.40820000 | -1.52420000 |
| O | 3.37240000 | -2.40780000 | -0.89530000 |
| H | 2.73200000 | -2.72660000 | -1.54130000 |
| F | -0.12980000 | -2.95370000 | 1.88450000 |
| E(RB3LYP): -1494.61188344 | | | |
| Sum of electronic and thermal free energies: -1494.238213 | | | |
| Number of imaginary frequencies: 0 | | | |

## 2′F-LN **5** Geometry for Log *P* calculation

| **Atom** | **x** | **y** | **z** |
| --- | --- | --- | --- |
| H | 0.88200000 | 2.59760000 | 1.43670000 |
| O | 1.62470000 | 2.99190000 | 1.91250000 |
| C | 2.80990000 | 2.79610000 | 1.14040000 |
| H | 3.62970000 | 3.24580000 | 1.70250000 |
| H | 2.73710000 | 3.29490000 | 0.16920000 |
| C | 3.09460000 | 1.31730000 | 0.92860000 |
| H | 3.09930000 | 0.81530000 | 1.90520000 |
| O | 1.99640000 | 0.81180000 | 0.14830000 |
| C | 1.99010000 | -0.60410000 | -0.00910000 |
| H | 1.96310000 | -1.08720000 | 0.97940000 |
| O | 0.87400000 | -0.93960000 | -0.76310000 |
| C | -0.39090000 | -0.82640000 | -0.08000000 |
| H | -0.22610000 | -0.61270000 | 0.97940000 |
| C | -1.19640000 | 0.31410000 | -0.70570000 |
| H | -1.21590000 | 0.14550000 | -1.79300000 |
| O | -0.62550000 | 1.58840000 | -0.42980000 |
| H | 0.33400000 | 1.51000000 | -0.55090000 |
| C | -2.63380000 | 0.32140000 | -0.17000000 |
| H | -2.62030000 | 0.59990000 | 0.88460000 |
| N | -3.43890000 | 1.30370000 | -0.86960000 |
| H | -3.48140000 | 1.24390000 | -1.87650000 |
| C | -4.11020000 | 2.31250000 | -0.25590000 |
| O | -4.09850000 | 2.48010000 | 0.96390000 |
| C | -4.88590000 | 3.23810000 | -1.17010000 |
| H | -4.81270000 | 2.97530000 | -2.22610000 |
| H | -5.93570000 | 3.22150000 | -0.86970000 |
| H | -4.51570000 | 4.25560000 | -1.02830000 |
| C | -3.25590000 | -1.08040000 | -0.27460000 |
| H | -3.36970000 | -1.37880000 | -1.33120000 |
| O | -4.48240000 | -1.06830000 | 0.36710000 |
| C | -5.29120000 | -2.22400000 | 0.10920000 |
| H | -4.80810000 | -3.13120000 | 0.48050000 |
| H | -5.48640000 | -2.32500000 | -0.96440000 |
| H | -6.23110000 | -2.06710000 | 0.63600000 |
| O | -2.41870000 | -2.03760000 | 0.37720000 |
| C | -1.12280000 | -2.16460000 | -0.21310000 |
| H | -1.20880000 | -2.42880000 | -1.27700000 |
| C | -0.41380000 | -3.29990000 | 0.51180000 |
| H | -0.99240000 | -4.21970000 | 0.37180000 |
| H | 0.57630000 | -3.44130000 | 0.07760000 |
| O | -0.21940000 | -3.02340000 | 1.89890000 |
| H | -1.08780000 | -2.88150000 | 2.29520000 |
| C | 3.24930000 | -0.99540000 | -0.77220000 |
| H | 3.19300000 | -0.61450000 | -1.79270000 |
| C | 4.50240000 | -0.49260000 | -0.05980000 |
| H | 4.58930000 | -1.01940000 | 0.89880000 |
| O | 5.66890000 | -0.70410000 | -0.84800000 |
| H | 5.74270000 | -1.64240000 | -1.05880000 |
| C | 4.41420000 | 1.01610000 | 0.21920000 |
| H | 5.24430000 | 1.29060000 | 0.88190000 |
| O | 4.47200000 | 1.76760000 | -0.98900000 |
| H | 5.22930000 | 1.44630000 | -1.49480000 |
| F | 3.32840000 | -2.40250000 | -0.84940000 |
| E(RB3LYP): -1494.60980005 | | | |
| Sum of electronic and thermal free energies: -1494.236813 | | | |
| Number of imaginary frequencies: 0 | | | |

## 3′F-LN **6** Geometry for Log *P* calculation

| **Atom** | **x** | **y** | **z** |
| --- | --- | --- | --- |
| H | 0.93260000 | 2.60110000 | 1.48810000 |
| O | 1.68700000 | 2.97350000 | 1.96250000 |
| C | 2.85920000 | 2.77040000 | 1.17320000 |
| H | 3.69320000 | 3.19780000 | 1.73170000 |
| H | 2.78300000 | 3.28400000 | 0.21040000 |
| C | 3.11530000 | 1.28970000 | 0.93750000 |
| H | 3.12420000 | 0.77550000 | 1.90770000 |
| O | 2.00470000 | 0.80930000 | 0.16290000 |
| C | 2.00230000 | -0.60370000 | -0.00610000 |
| H | 1.98640000 | -1.09200000 | 0.98020000 |
| O | 0.87640000 | -0.94430000 | -0.74810000 |
| C | -0.38600000 | -0.82150000 | -0.06090000 |
| H | -0.21540000 | -0.59820000 | 0.99550000 |
| C | -1.18610000 | 0.31840000 | -0.69560000 |
| H | -1.19780000 | 0.14540000 | -1.78240000 |
| O | -0.61470000 | 1.59220000 | -0.41980000 |
| H | 0.34690000 | 1.51100000 | -0.52170000 |
| C | -2.62790000 | 0.33210000 | -0.17210000 |
| H | -2.62290000 | 0.61190000 | 0.88220000 |
| N | -3.42330000 | 1.31630000 | -0.87990000 |
| H | -3.45620000 | 1.25670000 | -1.88720000 |
| C | -4.09680000 | 2.32760000 | -0.27270000 |
| O | -4.09550000 | 2.49550000 | 0.94710000 |
| C | -4.86110000 | 3.25570000 | -1.19390000 |
| H | -4.78230000 | 2.99030000 | -2.24890000 |
| H | -5.91290000 | 3.24560000 | -0.90050000 |
| H | -4.48610000 | 4.27130000 | -1.05160000 |
| C | -3.25180000 | -1.06790000 | -0.27960000 |
| H | -3.34940000 | -1.37250000 | -1.33610000 |
| O | -4.48780000 | -1.05120000 | 0.34320000 |
| C | -5.29260000 | -2.20890000 | 0.08170000 |
| H | -4.81530000 | -3.11310000 | 0.46730000 |
| H | -5.47120000 | -2.31810000 | -0.99400000 |
| H | -6.24050000 | -2.04800000 | 0.59270000 |
| O | -2.42550000 | -2.02140000 | 0.39110000 |
| C | -1.12180000 | -2.15950000 | -0.17890000 |
| H | -1.19260000 | -2.44340000 | -1.23880000 |
| C | -0.43050000 | -3.28340000 | 0.58110000 |
| H | 0.57060000 | -3.43410000 | 0.17660000 |
| C | 3.24170000 | -1.03240000 | -0.80150000 |
| H | 3.17990000 | -0.56990000 | -1.79210000 |
| C | 4.48540000 | -0.52200000 | -0.08990000 |
| H | 4.65190000 | -1.08750000 | 0.83040000 |
| C | 4.42390000 | 0.98130000 | 0.20700000 |
| H | 5.26470000 | 1.24360000 | 0.85990000 |
| O | 4.46550000 | 1.74350000 | -0.99440000 |
| H | 5.27940000 | 1.52200000 | -1.46240000 |
| O | 3.32530000 | -2.44700000 | -0.88980000 |
| H | 2.60610000 | -2.75320000 | -1.45500000 |
| F | 5.61060000 | -0.75370000 | -0.91400000 |
| O | -0.27190000 | -2.98490000 | 1.96770000 |
| H | -1.15000000 | -2.82980000 | 2.33700000 |
| H | -1.00580000 | -4.20520000 | 0.44080000 |
| E(RB3LYP): -1494.61059723 | | | |
| Sum of electronic and thermal free energies: -1494.236894 | | | |
| Number of imaginary frequencies: 0 | | | |

## 4′F-LN **7** Geometry for Log *P* calculation

| **Atom** | **x** | **y** | **z** |
| --- | --- | --- | --- |
| H | 0.91140000 | 2.63170000 | 1.43660000 |
| O | 1.65890000 | 3.02060000 | 1.90910000 |
| C | 2.83960000 | 2.80830000 | 1.13760000 |
| H | 3.66580000 | 3.24950000 | 1.69700000 |
| H | 2.77130000 | 3.30430000 | 0.16400000 |
| C | 3.10420000 | 1.32420000 | 0.93070000 |
| H | 3.12460000 | 0.82710000 | 1.91010000 |
| O | 1.99920000 | 0.82510000 | 0.16300000 |
| C | 2.00500000 | -0.59220000 | -0.01240000 |
| H | 1.99000000 | -1.08260000 | 0.97250000 |
| O | 0.88030000 | -0.92890000 | -0.75660000 |
| C | -0.38170000 | -0.81740000 | -0.06800000 |
| H | -0.21180000 | -0.59060000 | 0.98780000 |
| C | -1.19380000 | 0.31320000 | -0.70290000 |
| H | -1.21120000 | 0.13600000 | -1.78890000 |
| O | -0.62920000 | 1.59230000 | -0.43630000 |
| H | 0.33130000 | 1.51750000 | -0.55050000 |
| C | -2.63190000 | 0.31780000 | -0.16960000 |
| H | -2.62190000 | 0.60260000 | 0.88330000 |
| N | -3.44020000 | 1.29210000 | -0.87660000 |
| H | -3.47920000 | 1.22760000 | -1.88330000 |
| C | -4.11710000 | 2.30130000 | -0.26980000 |
| O | -4.10930000 | 2.47520000 | 0.94910000 |
| C | -4.89410000 | 3.21910000 | -1.19080000 |
| H | -4.81900000 | 2.94950000 | -2.24500000 |
| H | -5.94410000 | 3.20200000 | -0.89160000 |
| H | -4.52640000 | 4.23840000 | -1.05530000 |
| C | -3.24560000 | -1.08790000 | -0.26680000 |
| H | -3.35070000 | -1.39600000 | -1.32150000 |
| O | -4.47610000 | -1.07820000 | 0.36710000 |
| C | -5.27580000 | -2.24130000 | 0.11450000 |
| H | -4.78950000 | -3.14190000 | 0.49730000 |
| H | -5.46320000 | -2.35340000 | -0.95940000 |
| H | -6.22020000 | -2.08560000 | 0.63350000 |
| O | -2.40650000 | -2.03400000 | 0.39860000 |
| C | -1.10640000 | -2.16140000 | -0.18250000 |
| H | -1.18410000 | -2.44400000 | -1.24220000 |
| C | -0.39760000 | -3.28050000 | 0.56810000 |
| H | 0.59970000 | -3.42190000 | 0.15050000 |
| C | 3.25090000 | -0.99240000 | -0.80180000 |
| H | 3.18980000 | -0.53080000 | -1.79290000 |
| C | 4.50290000 | -0.48610000 | -0.08010000 |
| H | 4.56020000 | -0.99180000 | 0.89480000 |
| C | 4.40930000 | 1.00840000 | 0.20570000 |
| H | 5.27260000 | 1.34000000 | 0.78620000 |
| O | 3.37800000 | -2.40660000 | -0.89950000 |
| H | 2.72750000 | -2.73130000 | -1.53270000 |
| O | -0.22420000 | -2.98240000 | 1.95310000 |
| H | -1.09890000 | -2.83570000 | 2.33380000 |
| H | -0.96700000 | -4.20690000 | 0.43380000 |
| F | 4.43740000 | 1.72110000 | -1.01370000 |
| O | 5.67940000 | -0.74160000 | -0.83090000 |
| H | 5.67590000 | -1.67910000 | -1.06230000 |
| E(RB3LYP): -1494.61118141 | | | |
| Sum of electronic and thermal free energies: -1494.238375 | | | |
| Number of imaginary frequencies: 0 | | | |

## 6′F-LN **8** Geometry for Log *P* calculation

| **Atom** | **x** | **y** | **z** |
| --- | --- | --- | --- |
| C | 3.07590000 | 2.97820000 | 0.48910000 |
| H | 3.94230000 | 3.47620000 | 0.92690000 |
| H | 2.97240000 | 3.24550000 | -0.56170000 |
| C | 3.17640000 | 1.47980000 | 0.66320000 |
| H | 3.11400000 | 1.24130000 | 1.73310000 |
| O | 2.05520000 | 0.89960000 | -0.02160000 |
| C | 1.97320000 | -0.52580000 | 0.11140000 |
| H | 1.90180000 | -0.79040000 | 1.17690000 |
| O | 0.85690000 | -0.96270000 | -0.58650000 |
| C | -0.42560000 | -0.85220000 | 0.06320000 |
| H | -0.29050000 | -0.77750000 | 1.14660000 |
| C | -1.16730000 | 0.39170000 | -0.44310000 |
| H | -1.08210000 | 0.39210000 | -1.54050000 |
| O | -0.63330000 | 1.59400000 | 0.09090000 |
| H | 0.33660000 | 1.56790000 | 0.00870000 |
| C | -2.65370000 | 0.35340000 | -0.05820000 |
| H | -2.74590000 | 0.47290000 | 1.02200000 |
| N | -3.37060000 | 1.45000000 | -0.68020000 |
| H | -3.29970000 | 1.54810000 | -1.68240000 |
| C | -4.09830000 | 2.36910000 | 0.00460000 |
| O | -4.22360000 | 2.34860000 | 1.22970000 |
| C | -4.75980000 | 3.44230000 | -0.83550000 |
| H | -4.55980000 | 3.34890000 | -1.90370000 |
| H | -5.83810000 | 3.39720000 | -0.66890000 |
| H | -4.41040000 | 4.41740000 | -0.49000000 |
| C | -3.27410000 | -0.99940000 | -0.42870000 |
| H | -3.25670000 | -1.15400000 | -1.52180000 |
| O | -4.57100000 | -1.04400000 | 0.05410000 |
| C | -5.34600000 | -2.14210000 | -0.44520000 |
| H | -4.91580000 | -3.09800000 | -0.13640000 |
| H | -5.40840000 | -2.10350000 | -1.53860000 |
| H | -6.34300000 | -2.03470000 | -0.02080000 |
| O | -2.52980000 | -2.04830000 | 0.19080000 |
| C | -1.17990000 | -2.14700000 | -0.26550000 |
| H | -1.15300000 | -2.30880000 | -1.35270000 |
| C | -0.58640000 | -3.36790000 | 0.42620000 |
| H | 0.45460000 | -3.48690000 | 0.12780000 |
| C | 3.21570000 | -1.14900000 | -0.52430000 |
| H | 3.22850000 | -0.87100000 | -1.58290000 |
| C | 4.46720000 | -0.60550000 | 0.16090000 |
| H | 4.45890000 | -0.92950000 | 1.21030000 |
| C | 4.49710000 | 0.92620000 | 0.11940000 |
| H | 5.31330000 | 1.27680000 | 0.76350000 |
| O | 3.23790000 | -2.56410000 | -0.36600000 |
| H | 2.62140000 | -2.95200000 | -0.99690000 |
| O | -0.59350000 | -3.24180000 | 1.84780000 |
| H | -1.50800000 | -3.10260000 | 2.12350000 |
| H | -1.14610000 | -4.25490000 | 0.10890000 |
| O | 5.65220000 | -1.06550000 | -0.48320000 |
| H | 5.55620000 | -2.01370000 | -0.63890000 |
| O | 4.67870000 | 1.40330000 | -1.21080000 |
| H | 5.39680000 | 0.88940000 | -1.60280000 |
| F | 1.92580000 | 3.44330000 | 1.16270000 |
| E(RB3LYP): -1494.61260434 | | | |
| Sum of electronic and thermal free energies: -1494.238514 | | | |
| Number of imaginary frequencies: 0 | | | |
